# Trends in the global burden of aortic valve calcification disease in the working-age population from 1992 to 2021

**DOI:** 10.3389/fcvm.2025.1544273

**Published:** 2025-08-12

**Authors:** Songzhe Wu, Weiguang Yang, Yixin Li, Tao Wei, Yaodong Sun, Lin Xia, Yan Zhu, Naishi Wu

**Affiliations:** ^1^ Department of Cardiovascular Surgery, General Hospital of Tianjin Medical University, Tianjin, China; ^2^ School of Medical Technology, Shaanxi University of Chinese Medicine, Xianyang, China; ^3^ Department of Cardiovascular Surgery, General Hospital of Northern Theater Command, Shenyang, China

**Keywords:** global burden of disease, calcific aortic valve disease, sociodemographic index, prevalence, incidence

## Abstract

**Background:**

Calcific aortic valve disease (CAVD) is a degenerative condition marked by aortic valve thickening and calcification, leading to stenosis or regurgitation, with rising prevalence in developed countries.

**Objectives:**

This study aimed to analyze the epidemiological trends and disease burden of CAVD in the global working-age population (defined in this study as the age group of 15 to 64 years) from 1992 to 2021. It further examined differences by Sociodemographic Index (SDI) regions and gender, providing insights for public health strategies.

**Methods:**

Data were obtained from the Global Burden of Disease (GBD) 2021 study, using age-standardized rates (ASR) to adjust for differences in age distribution across populations, allowing for more accurate comparisons of disease burden over time and between regions. Trends were analyzed using Joinpoint regression, and health disparities were assessed using Slope and Concentration Indexes. The impact of demographic factors on CAVD burden was explored through decomposition analysis.

**Results:**

From 1992 to 2021, CAVD prevalence in working-age populations rose from 1.30 to 2.65 million cases (103.43% increase). The results of multiple analysis models (including age-period-cohort model, decomposition analysis and frontier analysis) of the working-age population showed that the prevalence of CAVD peaked at the age of 64, and the prevalence of CAVD was significantly higher in males than in females. Population growth and ageing are the main drivers of the increasing burden of disease, with the highest burden in high SDI regions, especially Europe. These findings highlight the need to implement targeted interventions in high-risk populations.

**Conclusions:**

The burden of CAVD in working-age populations is rising. Despite age-standardized death rate (ASDR) and age-standardized disability-adjusted life years (ASDALY) declines, the overall increase in cases requires targeted prevention and management strategies, especially in high-burden settings. Effective interventions are critical for reducing the global CAVD burden.

## 1 Introduction

Calcified disease of the aortic valve (CAVD) is a common degenerative heart disease characterized by a gradual thickening, stiffening, and deposition of calcium salts in the aortic valve, which eventually leads to valve stenosis or regurgitation (1–3). Heart failure, angina pectoris, or syncope may progress due to impaired valve function (2). The pathological mechanisms of CAVD are similar to those of atherosclerosis, including lipid deposition, chronic inflammation, and calcification (4–6).

Studies have shown that CAVD is a common valve disease in Western and developed countries (7). A study from Sweden showed that the prevalence of the disease increased significantly with age, with a median age of onset of aortic valve calcification stenosis of 68 (57 to 77) years, of which 66% were males, and the global prevalence of the disease reached 9,404,077 in 2019 (8, 9). Since the 1980s, due to changes in penicillin and living conditions, rheumatic valvular disease due to rheumatic fever has decreased significantly (10), and has been replaced by an annual increase in non-rheumatic valvular disease (7).

Organizations such as the United Nations typically define the working age range as 15 to 64 years old, and individuals within this age group are able to work and participate in economic activities. This age group represents the core of the labor force, and their health status has significant implications for economic productivity and societal development. Given the rising prevalence of CAVD in developed countries, understanding its burden in the working-age population is crucial for identifying early risk factors and implementing preventive measures. Despite the significant economic and societal implications of cardiovascular diseases in this population, there is a notable gap in research focusing on the burden of CAVD in the working-age group. Most existing studies have primarily focused on older populations, leaving the epidemiology and risk factors of CAVD in younger adults poorly understood. Furthermore, regional disparities and gender differences in CAVD burden remain underexplored, particularly in the context of varying sociodemographic and healthcare conditions. However, to date, there has been limited research on CAVD in the working-age range. In-depth study of the burden of CAVD in the working-age population is of great significance for understanding the development trend and potential specific etiology of the disease in younger populations. This study aims to address these gaps by providing a comprehensive analysis of CAVD burden trends, regional disparities, and gender differences, as well as projecting future disease burden to inform targeted public health interventions.

## 2 Method

### 2.1 Data source

The data used in this study are from the GBD 2021 project, developed by the Institute for Health Metrics and Evaluation (IHME), including death rate, prevalence, and Incidence (11). In this study, some of the data were reported with 95% uncertainty intervals, which were mainly based on data characteristics and research objectives. From the perspective of data sources, the GBD 2021 data is integrated from multiple sources, covering information from different regions and populations around the world, and there are many uncontrollable variation factors in the process of data collection and integration, which makes it difficult for the traditional confidence interval based on sampling theory to accurately reflect the true uncertainty of the data. In the International Classification of Diseases (ICD-10), non-rheumatic calcific aortic valve disease (NCAVD) is classified as I35–I35.9 and is specifically used to distinguish CAVD not associated with rheumatic heart disease. The GBD 2021 database provides the number of diseases in the working population in each country around the world, as well as the age-standardized rate of the working-age population. The GBD2021 database also provides the main causes of the burden of disease.

### 2.2 Estimation framework and indicator definitions

The GBD project uses the advanced Disease Model—Bayesian Meta Regression 2.1 (DisMod-MR) to estimate the prevalence and incidence of NCAVD. For death rate, the GBD project uses the Integrated Modeling of Causes of Death (CODEm) approach to generate robust estimates of NCAVD mortality by integrating global registry data and multi-source data such as oral autopsies (11, 12). This definition aims to clarify the research object, ensure the consistency and comparability of data among different studies, and provide a standardized basis for accurately analyzing the epidemiological characteristics and disease burden of CAVD. Through this classification, researchers can more precisely conduct statistics and analysis on the disease, avoiding confusion with other valvular diseases. This, in turn, lays a solid foundation for subsequent research and the formulation of public health strategies.

### 2.3 Sociodemographic index

SDI is an indicator that quantifies the level of socio-economic development of a country or region based on three factors: fertility rate, education level and per capita income. SDI values range from 0 to 1, with higher values representing higher levels of social and economic development (11, 12). In this study, we divided countries and regions into five SDI categories (low, low-Middle, Middle, High-Middle, and high).

### 2.4 Definition of working-age population

The World Population Prospect, published by the United Nations in the Population Division of the Department of Economic and Social Affairs, defines the working age as 15 to 64 years, a range that is widely used to analyze the labour market, economic participation rate, demographic structure, etc. For details, please refer to https://population.un.org/wpp/GlossaryOfDemographicTerms/.

### 2.5 Statistical analyses

In this paper, the Joinpoint model is used to identify the point in time at which there is a significant change in disease burden in time series data, called an “inflection point” (13). This approach provides a clear picture of the change in disease burden over time, especially in the prevalence of CAVD burden (Supplementary Material).

Spearman was used to assess the association between CAVD and the ASR of SDI regions.

To measure the inequality of the burden of CAVD across countries between 1992 and 2021, we used two indicators: the Slope Index of Inequality (SII) and the Concentration Index of Inequality (CII), which were used to analyze absolute and relative inequalities, respectively. The SII is used to measure the absolute difference in the burden of CAVD between countries with different SDI (14).

In this study, we employed Data Envelopment Analysis (DEA), a frontier analysis method, to determine the minimum achievable measres for CAVD based on SDI, including death rate, prevalence, incidence, and DALYs. DEA is a non-parametric approach that evaluates the efficiency of different regions or countries by comparing their performance against an optimal “frontier” of best practices. This method allows us to identify regions that are performing optimally and those that have room for improvement in reducing CAVD burden. This approach aims to assess potential room for improvement in the burden of CAVD in countries or regions at different SDI levels. The frontier value represents the lowest burden of disease value. that a country or region can theoretically achieve at its SDI level (Supplementary Material).

In order to explore the driving factors of the burden of CAVD in different SDI regions around the world, a four-factor decomposition analysis proposed by Angela Y et al. (15) was adopted. These four factors are population size, population age structure, disease prevalence, and case fatality rate along with disease severity. By means of this method, it is possible to deeply explore the causes that trigger changes in various epidemiological indicators (Supplementary Material).

In the analytical framework of this study, an age-period-cohort model was used to deconstruct and analyze the temporal trend of the burden of CAVD.

The Bayesian age—period—cohort (BAPC) model, combined with the nested Laplace approximation (INLA) method, was used to deconstruct and analyze the temporal trend of the burden of CAVD (16, 17) (Supplementary Material).

The statistical analysis and visualization tools involved in this study include the World Health Organization's Health Equity Assessment Toolkit, StataMP 16, and *R* 4.3.1.

### 2.6 Ethical considerations

This study adheres to the Guidelines for Accurate and Transparent Health Estimates Reporting (GATHER) (18), ensuring the accuracy and transparency of the reporting process. Ethical approval and informed consent were not required, as the study utilized publicly accessible, aggregated, and anonymized secondary data from the GBD database. The nature of this data ensures the privacy of the study subjects and compliance with data usage regulations.

## 3 Result

### 3.1 Global burden of CAVD in working-age populations

**Figure 1:**
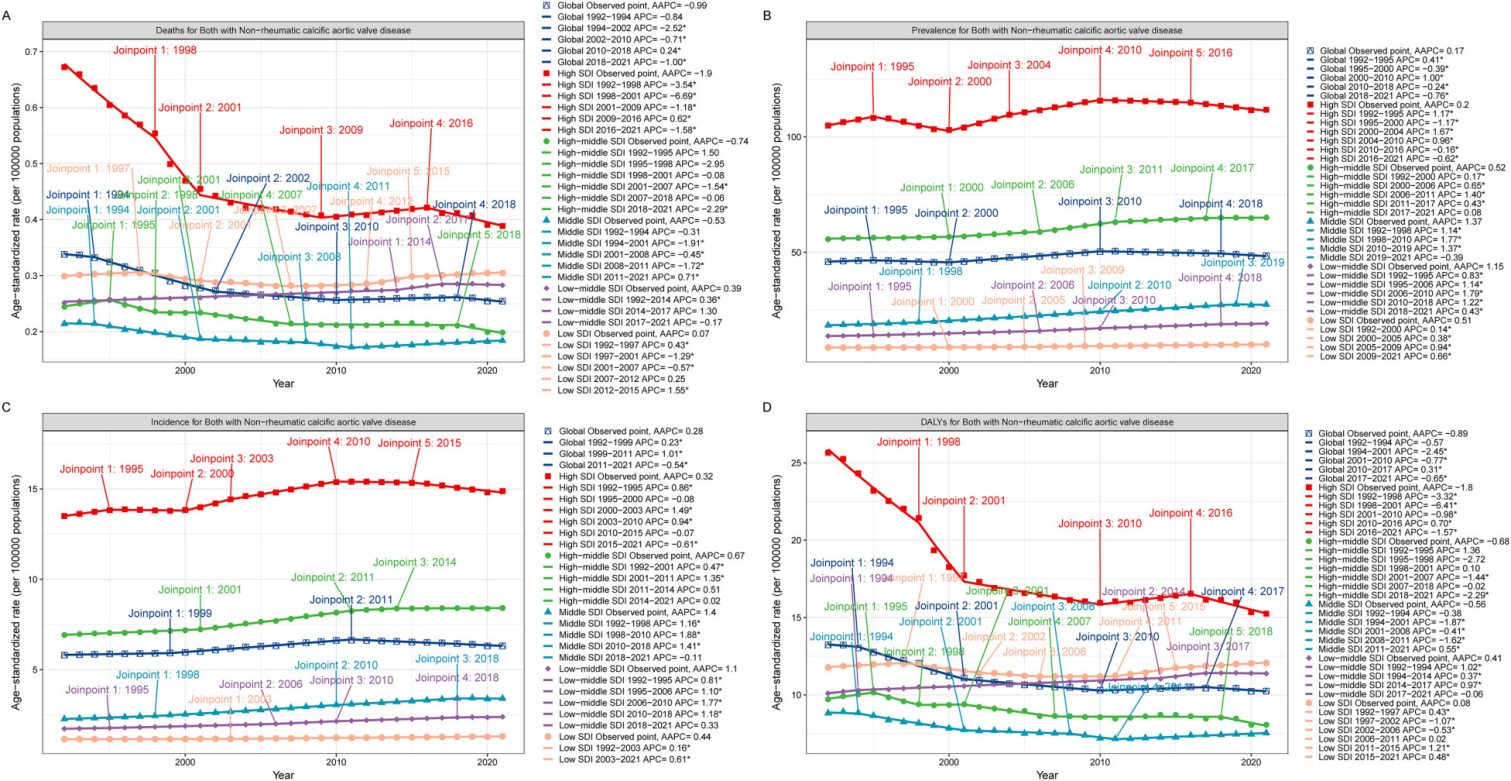
Joinpoint model of the disease burden of CAVD in working-age populations in different SDI regions around the world. Joinpoint model of CAVD in working-age populations in different SDI regions. **(A)** ASDR; **(B)** ASPR; **(C)** ASIR; **(D)** ASDALYs. AAPC, average annual percent change; APC, annual; percent change; SDI, sociodemographic index; DALYs, disability-adjusted life years.

From 1992 to 2021, the prevalence of CAVD in the working-age population increased from 1,306,712.79 cases (95% UI: 961,803.13 to 1,733,775.27) to 2,658,285.84 cases (95% UI: 1,995,025.60 to 3,458,767.58), an increase of 103.43%. In 1992, Age-standardized prevalence rate (ASPR) and Age-Standardized incidence rate (ASIR) for CAVD were 45.96per 100,000 persons (95% UI: 33.88 to 60.87) and 5.81 per 100,000 persons (95% UI: 4.00 to 8.13), respectively; In 2021, these two indicators rose to 48.50 per 100,000 persons (95% UI: 36.38 to 63.14) and 6.32 per 100,000 persons (95% UI: 4.51 to 8.62), respectively. The Joinpoint analysis showed that there were four inflection points in prevalence between 1992 and 2021 (in 1995, 2000, 2010 and 2018) and two inflection points in incidence (in 1999 and 2011) (Table 1, Figures 1B,C).

**Table 1:** Working-age CAVD burden from 1992 to 2021.

Classification	Measure	1992 Cases (95% UI)	1992 Age-standard prevalence per 100,000 (95% UI)	2021 Cases (95% UI)	2021 Age-standard prevalence per 100,000 (95% UI)	AAPC% (95% CI) 1992–2021	EAPC% (95% CI) 1992–2021
Global	prevalence	1306712.79 (961803.13 to 1733775.27)	45.96 (33.88 to 60.87)	2658285.84 (1995025.60 to 3458767.58)	48.50 (36.38 to 63.14)	0.17 (0.10 to 0.25)	0.33 (0.25 to 0.42)
Sex							
Male		863883.79 (640813.66 to 1136934.21)	60.76 (45.15 to 79.82)	1755369.81 (1325248.54 to 2261083.48)	65.02 (49.09 to 83.77)	0.24 (0.17 to 0.30)	0.37 (0.29 to 0.46)
Female		442829.00 (319594.09 to 600094.04)	31.14 (22.51 to 42.13)	902916.02 (661196.66 to 1202654.98)	32.45 (23.74 to 43.27)	0.13 (0.04 to 0.22)	0.30 (0.21 to 0.39)
SDI							
Low SDI		16376.99 (10999.91 to 23533.03)	8.78 (5.94 to 12.56)	41570.39 (28504.80 to 59013.04)	10.19 (7.04 to 14.38)	0.51 (0.45 to 0.56)	0.55 (0.50 to 0.59)
Low-middle SDI		68013.58 (47072.34 to 96077.14)	13.75 (9.57 to 19.33)	198866.20 (141530.01 to 273354.02)	19.18 (13.70 to 26.27)	1.18 (1.13 to 1.23)	1.25 (1.22 to 1.29)
Middle SDI		152399.30 (108830.85 to 209699.28)	18.44 (13.23 to 25.26)	502847.29 (364127.03 to 681316.57)	27.39 (19.83 to 37.11)	1.37 (1.32 to 1.41)	1.53 (1.48 to 1.58)
High-middle SDI		392184.28 (288092.18 to 521601.42)	55.72 (40.88 to 74.10)	782709.27 (860787.70 to 1450918.97)	65.13 (48.77 to 84.80)	0.52 (0.47 to 0.57)	0.66 (0.60 to 0.71)
High SDI		675243.31 (503486.78 to 895928.58)	104.92 (78.16 to 139.15)	1127877.32 (587770.67 to 1016138.01)	111.75 (84.90 to 144.32)	0.20 (0.14 to 0.26)	0.35 (0.24 to 0.45)
Classification	incidence	1992 Cases (95% UI)	1992 Age-standard incidence per 100,000 (95% UI)	2021 Cases (95% UI)	2021 Age-standard incidence per 100,000 (95% UI)	AAPC% (95% CI) 1992–2021	EAPC% (95% CI) 1992–2021
Global		165771.74 (114024.30 to 231799.27)	5.81 (4.00 to 8.13)	346057.36 (246627.10 to 472270.57)	6.32 (4.51 to 8.62)	0.28 (0.24 to 0.33)	0.45 (0.34 to 0.55)
Sex							
Male		106941.46 (74548.48 to 148486.42)	7.48 (5.22 to 10.39)	223484.89 (160778.87 to 302951.77)	8.28 (5.96 to 11.21)	0.34 (0.29 to 0.39)	0.52 (0.41 to 0.62)
Female		58830.28 (39260.07 to 84272.78)	4.13 (2.75 to 5.92)	122572.48 (84889.97 to 171775.30)	4.41 (3.05 to 6.18)	0.21 (0.14 to 0.28)	0.37 (0.27 to 0.47)
SDI							
Low SDI		2254.88 (1425.17 to 3369.33)	1.16 (0.74 to 1.73)	5664.62 (3665.77 to 8339.73)	1.32 (0.86 to 1.93)	0.44 (0.42 to 0.46)	0.46 (0.42 to 0.50)
Low-middle SDI		8821.65 (5608.26 to 13123.13)	1.74 (1.11 to 2.58)	25218.67 (16757.86 to 36760.58)	2.40 (1.60 to 3.48)	1.10 (1.01 to 1.19)	1.21 (1.18 to 1.25)
Middle SDI		19228.53 (12394.26 to 28165.83)	2.28 (1.47 to 3.33)	62747.17 (41859.22 to 90294.65)	3.41 (2.28 to 4.91)	1.40 (1.33 to 1.46)	1.58 (1.52 to 1.65)
High-middle SDI		48437.99 (33162.98 to 67972.85)	6.93 (4.72 to 9.75)	100609.93 (71997.93 to 136543.37)	8.42 (6.01 to 11.45)	0.67 (0.60 to 0.74)	0.83 (0.75 to 0.90)
High SDI		86724.96 (60561.71 to 120774.09)	13.50 (9.39 to 18.85)	151249.79 (109896.63 to 201230.06)	14.88 (10.71 to 19.94)	0.32 (0.22 to 0.42)	0.45 (0.36 to 0.55)
Classification	DALYs	1992 Cases (95% UI)	1992 Age-standard DALYs per 100,000 (95% I)	2021 Cases (95%UI)	2021 Age-standard DALYs per 100,000 (95% UI)	AAPC% (95% CI) 1992–2021	EAPC% (95% CI) 1992–2021
Global		397450.81 (355762.55 to 450707.67)	13.24 (11.89 to 14.94)	547176.89 (478166.10 to 613309.74)	10.24 (8.93 to 11.50)	−0.89 (−1.04 to −0.74)	−0.84 (−1.01 to −0.67)
Sex							
Male		268600.86 (240023.67 to 304407.12)	17.81 (15.97 to 20.11)	358825.01 (308789.77 to 401414.57)	13.50 (11.60 to 15.11)	−0.96 (−1.15 to −0.76)	−0.90 (−1.07 to −0.72)
Female		128849.95 (104531.15 to 168978.41)	8.60 (7.04 to 11.16)	188351.88 (143415.44 to 246270.03)	7.02 (5.32 to 9.23)	−0.71 (−0.86 to −0.57)	−0.69 (−0.86 to −0.52)
SDI							
Low SDI		25215.51 (15423.66 to 38853.00)	11.77 (7.25 to 18.03)	58149.36 (36847.99 to 79037.33)	12.07 (7.70 to 16.30)	0.08 (0.01 to 0.15)	−0.02 (−0.13 to 0.10)
Low-middle SDI		56776.34 (40243.87 to 80343.22)	10.10 (7.20 to 14.22)	126689.68 (97746.15 to 156564.47)	11.38 (8.79 to 14.04)	0.41 (0.31 to 0.52)	0.41 (0.39 to 0.43)
Middle SDI		83580.66 (73260.63 to 96183.70)	8.82 (7.76 to 10.11)	132878.31 (114226.79 to 153916.61)	7.53 (6.46 to 8.74)	−0.56 (−0.77 to −0.35)	−0.63 (−0.78 to −0.49)
High-middle SDI		68572.17 (60903.48 to 77988.60)	9.72 (8.64 to 11.05)	87609.53 (79842.54 to 97560.40)	8.06 (7.32 to 9.02)	−0.68 (−1.20 to −0.15)	−0.67 (−0.77 to −0.58)
High SDI		162815.31 (156611.18 to 169730.43)	25.66 (24.68 to 26.75)	141053.98 (134052.62 to 149347.25)	15.25 (14.49 to 16.15)	−1.80 (−2.10 to −1.51)	−1.57 (−1.91 to −1.23)
Classification	Deaths	1992 Cases (95% UI)	1992 Age-standard Deaths rate per 100,000 (95% UI)	2021 Cases (95% UI)	2021 Age-standard Deaths rate per 100,000 (95% UI）	AAPC% (95% CI) 1992–2021	EAPC% (95% CI) 1992–2021
Global		9890.81 (8941.46 to 11088.98)	0.34 (0.31 to 0.38)	13737.42 (12095.19 to 15265.90)	0.25 (0.22 to 0.28)	−0.99 (−1.17 to −0.81)	−0.93 (−1.12 to −0.73)
Sex							
Male		6655.47 (5988.93 to 7492.61)	0.45 (0.41 to 0.51)	8987.19 (7826.14 to 9947.28)	0.34 (0.29 to 0.37)	−1.05 (−1.24 to −0.85)	−0.97 (−1.16 to −0.77)
Female		3235.33 (2674.45 to 4131.32)	0.22 (0.18 to 0.28)	4750.24 (3670.37 to 6115.95)	0.17 (0.13 to 0.23)	−0.83 (−0.99 to −0.67)	−0.80 (−0.99 to −0.61)
SDI							
Low SDI		595.38 (364.79 to 912.10)	0.30 (0.18 to 0.46)	1344.39 (853.02 to 1818.44)	0.30 (0.19 to 0.41)	0.07 (−0.05 to 0.19)	−0.03 (−0.15 to 0.10)
Low-middle SDI		1332.86 (947.19 to 1881.02)	0.25 (0.18 to 0.35)	3049.37 (2347.50 to 3758.90)	0.28 (0.22 to 0.35)	0.39 (0.24 to 0.53)	0.42 (0.39 to 0.45)
Middle SDI		1920.56 (1687.87 to 2203.75)	0.21 (0.19 to 0.24)	3294.56 (2854.46 to 3803.38)	0.18 (0.16 to 0.21)	−0.53 (−0.74 to −0.32)	−0.63 (−0.79 to −0.46)
High-middle SDI		1722.33 (1551.01 to 1931.02)	0.24 (0.22 to 0.27)	2253.26 (2077.34 to 2477.81)	0.20 (0.18 to 0.22)	−0.74 (−1.25 to −0.23)	−0.76 (−0.86 to −0.66)
High SDI		4307.67 (4160.25 to 4469.17）	0.67 (0.65 to 0.70)	3775.75 (3610.36 to 3936.86)	0.39 (0.37 to 0.41)	−1.90 (−2.20 to −1.59)	−1.65 (−2.01 to −1.29)

95% UI, 95% uncertainty intervals; AAPC, average annual percent change; EAPC, estimated annual percentage change; 95% CI, 95% confidence interval; SDI, sociodemographic index; DALYs, disability-adjusted life years.

In 1992, the number of DALYs due to CAVD in the global working-age population was 397,450.81 cases (95% UI: 355,762.55 to 450,707.67), which increased to 547,176.89 cases (95% UI: 478,166.10 to 613,309.74) in 2021 (Table 1, Figure 1D). The number of deaths related to CAVD in the global working-age population increased from 9,890.81 cases (95% UI: 8,941.46 to 11,088.98) in 1992 to 13,737.42 cases (95% UI: 12,095.19 to 15,265.90) in 2021 (Table 1, Figure 1A).

### 3.2 Burden of CAVD among working-age populations in each SDI region

From the perspective of ASPR, there is a positive correlation between the SDI index and ASPR. In 1992, the number of cases among the working-age population in the High SDI region was 675,243.31 cases (95% UI: 503,486.78 to 895,928.58) and in 2021, this number increased to 1,127,877.32 cases (95% UI: 587,770.67 to 1,016,138.01). The region with the lowest ASPR is the Low SDI region. The Joinpoint analysis and the GBD 2021 showed that the prevalence was significantly higher in the high SDI region than in other regions (Table 1, Figure 1B). And Joinpoint analysis and the GBD 2021 showed that the incidence was significantly higher in the High SDI region than in other regions, consistent with the prevalence trend (Table 1, Figure 1C).

DALYs and mortality were also consistent with the above trends across SDI regions. Although the total number of DALYs and deaths was higher in the High SDI region, ASDALYs and ASDR showed a downward trend in the region, with lower AAPC and EAPC at −1.80 (95% CI: −2.10 to −1.51), −1.90 (95% CI: −2.20 to −1.59), and −1.57 (95% CI: −1.91 to −1.23), −1.65 (95% CI: −2.01 to −1.29). Joinpoint analysis showed that the most significant downward trend in ASDALYs and ASDR was seen between 1998 and 2001 (Table 1, Figures 1A,D).

In addition, from the correlation study, it can be seen that there is a certain linear relationship between SDI area and CAVD within the working-age population, especially for ASPR and ASIR, there is a positive correlation, and the correlation can reach 0.83 (Supplementary Figures S1B,C). The correlation trend between ASDALYs and ASDR is not as obvious as that of the above two indicators (Supplementary Figures S1A,D).

### 3.3 The disease burden of CAVD in the working-age population of countries worldwide

**Figure 2:**
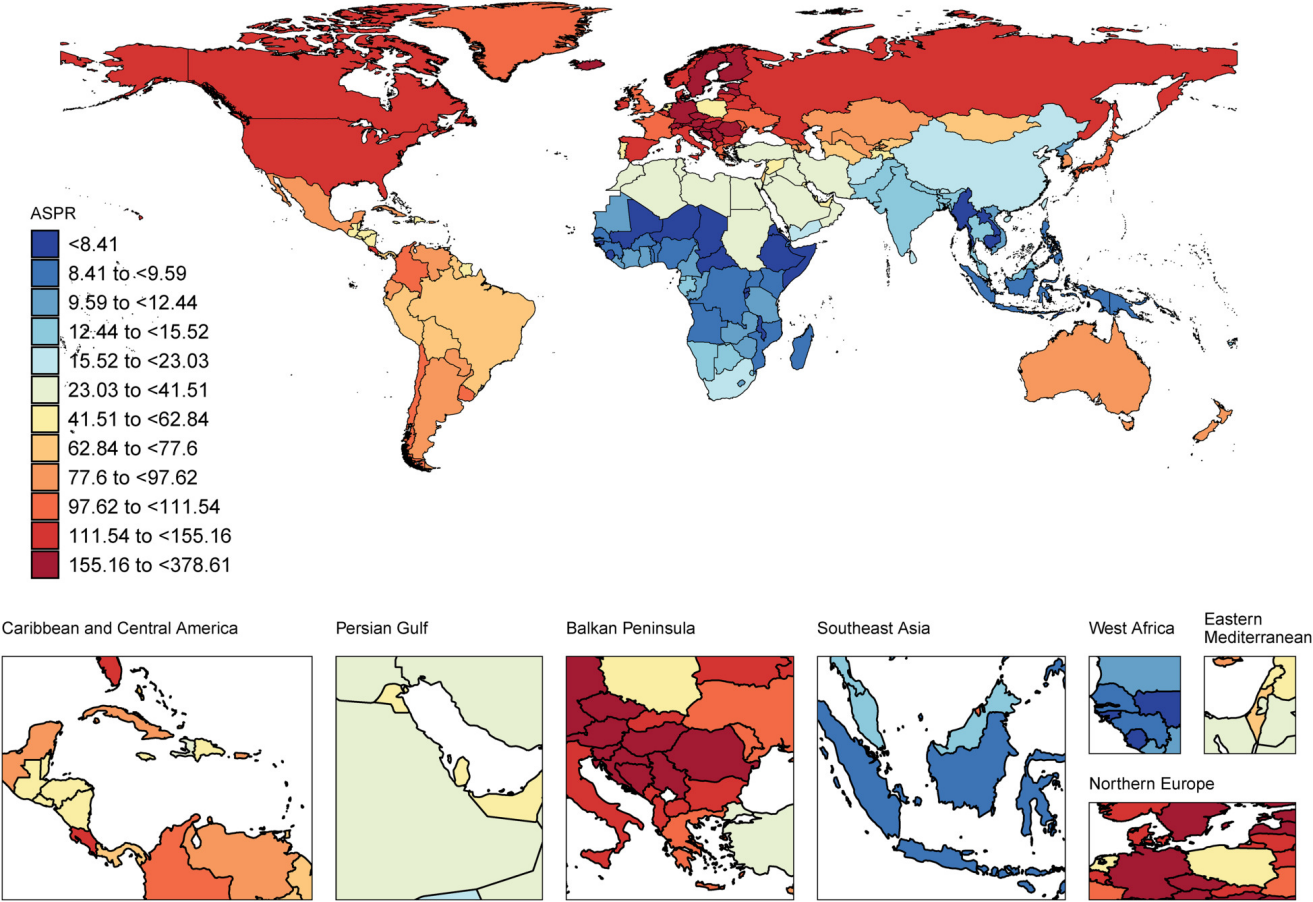
World map of the CAVD burden of the working-age population. ASPR, age-standard prevalence rate. World map from “Global country administrative boundary data” by Resource and Environmental Science Data Platform.

In 2021, among the working-age population in 204 countries and regions, Romania ranked first in the world in terms of ASPR of CAVD (378.61 per 100,000 persons) (95% UI: 265.40 to 505.17), followed by Slovenia and Hungary with 287.20 per 100,000 persons respectively (95% UI: 205.67 to 396.55) and 267.13 per 100,000 persons (95% UI: 196.41 to 348.20). These countries are all located in Europe, which is a more developed region and may have higher morbidity due to higher detection rates and lifestyle habits (Figure 2, Supplementary Table S1). In terms of ASIR, Romania also led the way with 47.82 per 100,000 persons (95% UI: 31.23 to 66.01), followed by Slovenia and Austria with 46.79 per 100,000 persons (95% UI: 34.16 to 64.12) and 39.05 per 100,000 persons (95%) respectively (95% UI: 25.65 to 56.55). The epidemiological differences in CAVD in working-age populations were highlighted among different countries worldwide (Supplementary Figure S2, Supplementary Table S1).

In terms of ASDALYs, Kiribati performed the highest at 66.53 per 100,000 persons (95% UI: 35.79 to 112.47). In contrast, the country with the lowest ASDALYs was Tajikistan with only 0.41per 100,000 persons (95% UI: 0.20 to 0.78) (Supplementary Figure S3, Supplementary Table S1). Tajikistan not only had the lowest ASDALYs but also the lowest ASDR. These data suggest that even economically developed countries may face a high burden of CAVD (Supplementary Figure S4, Supplementary Table S1).

### 3.4 Global burden of CAVD in working-age populations of different genders

Between 1992 and 2021, ASPR and ASIR showed an overall upward trend in men and women with CAVD in the working-age population. Both in terms of prevalence and incidence, men consistently had higher ASR than women, a trend that was validated in Joinpoint model analysis (Table 1, Supplementary Figures S5B,C). Although ASPR and ASIR showed an increase in both men and women, they differed in ASDALYs and ASDR (Table 1, Supplementary Figures S5A,D).

**Figure 3:**
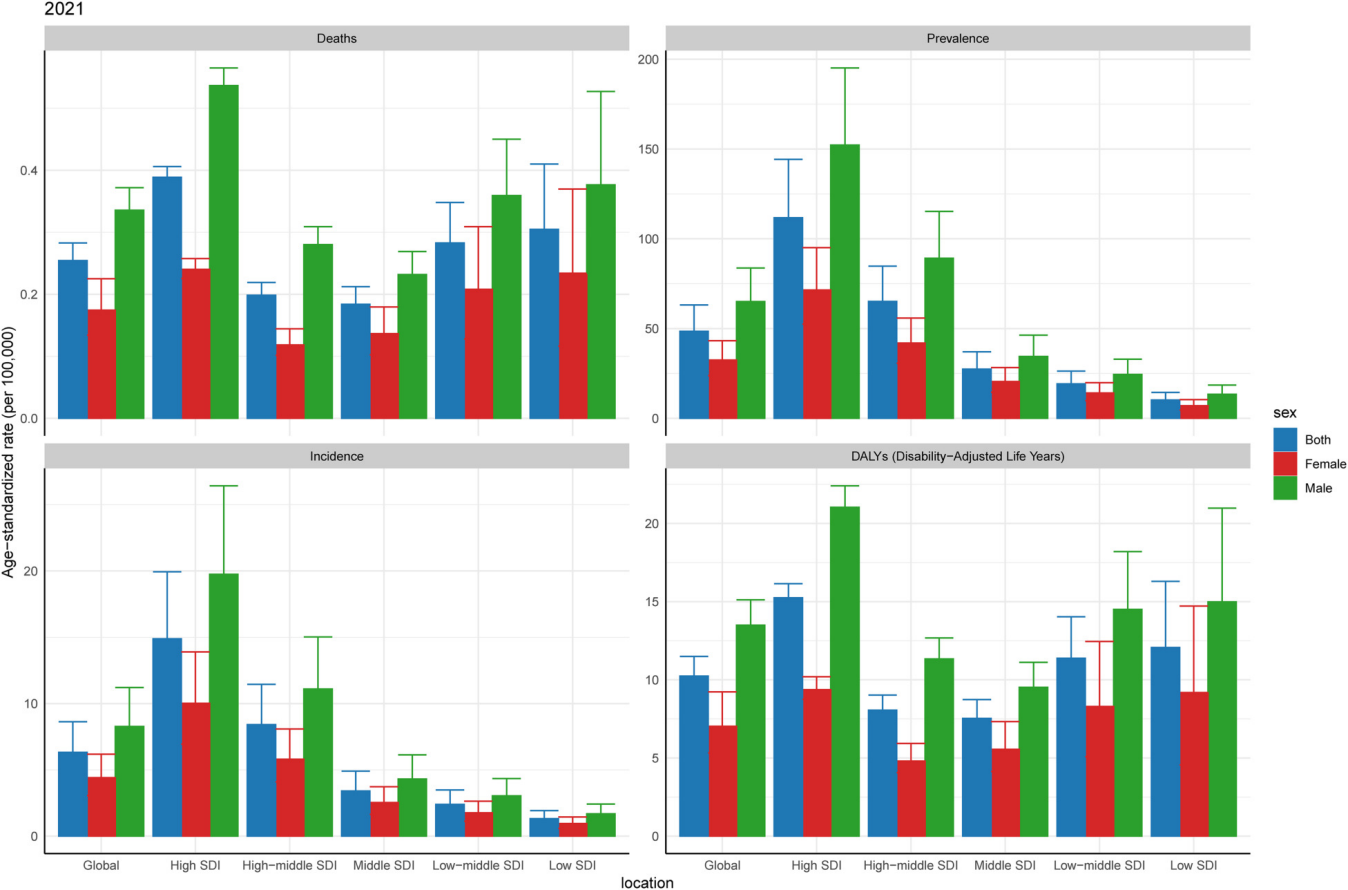
Burden of CAVD among working-age of different genders in 2021. SDI, sociodemographic index.

At present, according to the statistics of CAVD within the working-age population in 2021, the ASPR and ASIR of men are higher than those of women in all SDI regions. In terms of ASDALYs and ASDR, the High SDI region has relatively high values. For ASDALYs and ASDR, the Middle SDI region has the lowest values, which is consistent with the second part of the study (Table 1, Figure 3).

### 3.5 Analysis of health inequalities in CAVD in the global working-age population

**Figure 4:**
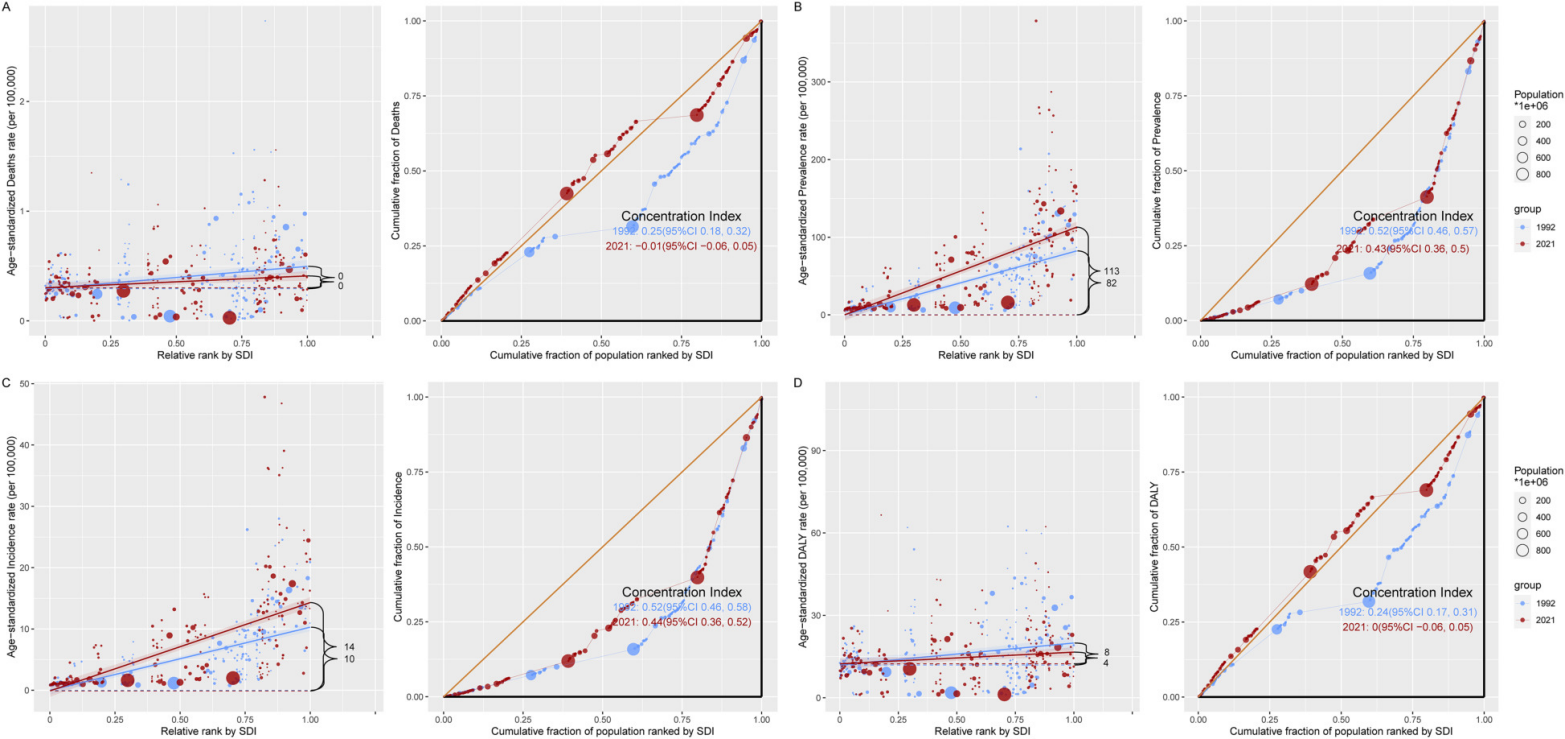
A model for analyzing the health inequality of CAVD in the working-age population. There are two images for each indicator, with the regression line on the left side of each indicator representing the analysis of absolute health inequalities related to the burden of CAVD in the working-age population in the SDI region in 1992 in 2021, and the graph on the right showing the trend line of the concentration index in 1992 vs. 2021. **(A)** ASDR; **(B)** ASPR; **(C)** ASIR; **(D)** ASDALY. DALYs, disability-adjusted life years; SDI, sociodemographic index; CI, confidence interval.

Taking ASPR as an example, the slope indices for ASPR's absolute health inequality analysis were 82 and 113 in 1992 and 2021, respectively, indicating an increase in the degree of absolute health inequities. The concentration index was 0.52 (95% concentration index: 0.46 to 0.57) in 1992 and 0.43 (95% concentration index: 0.36 to 0.50) in 2021. Both values were positive, indicating that the burden of working-age CAVD was mainly concentrated in the High SDI region, and the relative health inequality was reduced (Figure 4B, Supplementary Tables S2, S3). Similar to ASPR, the slope index of ASIR health inequality analysis was 10 and 14 in 1992 and 2021, respectively, and the concentration index for relative inequality analysis was 0.52 (95% concentration index: 0.46 to 0.58) in 1992 and 0.44 (95% concentration index: 0.36 to 0.52) in 2021 (Figure 4C, Supplementary Tables S2, S3). In addition, the slope indices of ASDALYs in 1992 and 2021 were 8 and 4, respectively, indicating a decline in absolute health inequality in ASDALYs. For relative inequality analysis, the concentration index in 1992 was 0.24 (95% concentration index: 0.17 to 0.31) and the concentration index in 2021 was 0 (95% concentration index: −0.06 to 0.05), indicating a decrease in relative health inequality and a balanced distribution of ASDALYs across SDI regions (Figure 4D, Supplementary Tables S2, S3). It is worth noting that for the ASDR indicator, the slope index in both 1992 and 2021 is 0 in the absolute inequality analysis. The relative inequality analysis is similar to ASDARY (Figure 4A, Supplementary Figure S1A, Supplementary Tables S2, S3).

### 3.6 Frontier analysis of CAVD in the working-age population of different countries around the world from 1992 to 2021

**Figure 5:**
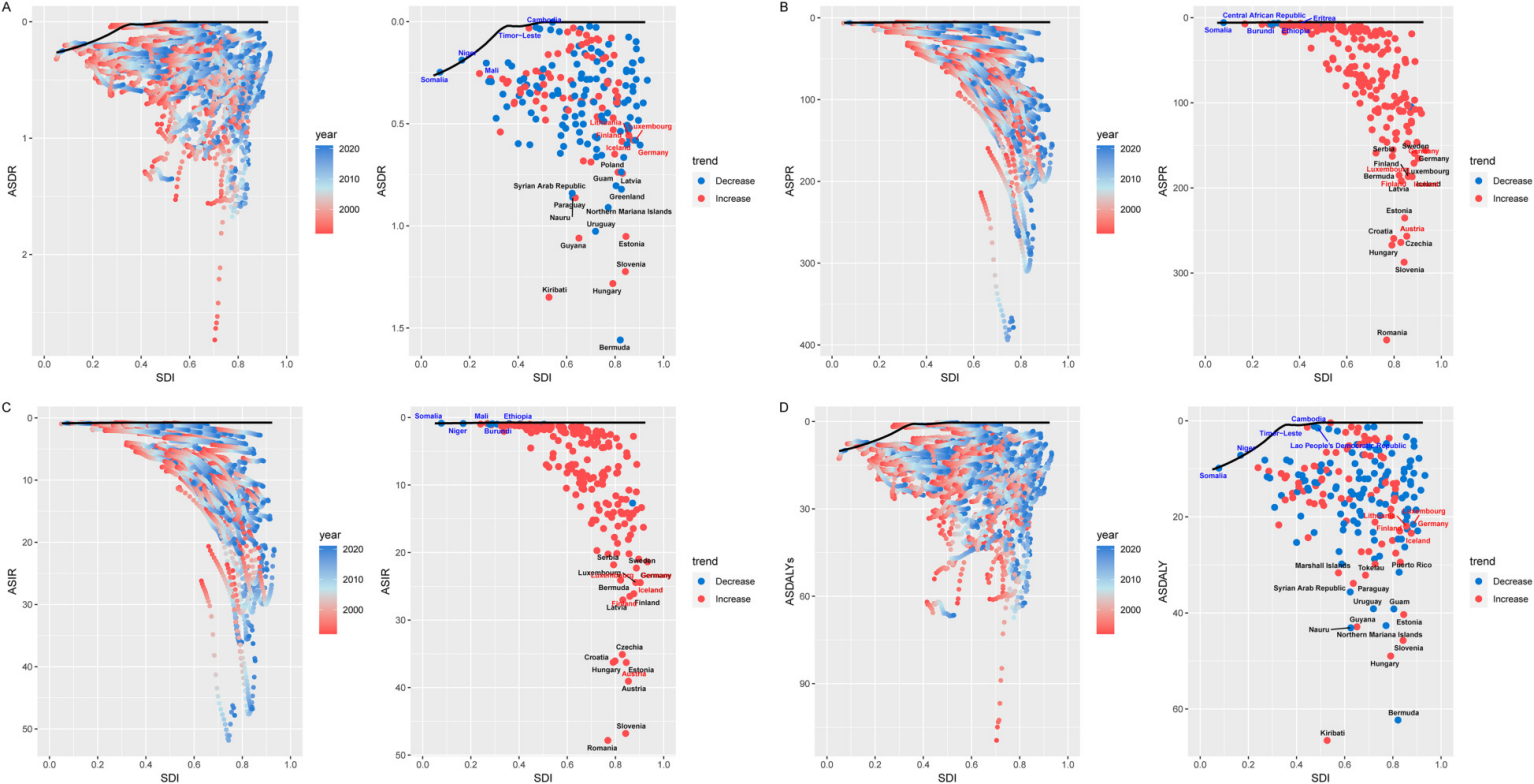
Frontier analysis of CAVD in the working-age population. The boundaries of the frontier analysis are marked in solid black, and countries and regions are shown as points. The red and blue gradients are used to show the SDI and rate trends for each country or region. The red dot indicates an increase in the burden of disease between 1992 and 2021, and the blue dot indicates an increase in the burden of disease during this period. The top 15 countries (the countries with the highest leading values for CAVD gap in the working-age population) are marked in black. Countries that are far from the frontier analysis are marked with red names. Countries with fewer gaps are marked with blue names compared to cutting-edge analysis. **(A)** ASDR; **(B)** ASPR; **(C)** ASIR; **(D)** ASDALY. SDI, sociodemographic index; ASDR, age-standard death rate; ASPR, age-standard prevalence rate; ASIR, age-standard incidence rate; ASDALYs, age-standard disability-adjusted life years rate.

According to our observations, for both ASPR and ASIR, most countries showed a clear upward trend between 1992 and 2021. Frontier analyses show that this trend is roughly straight, suggesting that those countries that perform prominently in the burden of CAVD have relatively small differences in the burden of CAVD across different SDI intervals and working-age population. Nevertheless, our study found that the countries with the largest gap between ASPR and best performance countries were mainly concentrated in countries with high SDI, among which the 10 countries with large effective differences in ASPR included Romania, Slovenia, Hungary, Czechia, Croatia, Austria, Estonia, Latvia, Iceland, and Finland (EF range was 181.26 to 373.39). The trend of ASIR is similar, and the horizontal linear relationship is similar to that of ASPR. In terms of ASIR, the 10 countries with the largest effective differences from the frontier countries are Romania, Slovenia, Austria, Estonia, Hungary, Croatia, Czechia, Latvia, Finland, (EF range is 25.35 to 47.07). Within the same SDI index, these countries have a large gap with the frontier countries, indicating that further steps are needed to close the gap (Figures 5B,C, Supplementary Table S4).

On both ASDR and ASDALYs, the trend curves are approximately the same. Both showed similar characteristics at a specific SDI point (around 0.3), and most countries or regions showed a downward trend between 1992 and 2021. Overall, the countries with the largest effective differences between ASDR and ASDALYs are still concentrated in countries with higher SDI indices. There is a gap between countries with an SDI below 0.3 and countries with an SDI above 0.3 between ASDR and ASDALYs front-line countries for the burden of the disease (Figures 5A,D, Supplementary Table S4).

### 3.7 Decomposition analysis of CAVD according to the working-age population

**Figure 6:**
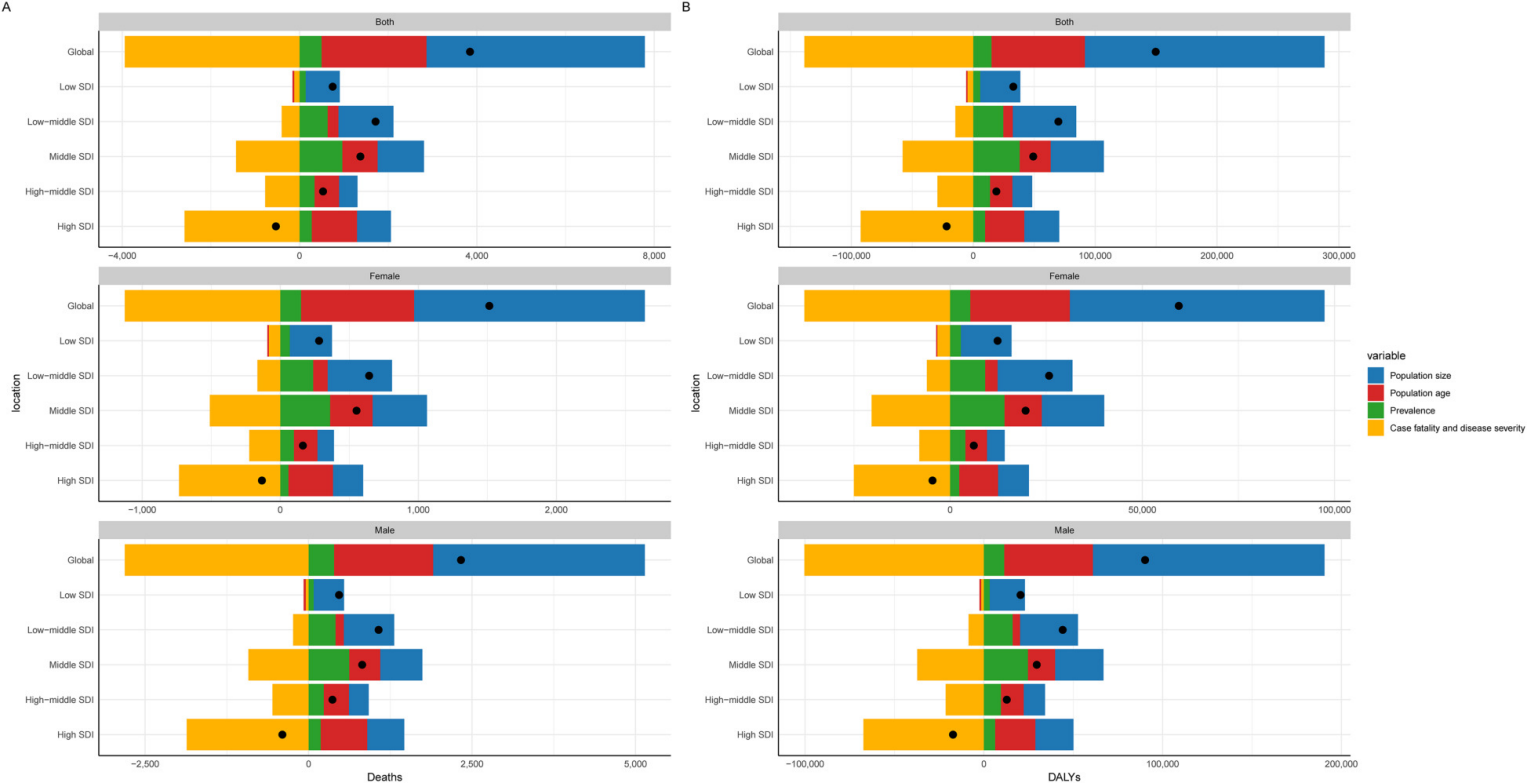
Decomposition analysis of CAVD in the working-age. **(A)** Death rate; **(B)** DALYs. SDI, sociodemographic index; DALYs, disability-adjusted life years rate.

ASDR and ASDALYs for CAVD in the global working-age population have shown a year-by-year decline, with ASDR experiencing the most notable reduction. The EAPC for ASDR was −0.93 (95% CI: −1.12 to −0.73), driven primarily by improvements in case fatality and disease severity, which accounted for a 102.47% decrease. However, the growing population size significantly increased the disease burden in the working-age population by 128.03%, suggesting that, despite population growth, the overall ASDR burden has declined. Other factors, such as population aging (61.50%) and prevalence (12.93%), had a comparatively minor impact on ASDR (Table 1, Figure 6A, Supplementary Table S5). Similarly, the EAPC for ASDALYs was −0.84 (95% CI: −1.01 to −0.67). Decomposition analysis indicated that reductions in case fatality and disease severity were the main drivers behind the 92.43% decrease in ASDALYs. Conversely, population size had the largest influence on increasing the disease burden (131.27%), followed by population aging (51.14%) and prevalence (10.02%). These findings reveal that while advancements in medical technology and disease management have contributed to a declining CAVD burden in the working-age population, the expanding population size has had a significant counteracting effect.

The marked reductions in ASDR and ASDALYs highlight the health benefits from improved medical interventions, early detection, and better disease management. However, as the population continues to grow, the negative impact becomes more pronounced, especially in high-SDI countries and specific gender groups, necessitating further public health measures. Additionally, the factors contributing to ASDR and ASDALYs differed by gender and SDI region (Table 1, Figure 6B, Supplementary Table S5), indicating a need for targeted strategies in these areas.

### 3.8 Changes in the indicators of the CAVD age-period-cohort model within the working-age population

**Figure 7:**
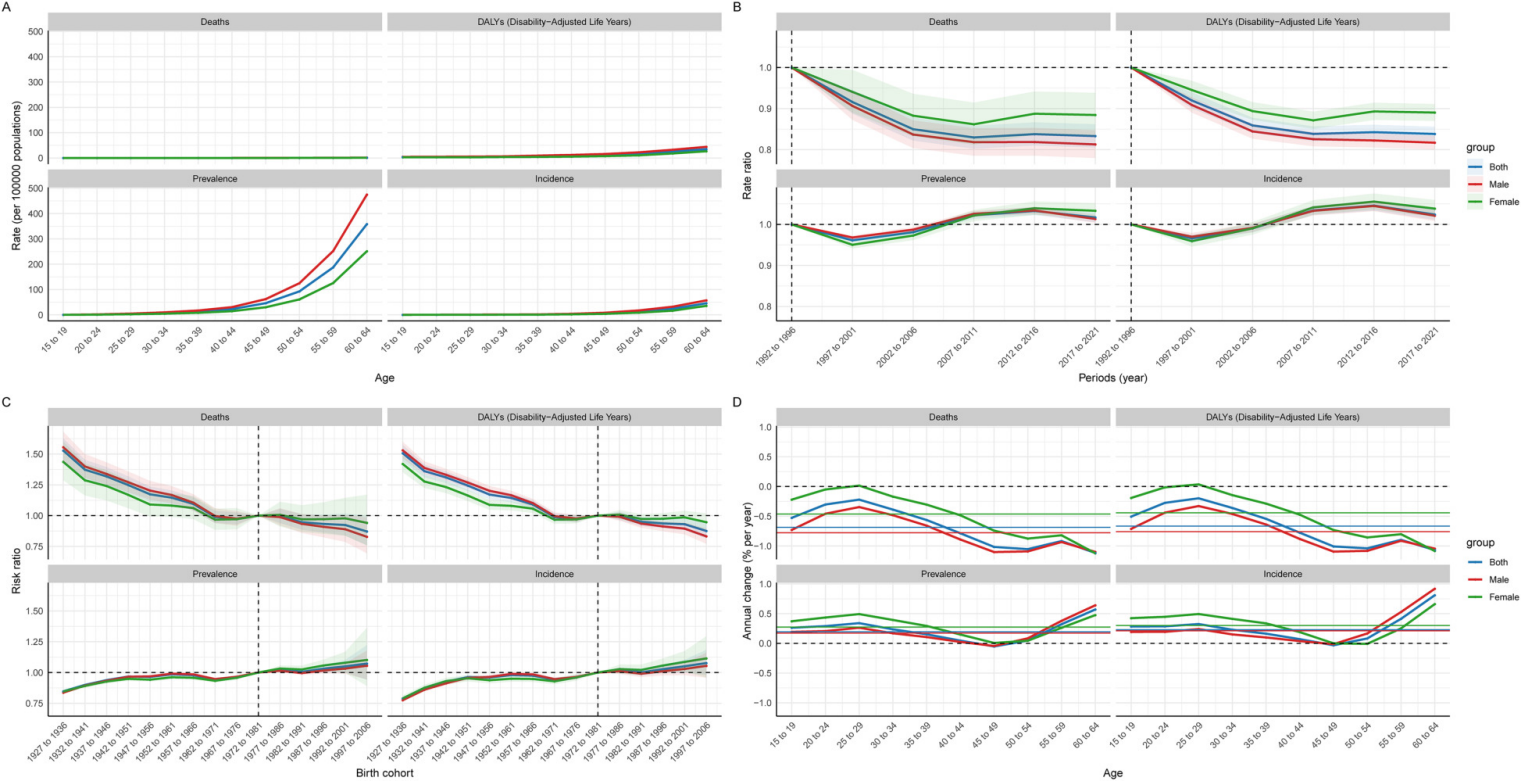
Global age-period-cohort model of CAVD in the global working age population. **(A)** Age cohort; **(B)** Period cohort; **(C)** Birth cohort; **(D)** The relationship between the age cohort cycle model and the annual rate of change.

In the age cohort model, it can be seen that the prevalence of CAVD in the working-age population gradually increases with increasing age, and the prevalence in men is significantly higher than that in women, reaching a peak at the age of 64. However, the other indicators (death rate, incidence, and DALYs) did not show a significant trend in the age cohort model (Figure 7A, Supplementary Table S6). In addition, from the analysis of the age cohort and the annual rate of change model, the mean annual rate of change of prevalence and incidence rate were positive, indicating that these two indicators still showed a year-on-year increasing trend in the working-age population, which is consistent with the results in Table 1 of this paper. Annual increases in incidence and prevalence increase with age, with males having the most significant annual change in these two indicators. In contrast, the annual rate of change of death rate and DALYs was negative, indicating an overall year-on-year downward trend (Table 1, Figure 7D, Supplementary Tables S7, S8).

In the period cohort model, the trend of death rate and DALYs was similar, showing a downward trend over time, indicating that the treatment of CAVD is gradually improving. The incidence and prevalence showed a phased change: from 1992 to 2001, there was a downward trend. Between 2002 and 2016, there was a marked increase in incidence and prevalence. Since 2016, there has been a slight decline in these indicators (Figure 7B, Supplementary Table S9).

In the birth cohort model, it can be observed that the population born between 1927 and 1933 has lower prevalence and death rate, but this population has a higher death rate. People born between 1997 and 2006 showed higher prevalence and incidence, but lower death rate and DALYs (Figure 7C, Supplementary Table S10).

### 3.9 Projected global burden of CAVD in the working-age population, 2035

**Figure 8:**
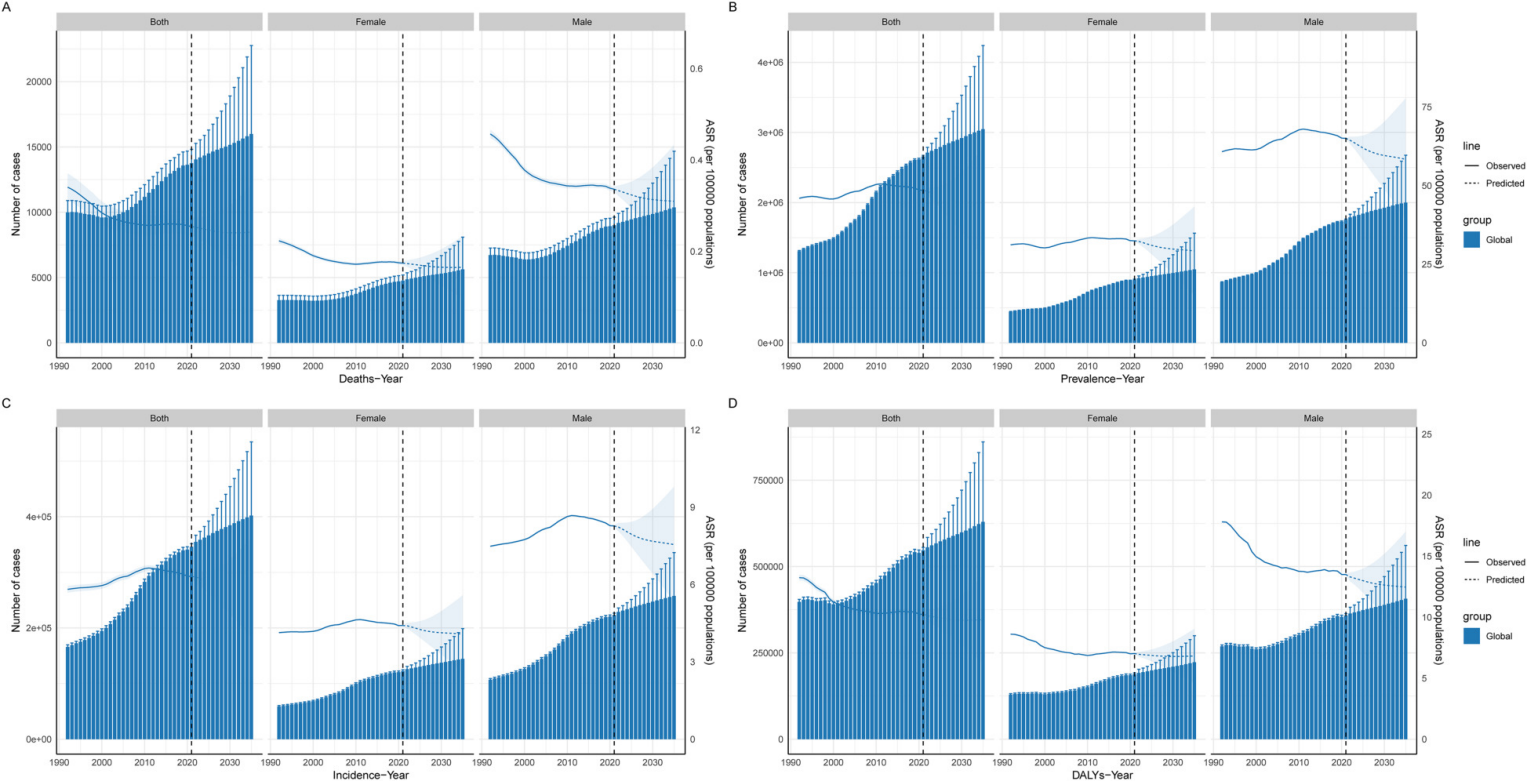
Projected global burden of CAVD in the working-age population. Prediction of CAVD in the global working-age population. The solid blue line represents the observed age-standardized rate from 1992 to 2021, the dotted line represents the predicted burden of disease from 2021 to 2035, and the histogram represents the number of people at burden. **(A)** Deaths; **(B)** Prevalence; **(C)** Incidence; **(D)** DALYs. DALYs, disability-adjusted life years; ASR, age-standard rate.

The BAPC model was used to predict future trends and the number of cases of CAVD in the global working-age population between 2022 and 2035. The results show that the number of cases due to the prevalence, incidence, DALYs, and mortality of the disease is expected to continue to rise from 2021 to 2035. For example, by 2035, the number of prevalence cases is expected to reach 3,048,178.27 cases (95% UI: 1856574.01 to 4239782.53), the number of incidence cases will be 402,046.29 cases (95% UI: 269661.58 to 534431.00), and DALYs will reach 629,405.83 cases (95% UI: 397900.02 to 860911.64), and the death toll was 15989.06 cases (95% UI: 9217.84 to 22760.28). These data suggest that CAVD will continue to be a significant health burden in the working-age population. Comparatively, ASPR, ASIR, ASDALYs, and ASDR may improve in the future. By 2035, these ASR are projected to be 44.49per 100,000 persons (95% UI: 27.09 to 61.88), 5.88per 100,000 persons (95% UI: 3.94 to 7.82), 9.80per 100,000 persons (95% UI: 6.17 to 13.43), and 0.24 per 100,000, respectively persons (95% UI: 0.14 to 0.35). This suggests that although the number of cases will increase in the future, the ASR of disease burden may decrease as medical technology advances and disease management strategies are optimized (Figure 8, Supplementary Tables S11, S12).

## 4 Discussion

As a progressive cardiovascular disease, the impact of CAVD in working-age people has attracted increasing attention, and the impact of CAVD in working-age people has attracted increasing attention (8, 19, 20). The working-age population usually refers to those aged 15 to 64 who form the economic backbone of society and bear the main responsibility for production, services and social development (21). From an economic perspective, the 15 to 64 age bracket constitutes the core pillar of socio—economic production. An increment in the disease burden of CAVD within this age group exerts a direct and profound influence on productivity. The resultant decrease in labor input not only leads to economic losses at the enterprise level but also has far—reaching implications for the overall society. These losses can be manifested in terms of reduced output, increased production costs, and potential disruptions to supply chains, thereby impeding economic growth and development. From a social standpoint, the augmented disease burden among the 15 to 64 age cohort has significant implications for family dynamics and the broader social fabric. It substantially elevates the burden of family caregiving. This diversion of resources can disrupt the normal life order within the family, imposing restrictions on the educational and career opportunities of other family members. Consequently, alleviating the disease burden of CAVD in the working—age population is of utmost importance. It serves as a linchpin for maintaining social stability, ensuring the well—being of families, and safeguarding the sustainability and functionality of public health systems. Unhealthy lifestyles such as sedentary, high-fat diets and smoking are increasingly becoming major risk factors for CAVD due to globalization and industrialization. These patterns of behaviors are prevalent in the working-age group, making them a susceptible population with a high ASIR of CAVD (19, 22–24). This study examines the ASPR and burden of CAVD in the global working-age population. This paper analyzes the significant increase in the overall ASPR and ASIR of CAVD from 1992 to 2021 which reveals the multiple characteristics and driving factors of the burden of CAVD on a global scale. At the same time, this study used Joinpoint to analyze the trend of CAVD burden over time, which can accurately identify the key time inflection points, and provide strong support for grasping the phased characteristics of disease development. Furthermore, the study underscores the trends in ASDR and ASDALYs. Collectively, these two metrics demonstrate a consistent downward trajectory over the years. This analysis furnishes deeper insights into the influence of CAVD on ASDR and ASDALYs within the working—age population.

First, we observed that the global prevalence and incidence of CAVD in the working-age population has continued to increase over the past few decades. Based on the disaggregated analysis of this study, it can be observed that population growth and population aging are the main drivers of CAVD in working age. Decomposition analyses show that the increase in the proportion of the elderly population has had an impact on the high incidence of cardiovascular diseases, including CAVD, in the working-age population due to the overlap between the working-age population and a portion of the geriatric population, as well as changes in global demographics (25, 26). Secondly, the study also found that regional analyses of different SDIs further revealed the inequality of the burden of the disease. The prevalence and incidence of CAVD in High SDI regions is significantly higher than in Low SDI regions. In this study, we used the Spearman analysis to explore the correlation between CAVD and the age-standardized rate of SDI regions, which helped us quickly determine the direction and extent of the association between the two. From the above indicators, it can be seen that this difference may be related to higher health awareness, advanced medical testing methods, and unhealthy lifestyles, such as high-fat diets and sedentary lifestyles, in high SDI areas. In particular, the impact of advances in cardiac imaging techniques (e.g., echocardiography) over the past 30 years on disease detection may be mentioned. This may have confounded estimates of prevalence between different countries and time periods. It may be suggested that these technological advances may have led to an increase in detection rates rather than a true increase in the burden of disease. This study found that some developed countries in Europe are the hardest hit by the burden of CAVD, which may be closely related to lifestyle and dietary habits in Europe, where hypertension and diabetes are more prevalent, both of which are risk factors for CAVD (25, 27–29). In addition, with sustained mechanical stress, aortic valve endothelial cells become susceptible to damage, increasing the penetration of lipids and the infiltration of inflammatory cells. This process activates oxidative stress and chronic inflammation, causing valvular stromal cells to exhibit osteoblastic features, thereby accelerating calcification and valve dysfunction, and these pathological changes can lead to collagen fibrillary hyperplasia and calcium deposition, promote CAVD, and multiple studies support this association (30–32). CAVD and atherosclerosis have similar pathological mechanisms, including lipid deposition, inflammatory response, and calcification, so prolonged high cholesterol intake may also accelerate the development of CAVD (1, 33–35). Alexia et al. (36) argue that, Lipid deposition plays a key role in aortic valve lesions, and the accumulation of lipid substances triggers inflammation and promotes the calcification process. This inflammatory environment further triggers the activation of osteogenic signals, which promote the deposition of calcium salts in the valve tissue. Some studies have similarly suggested that this pathological process is similar to the early stages of atherosclerosis, especially in terms of lipid retention and chronic inflammation (32, 37). According to ASDALYs and ASDR, the decline from 1998 to 2021 is the most obvious, which may be closely related to the improvement of treatment methods and the improvement of medical conditions. However, this phenomenon also reveals new challenges, particularly in terms of the need for differentiated management of growing population sizes and disease burdens.

In terms of gender differences, the burden of CAVD is consistently higher in men than in women, especially in terms of ASPR and ASIR. This sex difference may be related to physiological differences and higher rates of exposure to cardiovascular risk factors in men, such as smoking, alcohol consumption, and unhealthy eating habits (38). From the analysis of health inequality, and the disease burden is more concentrated in high SDI regions. This phenomenon reflects a significant imbalance in the global allocation of health resources, suggesting the need to strengthen basic health facilities and health interventions in low SDI regions to reduce the burden of disease.

Future trend projections show that the total number of CAVD cases will continue to grow in 2035, despite the expected decline in ASPR and ASIR. This projection reinforces the urgency of strengthening disease prevention and control, especially among high-risk working-age populations.

In conclusion, this study reveals significant prevalence trends and inequalities in the health burden of CAVD among working-age people. Despite progress in reducing disease disability and mortality in recent years, prevention and intervention efforts are needed globally, especially in Low SDI regions, to address this complex public health challenge.

## 5 Advantages

The innovation of this study is reflected in its focus on a specific population. Previous studies mostly concentrated on the cardiovascular disease burden of the general population. In contrast, this study focuses on the working—age population aged 15–64, deeply exploring the prevalence trends and disease burden of CAVD in this group, thus filling the gap in the research on specific populations in this field. This study uses the BAPC model to predict the disease burden of CAVD in the global working—age population from 2022 to 2035, which is relatively rare in similar studies. The data of this study are sourced from the GBD 2021 study. It covers data from 204 countries and regions in various aspects. With a large sample size and global representativeness, the research results are more reliable and persuasive. This study analyzes the epidemiological characteristics and patterns of CAVD through multiple statistical methods, endowing the study with certain practicality.

## 6 Limitations

Previous studies have already utilized the GBD 2019 database to research related diseases. Although this article employs the latest GBD 2021 database, in some regions, especially those with scarce medical resources and imperfect statistical systems, the accuracy and integrity of the data may be poor. To a certain extent, this will affect the accuracy of global disease burden estimates. This study does not fully consider the impact of environmental factors on CAVD. At the same time, some confounding factors, such as individual genetic susceptibility and psychological stress, are difficult to accurately measure and control in the study. These factors may interfere with the analysis of factors related to the CAVD burden, making it impossible to comprehensively reveal the causes and pathogenesis of the disease. The study is only based on the historical data from 1992 to 2021 for analysis and prediction, lacking a prospective research design.

## Data Availability

The datasets presented in this study can be found in online repositories. The names of the repository/repositories and accession number(s) can be found in the article/Supplementary Material.
