# Supplementary material for: Trends in the global burden of aortic valve calcification disease in the working-age population from 1992 to 2021
Source: Front Cardiovasc Med. 2025 Aug 12;12:1544273. doi: 10.3389/fcvm.2025.1544273 (PMC12379075; doi:10.3389/fcvm.2025.1544273)
Supplement: Supplementary file 1 [file Datasheet1.pdf]

Supplementary material

Method&Formulate

A disability-adjusted life year (DALY) is the sum of a lost life year and a disability life year. DALY is calculated as:

$$DALY_{a,g,e,t} = \sum_{k=1}^{20} a_{k,t} * p_t * e_{k,t}$$

($DALY_{a,g,e,t}$ represents DALYs number cumulated by population aging, population growth and epidemiologic changes in year t, $a_{k,t}$ is the proportion ofpopulation for the age group k at year t, p_t is the population size at year t, and $e_{k,t}$ is represented by DALYs rate for a specific age group k at year t.)

Age-standardized rate estimates and counts per 100,000 population are presented according to the GBD Population Standard Framework, with 95% confidence intervals for all projections. ASR is calculated per 100,000 people using the following formula:

$$ASR = \frac{\sum_{i=1}^{A} a_i w_i}{\sum_{i=1}^{A} w_i} * 1000{,}000$$

(a_i: the age-specific rate in ith the age group; w_i: the number of people in the corresponding ith age group among the standard population; A: the number of age groups).

To assess ASR trends in incidence, mortality, disability-adjusted life years (DALYs), and prevalence of CAVD, an estimated annual percentage change (EAPC) was used. EAPC is used to measure the trend of ASR over time and is calculated as follows [13]:

$$\text{EAPC} = 100 \times [exp(\beta) - 1]$$

(β : the annual rate of change of ASR). The 95% confidence interval for EAPC was calculated using a linear regression model. If both the EAPC and its lower bound of the 95% confidence interval are positive, it indicates an upward trend in ASR; Conversely, if the upper limit is negative, the ASR is trending downward. If the above conditions are not met, the ASR is considered to be relatively stable during the analysis period.

Based on the temporal characteristics of disease distribution, the Jointpoint model uses the method of segmented regression to divide the time series data into different intervals by identifying the change points in the data. Within each interval, the model trend-fitted the data and optimized it for slope changes in each segment to accurately capture significant changes in disease burden over time.The formula for the Jointpoint regression model is as follows:：

$$y_i = \beta_0 + \beta_1 t_i + \sum_{j=1}^{k} \gamma_j (t_i - \tau_j)_+ + \varepsilon_i$$

(β_0, β_1: the gaps and slopes of linear regression, γ_j:the slope change at the j inflection point τ_j, $t_i - \tau_j$, Otherwise, it is 0， ε_i:the error terms).

The decomposition analysis formula is as follows：

$$R_y = \sum\nolimits_a pop\ size_y \cdot \frac{pop\ age_{a,y}}{pop\ size_{y}} \cdot \frac{prevalence_{a,y}}{pop\ age_{a,y}} \cdot \frac{R_y}{prevalence_{a,y}}$$

(a: the age group, y: the year, R: indicator of disease burden for CAVD). By multiplying these four factors, we can more clearly identify and quantify the specific impact of each factor on the burden of CAVD in different regions and at different SDI

levels.

Age-period-cohort (APC) model

The model divides the factors affecting the burden of CAVD into three effects: age effect, period effect, and cohort effect.Key indicators of the model include: Net Drift: represents the annual average percentage change (AAPC) in age-adjusted CAVD burden (e.g., mortality), reflecting overall burden trends over time. Local Drift: The annual percentage change (APC) in the burden of CAVD in each age group during the study period, providing a snapshot of the change over time within each age group. Longitudinal age curve: used to represent the age effect, showing the expected age-specific burden for each age group after adjusting for the period effect on the basis of the reference cohort. Period RR: Represents a period effect and is used to describe the ratio of the burden of disease over a period relative to the reference period. Cohort Ratio (Cohort RR): Represents the cohort effect and is used to illustrate the ratio of the burden of CAVD in a given birth cohort relative to the burden of disease in the reference cohort.

Bayesian age-period-cohort modle

The Bayesian age-period-cohort (BAPC) model, combined with the nested Laplace approximation (INLA) method. was also used to predict future trends in the burden of CAVD with the formula:

$$\log(Y_{a,p}) = \mu + a_a + \beta_p + \gamma_c$$

($\log(Y_{a,p})$: the logarithmic transformation of a health metric, such as mortality, to ensure that the prediction is non-negative, μ: the population mean, which represents

the average across all ages, time periods, and cohorts, α_a the age effect, which reflects the impact of a specific age group, β_p : the period effect, which indicates the effect of a specific time period, γ_c: the cohort effect, which indicates the effect of the birth cohort, where c=p-a)
