# Supplementary material for: Trends in the global burden of aortic valve calcification disease in the working-age population from 1992 to 2021
Source: Front Cardiovasc Med. 2025 Aug 12;12:1544273. doi: 10.3389/fcvm.2025.1544273 (PMC12379075; doi:10.3389/fcvm.2025.1544273)
Supplement: Supplementary file 2 [file Datasheet2.pdf]

Supplementary Figure.1 Global and gender-specific spearman analysis of CAVD and SDI region within working-age population

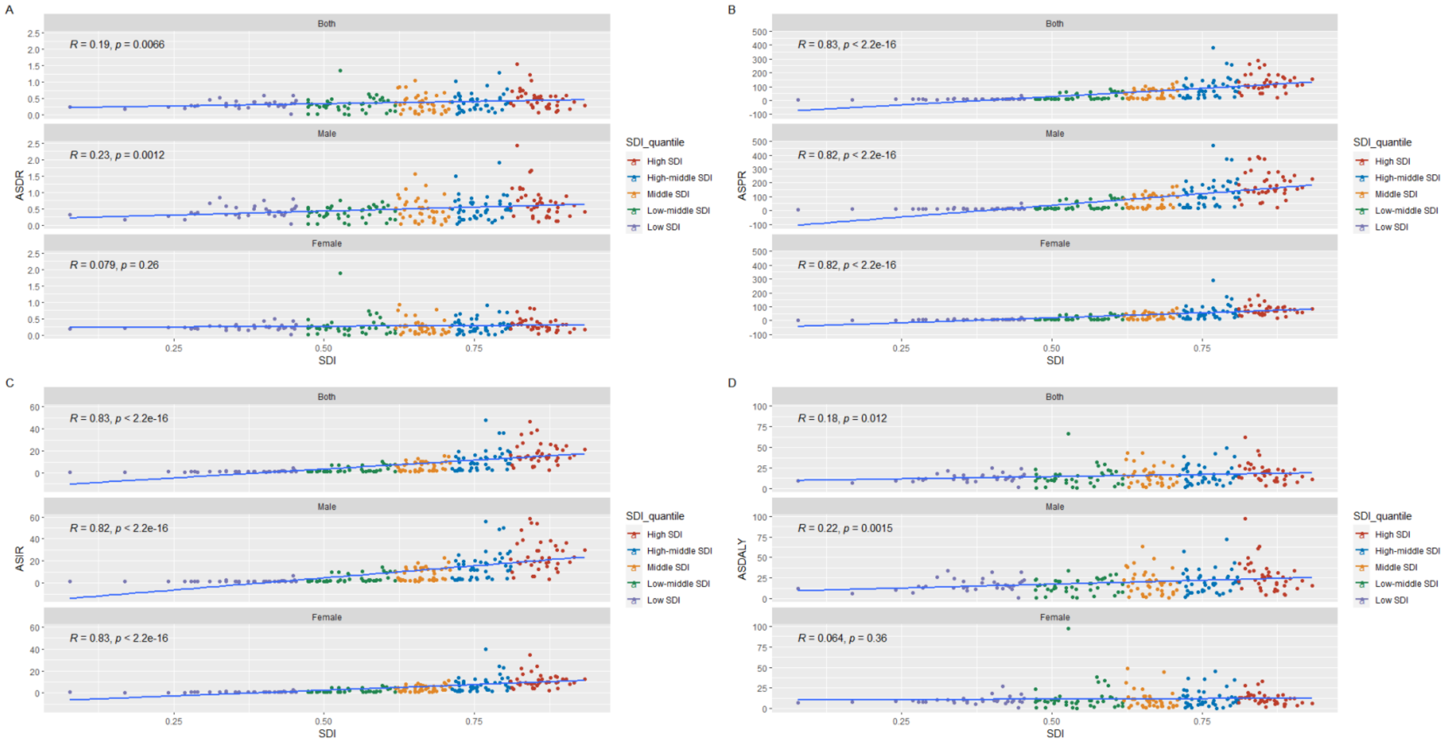

Abbreviation:SDI: Sociodemographic index; ASDALYs: Age-Standard Disability-Adjusted Life Years.

Supplementary Figure.2 World map of 204 countries and territories around the world with CAVD, disease burden and ASIR

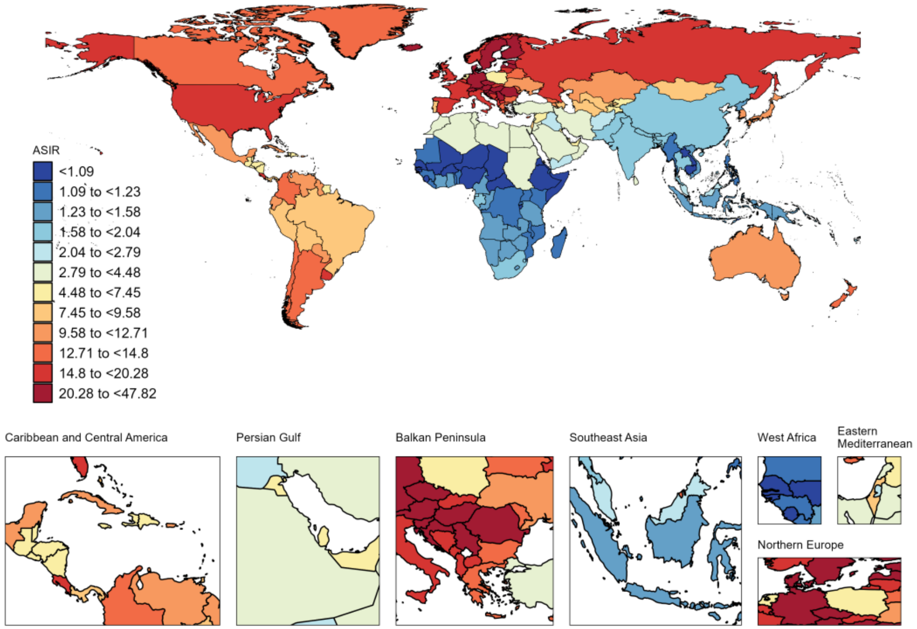

Abbreviation: ASIR: Age-standard incidence rate.

Supplementary Figure.3 World map of 204 countries and regions around the world with CAVD burden ASDALYs

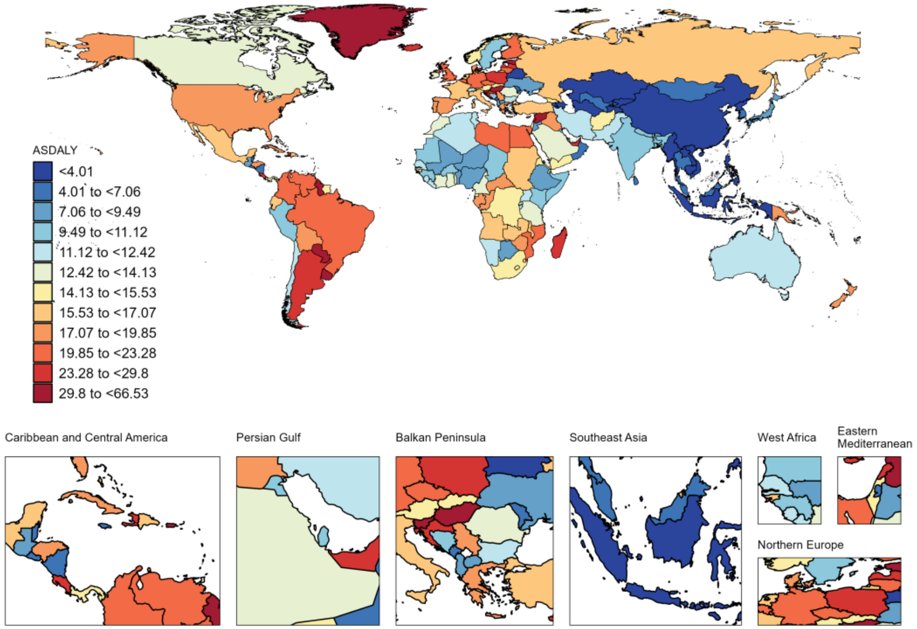

Abbreviation: ASDALY: Age-standard Disability-Adjusted Life Years rate.

Supplementary Figure.4 World map of CAVD burden for 204 countries and regions in the world

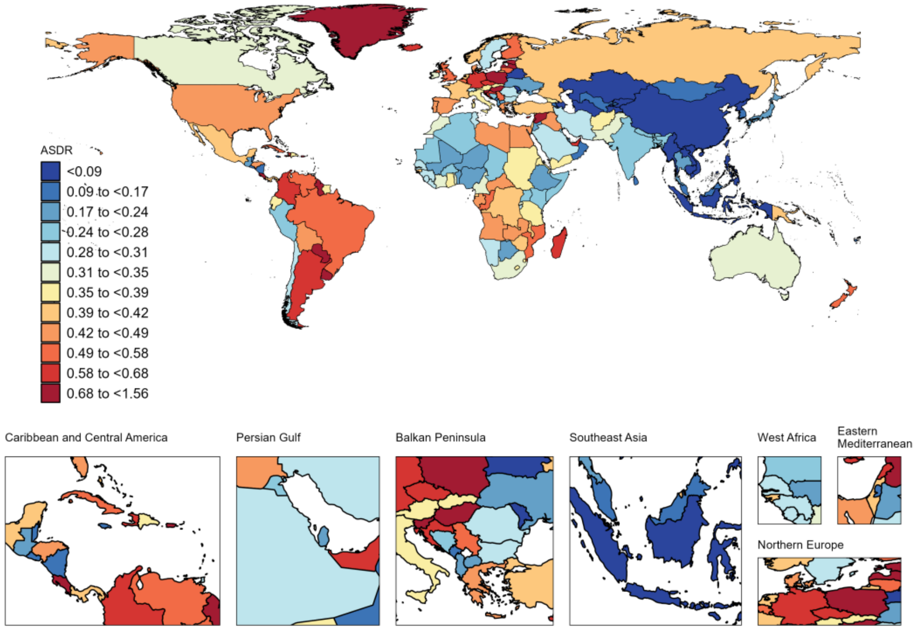

Abbreviation: ASDR: Age-standard death rate.

Supplementary Figure.5 Jionpoint model of the disease burden of CAVD in a global gender-specific working-age population

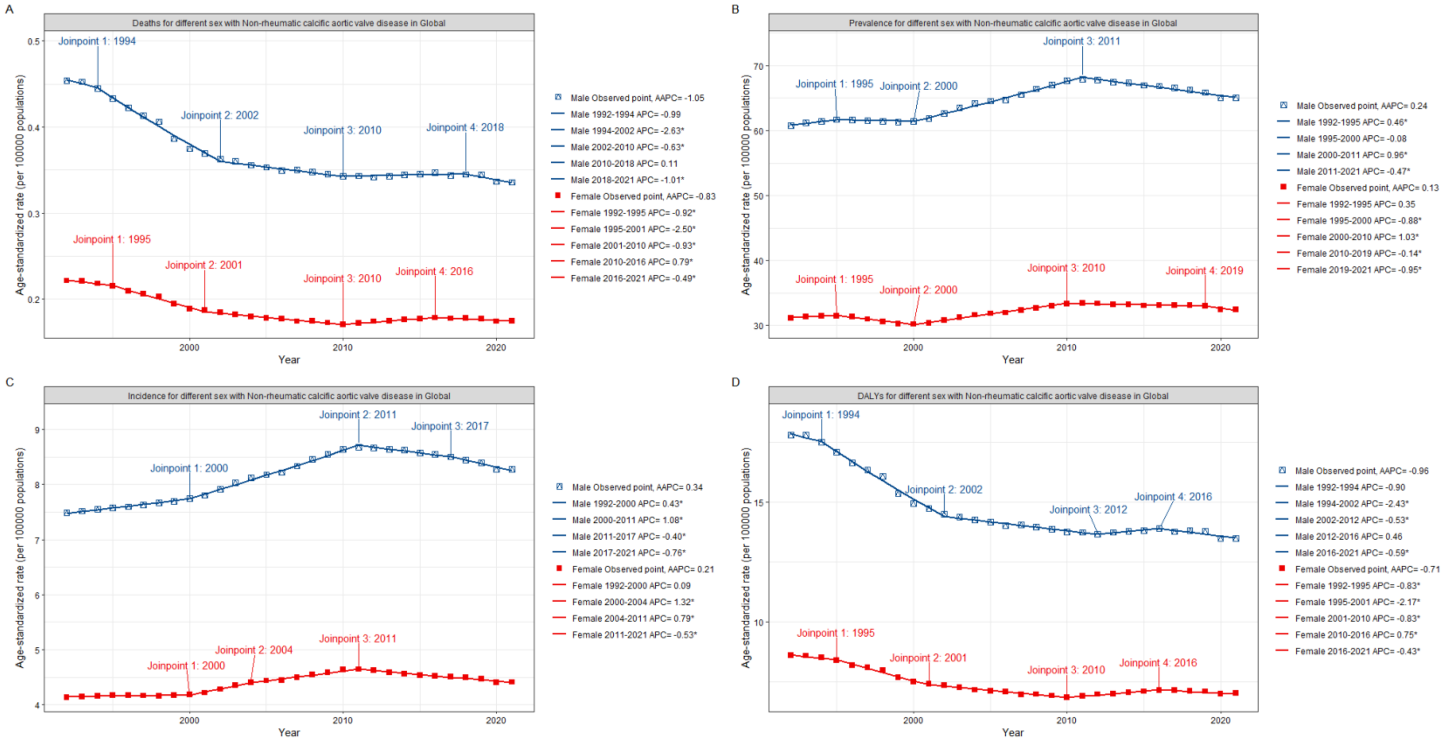

Abbreviation: AAPC: Average annual percent change; APC: Annual; percent change; DALYs: Disability-Adjusted Life Years.

Supplementary Figure 6: Frontier analysis of CAVD in the working-age population

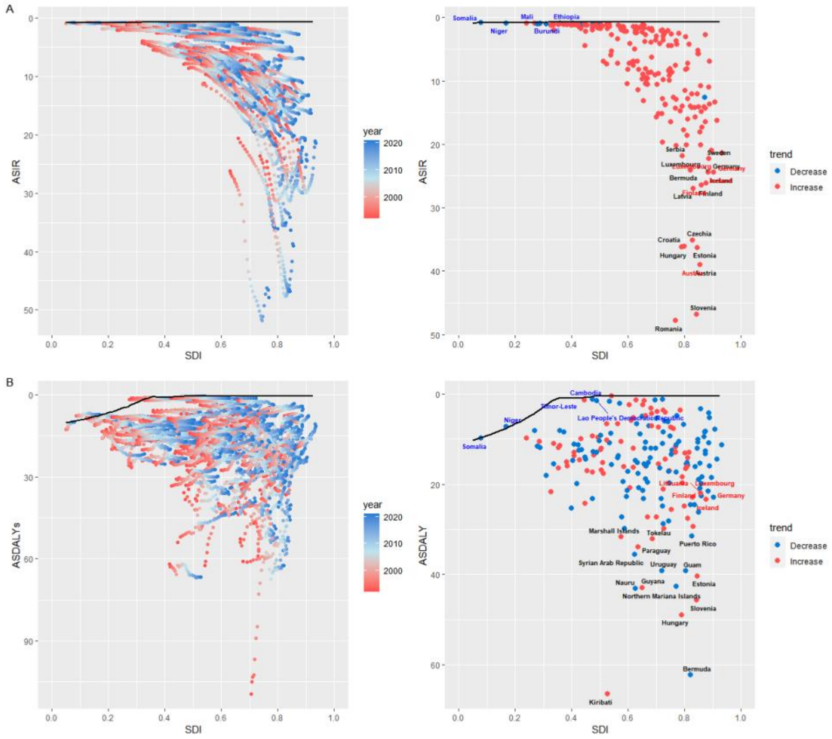

The boundaries of the frontier analysis are marked in solid black, and countries and regions are shown as points. The red and blue gradients are used to show the SDI and rate trends for each country or region. The red dot indicates an increase in the burden of disease between 1992 and 2021, and the blue dot indicates an increase in the burden of disease during this period. The top 15 countries (the countries with the highest leading values for CAVD gap in the working-age population) are marked in black. Countries that are far from the frontier analysis are marked with red names. Countries with fewer gaps are marked with blue names compared to cutting-edge analysis. Abbreviation: SDI: Sociodemographic index; ASIR: Age-standard incidence rate; ASDALYs: Age-standard Disability-Adjusted Life Years rate.
