# Supplementary material for: Trends in the global burden of aortic valve calcification disease in the working-age population from 1992 to 2021
Source: Front Cardiovasc Med. 2025 Aug 12;12:1544273. doi: 10.3389/fcvm.2025.1544273 (PMC12379075; doi:10.3389/fcvm.2025.1544273)
Supplement: Supplementary file 3 [file Datasheet3.zip › Supplementary Table 12.PDF]

Supplementary

Table S12. Global Burden of Aortic Valve Calcification Disease Burden in Working Age Range (Rate)

Measure	Sex	Year	Predict value	Predict low	Predict up
Deaths	Male	1992	0.457033574	0.447844865	0.466222282
Deaths	Male	1993	0.450625932	0.443084056	0.458167807
Deaths	Male	1994	0.442282538	0.435197174	0.449367902
Deaths	Male	1995	0.432037417	0.425128038	0.438946795
Deaths	Male	1996	0.421710256	0.414962	0.428458513
Deaths	Male	1997	0.411260856	0.404689622	0.417832089
Deaths	Male	1998	0.401529777	0.395122322	0.407937232
Deaths	Male	1999	0.388323742	0.382123383	0.394524102
Deaths	Male	2000	0.37797896	0.371922953	0.384034967
Deaths	Male	2001	0.370674746	0.364732956	0.376616535
Deaths	Male	2002	0.364424851	0.358596586	0.370253117
Deaths	Male	2003	0.36037264	0.354632969	0.366112312
Deaths	Male	2004	0.356110177	0.350461346	0.361759008
Deaths	Male	2005	0.35313228	0.347554251	0.358710309
Deaths	Male	2006	0.350302269	0.344795802	0.355808736
Deaths	Male	2007	0.348971468	0.343528445	0.354414491
Deaths	Male	2008	0.347014555	0.341646975	0.352382135
Deaths	Male	2009	0.34524818	0.339958638	0.350537722
Deaths	Male	2010	0.343413873	0.338191661	0.348636085
Deaths	Male	2011	0.343243458	0.33806204	0.348424876

Deaths	Male	2012	0.342645598	0.337511769	0.347779426
Deaths	Male	2013	0.343217828	0.338122608	0.348313049
Deaths	Male	2014	0.34424558	0.339172922	0.349318238
Deaths	Male	2015	0.344875104	0.339824923	0.349925284
Deaths	Male	2016	0.345173431	0.340132289	0.350214573
Deaths	Male	2017	0.343782144	0.338775761	0.348788527
Deaths	Male	2018	0.343727024	0.338749088	0.34870496
Deaths	Male	2019	0.342518358	0.337570984	0.347465731
Deaths	Male	2020	0.338407726	0.333345695	0.343469756
Deaths	Male	2021	0.336068229	0.330131917	0.342004541
Deaths	Male	2022	0.332994897	0.321475068	0.344514726
Deaths	Male	2023	0.329960881	0.31356247	0.346359292
Deaths	Male	2024	0.32683077	0.304570314	0.349091225
Deaths	Male	2025	0.323751584	0.294891527	0.352611642
Deaths	Male	2026	0.320916225	0.284822042	0.357010408
Deaths	Male	2027	0.318430192	0.27452299	0.362337394
Deaths	Male	2028	0.316378537	0.264150102	0.368606973
Deaths	Male	2029	0.314736075	0.253710779	0.375761372
Deaths	Male	2030	0.313457142	0.24316686	0.383747423
Deaths	Male	2031	0.312483224	0.232456315	0.392510133
Deaths	Male	2032	0.311706282	0.221486831	0.401925733
Deaths	Male	2033	0.31106045	0.210237099	0.411883801
Deaths	Male	2034	0.31045399	0.198661378	0.422246603
Deaths	Male	2035	0.309828105	0.186733526	0.432922684

Deaths	Female	1992	0.223139788	0.21705489	0.229224687
Deaths	Female	1993	0.220184902	0.215239558	0.225130245
Deaths	Female	1994	0.216796011	0.212251658	0.221340364
Deaths	Female	1995	0.213274055	0.208858698	0.217689412
Deaths	Female	1996	0.208883533	0.20456884	0.213198226
Deaths	Female	1997	0.204719562	0.200505022	0.208934102
Deaths	Female	1998	0.200448907	0.196336994	0.20456082
Deaths	Female	1999	0.195148722	0.191152895	0.19914455
Deaths	Female	2000	0.190728586	0.186821293	0.194635879
Deaths	Female	2001	0.187495079	0.183658637	0.19133152
Deaths	Female	2002	0.184595643	0.180829889	0.188361398
Deaths	Female	2003	0.181883254	0.178184218	0.185582289
Deaths	Female	2004	0.179652337	0.17601051	0.183294163
Deaths	Female	2005	0.177914707	0.174318067	0.181511347
Deaths	Female	2006	0.176404058	0.172854425	0.179953691
Deaths	Female	2007	0.174710717	0.171220277	0.178201157
Deaths	Female	2008	0.173851863	0.17040858	0.177295146
Deaths	Female	2009	0.172655117	0.169269268	0.176040966
Deaths	Female	2010	0.171964709	0.168622429	0.175306988
Deaths	Female	2011	0.172536737	0.169221141	0.175852332
Deaths	Female	2012	0.173592044	0.170289812	0.176894275
Deaths	Female	2013	0.174252669	0.170969721	0.177535617
Deaths	Female	2014	0.175497822	0.172212737	0.178782907
Deaths	Female	2015	0.17654439	0.173257316	0.179831465

Deaths	Female	2016	0.177050063	0.173767377	0.180332749
Deaths	Female	2017	0.176973128	0.173708534	0.180237722
Deaths	Female	2018	0.177130717	0.173881207	0.180380226
Deaths	Female	2019	0.176772054	0.173515439	0.180028669
Deaths	Female	2020	0.175254583	0.171846209	0.178662957
Deaths	Female	2021	0.174514198	0.170455429	0.178572967
Deaths	Female	2022	0.17341536	0.166297625	0.180533095
Deaths	Female	2023	0.172273209	0.162468543	0.182077876
Deaths	Female	2024	0.17104986	0.158033525	0.184066195
Deaths	Female	2025	0.169804664	0.15317854	0.186430787
Deaths	Female	2026	0.168617067	0.148038867	0.189195268
Deaths	Female	2027	0.167542012	0.142699975	0.192384049
Deaths	Female	2028	0.166635879	0.137254037	0.196017721
Deaths	Female	2029	0.165927685	0.131743683	0.200111687
Deaths	Female	2030	0.165437936	0.126186011	0.204689861
Deaths	Female	2031	0.165162757	0.120566798	0.209758716
Deaths	Female	2032	0.165054485	0.114838553	0.215270416
Deaths	Female	2033	0.16505576	0.108964152	0.221147368
Deaths	Female	2034	0.165104171	0.102903926	0.227304415
Deaths	Female	2035	0.165158781	0.096634419	0.233683144
Deaths	Both	1992	0.340594237	0.310706428	0.370482047
Deaths	Both	1993	0.335879816	0.306980002	0.36477963
Deaths	Both	1994	0.329968109	0.30171172	0.358224499
Deaths	Both	1995	0.323012878	0.295303268	0.350722488

Deaths	Both	1996	0.315571658	0.288422406	0.342720911
Deaths	Both	1997	0.308184036	0.281602307	0.334765764
Deaths	Both	1998	0.301124183	0.275099306	0.327149059
Deaths	Both	1999	0.291824289	0.26644575	0.317202829
Deaths	Both	2000	0.284396365	0.259556213	0.309236518
Deaths	Both	2001	0.279088109	0.254708342	0.303467876
Deaths	Both	2002	0.274486897	0.25055014	0.298423653
Deaths	Both	2003	0.271087584	0.247542506	0.294632662
Deaths	Both	2004	0.267826529	0.244660832	0.290992227
Deaths	Both	2005	0.265454494	0.2426122	0.288296787
Deaths	Both	2006	0.263249272	0.240729956	0.285768589
Deaths	Both	2007	0.261689475	0.239485584	0.283893366
Deaths	Both	2008	0.260212124	0.238313594	0.282110655
Deaths	Both	2009	0.258661671	0.237074947	0.280248395
Deaths	Both	2010	0.257315162	0.236003032	0.278627291
Deaths	Both	2011	0.257437478	0.236324962	0.278549994
Deaths	Both	2012	0.257614943	0.236657751	0.278572135
Deaths	Both	2013	0.258170569	0.237351356	0.278989782
Deaths	Both	2014	0.259256162	0.238531729	0.279980596
Deaths	Both	2015	0.260082808	0.239469083	0.280696533
Deaths	Both	2016	0.26048215	0.239962818	0.281001482
Deaths	Both	2017	0.259756554	0.239374292	0.280138816
Deaths	Both	2018	0.259812278	0.239539811	0.280084744
Deaths	Both	2019	0.259035644	0.238884442	0.279186845

Deaths	Both	2020	0.256229463	0.236205732	0.276253194
Deaths	Both	2021	0.254675146	0.234544642	0.27480565
Deaths	Both	2022	0.252647402	0.230129616	0.275165187
Deaths	Both	2023	0.250634476	0.226189352	0.2750796
Deaths	Both	2024	0.248567806	0.221270937	0.275864675
Deaths	Both	2025	0.246554584	0.215538679	0.277570488
Deaths	Both	2026	0.244735506	0.209207153	0.280263858
Deaths	Both	2027	0.243196527	0.202438395	0.283954659
Deaths	Both	2028	0.242017038	0.195401963	0.288632113
Deaths	Both	2029	0.241208307	0.188158931	0.294257683
Deaths	Both	2030	0.240765207	0.180719751	0.300810662
Deaths	Both	2031	0.240665309	0.173056987	0.308273632
Deaths	Both	2032	0.240839191	0.165102498	0.316575883
Deaths	Both	2033	0.24123057	0.156818626	0.325642514
Deaths	Both	2034	0.241772794	0.148149421	0.335396168
Deaths	Both	2035	0.242428896	0.1390496	0.345808192
Incidence	Male	1992	7.486298111	7.443148581	7.529447641
Incidence	Male	1993	7.519587604	7.479220944	7.559954265
Incidence	Male	1994	7.549711223	7.509567007	7.589855439
Incidence	Male	1995	7.578636454	7.53860239	7.618670518
Incidence	Male	1996	7.597708476	7.557863082	7.63755387
Incidence	Male	1997	7.627851156	7.588219473	7.667482839
Incidence	Male	1998	7.664680634	7.625229555	7.704131712
Incidence	Male	1999	7.701330372	7.662048687	7.740612058

Incidence	Male	2000	7.746158119	7.706999567	7.785316672
Incidence	Male	2001	7.810334907	7.771288768	7.849381046
Incidence	Male	2002	7.913360717	7.874389529	7.952331906
Incidence	Male	2003	8.0250533	7.986135214	8.063971386
Incidence	Male	2004	8.115349981	8.076596722	8.154103241
Incidence	Male	2005	8.178307143	8.139784373	8.216829913
Incidence	Male	2006	8.223773572	8.185600943	8.261946202
Incidence	Male	2007	8.336996517	8.299091681	8.374901353
Incidence	Male	2008	8.459460662	8.421806108	8.497115216
Incidence	Male	2009	8.549652208	8.512408224	8.586896192
Incidence	Male	2010	8.639166238	8.602247742	8.676084734
Incidence	Male	2011	8.676689746	8.640242809	8.713136683
Incidence	Male	2012	8.665266406	8.629292346	8.701240466
Incidence	Male	2013	8.63913337	8.603660694	8.674606046
Incidence	Male	2014	8.623335699	8.588259828	8.658411569
Incidence	Male	2015	8.577451337	8.542901545	8.612001128
Incidence	Male	2016	8.55123189	8.517066796	8.585396984
Incidence	Male	2017	8.503320727	8.469587087	8.537054368
Incidence	Male	2018	8.447395554	8.414079918	8.48071119
Incidence	Male	2019	8.38818307	8.355256623	8.421109517
Incidence	Male	2020	8.285431663	8.252920674	8.317942651
Incidence	Male	2021	8.27381245	8.240320733	8.307304168
Incidence	Male	2022	8.229596989	8.064693213	8.394500765
Incidence	Male	2023	8.142683275	7.891950946	8.393415604

Incidence	Male	2024	8.047116187	7.690627236	8.403605139
Incidence	Male	2025	7.953318146	7.477177829	8.429458463
Incidence	Male	2026	7.872465531	7.263740371	8.48119069
Incidence	Male	2027	7.804591681	7.049627922	8.559555441
Incidence	Male	2028	7.751690347	6.840157785	8.663222908
Incidence	Male	2029	7.710836219	6.633909892	8.787762545
Incidence	Male	2030	7.679021613	6.428334516	8.929708711
Incidence	Male	2031	7.654900424	6.221251283	9.088549564
Incidence	Male	2032	7.629732425	6.004084449	9.255380402
Incidence	Male	2033	7.604855386	5.780110152	9.429600621
Incidence	Male	2034	7.579313075	5.54977529	9.608850859
Incidence	Male	2035	7.552759503	5.313302441	9.792216566
Incidence	Female	1992	4.134884238	4.10291287	4.166855605
Incidence	Female	1993	4.148870953	4.119123082	4.178618823
Incidence	Female	1994	4.160022341	4.130499619	4.189545063
Incidence	Female	1995	4.167143711	4.137742119	4.196545303
Incidence	Female	1996	4.16411666	4.13493051	4.19330281
Incidence	Female	1997	4.162333191	4.133414801	4.191251582
Incidence	Female	1998	4.163385949	4.134689559	4.192082339
Incidence	Female	1999	4.169345236	4.140825303	4.197865169
Incidence	Female	2000	4.184498594	4.156102452	4.212894736
Incidence	Female	2001	4.21873578	4.190419269	4.247052292
Incidence	Female	2002	4.278703759	4.25040118	4.307006338
Incidence	Female	2003	4.348058696	4.319715578	4.376401815

Incidence	Female	2004	4.402717798	4.374429148	4.431006447
Incidence	Female	2005	4.432888609	4.404763041	4.461014178
Incidence	Female	2006	4.444667335	4.416844491	4.47249018
Incidence	Female	2007	4.495159987	4.467587513	4.522732461
Incidence	Female	2008	4.550037159	4.522722789	4.577351529
Incidence	Female	2009	4.588012274	4.561080337	4.614944212
Incidence	Female	2010	4.630358724	4.603722111	4.656995336
Incidence	Female	2011	4.641909623	4.61569327	4.668125976
Incidence	Female	2012	4.619820819	4.594035613	4.645606024
Incidence	Female	2013	4.587798409	4.562469892	4.613126927
Incidence	Female	2014	4.563810807	4.538843655	4.588777958
Incidence	Female	2015	4.531603601	4.507055309	4.556151893
Incidence	Female	2016	4.521346435	4.497067625	4.545625245
Incidence	Female	2017	4.507077129	4.483065431	4.531088826
Incidence	Female	2018	4.491003669	4.467239294	4.514768044
Incidence	Female	2019	4.468986245	4.445469161	4.49250333
Incidence	Female	2020	4.408165569	4.384950355	4.431380782
Incidence	Female	2021	4.408604919	4.384590701	4.432619137
Incidence	Female	2022	4.400441251	4.287088311	4.513794191
Incidence	Female	2023	4.355893634	4.186434248	4.52535302
Incidence	Female	2024	4.30634521	4.066956384	4.545734036
Incidence	Female	2025	4.257840799	3.938776727	4.576904871
Incidence	Female	2026	4.217067075	3.809573159	4.624560991
Incidence	Female	2027	4.18510679	3.680417084	4.689796497

Incidence	Female	2028	4.161407563	3.552330454	4.770484671
Incidence	Female	2029	4.143902295	3.424101163	4.863703427
Incidence	Female	2030	4.130806235	3.294289311	4.96732316
Incidence	Female	2031	4.121405664	3.161894992	5.080916337
Incidence	Female	2032	4.111845516	3.023449649	5.200241383
Incidence	Female	2033	4.102096285	2.879928309	5.324264262
Incidence	Female	2034	4.091583339	2.731552063	5.451614615
Incidence	Female	2035	4.080276234	2.578621353	5.581931115
Incidence	Both	1992	5.815065257	5.686420687	5.943709827
Incidence	Both	1993	5.838521704	5.711898675	5.965144733
Incidence	Both	1994	5.85861	5.732872571	5.984347429
Incidence	Both	1995	5.875366003	5.75039406	6.000337946
Incidence	Both	1996	5.881947567	5.758064013	6.005831121
Incidence	Both	1997	5.894522196	5.771941168	6.017103225
Incidence	Both	1998	5.912437188	5.791058708	6.033815668
Incidence	Both	1999	5.932640765	5.812383966	6.052897563
Incidence	Both	2000	5.961568127	5.842179478	6.080956777
Incidence	Both	2001	6.00994245	5.891243716	6.128641184
Incidence	Both	2002	6.090952868	5.972709533	6.209196204
Incidence	Both	2003	6.181457949	6.063545483	6.299370414
Incidence	Both	2004	6.253678033	6.136327698	6.371028368
Incidence	Both	2005	6.299838509	6.183235492	6.416441526
Incidence	Both	2006	6.327500827	6.211901295	6.443100359
Incidence	Both	2007	6.407937872	6.293147068	6.522728676

Incidence	Both	2008	6.494436336	6.380454895	6.608417778
Incidence	Both	2009	6.556599604	6.443703975	6.669495233
Incidence	Both	2010	6.620012162	6.508141521	6.731882804
Incidence	Both	2011	6.642321188	6.531696866	6.75294551
Incidence	Both	2012	6.623855033	6.514470608	6.733239458
Incidence	Both	2013	6.593009406	6.48495323	6.701065582
Incidence	Both	2014	6.571475692	6.464547056	6.678404327
Incidence	Both	2015	6.531918658	6.426316722	6.637520593
Incidence	Both	2016	6.513359185	6.408735982	6.617982388
Incidence	Both	2017	6.482212204	6.378573615	6.585850792
Incidence	Both	2018	6.446269843	6.34363063	6.548909057
Incidence	Both	2019	6.405711606	6.304073598	6.507349613
Incidence	Both	2020	6.323746056	6.22330145	6.424190662
Incidence	Both	2021	6.317115884	6.216827955	6.417403813
Incidence	Both	2022	6.292432552	6.078641019	6.506224086
Incidence	Both	2023	6.22800773	5.961940065	6.494075394
Incidence	Both	2024	6.157419497	5.817276391	6.497562602
Incidence	Both	2025	6.088851154	5.658101235	6.519601073
Incidence	Both	2026	6.031234897	5.495608983	6.566860811
Incidence	Both	2027	5.985222856	5.331017597	6.639428115
Incidence	Both	2028	5.951690954	5.168320684	6.735061224
Incidence	Both	2029	5.928324476	5.006557409	6.850091543
Incidence	Both	2030	5.91280583	4.843777397	6.981834262
Incidence	Both	2031	5.904236282	4.678356607	7.130115957

Incidence	Both	2032	5.896490084	4.504180459	7.288799709
Incidence	Both	2033	5.890210218	4.323197166	7.457223269
Incidence	Both	2034	5.884788911	4.135519096	7.634058726
Incidence	Both	2035	5.880298814	3.941170848	7.819426781
Prevalence	Male	1992	60.76331081	60.63578943	60.89083219
Prevalence	Male	1993	61.18085482	61.05552973	61.30617991
Prevalence	Male	1994	61.46323904	61.3384423	61.58803579
Prevalence	Male	1995	61.68514781	61.56090133	61.80939429
Prevalence	Male	1996	61.64326657	61.51993372	61.76659943
Prevalence	Male	1997	61.5334464	61.41123802	61.65565479
Prevalence	Male	1998	61.40092168	61.27983222	61.52201113
Prevalence	Male	1999	61.31929617	61.19929825	61.43929409
Prevalence	Male	2000	61.44907894	61.32988599	61.5682719
Prevalence	Male	2001	61.86840319	61.74986133	61.98694505
Prevalence	Male	2002	62.65839354	62.54028279	62.77650428
Prevalence	Male	2003	63.49519416	63.37752115	63.61286717
Prevalence	Male	2004	64.14217482	64.0253104	64.25903925
Prevalence	Male	2005	64.54100567	64.4250946	64.65691673
Prevalence	Male	2006	64.7573962	64.64286984	64.87192257
Prevalence	Male	2007	65.53971345	65.42634858	65.65307831
Prevalence	Male	2008	66.41269063	66.30041176	66.5249695
Prevalence	Male	2009	67.04285576	66.93207043	67.1536411
Prevalence	Male	2010	67.70952062	67.59997809	67.81906316
Prevalence	Male	2011	67.94254507	67.83460211	68.05048804

Prevalence	Male	2012	67.76719874	67.66079977	67.8735977
Prevalence	Male	2013	67.4934839	67.388695	67.5982728
Prevalence	Male	2014	67.33724224	67.2337207	67.44076378
Prevalence	Male	2015	66.97900389	66.8771086	67.08089919
Prevalence	Male	2016	66.84721013	66.74646075	66.9479595
Prevalence	Male	2017	66.56304351	66.46353763	66.66254938
Prevalence	Male	2018	66.2243362	66.12605301	66.3226194
Prevalence	Male	2019	65.85369811	65.75654842	65.9508478
Prevalence	Male	2020	65.07821124	64.98241396	65.17400851
Prevalence	Male	2021	65.01562887	64.91979993	65.11145781
Prevalence	Male	2022	64.36364646	63.19265226	65.53464065
Prevalence	Male	2023	63.59950421	61.6733836	65.52562481
Prevalence	Male	2024	62.76441414	59.9023979	65.62643038
Prevalence	Male	2025	61.94891196	58.02948213	65.86834179
Prevalence	Male	2026	61.25026134	56.16571352	66.33480915
Prevalence	Male	2027	60.66467282	54.30011569	67.02922996
Prevalence	Male	2028	60.20118227	52.4642436	67.93812095
Prevalence	Male	2029	59.83014999	50.64041549	69.01988448
Prevalence	Male	2030	59.53439455	48.81728597	70.25150313
Prevalence	Male	2031	59.3273456	47.00035494	71.65433626
Prevalence	Male	2032	59.12133236	45.10495252	73.1377122
Prevalence	Male	2033	58.88996417	43.12729619	74.65263215
Prevalence	Male	2034	58.62653528	41.07426412	76.17880645
Prevalence	Male	2035	58.34138861	38.9616675	77.72110972

Prevalence	Female	1992	31.15135752	31.0601207	31.24259434
Prevalence	Female	1993	31.34184769	31.25241454	31.43128085
Prevalence	Female	1994	31.44117351	31.35219182	31.53015519
Prevalence	Female	1995	31.48291437	31.39449108	31.57133766
Prevalence	Female	1996	31.29995984	31.21250067	31.38741901
Prevalence	Female	1997	30.96596067	30.87980886	31.05211248
Prevalence	Female	1998	30.58160067	30.49674023	30.66646112
Prevalence	Female	1999	30.28033645	30.19663292	30.36403997
Prevalence	Female	2000	30.19645725	30.11355442	30.27936007
Prevalence	Female	2001	30.37006417	30.28768609	30.45244225
Prevalence	Female	2002	30.76762052	30.68552872	30.84971232
Prevalence	Female	2003	31.23370915	31.15180857	31.31560974
Prevalence	Female	2004	31.59933271	31.51790953	31.6807559
Prevalence	Female	2005	31.82987713	31.74903642	31.91071784
Prevalence	Female	2006	31.9403686	31.86048936	32.02024785
Prevalence	Female	2007	32.30103655	32.22204487	32.38002824
Prevalence	Female	2008	32.69917803	32.62106941	32.77728665
Prevalence	Female	2009	33.00241698	32.92543799	33.07939597
Prevalence	Female	2010	33.32328088	33.24728542	33.39927634
Prevalence	Female	2011	33.44969247	33.37489922	33.52448571
Prevalence	Female	2012	33.36680464	33.29315108	33.44045821
Prevalence	Female	2013	33.23451395	33.16204687	33.30698104
Prevalence	Female	2014	33.16691314	33.09537542	33.23845087
Prevalence	Female	2015	33.04509765	32.97464785	33.11554744

Prevalence	Female	2016	33.0957824	33.0260109	33.16555391
Prevalence	Female	2017	33.13107972	33.06198169	33.20017776
Prevalence	Female	2018	33.12097258	33.05255161	33.18939355
Prevalence	Female	2019	33.00278408	32.9350776	33.07049055
Prevalence	Female	2020	32.46810542	32.40150895	32.5347019
Prevalence	Female	2021	32.45230955	32.38562369	32.51899541
Prevalence	Female	2022	32.23273581	31.43562371	33.02984791
Prevalence	Female	2023	31.84006011	30.49298686	33.18713337
Prevalence	Female	2024	31.41334502	29.38470928	33.44198075
Prevalence	Female	2025	31.00183923	28.20150444	33.80217403
Prevalence	Female	2026	30.65261536	27.00218594	34.30304479
Prevalence	Female	2027	30.36415113	25.78583302	34.94246924
Prevalence	Female	2028	30.13471249	24.56116954	35.70825544
Prevalence	Female	2029	29.95215538	23.32256625	36.58174452
Prevalence	Female	2030	29.8082313	22.06532008	37.55114251
Prevalence	Female	2031	29.70382434	20.78793487	38.61971382
Prevalence	Female	2032	29.59990829	19.45761126	39.74220531
Prevalence	Female	2033	29.48760013	18.07590471	40.89929556
Prevalence	Female	2034	29.36205765	16.64626544	42.07784987
Prevalence	Female	2035	29.22850314	15.17685257	43.28015371
Prevalence	Both	1992	45.96207996	45.58808234	46.33607759
Prevalence	Both	1993	46.26434443	45.89292131	46.63576755
Prevalence	Both	1994	46.45051802	46.0812531	46.81978294
Prevalence	Both	1995	46.57021775	46.20331017	46.93712532

Prevalence	Both	1996	46.44384255	46.08092271	46.80676238
Prevalence	Both	1997	46.20587198	45.84824333	46.56350063
Prevalence	Both	1998	45.93708763	45.58488329	46.28929196
Prevalence	Both	1999	45.73515696	45.38799882	46.08231511
Prevalence	Both	2000	45.74805559	45.40448832	46.09162286
Prevalence	Both	2001	46.03715891	45.69605387	46.37826394
Prevalence	Both	2002	46.62679973	46.28726723	46.96633223
Prevalence	Both	2003	47.28075832	46.94254153	47.61897511
Prevalence	Both	2004	47.78705274	47.45086288	48.12324259
Prevalence	Both	2005	48.10274839	47.76897489	48.4365219
Prevalence	Both	2006	48.26180214	47.93122172	48.59238256
Prevalence	Both	2007	48.82307662	48.49518684	49.15096641
Prevalence	Both	2008	49.44064526	49.11542862	49.7658619
Prevalence	Both	2009	49.89201228	49.57017125	50.21385332
Prevalence	Both	2010	50.36227246	50.04374282	50.6808021
Prevalence	Both	2011	50.52149415	50.20680698	50.83618133
Prevalence	Both	2012	50.37783659	50.0668618	50.68881138
Prevalence	Both	2013	50.15993347	49.85284592	50.46702102
Prevalence	Both	2014	50.03432877	49.73049749	50.33816006
Prevalence	Both	2015	49.79135553	49.49131152	50.09139953
Prevalence	Both	2016	49.74944299	49.45211546	50.04677052
Prevalence	Both	2017	49.62578391	49.33116264	49.92040518
Prevalence	Both	2018	49.45221466	49.16039504	49.74403427
Prevalence	Both	2019	49.20737212	48.91850574	49.49623851

Prevalence	Both	2020	48.54870171	48.26380698	48.83359644
Prevalence	Both	2021	48.49821979	48.21500118	48.7814384
Prevalence	Both	2022	48.07378659	46.74525072	49.40232246
Prevalence	Both	2023	47.50680393	45.64513196	49.3684759
Prevalence	Both	2024	46.89404036	44.29483681	49.4932439
Prevalence	Both	2025	46.30533023	42.83129677	49.77936369
Prevalence	Both	2026	45.81315248	41.35193479	50.27437018
Prevalence	Both	2027	45.41599633	39.85724057	50.97475208
Prevalence	Both	2028	45.11952612	38.37147113	51.86758111
Prevalence	Both	2029	44.90496755	36.88401641	52.92591869
Prevalence	Both	2030	44.76030473	35.38534045	54.13526902
Prevalence	Both	2031	44.69376832	33.87547457	55.51206207
Prevalence	Both	2032	44.64371231	32.29304933	56.99437529
Prevalence	Both	2033	44.59285321	30.63322037	58.55248606
Prevalence	Both	2034	44.53725632	28.89721984	60.17729279
Prevalence	Both	2035	44.48723684	27.09295501	61.88151867
DALYs (Disability-Adjusted Life Years)	Male	1992	17.81407301	17.74686382	17.88128219
DALYs (Disability-Adjusted Life Years)	Male	1993	17.79145926	17.72562429	17.85729424
DALYs (Disability-Adjusted Life Years)	Male	1994	17.50421442	17.43948817	17.56894067
DALYs (Disability-Adjusted Life Years)	Male	1995	17.08430309	17.02085814	17.14774805
DALYs (Disability-Adjusted Life Years)	Male	1996	16.66058492	16.59846365	16.72270619
DALYs (Disability-Adjusted Life Years)	Male	1997	16.34799575	16.28704711	16.4089444
DALYs (Disability-Adjusted Life Years)	Male	1998	16.08286574	16.02297973	16.14275174
DALYs (Disability-Adjusted Life Years)	Male	1999	15.3658297	15.30792674	15.42373267

DALYs (Disability-Adjusted Life Years)	Male	2000	14.95359818	14.89701582	15.01018053
DALYs (Disability-Adjusted Life Years)	Male	2001	14.75039207	14.69474641	14.80603773
DALYs (Disability-Adjusted Life Years)	Male	2002	14.50748719	14.45289713	14.56207725
DALYs (Disability-Adjusted Life Years)	Male	2003	14.39638006	14.3425749	14.45018522
DALYs (Disability-Adjusted Life Years)	Male	2004	14.26144045	14.20853554	14.31434537
DALYs (Disability-Adjusted Life Years)	Male	2005	14.17821871	14.12604364	14.23039378
DALYs (Disability-Adjusted Life Years)	Male	2006	14.03626135	13.98496133	14.08756136
DALYs (Disability-Adjusted Life Years)	Male	2007	14.06790389	14.01721456	14.11859322
DALYs (Disability-Adjusted Life Years)	Male	2008	13.97319177	13.92334027	14.02304327
DALYs (Disability-Adjusted Life Years)	Male	2009	13.87809791	13.82909937	13.92709645
DALYs (Disability-Adjusted Life Years)	Male	2010	13.75716262	13.70900347	13.80532176
DALYs (Disability-Adjusted Life Years)	Male	2011	13.74887935	13.70131061	13.7964481
DALYs (Disability-Adjusted Life Years)	Male	2012	13.68178571	13.63481938	13.72875204
DALYs (Disability-Adjusted Life Years)	Male	2013	13.74469329	13.69809379	13.7912928
DALYs (Disability-Adjusted Life Years)	Male	2014	13.79758261	13.75129023	13.84387499
DALYs (Disability-Adjusted Life Years)	Male	2015	13.83182062	13.78591865	13.87772259
DALYs (Disability-Adjusted Life Years)	Male	2016	13.89998276	13.85433168	13.94563383
DALYs (Disability-Adjusted Life Years)	Male	2017	13.77633149	13.7312532	13.82140977
DALYs (Disability-Adjusted Life Years)	Male	2018	13.81558434	13.77079247	13.86037622
DALYs (Disability-Adjusted Life Years)	Male	2019	13.78777412	13.74336392	13.83218432
DALYs (Disability-Adjusted Life Years)	Male	2020	13.50608767	13.46241963	13.54975572
DALYs (Disability-Adjusted Life Years)	Male	2021	13.49411307	13.45028378	13.53794237
DALYs (Disability-Adjusted Life Years)	Male	2022	13.30170192	12.9412035	13.66220035
DALYs (Disability-Adjusted Life Years)	Male	2023	13.20115512	12.67411805	13.72819219

DALYs (Disability-Adjusted Life Years)	Male	2024	13.09709868	12.35900263	13.83519474
DALYs (Disability-Adjusted Life Years)	Male	2025	12.99232452	12.01138462	13.97326441
DALYs (Disability-Adjusted Life Years)	Male	2026	12.89491564	11.64454331	14.14528798
DALYs (Disability-Adjusted Life Years)	Male	2027	12.80706649	11.26320187	14.3509311
DALYs (Disability-Adjusted Life Years)	Male	2028	12.73758632	10.87893972	14.59623292
DALYs (Disability-Adjusted Life Years)	Male	2029	12.68262925	10.48997426	14.87528424
DALYs (Disability-Adjusted Life Years)	Male	2030	12.63728544	10.09269773	15.18187316
DALYs (Disability-Adjusted Life Years)	Male	2031	12.5991854	9.68491521	15.51345559
DALYs (Disability-Adjusted Life Years)	Male	2032	12.56239091	9.261711819	15.86307001
DALYs (Disability-Adjusted Life Years)	Male	2033	12.53149475	8.828285109	16.23470438
DALYs (Disability-Adjusted Life Years)	Male	2034	12.50056497	8.381411638	16.6197183
DALYs (Disability-Adjusted Life Years)	Male	2035	12.46388132	7.918112591	17.00965005
DALYs (Disability-Adjusted Life Years)	Female	1992	8.608516993	8.562110369	8.654923616
DALYs (Disability-Adjusted Life Years)	Female	1993	8.588583324	8.543491352	8.633675296
DALYs (Disability-Adjusted Life Years)	Female	1994	8.499237916	8.454827904	8.543647928
DALYs (Disability-Adjusted Life Years)	Female	1995	8.38351603	8.339744333	8.427287726
DALYs (Disability-Adjusted Life Years)	Female	1996	8.187117354	8.144263895	8.229970812
DALYs (Disability-Adjusted Life Years)	Female	1997	8.086980244	8.044806743	8.129153746
DALYs (Disability-Adjusted Life Years)	Female	1998	7.973566025	7.932104611	8.01502744
DALYs (Disability-Adjusted Life Years)	Female	1999	7.686663326	7.646428036	7.726898616
DALYs (Disability-Adjusted Life Years)	Female	2000	7.506530867	7.467137427	7.545924307
DALYs (Disability-Adjusted Life Years)	Female	2001	7.419696238	7.380897052	7.458495423
DALYs (Disability-Adjusted Life Years)	Female	2002	7.352543501	7.314340099	7.390746903
DALYs (Disability-Adjusted Life Years)	Female	2003	7.257769268	7.220218891	7.295319645

DALYs (Disability-Adjusted Life Years)	Female	2004	7.166965351	7.130073539	7.203857163
DALYs (Disability-Adjusted Life Years)	Female	2005	7.10969609	7.073327617	7.146064562
DALYs (Disability-Adjusted Life Years)	Female	2006	7.075839835	7.039989489	7.111690181
DALYs (Disability-Adjusted Life Years)	Female	2007	6.968805902	6.933723455	7.003888348
DALYs (Disability-Adjusted Life Years)	Female	2008	6.98202785	6.947391673	7.016664027
DALYs (Disability-Adjusted Life Years)	Female	2009	6.920188405	6.886224897	6.954151912
DALYs (Disability-Adjusted Life Years)	Female	2010	6.862994462	6.829634787	6.896354136
DALYs (Disability-Adjusted Life Years)	Female	2011	6.900894147	6.867860848	6.933927445
DALYs (Disability-Adjusted Life Years)	Female	2012	6.979942791	6.947053321	7.01283226
DALYs (Disability-Adjusted Life Years)	Female	2013	7.006363575	6.97374893	7.038978219
DALYs (Disability-Adjusted Life Years)	Female	2014	7.052741632	7.020293006	7.085190257
DALYs (Disability-Adjusted Life Years)	Female	2015	7.101521473	7.069258221	7.133784725
DALYs (Disability-Adjusted Life Years)	Female	2016	7.146839381	7.114718926	7.178959837
DALYs (Disability-Adjusted Life Years)	Female	2017	7.132297774	7.100456291	7.164139257
DALYs (Disability-Adjusted Life Years)	Female	2018	7.116834094	7.085263028	7.148405161
DALYs (Disability-Adjusted Life Years)	Female	2019	7.099687269	7.068376926	7.130997613
DALYs (Disability-Adjusted Life Years)	Female	2020	7.001047369	6.97011329	7.031981448
DALYs (Disability-Adjusted Life Years)	Female	2021	7.022357685	6.991054181	7.053661189
DALYs (Disability-Adjusted Life Years)	Female	2022	6.957301197	6.763985255	7.150617139
DALYs (Disability-Adjusted Life Years)	Female	2023	6.929912099	6.660017534	7.199806663
DALYs (Disability-Adjusted Life Years)	Female	2024	6.899010554	6.531059924	7.266961183
DALYs (Disability-Adjusted Life Years)	Female	2025	6.867782033	6.385853403	7.349710663
DALYs (Disability-Adjusted Life Years)	Female	2026	6.839855651	6.230380896	7.449330406
DALYs (Disability-Adjusted Life Years)	Female	2027	6.816270853	6.066817345	7.565724361

DALYs (Disability-Adjusted Life Years)	Female	2028	6.80018119	5.899989715	7.700372666
DALYs (Disability-Adjusted Life Years)	Female	2029	6.789635563	5.729048228	7.850222899
DALYs (Disability-Adjusted Life Years)	Female	2030	6.784590575	5.554163903	8.015017246
DALYs (Disability-Adjusted Life Years)	Female	2031	6.785671094	5.375455807	8.19588638
DALYs (Disability-Adjusted Life Years)	Female	2032	6.791345401	5.191186313	8.39150449
DALYs (Disability-Adjusted Life Years)	Female	2033	6.802099499	5.002361893	8.601837105
DALYs (Disability-Adjusted Life Years)	Female	2034	6.813126857	4.805712865	8.820540849
DALYs (Disability-Adjusted Life Years)	Female	2035	6.821891264	4.599674958	9.044107571
DALYs (Disability-Adjusted Life Years)	Both	1992	13.23991942	13.00657354	13.4732653
DALYs (Disability-Adjusted Life Years)	Both	1993	13.21808603	12.98702358	13.44914848
DALYs (Disability-Adjusted Life Years)	Both	1994	13.02757004	12.7999587	13.25518137
DALYs (Disability-Adjusted Life Years)	Both	1995	12.75718829	12.53357989	12.98079669
DALYs (Disability-Adjusted Life Years)	Both	1996	12.44341556	12.2243317	12.66249942
DALYs (Disability-Adjusted Life Years)	Both	1997	12.23401977	12.01846012	12.44957942
DALYs (Disability-Adjusted Life Years)	Both	1998	12.0419265	11.82984986	12.25400314
DALYs (Disability-Adjusted Life Years)	Both	1999	11.53710794	11.33112677	11.7430891
DALYs (Disability-Adjusted Life Years)	Both	2000	11.23894656	11.03721078	11.44068234
DALYs (Disability-Adjusted Life Years)	Both	2001	11.09216731	10.89360959	11.29072502
DALYs (Disability-Adjusted Life Years)	Both	2002	10.93561867	10.74033672	11.13090062
DALYs (Disability-Adjusted Life Years)	Both	2003	10.83118216	10.63878073	11.0235836
DALYs (Disability-Adjusted Life Years)	Both	2004	10.71799723	10.52845864	10.90753583
DALYs (Disability-Adjusted Life Years)	Both	2005	10.64722527	10.46018107	10.83426947
DALYs (Disability-Adjusted Life Years)	Both	2006	10.55776086	10.37336364	10.74215809
DALYs (Disability-Adjusted Life Years)	Both	2007	10.51826268	10.33644834	10.70007702

DALYs (Disability-Adjusted Life Years)	Both	2008	10.47503249	10.29559617	10.65446881
DALYs (Disability-Adjusted Life Years)	Both	2009	10.39353798	10.21685916	10.5702168
DALYs (Disability-Adjusted Life Years)	Both	2010	10.30140876	10.12738085	10.47543668
DALYs (Disability-Adjusted Life Years)	Both	2011	10.31337373	10.14109935	10.48564811
DALYs (Disability-Adjusted Life Years)	Both	2012	10.31772037	10.14682844	10.48861231
DALYs (Disability-Adjusted Life Years)	Both	2013	10.36037674	10.19047783	10.53027566
DALYs (Disability-Adjusted Life Years)	Both	2014	10.40856319	10.23960538	10.577521
DALYs (Disability-Adjusted Life Years)	Both	2015	10.44992099	10.28201223	10.61782976
DALYs (Disability-Adjusted Life Years)	Both	2016	10.50665689	10.33927554	10.67403824
DALYs (Disability-Adjusted Life Years)	Both	2017	10.43797315	10.27213395	10.60381235
DALYs (Disability-Adjusted Life Years)	Both	2018	10.45029407	10.28563681	10.61495134
DALYs (Disability-Adjusted Life Years)	Both	2019	10.42818357	10.26471728	10.59164987
DALYs (Disability-Adjusted Life Years)	Both	2020	10.23812137	10.07688632	10.39935643
DALYs (Disability-Adjusted Life Years)	Both	2021	10.24236164	10.08156689	10.40315639
DALYs (Disability-Adjusted Life Years)	Both	2022	10.11711959	9.589764447	10.64447473
DALYs (Disability-Adjusted Life Years)	Both	2023	10.05509017	9.450731414	10.65944893
DALYs (Disability-Adjusted Life Years)	Both	2024	9.990597374	9.269283494	10.71191125
DALYs (Disability-Adjusted Life Years)	Both	2025	9.92678579	9.053860742	10.79971084
DALYs (Disability-Adjusted Life Years)	Both	2026	9.869681785	8.815400042	10.92396353
DALYs (Disability-Adjusted Life Years)	Both	2027	9.821129192	8.559462945	11.08279544
DALYs (Disability-Adjusted Life Years)	Both	2028	9.787385552	8.296075668	11.27869544
DALYs (Disability-Adjusted Life Years)	Both	2029	9.765854114	8.025142407	11.50656582
DALYs (Disability-Adjusted Life Years)	Both	2030	9.75433844	7.745561354	11.76311553
DALYs (Disability-Adjusted Life Years)	Both	2031	9.752333381	7.456537534	12.04812923

DALYs (Disability-Adjusted Life Years)	Both	2032	9.756450157	7.154659013	12.3582413
DALYs (Disability-Adjusted Life Years)	Both	2033	9.769403703	6.842714587	12.69609282
DALYs (Disability-Adjusted Life Years)	Both	2034	9.786111756	6.516976457	13.05524705
DALYs (Disability-Adjusted Life Years)	Both	2035	9.802805878	6.174530039	13.43108172
