# Supplementary material for: Trends in the global burden of aortic valve calcification disease in the working-age population from 1992 to 2021
Source: Front Cardiovasc Med. 2025 Aug 12;12:1544273. doi: 10.3389/fcvm.2025.1544273 (PMC12379075; doi:10.3389/fcvm.2025.1544273)
Supplement: Supplementary file 3 [file Datasheet3.zip › Supplementary Table 11.PDF]

Supplementary

Table S11. Projected Global Burden of Aortic Valve Calcification Disease in Working Age Range (number)

Measure	Sex	Year	Predict value	Predict low	Predict up
Deaths	Male	1992	6717.174585	6176.323869	7258.025301
Deaths	Male	1993	6732.671534	6200.374799	7264.968269
Deaths	Male	1994	6712.747022	6183.6496	7241.844444
Deaths	Male	1995	6657.571602	6131.031105	7184.112098
Deaths	Male	1996	6606.342587	6082.077276	7130.607898
Deaths	Male	1997	6561.574495	6039.331233	7083.817758
Deaths	Male	1998	6525.389686	6004.973963	7045.805408
Deaths	Male	1999	6435.202982	5918.612953	6951.793011
Deaths	Male	2000	6381.943939	5867.270593	6896.617285
Deaths	Male	2001	6383.228471	5868.409237	6898.047704
Deaths	Male	2002	6410.728017	5894.814293	6926.641741
Deaths	Male	2003	6478.766928	5960.183872	6997.349983
Deaths	Male	2004	6556.960069	6035.015014	7078.905124
Deaths	Male	2005	6652.997939	6126.949454	7179.046424
Deaths	Male	2006	6767.006622	6236.286755	7297.726489
Deaths	Male	2007	6929.698216	6392.668334	7466.728099
Deaths	Male	2008	7085.178215	6542.334037	7628.022393
Deaths	Male	2009	7258.814652	6709.802136	7807.827167
Deaths	Male	2010	7423.264231	6868.259405	7978.269056
Deaths	Male	2011	7620.6732	7058.551834	8182.794565

Deaths	Male	2012	7779.935596	7211.865982	8348.005211
Deaths	Male	2013	7969.889409	7394.875444	8544.903373
Deaths	Male	2014	8150.451596	7568.822461	8732.08073
Deaths	Male	2015	8351.010294	7762.038041	8939.982546
Deaths	Male	2016	8513.800947	7918.358922	9109.242973
Deaths	Male	2017	8634.939298	8034.598906	9235.279691
Deaths	Male	2018	8786.295782	8180.228753	9392.362811
Deaths	Male	2019	8901.05376	8290.316022	9511.791498
Deaths	Male	2020	8928.823555	8314.696461	9542.95065
Deaths	Male	2021	8997.377673	8372.513684	9622.241661
Deaths	Male	2022	9176.994329	8445.573468	9908.41519
Deaths	Male	2023	9262.086572	8447.034071	10077.13907
Deaths	Male	2024	9354.528539	8416.588999	10292.46808
Deaths	Male	2025	9447.936868	8349.232692	10546.64104
Deaths	Male	2026	9538.977654	8244.300689	10833.65462
Deaths	Male	2027	9623.736596	8101.341259	11146.13193
Deaths	Male	2028	9702.519404	7925.019304	11480.0195
Deaths	Male	2029	9780.562034	7722.341049	11838.78302
Deaths	Male	2030	9862.585762	7498.067672	12227.10385
Deaths	Male	2031	9952.434696	7254.881257	12649.98814
Deaths	Male	2032	10052.16007	6993.392024	13110.92812
Deaths	Male	2033	10156.44754	6709.95481	13602.94027
Deaths	Male	2034	10260.3753	6400.827392	14119.9232
Deaths	Male	2035	10367.00423	6067.171852	14666.83661

Deaths	Female	1992	3268.743132	2903.63969	3633.846574
Deaths	Female	1993	3278.072736	2918.466511	3637.678961
Deaths	Female	1994	3278.615975	2920.791986	3636.439964
Deaths	Female	1995	3276.641063	2919.225434	3634.056692
Deaths	Female	1996	3265.633164	2908.702397	3622.563931
Deaths	Female	1997	3262.633257	2905.749586	3619.516929
Deaths	Female	1998	3255.1293	2898.533394	3611.725206
Deaths	Female	1999	3231.38033	2875.824526	3586.936134
Deaths	Female	2000	3217.535432	2862.361179	3572.709686
Deaths	Female	2001	3224.590017	2868.777513	3580.40252
Deaths	Female	2002	3242.14261	2885.147687	3599.137533
Deaths	Female	2003	3262.791846	2904.384766	3621.198926
Deaths	Female	2004	3298.869595	2938.304698	3659.434493
Deaths	Female	2005	3340.158369	2977.072895	3703.243842
Deaths	Female	2006	3397.801587	3031.473214	3764.12996
Deaths	Female	2007	3465.85438	3095.963398	3835.745361
Deaths	Female	2008	3554.969147	3180.468533	3929.46976
Deaths	Female	2009	3646.879375	3267.681702	4026.077048
Deaths	Female	2010	3745.831097	3361.566619	4130.095575
Deaths	Female	2011	3871.769464	3481.402855	4262.136073
Deaths	Female	2012	3990.668663	3594.343806	4386.993521
Deaths	Female	2013	4103.892649	3701.822369	4505.96293
Deaths	Female	2014	4219.743446	3811.829004	4627.657887
Deaths	Female	2015	4345.227381	3931.185886	4759.268875

Deaths	Female	2016	4439.868269	4020.972479	4858.764059
Deaths	Female	2017	4519.64034	4096.699922	4942.580758
Deaths	Female	2018	4603.800815	4176.751853	5030.849777
Deaths	Female	2019	4670.990407	4240.157173	5101.823641
Deaths	Female	2020	4703.036317	4268.485522	5137.587111
Deaths	Female	2021	4757.138083	4314.021126	5200.25504
Deaths	Female	2022	4863.534825	4362.63006	5364.439589
Deaths	Female	2023	4921.609634	4374.990349	5468.22892
Deaths	Female	2024	4983.755549	4371.365757	5596.145342
Deaths	Female	2025	5045.719694	4347.566674	5743.872713
Deaths	Female	2026	5104.477788	4301.395907	5907.55967
Deaths	Female	2027	5157.146151	4231.510023	6082.782279
Deaths	Female	2028	5203.397769	4139.837563	6266.957976
Deaths	Female	2029	5247.623535	4031.564243	6463.682826
Deaths	Female	2030	5294.714448	3911.217682	6678.211214
Deaths	Female	2031	5348.508834	3781.501253	6915.516415
Deaths	Female	2032	5411.240147	3643.249409	7179.230885
Deaths	Female	2033	5479.356767	3493.553168	7465.160367
Deaths	Female	2034	5549.250544	3329.370104	7769.130984
Deaths	Female	2035	5622.053372	3150.66442	8093.442325
Deaths	Both	1992	9985.917718	9079.96356	10891.87188
Deaths	Both	1993	10010.74427	9118.84131	10902.64723
Deaths	Both	1994	9991.362997	9104.441586	10878.28441
Deaths	Both	1995	9934.212665	9050.256539	10818.16879

Deaths	Both	1996	9871.975751	8990.779673	10753.17183
Deaths	Both	1997	9824.207753	8945.080818	10703.33469
Deaths	Both	1998	9780.518986	8903.507358	10657.53061
Deaths	Both	1999	9666.583312	8794.437479	10538.72914
Deaths	Both	2000	9599.479371	8729.631772	10469.32697
Deaths	Both	2001	9607.818488	8737.186751	10478.45022
Deaths	Both	2002	9652.870627	8779.96198	10525.77927
Deaths	Both	2003	9741.558773	8864.568638	10618.54891
Deaths	Both	2004	9855.829664	8973.319711	10738.33962
Deaths	Both	2005	9993.156308	9104.022349	10882.29027
Deaths	Both	2006	10164.80821	9267.759969	11061.85645
Deaths	Both	2007	10395.5526	9488.631732	11302.47346
Deaths	Both	2008	10640.14736	9722.80257	11557.49215
Deaths	Both	2009	10905.69403	9977.483839	11833.90421
Deaths	Both	2010	11169.09533	10229.82602	12108.36463
Deaths	Both	2011	11492.44266	10539.95469	12444.93064
Deaths	Both	2012	11770.60426	10806.20979	12734.99873
Deaths	Both	2013	12073.78206	11096.69781	13050.8663
Deaths	Both	2014	12370.19504	11380.65147	13359.73862
Deaths	Both	2015	12696.23767	11693.22393	13699.25142
Deaths	Both	2016	12953.66922	11939.3314	13968.00703
Deaths	Both	2017	13154.57964	12131.29883	14177.86045
Deaths	Both	2018	13390.0966	12356.98061	14423.21259
Deaths	Both	2019	13572.04417	12530.4732	14613.61514

Deaths	Both	2020	13631.85987	12583.18198	14680.53776
Deaths	Both	2021	13754.51576	12686.53481	14822.4967
Deaths	Both	2022	14040.52915	12808.20353	15272.85478
Deaths	Both	2023	14183.69621	12822.02442	15545.36799
Deaths	Both	2024	14338.28409	12787.95476	15888.61342
Deaths	Both	2025	14493.65656	12696.79937	16290.51376
Deaths	Both	2026	14643.45544	12545.6966	16741.21429
Deaths	Both	2027	14780.88275	12332.85128	17228.91421
Deaths	Both	2028	14905.91717	12064.85687	17746.97748
Deaths	Both	2029	15028.18557	11753.90529	18302.46584
Deaths	Both	2030	15157.30021	11409.28535	18905.31507
Deaths	Both	2031	15300.94353	11036.38251	19565.50455
Deaths	Both	2032	15463.40022	10636.64143	20290.159
Deaths	Both	2033	15635.80431	10203.50798	21068.10064
Deaths	Both	2034	15809.62584	9730.197495	21889.05418
Deaths	Both	2035	15989.05761	9217.836272	22760.27894
Incidence	Male	1992	106985.3297	104772.9425	109197.717
Incidence	Male	1993	109134.2595	106918.3452	111350.1738
Incidence	Male	1994	111205.2973	108967.693	113442.9017
Incidence	Male	1995	113183.4369	110923.9859	115442.8878
Incidence	Male	1996	115246.5753	112967.027	117526.1237
Incidence	Male	1997	117718.7959	115418.6142	120018.9776
Incidence	Male	1998	120379.0465	118056.1212	122701.9717
Incidence	Male	1999	123234.5253	120885.9808	125583.0698

Incidence	Male	2000	126194.8916	123818.3689	128571.4142
Incidence	Male	2001	129778.3374	127367.6699	132189.0048
Incidence	Male	2002	134281.9824	131829.5411	136734.4238
Incidence	Male	2003	139208.3169	136709.9858	141706.648
Incidence	Male	2004	144293.5414	141748.7051	146838.3778
Incidence	Male	2005	148968.5351	146381.218	151555.8521
Incidence	Male	2006	153992.5688	151361.0562	156624.0813
Incidence	Male	2007	160910.8677	158223.9644	163597.771
Incidence	Male	2008	168360.0732	165615.667	171104.4794
Incidence	Male	2009	175940.8232	173140.8096	178740.8368
Incidence	Male	2010	183411.098	180558.6282	186263.5677
Incidence	Male	2011	190029.1797	187131.5337	192926.8257
Incidence	Male	2012	194691.0875	191759.2885	197622.8865
Incidence	Male	2013	199172.3434	196208.7565	202135.9302
Incidence	Male	2014	203219.074	200227.3628	206210.7851
Incidence	Male	2015	207434.2388	204414.5361	210453.9415
Incidence	Male	2016	211128.5845	208083.7471	214173.4219
Incidence	Male	2017	214267.9389	211200.334	217335.5439
Incidence	Male	2018	217026.7963	213939.6728	220113.9197
Incidence	Male	2019	219396.6854	216292.9177	222500.4532
Incidence	Male	2020	220260.7279	217148.0803	223373.3755
Incidence	Male	2021	223418.8706	220269.3955	226568.3457
Incidence	Male	2022	228873.0924	221811.3555	235934.8293
Incidence	Male	2023	231195.7046	222194.7946	240196.6147

Incidence	Male	2024	233607.1201	221814.8537	245399.3866
Incidence	Male	2025	236040.8603	220756.2152	251325.5053
Incidence	Male	2026	238536.5137	219121.5176	257951.5099
Incidence	Male	2027	240871.2838	216706.8793	265035.6882
Incidence	Male	2028	243030.422	213644.4254	272416.4186
Incidence	Male	2029	245109.0782	210082.9801	280135.1762
Incidence	Male	2030	247190.5129	206111.6063	288269.4195
Incidence	Male	2031	249394.6969	201800.1178	296989.2759
Incidence	Male	2032	251578.3715	196973.5902	306183.1529
Incidence	Male	2033	253685.7859	191654.7731	315716.7988
Incidence	Male	2034	255609.7704	185795.1694	325424.3714
Incidence	Male	2035	257497.8745	179510.7709	335484.978
Incidence	Female	1992	58875.17901	57299.89383	60450.46419
Incidence	Female	1993	59974.57591	58398.65153	61550.50028
Incidence	Female	1994	61022.22651	59432.30911	62612.14392
Incidence	Female	1995	62036.0144	60431.41948	63640.60931
Incidence	Female	1996	63047.0903	61430.12366	64664.05694
Incidence	Female	1997	64241.48936	62613.15248	65869.82624
Incidence	Female	1998	65464.81789	63824.6391	67104.99667
Incidence	Female	1999	66844.91142	65189.60712	68500.21573
Incidence	Female	2000	68345.82502	66672.09085	70019.55919
Incidence	Female	2001	70271.16109	68573.16164	71969.16055
Incidence	Female	2002	72729.30197	71000.57606	74458.02788
Incidence	Female	2003	75420.72062	73657.80567	77183.63556

Incidence	Female	2004	78166.96533	76369.3186	79964.61207
Incidence	Female	2005	80511.19023	78683.25136	82339.12909
Incidence	Female	2006	83004.93184	81146.05769	84863.80599
Incidence	Female	2007	86759.08767	84860.34877	88657.82657
Incidence	Female	2008	90869.85937	88929.38361	92810.33512
Incidence	Female	2009	95142.35579	93160.8123	97123.89928
Incidence	Female	2010	99472.1344	97450.41435	101493.8545
Incidence	Female	2011	103346.9554	101290.356	105403.5547
Incidence	Female	2012	105825.5985	103744.4094	107906.7875
Incidence	Female	2013	108172.939	106068.9016	110276.9765
Incidence	Female	2014	110263.9015	108139.4669	112388.336
Incidence	Female	2015	112582.8699	110437.0696	114728.6702
Incidence	Female	2016	114767.3836	112600.7688	116933.9985
Incidence	Female	2017	116791.6219	114604.6322	118978.6115
Incidence	Female	2018	118631.9566	116426.6226	120837.2907
Incidence	Female	2019	120144.6683	117924.6209	122364.7157
Incidence	Female	2020	120464.533	118239.5953	122689.4706
Incidence	Female	2021	122540.1212	120285.8691	124794.3733
Incidence	Female	2022	125917.899	120987.9623	130847.8356
Incidence	Female	2023	127313.1016	121065.2934	133560.9098
Incidence	Female	2024	128789.6404	120632.2665	136947.0143
Incidence	Female	2025	130303.3183	119742.2708	140864.3658
Incidence	Female	2026	131880.1589	118471.2083	145289.1095
Incidence	Female	2027	133413.629	116740.6051	150086.6528

Incidence	Female	2028	134821.8873	114549.9202	155093.8543
Incidence	Female	2029	136152.0418	111978.032	160326.0517
Incidence	Female	2030	137469.6352	109091.1708	165848.0995
Incidence	Female	2031	138866.1481	105948.0117	171784.2845
Incidence	Female	2032	140316.0035	102500.2795	178131.7275
Incidence	Female	2033	141771.8479	98732.74115	184810.9546
Incidence	Female	2034	143155.8067	94599.2533	191712.3601
Incidence	Female	2035	144548.4162	90150.80557	198946.0269
Incidence	Both	1992	165860.5087	162072.8363	169648.1811
Incidence	Both	1993	169108.8354	165316.9967	172900.6741
Incidence	Both	1994	172227.5239	168400.0021	176055.0456
Incidence	Both	1995	175219.4513	171355.4054	179083.4972
Incidence	Both	1996	178293.6656	174397.1507	182190.1806
Incidence	Both	1997	181960.2853	178031.7667	185888.8039
Incidence	Both	1998	185843.8644	181880.7603	189806.9684
Incidence	Both	1999	190079.4367	186075.5879	194083.2855
Incidence	Both	2000	194540.7166	190490.4598	198590.9734
Incidence	Both	2001	200049.4985	195940.8316	204158.1654
Incidence	Both	2002	207011.2844	202830.1171	211192.4517
Incidence	Both	2003	214629.0375	210367.7915	218890.2836
Incidence	Both	2004	222460.5068	218118.0237	226802.9899
Incidence	Both	2005	229479.7253	225064.4694	233894.9812
Incidence	Both	2006	236997.5006	232507.1139	241487.8873
Incidence	Both	2007	247669.9554	243084.3132	252255.5976

Incidence	Both	2008	259229.9326	254545.0507	263914.8145
Incidence	Both	2009	271083.179	266301.6219	275864.7361
Incidence	Both	2010	282883.2324	278009.0426	287757.4222
Incidence	Both	2011	293376.1351	288421.8898	298330.3804
Incidence	Both	2012	300516.686	295503.6979	305529.674
Incidence	Both	2013	307345.2824	302277.6581	312412.9067
Incidence	Both	2014	313482.9754	308366.8297	318599.1212
Incidence	Both	2015	320017.1087	314851.6057	325182.6117
Incidence	Both	2016	325895.9681	320684.5159	331107.4204
Incidence	Both	2017	331059.5608	325804.9662	336314.1554
Incidence	Both	2018	335658.7529	330366.2954	340951.2104
Incidence	Both	2019	339541.3537	334217.5386	344865.1689
Incidence	Both	2020	340725.2609	335387.6756	346062.8462
Incidence	Both	2021	345958.9918	340555.2646	351362.719
Incidence	Both	2022	354790.9914	342799.3179	366782.6649
Incidence	Both	2023	358508.8062	343260.0879	373757.5245
Incidence	Both	2024	362396.7606	342447.1202	382346.4009
Incidence	Both	2025	366344.1785	340498.486	392189.8711
Incidence	Both	2026	370416.6726	337592.7259	403240.6194
Incidence	Both	2027	374284.9127	333447.4844	415122.341
Incidence	Both	2028	377852.3093	328194.3456	427510.273
Incidence	Both	2029	381261.12	322061.0121	440461.2279
Incidence	Both	2030	384660.1481	315202.7772	454117.519
Incidence	Both	2031	388260.845	307748.1295	468773.5604

Incidence	Both	2032	391894.375	299473.8697	484314.8803
Incidence	Both	2033	395457.6338	290387.5142	500527.7535
Incidence	Both	2034	398765.5771	280394.4227	517136.7315
Incidence	Both	2035	402046.2907	269661.5765	534431.0049
Prevalence	Male	1992	863964.2767	857509.3534	870419.1999
Prevalence	Male	1993	883443.2626	876922.0699	889964.4553
Prevalence	Male	1994	900650.0809	894057.0455	907243.1163
Prevalence	Male	1995	916423.501	909766.6141	923080.388
Prevalence	Male	1996	930111.4248	923405.6213	936817.2282
Prevalence	Male	1997	944440.0851	937693.6289	951186.5414
Prevalence	Male	1998	958885.1232	952099.4194	965670.8271
Prevalence	Male	1999	975309.23	968475.792	982142.6679
Prevalence	Male	2000	994761.6838	987864.2377	1001659.13
Prevalence	Male	2001	1021206.609	1014219.067	1028194.151
Prevalence	Male	2002	1055959.61	1048856.002	1063063.217
Prevalence	Male	2003	1093631.955	1086403.083	1100860.828
Prevalence	Male	2004	1132377.066	1125022.145	1139731.987
Prevalence	Male	2005	1167107.679	1159638.875	1174576.483
Prevalence	Male	2006	1204352.117	1196765.43	1211938.803
Prevalence	Male	2007	1257444.872	1249707.691	1265182.054
Prevalence	Male	2008	1314921.963	1307028.273	1322815.653
Prevalence	Male	2009	1373959.391	1365913.52	1382005.262
Prevalence	Male	2010	1432501.155	1424314.237	1440688.074
Prevalence	Male	2011	1484018.317	1475710.973	1492325.661

Prevalence	Male	2012	1519013.068	1510617.117	1527409.02
Prevalence	Male	2013	1552850.649	1544371.491	1561329.807
Prevalence	Male	2014	1583813.462	1575259.234	1592367.689
Prevalence	Male	2015	1617446.531	1608815.084	1626077.979
Prevalence	Male	2016	1648440.851	1639737.143	1657144.558
Prevalence	Male	2017	1675604.297	1666834.299	1684374.295
Prevalence	Male	2018	1700053.59	1691226.241	1708880.939
Prevalence	Male	2019	1721339.462	1712464.767	1730214.157
Prevalence	Male	2020	1729254.287	1720364.004	1738144.569
Prevalence	Male	2021	1755302.187	1746340.585	1764263.788
Prevalence	Male	2022	1789595.401	1745075.021	1834115.78
Prevalence	Male	2023	1806382.159	1743548.777	1869215.54
Prevalence	Male	2024	1824045.409	1735105.618	1912985.199
Prevalence	Male	2025	1842086.942	1721148.09	1963025.794
Prevalence	Male	2026	1860897.991	1702817.072	2018978.909
Prevalence	Male	2027	1878425.147	1678159.304	2078690.99
Prevalence	Male	2028	1894407.571	1647903.316	2140911.826
Prevalence	Male	2029	1909568.968	1613141.553	2205996.383
Prevalence	Male	2030	1924844.033	1574884.026	2274804.04
Prevalence	Male	2031	1942001.169	1534424.752	2349577.586
Prevalence	Male	2032	1959275.759	1489765.128	2428786.391
Prevalence	Male	2033	1974933.783	1440015.153	2509852.412
Prevalence	Male	2034	1988160.615	1384897.909	2591423.322
Prevalence	Male	2035	2000709.436	1325851.998	2675566.874

Prevalence	Female	1992	442891.1421	438380.0647	447402.2195
Prevalence	Female	1993	452504.3662	447945.8645	457062.8679
Prevalence	Female	1994	460719.24	456109.6915	465328.7884
Prevalence	Female	1995	468294.9581	463640.6942	472949.2221
Prevalence	Female	1996	473553.0759	468873.3749	478232.7769
Prevalence	Female	1997	477484.4114	472796.7875	482172.0353
Prevalence	Female	1998	480302.8725	475613.7785	484991.9664
Prevalence	Female	1999	484771.3792	480069.8116	489472.9467
Prevalence	Female	2000	492405.5365	487669.3729	497141.7001
Prevalence	Female	2001	505077.707	500278.7131	509876.7008
Prevalence	Female	2002	522387.82	517503.9826	527271.6575
Prevalence	Female	2003	541442.7478	536465.8783	546419.6173
Prevalence	Female	2004	561142.6934	556071.8555	566213.5312
Prevalence	Female	2005	578553.7739	573398.4822	583709.0656
Prevalence	Female	2006	597307.7855	592066.2238	602549.3472
Prevalence	Female	2007	624289.903	618940.1377	629639.6682
Prevalence	Female	2008	653942.6248	648479.1977	659406.052
Prevalence	Female	2009	685054.0032	679477.8271	690630.1792
Prevalence	Female	2010	716508.7906	710825.0277	722192.5534
Prevalence	Female	2011	745016.4171	739237.8413	750794.9929
Prevalence	Female	2012	764379.3515	758530.4995	770228.2034
Prevalence	Female	2013	783312.803	777396.8905	789228.7156
Prevalence	Female	2014	800741.3744	794764.1678	806718.581
Prevalence	Female	2015	820062.7575	814021.4737	826104.0412

Prevalence	Female	2016	839131.53	833025.6276	845237.4325
Prevalence	Female	2017	857700.9079	851530.1933	863871.6224
Prevalence	Female	2018	874426.2659	868198.3549	880654.177
Prevalence	Female	2019	887222.757	880952.9924	893492.5215
Prevalence	Female	2020	887734.7118	881465.8645	894003.559
Prevalence	Female	2021	902870.9153	896546.1388	909195.6919
Prevalence	Female	2022	923231.3408	892882.6677	953580.0138
Prevalence	Female	2023	931608.1477	887244.3106	975971.9847
Prevalence	Female	2024	940519.1981	876314.3596	1004724.037
Prevalence	Female	2025	949860.025	861407.0609	1038312.989
Prevalence	Female	2026	959822.8327	843282.9164	1076362.749
Prevalence	Female	2027	969397.5842	821141.5087	1117653.66
Prevalence	Female	2028	978127.8593	795046.3202	1161209.398
Prevalence	Female	2029	986500.3569	765651.3372	1207349.376
Prevalence	Female	2030	995210.0811	733591.1065	1256829.056
Prevalence	Female	2031	1005160.15	699398.399	1310921.902
Prevalence	Female	2032	1015807.106	662316.7654	1369297.446
Prevalence	Female	2033	1026545.59	621952.2281	1431138.952
Prevalence	Female	2034	1036821.585	577952.944	1495690.226
Prevalence	Female	2035	1047468.834	530722.0125	1564215.656
Prevalence	Both	1992	1306855.419	1295889.418	1317821.419
Prevalence	Both	1993	1335947.629	1324867.934	1347027.323
Prevalence	Both	1994	1361369.321	1350166.737	1372571.905
Prevalence	Both	1995	1384718.459	1373407.308	1396029.61

Prevalence	Both	1996	1403664.501	1392278.996	1415050.005
Prevalence	Both	1997	1421924.497	1410490.416	1433358.577
Prevalence	Both	1998	1439187.996	1427713.198	1450662.793
Prevalence	Both	1999	1460080.609	1448545.604	1471615.615
Prevalence	Both	2000	1487167.22	1475533.611	1498800.83
Prevalence	Both	2001	1526284.316	1514497.78	1538070.852
Prevalence	Both	2002	1578347.43	1566359.985	1590334.874
Prevalence	Both	2003	1635074.703	1622868.961	1647280.445
Prevalence	Both	2004	1693519.76	1681094.001	1705945.519
Prevalence	Both	2005	1745661.453	1733037.358	1758285.549
Prevalence	Both	2006	1801659.902	1788831.654	1814488.15
Prevalence	Both	2007	1881734.775	1868647.829	1894821.722
Prevalence	Both	2008	1968864.588	1955507.471	1982221.705
Prevalence	Both	2009	2059013.394	2045391.347	2072635.441
Prevalence	Both	2010	2149009.946	2135139.265	2162880.627
Prevalence	Both	2011	2229034.734	2214948.814	2243120.653
Prevalence	Both	2012	2283392.42	2269147.616	2297637.223
Prevalence	Both	2013	2336163.452	2321768.382	2350558.523
Prevalence	Both	2014	2384554.836	2370023.402	2399086.27
Prevalence	Both	2015	2437509.289	2422836.557	2452182.021
Prevalence	Both	2016	2487572.381	2472762.771	2502381.991
Prevalence	Both	2017	2533305.205	2518364.492	2548245.918
Prevalence	Both	2018	2574479.856	2559424.596	2589535.116
Prevalence	Both	2019	2608562.219	2593417.76	2623706.679

Prevalence	Both	2020	2616988.999	2601829.869	2632148.128
Prevalence	Both	2021	2658173.102	2642886.724	2673459.48
Prevalence	Both	2022	2712826.741	2637957.689	2787695.794
Prevalence	Both	2023	2737990.306	2630793.088	2845187.525
Prevalence	Both	2024	2764564.607	2611419.978	2917709.236
Prevalence	Both	2025	2791946.967	2582555.151	3001338.783
Prevalence	Both	2026	2820720.823	2546099.989	3095341.658
Prevalence	Both	2027	2847822.731	2499300.813	3196344.65
Prevalence	Both	2028	2872535.43	2442949.636	3302121.224
Prevalence	Both	2029	2896069.325	2378792.89	3413345.76
Prevalence	Both	2030	2920054.114	2308475.132	3531633.096
Prevalence	Both	2031	2947161.319	2233823.151	3660499.488
Prevalence	Both	2032	2975082.865	2152081.893	3798083.837
Prevalence	Both	2033	3001479.373	2061967.381	3940991.365
Prevalence	Both	2034	3024982.2	1962850.853	4087113.548
Prevalence	Both	2035	3048178.27	1856574.01	4239782.53
DALYs (Disability-Adjusted Life Years)	Male	1992	268743.969	264446.9561	273040.982
DALYs (Disability-Adjusted Life Years)	Male	1993	273121.964	268790.5886	277453.3393
DALYs (Disability-Adjusted Life Years)	Male	1994	273168.6966	268835.7606	277501.6325
DALYs (Disability-Adjusted Life Years)	Male	1995	270908.7887	266590.6711	275226.9063
DALYs (Disability-Adjusted Life Years)	Male	1996	268644.8403	264343.9819	272945.6986
DALYs (Disability-Adjusted Life Years)	Male	1997	268606.2893	264302.3784	272910.2003
DALYs (Disability-Adjusted Life Years)	Male	1998	269261.5341	264950.7973	273572.271
DALYs (Disability-Adjusted Life Years)	Male	1999	262523.4262	258261.9621	266784.8904

DALYs (Disability-Adjusted Life Years)	Male	2000	260486.0903	256237.2358	264734.9449
DALYs (Disability-Adjusted Life Years)	Male	2001	262133.5658	257871.0101	266396.1215
DALYs (Disability-Adjusted Life Years)	Male	2002	263275.4885	259005.1152	267545.8618
DALYs (Disability-Adjusted Life Years)	Male	2003	266797.1626	262500.2906	271094.0345
DALYs (Disability-Adjusted Life Years)	Male	2004	270702.8254	266369.549	275036.1018
DALYs (Disability-Adjusted Life Years)	Male	2005	275185.0561	270815.4124	279554.6998
DALYs (Disability-Adjusted Life Years)	Male	2006	278829.4115	274431.4075	283227.4155
DALYs (Disability-Adjusted Life Years)	Male	2007	286363.6125	281911.7892	290815.4357
DALYs (Disability-Adjusted Life Years)	Male	2008	291483.0048	286997.9186	295968.091
DALYs (Disability-Adjusted Life Years)	Male	2009	296871.9909	292353.6814	301390.3003
DALYs (Disability-Adjusted Life Years)	Male	2010	301526.2594	296979.5739	306072.945
DALYs (Disability-Adjusted Life Years)	Male	2011	308374.6246	303787.7183	312961.5308
DALYs (Disability-Adjusted Life Years)	Male	2012	313090.4595	308475.5175	317705.4016
DALYs (Disability-Adjusted Life Years)	Male	2013	320938.831	316269.695	325607.967
DALYs (Disability-Adjusted Life Years)	Male	2014	327847.1278	323132.075	332562.1805
DALYs (Disability-Adjusted Life Years)	Male	2015	335268.1915	330507.0631	340029.32
DALYs (Disability-Adjusted Life Years)	Male	2016	342557.7798	337745.6491	347369.9104
DALYs (Disability-Adjusted Life Years)	Male	2017	345144.6387	340317.1826	349972.0948
DALYs (Disability-Adjusted Life Years)	Male	2018	351812.9499	346945.4591	356680.4407
DALYs (Disability-Adjusted Life Years)	Male	2019	356578.6196	351681.8356	361475.4035
DALYs (Disability-Adjusted Life Years)	Male	2020	354300.2501	349420.0992	359180.4011
DALYs (Disability-Adjusted Life Years)	Male	2021	358786.5715	353868.0929	363705.0501
DALYs (Disability-Adjusted Life Years)	Male	2022	363818.7266	345436.8478	382200.6055
DALYs (Disability-Adjusted Life Years)	Male	2023	367013.277	345402.6988	388623.8552

DALYs (Disability-Adjusted Life Years)	Male	2024	370371.9978	343855.3144	396888.6811
DALYs (Disability-Adjusted Life Years)	Male	2025	373664.2258	340746.2973	406582.1543
DALYs (Disability-Adjusted Life Years)	Male	2026	376863.1443	336231.5015	417494.7872
DALYs (Disability-Adjusted Life Years)	Male	2027	379846.0813	330339.3879	429352.7747
DALYs (Disability-Adjusted Life Years)	Male	2028	382816.8583	323424.2666	442209.45
DALYs (Disability-Adjusted Life Years)	Male	2029	385834.4445	315628.5014	456040.3876
DALYs (Disability-Adjusted Life Years)	Male	2030	388944.2597	307024.1295	470864.3898
DALYs (Disability-Adjusted Life Years)	Male	2031	392243.0626	297674.5059	486811.6194
DALYs (Disability-Adjusted Life Years)	Male	2032	395723.8287	287533.7107	503913.9467
DALYs (Disability-Adjusted Life Years)	Male	2033	399421.3456	276654.9702	522187.7209
DALYs (Disability-Adjusted Life Years)	Male	2034	403093.6085	264858.4391	541328.778
DALYs (Disability-Adjusted Life Years)	Male	2035	406691.4449	252095.7836	561287.1062
DALYs (Disability-Adjusted Life Years)	Female	1992	128959.9923	126037.6094	131882.3752
DALYs (Disability-Adjusted Life Years)	Female	1993	130890.054	127946.3854	133833.7226
DALYs (Disability-Adjusted Life Years)	Female	1994	131692.4184	128736.3985	134648.4383
DALYs (Disability-Adjusted Life Years)	Female	1995	131973.0876	129013.4565	134932.7187
DALYs (Disability-Adjusted Life Years)	Female	1996	131192.9706	128239.1368	134146.8044
DALYs (Disability-Adjusted Life Years)	Female	1997	132129.2373	129160.3555	135098.1192
DALYs (Disability-Adjusted Life Years)	Female	1998	132838.5015	129857.4163	135819.5868
DALYs (Disability-Adjusted Life Years)	Female	1999	130801.9628	127837.5548	133766.3708
DALYs (Disability-Adjusted Life Years)	Female	2000	130266.77	127302.1864	133231.3537
DALYs (Disability-Adjusted Life Years)	Female	2001	131252.4711	128276.0985	134228.8436
DALYs (Disability-Adjusted Life Years)	Female	2002	132869.6898	129872.5926	135866.7871
DALYs (Disability-Adjusted Life Years)	Female	2003	134018.632	131005.0231	137032.241

DALYs (Disability-Adjusted Life Years)	Female	2004	135350.7241	132321.9606	138379.4876
DALYs (Disability-Adjusted Life Years)	Female	2005	137133.0494	134084.841	140181.2579
DALYs (Disability-Adjusted Life Years)	Female	2006	139745.5716	136669.7722	142821.3709
DALYs (Disability-Adjusted Life Years)	Female	2007	141227.1452	138139.4264	144314.8639
DALYs (Disability-Adjusted Life Years)	Female	2008	145301.9401	142172.9849	148430.8952
DALYs (Disability-Adjusted Life Years)	Female	2009	148121.3611	144964.884	151277.8381
DALYs (Disability-Adjusted Life Years)	Female	2010	150866.8489	147684.4168	154049.2809
DALYs (Disability-Adjusted Life Years)	Female	2011	155598.6517	152373.5506	158823.7528
DALYs (Disability-Adjusted Life Years)	Female	2012	160765.2458	157490.4675	164040.024
DALYs (Disability-Adjusted Life Years)	Female	2013	164815.3013	161499.9656	168130.637
DALYs (Disability-Adjusted Life Years)	Female	2014	168994.3625	165644.0228	172344.7022
DALYs (Disability-Adjusted Life Years)	Female	2015	173673.9058	170282.9162	177064.8955
DALYs (Disability-Adjusted Life Years)	Female	2016	177714.5801	174282.5036	181146.6566
DALYs (Disability-Adjusted Life Years)	Female	2017	180250.925	176794.3793	183707.4707
DALYs (Disability-Adjusted Life Years)	Female	2018	182819.4002	179347.1568	186291.6437
DALYs (Disability-Adjusted Life Years)	Female	2019	185152.1137	181660.2305	188643.9968
DALYs (Disability-Adjusted Life Years)	Female	2020	185127.7124	181632.1722	188623.2525
DALYs (Disability-Adjusted Life Years)	Female	2021	188300.5053	184767.4769	191833.5337
DALYs (Disability-Adjusted Life Years)	Female	2022	191829.6929	181328.2561	202331.1296
DALYs (Disability-Adjusted Life Years)	Female	2023	194179.1572	182173.863	206184.4514
DALYs (Disability-Adjusted Life Years)	Female	2024	196592.5076	182330.9142	210854.1009
DALYs (Disability-Adjusted Life Years)	Female	2025	198990.3757	181756.6639	216224.0875
DALYs (Disability-Adjusted Life Years)	Female	2026	201333.8046	180466.6462	222200.9629
DALYs (Disability-Adjusted Life Years)	Female	2027	203536.2508	178433.1797	228639.3219

DALYs (Disability-Adjusted Life Years)	Female	2028	205632.959	175777.8023	235488.1156
DALYs (Disability-Adjusted Life Years)	Female	2029	207650.1123	172576.52	242723.7047
DALYs (Disability-Adjusted Life Years)	Female	2030	209713.2434	168957.3555	250469.1314
DALYs (Disability-Adjusted Life Years)	Female	2031	211967.4419	165027.9114	258906.9723
DALYs (Disability-Adjusted Life Years)	Female	2032	214491.2901	160819.1366	268163.4436
DALYs (Disability-Adjusted Life Years)	Female	2033	217234.6004	156300.7857	278168.415
DALYs (Disability-Adjusted Life Years)	Female	2034	219982.3134	151304.1919	288660.4348
DALYs (Disability-Adjusted Life Years)	Female	2035	222714.3829	145804.2349	299624.5309
DALYs (Disability-Adjusted Life Years)	Both	1992	397703.9613	390484.5655	404923.3572
DALYs (Disability-Adjusted Life Years)	Both	1993	404012.018	396736.974	411287.0619
DALYs (Disability-Adjusted Life Years)	Both	1994	404861.115	397572.1591	412150.0708
DALYs (Disability-Adjusted Life Years)	Both	1995	402881.8763	395604.1276	410159.625
DALYs (Disability-Adjusted Life Years)	Both	1996	399837.8108	392583.1187	407092.503
DALYs (Disability-Adjusted Life Years)	Both	1997	400735.5266	393462.7338	408008.3194
DALYs (Disability-Adjusted Life Years)	Both	1998	402100.0357	394808.2136	409391.8578
DALYs (Disability-Adjusted Life Years)	Both	1999	393325.389	386099.517	400551.2611
DALYs (Disability-Adjusted Life Years)	Both	2000	390752.8604	383539.4221	397966.2986
DALYs (Disability-Adjusted Life Years)	Both	2001	393386.0369	386147.1087	400624.9651
DALYs (Disability-Adjusted Life Years)	Both	2002	396145.1784	388877.7078	403412.6489
DALYs (Disability-Adjusted Life Years)	Both	2003	400815.7946	393505.3137	408126.2755
DALYs (Disability-Adjusted Life Years)	Both	2004	406053.5495	398691.5096	413415.5894
DALYs (Disability-Adjusted Life Years)	Both	2005	412318.1055	404900.2534	419735.9576
DALYs (Disability-Adjusted Life Years)	Both	2006	418574.9831	411101.1798	426048.7864
DALYs (Disability-Adjusted Life Years)	Both	2007	427590.7576	420051.2156	435130.2996

DALYs (Disability-Adjusted Life Years)	Both	2008	436784.9449	429170.9035	444398.9862
DALYs (Disability-Adjusted Life Years)	Both	2009	444993.3519	437318.5654	452668.1384
DALYs (Disability-Adjusted Life Years)	Both	2010	452393.1083	444663.9907	460122.2259
DALYs (Disability-Adjusted Life Years)	Both	2011	463973.2763	456161.269	471785.2836
DALYs (Disability-Adjusted Life Years)	Both	2012	473855.7053	465965.985	481745.4256
DALYs (Disability-Adjusted Life Years)	Both	2013	485754.1323	477769.6607	493738.6039
DALYs (Disability-Adjusted Life Years)	Both	2014	496841.4902	488776.0977	504906.8827
DALYs (Disability-Adjusted Life Years)	Both	2015	508942.0974	500789.9793	517094.2154
DALYs (Disability-Adjusted Life Years)	Both	2016	520272.3599	512028.1527	528516.567
DALYs (Disability-Adjusted Life Years)	Both	2017	525395.5637	517111.5619	533679.5654
DALYs (Disability-Adjusted Life Years)	Both	2018	534632.3501	526292.6159	542972.0844
DALYs (Disability-Adjusted Life Years)	Both	2019	541730.7332	533342.0661	550119.4004
DALYs (Disability-Adjusted Life Years)	Both	2020	539427.9625	531052.2714	547803.6536
DALYs (Disability-Adjusted Life Years)	Both	2021	547087.0768	538635.5698	555538.5838
DALYs (Disability-Adjusted Life Years)	Both	2022	555648.4195	526765.1039	584531.735
DALYs (Disability-Adjusted Life Years)	Both	2023	561192.4343	527576.5619	594808.3066
DALYs (Disability-Adjusted Life Years)	Both	2024	566964.5053	526186.2285	607742.7821
DALYs (Disability-Adjusted Life Years)	Both	2025	572654.6015	522502.9612	622806.2418
DALYs (Disability-Adjusted Life Years)	Both	2026	578196.9489	516698.1477	639695.7502
DALYs (Disability-Adjusted Life Years)	Both	2027	583382.3321	508772.5676	657992.0965
DALYs (Disability-Adjusted Life Years)	Both	2028	588449.8173	499202.0689	677697.5657
DALYs (Disability-Adjusted Life Years)	Both	2029	593484.5568	488205.0214	698764.0922
DALYs (Disability-Adjusted Life Years)	Both	2030	598657.5031	475981.485	721333.5212
DALYs (Disability-Adjusted Life Years)	Both	2031	604210.5045	462702.4173	745718.5917

DALYs (Disability-Adjusted Life Years)	Both	2032	610215.1188	448352.8472	772077.3903
DALYs (Disability-Adjusted Life Years)	Both	2033	616655.9459	432955.756	800356.1359
DALYs (Disability-Adjusted Life Years)	Both	2034	623075.9219	416162.631	829989.2128
DALYs (Disability-Adjusted Life Years)	Both	2035	629405.8278	397900.0185	860911.6371
