# Supplementary material for: Trends in the global burden of aortic valve calcification disease in the working-age population from 1992 to 2021
Source: Front Cardiovasc Med. 2025 Aug 12;12:1544273. doi: 10.3389/fcvm.2025.1544273 (PMC12379075; doi:10.3389/fcvm.2025.1544273)
Supplement: Supplementary file 3 [file Datasheet3.zip › Supplementary Table 10.PDF]

Supplementary

Table S10. A birth cohort model of aortic valve calcification in the working-age range from 1992 to 2021

Measure	Sex	Label	Cohort	Rate Ratio	CI Low	CI High	Location
Deaths	Male	1927 to 1936	1932	2.317264517	2.016046647	2.6634874	High SDI
Deaths	Male	1932 to 1941	1937	2.048843997	1.797697853	2.335076341	High SDI
Deaths	Male	1937 to 1946	1942	1.829736107	1.608581657	2.081295784	High SDI
Deaths	Male	1942 to 1951	1947	1.657494524	1.459632055	1.882178517	High SDI
Deaths	Male	1947 to 1956	1952	1.589506887	1.404636064	1.798709437	High SDI
Deaths	Male	1952 to 1961	1957	1.449309826	1.285019951	1.634604171	High SDI
Deaths	Male	1957 to 1966	1962	1.316600103	1.166620996	1.485860306	High SDI
Deaths	Male	1962 to 1971	1967	1.176872791	1.040033812	1.331715903	High SDI
Deaths	Male	1967 to 1976	1972	1.062981687	0.934358626	1.209310896	High SDI
Deaths	Male	1972 to 1981	1977	1	1	1	High SDI
Deaths	Male	1977 to 1986	1982	0.96930172	0.827290284	1.135690631	High SDI
Deaths	Male	1982 to 1991	1987	0.943441762	0.781225104	1.139341725	High SDI
Deaths	Male	1987 to 1996	1992	0.848948179	0.666332497	1.081611679	High SDI
Deaths	Male	1992 to 2001	1997	0.734798463	0.521106292	1.036120248	High SDI
Deaths	Male	1997 to 2006	2002	0.566943421	0.314090607	1.023350703	High SDI
Deaths	Male	1927 to 1936	1932	0.822106967	0.676240034	0.999437821	Low-middle SDI
Deaths	Male	1932 to 1941	1937	0.787906021	0.668398296	0.928781389	Low-middle SDI
Deaths	Male	1937 to 1946	1942	0.823243394	0.705027024	0.961281855	Low-middle SDI
Deaths	Male	1942 to 1951	1947	0.841455079	0.724847001	0.976822209	Low-middle SDI
Deaths	Male	1947 to 1956	1952	0.856102847	0.743973404	0.985132104	Low-middle SDI

Deaths	Male	1952 to 1961	1957	0.897707949	0.787109528	1.023846789	Low-middle SDI
Deaths	Male	1957 to 1966	1962	0.92651521	0.814804244	1.053541929	Low-middle SDI
Deaths	Male	1962 to 1971	1967	0.919325575	0.809808814	1.043653142	Low-middle SDI
Deaths	Male	1967 to 1976	1972	0.941480898	0.829869092	1.068103739	Low-middle SDI
Deaths	Male	1972 to 1981	1977	1	1	1	Low-middle SDI
Deaths	Male	1977 to 1986	1982	1.006618938	0.874669813	1.158473371	Low-middle SDI
Deaths	Male	1982 to 1991	1987	1.027676657	0.875283956	1.206601932	Low-middle SDI
Deaths	Male	1987 to 1996	1992	1.015442135	0.843965847	1.221758834	Low-middle SDI
Deaths	Male	1992 to 2001	1997	1.004815297	0.802841499	1.257600389	Low-middle SDI
Deaths	Male	1997 to 2006	2002	0.989846391	0.724015256	1.353280708	Low-middle SDI
Deaths	Male	1927 to 1936	1932	1.319670684	1.103092715	1.578770933	High-middle SDI
Deaths	Male	1932 to 1941	1937	1.224508583	1.040152901	1.441539286	High-middle SDI
Deaths	Male	1937 to 1946	1942	1.20535823	1.026553038	1.415307743	High-middle SDI
Deaths	Male	1942 to 1951	1947	1.150914278	0.983561986	1.34674143	High-middle SDI
Deaths	Male	1947 to 1956	1952	1.079135505	0.928259301	1.254534629	High-middle SDI
Deaths	Male	1952 to 1961	1957	1.072095967	0.927943867	1.238641476	High-middle SDI
Deaths	Male	1957 to 1966	1962	1.018304875	0.882250461	1.175340635	High-middle SDI
Deaths	Male	1962 to 1971	1967	0.902368102	0.780821902	1.042834723	High-middle SDI
Deaths	Male	1967 to 1976	1972	0.913371222	0.788084053	1.058576159	High-middle SDI
Deaths	Male	1972 to 1981	1977	1	1	1	High-middle SDI
Deaths	Male	1977 to 1986	1982	0.959811118	0.805891402	1.143128442	High-middle SDI
Deaths	Male	1982 to 1991	1987	0.800351752	0.64901741	0.986973413	High-middle SDI
Deaths	Male	1987 to 1996	1992	0.795214312	0.611625911	1.033909438	High-middle SDI
Deaths	Male	1992 to 2001	1997	0.84218471	0.597128112	1.187810574	High-middle SDI

Deaths	Male	1997 to 2006	2002	0.801214952	0.479477006	1.338845014	High-middle SDI
Deaths	Male	1927 to 1936	1932	0.933544987	0.689641695	1.263708747	Low SDI
Deaths	Male	1932 to 1941	1937	0.937644216	0.727663924	1.208218035	Low SDI
Deaths	Male	1937 to 1946	1942	0.936852948	0.735883543	1.192706991	Low SDI
Deaths	Male	1942 to 1951	1947	0.947198604	0.750206979	1.195916888	Low SDI
Deaths	Male	1947 to 1956	1952	0.935210911	0.750453731	1.165454195	Low SDI
Deaths	Male	1952 to 1961	1957	0.944803487	0.76910264	1.160643044	Low SDI
Deaths	Male	1957 to 1966	1962	0.950196613	0.776818441	1.162271073	Low SDI
Deaths	Male	1962 to 1971	1967	0.94123515	0.771682238	1.148041984	Low SDI
Deaths	Male	1967 to 1976	1972	0.978153465	0.805302885	1.188104771	Low SDI
Deaths	Male	1972 to 1981	1977	1	1	1	Low SDI
Deaths	Male	1977 to 1986	1982	0.992378648	0.799467894	1.231838562	Low SDI
Deaths	Male	1982 to 1991	1987	1.022041271	0.79683126	1.310902838	Low SDI
Deaths	Male	1987 to 1996	1992	1.01536965	0.768191913	1.342080682	Low SDI
Deaths	Male	1992 to 2001	1997	1.011825859	0.732878364	1.396946094	Low SDI
Deaths	Male	1997 to 2006	2002	0.972004729	0.639356023	1.477726273	Low SDI
Deaths	Male	1927 to 1936	1932	1.252551194	1.050860565	1.492952105	Middle SDI
Deaths	Male	1932 to 1941	1937	1.232946526	1.062416672	1.430848344	Middle SDI
Deaths	Male	1937 to 1946	1942	1.263151963	1.097680039	1.453568275	Middle SDI
Deaths	Male	1942 to 1951	1947	1.208237101	1.055540813	1.383022687	Middle SDI
Deaths	Male	1947 to 1956	1952	1.139520298	1.002794949	1.294887364	Middle SDI
Deaths	Male	1952 to 1961	1957	1.163281493	1.030910948	1.312648618	Middle SDI
Deaths	Male	1957 to 1966	1962	1.110158141	0.985769619	1.250242527	Middle SDI
Deaths	Male	1962 to 1971	1967	1.001163759	0.889786644	1.126482263	Middle SDI

Deaths	Male	1967 to 1976	1972	0.989356749	0.878513484	1.114185263	Middle SDI
Deaths	Male	1972 to 1981	1977	1	1	1	Middle SDI
Deaths	Male	1977 to 1986	1982	0.990882704	0.864918863	1.13519149	Middle SDI
Deaths	Male	1982 to 1991	1987	0.896363513	0.765811447	1.049171505	Middle SDI
Deaths	Male	1987 to 1996	1992	0.866591822	0.718766829	1.044819203	Middle SDI
Deaths	Male	1992 to 2001	1997	0.839916945	0.663965487	1.062495698	Middle SDI
Deaths	Male	1997 to 2006	2002	0.724152271	0.514048265	1.020130887	Middle SDI
Deaths	Male	1927 to 1936	1932	1.55512873	1.439710386	1.679799902	Global
Deaths	Male	1932 to 1941	1937	1.399159142	1.30526633	1.499806025	Global
Deaths	Male	1937 to 1946	1942	1.338396166	1.251011313	1.431884971	Global
Deaths	Male	1942 to 1951	1947	1.270963798	1.189944676	1.357499224	Global
Deaths	Male	1947 to 1956	1952	1.203519633	1.130002814	1.281819381	Global
Deaths	Male	1952 to 1961	1957	1.167647233	1.099372468	1.240162092	Global
Deaths	Male	1957 to 1966	1962	1.105244299	1.041017037	1.173434167	Global
Deaths	Male	1962 to 1971	1967	0.997126471	0.938892787	1.058972028	Global
Deaths	Male	1967 to 1976	1972	0.9758353	0.917950197	1.037370585	Global
Deaths	Male	1972 to 1981	1977	1	1	1	Global
Deaths	Male	1977 to 1986	1982	0.988075382	0.920497261	1.060614737	Global
Deaths	Male	1982 to 1991	1987	0.933488588	0.859627405	1.013696096	Global
Deaths	Male	1987 to 1996	1992	0.909232687	0.824233947	1.002996882	Global
Deaths	Male	1992 to 2001	1997	0.88975893	0.786905572	1.006055849	Global
Deaths	Male	1997 to 2006	2002	0.828133174	0.694813461	0.987034063	Global
Deaths	Female	1927 to 1936	1932	2.053201271	1.656777967	2.544478224	High SDI
Deaths	Female	1932 to 1941	1937	1.751959088	1.426094754	2.152283808	High SDI

Deaths	Female	1937 to 1946	1942	1.548880972	1.26222937	1.900630996	High SDI
Deaths	Female	1942 to 1951	1947	1.375309118	1.122587278	1.684924824	High SDI
Deaths	Female	1947 to 1956	1952	1.295753273	1.062848173	1.579695564	High SDI
Deaths	Female	1952 to 1961	1957	1.243275293	1.02567693	1.507037362	High SDI
Deaths	Female	1957 to 1966	1962	1.174257561	0.968358051	1.423936961	High SDI
Deaths	Female	1962 to 1971	1967	1.094386204	0.899202414	1.331937219	High SDI
Deaths	Female	1967 to 1976	1972	1.032292448	0.841299093	1.266645486	High SDI
Deaths	Female	1972 to 1981	1977	1	1	1	High SDI
Deaths	Female	1977 to 1986	1982	1.018275395	0.795891312	1.302796959	High SDI
Deaths	Female	1982 to 1991	1987	1.025137127	0.765455638	1.372915787	High SDI
Deaths	Female	1987 to 1996	1992	0.959236837	0.659565554	1.395062712	High SDI
Deaths	Female	1992 to 2001	1997	0.855310816	0.509372344	1.436192209	High SDI
Deaths	Female	1997 to 2006	2002	0.753200947	0.331718349	1.710220938	High SDI
Deaths	Female	1927 to 1936	1932	0.820377579	0.635574299	1.058915335	Low-middle SDI
Deaths	Female	1932 to 1941	1937	0.81634333	0.655778013	1.016222592	Low-middle SDI
Deaths	Female	1937 to 1946	1942	0.857343285	0.693116891	1.06048131	Low-middle SDI
Deaths	Female	1942 to 1951	1947	0.875941733	0.712787699	1.076441024	Low-middle SDI
Deaths	Female	1947 to 1956	1952	0.883955757	0.727397077	1.074210777	Low-middle SDI
Deaths	Female	1952 to 1961	1957	0.896399064	0.746492844	1.076408553	Low-middle SDI
Deaths	Female	1957 to 1966	1962	0.971808956	0.813325596	1.161174136	Low-middle SDI
Deaths	Female	1962 to 1971	1967	0.94636653	0.793336656	1.128914947	Low-middle SDI
Deaths	Female	1967 to 1976	1972	0.964416065	0.811097902	1.146715266	Low-middle SDI
Deaths	Female	1972 to 1981	1977	1	1	1	Low-middle SDI
Deaths	Female	1977 to 1986	1982	1.013409367	0.841175703	1.220908475	Low-middle SDI

Deaths	Female	1982 to 1991	1987	1.015879574	0.827947832	1.246469003	Low-middle SDI
Deaths	Female	1987 to 1996	1992	0.987968133	0.779462179	1.252249382	Low-middle SDI
Deaths	Female	1992 to 2001	1997	0.949651793	0.718384814	1.255369699	Low-middle SDI
Deaths	Female	1997 to 2006	2002	0.936654465	0.642613383	1.365240141	Low-middle SDI
Deaths	Female	1927 to 1936	1932	1.239210905	0.9375432	1.637944435	High-middle SDI
Deaths	Female	1932 to 1941	1937	1.134916619	0.872863893	1.475643273	High-middle SDI
Deaths	Female	1937 to 1946	1942	1.116367011	0.859009169	1.450828871	High-middle SDI
Deaths	Female	1942 to 1951	1947	1.049857459	0.811488056	1.358246341	High-middle SDI
Deaths	Female	1947 to 1956	1952	0.961087914	0.749828078	1.231869019	High-middle SDI
Deaths	Female	1952 to 1961	1957	0.96737615	0.762389742	1.227477974	High-middle SDI
Deaths	Female	1957 to 1966	1962	0.920762908	0.72676349	1.166547774	High-middle SDI
Deaths	Female	1962 to 1971	1967	0.853587426	0.672629583	1.083228439	High-middle SDI
Deaths	Female	1967 to 1976	1972	0.91353991	0.717490534	1.163158437	High-middle SDI
Deaths	Female	1972 to 1981	1977	1	1	1	High-middle SDI
Deaths	Female	1977 to 1986	1982	0.990651622	0.746203727	1.31517788	High-middle SDI
Deaths	Female	1982 to 1991	1987	0.911283922	0.655455271	1.266964235	High-middle SDI
Deaths	Female	1987 to 1996	1992	0.980208662	0.649909706	1.478373091	High-middle SDI
Deaths	Female	1992 to 2001	1997	1.033705694	0.610409124	1.750543069	High-middle SDI
Deaths	Female	1997 to 2006	2002	1.068435504	0.509880985	2.23886448	High-middle SDI
Deaths	Female	1927 to 1936	1932	1.128401839	0.772601627	1.648055952	Low SDI
Deaths	Female	1932 to 1941	1937	1.110828523	0.801419085	1.539693814	Low SDI
Deaths	Female	1937 to 1946	1942	1.126045522	0.8176302	1.550797069	Low SDI
Deaths	Female	1942 to 1951	1947	1.115079668	0.816537564	1.522774605	Low SDI
Deaths	Female	1947 to 1956	1952	1.090739291	0.810853682	1.467234137	Low SDI

Deaths	Female	1952 to 1961	1957	1.094706412	0.828632708	1.4462163	Low SDI
Deaths	Female	1957 to 1966	1962	1.110124835	0.845619447	1.457366139	Low SDI
Deaths	Female	1962 to 1971	1967	1.042725889	0.796334252	1.365352899	Low SDI
Deaths	Female	1967 to 1976	1972	1.022899267	0.790313415	1.323934139	Low SDI
Deaths	Female	1972 to 1981	1977	1	1	1	Low SDI
Deaths	Female	1977 to 1986	1982	1.011234447	0.773876901	1.321392465	Low SDI
Deaths	Female	1982 to 1991	1987	1.00626362	0.758660117	1.334677348	Low SDI
Deaths	Female	1987 to 1996	1992	0.981524997	0.719242849	1.339452067	Low SDI
Deaths	Female	1992 to 2001	1997	0.953251794	0.675983561	1.344247159	Low SDI
Deaths	Female	1997 to 2006	2002	0.938066126	0.593385565	1.482961683	Low SDI
Deaths	Female	1927 to 1936	1932	1.225514541	0.972176949	1.544868855	Middle SDI
Deaths	Female	1932 to 1941	1937	1.239973159	1.015620119	1.513886351	Middle SDI
Deaths	Female	1937 to 1946	1942	1.2455086	1.027475521	1.509808886	Middle SDI
Deaths	Female	1942 to 1951	1947	1.200202114	0.99661363	1.445379704	Middle SDI
Deaths	Female	1947 to 1956	1952	1.11729473	0.936478028	1.333023816	Middle SDI
Deaths	Female	1952 to 1961	1957	1.153901027	0.976867367	1.363017769	Middle SDI
Deaths	Female	1957 to 1966	1962	1.111504291	0.943903784	1.308864112	Middle SDI
Deaths	Female	1962 to 1971	1967	0.990187258	0.841506376	1.165137702	Middle SDI
Deaths	Female	1967 to 1976	1972	0.995685696	0.845485503	1.1725689	Middle SDI
Deaths	Female	1972 to 1981	1977	1	1	1	Middle SDI
Deaths	Female	1977 to 1986	1982	0.973650992	0.810401519	1.169785881	Middle SDI
Deaths	Female	1982 to 1991	1987	0.879181498	0.714319217	1.082093395	Middle SDI
Deaths	Female	1987 to 1996	1992	0.86900181	0.677265232	1.115019802	Middle SDI
Deaths	Female	1992 to 2001	1997	0.844293431	0.620713246	1.148406938	Middle SDI

Deaths	Female	1997 to 2006	2002	0.731774952	0.475341108	1.126548012	Middle SDI
Deaths	Female	1927 to 1936	1932	1.436706714	1.288318889	1.602185763	Global
Deaths	Female	1932 to 1941	1937	1.286955398	1.16427797	1.422559079	Global
Deaths	Female	1937 to 1946	1942	1.240316036	1.123595773	1.369161319	Global
Deaths	Female	1942 to 1951	1947	1.168615474	1.06072709	1.287477372	Global
Deaths	Female	1947 to 1956	1952	1.090403694	0.993529279	1.196723882	Global
Deaths	Female	1952 to 1961	1957	1.081702941	0.9899256	1.181989084	Global
Deaths	Female	1957 to 1966	1962	1.061547646	0.972387895	1.158882593	Global
Deaths	Female	1962 to 1971	1967	0.967255988	0.885736801	1.05627783	Global
Deaths	Female	1967 to 1976	1972	0.971042494	0.888740554	1.060966016	Global
Deaths	Female	1972 to 1981	1977	1	1	1	Global
Deaths	Female	1977 to 1986	1982	1.00673014	0.911823884	1.111514616	Global
Deaths	Female	1982 to 1991	1987	0.970524932	0.868607202	1.084401145	Global
Deaths	Female	1987 to 1996	1992	0.971267425	0.851897669	1.107363531	Global
Deaths	Female	1992 to 2001	1997	0.978149499	0.836137806	1.144280805	Global
Deaths	Female	1997 to 2006	2002	0.941456943	0.757892522	1.169481358	Global
Deaths	Both	1927 to 1936	1932	2.219524775	1.975186186	2.494089044	High SDI
Deaths	Both	1932 to 1941	1937	1.946830614	1.7434588	2.173925441	High SDI
Deaths	Both	1937 to 1946	1942	1.738625545	1.559026397	1.938914434	High SDI
Deaths	Both	1942 to 1951	1947	1.566860484	1.406758459	1.745183589	High SDI
Deaths	Both	1947 to 1956	1952	1.493622425	1.344808449	1.658903877	High SDI
Deaths	Both	1952 to 1961	1957	1.382142768	1.248027853	1.53066987	High SDI
Deaths	Both	1957 to 1966	1962	1.272314403	1.148362532	1.409645382	High SDI
Deaths	Both	1962 to 1971	1967	1.150899902	1.036479834	1.277951139	High SDI

Deaths	Both	1967 to 1976	1972	1.052880994	0.943954154	1.17437736	High SDI
Deaths	Both	1972 to 1981	1977	1	1	1	High SDI
Deaths	Both	1977 to 1986	1982	0.985859524	0.862860634	1.126391636	High SDI
Deaths	Both	1982 to 1991	1987	0.966503583	0.824723524	1.132657368	High SDI
Deaths	Both	1987 to 1996	1992	0.883047531	0.7205696	1.082161865	High SDI
Deaths	Both	1992 to 2001	1997	0.758897215	0.568994748	1.012179788	High SDI
Deaths	Both	1997 to 2006	2002	0.624458274	0.386897153	1.007885773	High SDI
Deaths	Both	1927 to 1936	1932	0.833949002	0.714350916	0.973570444	Low-middle SDI
Deaths	Both	1932 to 1941	1937	0.806647604	0.707382381	0.91984247	Low-middle SDI
Deaths	Both	1937 to 1946	1942	0.847544732	0.747851118	0.960528178	Low-middle SDI
Deaths	Both	1942 to 1951	1947	0.863582353	0.765342805	0.974431948	Low-middle SDI
Deaths	Both	1947 to 1956	1952	0.874318553	0.780224952	0.979759659	Low-middle SDI
Deaths	Both	1952 to 1961	1957	0.90423075	0.812692035	1.006080057	Low-middle SDI
Deaths	Both	1957 to 1966	1962	0.948846893	0.855014127	1.052977253	Low-middle SDI
Deaths	Both	1962 to 1971	1967	0.931643255	0.840493761	1.032677687	Low-middle SDI
Deaths	Both	1967 to 1976	1972	0.94869683	0.856711731	1.050558366	Low-middle SDI
Deaths	Both	1972 to 1981	1977	1	1	1	Low-middle SDI
Deaths	Both	1977 to 1986	1982	1.008084242	0.901042515	1.127842273	Low-middle SDI
Deaths	Both	1982 to 1991	1987	1.025929245	0.904272231	1.163953486	Low-middle SDI
Deaths	Both	1987 to 1996	1992	1.006436382	0.869900328	1.164402585	Low-middle SDI
Deaths	Both	1992 to 2001	1997	0.983766767	0.82599922	1.17166824	Low-middle SDI
Deaths	Both	1997 to 2006	2002	0.969822246	0.762495064	1.233522985	Low-middle SDI
Deaths	Both	1927 to 1936	1932	1.294640633	1.11389259	1.504718125	High-middle SDI
Deaths	Both	1932 to 1941	1937	1.200106306	1.045041016	1.378180496	High-middle SDI

Deaths	Both	1937 to 1946	1942	1.182023555	1.030914662	1.355281612	High-middle SDI
Deaths	Both	1942 to 1951	1947	1.125263749	0.984102718	1.28667311	High-middle SDI
Deaths	Both	1947 to 1956	1952	1.045224887	0.91897951	1.188813301	High-middle SDI
Deaths	Both	1952 to 1961	1957	1.041663546	0.920712068	1.178504097	High-middle SDI
Deaths	Both	1957 to 1966	1962	0.991228459	0.876836033	1.120544572	High-middle SDI
Deaths	Both	1962 to 1971	1967	0.887944568	0.784626399	1.004867484	High-middle SDI
Deaths	Both	1967 to 1976	1972	0.913836466	0.805727806	1.036450622	High-middle SDI
Deaths	Both	1972 to 1981	1977	1	1	1	High-middle SDI
Deaths	Both	1977 to 1986	1982	0.966734945	0.83302626	1.121905152	High-middle SDI
Deaths	Both	1982 to 1991	1987	0.833025909	0.698070085	0.994072344	High-middle SDI
Deaths	Both	1987 to 1996	1992	0.852846962	0.683674232	1.063880874	High-middle SDI
Deaths	Both	1992 to 2001	1997	0.893486449	0.668877173	1.193519628	High-middle SDI
Deaths	Both	1997 to 2006	2002	0.888210622	0.582686551	1.353932245	High-middle SDI
Deaths	Both	1927 to 1936	1932	1.000021882	0.790438458	1.265175998	Low SDI
Deaths	Both	1932 to 1941	1937	0.992206354	0.812879388	1.211094123	Low SDI
Deaths	Both	1937 to 1946	1942	0.995885159	0.821707446	1.206983404	Low SDI
Deaths	Both	1942 to 1951	1947	0.998783831	0.829015232	1.203318229	Low SDI
Deaths	Both	1947 to 1956	1952	0.98420414	0.825417723	1.173536454	Low SDI
Deaths	Both	1952 to 1961	1957	0.989546679	0.839138005	1.166914887	Low SDI
Deaths	Both	1957 to 1966	1962	0.997779905	0.848990202	1.172645734	Low SDI
Deaths	Both	1962 to 1971	1967	0.969448888	0.826529193	1.137081611	Low SDI
Deaths	Both	1967 to 1976	1972	0.987737924	0.845886812	1.153376779	Low SDI
Deaths	Both	1972 to 1981	1977	1	1	1	Low SDI
Deaths	Both	1977 to 1986	1982	1.001249583	0.846881938	1.183754998	Low SDI

Deaths	Both	1982 to 1991	1987	1.010462961	0.838860213	1.217169892	Low SDI
Deaths	Both	1987 to 1996	1992	0.994463959	0.808583292	1.223075689	Low SDI
Deaths	Both	1992 to 2001	1997	0.986396621	0.780810016	1.246114001	Low SDI
Deaths	Both	1997 to 2006	2002	0.955882064	0.702094988	1.301405843	Low SDI
Deaths	Both	1927 to 1936	1932	1.258660407	1.094438402	1.447524151	Middle SDI
Deaths	Both	1932 to 1941	1937	1.25258095	1.111778168	1.411215908	Middle SDI
Deaths	Both	1937 to 1946	1942	1.270480505	1.134214709	1.423117423	Middle SDI
Deaths	Both	1942 to 1951	1947	1.216220148	1.090267534	1.356723373	Middle SDI
Deaths	Both	1947 to 1956	1952	1.140852874	1.028612705	1.265340466	Middle SDI
Deaths	Both	1952 to 1961	1957	1.167474805	1.058661122	1.28747282	Middle SDI
Deaths	Both	1957 to 1966	1962	1.114592612	1.012430785	1.227063329	Middle SDI
Deaths	Both	1962 to 1971	1967	0.998488553	0.907521605	1.098573725	Middle SDI
Deaths	Both	1967 to 1976	1972	0.992902017	0.901852323	1.093143954	Middle SDI
Deaths	Both	1972 to 1981	1977	1	1	1	Middle SDI
Deaths	Both	1977 to 1986	1982	0.98352479	0.881642718	1.097180291	Middle SDI
Deaths	Both	1982 to 1991	1987	0.890109199	0.785112886	1.009147092	Middle SDI
Deaths	Both	1987 to 1996	1992	0.867892351	0.747154083	1.008141628	Middle SDI
Deaths	Both	1992 to 2001	1997	0.85337358	0.708250398	1.028233049	Middle SDI
Deaths	Both	1997 to 2006	2002	0.734311701	0.561478082	0.960346791	Middle SDI
Deaths	Both	1927 to 1936	1932	1.528410309	1.435288681	1.627573675	Global
Deaths	Both	1932 to 1941	1937	1.373067708	1.296963817	1.45363726	Global
Deaths	Both	1937 to 1946	1942	1.318039717	1.246617911	1.393553454	Global
Deaths	Both	1942 to 1951	1947	1.248121501	1.181995187	1.317947228	Global
Deaths	Both	1947 to 1956	1952	1.173404775	1.113773151	1.236229087	Global

Deaths	Both	1952 to 1961	1957	1.145735882	1.090065769	1.20424909	Global
Deaths	Both	1957 to 1966	1962	1.094900295	1.04209672	1.150379454	Global
Deaths	Both	1962 to 1971	1967	0.988899356	0.940979339	1.039259733	Global
Deaths	Both	1967 to 1976	1972	0.975966186	0.928082119	1.026320814	Global
Deaths	Both	1972 to 1981	1977	1	1	1	Global
Deaths	Both	1977 to 1986	1982	0.995242337	0.939528669	1.054259803	Global
Deaths	Both	1982 to 1991	1987	0.947107813	0.886483728	1.011877806	Global
Deaths	Both	1987 to 1996	1992	0.932726526	0.862264304	1.008946755	Global
Deaths	Both	1992 to 2001	1997	0.924205359	0.839066804	1.017982767	Global
Deaths	Both	1997 to 2006	2002	0.871392753	0.760339225	0.998666524	Global
DALYs (Disability-Adjusted Life Years)	Male	1927 to 1936	1932	2.25740912	2.146908715	2.373596931	High SDI
DALYs (Disability-Adjusted Life Years)	Male	1932 to 1941	1937	2.018531439	1.928755372	2.112486234	High SDI
DALYs (Disability-Adjusted Life Years)	Male	1937 to 1946	1942	1.81396765	1.735887056	1.895560328	High SDI
DALYs (Disability-Adjusted Life Years)	Male	1942 to 1951	1947	1.652851443	1.583570626	1.725163279	High SDI
DALYs (Disability-Adjusted Life Years)	Male	1947 to 1956	1952	1.583290188	1.519347527	1.649923914	High SDI
DALYs (Disability-Adjusted Life Years)	Male	1952 to 1961	1957	1.447383301	1.390720465	1.506354779	High SDI
DALYs (Disability-Adjusted Life Years)	Male	1957 to 1966	1962	1.310350967	1.259052868	1.363739126	High SDI
DALYs (Disability-Adjusted Life Years)	Male	1962 to 1971	1967	1.169248254	1.122732786	1.217690884	High SDI
DALYs (Disability-Adjusted Life Years)	Male	1967 to 1976	1972	1.057932103	1.014493407	1.103230763	High SDI
DALYs (Disability-Adjusted Life Years)	Male	1972 to 1981	1977	1	1	1	High SDI
DALYs (Disability-Adjusted Life Years)	Male	1977 to 1986	1982	0.973766879	0.926840051	1.023069659	High SDI
DALYs (Disability-Adjusted Life Years)	Male	1982 to 1991	1987	0.937479096	0.885148231	0.992903815	High SDI
DALYs (Disability-Adjusted Life Years)	Male	1987 to 1996	1992	0.845802807	0.787285788	0.908669252	High SDI
DALYs (Disability-Adjusted Life Years)	Male	1992 to 2001	1997	0.737018966	0.667728744	0.813499436	High SDI

DALYs (Disability-Adjusted Life Years)	Male	1997 to 2006	2002	0.587368513	0.498894473	0.691532556	High SDI
DALYs (Disability-Adjusted Life Years)	Male	1927 to 1936	1932	0.819420268	0.776226052	0.865018089	Low-middle SDI
DALYs (Disability-Adjusted Life Years)	Male	1932 to 1941	1937	0.781736581	0.748948856	0.815959698	Low-middle SDI
DALYs (Disability-Adjusted Life Years)	Male	1937 to 1946	1942	0.820865495	0.789691889	0.8532697	Low-middle SDI
DALYs (Disability-Adjusted Life Years)	Male	1942 to 1951	1947	0.838341568	0.808427698	0.869362327	Low-middle SDI
DALYs (Disability-Adjusted Life Years)	Male	1947 to 1956	1952	0.855163301	0.826924708	0.884366213	Low-middle SDI
DALYs (Disability-Adjusted Life Years)	Male	1952 to 1961	1957	0.894864894	0.867443062	0.923153593	Low-middle SDI
DALYs (Disability-Adjusted Life Years)	Male	1957 to 1966	1962	0.921302273	0.894054222	0.949380761	Low-middle SDI
DALYs (Disability-Adjusted Life Years)	Male	1962 to 1971	1967	0.919438901	0.892970747	0.946691585	Low-middle SDI
DALYs (Disability-Adjusted Life Years)	Male	1967 to 1976	1972	0.94382344	0.917255764	0.971160631	Low-middle SDI
DALYs (Disability-Adjusted Life Years)	Male	1972 to 1981	1977	1	1	1	Low-middle SDI
DALYs (Disability-Adjusted Life Years)	Male	1977 to 1986	1982	1.007037831	0.976842661	1.038166363	Low-middle SDI
DALYs (Disability-Adjusted Life Years)	Male	1982 to 1991	1987	1.033808714	0.999429525	1.069370506	Low-middle SDI
DALYs (Disability-Adjusted Life Years)	Male	1987 to 1996	1992	1.01851445	0.980347488	1.058167331	Low-middle SDI
DALYs (Disability-Adjusted Life Years)	Male	1992 to 2001	1997	1.008060616	0.963265376	1.054938992	Low-middle SDI
DALYs (Disability-Adjusted Life Years)	Male	1997 to 2006	2002	0.993376946	0.933514601	1.05707801	Low-middle SDI
DALYs (Disability-Adjusted Life Years)	Male	1927 to 1936	1932	1.292531243	1.226034024	1.362635115	High-middle SDI
DALYs (Disability-Adjusted Life Years)	Male	1932 to 1941	1937	1.213858566	1.15936605	1.270912339	High-middle SDI
DALYs (Disability-Adjusted Life Years)	Male	1937 to 1946	1942	1.194379861	1.142675409	1.248423865	High-middle SDI
DALYs (Disability-Adjusted Life Years)	Male	1942 to 1951	1947	1.144668591	1.096952874	1.194459866	High-middle SDI
DALYs (Disability-Adjusted Life Years)	Male	1947 to 1956	1952	1.075689776	1.033248482	1.119874371	High-middle SDI
DALYs (Disability-Adjusted Life Years)	Male	1952 to 1961	1957	1.06823562	1.028048019	1.109994202	High-middle SDI
DALYs (Disability-Adjusted Life Years)	Male	1957 to 1966	1962	1.01614933	0.978483413	1.055265166	High-middle SDI
DALYs (Disability-Adjusted Life Years)	Male	1962 to 1971	1967	0.904832772	0.871337006	0.939616175	High-middle SDI

DALYs (Disability-Adjusted Life Years)	Male	1967 to 1976	1972	0.916308283	0.882203377	0.951731644	High-middle SDI
DALYs (Disability-Adjusted Life Years)	Male	1972 to 1981	1977	1	1	1	High-middle SDI
DALYs (Disability-Adjusted Life Years)	Male	1977 to 1986	1982	0.956063239	0.915688636	0.998218041	High-middle SDI
DALYs (Disability-Adjusted Life Years)	Male	1982 to 1991	1987	0.804481166	0.765046688	0.845948302	High-middle SDI
DALYs (Disability-Adjusted Life Years)	Male	1987 to 1996	1992	0.805874462	0.758004123	0.856767964	High-middle SDI
DALYs (Disability-Adjusted Life Years)	Male	1992 to 2001	1997	0.839141701	0.775383426	0.908142696	High-middle SDI
DALYs (Disability-Adjusted Life Years)	Male	1997 to 2006	2002	0.790873634	0.704208231	0.888204763	High-middle SDI
DALYs (Disability-Adjusted Life Years)	Male	1927 to 1936	1932	0.928814442	0.882927276	0.977086439	Low SDI
DALYs (Disability-Adjusted Life Years)	Male	1932 to 1941	1937	0.928277592	0.892071487	0.965953179	Low SDI
DALYs (Disability-Adjusted Life Years)	Male	1937 to 1946	1942	0.933229823	0.89983296	0.967866194	Low SDI
DALYs (Disability-Adjusted Life Years)	Male	1942 to 1951	1947	0.941729327	0.909971473	0.974595524	Low SDI
DALYs (Disability-Adjusted Life Years)	Male	1947 to 1956	1952	0.932470611	0.90329296	0.962590742	Low SDI
DALYs (Disability-Adjusted Life Years)	Male	1952 to 1961	1957	0.937801101	0.910604118	0.965810376	Low SDI
DALYs (Disability-Adjusted Life Years)	Male	1957 to 1966	1962	0.944410328	0.917904042	0.971682036	Low SDI
DALYs (Disability-Adjusted Life Years)	Male	1962 to 1971	1967	0.935353292	0.909828454	0.961594218	Low SDI
DALYs (Disability-Adjusted Life Years)	Male	1967 to 1976	1972	0.975118901	0.949541975	1.001384769	Low SDI
DALYs (Disability-Adjusted Life Years)	Male	1972 to 1981	1977	1	1	1	Low SDI
DALYs (Disability-Adjusted Life Years)	Male	1977 to 1986	1982	0.997347277	0.969606371	1.025881863	Low SDI
DALYs (Disability-Adjusted Life Years)	Male	1982 to 1991	1987	1.023447985	0.991626003	1.056291157	Low SDI
DALYs (Disability-Adjusted Life Years)	Male	1987 to 1996	1992	1.027539245	0.992672229	1.063630944	Low SDI
DALYs (Disability-Adjusted Life Years)	Male	1992 to 2001	1997	1.017923818	0.978802604	1.058608646	Low SDI
DALYs (Disability-Adjusted Life Years)	Male	1997 to 2006	2002	0.978221762	0.930342612	1.028564964	Low SDI
DALYs (Disability-Adjusted Life Years)	Male	1927 to 1936	1932	1.231338228	1.166597125	1.299672182	Middle SDI
DALYs (Disability-Adjusted Life Years)	Male	1932 to 1941	1937	1.218065269	1.166616467	1.271783008	Middle SDI

DALYs (Disability-Adjusted Life Years)	Male	1937 to 1946	1942	1.252530852	1.204392731	1.302592995	Middle SDI
DALYs (Disability-Adjusted Life Years)	Male	1942 to 1951	1947	1.203358306	1.159920987	1.248422287	Middle SDI
DALYs (Disability-Adjusted Life Years)	Male	1947 to 1956	1952	1.138443602	1.100242535	1.177971033	Middle SDI
DALYs (Disability-Adjusted Life Years)	Male	1952 to 1961	1957	1.160614998	1.12415612	1.19825632	Middle SDI
DALYs (Disability-Adjusted Life Years)	Male	1957 to 1966	1962	1.108497628	1.074665627	1.143394709	Middle SDI
DALYs (Disability-Adjusted Life Years)	Male	1962 to 1971	1967	1.001256925	0.971339756	1.032095539	Middle SDI
DALYs (Disability-Adjusted Life Years)	Male	1967 to 1976	1972	0.988388206	0.959094302	1.018576841	Middle SDI
DALYs (Disability-Adjusted Life Years)	Male	1972 to 1981	1977	1	1	1	Middle SDI
DALYs (Disability-Adjusted Life Years)	Male	1977 to 1986	1982	0.992469563	0.960223509	1.025798498	Middle SDI
DALYs (Disability-Adjusted Life Years)	Male	1982 to 1991	1987	0.897710167	0.864864019	0.93180376	Middle SDI
DALYs (Disability-Adjusted Life Years)	Male	1987 to 1996	1992	0.865429104	0.82874061	0.903741805	Middle SDI
DALYs (Disability-Adjusted Life Years)	Male	1992 to 2001	1997	0.844213873	0.800416366	0.890407909	Middle SDI
DALYs (Disability-Adjusted Life Years)	Male	1997 to 2006	2002	0.734911604	0.681177553	0.792884414	Middle SDI
DALYs (Disability-Adjusted Life Years)	Male	1927 to 1936	1932	1.530247538	1.465049202	1.598347363	Global
DALYs (Disability-Adjusted Life Years)	Male	1932 to 1941	1937	1.387518384	1.336628139	1.440346203	Global
DALYs (Disability-Adjusted Life Years)	Male	1937 to 1946	1942	1.331494407	1.285262752	1.379389042	Global
DALYs (Disability-Adjusted Life Years)	Male	1942 to 1951	1947	1.268845968	1.226678444	1.312463015	Global
DALYs (Disability-Adjusted Life Years)	Male	1947 to 1956	1952	1.201870674	1.164168833	1.240793496	Global
DALYs (Disability-Adjusted Life Years)	Male	1952 to 1961	1957	1.165919419	1.131188093	1.201717115	Global
DALYs (Disability-Adjusted Life Years)	Male	1957 to 1966	1962	1.101840941	1.069522605	1.135135857	Global
DALYs (Disability-Adjusted Life Years)	Male	1962 to 1971	1967	0.996116325	0.967059587	1.026046115	Global
DALYs (Disability-Adjusted Life Years)	Male	1967 to 1976	1972	0.976755061	0.948234969	1.006132952	Global
DALYs (Disability-Adjusted Life Years)	Male	1972 to 1981	1977	1	1	1	Global
DALYs (Disability-Adjusted Life Years)	Male	1977 to 1986	1982	0.991066579	0.958999371	1.024206056	Global

DALYs (Disability-Adjusted Life Years)	Male	1982 to 1991	1987	0.935494754	0.901268943	0.971020294	Global
DALYs (Disability-Adjusted Life Years)	Male	1987 to 1996	1992	0.912573569	0.873885767	0.952974119	Global
DALYs (Disability-Adjusted Life Years)	Male	1992 to 2001	1997	0.894595757	0.848381382	0.943327594	Global
DALYs (Disability-Adjusted Life Years)	Male	1997 to 2006	2002	0.832140192	0.772521705	0.896359668	Global
DALYs (Disability-Adjusted Life Years)	Female	1927 to 1936	1932	1.998951545	1.903544932	2.099139984	High SDI
DALYs (Disability-Adjusted Life Years)	Female	1932 to 1941	1937	1.722653781	1.645841524	1.803050905	High SDI
DALYs (Disability-Adjusted Life Years)	Female	1937 to 1946	1942	1.532679549	1.465632496	1.602793747	High SDI
DALYs (Disability-Adjusted Life Years)	Female	1942 to 1951	1947	1.362114115	1.30358897	1.423266769	High SDI
DALYs (Disability-Adjusted Life Years)	Female	1947 to 1956	1952	1.285050242	1.23155855	1.3408653	High SDI
DALYs (Disability-Adjusted Life Years)	Female	1952 to 1961	1957	1.232635201	1.183015189	1.284336459	High SDI
DALYs (Disability-Adjusted Life Years)	Female	1957 to 1966	1962	1.169386989	1.12250803	1.21822374	High SDI
DALYs (Disability-Adjusted Life Years)	Female	1962 to 1971	1967	1.089079461	1.044840274	1.135191762	High SDI
DALYs (Disability-Adjusted Life Years)	Female	1967 to 1976	1972	1.023514062	0.980668051	1.068232042	High SDI
DALYs (Disability-Adjusted Life Years)	Female	1972 to 1981	1977	1	1	1	High SDI
DALYs (Disability-Adjusted Life Years)	Female	1977 to 1986	1982	1.012268927	0.963411129	1.063604466	High SDI
DALYs (Disability-Adjusted Life Years)	Female	1982 to 1991	1987	1.01101149	0.954800342	1.070531908	High SDI
DALYs (Disability-Adjusted Life Years)	Female	1987 to 1996	1992	0.945044485	0.880037413	1.014853534	High SDI
DALYs (Disability-Adjusted Life Years)	Female	1992 to 2001	1997	0.832181364	0.755511119	0.916632203	High SDI
DALYs (Disability-Adjusted Life Years)	Female	1997 to 2006	2002	0.761686051	0.656277072	0.88402546	High SDI
DALYs (Disability-Adjusted Life Years)	Female	1927 to 1936	1932	0.816555311	0.766446971	0.869939606	Low-middle SDI
DALYs (Disability-Adjusted Life Years)	Female	1932 to 1941	1937	0.812854387	0.772440415	0.855382812	Low-middle SDI
DALYs (Disability-Adjusted Life Years)	Female	1937 to 1946	1942	0.85329256	0.813409092	0.895131613	Low-middle SDI
DALYs (Disability-Adjusted Life Years)	Female	1942 to 1951	1947	0.869598834	0.830977466	0.910015208	Low-middle SDI
DALYs (Disability-Adjusted Life Years)	Female	1947 to 1956	1952	0.879521051	0.843097335	0.917518354	Low-middle SDI

DALYs (Disability-Adjusted Life Years)	Female	1952 to 1961	1957	0.896414845	0.861838034	0.932378872	Low-middle SDI
DALYs (Disability-Adjusted Life Years)	Female	1957 to 1966	1962	0.966502963	0.930809603	1.003565042	Low-middle SDI
DALYs (Disability-Adjusted Life Years)	Female	1962 to 1971	1967	0.944212728	0.9101925	0.979504528	Low-middle SDI
DALYs (Disability-Adjusted Life Years)	Female	1967 to 1976	1972	0.961830365	0.928450964	0.996409812	Low-middle SDI
DALYs (Disability-Adjusted Life Years)	Female	1972 to 1981	1977	1	1	1	Low-middle SDI
DALYs (Disability-Adjusted Life Years)	Female	1977 to 1986	1982	1.017062017	0.980757382	1.054710539	Low-middle SDI
DALYs (Disability-Adjusted Life Years)	Female	1982 to 1991	1987	1.019060781	0.979969548	1.059711372	Low-middle SDI
DALYs (Disability-Adjusted Life Years)	Female	1987 to 1996	1992	0.990132265	0.947246379	1.034959779	Low-middle SDI
DALYs (Disability-Adjusted Life Years)	Female	1992 to 2001	1997	0.957975575	0.910162258	1.008300657	Low-middle SDI
DALYs (Disability-Adjusted Life Years)	Female	1997 to 2006	2002	0.942013772	0.880110168	1.008271439	Low-middle SDI
DALYs (Disability-Adjusted Life Years)	Female	1927 to 1936	1932	1.199544916	1.139532956	1.262717325	High-middle SDI
DALYs (Disability-Adjusted Life Years)	Female	1932 to 1941	1937	1.114977622	1.064242784	1.168131103	High-middle SDI
DALYs (Disability-Adjusted Life Years)	Female	1937 to 1946	1942	1.102658017	1.053328328	1.154297923	High-middle SDI
DALYs (Disability-Adjusted Life Years)	Female	1942 to 1951	1947	1.042819859	0.997565249	1.090127447	High-middle SDI
DALYs (Disability-Adjusted Life Years)	Female	1947 to 1956	1952	0.952649604	0.913171078	0.993834879	High-middle SDI
DALYs (Disability-Adjusted Life Years)	Female	1952 to 1961	1957	0.958955622	0.921018041	0.998455887	High-middle SDI
DALYs (Disability-Adjusted Life Years)	Female	1957 to 1966	1962	0.917162622	0.881395397	0.95438129	High-middle SDI
DALYs (Disability-Adjusted Life Years)	Female	1962 to 1971	1967	0.85045406	0.817331489	0.884918932	High-middle SDI
DALYs (Disability-Adjusted Life Years)	Female	1967 to 1976	1972	0.907143376	0.871848114	0.943867505	High-middle SDI
DALYs (Disability-Adjusted Life Years)	Female	1972 to 1981	1977	1	1	1	High-middle SDI
DALYs (Disability-Adjusted Life Years)	Female	1977 to 1986	1982	0.990010386	0.94700973	1.034963563	High-middle SDI
DALYs (Disability-Adjusted Life Years)	Female	1982 to 1991	1987	0.892526619	0.848470898	0.938869875	High-middle SDI
DALYs (Disability-Adjusted Life Years)	Female	1987 to 1996	1992	0.950099515	0.893394858	1.010403272	High-middle SDI
DALYs (Disability-Adjusted Life Years)	Female	1992 to 2001	1997	1.022632915	0.947003051	1.104302756	High-middle SDI

DALYs (Disability-Adjusted Life Years)	Female	1997 to 2006	2002	1.083220913	0.97506988	1.203367647	High-middle SDI
DALYs (Disability-Adjusted Life Years)	Female	1927 to 1936	1932	1.113417974	1.046635818	1.184461264	Low SDI
DALYs (Disability-Adjusted Life Years)	Female	1932 to 1941	1937	1.090581985	1.037345914	1.146550104	Low SDI
DALYs (Disability-Adjusted Life Years)	Female	1937 to 1946	1942	1.096466033	1.045481298	1.149937128	Low SDI
DALYs (Disability-Adjusted Life Years)	Female	1942 to 1951	1947	1.090316013	1.041889815	1.140993021	Low SDI
DALYs (Disability-Adjusted Life Years)	Female	1947 to 1956	1952	1.069837693	1.025386511	1.116215863	Low SDI
DALYs (Disability-Adjusted Life Years)	Female	1952 to 1961	1957	1.071032373	1.029440152	1.114305035	Low SDI
DALYs (Disability-Adjusted Life Years)	Female	1957 to 1966	1962	1.08194076	1.041609869	1.123833254	Low SDI
DALYs (Disability-Adjusted Life Years)	Female	1962 to 1971	1967	1.025986291	0.988911404	1.064451138	Low SDI
DALYs (Disability-Adjusted Life Years)	Female	1967 to 1976	1972	1.006704028	0.972719932	1.041875433	Low SDI
DALYs (Disability-Adjusted Life Years)	Female	1972 to 1981	1977	1	1	1	Low SDI
DALYs (Disability-Adjusted Life Years)	Female	1977 to 1986	1982	1.0003916	0.966783185	1.035168349	Low SDI
DALYs (Disability-Adjusted Life Years)	Female	1982 to 1991	1987	1.00210008	0.967084141	1.038383867	Low SDI
DALYs (Disability-Adjusted Life Years)	Female	1987 to 1996	1992	0.971786662	0.935079695	1.009934577	Low SDI
DALYs (Disability-Adjusted Life Years)	Female	1992 to 2001	1997	0.955660633	0.916380804	0.996624156	Low SDI
DALYs (Disability-Adjusted Life Years)	Female	1997 to 2006	2002	0.932333652	0.882194616	0.985322311	Low SDI
DALYs (Disability-Adjusted Life Years)	Female	1927 to 1936	1932	1.207491393	1.117908819	1.304252582	Middle SDI
DALYs (Disability-Adjusted Life Years)	Female	1932 to 1941	1937	1.227057856	1.152526852	1.306408592	Middle SDI
DALYs (Disability-Adjusted Life Years)	Female	1937 to 1946	1942	1.231029942	1.161032263	1.305247725	Middle SDI
DALYs (Disability-Adjusted Life Years)	Female	1942 to 1951	1947	1.190262219	1.126256615	1.257905287	Middle SDI
DALYs (Disability-Adjusted Life Years)	Female	1947 to 1956	1952	1.114570143	1.058508563	1.173600901	Middle SDI
DALYs (Disability-Adjusted Life Years)	Female	1952 to 1961	1957	1.151001596	1.096869836	1.207804819	Middle SDI
DALYs (Disability-Adjusted Life Years)	Female	1957 to 1966	1962	1.10735688	1.056974487	1.160140831	Middle SDI
DALYs (Disability-Adjusted Life Years)	Female	1962 to 1971	1967	0.991497756	0.947201711	1.037865312	Middle SDI

DALYs (Disability-Adjusted Life Years)	Female	1967 to 1976	1972	0.994641894	0.950682586	1.040633869	Middle SDI
DALYs (Disability-Adjusted Life Years)	Female	1972 to 1981	1977	1	1	1	Middle SDI
DALYs (Disability-Adjusted Life Years)	Female	1977 to 1986	1982	0.983032687	0.936358079	1.032033884	Middle SDI
DALYs (Disability-Adjusted Life Years)	Female	1982 to 1991	1987	0.890328199	0.843666949	0.939570175	Middle SDI
DALYs (Disability-Adjusted Life Years)	Female	1987 to 1996	1992	0.880062615	0.826275181	0.93735141	Middle SDI
DALYs (Disability-Adjusted Life Years)	Female	1992 to 2001	1997	0.870152348	0.806536869	0.938785487	Middle SDI
DALYs (Disability-Adjusted Life Years)	Female	1997 to 2006	2002	0.740045313	0.666000769	0.822321973	Middle SDI
DALYs (Disability-Adjusted Life Years)	Female	1927 to 1936	1932	1.419715309	1.35375588	1.488888497	Global
DALYs (Disability-Adjusted Life Years)	Female	1932 to 1941	1937	1.277294827	1.224967646	1.331857278	Global
DALYs (Disability-Adjusted Life Years)	Female	1937 to 1946	1942	1.231761226	1.183054714	1.282472992	Global
DALYs (Disability-Adjusted Life Years)	Female	1942 to 1951	1947	1.163352829	1.118994257	1.20946984	Global
DALYs (Disability-Adjusted Life Years)	Female	1947 to 1956	1952	1.087552467	1.048179424	1.128404491	Global
DALYs (Disability-Adjusted Life Years)	Female	1952 to 1961	1957	1.079823794	1.042801431	1.118160554	Global
DALYs (Disability-Adjusted Life Years)	Female	1957 to 1966	1962	1.056654919	1.021219897	1.093319491	Global
DALYs (Disability-Adjusted Life Years)	Female	1962 to 1971	1967	0.966912565	0.934752103	1.000179519	Global
DALYs (Disability-Adjusted Life Years)	Female	1967 to 1976	1972	0.969357344	0.937459728	1.002340295	Global
DALYs (Disability-Adjusted Life Years)	Female	1972 to 1981	1977	1	1	1	Global
DALYs (Disability-Adjusted Life Years)	Female	1977 to 1986	1982	1.008443625	0.972938749	1.045244159	Global
DALYs (Disability-Adjusted Life Years)	Female	1982 to 1991	1987	0.973129935	0.935639476	1.012122613	Global
DALYs (Disability-Adjusted Life Years)	Female	1987 to 1996	1992	0.975039003	0.931846964	1.020233037	Global
DALYs (Disability-Adjusted Life Years)	Female	1992 to 2001	1997	0.986280803	0.935185171	1.040168144	Global
DALYs (Disability-Adjusted Life Years)	Female	1997 to 2006	2002	0.946943907	0.880938951	1.017894329	Global
DALYs (Disability-Adjusted Life Years)	Both	1927 to 1936	1932	2.154689529	2.069889565	2.242963607	High SDI
DALYs (Disability-Adjusted Life Years)	Both	1932 to 1941	1937	1.912002918	1.843027028	1.983560253	High SDI

DALYs (Disability-Adjusted Life Years)	Both	1937 to 1946	1942	1.717895571	1.657659601	1.780320393	High SDI
DALYs (Disability-Adjusted Life Years)	Both	1942 to 1951	1947	1.555076686	1.501834467	1.610206419	High SDI
DALYs (Disability-Adjusted Life Years)	Both	1947 to 1956	1952	1.481594224	1.4326392	1.5322221	High SDI
DALYs (Disability-Adjusted Life Years)	Both	1952 to 1961	1957	1.374059395	1.330083722	1.419489007	High SDI
DALYs (Disability-Adjusted Life Years)	Both	1957 to 1966	1962	1.262746122	1.22237618	1.304449313	High SDI
DALYs (Disability-Adjusted Life Years)	Both	1962 to 1971	1967	1.142259425	1.105173352	1.180589987	High SDI
DALYs (Disability-Adjusted Life Years)	Both	1967 to 1976	1972	1.046056533	1.011011609	1.082316228	High SDI
DALYs (Disability-Adjusted Life Years)	Both	1972 to 1981	1977	1	1	1	High SDI
DALYs (Disability-Adjusted Life Years)	Both	1977 to 1986	1982	0.985928319	0.94733371	1.026095283	High SDI
DALYs (Disability-Adjusted Life Years)	Both	1982 to 1991	1987	0.961189946	0.917649348	1.006796457	High SDI
DALYs (Disability-Adjusted Life Years)	Both	1987 to 1996	1992	0.875553837	0.82635675	0.92767987	High SDI
DALYs (Disability-Adjusted Life Years)	Both	1992 to 2001	1997	0.765548837	0.707198691	0.828713386	High SDI
DALYs (Disability-Adjusted Life Years)	Both	1997 to 2006	2002	0.638179018	0.561433644	0.725415128	High SDI
DALYs (Disability-Adjusted Life Years)	Both	1927 to 1936	1932	0.83305231	0.791218485	0.877098001	Low-middle SDI
DALYs (Disability-Adjusted Life Years)	Both	1932 to 1941	1937	0.803976616	0.771657985	0.837648818	Low-middle SDI
DALYs (Disability-Adjusted Life Years)	Both	1937 to 1946	1942	0.843567221	0.812414335	0.875914698	Low-middle SDI
DALYs (Disability-Adjusted Life Years)	Both	1942 to 1951	1947	0.859983004	0.830021637	0.89102589	Low-middle SDI
DALYs (Disability-Adjusted Life Years)	Both	1947 to 1956	1952	0.87339667	0.845170697	0.902565298	Low-middle SDI
DALYs (Disability-Adjusted Life Years)	Both	1952 to 1961	1957	0.904061329	0.876917915	0.932044919	Low-middle SDI
DALYs (Disability-Adjusted Life Years)	Both	1957 to 1966	1962	0.943333284	0.916072477	0.971405328	Low-middle SDI
DALYs (Disability-Adjusted Life Years)	Both	1962 to 1971	1967	0.930902924	0.904691091	0.957874198	Low-middle SDI
DALYs (Disability-Adjusted Life Years)	Both	1967 to 1976	1972	0.951009633	0.924961682	0.977791123	Low-middle SDI
DALYs (Disability-Adjusted Life Years)	Both	1972 to 1981	1977	1	1	1	Low-middle SDI
DALYs (Disability-Adjusted Life Years)	Both	1977 to 1986	1982	1.012651728	0.983476425	1.042692531	Low-middle SDI

DALYs (Disability-Adjusted Life Years)	Both	1982 to 1991	1987	1.030901988	0.998349289	1.064516116	Low-middle SDI
DALYs (Disability-Adjusted Life Years)	Both	1987 to 1996	1992	1.010408027	0.974413695	1.04773197	Low-middle SDI
DALYs (Disability-Adjusted Life Years)	Both	1992 to 2001	1997	0.99108584	0.949676379	1.03430091	Low-middle SDI
DALYs (Disability-Adjusted Life Years)	Both	1997 to 2006	2002	0.974749597	0.920124079	1.032618099	Low-middle SDI
DALYs (Disability-Adjusted Life Years)	Both	1927 to 1936	1932	1.265208152	1.20515685	1.328251729	High-middle SDI
DALYs (Disability-Adjusted Life Years)	Both	1932 to 1941	1937	1.185932426	1.136155271	1.237890415	High-middle SDI
DALYs (Disability-Adjusted Life Years)	Both	1937 to 1946	1942	1.169991223	1.122339111	1.219666542	High-middle SDI
DALYs (Disability-Adjusted Life Years)	Both	1942 to 1951	1947	1.11977243	1.075748795	1.165597675	High-middle SDI
DALYs (Disability-Adjusted Life Years)	Both	1947 to 1956	1952	1.040930154	1.002093954	1.081271454	High-middle SDI
DALYs (Disability-Adjusted Life Years)	Both	1952 to 1961	1957	1.037623279	1.000699579	1.075909384	High-middle SDI
DALYs (Disability-Adjusted Life Years)	Both	1957 to 1966	1962	0.988284277	0.953623334	1.024205026	High-middle SDI
DALYs (Disability-Adjusted Life Years)	Both	1962 to 1971	1967	0.890347924	0.859160277	0.922667688	High-middle SDI
DALYs (Disability-Adjusted Life Years)	Both	1967 to 1976	1972	0.914313466	0.882167458	0.947630868	High-middle SDI
DALYs (Disability-Adjusted Life Years)	Both	1972 to 1981	1977	1	1	1	High-middle SDI
DALYs (Disability-Adjusted Life Years)	Both	1977 to 1986	1982	0.96663068	0.928235868	1.006613625	High-middle SDI
DALYs (Disability-Adjusted Life Years)	Both	1982 to 1991	1987	0.830827839	0.792752192	0.870732248	High-middle SDI
DALYs (Disability-Adjusted Life Years)	Both	1987 to 1996	1992	0.852052775	0.804744263	0.902142412	High-middle SDI
DALYs (Disability-Adjusted Life Years)	Both	1992 to 2001	1997	0.901158283	0.837725827	0.969393833	High-middle SDI
DALYs (Disability-Adjusted Life Years)	Both	1997 to 2006	2002	0.882780888	0.794966151	0.980295949	High-middle SDI
DALYs (Disability-Adjusted Life Years)	Both	1927 to 1936	1932	1.001277565	0.961708655	1.042474513	Low SDI
DALYs (Disability-Adjusted Life Years)	Both	1932 to 1941	1937	0.989217398	0.958014111	1.021437001	Low SDI
DALYs (Disability-Adjusted Life Years)	Both	1937 to 1946	1942	0.993058422	0.963869368	1.023131414	Low SDI
DALYs (Disability-Adjusted Life Years)	Both	1942 to 1951	1947	0.996365602	0.968639997	1.024884805	Low SDI
DALYs (Disability-Adjusted Life Years)	Both	1947 to 1956	1952	0.981173443	0.955786132	1.007235085	Low SDI

DALYs (Disability-Adjusted Life Years)	Both	1952 to 1961	1957	0.984856005	0.961177639	1.009117682	Low SDI
DALYs (Disability-Adjusted Life Years)	Both	1957 to 1966	1962	0.992879574	0.969845096	1.016461137	Low SDI
DALYs (Disability-Adjusted Life Years)	Both	1962 to 1971	1967	0.969527482	0.947663125	0.99189629	Low SDI
DALYs (Disability-Adjusted Life Years)	Both	1967 to 1976	1972	0.98984661	0.968609232	1.011549631	Low SDI
DALYs (Disability-Adjusted Life Years)	Both	1972 to 1981	1977	1	1	1	Low SDI
DALYs (Disability-Adjusted Life Years)	Both	1977 to 1986	1982	0.996719555	0.974602851	1.019338153	Low SDI
DALYs (Disability-Adjusted Life Years)	Both	1982 to 1991	1987	1.011222254	0.986917847	1.036125196	Low SDI
DALYs (Disability-Adjusted Life Years)	Both	1987 to 1996	1992	1.000024484	0.973897886	1.026851975	Low SDI
DALYs (Disability-Adjusted Life Years)	Both	1992 to 2001	1997	0.987010958	0.958334957	1.016545024	Low SDI
DALYs (Disability-Adjusted Life Years)	Both	1997 to 2006	2002	0.955826795	0.919897839	0.993159049	Low SDI
DALYs (Disability-Adjusted Life Years)	Both	1927 to 1936	1932	1.239382612	1.169548886	1.313386107	Middle SDI
DALYs (Disability-Adjusted Life Years)	Both	1932 to 1941	1937	1.237303375	1.180928714	1.296369225	Middle SDI
DALYs (Disability-Adjusted Life Years)	Both	1937 to 1946	1942	1.260258097	1.207456029	1.3153692	Middle SDI
DALYs (Disability-Adjusted Life Years)	Both	1942 to 1951	1947	1.211116482	1.163338474	1.260856721	Middle SDI
DALYs (Disability-Adjusted Life Years)	Both	1947 to 1956	1952	1.139044795	1.097187992	1.1824984	Middle SDI
DALYs (Disability-Adjusted Life Years)	Both	1952 to 1961	1957	1.16540565	1.125334997	1.206903129	Middle SDI
DALYs (Disability-Adjusted Life Years)	Both	1957 to 1966	1962	1.111556673	1.074478529	1.149914311	Middle SDI
DALYs (Disability-Adjusted Life Years)	Both	1962 to 1971	1967	0.999040177	0.966380957	1.032803127	Middle SDI
DALYs (Disability-Adjusted Life Years)	Both	1967 to 1976	1972	0.992322096	0.96017485	1.025545653	Middle SDI
DALYs (Disability-Adjusted Life Years)	Both	1972 to 1981	1977	1	1	1	Middle SDI
DALYs (Disability-Adjusted Life Years)	Both	1977 to 1986	1982	0.989453205	0.954560758	1.025621091	Middle SDI
DALYs (Disability-Adjusted Life Years)	Both	1982 to 1991	1987	0.895238191	0.859938928	0.931986438	Middle SDI
DALYs (Disability-Adjusted Life Years)	Both	1987 to 1996	1992	0.871381936	0.831461384	0.913219175	Middle SDI
DALYs (Disability-Adjusted Life Years)	Both	1992 to 2001	1997	0.855687945	0.808100694	0.906077503	Middle SDI

DALYs (Disability-Adjusted Life Years)	Both	1997 to 2006	2002	0.73904728	0.681718908	0.801196616	Middle SDI
DALYs (Disability-Adjusted Life Years)	Both	1927 to 1936	1932	1.506537791	1.441934412	1.574035613	Global
DALYs (Disability-Adjusted Life Years)	Both	1932 to 1941	1937	1.361736424	1.311047048	1.414385618	Global
DALYs (Disability-Adjusted Life Years)	Both	1937 to 1946	1942	1.310160257	1.263680573	1.358349519	Global
DALYs (Disability-Adjusted Life Years)	Both	1942 to 1951	1947	1.244317114	1.201957676	1.288169385	Global
DALYs (Disability-Adjusted Life Years)	Both	1947 to 1956	1952	1.171107	1.133404924	1.210063215	Global
DALYs (Disability-Adjusted Life Years)	Both	1952 to 1961	1957	1.14385986	1.108921451	1.179899062	Global
DALYs (Disability-Adjusted Life Years)	Both	1957 to 1966	1962	1.090470182	1.057739221	1.124213978	Global
DALYs (Disability-Adjusted Life Years)	Both	1962 to 1971	1967	0.988254885	0.958776394	1.018639721	Global
DALYs (Disability-Adjusted Life Years)	Both	1967 to 1976	1972	0.975564122	0.94654378	1.005474207	Global
DALYs (Disability-Adjusted Life Years)	Both	1972 to 1981	1977	1	1	1	Global
DALYs (Disability-Adjusted Life Years)	Both	1977 to 1986	1982	0.998034784	0.965519273	1.03164531	Global
DALYs (Disability-Adjusted Life Years)	Both	1982 to 1991	1987	0.95035143	0.91574806	0.986262358	Global
DALYs (Disability-Adjusted Life Years)	Both	1987 to 1996	1992	0.936779453	0.897358483	0.977932187	Global
DALYs (Disability-Adjusted Life Years)	Both	1992 to 2001	1997	0.930400078	0.883430918	0.979866437	Global
DALYs (Disability-Adjusted Life Years)	Both	1997 to 2006	2002	0.875771298	0.815162768	0.94088616	Global
Prevalence	Male	1927 to 1936	1932	0.815947729	0.798256249	0.834031299	High SDI
Prevalence	Male	1932 to 1941	1937	0.883776215	0.865702212	0.902227564	High SDI
Prevalence	Male	1937 to 1946	1942	0.922703981	0.903991352	0.941803961	High SDI
Prevalence	Male	1942 to 1951	1947	0.919367344	0.900955767	0.938155172	High SDI
Prevalence	Male	1947 to 1956	1952	0.922109407	0.904089169	0.940488823	High SDI
Prevalence	Male	1952 to 1961	1957	0.935240022	0.917519169	0.953303133	High SDI
Prevalence	Male	1957 to 1966	1962	0.95472988	0.936652749	0.973155895	High SDI
Prevalence	Male	1962 to 1971	1967	0.969106023	0.950497576	0.988078778	High SDI

Prevalence	Male	1967 to 1976	1972	0.985513425	0.965788894	1.005640795	High SDI
Prevalence	Male	1972 to 1981	1977	1	1	1	High SDI
Prevalence	Male	1977 to 1986	1982	1.010976129	0.984353232	1.038319071	High SDI
Prevalence	Male	1982 to 1991	1987	1.026606657	0.991258317	1.063215521	High SDI
Prevalence	Male	1987 to 1996	1992	1.088068023	1.033010973	1.146059486	High SDI
Prevalence	Male	1992 to 2001	1997	1.138306777	1.036059172	1.250645092	High SDI
Prevalence	Male	1997 to 2006	2002	1.183468455	0.909815585	1.539430196	High SDI
Prevalence	Male	1927 to 1936	1932	0.57758695	0.562027496	0.59357716	Low-middle SDI
Prevalence	Male	1932 to 1941	1937	0.629966276	0.614779042	0.64552869	Low-middle SDI
Prevalence	Male	1937 to 1946	1942	0.681553414	0.665534199	0.697958207	Low-middle SDI
Prevalence	Male	1942 to 1951	1947	0.742824928	0.725714079	0.760339215	Low-middle SDI
Prevalence	Male	1947 to 1956	1952	0.80016677	0.782421017	0.818315007	Low-middle SDI
Prevalence	Male	1952 to 1961	1957	0.846853593	0.828989195	0.865102961	Low-middle SDI
Prevalence	Male	1957 to 1966	1962	0.887042294	0.868553252	0.905924915	Low-middle SDI
Prevalence	Male	1962 to 1971	1967	0.917496718	0.898407586	0.936991451	Low-middle SDI
Prevalence	Male	1967 to 1976	1972	0.951193701	0.930905865	0.971923683	Low-middle SDI
Prevalence	Male	1972 to 1981	1977	1	1	1	Low-middle SDI
Prevalence	Male	1977 to 1986	1982	1.040822514	1.012781617	1.06963978	Low-middle SDI
Prevalence	Male	1982 to 1991	1987	1.068669268	1.032721974	1.105867827	Low-middle SDI
Prevalence	Male	1987 to 1996	1992	1.085755499	1.035502148	1.138447665	Low-middle SDI
Prevalence	Male	1992 to 2001	1997	1.105979884	1.023521131	1.195081827	Low-middle SDI
Prevalence	Male	1997 to 2006	2002	1.139985379	0.938926799	1.384097957	Low-middle SDI
Prevalence	Male	1927 to 1936	1932	0.656956927	0.647190899	0.666870324	High-middle SDI
Prevalence	Male	1932 to 1941	1937	0.709482977	0.699478455	0.719630591	High-middle SDI

Prevalence	Male	1937 to 1946	1942	0.736105789	0.72568499	0.746676231	High-middle SDI
Prevalence	Male	1942 to 1951	1947	0.752835135	0.742271458	0.763549149	High-middle SDI
Prevalence	Male	1947 to 1956	1952	0.771560563	0.761037836	0.782228785	High-middle SDI
Prevalence	Male	1952 to 1961	1957	0.815823009	0.805089716	0.826699396	High-middle SDI
Prevalence	Male	1957 to 1966	1962	0.812925971	0.802254823	0.823739061	High-middle SDI
Prevalence	Male	1962 to 1971	1967	0.790353935	0.779865974	0.800982942	High-middle SDI
Prevalence	Male	1967 to 1976	1972	0.873443782	0.861436889	0.88561803	High-middle SDI
Prevalence	Male	1972 to 1981	1977	1	1	1	High-middle SDI
Prevalence	Male	1977 to 1986	1982	1.027719821	1.008736643	1.047060238	High-middle SDI
Prevalence	Male	1982 to 1991	1987	0.983177293	0.95946566	1.007474919	High-middle SDI
Prevalence	Male	1987 to 1996	1992	1.040925803	1.002776631	1.080526304	High-middle SDI
Prevalence	Male	1992 to 2001	1997	1.143823656	1.067462797	1.225646982	High-middle SDI
Prevalence	Male	1997 to 2006	2002	1.270143174	1.050378092	1.535888549	High-middle SDI
Prevalence	Male	1927 to 1936	1932	0.784450501	0.743197159	0.827993732	Low SDI
Prevalence	Male	1932 to 1941	1937	0.815254762	0.77829681	0.853967687	Low SDI
Prevalence	Male	1937 to 1946	1942	0.845129773	0.808559688	0.883353875	Low SDI
Prevalence	Male	1942 to 1951	1947	0.874422799	0.837632913	0.912828543	Low SDI
Prevalence	Male	1947 to 1956	1952	0.894457551	0.858404563	0.932024764	Low SDI
Prevalence	Male	1952 to 1961	1957	0.906953561	0.872290408	0.942994162	Low SDI
Prevalence	Male	1957 to 1966	1962	0.92553474	0.89074466	0.961683626	Low SDI
Prevalence	Male	1962 to 1971	1967	0.950161555	0.914860101	0.986825176	Low SDI
Prevalence	Male	1967 to 1976	1972	0.978788724	0.942122351	1.016882111	Low SDI
Prevalence	Male	1972 to 1981	1977	1	1	1	Low SDI
Prevalence	Male	1977 to 1986	1982	1.017335123	0.970275476	1.066677225	Low SDI

Prevalence	Male	1982 to 1991	1987	1.039234172	0.980156776	1.101872365	Low SDI
Prevalence	Male	1987 to 1996	1992	1.059919048	0.980150743	1.146179195	Low SDI
Prevalence	Male	1992 to 2001	1997	1.077662025	0.955065023	1.215996201	Low SDI
Prevalence	Male	1997 to 2006	2002	1.096974321	0.830757603	1.448500329	Low SDI
Prevalence	Male	1927 to 1936	1932	0.503920645	0.494508132	0.513512316	Middle SDI
Prevalence	Male	1932 to 1941	1937	0.562151639	0.552555107	0.57191484	Middle SDI
Prevalence	Male	1937 to 1946	1942	0.633573684	0.622931711	0.644397462	Middle SDI
Prevalence	Male	1942 to 1951	1947	0.691472348	0.680059351	0.703076882	Middle SDI
Prevalence	Male	1947 to 1956	1952	0.737377018	0.725623941	0.749320462	Middle SDI
Prevalence	Male	1952 to 1961	1957	0.809382165	0.797051249	0.821903849	Middle SDI
Prevalence	Male	1957 to 1966	1962	0.856058555	0.843151677	0.86916301	Middle SDI
Prevalence	Male	1962 to 1971	1967	0.861132817	0.848155882	0.874308302	Middle SDI
Prevalence	Male	1967 to 1976	1972	0.911032927	0.89685643	0.925433511	Middle SDI
Prevalence	Male	1972 to 1981	1977	1	1	1	Middle SDI
Prevalence	Male	1977 to 1986	1982	1.075884308	1.053889521	1.098338128	Middle SDI
Prevalence	Male	1982 to 1991	1987	1.091270171	1.06312124	1.12016442	Middle SDI
Prevalence	Male	1987 to 1996	1992	1.138331672	1.096209534	1.182072363	Middle SDI
Prevalence	Male	1992 to 2001	1997	1.195484926	1.11973102	1.276363863	Middle SDI
Prevalence	Male	1997 to 2006	2002	1.224755707	1.03017489	1.456089209	Middle SDI
Prevalence	Male	1927 to 1936	1932	0.836817917	0.828238908	0.845485789	Global
Prevalence	Male	1932 to 1941	1937	0.89435701	0.885755412	0.903042138	Global
Prevalence	Male	1937 to 1946	1942	0.933733348	0.924807476	0.942745368	Global
Prevalence	Male	1942 to 1951	1947	0.96605081	0.956933071	0.975255423	Global
Prevalence	Male	1947 to 1956	1952	0.968671796	0.959783765	0.977642135	Global

Prevalence	Male	1952 to 1961	1957	0.990604587	0.981838325	0.999449118	Global
Prevalence	Male	1957 to 1966	1962	0.9848291	0.976140444	0.993595093	Global
Prevalence	Male	1962 to 1971	1967	0.946649314	0.938215691	0.955158748	Global
Prevalence	Male	1967 to 1976	1972	0.965568307	0.956640774	0.974579154	Global
Prevalence	Male	1972 to 1981	1977	1	1	1	Global
Prevalence	Male	1977 to 1986	1982	1.014643446	1.002283813	1.027155491	Global
Prevalence	Male	1982 to 1991	1987	0.995412792	0.979743262	1.011332932	Global
Prevalence	Male	1987 to 1996	1992	1.016167913	0.992766012	1.040121454	Global
Prevalence	Male	1992 to 2001	1997	1.031833028	0.990177387	1.075241074	Global
Prevalence	Male	1997 to 2006	2002	1.054990628	0.944504922	1.178400662	Global
Prevalence	Female	1927 to 1936	1932	0.78463725	0.757371777	0.812884283	High SDI
Prevalence	Female	1932 to 1941	1937	0.854416496	0.826007068	0.883803029	High SDI
Prevalence	Female	1937 to 1946	1942	0.888346597	0.858890476	0.918812932	High SDI
Prevalence	Female	1942 to 1951	1947	0.875332682	0.846564407	0.905078571	High SDI
Prevalence	Female	1947 to 1956	1952	0.864321203	0.836548116	0.893016347	High SDI
Prevalence	Female	1952 to 1961	1957	0.873871812	0.846615212	0.902005932	High SDI
Prevalence	Female	1957 to 1966	1962	0.898809911	0.870727976	0.927797519	High SDI
Prevalence	Female	1962 to 1971	1967	0.925265545	0.895888366	0.955606034	High SDI
Prevalence	Female	1967 to 1976	1972	0.958283528	0.92654285	0.99111155	High SDI
Prevalence	Female	1972 to 1981	1977	1	1	1	High SDI
Prevalence	Female	1977 to 1986	1982	1.039267187	0.994199727	1.086377573	High SDI
Prevalence	Female	1982 to 1991	1987	1.085408386	1.024071335	1.150419238	High SDI
Prevalence	Female	1987 to 1996	1992	1.1668341	1.070633806	1.271678336	High SDI
Prevalence	Female	1992 to 2001	1997	1.243094192	1.06314559	1.453500993	High SDI

Prevalence	Female	1997 to 2006	2002	1.334113316	0.863407516	2.061434847	High SDI
Prevalence	Female	1927 to 1936	1932	0.599591467	0.57827182	0.621697123	Low-middle SDI
Prevalence	Female	1932 to 1941	1937	0.634209084	0.613877604	0.655213938	Low-middle SDI
Prevalence	Female	1937 to 1946	1942	0.682974924	0.661517674	0.70512817	Low-middle SDI
Prevalence	Female	1942 to 1951	1947	0.742153486	0.719281801	0.765752443	Low-middle SDI
Prevalence	Female	1947 to 1956	1952	0.798993558	0.775323793	0.823385934	Low-middle SDI
Prevalence	Female	1952 to 1961	1957	0.850798437	0.826875534	0.875413469	Low-middle SDI
Prevalence	Female	1957 to 1966	1962	0.893495982	0.868673224	0.919028063	Low-middle SDI
Prevalence	Female	1962 to 1971	1967	0.922939161	0.897356543	0.949251111	Low-middle SDI
Prevalence	Female	1967 to 1976	1972	0.949741019	0.922770692	0.977499624	Low-middle SDI
Prevalence	Female	1972 to 1981	1977	1	1	1	Low-middle SDI
Prevalence	Female	1977 to 1986	1982	1.048731197	1.011111223	1.087750881	Low-middle SDI
Prevalence	Female	1982 to 1991	1987	1.076578958	1.028134826	1.127305703	Low-middle SDI
Prevalence	Female	1987 to 1996	1992	1.083621277	1.016104486	1.155624336	Low-middle SDI
Prevalence	Female	1992 to 2001	1997	1.086222425	0.975781082	1.209163796	Low-middle SDI
Prevalence	Female	1997 to 2006	2002	1.10756895	0.839788232	1.460736093	Low-middle SDI
Prevalence	Female	1927 to 1936	1932	0.660285322	0.64717826	0.673657836	High-middle SDI
Prevalence	Female	1932 to 1941	1937	0.721195904	0.707402102	0.735258674	High-middle SDI
Prevalence	Female	1937 to 1946	1942	0.761486712	0.746804885	0.776457176	High-middle SDI
Prevalence	Female	1942 to 1951	1947	0.774127834	0.75929373	0.789251748	High-middle SDI
Prevalence	Female	1947 to 1956	1952	0.785706747	0.771049924	0.80064218	High-middle SDI
Prevalence	Female	1952 to 1961	1957	0.824931538	0.810065537	0.840070355	High-middle SDI
Prevalence	Female	1957 to 1966	1962	0.813584873	0.798925636	0.828513088	High-middle SDI
Prevalence	Female	1962 to 1971	1967	0.801746988	0.787124648	0.816640965	High-middle SDI

Prevalence	Female	1967 to 1976	1972	0.878792402	0.862156119	0.8957497	High-middle SDI
Prevalence	Female	1972 to 1981	1977	1	1	1	High-middle SDI
Prevalence	Female	1977 to 1986	1982	1.035929524	1.009591535	1.062954612	High-middle SDI
Prevalence	Female	1982 to 1991	1987	0.999881683	0.966728031	1.034172331	High-middle SDI
Prevalence	Female	1987 to 1996	1992	1.085472913	1.030803418	1.143041849	High-middle SDI
Prevalence	Female	1992 to 2001	1997	1.221018053	1.109431337	1.343828171	High-middle SDI
Prevalence	Female	1997 to 2006	2002	1.353045502	1.037951854	1.763792918	High-middle SDI
Prevalence	Female	1927 to 1936	1932	0.725535509	0.670897588	0.784623144	Low SDI
Prevalence	Female	1932 to 1941	1937	0.761148257	0.709896142	0.816100602	Low SDI
Prevalence	Female	1937 to 1946	1942	0.791564849	0.73950732	0.847286962	Low SDI
Prevalence	Female	1942 to 1951	1947	0.828085952	0.774589167	0.885277477	Low SDI
Prevalence	Female	1947 to 1956	1952	0.864207872	0.81058924	0.921373254	Low SDI
Prevalence	Female	1952 to 1961	1957	0.888361584	0.836114074	0.943873962	Low SDI
Prevalence	Female	1957 to 1966	1962	0.913124677	0.860045745	0.969479449	Low SDI
Prevalence	Female	1962 to 1971	1967	0.94741493	0.892699786	1.005483661	Low SDI
Prevalence	Female	1967 to 1976	1972	0.981738633	0.924395901	1.042638488	Low SDI
Prevalence	Female	1972 to 1981	1977	1	1	1	Low SDI
Prevalence	Female	1977 to 1986	1982	1.021664163	0.948289513	1.100716234	Low SDI
Prevalence	Female	1982 to 1991	1987	1.042930967	0.950942842	1.143817433	Low SDI
Prevalence	Female	1987 to 1996	1992	1.059400654	0.935355901	1.199895938	Low SDI
Prevalence	Female	1992 to 2001	1997	1.065374617	0.8759614	1.295745536	Low SDI
Prevalence	Female	1997 to 2006	2002	1.061802825	0.666118305	1.692530038	Low SDI
Prevalence	Female	1927 to 1936	1932	0.510254681	0.49809108	0.522715324	Middle SDI
Prevalence	Female	1932 to 1941	1937	0.560020897	0.547813765	0.572500045	Middle SDI

Prevalence	Female	1937 to 1946	1942	0.625565166	0.612146361	0.639278123	Middle SDI
Prevalence	Female	1942 to 1951	1947	0.68353849	0.669166508	0.698219145	Middle SDI
Prevalence	Female	1947 to 1956	1952	0.726614708	0.711908532	0.741624675	Middle SDI
Prevalence	Female	1952 to 1961	1957	0.800838492	0.785395399	0.816585239	Middle SDI
Prevalence	Female	1957 to 1966	1962	0.844057411	0.827971518	0.860455823	Middle SDI
Prevalence	Female	1962 to 1971	1967	0.853149125	0.836922749	0.869690101	Middle SDI
Prevalence	Female	1967 to 1976	1972	0.908307959	0.890479562	0.926493301	Middle SDI
Prevalence	Female	1972 to 1981	1977	1	1	1	Middle SDI
Prevalence	Female	1977 to 1986	1982	1.077128145	1.04952249	1.105459914	Middle SDI
Prevalence	Female	1982 to 1991	1987	1.076406165	1.041379997	1.112610416	Middle SDI
Prevalence	Female	1987 to 1996	1992	1.119767818	1.067001186	1.17514393	Middle SDI
Prevalence	Female	1992 to 2001	1997	1.185148483	1.088763613	1.290066006	Middle SDI
Prevalence	Female	1997 to 2006	2002	1.219518342	0.970128495	1.533018557	Middle SDI
Prevalence	Female	1927 to 1936	1932	0.845669321	0.829671596	0.861975515	Global
Prevalence	Female	1932 to 1941	1937	0.891406078	0.875376441	0.907729245	Global
Prevalence	Female	1937 to 1946	1942	0.927281004	0.910625118	0.944241537	Global
Prevalence	Female	1942 to 1951	1947	0.948782186	0.931914576	0.9659551	Global
Prevalence	Female	1947 to 1956	1952	0.941960934	0.925672848	0.958535623	Global
Prevalence	Female	1952 to 1961	1957	0.961567121	0.945535298	0.977870767	Global
Prevalence	Female	1957 to 1966	1962	0.957344403	0.941410131	0.973548377	Global
Prevalence	Female	1962 to 1971	1967	0.933009232	0.917323228	0.948963464	Global
Prevalence	Female	1967 to 1976	1972	0.957412187	0.940703477	0.974417677	Global
Prevalence	Female	1972 to 1981	1977	1	1	1	Global
Prevalence	Female	1977 to 1986	1982	1.032554955	1.008922951	1.056740492	Global

Prevalence	Female	1982 to 1991	1987	1.024809404	0.994557557	1.055981432	Global
Prevalence	Female	1987 to 1996	1992	1.057065858	1.011527627	1.104654187	Global
Prevalence	Female	1992 to 2001	1997	1.079605109	0.998347374	1.167476593	Global
Prevalence	Female	1997 to 2006	2002	1.101890413	0.891377509	1.362119272	Global
Prevalence	Both	1927 to 1936	1932	0.797305812	0.778389565	0.816681757	High SDI
Prevalence	Both	1932 to 1941	1937	0.866237311	0.846751513	0.886171525	High SDI
Prevalence	Both	1937 to 1946	1942	0.904915412	0.88469024	0.925602959	High SDI
Prevalence	Both	1942 to 1951	1947	0.899992377	0.880105154	0.920328981	High SDI
Prevalence	Both	1947 to 1956	1952	0.8976763	0.878308259	0.917471436	High SDI
Prevalence	Both	1952 to 1961	1957	0.910343703	0.891293848	0.929800715	High SDI
Prevalence	Both	1957 to 1966	1962	0.932704703	0.913185738	0.952640878	High SDI
Prevalence	Both	1962 to 1971	1967	0.952315965	0.932092148	0.972978583	High SDI
Prevalence	Both	1967 to 1976	1972	0.975262416	0.953667916	0.997345894	High SDI
Prevalence	Both	1972 to 1981	1977	1	1	1	High SDI
Prevalence	Both	1977 to 1986	1982	1.019752654	0.990079612	1.050315007	High SDI
Prevalence	Both	1982 to 1991	1987	1.046206767	1.006428402	1.087557344	High SDI
Prevalence	Both	1987 to 1996	1992	1.111852064	1.049816578	1.177553335	High SDI
Prevalence	Both	1992 to 2001	1997	1.170179397	1.054455206	1.29860407	High SDI
Prevalence	Both	1997 to 2006	2002	1.227041496	0.91759654	1.640841881	High SDI
Prevalence	Both	1927 to 1936	1932	0.59638173	0.583521712	0.609525166	Low-middle SDI
Prevalence	Both	1932 to 1941	1937	0.639316108	0.626950806	0.65192529	Low-middle SDI
Prevalence	Both	1937 to 1946	1942	0.690267245	0.677227704	0.703557853	Low-middle SDI
Prevalence	Both	1942 to 1951	1947	0.750973721	0.737067023	0.765142804	Low-middle SDI
Prevalence	Both	1947 to 1956	1952	0.806854967	0.792479557	0.821491144	Low-middle SDI

Prevalence	Both	1952 to 1961	1957	0.854234095	0.839771299	0.868945974	Low-middle SDI
Prevalence	Both	1957 to 1966	1962	0.89379082	0.878839268	0.90899674	Low-middle SDI
Prevalence	Both	1962 to 1971	1967	0.921749911	0.906360871	0.937400242	Low-middle SDI
Prevalence	Both	1967 to 1976	1972	0.951106201	0.934830266	0.967665509	Low-middle SDI
Prevalence	Both	1972 to 1981	1977	1	1	1	Low-middle SDI
Prevalence	Both	1977 to 1986	1982	1.045766914	1.023140375	1.068893834	Low-middle SDI
Prevalence	Both	1982 to 1991	1987	1.075667229	1.046528393	1.105617388	Low-middle SDI
Prevalence	Both	1987 to 1996	1992	1.089810836	1.049013004	1.132195362	Low-middle SDI
Prevalence	Both	1992 to 2001	1997	1.103932194	1.036737792	1.175481686	Low-middle SDI
Prevalence	Both	1997 to 2006	2002	1.132239656	0.965918249	1.327199936	Low-middle SDI
Prevalence	Both	1927 to 1936	1932	0.65558491	0.64647709	0.664821045	High-middle SDI
Prevalence	Both	1932 to 1941	1937	0.711572704	0.702142881	0.721129171	High-middle SDI
Prevalence	Both	1937 to 1946	1942	0.744031615	0.734118113	0.754078989	High-middle SDI
Prevalence	Both	1942 to 1951	1947	0.760818726	0.750761661	0.771010513	High-middle SDI
Prevalence	Both	1947 to 1956	1952	0.77533716	0.765371068	0.785433023	High-middle SDI
Prevalence	Both	1952 to 1961	1957	0.818464628	0.80831193	0.828744846	High-middle SDI
Prevalence	Both	1957 to 1966	1962	0.812846961	0.802779086	0.8230411	High-middle SDI
Prevalence	Both	1962 to 1971	1967	0.794053549	0.78410689	0.804126385	High-middle SDI
Prevalence	Both	1967 to 1976	1972	0.875258412	0.863893335	0.886773003	High-middle SDI
Prevalence	Both	1972 to 1981	1977	1	1	1	High-middle SDI
Prevalence	Both	1977 to 1986	1982	1.031644476	1.01364033	1.049968409	High-middle SDI
Prevalence	Both	1982 to 1991	1987	0.990867192	0.968286007	1.013974987	High-middle SDI
Prevalence	Both	1987 to 1996	1992	1.062563461	1.025728239	1.100721483	High-middle SDI
Prevalence	Both	1992 to 2001	1997	1.181724813	1.106995542	1.261498787	High-middle SDI

Prevalence	Both	1997 to 2006	2002	1.305131082	1.089850141	1.562937029	High-middle SDI
Prevalence	Both	1927 to 1936	1932	0.776884224	0.743212816	0.812081122	Low SDI
Prevalence	Both	1932 to 1941	1937	0.805185606	0.774753236	0.836813361	Low SDI
Prevalence	Both	1937 to 1946	1942	0.834028603	0.803700142	0.865501539	Low SDI
Prevalence	Both	1942 to 1951	1947	0.86691832	0.836162932	0.898804939	Low SDI
Prevalence	Both	1947 to 1956	1952	0.890541094	0.860260729	0.921887298	Low SDI
Prevalence	Both	1952 to 1961	1957	0.906993547	0.877769219	0.937190866	Low SDI
Prevalence	Both	1957 to 1966	1962	0.926289765	0.896883221	0.956660477	Low SDI
Prevalence	Both	1962 to 1971	1967	0.953452972	0.923483651	0.984394872	Low SDI
Prevalence	Both	1967 to 1976	1972	0.983159457	0.951968744	1.015372116	Low SDI
Prevalence	Both	1972 to 1981	1977	1	1	1	Low SDI
Prevalence	Both	1977 to 1986	1982	1.016389525	0.976566073	1.057836939	Low SDI
Prevalence	Both	1982 to 1991	1987	1.039683253	0.989540043	1.092367383	Low SDI
Prevalence	Both	1987 to 1996	1992	1.061387357	0.993337492	1.134099067	Low SDI
Prevalence	Both	1992 to 2001	1997	1.07571963	0.970588715	1.192237973	Low SDI
Prevalence	Both	1997 to 2006	2002	1.086079456	0.855412782	1.378946644	Low SDI
Prevalence	Both	1927 to 1936	1932	0.512232068	0.503596127	0.521016104	Middle SDI
Prevalence	Both	1932 to 1941	1937	0.567252474	0.55851237	0.576129351	Middle SDI
Prevalence	Both	1937 to 1946	1942	0.636661561	0.627009512	0.646462192	Middle SDI
Prevalence	Both	1942 to 1951	1947	0.694118178	0.683787278	0.704605162	Middle SDI
Prevalence	Both	1947 to 1956	1952	0.737566622	0.726978806	0.748308641	Middle SDI
Prevalence	Both	1952 to 1961	1957	0.80966285	0.798567451	0.820912409	Middle SDI
Prevalence	Both	1957 to 1966	1962	0.852568011	0.841011751	0.864283066	Middle SDI
Prevalence	Both	1962 to 1971	1967	0.858147619	0.84652805	0.869926679	Middle SDI

Prevalence	Both	1967 to 1976	1972	0.910752469	0.8980207	0.923664745	Middle SDI
Prevalence	Both	1972 to 1981	1977	1	1	1	Middle SDI
Prevalence	Both	1977 to 1986	1982	1.076184771	1.05645665	1.096281292	Middle SDI
Prevalence	Both	1982 to 1991	1987	1.084905298	1.059737448	1.110670864	Middle SDI
Prevalence	Both	1987 to 1996	1992	1.132327059	1.09446728	1.171496483	Middle SDI
Prevalence	Both	1992 to 2001	1997	1.194528993	1.125746502	1.267514056	Middle SDI
Prevalence	Both	1997 to 2006	2002	1.2250634	1.046083225	1.434666285	Middle SDI
Prevalence	Both	1927 to 1936	1932	0.846944538	0.836811152	0.857200634	Global
Prevalence	Both	1932 to 1941	1937	0.899225596	0.889084299	0.909482569	Global
Prevalence	Both	1937 to 1946	1942	0.938127592	0.927593617	0.948781193	Global
Prevalence	Both	1942 to 1951	1947	0.96713322	0.956402408	0.977984432	Global
Prevalence	Both	1947 to 1956	1952	0.964327521	0.953924142	0.974844358	Global
Prevalence	Both	1952 to 1961	1957	0.984909641	0.974662991	0.995264014	Global
Prevalence	Both	1957 to 1966	1962	0.977939975	0.967792048	0.988194309	Global
Prevalence	Both	1962 to 1971	1967	0.943311976	0.933426425	0.953302221	Global
Prevalence	Both	1967 to 1976	1972	0.963856476	0.953372542	0.974455698	Global
Prevalence	Both	1972 to 1981	1977	1	1	1	Global
Prevalence	Both	1977 to 1986	1982	1.020641509	1.006034627	1.035460472	Global
Prevalence	Both	1982 to 1991	1987	1.005700755	0.987108683	1.024643005	Global
Prevalence	Both	1987 to 1996	1992	1.031408645	1.003524566	1.060067516	Global
Prevalence	Both	1992 to 2001	1997	1.050207971	1.00044165	1.102449885	Global
Prevalence	Both	1997 to 2006	2002	1.072391469	0.940996533	1.222133581	Global
Incidence	Male	1927 to 1936	1932	0.773788864	0.751022713	0.797245138	High SDI
Incidence	Male	1932 to 1941	1937	0.893354715	0.868571759	0.918844802	High SDI

Incidence	Male	1937 to 1946	1942	0.950046714	0.923951775	0.976878646	High SDI
Incidence	Male	1942 to 1951	1947	0.967452311	0.941147206	0.994492645	High SDI
Incidence	Male	1947 to 1956	1952	0.97928821	0.953200879	1.006089503	High SDI
Incidence	Male	1952 to 1961	1957	0.991368478	0.965701351	1.017717806	High SDI
Incidence	Male	1957 to 1966	1962	0.998813927	0.972969647	1.02534469	High SDI
Incidence	Male	1962 to 1971	1967	0.9910958	0.965171997	1.017715898	High SDI
Incidence	Male	1967 to 1976	1972	0.982299589	0.955517433	1.009832421	High SDI
Incidence	Male	1972 to 1981	1977	1	1	1	High SDI
Incidence	Male	1977 to 1986	1982	1.015038851	0.979907609	1.051429604	High SDI
Incidence	Male	1982 to 1991	1987	1.023888329	0.981746347	1.067839278	High SDI
Incidence	Male	1987 to 1996	1992	1.077076361	1.020187894	1.137137084	High SDI
Incidence	Male	1992 to 2001	1997	1.118541396	1.032196237	1.21210949	High SDI
Incidence	Male	1997 to 2006	2002	1.159154597	0.988899047	1.358722495	High SDI
Incidence	Male	1927 to 1936	1932	0.580198939	0.537405844	0.626399605	Low-middle SDI
Incidence	Male	1932 to 1941	1937	0.626642928	0.587265509	0.668660687	Low-middle SDI
Incidence	Male	1937 to 1946	1942	0.678558082	0.638082042	0.721601674	Low-middle SDI
Incidence	Male	1942 to 1951	1947	0.739163925	0.696504253	0.784436428	Low-middle SDI
Incidence	Male	1947 to 1956	1952	0.796444996	0.752303121	0.843176925	Low-middle SDI
Incidence	Male	1952 to 1961	1957	0.847456451	0.802744838	0.89465843	Low-middle SDI
Incidence	Male	1957 to 1966	1962	0.893942566	0.847603061	0.94281551	Low-middle SDI
Incidence	Male	1962 to 1971	1967	0.924415505	0.877006042	0.974387843	Low-middle SDI
Incidence	Male	1967 to 1976	1972	0.956127024	0.906504242	1.008466196	Low-middle SDI
Incidence	Male	1972 to 1981	1977	1	1	1	Low-middle SDI
Incidence	Male	1977 to 1986	1982	1.029067992	0.961845825	1.10098823	Low-middle SDI

Incidence	Male	1982 to 1991	1987	1.050793235	0.97208737	1.135871586	Low-middle SDI
Incidence	Male	1987 to 1996	1992	1.065066234	0.967459952	1.172519939	Low-middle SDI
Incidence	Male	1992 to 2001	1997	1.082838939	0.948416922	1.236312998	Low-middle SDI
Incidence	Male	1997 to 2006	2002	1.121254541	0.879419351	1.429592999	Low-middle SDI
Incidence	Male	1927 to 1936	1932	0.619647648	0.596494497	0.643699496	High-middle SDI
Incidence	Male	1932 to 1941	1937	0.685175651	0.661427695	0.709776256	High-middle SDI
Incidence	Male	1937 to 1946	1942	0.727331551	0.702246604	0.753312557	High-middle SDI
Incidence	Male	1942 to 1951	1947	0.756122839	0.730320651	0.782836617	High-middle SDI
Incidence	Male	1947 to 1956	1952	0.77955412	0.753644604	0.806354379	High-middle SDI
Incidence	Male	1952 to 1961	1957	0.829062292	0.802427539	0.856581125	High-middle SDI
Incidence	Male	1957 to 1966	1962	0.824678275	0.798310171	0.851917315	High-middle SDI
Incidence	Male	1962 to 1971	1967	0.798766797	0.773072548	0.825315034	High-middle SDI
Incidence	Male	1967 to 1976	1972	0.877554321	0.848474946	0.90763032	High-middle SDI
Incidence	Male	1972 to 1981	1977	1	1	1	High-middle SDI
Incidence	Male	1977 to 1986	1982	1.022604055	0.976462447	1.070926031	High-middle SDI
Incidence	Male	1982 to 1991	1987	0.976116802	0.923694776	1.031513912	High-middle SDI
Incidence	Male	1987 to 1996	1992	1.029518628	0.956178545	1.108483987	High-middle SDI
Incidence	Male	1992 to 2001	1997	1.133947104	1.013457209	1.268762039	High-middle SDI
Incidence	Male	1997 to 2006	2002	1.266936692	1.016680765	1.578793105	High-middle SDI
Incidence	Male	1927 to 1936	1932	0.819754727	0.703055026	0.955825343	Low SDI
Incidence	Male	1932 to 1941	1937	0.853354558	0.753269834	0.966737241	Low SDI
Incidence	Male	1937 to 1946	1942	0.879926453	0.785393774	0.985837409	Low SDI
Incidence	Male	1942 to 1951	1947	0.907622109	0.815367503	1.010314845	Low SDI
Incidence	Male	1947 to 1956	1952	0.926825626	0.837353495	1.025857951	Low SDI

Incidence	Male	1952 to 1961	1957	0.936403538	0.850523756	1.030954844	Low SDI
Incidence	Male	1957 to 1966	1962	0.950174933	0.865012643	1.043721627	Low SDI
Incidence	Male	1962 to 1971	1967	0.969454244	0.884771282	1.062242356	Low SDI
Incidence	Male	1967 to 1976	1972	0.988325732	0.902761521	1.081999763	Low SDI
Incidence	Male	1972 to 1981	1977	1	1	1	Low SDI
Incidence	Male	1977 to 1986	1982	1.00862886	0.904263124	1.125039992	Low SDI
Incidence	Male	1982 to 1991	1987	1.024064769	0.90453363	1.159391556	Low SDI
Incidence	Male	1987 to 1996	1992	1.032944823	0.889700549	1.199251826	Low SDI
Incidence	Male	1992 to 2001	1997	1.050992116	0.862896146	1.280089654	Low SDI
Incidence	Male	1997 to 2006	2002	1.063011421	0.756295467	1.494116163	Low SDI
Incidence	Male	1927 to 1936	1932	0.488867196	0.463751115	0.515343527	Middle SDI
Incidence	Male	1932 to 1941	1937	0.545503203	0.521183155	0.5709581	Middle SDI
Incidence	Male	1937 to 1946	1942	0.620029616	0.59343322	0.647818005	Middle SDI
Incidence	Male	1942 to 1951	1947	0.683092856	0.6545501	0.712880267	Middle SDI
Incidence	Male	1947 to 1956	1952	0.733638705	0.704063002	0.764456801	Middle SDI
Incidence	Male	1952 to 1961	1957	0.811637532	0.780350233	0.844179261	Middle SDI
Incidence	Male	1957 to 1966	1962	0.865797131	0.832948494	0.899941207	Middle SDI
Incidence	Male	1962 to 1971	1967	0.872010607	0.839173605	0.906132526	Middle SDI
Incidence	Male	1967 to 1976	1972	0.916345129	0.881057331	0.953046262	Middle SDI
Incidence	Male	1972 to 1981	1977	1	1	1	Middle SDI
Incidence	Male	1977 to 1986	1982	1.062008011	1.007217255	1.119779283	Middle SDI
Incidence	Male	1982 to 1991	1987	1.067323369	1.00354299	1.135157324	Middle SDI
Incidence	Male	1987 to 1996	1992	1.107548813	1.023640218	1.198335462	Middle SDI
Incidence	Male	1992 to 2001	1997	1.165151806	1.039561986	1.305914174	Middle SDI

Incidence	Male	1997 to 2006	2002	1.201079876	0.965470287	1.494186706	Middle SDI
Incidence	Male	1927 to 1936	1932	0.775771899	0.760572508	0.791275036	Global
Incidence	Male	1932 to 1941	1937	0.860793494	0.845206685	0.876667746	Global
Incidence	Male	1937 to 1946	1942	0.912720337	0.896406784	0.929330777	Global
Incidence	Male	1942 to 1951	1947	0.956758625	0.939889089	0.973930942	Global
Incidence	Male	1947 to 1956	1952	0.963841417	0.947287576	0.980684537	Global
Incidence	Male	1952 to 1961	1957	0.988777296	0.972386689	1.005444184	Global
Incidence	Male	1957 to 1966	1962	0.984681354	0.968431932	1.001203428	Global
Incidence	Male	1962 to 1971	1967	0.946055558	0.930369815	0.962005758	Global
Incidence	Male	1967 to 1976	1972	0.963638624	0.94714834	0.980416011	Global
Incidence	Male	1972 to 1981	1977	1	1	1	Global
Incidence	Male	1977 to 1986	1982	1.010544597	0.987976574	1.033628133	Global
Incidence	Male	1982 to 1991	1987	0.990305966	0.964252175	1.017063722	Global
Incidence	Male	1987 to 1996	1992	1.010210736	0.976158926	1.04545039	Global
Incidence	Male	1992 to 2001	1997	1.028946246	0.978844387	1.081612553	Global
Incidence	Male	1997 to 2006	2002	1.054070209	0.957606189	1.160251489	Global
Incidence	Female	1927 to 1936	1932	0.760879505	0.728443583	0.794759724	High SDI
Incidence	Female	1932 to 1941	1937	0.896527975	0.859602725	0.935039393	High SDI
Incidence	Female	1937 to 1946	1942	0.960587503	0.921130419	1.001734751	High SDI
Incidence	Female	1942 to 1951	1947	0.953228616	0.914305655	0.993808568	High SDI
Incidence	Female	1947 to 1956	1952	0.933567441	0.896112225	0.97258819	High SDI
Incidence	Female	1952 to 1961	1957	0.926142219	0.889967648	0.96378718	High SDI
Incidence	Female	1957 to 1966	1962	0.935119645	0.898464323	0.973270422	High SDI
Incidence	Female	1962 to 1971	1967	0.940789426	0.903428944	0.979694917	High SDI

Incidence	Female	1967 to 1976	1972	0.96013675	0.920338529	1.001655965	High SDI
Incidence	Female	1972 to 1981	1977	1	1	1	High SDI
Incidence	Female	1977 to 1986	1982	1.044542828	0.989384827	1.102775875	High SDI
Incidence	Female	1982 to 1991	1987	1.087394493	1.01928945	1.160050056	High SDI
Incidence	Female	1987 to 1996	1992	1.167731669	1.074457581	1.269102916	High SDI
Incidence	Female	1992 to 2001	1997	1.242155786	1.098196196	1.404986653	High SDI
Incidence	Female	1997 to 2006	2002	1.324811335	1.039442267	1.688525788	High SDI
Incidence	Female	1927 to 1936	1932	0.606914015	0.548895569	0.671065029	Low-middle SDI
Incidence	Female	1932 to 1941	1937	0.633084851	0.579964388	0.691070757	Low-middle SDI
Incidence	Female	1937 to 1946	1942	0.676946953	0.622254723	0.736446283	Low-middle SDI
Incidence	Female	1942 to 1951	1947	0.732671187	0.675080773	0.795174577	Low-middle SDI
Incidence	Female	1947 to 1956	1952	0.786015092	0.726651444	0.850228441	Low-middle SDI
Incidence	Female	1952 to 1961	1957	0.84127134	0.780936672	0.906267425	Low-middle SDI
Incidence	Female	1957 to 1966	1962	0.888307069	0.825557635	0.955825996	Low-middle SDI
Incidence	Female	1962 to 1971	1967	0.923670479	0.859120108	0.993070871	Low-middle SDI
Incidence	Female	1967 to 1976	1972	0.953667176	0.886250372	1.026212356	Low-middle SDI
Incidence	Female	1972 to 1981	1977	1	1	1	Low-middle SDI
Incidence	Female	1977 to 1986	1982	1.036760753	0.944219563	1.138371731	Low-middle SDI
Incidence	Female	1982 to 1991	1987	1.060336349	0.951618418	1.181474792	Low-middle SDI
Incidence	Female	1987 to 1996	1992	1.069278646	0.935092694	1.222720302	Low-middle SDI
Incidence	Female	1992 to 2001	1997	1.071011912	0.888424587	1.291124235	Low-middle SDI
Incidence	Female	1997 to 2006	2002	1.096411532	0.773905106	1.553314792	Low-middle SDI
Incidence	Female	1927 to 1936	1932	0.59380219	0.561625287	0.627822587	High-middle SDI
Incidence	Female	1932 to 1941	1937	0.689709079	0.653908006	0.72747024	High-middle SDI

Incidence	Female	1937 to 1946	1942	0.754615087	0.71531494	0.796074425	High-middle SDI
Incidence	Female	1942 to 1951	1947	0.779115739	0.73875735	0.821678912	High-middle SDI
Incidence	Female	1947 to 1956	1952	0.789117195	0.74917341	0.831190668	High-middle SDI
Incidence	Female	1952 to 1961	1957	0.828870623	0.788252916	0.871581311	High-middle SDI
Incidence	Female	1957 to 1966	1962	0.819605331	0.779465081	0.861812691	High-middle SDI
Incidence	Female	1962 to 1971	1967	0.81156105	0.771533014	0.853665786	High-middle SDI
Incidence	Female	1967 to 1976	1972	0.88521447	0.84007727	0.932776883	High-middle SDI
Incidence	Female	1972 to 1981	1977	1	1	1	High-middle SDI
Incidence	Female	1977 to 1986	1982	1.033842278	0.962374359	1.110617552	High-middle SDI
Incidence	Female	1982 to 1991	1987	0.995405834	0.913720765	1.084393407	High-middle SDI
Incidence	Female	1987 to 1996	1992	1.078357333	0.961499873	1.209417256	High-middle SDI
Incidence	Female	1992 to 2001	1997	1.21510175	1.020611905	1.446653969	High-middle SDI
Incidence	Female	1997 to 2006	2002	1.342542757	0.951992571	1.8933142	High-middle SDI
Incidence	Female	1927 to 1936	1932	0.750365108	0.610124832	0.922840319	Low SDI
Incidence	Female	1932 to 1941	1937	0.783140063	0.654627004	0.936882154	Low SDI
Incidence	Female	1937 to 1946	1942	0.811329715	0.683598669	0.962927425	Low SDI
Incidence	Female	1942 to 1951	1947	0.842024612	0.713009937	0.994383684	Low SDI
Incidence	Female	1947 to 1956	1952	0.877065226	0.748215002	1.028104767	Low SDI
Incidence	Female	1952 to 1961	1957	0.900504339	0.774791899	1.046614021	Low SDI
Incidence	Female	1957 to 1966	1962	0.92919974	0.801328916	1.077475353	Low SDI
Incidence	Female	1962 to 1971	1967	0.960097467	0.830054964	1.110513382	Low SDI
Incidence	Female	1967 to 1976	1972	0.988683093	0.854962487	1.143318302	Low SDI
Incidence	Female	1972 to 1981	1977	1	1	1	Low SDI
Incidence	Female	1977 to 1986	1982	1.005852107	0.842430356	1.20097579	Low SDI

Incidence	Female	1982 to 1991	1987	1.017840826	0.831696378	1.245646817	Low SDI
Incidence	Female	1987 to 1996	1992	1.025255336	0.803498282	1.308214999	Low SDI
Incidence	Female	1992 to 2001	1997	1.027142037	0.741028373	1.423725195	Low SDI
Incidence	Female	1997 to 2006	2002	1.050900103	0.593997916	1.859250677	Low SDI
Incidence	Female	1927 to 1936	1932	0.493714478	0.461225941	0.528491493	Middle SDI
Incidence	Female	1932 to 1941	1937	0.539737351	0.508335872	0.573078597	Middle SDI
Incidence	Female	1937 to 1946	1942	0.604591925	0.570577962	0.640633568	Middle SDI
Incidence	Female	1942 to 1951	1947	0.665019414	0.62860166	0.703547013	Middle SDI
Incidence	Female	1947 to 1956	1952	0.710911482	0.673432513	0.750476292	Middle SDI
Incidence	Female	1952 to 1961	1957	0.789650668	0.749970852	0.831429883	Middle SDI
Incidence	Female	1957 to 1966	1962	0.836559067	0.795165269	0.880107695	Middle SDI
Incidence	Female	1962 to 1971	1967	0.853459383	0.811620306	0.897455266	Middle SDI
Incidence	Female	1967 to 1976	1972	0.912653842	0.866975333	0.960739025	Middle SDI
Incidence	Female	1972 to 1981	1977	1	1	1	Middle SDI
Incidence	Female	1977 to 1986	1982	1.065138526	0.994254065	1.141076632	Middle SDI
Incidence	Female	1982 to 1991	1987	1.060083338	0.978120045	1.14891489	Middle SDI
Incidence	Female	1987 to 1996	1992	1.100241898	0.99194999	1.220356114	Middle SDI
Incidence	Female	1992 to 2001	1997	1.168951406	1.005272089	1.359281139	Middle SDI
Incidence	Female	1997 to 2006	2002	1.208507749	0.90287982	1.617591786	Middle SDI
Incidence	Female	1927 to 1936	1932	0.789581769	0.766978287	0.812851394	Global
Incidence	Female	1932 to 1941	1937	0.876127817	0.852281052	0.900641813	Global
Incidence	Female	1937 to 1946	1942	0.930424686	0.905207728	0.95634413	Global
Incidence	Female	1942 to 1951	1947	0.953186007	0.927591614	0.979486609	Global
Incidence	Female	1947 to 1956	1952	0.937478248	0.912913858	0.962703607	Global

Incidence	Female	1952 to 1961	1957	0.950023082	0.925999685	0.974669723	Global
Incidence	Female	1957 to 1966	1962	0.947895604	0.92394674	0.972465227	Global
Incidence	Female	1962 to 1971	1967	0.928448127	0.904873809	0.952636617	Global
Incidence	Female	1967 to 1976	1972	0.959323478	0.93416994	0.985154302	Global
Incidence	Female	1972 to 1981	1977	1	1	1	Global
Incidence	Female	1977 to 1986	1982	1.028036008	0.992794746	1.06452823	Global
Incidence	Female	1982 to 1991	1987	1.021148565	0.97996188	1.06406628	Global
Incidence	Female	1987 to 1996	1992	1.05673949	1.002240045	1.114202486	Global
Incidence	Female	1992 to 2001	1997	1.086291158	1.005509226	1.173563056	Global
Incidence	Female	1997 to 2006	2002	1.114359158	0.959789898	1.293821007	Global
Incidence	Both	1927 to 1936	1932	0.7634989	0.743693546	0.783831692	High SDI
Incidence	Both	1932 to 1941	1937	0.887713982	0.86579078	0.910192314	High SDI
Incidence	Both	1937 to 1946	1942	0.947629182	0.924394157	0.97144823	High SDI
Incidence	Both	1942 to 1951	1947	0.957285648	0.934016383	0.981134622	High SDI
Incidence	Both	1947 to 1956	1952	0.957509619	0.9346859	0.980890662	High SDI
Incidence	Both	1952 to 1961	1957	0.963842654	0.941503473	0.986711879	High SDI
Incidence	Both	1957 to 1966	1962	0.974062978	0.951467595	0.997194953	High SDI
Incidence	Both	1962 to 1971	1967	0.9722795	0.949463774	0.995643491	High SDI
Incidence	Both	1967 to 1976	1972	0.973570211	0.94974235	0.997995884	High SDI
Incidence	Both	1972 to 1981	1977	1	1	1	High SDI
Incidence	Both	1977 to 1986	1982	1.024354976	0.992466073	1.057268501	High SDI
Incidence	Both	1982 to 1991	1987	1.045303667	1.006604499	1.085490634	High SDI
Incidence	Both	1987 to 1996	1992	1.104491333	1.052025846	1.159573322	High SDI
Incidence	Both	1992 to 2001	1997	1.156382971	1.076017625	1.242750625	High SDI

Incidence	Both	1997 to 2006	2002	1.208480984	1.048239126	1.39321864	High SDI
Incidence	Both	1927 to 1936	1932	0.601620661	0.566103851	0.639365761	Low-middle SDI
Incidence	Both	1932 to 1941	1937	0.637622997	0.605238172	0.671740655	Low-middle SDI
Incidence	Both	1937 to 1946	1942	0.687009378	0.653724573	0.721988901	Low-middle SDI
Incidence	Both	1942 to 1951	1947	0.745984938	0.710955437	0.782740377	Low-middle SDI
Incidence	Both	1947 to 1956	1952	0.800783093	0.764678762	0.83859209	Low-middle SDI
Incidence	Both	1952 to 1961	1957	0.852423649	0.81588821	0.890595144	Low-middle SDI
Incidence	Both	1957 to 1966	1962	0.89736125	0.85953983	0.936846886	Low-middle SDI
Incidence	Both	1962 to 1971	1967	0.927462889	0.888793644	0.967814538	Low-middle SDI
Incidence	Both	1967 to 1976	1972	0.956283603	0.915934473	0.99841021	Low-middle SDI
Incidence	Both	1972 to 1981	1977	1	1	1	Low-middle SDI
Incidence	Both	1977 to 1986	1982	1.034011361	0.978904293	1.092220662	Low-middle SDI
Incidence	Both	1982 to 1991	1987	1.058850225	0.994006784	1.127923689	Low-middle SDI
Incidence	Both	1987 to 1996	1992	1.072026209	0.991459583	1.15913973	Low-middle SDI
Incidence	Both	1992 to 2001	1997	1.087022327	0.975680481	1.211070184	Low-middle SDI
Incidence	Both	1997 to 2006	2002	1.114890394	0.913489414	1.360695123	Low-middle SDI
Incidence	Both	1927 to 1936	1932	0.607125955	0.588423493	0.626422857	High-middle SDI
Incidence	Both	1932 to 1941	1937	0.684878846	0.665063313	0.705284781	High-middle SDI
Incidence	Both	1937 to 1946	1942	0.736247341	0.714980085	0.758147196	High-middle SDI
Incidence	Both	1942 to 1951	1947	0.764637832	0.742741791	0.787179368	High-middle SDI
Incidence	Both	1947 to 1956	1952	0.781745046	0.759920781	0.804196084	High-middle SDI
Incidence	Both	1952 to 1961	1957	0.828223872	0.805870347	0.851197447	High-middle SDI
Incidence	Both	1957 to 1966	1962	0.82253713	0.800405363	0.845280855	High-middle SDI
Incidence	Both	1962 to 1971	1967	0.802776805	0.781036816	0.825121922	High-middle SDI

Incidence	Both	1967 to 1976	1972	0.879799226	0.855223079	0.905081605	High-middle SDI
Incidence	Both	1972 to 1981	1977	1	1	1	High-middle SDI
Incidence	Both	1977 to 1986	1982	1.026578462	0.987501894	1.067201335	High-middle SDI
Incidence	Both	1982 to 1991	1987	0.984507948	0.939882418	1.031252294	High-middle SDI
Incidence	Both	1987 to 1996	1992	1.052149466	0.988779511	1.119580742	High-middle SDI
Incidence	Both	1992 to 2001	1997	1.172039345	1.06642358	1.288115016	High-middle SDI
Incidence	Both	1997 to 2006	2002	1.305090649	1.084328501	1.570798518	High-middle SDI
Incidence	Both	1927 to 1936	1932	0.805668038	0.713094055	0.910259989	Low SDI
Incidence	Both	1932 to 1941	1937	0.83326507	0.752747916	0.922394686	Low SDI
Incidence	Both	1937 to 1946	1942	0.859659847	0.782285078	0.944687652	Low SDI
Incidence	Both	1942 to 1951	1947	0.890107142	0.813569879	0.973844712	Low SDI
Incidence	Both	1947 to 1956	1952	0.914015307	0.83918921	0.995513255	Low SDI
Incidence	Both	1952 to 1961	1957	0.929328543	0.857128326	1.007610547	Low SDI
Incidence	Both	1957 to 1966	1962	0.94673105	0.874609371	1.024799997	Low SDI
Incidence	Both	1962 to 1971	1967	0.969213612	0.897038426	1.04719597	Low SDI
Incidence	Both	1967 to 1976	1972	0.989893537	0.916671867	1.068963989	Low SDI
Incidence	Both	1972 to 1981	1977	1	1	1	Low SDI
Incidence	Both	1977 to 1986	1982	1.006674165	0.917325776	1.10472517	Low SDI
Incidence	Both	1982 to 1991	1987	1.019658756	0.917313874	1.133422276	Low SDI
Incidence	Both	1987 to 1996	1992	1.038805963	0.914801644	1.179619468	Low SDI
Incidence	Both	1992 to 2001	1997	1.044919517	0.882673867	1.236987791	Low SDI
Incidence	Both	1997 to 2006	2002	1.05387897	0.786896483	1.411444718	Low SDI
Incidence	Both	1927 to 1936	1932	0.497060046	0.476766729	0.518217138	Middle SDI
Incidence	Both	1932 to 1941	1937	0.549668738	0.530077319	0.569984247	Middle SDI

Incidence	Both	1937 to 1946	1942	0.621071418	0.599740166	0.643161369	Middle SDI
Incidence	Both	1942 to 1951	1947	0.682816454	0.659981695	0.706441275	Middle SDI
Incidence	Both	1947 to 1956	1952	0.730244251	0.706707019	0.754565403	Middle SDI
Incidence	Both	1952 to 1961	1957	0.80790058	0.783036951	0.833553698	Middle SDI
Incidence	Both	1957 to 1966	1962	0.856697588	0.830745351	0.883460565	Middle SDI
Incidence	Both	1962 to 1971	1967	0.865692142	0.839681569	0.892508437	Middle SDI
Incidence	Both	1967 to 1976	1972	0.916065997	0.88793071	0.945092789	Middle SDI
Incidence	Both	1972 to 1981	1977	1	1	1	Middle SDI
Incidence	Both	1977 to 1986	1982	1.063715467	1.019984497	1.109321364	Middle SDI
Incidence	Both	1982 to 1991	1987	1.064097943	1.013291603	1.117451709	Middle SDI
Incidence	Both	1987 to 1996	1992	1.106333934	1.039095522	1.177923249	Middle SDI
Incidence	Both	1992 to 2001	1997	1.168077639	1.066507798	1.279320575	Middle SDI
Incidence	Both	1997 to 2006	2002	1.197872093	1.005366477	1.427238311	Middle SDI
Incidence	Both	1927 to 1936	1932	0.787975086	0.773040236	0.803198472	Global
Incidence	Both	1932 to 1941	1937	0.872412091	0.85695967	0.888143146	Global
Incidence	Both	1937 to 1946	1942	0.925709948	0.909471749	0.942238072	Global
Incidence	Both	1942 to 1951	1947	0.962335292	0.945656965	0.97930777	Global
Incidence	Both	1947 to 1956	1952	0.959259559	0.943056049	0.975741477	Global
Incidence	Both	1952 to 1961	1957	0.979364889	0.963399857	0.995594488	Global
Incidence	Both	1957 to 1966	1962	0.974797442	0.958956883	0.990899664	Global
Incidence	Both	1962 to 1971	1967	0.94159412	0.926219167	0.957224293	Global
Incidence	Both	1967 to 1976	1972	0.963273626	0.947035927	0.979789734	Global
Incidence	Both	1972 to 1981	1977	1	1	1	Global
Incidence	Both	1977 to 1986	1982	1.016434992	0.994050786	1.03932325	Global

Incidence	Both	1982 to 1991	1987	1.001318243	0.975341777	1.027986546	Global
Incidence	Both	1987 to 1996	1992	1.027324967	0.99317458	1.062649618	Global
Incidence	Both	1992 to 2001	1997	1.050692769	1.000197205	1.103737633	Global
Incidence	Both	1997 to 2006	2002	1.076616866	0.979194659	1.183731821	Global

Abbreviation: CI: confidence interval; SDI: Sociodemographic index.
