# Supplementary material for: Trends in the global burden of aortic valve calcification disease in the working-age population from 1992 to 2021
Source: Front Cardiovasc Med. 2025 Aug 12;12:1544273. doi: 10.3389/fcvm.2025.1544273 (PMC12379075; doi:10.3389/fcvm.2025.1544273)
Supplement: Supplementary file 3 [file Datasheet3.zip › Supplementary Table 9.PDF]

Supplementary

Table S9. A cohort model of the time cycle of aortic valve calcification of working-age from 1992 to 2021

Measure	Sex	Label	Period	Rate Ratio	CI Low	CI High	Location
Deaths	Male	1992 to 1996	1994.5	1	1	1	High SDI
Deaths	Male	1997 to 2001	1999.5	0.810702325	0.761799229	0.862744715	High SDI
Deaths	Male	2002 to 2006	2004.5	0.679211533	0.633188678	0.728579525	High SDI
Deaths	Male	2007 to 2011	2009.5	0.652878856	0.605053337	0.704484671	High SDI
Deaths	Male	2012 to 2016	2014.5	0.655461993	0.604101089	0.711189621	High SDI
Deaths	Male	2017 to 2021	2019.5	0.638853004	0.585709446	0.696818472	High SDI
Deaths	Male	1992 to 1996	1994.5	1	1	1	Low-middle SDI
Deaths	Male	1997 to 2001	1999.5	1.039618976	0.943522219	1.145503087	Low-middle SDI
Deaths	Male	2002 to 2006	2004.5	1.053658979	0.954979597	1.162535041	Low-middle SDI
Deaths	Male	2007 to 2011	2009.5	1.080758507	0.980445673	1.191334699	Low-middle SDI
Deaths	Male	2012 to 2016	2014.5	1.093702255	0.994033898	1.203364016	Low-middle SDI
Deaths	Male	2017 to 2021	2019.5	1.12162603	1.02151013	1.231554063	Low-middle SDI
Deaths	Male	1992 to 1996	1994.5	1	1	1	High-middle SDI
Deaths	Male	1997 to 2001	1999.5	0.945475087	0.865394293	1.0329663	High-middle SDI
Deaths	Male	2002 to 2006	2004.5	0.905956116	0.824473367	0.99549181	High-middle SDI
Deaths	Male	2007 to 2011	2009.5	0.858927303	0.778807157	0.947289846	High-middle SDI
Deaths	Male	2012 to 2016	2014.5	0.865812074	0.783078681	0.957286368	High-middle SDI
Deaths	Male	2017 to 2021	2019.5	0.835468234	0.753110906	0.926831844	High-middle SDI
Deaths	Male	1992 to 1996	1994.5	1	1	1	Low SDI
Deaths	Male	1997 to 2001	1999.5	0.998109187	0.858378339	1.160586078	Low SDI

Deaths	Male	2002 to 2006	2004.5	0.969592116	0.830635432	1.131794811	Low SDI
Deaths	Male	2007 to 2011	2009.5	0.975331474	0.836752446	1.136861312	Low SDI
Deaths	Male	2012 to 2016	2014.5	1.00840449	0.868983547	1.170194325	Low SDI
Deaths	Male	2017 to 2021	2019.5	1.049927426	0.90917733	1.212467098	Low SDI
Deaths	Male	1992 to 1996	1994.5	1	1	1	Middle SDI
Deaths	Male	1997 to 2001	1999.5	0.902924709	0.831112607	0.98094172	Middle SDI
Deaths	Male	2002 to 2006	2004.5	0.853959976	0.784548146	0.929512923	Middle SDI
Deaths	Male	2007 to 2011	2009.5	0.836547622	0.768597148	0.910505492	Middle SDI
Deaths	Male	2012 to 2016	2014.5	0.817482105	0.751376095	0.889404116	Middle SDI
Deaths	Male	2017 to 2021	2019.5	0.834034502	0.767105491	0.906802987	Middle SDI
Deaths	Male	1992 to 1996	1994.5	1	1	1	Global
Deaths	Male	1997 to 2001	1999.5	0.906550371	0.872687605	0.941727109	Global
Deaths	Male	2002 to 2006	2004.5	0.836783941	0.803824803	0.871094497	Global
Deaths	Male	2007 to 2011	2009.5	0.818203981	0.78534082	0.852442325	Global
Deaths	Male	2012 to 2016	2014.5	0.818474718	0.785328689	0.853019728	Global
Deaths	Male	2017 to 2021	2019.5	0.812733077	0.779648282	0.847221842	Global
Deaths	Female	1992 to 1996	1994.5	1	1	1	High SDI
Deaths	Female	1997 to 2001	1999.5	0.882541134	0.801023024	0.972355139	High SDI
Deaths	Female	2002 to 2006	2004.5	0.780961171	0.699783817	0.871555382	High SDI
Deaths	Female	2007 to 2011	2009.5	0.762733533	0.676977431	0.859352786	High SDI
Deaths	Female	2012 to 2016	2014.5	0.783818465	0.690104352	0.890258674	High SDI
Deaths	Female	2017 to 2021	2019.5	0.752246117	0.656864498	0.861477859	High SDI
Deaths	Female	1992 to 1996	1994.5	1	1	1	Low-middle SDI
Deaths	Female	1997 to 2001	1999.5	1.00758011	0.884754716	1.147456645	Low-middle SDI

Deaths	Female	2002 to 2006	2004.5	1.009134383	0.883482179	1.152657321	Low-middle SDI
Deaths	Female	2007 to 2011	2009.5	1.009248862	0.885039557	1.150890102	Low-middle SDI
Deaths	Female	2012 to 2016	2014.5	1.061617146	0.934298432	1.206285835	Low-middle SDI
Deaths	Female	2017 to 2021	2019.5	1.082140917	0.954629648	1.226684051	Low-middle SDI
Deaths	Female	1992 to 1996	1994.5	1	1	1	High-middle SDI
Deaths	Female	1997 to 2001	1999.5	0.996703419	0.865918827	1.147241144	High-middle SDI
Deaths	Female	2002 to 2006	2004.5	0.953489304	0.819096639	1.109932344	High-middle SDI
Deaths	Female	2007 to 2011	2009.5	0.962078428	0.822376189	1.125512769	High-middle SDI
Deaths	Female	2012 to 2016	2014.5	0.997625755	0.849446057	1.171654326	High-middle SDI
Deaths	Female	2017 to 2021	2019.5	0.941257914	0.796657408	1.112104716	High-middle SDI
Deaths	Female	1992 to 1996	1994.5	1	1	1	Low SDI
Deaths	Female	1997 to 2001	1999.5	0.969841352	0.802993432	1.171357337	Low SDI
Deaths	Female	2002 to 2006	2004.5	0.912881371	0.750513977	1.110375588	Low SDI
Deaths	Female	2007 to 2011	2009.5	0.885100562	0.729792889	1.073459358	Low SDI
Deaths	Female	2012 to 2016	2014.5	0.919106689	0.762590936	1.10774606	Low SDI
Deaths	Female	2017 to 2021	2019.5	0.940543086	0.784382694	1.12779298	Low SDI
Deaths	Female	1992 to 1996	1994.5	1	1	1	Middle SDI
Deaths	Female	1997 to 2001	1999.5	0.901122792	0.804817374	1.008952232	Middle SDI
Deaths	Female	2002 to 2006	2004.5	0.848383026	0.755257763	0.952990879	Middle SDI
Deaths	Female	2007 to 2011	2009.5	0.799673526	0.71191932	0.89824469	Middle SDI
Deaths	Female	2012 to 2016	2014.5	0.808830161	0.721279267	0.907008227	Middle SDI
Deaths	Female	2017 to 2021	2019.5	0.848663347	0.757621709	0.950645248	Middle SDI
Deaths	Female	1992 to 1996	1994.5	1	1	1	Global
Deaths	Female	1997 to 2001	1999.5	0.940643345	0.890028435	0.994136667	Global

Deaths	Female	2002 to 2006	2004.5	0.882701361	0.832528411	0.935898021	Global
Deaths	Female	2007 to 2011	2009.5	0.861717768	0.812079974	0.914389635	Global
Deaths	Female	2012 to 2016	2014.5	0.887451825	0.83650036	0.941506758	Global
Deaths	Female	2017 to 2021	2019.5	0.884363214	0.833486175	0.938345851	Global
Deaths	Both	1992 to 1996	1994.5	1	1	1	High SDI
Deaths	Both	1997 to 2001	1999.5	0.831720847	0.789333009	0.876384947	High SDI
Deaths	Both	2002 to 2006	2004.5	0.707423179	0.666863857	0.75044936	High SDI
Deaths	Both	2007 to 2011	2009.5	0.683581887	0.641168704	0.728800693	High SDI
Deaths	Both	2012 to 2016	2014.5	0.691588596	0.645726098	0.740708463	High SDI
Deaths	Both	2017 to 2021	2019.5	0.671807118	0.624493184	0.722705732	High SDI
Deaths	Both	1992 to 1996	1994.5	1	1	1	Low-middle SDI
Deaths	Both	1997 to 2001	1999.5	1.025373449	0.948698962	1.108244821	Low-middle SDI
Deaths	Both	2002 to 2006	2004.5	1.034392798	0.955846468	1.119393644	Low-middle SDI
Deaths	Both	2007 to 2011	2009.5	1.051206809	0.972212945	1.136619051	Low-middle SDI
Deaths	Both	2012 to 2016	2014.5	1.075642606	0.996518812	1.161048845	Low-middle SDI
Deaths	Both	2017 to 2021	2019.5	1.099252869	1.019974149	1.184693624	Low-middle SDI
Deaths	Both	1992 to 1996	1994.5	1	1	1	High-middle SDI
Deaths	Both	1997 to 2001	1999.5	0.957099965	0.88805699	1.031510763	High-middle SDI
Deaths	Both	2002 to 2006	2004.5	0.919242034	0.848551472	0.995821638	High-middle SDI
Deaths	Both	2007 to 2011	2009.5	0.886996751	0.81635353	0.96375309	High-middle SDI
Deaths	Both	2012 to 2016	2014.5	0.900695601	0.827186178	0.980737573	High-middle SDI
Deaths	Both	2017 to 2021	2019.5	0.865172264	0.792251773	0.944804508	High-middle SDI
Deaths	Both	1992 to 1996	1994.5	1	1	1	Low SDI
Deaths	Both	1997 to 2001	1999.5	0.990332863	0.880324949	1.11408768	Low SDI

Deaths	Both	2002 to 2006	2004.5	0.949545099	0.841177445	1.071873597	Low SDI
Deaths	Both	2007 to 2011	2009.5	0.942791057	0.836466693	1.062630449	Low SDI
Deaths	Both	2012 to 2016	2014.5	0.976726652	0.86988842	1.096686575	Low SDI
Deaths	Both	2017 to 2021	2019.5	1.010842731	0.903521833	1.130911273	Low SDI
Deaths	Both	1992 to 1996	1994.5	1	1	1	Middle SDI
Deaths	Both	1997 to 2001	1999.5	0.900791439	0.842545951	0.963063458	Middle SDI
Deaths	Both	2002 to 2006	2004.5	0.849886634	0.793622044	0.910140155	Middle SDI
Deaths	Both	2007 to 2011	2009.5	0.821033764	0.766721795	0.87919301	Middle SDI
Deaths	Both	2012 to 2016	2014.5	0.810868003	0.757643758	0.867831235	Middle SDI
Deaths	Both	2017 to 2021	2019.5	0.835826779	0.781412462	0.894030283	Middle SDI
Deaths	Both	1992 to 1996	1994.5	1	1	1	Global
Deaths	Both	1997 to 2001	1999.5	0.916010245	0.88774464	0.945175821	Global
Deaths	Both	2002 to 2006	2004.5	0.849714107	0.822056496	0.878302243	Global
Deaths	Both	2007 to 2011	2009.5	0.829637496	0.802163713	0.858052246	Global
Deaths	Both	2012 to 2016	2014.5	0.83771724	0.809863556	0.866528897	Global
Deaths	Both	2017 to 2021	2019.5	0.832991092	0.805181575	0.8617611	Global
DALYs (Disability-Adjusted Life Years)	Male	1992 to 1996	1994.5	1	1	1	High SDI
DALYs (Disability-Adjusted Life Years)	Male	1997 to 2001	1999.5	0.812458898	0.794177876	0.831160727	High SDI
DALYs (Disability-Adjusted Life Years)	Male	2002 to 2006	2004.5	0.685874569	0.669076101	0.703094796	High SDI
DALYs (Disability-Adjusted Life Years)	Male	2007 to 2011	2009.5	0.657910346	0.64104133	0.675223271	High SDI
DALYs (Disability-Adjusted Life Years)	Male	2012 to 2016	2014.5	0.658307837	0.640845991	0.676245486	High SDI
DALYs (Disability-Adjusted Life Years)	Male	2017 to 2021	2019.5	0.643694804	0.626187895	0.66169117	High SDI
DALYs (Disability-Adjusted Life Years)	Male	1992 to 1996	1994.5	1	1	1	Low-middle SDI
DALYs (Disability-Adjusted Life Years)	Male	1997 to 2001	1999.5	1.043503653	1.018824178	1.06878095	Low-middle SDI

DALYs (Disability-Adjusted Life Years)	Male	2002 to 2006	2004.5	1.061482865	1.036516815	1.08705026	Low-middle SDI
DALYs (Disability-Adjusted Life Years)	Male	2007 to 2011	2009.5	1.089344055	1.064317394	1.114959199	Low-middle SDI
DALYs (Disability-Adjusted Life Years)	Male	2012 to 2016	2014.5	1.09904029	1.074541901	1.124097215	Low-middle SDI
DALYs (Disability-Adjusted Life Years)	Male	2017 to 2021	2019.5	1.127009919	1.102673179	1.151883788	Low-middle SDI
DALYs (Disability-Adjusted Life Years)	Male	1992 to 1996	1994.5	1	1	1	High-middle SDI
DALYs (Disability-Adjusted Life Years)	Male	1997 to 2001	1999.5	0.946736216	0.923010802	0.971071476	High-middle SDI
DALYs (Disability-Adjusted Life Years)	Male	2002 to 2006	2004.5	0.913922319	0.890213683	0.938262375	High-middle SDI
DALYs (Disability-Adjusted Life Years)	Male	2007 to 2011	2009.5	0.866224131	0.84340039	0.889665518	High-middle SDI
DALYs (Disability-Adjusted Life Years)	Male	2012 to 2016	2014.5	0.869914552	0.846974159	0.893476289	High-middle SDI
DALYs (Disability-Adjusted Life Years)	Male	2017 to 2021	2019.5	0.839822844	0.817515823	0.862738541	High-middle SDI
DALYs (Disability-Adjusted Life Years)	Male	1992 to 1996	1994.5	1	1	1	Low SDI
DALYs (Disability-Adjusted Life Years)	Male	1997 to 2001	1999.5	1.003948716	0.981606722	1.026799227	Low SDI
DALYs (Disability-Adjusted Life Years)	Male	2002 to 2006	2004.5	0.973046787	0.951286442	0.995304892	Low SDI
DALYs (Disability-Adjusted Life Years)	Male	2007 to 2011	2009.5	0.98082815	0.959445514	1.002687329	Low SDI
DALYs (Disability-Adjusted Life Years)	Male	2012 to 2016	2014.5	1.015722427	0.994496033	1.037401874	Low SDI
DALYs (Disability-Adjusted Life Years)	Male	2017 to 2021	2019.5	1.057973861	1.036725701	1.079657512	Low SDI
DALYs (Disability-Adjusted Life Years)	Male	1992 to 1996	1994.5	1	1	1	Middle SDI
DALYs (Disability-Adjusted Life Years)	Male	1997 to 2001	1999.5	0.905795192	0.885282384	0.926783301	Middle SDI
DALYs (Disability-Adjusted Life Years)	Male	2002 to 2006	2004.5	0.859909339	0.840326204	0.879948843	Middle SDI
DALYs (Disability-Adjusted Life Years)	Male	2007 to 2011	2009.5	0.845411887	0.82644669	0.864812295	Middle SDI
DALYs (Disability-Adjusted Life Years)	Male	2012 to 2016	2014.5	0.822544011	0.804408182	0.841088721	Middle SDI
DALYs (Disability-Adjusted Life Years)	Male	2017 to 2021	2019.5	0.83798913	0.819930725	0.856445259	Middle SDI
DALYs (Disability-Adjusted Life Years)	Male	1992 to 1996	1994.5	1	1	1	Global
DALYs (Disability-Adjusted Life Years)	Male	1997 to 2001	1999.5	0.908733329	0.890286452	0.927562428	Global

DALYs (Disability-Adjusted Life Years)	Male	2002 to 2006	2004.5	0.844367693	0.826714777	0.862397553	Global
DALYs (Disability-Adjusted Life Years)	Male	2007 to 2011	2009.5	0.825479121	0.80823636	0.843089736	Global
DALYs (Disability-Adjusted Life Years)	Male	2012 to 2016	2014.5	0.822332267	0.805369404	0.839652406	Global
DALYs (Disability-Adjusted Life Years)	Male	2017 to 2021	2019.5	0.816859869	0.800286968	0.833775973	Global
DALYs (Disability-Adjusted Life Years)	Female	1992 to 1996	1994.5	1	1	1	High SDI
DALYs (Disability-Adjusted Life Years)	Female	1997 to 2001	1999.5	0.886586246	0.8664097	0.907232654	High SDI
DALYs (Disability-Adjusted Life Years)	Female	2002 to 2006	2004.5	0.788247648	0.768688746	0.808304217	High SDI
DALYs (Disability-Adjusted Life Years)	Female	2007 to 2011	2009.5	0.769249099	0.749232772	0.789800178	High SDI
DALYs (Disability-Adjusted Life Years)	Female	2012 to 2016	2014.5	0.786263502	0.765188162	0.807919314	High SDI
DALYs (Disability-Adjusted Life Years)	Female	2017 to 2021	2019.5	0.753952789	0.733173082	0.775321438	High SDI
DALYs (Disability-Adjusted Life Years)	Female	1992 to 1996	1994.5	1	1	1	Low-middle SDI
DALYs (Disability-Adjusted Life Years)	Female	1997 to 2001	1999.5	1.010967111	0.982013469	1.04077442	Low-middle SDI
DALYs (Disability-Adjusted Life Years)	Female	2002 to 2006	2004.5	1.020435497	0.991187279	1.05054678	Low-middle SDI
DALYs (Disability-Adjusted Life Years)	Female	2007 to 2011	2009.5	1.020299225	0.991724838	1.049696921	Low-middle SDI
DALYs (Disability-Adjusted Life Years)	Female	2012 to 2016	2014.5	1.066035754	1.037219099	1.095653012	Low-middle SDI
DALYs (Disability-Adjusted Life Years)	Female	2017 to 2021	2019.5	1.088988755	1.060382983	1.118366221	Low-middle SDI
DALYs (Disability-Adjusted Life Years)	Female	1992 to 1996	1994.5	1	1	1	High-middle SDI
DALYs (Disability-Adjusted Life Years)	Female	1997 to 2001	1999.5	0.98804278	0.962719176	1.014032502	High-middle SDI
DALYs (Disability-Adjusted Life Years)	Female	2002 to 2006	2004.5	0.953160162	0.927576601	0.979449346	High-middle SDI
DALYs (Disability-Adjusted Life Years)	Female	2007 to 2011	2009.5	0.960585764	0.934615211	0.987277973	High-middle SDI
DALYs (Disability-Adjusted Life Years)	Female	2012 to 2016	2014.5	0.995402184	0.968520497	1.023029983	High-middle SDI
DALYs (Disability-Adjusted Life Years)	Female	2017 to 2021	2019.5	0.938885898	0.913208038	0.965285776	High-middle SDI
DALYs (Disability-Adjusted Life Years)	Female	1992 to 1996	1994.5	1	1	1	Low SDI
DALYs (Disability-Adjusted Life Years)	Female	1997 to 2001	1999.5	0.985011585	0.958043863	1.012738415	Low SDI

DALYs (Disability-Adjusted Life Years)	Female	2002 to 2006	2004.5	0.930836744	0.904943119	0.957471276	Low SDI
DALYs (Disability-Adjusted Life Years)	Female	2007 to 2011	2009.5	0.899746945	0.875287527	0.924889867	Low SDI
DALYs (Disability-Adjusted Life Years)	Female	2012 to 2016	2014.5	0.932038496	0.907668732	0.957062558	Low SDI
DALYs (Disability-Adjusted Life Years)	Female	2017 to 2021	2019.5	0.954815049	0.930595134	0.979665317	Low SDI
DALYs (Disability-Adjusted Life Years)	Female	1992 to 1996	1994.5	1	1	1	Middle SDI
DALYs (Disability-Adjusted Life Years)	Female	1997 to 2001	1999.5	0.907700149	0.877138552	0.939326586	Middle SDI
DALYs (Disability-Adjusted Life Years)	Female	2002 to 2006	2004.5	0.864717959	0.835360703	0.895106923	Middle SDI
DALYs (Disability-Adjusted Life Years)	Female	2007 to 2011	2009.5	0.813040536	0.785745029	0.841284245	Middle SDI
DALYs (Disability-Adjusted Life Years)	Female	2012 to 2016	2014.5	0.820352905	0.793529158	0.848083378	Middle SDI
DALYs (Disability-Adjusted Life Years)	Female	2017 to 2021	2019.5	0.859815842	0.832417201	0.888116297	Middle SDI
DALYs (Disability-Adjusted Life Years)	Female	1992 to 1996	1994.5	1	1	1	Global
DALYs (Disability-Adjusted Life Years)	Female	1997 to 2001	1999.5	0.945178689	0.923337611	0.967536406	Global
DALYs (Disability-Adjusted Life Years)	Female	2002 to 2006	2004.5	0.893840784	0.872548814	0.915652323	Global
DALYs (Disability-Adjusted Life Years)	Female	2007 to 2011	2009.5	0.871376466	0.85071659	0.892538073	Global
DALYs (Disability-Adjusted Life Years)	Female	2012 to 2016	2014.5	0.89304336	0.872313153	0.914266213	Global
DALYs (Disability-Adjusted Life Years)	Female	2017 to 2021	2019.5	0.890258609	0.86994155	0.911050162	Global
DALYs (Disability-Adjusted Life Years)	Both	1992 to 1996	1994.5	1	1	1	High SDI
DALYs (Disability-Adjusted Life Years)	Both	1997 to 2001	1999.5	0.833454556	0.818223562	0.84896907	High SDI
DALYs (Disability-Adjusted Life Years)	Both	2002 to 2006	2004.5	0.714791417	0.700568861	0.729302712	High SDI
DALYs (Disability-Adjusted Life Years)	Both	2007 to 2011	2009.5	0.689865758	0.675489006	0.704548497	High SDI
DALYs (Disability-Adjusted Life Years)	Both	2012 to 2016	2014.5	0.695611958	0.68063401	0.710919508	High SDI
DALYs (Disability-Adjusted Life Years)	Both	2017 to 2021	2019.5	0.677263908	0.662296268	0.692569812	High SDI
DALYs (Disability-Adjusted Life Years)	Both	1992 to 1996	1994.5	1	1	1	Low-middle SDI
DALYs (Disability-Adjusted Life Years)	Both	1997 to 2001	1999.5	1.03074133	1.007184254	1.054849383	Low-middle SDI

DALYs (Disability-Adjusted Life Years)	Both	2002 to 2006	2004.5	1.044372628	1.020591508	1.06870788	Low-middle SDI
DALYs (Disability-Adjusted Life Years)	Both	2007 to 2011	2009.5	1.060125539	1.036550531	1.084236731	Low-middle SDI
DALYs (Disability-Adjusted Life Years)	Both	2012 to 2016	2014.5	1.081070774	1.057794588	1.104859141	Low-middle SDI
DALYs (Disability-Adjusted Life Years)	Both	2017 to 2021	2019.5	1.106325943	1.083221846	1.129922828	Low-middle SDI
DALYs (Disability-Adjusted Life Years)	Both	1992 to 1996	1994.5	1	1	1	High-middle SDI
DALYs (Disability-Adjusted Life Years)	Both	1997 to 2001	1999.5	0.958062892	0.935537746	0.981130381	High-middle SDI
DALYs (Disability-Adjusted Life Years)	Both	2002 to 2006	2004.5	0.924787456	0.902207734	0.947932285	High-middle SDI
DALYs (Disability-Adjusted Life Years)	Both	2007 to 2011	2009.5	0.892297058	0.870224253	0.914929729	High-middle SDI
DALYs (Disability-Adjusted Life Years)	Both	2012 to 2016	2014.5	0.904925363	0.882540083	0.927878438	High-middle SDI
DALYs (Disability-Adjusted Life Years)	Both	2017 to 2021	2019.5	0.869306177	0.847611396	0.89155624	High-middle SDI
DALYs (Disability-Adjusted Life Years)	Both	1992 to 1996	1994.5	1	1	1	Low SDI
DALYs (Disability-Adjusted Life Years)	Both	1997 to 2001	1999.5	0.996302892	0.978495231	1.014434636	Low SDI
DALYs (Disability-Adjusted Life Years)	Both	2002 to 2006	2004.5	0.956168661	0.93894257	0.973710786	Low SDI
DALYs (Disability-Adjusted Life Years)	Both	2007 to 2011	2009.5	0.947885833	0.931241623	0.964827528	Low SDI
DALYs (Disability-Adjusted Life Years)	Both	2012 to 2016	2014.5	0.981359686	0.964836461	0.998165878	Low SDI
DALYs (Disability-Adjusted Life Years)	Both	2017 to 2021	2019.5	1.014995962	0.998521321	1.031742418	Low SDI
DALYs (Disability-Adjusted Life Years)	Both	1992 to 1996	1994.5	1	1	1	Middle SDI
DALYs (Disability-Adjusted Life Years)	Both	1997 to 2001	1999.5	0.905219711	0.882843197	0.928163379	Middle SDI
DALYs (Disability-Adjusted Life Years)	Both	2002 to 2006	2004.5	0.859193205	0.83781953	0.881112147	Middle SDI
DALYs (Disability-Adjusted Life Years)	Both	2007 to 2011	2009.5	0.830504844	0.810126663	0.851395625	Middle SDI
DALYs (Disability-Adjusted Life Years)	Both	2012 to 2016	2014.5	0.817499662	0.797849398	0.837633893	Middle SDI
DALYs (Disability-Adjusted Life Years)	Both	2017 to 2021	2019.5	0.841295867	0.821549999	0.861516326	Middle SDI
DALYs (Disability-Adjusted Life Years)	Both	1992 to 1996	1994.5	1	1	1	Global
DALYs (Disability-Adjusted Life Years)	Both	1997 to 2001	1999.5	0.919674869	0.900599362	0.939154412	Global

DALYs (Disability-Adjusted Life Years)	Both	2002 to 2006	2004.5	0.858927167	0.840580916	0.877673837	Global
DALYs (Disability-Adjusted Life Years)	Both	2007 to 2011	2009.5	0.838288632	0.82042707	0.856539058	Global
DALYs (Disability-Adjusted Life Years)	Both	2012 to 2016	2014.5	0.842523473	0.824852198	0.86057333	Global
DALYs (Disability-Adjusted Life Years)	Both	2017 to 2021	2019.5	0.838191327	0.82090392	0.85584279	Global
Prevalence	Male	1992 to 1996	1994.5	1	1	1	High SDI
Prevalence	Male	1997 to 2001	1999.5	0.965273604	0.954995549	0.975662276	High SDI
Prevalence	Male	2002 to 2006	2004.5	1.010698677	0.997687052	1.023879997	High SDI
Prevalence	Male	2007 to 2011	2009.5	1.070689225	1.05411753	1.087521441	High SDI
Prevalence	Male	2012 to 2016	2014.5	1.080321031	1.060512112	1.100499955	High SDI
Prevalence	Male	2017 to 2021	2019.5	1.050129271	1.027903117	1.072836017	High SDI
Prevalence	Male	1992 to 1996	1994.5	1	1	1	Low-middle SDI
Prevalence	Male	1997 to 2001	1999.5	1.03176429	1.01719387	1.04654342	Low-middle SDI
Prevalence	Male	2002 to 2006	2004.5	1.062174704	1.045808639	1.078796885	Low-middle SDI
Prevalence	Male	2007 to 2011	2009.5	1.125506915	1.106659468	1.144675352	Low-middle SDI
Prevalence	Male	2012 to 2016	2014.5	1.189795749	1.16812472	1.211868819	Low-middle SDI
Prevalence	Male	2017 to 2021	2019.5	1.250318555	1.225786219	1.275341871	Low-middle SDI
Prevalence	Male	1992 to 1996	1994.5	1	1	1	High-middle SDI
Prevalence	Male	1997 to 2001	1999.5	0.999032867	0.991622069	1.00649905	High-middle SDI
Prevalence	Male	2002 to 2006	2004.5	1.03404469	1.024551683	1.043625655	High-middle SDI
Prevalence	Male	2007 to 2011	2009.5	1.10895835	1.096682964	1.121371137	High-middle SDI
Prevalence	Male	2012 to 2016	2014.5	1.168328632	1.152971646	1.183890165	High-middle SDI
Prevalence	Male	2017 to 2021	2019.5	1.190118588	1.171951737	1.20856705	High-middle SDI
Prevalence	Male	1992 to 1996	1994.5	1	1	1	Low SDI
Prevalence	Male	1997 to 2001	1999.5	1.002460907	0.974905876	1.030794762	Low SDI

Prevalence	Male	2002 to 2006	2004.5	1.014618122	0.984966026	1.045162886	Low SDI
Prevalence	Male	2007 to 2011	2009.5	1.051543739	1.019213165	1.084899876	Low SDI
Prevalence	Male	2012 to 2016	2014.5	1.079042752	1.044372064	1.114864424	Low SDI
Prevalence	Male	2017 to 2021	2019.5	1.114570658	1.077910155	1.15247801	Low SDI
Prevalence	Male	1992 to 1996	1994.5	1	1	1	Middle SDI
Prevalence	Male	1997 to 2001	1999.5	1.033197731	1.023191718	1.043301595	Middle SDI
Prevalence	Male	2002 to 2006	2004.5	1.090608884	1.078752184	1.102595903	Middle SDI
Prevalence	Male	2007 to 2011	2009.5	1.17665712	1.162250953	1.191241853	Middle SDI
Prevalence	Male	2012 to 2016	2014.5	1.257900171	1.240500619	1.275543774	Middle SDI
Prevalence	Male	2017 to 2021	2019.5	1.32844754	1.307853681	1.349365676	Middle SDI
Prevalence	Male	1992 to 1996	1994.5	1	1	1	Global
Prevalence	Male	1997 to 2001	1999.5	0.967991029	0.96303274	0.972974846	Global
Prevalence	Male	2002 to 2006	2004.5	0.987221746	0.981245872	0.993234013	Global
Prevalence	Male	2007 to 2011	2009.5	1.02576821	1.018485884	1.033102606	Global
Prevalence	Male	2012 to 2016	2014.5	1.03383064	1.025290279	1.04244214	Global
Prevalence	Male	2017 to 2021	2019.5	1.0132911	1.003738792	1.022934314	Global
Prevalence	Female	1992 to 1996	1994.5	1	1	1	High SDI
Prevalence	Female	1997 to 2001	1999.5	0.929755985	0.914040826	0.945741335	High SDI
Prevalence	Female	2002 to 2006	2004.5	0.979372874	0.959021545	1.000156077	High SDI
Prevalence	Female	2007 to 2011	2009.5	1.067925617	1.041020707	1.095525877	High SDI
Prevalence	Female	2012 to 2016	2014.5	1.102055509	1.069006838	1.13612589	High SDI
Prevalence	Female	2017 to 2021	2019.5	1.099667904	1.061498401	1.139209911	High SDI
Prevalence	Female	1992 to 1996	1994.5	1	1	1	Low-middle SDI
Prevalence	Female	1997 to 2001	1999.5	1.026537309	1.006997899	1.046455854	Low-middle SDI

Prevalence	Female	2002 to 2006	2004.5	1.057485554	1.035432209	1.080008606	Low-middle SDI
Prevalence	Female	2007 to 2011	2009.5	1.112967101	1.087669621	1.13885296	Low-middle SDI
Prevalence	Female	2012 to 2016	2014.5	1.176702974	1.14751927	1.206628878	Low-middle SDI
Prevalence	Female	2017 to 2021	2019.5	1.247863221	1.214316553	1.282336648	Low-middle SDI
Prevalence	Female	1992 to 1996	1994.5	1	1	1	High-middle SDI
Prevalence	Female	1997 to 2001	1999.5	0.992605985	0.982913057	1.002394499	High-middle SDI
Prevalence	Female	2002 to 2006	2004.5	1.031233182	1.018511572	1.044113691	High-middle SDI
Prevalence	Female	2007 to 2011	2009.5	1.103147498	1.08653433	1.120014681	High-middle SDI
Prevalence	Female	2012 to 2016	2014.5	1.165438935	1.144442582	1.186820493	High-middle SDI
Prevalence	Female	2017 to 2021	2019.5	1.19639213	1.171214155	1.222111365	High-middle SDI
Prevalence	Female	1992 to 1996	1994.5	1	1	1	Low SDI
Prevalence	Female	1997 to 2001	1999.5	0.995279536	0.954628002	1.03766216	Low SDI
Prevalence	Female	2002 to 2006	2004.5	1.003974947	0.959680228	1.05031412	Low SDI
Prevalence	Female	2007 to 2011	2009.5	1.040111741	0.99129811	1.091329059	Low SDI
Prevalence	Female	2012 to 2016	2014.5	1.091247917	1.037136303	1.148182752	Low SDI
Prevalence	Female	2017 to 2021	2019.5	1.14746089	1.088097805	1.210062632	Low SDI
Prevalence	Female	1992 to 1996	1994.5	1	1	1	Middle SDI
Prevalence	Female	1997 to 2001	1999.5	1.03039033	1.017295795	1.043653416	Middle SDI
Prevalence	Female	2002 to 2006	2004.5	1.090708162	1.075224545	1.106414748	Middle SDI
Prevalence	Female	2007 to 2011	2009.5	1.177403887	1.158656542	1.196454568	Middle SDI
Prevalence	Female	2012 to 2016	2014.5	1.256057598	1.233506968	1.279020493	Middle SDI
Prevalence	Female	2017 to 2021	2019.5	1.329597171	1.302855558	1.356887666	Middle SDI
Prevalence	Female	1992 to 1996	1994.5	1	1	1	Global
Prevalence	Female	1997 to 2001	1999.5	0.950296445	0.941262257	0.959417343	Global

Prevalence	Female	2002 to 2006	2004.5	0.972748698	0.961717054	0.983906883	Global
Prevalence	Female	2007 to 2011	2009.5	1.021590687	1.007943814	1.035422329	Global
Prevalence	Female	2012 to 2016	2014.5	1.039301217	1.023102781	1.055756118	Global
Prevalence	Female	2017 to 2021	2019.5	1.033114529	1.014691695	1.05187185	Global
Prevalence	Both	1992 to 1996	1994.5	1	1	1	High SDI
Prevalence	Both	1997 to 2001	1999.5	0.9542277	0.943135942	0.965449902	High SDI
Prevalence	Both	2002 to 2006	2004.5	1.001700497	0.987549211	1.016054568	High SDI
Prevalence	Both	2007 to 2011	2009.5	1.071891112	1.053636874	1.090461604	High SDI
Prevalence	Both	2012 to 2016	2014.5	1.090394201	1.068360279	1.112882553	High SDI
Prevalence	Both	2017 to 2021	2019.5	1.069991785	1.045009334	1.095571478	High SDI
Prevalence	Both	1992 to 1996	1994.5	1	1	1	Low-middle SDI
Prevalence	Both	1997 to 2001	1999.5	1.029142209	1.017444358	1.040974553	Low-middle SDI
Prevalence	Both	2002 to 2006	2004.5	1.059132057	1.045976	1.072453588	Low-middle SDI
Prevalence	Both	2007 to 2011	2009.5	1.118317265	1.103202691	1.133638917	Low-middle SDI
Prevalence	Both	2012 to 2016	2014.5	1.180524588	1.163147982	1.198160788	Low-middle SDI
Prevalence	Both	2017 to 2021	2019.5	1.243690625	1.223922453	1.263778084	Low-middle SDI
Prevalence	Both	1992 to 1996	1994.5	1	1	1	High-middle SDI
Prevalence	Both	1997 to 2001	1999.5	0.996679247	0.989797834	1.003608503	High-middle SDI
Prevalence	Both	2002 to 2006	2004.5	1.033324897	1.024435781	1.042291145	High-middle SDI
Prevalence	Both	2007 to 2011	2009.5	1.107744858	1.096206027	1.119405148	High-middle SDI
Prevalence	Both	2012 to 2016	2014.5	1.169083905	1.154584483	1.183765414	High-middle SDI
Prevalence	Both	2017 to 2021	2019.5	1.195824658	1.178563603	1.213338515	High-middle SDI
Prevalence	Both	1992 to 1996	1994.5	1	1	1	Low SDI
Prevalence	Both	1997 to 2001	1999.5	0.999334291	0.976453712	1.022751016	Low SDI

Prevalence	Both	2002 to 2006	2004.5	1.009708986	0.985013023	1.03502412	Low SDI
Prevalence	Both	2007 to 2011	2009.5	1.04501034	1.018028877	1.072706911	Low SDI
Prevalence	Both	2012 to 2016	2014.5	1.078719584	1.049510646	1.108741436	Low SDI
Prevalence	Both	2017 to 2021	2019.5	1.119552189	1.08832798	1.151672222	Low SDI
Prevalence	Both	1992 to 1996	1994.5	1	1	1	Middle SDI
Prevalence	Both	1997 to 2001	1999.5	1.03095352	1.021855104	1.040132947	Middle SDI
Prevalence	Both	2002 to 2006	2004.5	1.088347515	1.077584042	1.099218498	Middle SDI
Prevalence	Both	2007 to 2011	2009.5	1.173440243	1.160389334	1.186637936	Middle SDI
Prevalence	Both	2012 to 2016	2014.5	1.252442103	1.236714493	1.268369725	Middle SDI
Prevalence	Both	2017 to 2021	2019.5	1.323165237	1.304543816	1.342052465	Middle SDI
Prevalence	Both	1992 to 1996	1994.5	1	1	1	Global
Prevalence	Both	1997 to 2001	1999.5	0.96124436	0.955485202	0.96703823	Global
Prevalence	Both	2002 to 2006	2004.5	0.980937617	0.973970936	0.987954131	Global
Prevalence	Both	2007 to 2011	2009.5	1.022124793	1.013598612	1.030722694	Global
Prevalence	Both	2012 to 2016	2014.5	1.032703453	1.022670165	1.042835176	Global
Prevalence	Both	2017 to 2021	2019.5	1.016894926	1.005610064	1.028306426	Global
Incidence	Male	1992 to 1996	1994.5	1	1	1	High SDI
Incidence	Male	1997 to 2001	1999.5	0.9561744	0.943399295	0.969122501	High SDI
Incidence	Male	2002 to 2006	2004.5	0.985456684	0.970788107	1.000346902	High SDI
Incidence	Male	2007 to 2011	2009.5	1.034050061	1.016970104	1.051416875	High SDI
Incidence	Male	2012 to 2016	2014.5	1.049678787	1.030474656	1.069240811	High SDI
Incidence	Male	2017 to 2021	2019.5	1.027306225	1.006685564	1.048349273	High SDI
Incidence	Male	1992 to 1996	1994.5	1	1	1	Low-middle SDI
Incidence	Male	1997 to 2001	1999.5	1.034791876	0.996588526	1.074459718	Low-middle SDI

Incidence	Male	2002 to 2006	2004.5	1.061775949	1.021266546	1.103892192	Low-middle SDI
Incidence	Male	2007 to 2011	2009.5	1.124405749	1.080503385	1.170091927	Low-middle SDI
Incidence	Male	2012 to 2016	2014.5	1.189936307	1.142414305	1.239435122	Low-middle SDI
Incidence	Male	2017 to 2021	2019.5	1.241174212	1.190968155	1.293496738	Low-middle SDI
Incidence	Male	1992 to 1996	1994.5	1	1	1	High-middle SDI
Incidence	Male	1997 to 2001	1999.5	0.992837707	0.975331961	1.010657656	High-middle SDI
Incidence	Male	2002 to 2006	2004.5	1.036061344	1.015487798	1.057051706	High-middle SDI
Incidence	Male	2007 to 2011	2009.5	1.11470547	1.090187506	1.139774835	High-middle SDI
Incidence	Male	2012 to 2016	2014.5	1.171868372	1.143243235	1.20121024	High-middle SDI
Incidence	Male	2017 to 2021	2019.5	1.183450251	1.151590979	1.216190924	High-middle SDI
Incidence	Male	1992 to 1996	1994.5	1	1	1	Low SDI
Incidence	Male	1997 to 2001	1999.5	0.999265641	0.92939377	1.074390483	Low SDI
Incidence	Male	2002 to 2006	2004.5	1.007757445	0.936320395	1.084644821	Low SDI
Incidence	Male	2007 to 2011	2009.5	1.035661796	0.962131604	1.114811479	Low SDI
Incidence	Male	2012 to 2016	2014.5	1.054455656	0.980213999	1.134320395	Low SDI
Incidence	Male	2017 to 2021	2019.5	1.079684402	1.006133072	1.158612554	Low SDI
Incidence	Male	1992 to 1996	1994.5	1	1	1	Middle SDI
Incidence	Male	1997 to 2001	1999.5	1.033901757	1.007727913	1.060755418	Middle SDI
Incidence	Male	2002 to 2006	2004.5	1.090670237	1.061764919	1.120362469	Middle SDI
Incidence	Male	2007 to 2011	2009.5	1.178177126	1.145505505	1.211780593	Middle SDI
Incidence	Male	2012 to 2016	2014.5	1.260677248	1.223762219	1.298705826	Middle SDI
Incidence	Male	2017 to 2021	2019.5	1.322699575	1.281820985	1.364881824	Middle SDI
Incidence	Male	1992 to 1996	1994.5	1	1	1	Global
Incidence	Male	1997 to 2001	1999.5	0.969926445	0.960865188	0.979073151	Global

Incidence	Male	2002 to 2006	2004.5	0.991622525	0.981494045	1.001855524	Global
Incidence	Male	2007 to 2011	2009.5	1.033035583	1.021593627	1.044605691	Global
Incidence	Male	2012 to 2016	2014.5	1.044902355	1.03232757	1.057630313	Global
Incidence	Male	2017 to 2021	2019.5	1.021298083	1.00804175	1.034728745	Global
Incidence	Female	1992 to 1996	1994.5	1	1	1	High SDI
Incidence	Female	1997 to 2001	1999.5	0.936841733	0.919170263	0.954852944	High SDI
Incidence	Female	2002 to 2006	2004.5	0.975969332	0.955028791	0.997369028	High SDI
Incidence	Female	2007 to 2011	2009.5	1.044128658	1.018792914	1.070094462	High SDI
Incidence	Female	2012 to 2016	2014.5	1.074272324	1.044993568	1.104371416	High SDI
Incidence	Female	2017 to 2021	2019.5	1.069614489	1.037333067	1.102900497	High SDI
Incidence	Female	1992 to 1996	1994.5	1	1	1	Low-middle SDI
Incidence	Female	1997 to 2001	1999.5	1.025854454	0.974473701	1.079944344	Low-middle SDI
Incidence	Female	2002 to 2006	2004.5	1.058901046	1.003887988	1.11692882	Low-middle SDI
Incidence	Female	2007 to 2011	2009.5	1.114901463	1.055461276	1.17768913	Low-middle SDI
Incidence	Female	2012 to 2016	2014.5	1.175868799	1.111570927	1.243885927	Low-middle SDI
Incidence	Female	2017 to 2021	2019.5	1.245974343	1.176510418	1.319539581	Low-middle SDI
Incidence	Female	1992 to 1996	1994.5	1	1	1	High-middle SDI
Incidence	Female	1997 to 2001	1999.5	0.978512142	0.954107962	1.003540533	High-middle SDI
Incidence	Female	2002 to 2006	2004.5	1.027869408	0.998258268	1.058358898	High-middle SDI
Incidence	Female	2007 to 2011	2009.5	1.11180732	1.075615003	1.149217437	High-middle SDI
Incidence	Female	2012 to 2016	2014.5	1.172152378	1.129132019	1.216811829	High-middle SDI
Incidence	Female	2017 to 2021	2019.5	1.190310598	1.141477124	1.241233213	High-middle SDI
Incidence	Female	1992 to 1996	1994.5	1	1	1	Low SDI
Incidence	Female	1997 to 2001	1999.5	0.990514207	0.890112626	1.102240733	Low SDI

Incidence	Female	2002 to 2006	2004.5	0.993364786	0.889482003	1.10938006	Low SDI
Incidence	Female	2007 to 2011	2009.5	1.022288033	0.913946679	1.143472422	Low SDI
Incidence	Female	2012 to 2016	2014.5	1.069507718	0.955854576	1.196674461	Low SDI
Incidence	Female	2017 to 2021	2019.5	1.120456212	1.002764137	1.251961529	Low SDI
Incidence	Female	1992 to 1996	1994.5	1	1	1	Middle SDI
Incidence	Female	1997 to 2001	1999.5	1.030288438	0.995476754	1.066317481	Middle SDI
Incidence	Female	2002 to 2006	2004.5	1.096559389	1.057916984	1.136613281	Middle SDI
Incidence	Female	2007 to 2011	2009.5	1.190055755	1.146344028	1.235434272	Middle SDI
Incidence	Female	2012 to 2016	2014.5	1.26830063	1.219227842	1.319348552	Middle SDI
Incidence	Female	2017 to 2021	2019.5	1.338423618	1.283814852	1.395355239	Middle SDI
Incidence	Female	1992 to 1996	1994.5	1	1	1	Global
Incidence	Female	1997 to 2001	1999.5	0.959123461	0.945985508	0.972443875	Global
Incidence	Female	2002 to 2006	2004.5	0.990261736	0.975229894	1.005525274	Global
Incidence	Female	2007 to 2011	2009.5	1.041344268	1.02400756	1.058974491	Global
Incidence	Female	2012 to 2016	2014.5	1.055506953	1.036242329	1.075129724	Global
Incidence	Female	2017 to 2021	2019.5	1.038454774	1.017846721	1.059480073	Global
Incidence	Both	1992 to 1996	1994.5	1	1	1	High SDI
Incidence	Both	1997 to 2001	1999.5	0.950793365	0.939679468	0.96203871	High SDI
Incidence	Both	2002 to 2006	2004.5	0.984470635	0.971564437	0.997548279	High SDI
Incidence	Both	2007 to 2011	2009.5	1.040407158	1.025176768	1.055863815	High SDI
Incidence	Both	2012 to 2016	2014.5	1.0611584	1.043870468	1.078732644	High SDI
Incidence	Both	2017 to 2021	2019.5	1.044625748	1.025892793	1.063700769	High SDI
Incidence	Both	1992 to 1996	1994.5	1	1	1	Low-middle SDI
Incidence	Both	1997 to 2001	1999.5	1.031046183	1.00022546	1.062816609	Low-middle SDI

Incidence	Both	2002 to 2006	2004.5	1.059215329	1.026448052	1.093028634	Low-middle SDI
Incidence	Both	2007 to 2011	2009.5	1.118043498	1.082609621	1.154637127	Low-middle SDI
Incidence	Both	2012 to 2016	2014.5	1.179905156	1.1416146	1.219480005	Low-middle SDI
Incidence	Both	2017 to 2021	2019.5	1.236904144	1.196145808	1.279051309	Low-middle SDI
Incidence	Both	1992 to 1996	1994.5	1	1	1	High-middle SDI
Incidence	Both	1997 to 2001	1999.5	0.987966321	0.973716195	1.002424995	High-middle SDI
Incidence	Both	2002 to 2006	2004.5	1.03421248	1.017273775	1.051433233	High-middle SDI
Incidence	Both	2007 to 2011	2009.5	1.115063537	1.094697071	1.135808914	High-middle SDI
Incidence	Both	2012 to 2016	2014.5	1.174107228	1.150161143	1.198551866	High-middle SDI
Incidence	Both	2017 to 2021	2019.5	1.189402597	1.162526949	1.216899563	High-middle SDI
Incidence	Both	1992 to 1996	1994.5	1	1	1	Low SDI
Incidence	Both	1997 to 2001	1999.5	0.997472211	0.939391118	1.059144368	Low SDI
Incidence	Both	2002 to 2006	2004.5	1.003298441	0.943774517	1.066576544	Low SDI
Incidence	Both	2007 to 2011	2009.5	1.030812996	0.969358941	1.096163029	Low SDI
Incidence	Both	2012 to 2016	2014.5	1.057902818	0.995147096	1.124616027	Low SDI
Incidence	Both	2017 to 2021	2019.5	1.090690972	1.027686921	1.157557591	Low SDI
Incidence	Both	1992 to 1996	1994.5	1	1	1	Middle SDI
Incidence	Both	1997 to 2001	1999.5	1.03105167	1.010078401	1.052460428	Middle SDI
Incidence	Both	2002 to 2006	2004.5	1.089917104	1.066734357	1.113603668	Middle SDI
Incidence	Both	2007 to 2011	2009.5	1.178117832	1.151927135	1.204904012	Middle SDI
Incidence	Both	2012 to 2016	2014.5	1.257433934	1.227931507	1.287645191	Middle SDI
Incidence	Both	2017 to 2021	2019.5	1.32142755	1.288702056	1.35498408	Middle SDI
Incidence	Both	1992 to 1996	1994.5	1	1	1	Global
Incidence	Both	1997 to 2001	1999.5	0.965816902	0.957066437	0.974647373	Global

Incidence	Both	2002 to 2006	2004.5	0.990382244	0.980528264	1.000335253	Global
Incidence	Both	2007 to 2011	2009.5	1.034133658	1.022932833	1.045457128	Global
Incidence	Both	2012 to 2016	2014.5	1.04576559	1.033420074	1.058258588	Global
Incidence	Both	2017 to 2021	2019.5	1.023959349	1.010883984	1.037203838	Global

Abbreviation: CI: confidence interval; SDI: Sociodemographic index.
