# Supplementary material for: Trends in the global burden of aortic valve calcification disease in the working-age population from 1992 to 2021
Source: Front Cardiovasc Med. 2025 Aug 12;12:1544273. doi: 10.3389/fcvm.2025.1544273 (PMC12379075; doi:10.3389/fcvm.2025.1544273)
Supplement: Supplementary file 3 [file Datasheet3.zip › Supplementary Table 8.PDF]

Supplementary

Table S8. The rate of change in the age-cohort and annual displacement of aortic valve calcification in the working-age range from 1992 to 2021

Measure	Sex	Net Drift (%/year)	CI Low	CI High	Location
Deaths	Male	-1.653394497	-1.975700689	-1.330028554	High SDI
Deaths	Male	0.430314164	0.104543954	0.757144527	Low-middle SDI
Deaths	Male	-0.692550917	-1.067195437	-0.31648767	High-middle SDI
Deaths	Male	0.160295783	-0.340946464	0.664059064	Low SDI
Deaths	Male	-0.698255867	-0.991531318	-0.404111697	Middle SDI
Deaths	Male	-0.777436041	-0.926050863	-0.62859829	Global
Deaths	Female	-1.024973369	-1.52987289	-0.517485007	High SDI
Deaths	Female	0.315666308	-0.121731123	0.754979235	Low-middle SDI
Deaths	Female	-0.166117767	-0.771588441	0.44304736	High-middle SDI
Deaths	Female	-0.284500048	-0.915889891	0.350913176	Low SDI
Deaths	Female	-0.685496756	-1.083470091	-0.285922244	Middle SDI
Deaths	Female	-0.463565457	-0.674853786	-0.251827667	Global
Deaths	Both	-1.461624136	-1.733097202	-1.189401095	High SDI
Deaths	Both	0.362290154	0.101401874	0.623858372	Low-middle SDI
Deaths	Both	-0.536875253	-0.855585231	-0.217140748	High-middle SDI
Deaths	Both	0.003017567	-0.38689858	0.394459965	Low SDI
Deaths	Both	-0.70987616	-0.94599384	-0.47319564	Middle SDI
Deaths	Both	-0.68655439	-0.807830869	-0.565129633	Global
DALYs (Disability-Adjusted Life Years)	Male	-1.629692705	-1.729418402	-1.529865806	High SDI
DALYs (Disability-Adjusted Life Years)	Male	0.446312573	0.37199997	0.520680194	Low-middle SDI

DALYs (Disability-Adjusted Life Years)	Male	-0.672187779	-0.766619913	-0.577665781	High-middle SDI
DALYs (Disability-Adjusted Life Years)	Male	0.185726971	0.116638375	0.254863243	Low SDI
DALYs (Disability-Adjusted Life Years)	Male	-0.677686118	-0.752245027	-0.603071196	Middle SDI
DALYs (Disability-Adjusted Life Years)	Male	-0.759265268	-0.830440106	-0.688039346	Global
DALYs (Disability-Adjusted Life Years)	Female	-1.02148102	-1.122914656	-0.919943327	High SDI
DALYs (Disability-Adjusted Life Years)	Female	0.334978324	0.243851951	0.426187535	Low-middle SDI
DALYs (Disability-Adjusted Life Years)	Female	-0.162886451	-0.260774448	-0.064902382	High-middle SDI
DALYs (Disability-Adjusted Life Years)	Female	-0.245980585	-0.334164362	-0.157718782	Low SDI
DALYs (Disability-Adjusted Life Years)	Female	-0.638152867	-0.749224385	-0.526957049	Middle SDI
DALYs (Disability-Adjusted Life Years)	Female	-0.442951254	-0.523042474	-0.362795551	Global
DALYs (Disability-Adjusted Life Years)	Both	-1.43324508	-1.514171292	-1.352252371	High SDI
DALYs (Disability-Adjusted Life Years)	Both	0.379699028	0.307782727	0.451666891	Low-middle SDI
DALYs (Disability-Adjusted Life Years)	Both	-0.517085412	-0.605888736	-0.428202746	High-middle SDI
DALYs (Disability-Adjusted Life Years)	Both	0.011649872	-0.044107979	0.067438826	Low SDI
DALYs (Disability-Adjusted Life Years)	Both	-0.685525783	-0.766845318	-0.604139609	Middle SDI
DALYs (Disability-Adjusted Life Years)	Both	-0.666183752	-0.738482209	-0.593832636	Global
Prevalence	Male	0.366402705	0.28230084	0.450575101	High SDI
Prevalence	Male	0.919885846	0.843792267	0.996036843	Low-middle SDI
Prevalence	Male	0.808872906	0.748026359	0.869756201	High-middle SDI
Prevalence	Male	0.457582369	0.332141082	0.583180489	Low SDI
Prevalence	Male	1.199336156	1.138355736	1.260353344	Middle SDI
Prevalence	Male	0.172566116	0.135556735	0.209589177	Global
Prevalence	Female	0.614240276	0.474551231	0.754123529	High SDI
Prevalence	Female	0.899953607	0.795146575	1.004869617	Low-middle SDI

Prevalence	Female	0.82943362	0.745099267	0.91383857	High-middle SDI
Prevalence	Female	0.572650358	0.371324592	0.774379944	Low SDI
Prevalence	Female	1.204328926	1.125139885	1.28357998	Middle SDI
Prevalence	Female	0.274931711	0.204410847	0.345502206	Global
Prevalence	Both	0.461723821	0.368655299	0.554878643	High SDI
Prevalence	Both	0.893390827	0.831836425	0.954982806	Low-middle SDI
Prevalence	Both	0.827614533	0.770046186	0.885215767	High-middle SDI
Prevalence	Both	0.474453747	0.368017974	0.58100239	Low SDI
Prevalence	Both	1.183662949	1.128378731	1.23897739	Middle SDI
Prevalence	Both	0.194485494	0.150842324	0.238147683	Global
Incidence	Male	0.264767645	0.18691364	0.342682149	High SDI
Incidence	Male	0.893510764	0.741421714	1.045829423	Low-middle SDI
Incidence	Male	0.810522376	0.704927555	0.916227919	High-middle SDI
Incidence	Male	0.327354587	0.076888329	0.578447697	Low SDI
Incidence	Male	1.190156887	1.071709707	1.308742877	Middle SDI
Incidence	Male	0.211458739	0.161728223	0.261213946	Global
Incidence	Female	0.466600422	0.34767419	0.585667599	High SDI
Incidence	Female	0.89574096	0.683617129	1.108311702	Low-middle SDI
Incidence	Female	0.855790571	0.692504662	1.01934127	High-middle SDI
Incidence	Female	0.474016428	0.071441182	0.878211185	Low SDI
Incidence	Female	1.243555873	1.086698175	1.400656969	Middle SDI
Incidence	Female	0.301159694	0.224315157	0.378063149	Global
Incidence	Both	0.345174794	0.275435633	0.414962457	High SDI
Incidence	Both	0.873328647	0.749739627	0.997069273	Low-middle SDI

Incidence	Both	0.837989418	0.749311032	0.926745858	High-middle SDI
Incidence	Both	0.364990336	0.152331481	0.578100741	Low SDI
Incidence	Both	1.188065467	1.093513391	1.282705977	Middle SDI
Incidence	Both	0.228949234	0.179916126	0.278006341	Global

Abbreviation: CI: confidence interval; SDI: Sociodemographic index.
