# Supplementary material for: Trends in the global burden of aortic valve calcification disease in the working-age population from 1992 to 2021
Source: Front Cardiovasc Med. 2025 Aug 12;12:1544273. doi: 10.3389/fcvm.2025.1544273 (PMC12379075; doi:10.3389/fcvm.2025.1544273)
Supplement: Supplementary file 3 [file Datasheet3.zip › Supplementary Table 7.PDF]

Supplementary

Table S7. Relationship between working-age aortic valve calcification age-cohort and Mean Percent Change per year from 1992 to 2021

Measure	Sex	Label	Age	Mean Percent Change per Calendar Year	CI Low	CI High	Location
Deaths	Male	15 to 19	17.5	-2.13345816	-3.923247955	-0.310326819	High SDI
Deaths	Male	20 to 24	22.5	-1.342054283	-2.445388338	-0.226241618	High SDI
Deaths	Male	25 to 29	27.5	-1.148871915	-1.951806166	-0.339362293	High SDI
Deaths	Male	30 to 34	32.5	-1.311080901	-1.937524047	-0.680635909	High SDI
Deaths	Male	35 to 39	37.5	-1.664997452	-2.170673515	-1.156707569	High SDI
Deaths	Male	40 to 44	42.5	-1.901315389	-2.317314431	-1.483544742	High SDI
Deaths	Male	45 to 49	47.5	-1.822541594	-2.168040418	-1.475822622	High SDI
Deaths	Male	50 to 54	52.5	-1.693864112	-1.982024387	-1.404856683	High SDI
Deaths	Male	55 to 59	57.5	-1.672850491	-1.921836858	-1.423232034	High SDI
Deaths	Male	60 to 64	62.5	-1.815837064	-2.082990176	-1.547955061	High SDI
Deaths	Male	15 to 19	17.5	-0.039069036	-1.067043723	0.999586944	Low-middle SDI
Deaths	Male	20 to 24	22.5	0.22436596	-0.572040562	1.027151606	Low-middle SDI
Deaths	Male	25 to 29	27.5	0.439016793	-0.25673108	1.139617776	Low-middle SDI
Deaths	Male	30 to 34	32.5	0.4872199	-0.135500822	1.113823696	Low-middle SDI
Deaths	Male	35 to 39	37.5	0.472728859	-0.081801067	1.030336336	Low-middle SDI
Deaths	Male	40 to 44	42.5	0.522423598	0.014805641	1.032617934	Low-middle SDI
Deaths	Male	45 to 49	47.5	0.462174608	-0.005489586	0.932026019	Low-middle SDI
Deaths	Male	50 to 54	52.5	0.508884695	0.081512563	0.93808181	Low-middle SDI
Deaths	Male	55 to 59	57.5	0.623245449	0.208080363	1.040130578	Low-middle SDI
Deaths	Male	60 to 64	62.5	0.406990078	-0.108523148	0.92516373	Low-middle SDI

Deaths	Male	15 to 19	17.5	-0.857321683	-2.480710751	0.793091701	High-middle SDI
Deaths	Male	20 to 24	22.5	-0.725828045	-1.869493515	0.431166315	High-middle SDI
Deaths	Male	25 to 29	27.5	-0.60919193	-1.514915647	0.304861328	High-middle SDI
Deaths	Male	30 to 34	32.5	-0.52914464	-1.270967986	0.218252566	High-middle SDI
Deaths	Male	35 to 39	37.5	-0.339690278	-0.959528518	0.284027178	High-middle SDI
Deaths	Male	40 to 44	42.5	-0.559775277	-1.090336801	-0.026367767	High-middle SDI
Deaths	Male	45 to 49	47.5	-0.991623766	-1.455772054	-0.525289316	High-middle SDI
Deaths	Male	50 to 54	52.5	-1.035370944	-1.436866771	-0.632239629	High-middle SDI
Deaths	Male	55 to 59	57.5	-0.761588718	-1.11428317	-0.407636316	High-middle SDI
Deaths	Male	60 to 64	62.5	-0.833190375	-1.228695013	-0.436102039	High-middle SDI
Deaths	Male	15 to 19	17.5	-0.051587328	-1.462078513	1.379093905	Low SDI
Deaths	Male	20 to 24	22.5	0.139775616	-1.033959949	1.327431665	Low SDI
Deaths	Male	25 to 29	27.5	0.287897197	-0.780603614	1.367904771	Low SDI
Deaths	Male	30 to 34	32.5	0.312065943	-0.659964323	1.293607409	Low SDI
Deaths	Male	35 to 39	37.5	0.25023917	-0.612752143	1.12072394	Low SDI
Deaths	Male	40 to 44	42.5	0.245735116	-0.545261161	1.043022448	Low SDI
Deaths	Male	45 to 49	47.5	0.106195582	-0.619781665	0.837476128	Low SDI
Deaths	Male	50 to 54	52.5	0.024585098	-0.642371947	0.696019219	Low SDI
Deaths	Male	55 to 59	57.5	0.04521416	-0.595369321	0.689925689	Low SDI
Deaths	Male	60 to 64	62.5	0.036078492	-0.762618908	0.84120409	Low SDI
Deaths	Male	15 to 19	17.5	-1.217344177	-2.30474038	-0.117844721	Middle SDI
Deaths	Male	20 to 24	22.5	-0.767650744	-1.56367052	0.034806163	Middle SDI
Deaths	Male	25 to 29	27.5	-0.585159744	-1.248375813	0.082510485	Middle SDI
Deaths	Male	30 to 34	32.5	-0.620821957	-1.193835022	-0.04448578	Middle SDI

Deaths	Male	35 to 39	37.5	-0.64215264	-1.142528323	-0.139244261	Middle SDI
Deaths	Male	40 to 44	42.5	-0.707321959	-1.158088045	-0.254500165	Middle SDI
Deaths	Male	45 to 49	47.5	-0.816300632	-1.226182414	-0.404717963	Middle SDI
Deaths	Male	50 to 54	52.5	-0.794301481	-1.168946372	-0.418236401	Middle SDI
Deaths	Male	55 to 59	57.5	-0.473260963	-0.839381869	-0.105788266	Middle SDI
Deaths	Male	60 to 64	62.5	-0.371043596	-0.821428612	0.081386687	Middle SDI
Deaths	Male	15 to 19	17.5	-0.730836018	-1.298111121	-0.160300582	Global
Deaths	Male	20 to 24	22.5	-0.458380086	-0.879361485	-0.035610711	Global
Deaths	Male	25 to 29	27.5	-0.345956981	-0.695563663	0.00488051	Global
Deaths	Male	30 to 34	32.5	-0.48303481	-0.780706119	-0.184470446	Global
Deaths	Male	35 to 39	37.5	-0.658802302	-0.912470031	-0.404485175	Global
Deaths	Male	40 to 44	42.5	-0.891744349	-1.112908732	-0.670085324	Global
Deaths	Male	45 to 49	47.5	-1.102728092	-1.297900019	-0.907170234	Global
Deaths	Male	50 to 54	52.5	-1.091787788	-1.263505667	-0.919771265	Global
Deaths	Male	55 to 59	57.5	-0.934460265	-1.091157267	-0.777515015	Global
Deaths	Male	60 to 64	62.5	-1.100413536	-1.278898202	-0.921606176	Global
Deaths	Female	15 to 19	17.5	-1.140171386	-3.685282243	1.472193879	High SDI
Deaths	Female	20 to 24	22.5	-0.603030209	-2.292480818	1.115632515	High SDI
Deaths	Female	25 to 29	27.5	-0.377463591	-1.635047256	0.896198124	High SDI
Deaths	Female	30 to 34	32.5	-0.528359396	-1.515203098	0.468372741	High SDI
Deaths	Female	35 to 39	37.5	-0.875297732	-1.681330453	-0.062657022	High SDI
Deaths	Female	40 to 44	42.5	-1.093296853	-1.767941223	-0.414019116	High SDI
Deaths	Female	45 to 49	47.5	-1.135386066	-1.691406448	-0.576220907	High SDI
Deaths	Female	50 to 54	52.5	-1.278705912	-1.731197402	-0.824130866	High SDI

Deaths	Female	55 to 59	57.5	-1.541948034	-1.912329625	-1.170167872	High SDI
Deaths	Female	60 to 64	62.5	-1.998074851	-2.3710963	-1.623628157	High SDI
Deaths	Female	15 to 19	17.5	-0.313794786	-1.552642164	0.94064207	Low-middle SDI
Deaths	Female	20 to 24	22.5	-0.063418199	-1.055594228	0.938706985	Low-middle SDI
Deaths	Female	25 to 29	27.5	0.219889749	-0.676005147	1.12386555	Low-middle SDI
Deaths	Female	30 to 34	32.5	0.265107083	-0.552727435	1.089667309	Low-middle SDI
Deaths	Female	35 to 39	37.5	0.411203228	-0.35405137	1.182334778	Low-middle SDI
Deaths	Female	40 to 44	42.5	0.463713759	-0.253166853	1.185746593	Low-middle SDI
Deaths	Female	45 to 49	47.5	0.438994192	-0.223904784	1.106297379	Low-middle SDI
Deaths	Female	50 to 54	52.5	0.469392719	-0.124027798	1.066339087	Low-middle SDI
Deaths	Female	55 to 59	57.5	0.581323415	0.051556417	1.113895498	Low-middle SDI
Deaths	Female	60 to 64	62.5	0.402684132	-0.231914481	1.041319259	Low-middle SDI
Deaths	Female	15 to 19	17.5	0.304184844	-2.0905878	2.757531387	High-middle SDI
Deaths	Female	20 to 24	22.5	0.271461366	-1.515528324	2.090875784	High-middle SDI
Deaths	Female	25 to 29	27.5	0.386331478	-1.060131969	1.853941674	High-middle SDI
Deaths	Female	30 to 34	32.5	0.277775237	-0.917091095	1.48705077	High-middle SDI
Deaths	Female	35 to 39	37.5	0.24854541	-0.774121329	1.281752215	High-middle SDI
Deaths	Female	40 to 44	42.5	-0.028046735	-0.908879131	0.860615481	High-middle SDI
Deaths	Female	45 to 49	47.5	-0.626970098	-1.387295295	0.139217371	High-middle SDI
Deaths	Female	50 to 54	52.5	-0.983154956	-1.629706586	-0.332353781	High-middle SDI
Deaths	Female	55 to 59	57.5	-0.889531976	-1.427503314	-0.348624594	High-middle SDI
Deaths	Female	60 to 64	62.5	-1.022385974	-1.579202347	-0.462419408	High-middle SDI
Deaths	Female	15 to 19	17.5	-0.297676112	-1.809928851	1.237867254	Low SDI
Deaths	Female	20 to 24	22.5	-0.235982367	-1.481936218	1.02572901	Low SDI

Deaths	Female	25 to 29	27.5	-0.194353527	-1.407383247	1.033600647	Low SDI
Deaths	Female	30 to 34	32.5	-0.345562991	-1.521185854	0.844094252	Low SDI
Deaths	Female	35 to 39	37.5	-0.41581124	-1.558183641	0.739817872	Low SDI
Deaths	Female	40 to 44	42.5	-0.399456219	-1.491187086	0.704373832	Low SDI
Deaths	Female	45 to 49	47.5	-0.315210787	-1.328921754	0.70891468	Low SDI
Deaths	Female	50 to 54	52.5	-0.224945347	-1.133310513	0.691765676	Low SDI
Deaths	Female	55 to 59	57.5	-0.062789298	-0.848911219	0.729565411	Low SDI
Deaths	Female	60 to 64	62.5	-0.123419783	-1.043798431	0.805519186	Low SDI
Deaths	Female	15 to 19	17.5	-1.136755757	-2.516644805	0.262665792	Middle SDI
Deaths	Female	20 to 24	22.5	-0.767297965	-1.814596726	0.291171852	Middle SDI
Deaths	Female	25 to 29	27.5	-0.599775577	-1.485773763	0.294190927	Middle SDI
Deaths	Female	30 to 34	32.5	-0.693918909	-1.460844347	0.078975473	Middle SDI
Deaths	Female	35 to 39	37.5	-0.661152813	-1.348142457	0.030620874	Middle SDI
Deaths	Female	40 to 44	42.5	-0.633722511	-1.262154958	-0.001290308	Middle SDI
Deaths	Female	45 to 49	47.5	-0.759283778	-1.332991938	-0.182239739	Middle SDI
Deaths	Female	50 to 54	52.5	-0.765690532	-1.283085374	-0.245583922	Middle SDI
Deaths	Female	55 to 59	57.5	-0.483196492	-0.959097278	-0.005008958	Middle SDI
Deaths	Female	60 to 64	62.5	-0.371112019	-0.94038336	0.201430785	Middle SDI
Deaths	Female	15 to 19	17.5	-0.22105225	-0.930369713	0.493343775	Global
Deaths	Female	20 to 24	22.5	-0.05005869	-0.598610364	0.501520195	Global
Deaths	Female	25 to 29	27.5	0.014744784	-0.461734875	0.493505304	Global
Deaths	Female	30 to 34	32.5	-0.17062356	-0.587035033	0.247532138	Global
Deaths	Female	35 to 39	37.5	-0.304917966	-0.67612859	0.067680011	Global
Deaths	Female	40 to 44	42.5	-0.484266409	-0.816753295	-0.150664944	Global

Deaths	Female	45 to 49	47.5	-0.742573578	-1.037283633	-0.446985879	Global
Deaths	Female	50 to 54	52.5	-0.875900505	-1.130895369	-0.62024798	Global
Deaths	Female	55 to 59	57.5	-0.820907704	-1.040015093	-0.60131519	Global
Deaths	Female	60 to 64	62.5	-1.122692999	-1.360460539	-0.884352327	Global
Deaths	Both	15 to 19	17.5	-1.828562244	-3.294473015	-0.340430463	High SDI
Deaths	Both	20 to 24	22.5	-1.153328065	-2.081844608	-0.216006791	High SDI
Deaths	Both	25 to 29	27.5	-0.907652826	-1.585038942	-0.225604289	High SDI
Deaths	Both	30 to 34	32.5	-1.074429924	-1.603914308	-0.542096304	High SDI
Deaths	Both	35 to 39	37.5	-1.41892778	-1.847661717	-0.988321114	High SDI
Deaths	Both	40 to 44	42.5	-1.656205591	-2.010716243	-1.300412371	High SDI
Deaths	Both	45 to 49	47.5	-1.616785415	-1.910370274	-1.322321849	High SDI
Deaths	Both	50 to 54	52.5	-1.567601412	-1.810593385	-1.3240081	High SDI
Deaths	Both	55 to 59	57.5	-1.622732231	-1.829240897	-1.415789161	High SDI
Deaths	Both	60 to 64	62.5	-1.849711237	-2.066995295	-1.631945091	High SDI
Deaths	Both	15 to 19	17.5	-0.140272914	-0.932349421	0.65813649	Low-middle SDI
Deaths	Both	20 to 24	22.5	0.124816176	-0.496859413	0.750375869	Low-middle SDI
Deaths	Both	25 to 29	27.5	0.360047424	-0.189810111	0.912934141	Low-middle SDI
Deaths	Both	30 to 34	32.5	0.389195519	-0.106210645	0.887058566	Low-middle SDI
Deaths	Both	35 to 39	37.5	0.411860098	-0.037273987	0.863012149	Low-middle SDI
Deaths	Both	40 to 44	42.5	0.456621689	0.042335461	0.872623522	Low-middle SDI
Deaths	Both	45 to 49	47.5	0.40578022	0.023753155	0.789266385	Low-middle SDI
Deaths	Both	50 to 54	52.5	0.451958896	0.105676005	0.799439639	Low-middle SDI
Deaths	Both	55 to 59	57.5	0.583628614	0.257131122	0.911189379	Low-middle SDI
Deaths	Both	60 to 64	62.5	0.380711995	-0.019572448	0.782599028	Low-middle SDI

Deaths	Both	15 to 19	17.5	-0.459282671	-1.806665831	0.906588926	High-middle SDI
Deaths	Both	20 to 24	22.5	-0.421388182	-1.390251917	0.556994865	High-middle SDI
Deaths	Both	25 to 29	27.5	-0.292848973	-1.061714196	0.481991225	High-middle SDI
Deaths	Both	30 to 34	32.5	-0.299124708	-0.930092081	0.33586124	High-middle SDI
Deaths	Both	35 to 39	37.5	-0.181592962	-0.712101696	0.351750352	High-middle SDI
Deaths	Both	40 to 44	42.5	-0.412839171	-0.867576276	0.04398389	High-middle SDI
Deaths	Both	45 to 49	47.5	-0.898492813	-1.294688999	-0.500706323	High-middle SDI
Deaths	Both	50 to 54	52.5	-1.03136751	-1.372318235	-0.689238136	High-middle SDI
Deaths	Both	55 to 59	57.5	-0.801974785	-1.096589287	-0.506482681	High-middle SDI
Deaths	Both	60 to 64	62.5	-0.882265222	-1.204840292	-0.558636915	High-middle SDI
Deaths	Both	15 to 19	17.5	-0.163523514	-1.194370771	0.878078658	Low SDI
Deaths	Both	20 to 24	22.5	-0.008164724	-0.861016272	0.852023552	Low SDI
Deaths	Both	25 to 29	27.5	0.112559735	-0.687049652	0.918607107	Low SDI
Deaths	Both	30 to 34	32.5	0.098518606	-0.647801256	0.850444719	Low SDI
Deaths	Both	35 to 39	37.5	0.048093307	-0.63847819	0.739408897	Low SDI
Deaths	Both	40 to 44	42.5	0.025898299	-0.613183207	0.669089255	Low SDI
Deaths	Both	45 to 49	47.5	-0.052920734	-0.643363798	0.541031135	Low SDI
Deaths	Both	50 to 54	52.5	-0.075471126	-0.612628722	0.464589639	Low SDI
Deaths	Both	55 to 59	57.5	-0.003343948	-0.499222851	0.49500625	Low SDI
Deaths	Both	60 to 64	62.5	-0.042298481	-0.64584257	0.564911942	Low SDI
Deaths	Both	15 to 19	17.5	-1.133651567	-1.989841539	-0.269982153	Middle SDI
Deaths	Both	20 to 24	22.5	-0.729914142	-1.364063782	-0.09168743	Middle SDI
Deaths	Both	25 to 29	27.5	-0.595562846	-1.127445731	-0.060818708	Middle SDI
Deaths	Both	30 to 34	32.5	-0.662184528	-1.121644005	-0.200590074	Middle SDI

Deaths	Both	35 to 39	37.5	-0.676759844	-1.081614184	-0.270248511	Middle SDI
Deaths	Both	40 to 44	42.5	-0.714453513	-1.081005588	-0.34654315	Middle SDI
Deaths	Both	45 to 49	47.5	-0.831141506	-1.164716866	-0.496440309	Middle SDI
Deaths	Both	50 to 54	52.5	-0.82132192	-1.124675697	-0.517037441	Middle SDI
Deaths	Both	55 to 59	57.5	-0.513655634	-0.803588349	-0.222875499	Middle SDI
Deaths	Both	60 to 64	62.5	-0.399179349	-0.752492574	-0.044608356	Middle SDI
Deaths	Both	15 to 19	17.5	-0.527614168	-0.970712099	-0.082533633	Global
Deaths	Both	20 to 24	22.5	-0.30295231	-0.636860844	0.032078319	Global
Deaths	Both	25 to 29	27.5	-0.22102203	-0.502811298	0.061565303	Global
Deaths	Both	30 to 34	32.5	-0.388682558	-0.630672574	-0.146103233	Global
Deaths	Both	35 to 39	37.5	-0.563682586	-0.773002692	-0.353920918	Global
Deaths	Both	40 to 44	42.5	-0.786886191	-0.970971433	-0.602458752	Global
Deaths	Both	45 to 49	47.5	-1.016758858	-1.179403036	-0.853846992	Global
Deaths	Both	50 to 54	52.5	-1.053459177	-1.195764772	-0.910948623	Global
Deaths	Both	55 to 59	57.5	-0.918015161	-1.045327941	-0.790538584	Global
Deaths	Both	60 to 64	62.5	-1.117607728	-1.260359404	-0.974649671	Global
DALYs (Disability-Adjusted Life Years)	Male	15 to 19	17.5	-2.035622785	-2.536727267	-1.531941891	High SDI
DALYs (Disability-Adjusted Life Years)	Male	20 to 24	22.5	-1.332569406	-1.652912452	-1.011182916	High SDI
DALYs (Disability-Adjusted Life Years)	Male	25 to 29	27.5	-1.141076666	-1.38221285	-0.899350865	High SDI
DALYs (Disability-Adjusted Life Years)	Male	30 to 34	32.5	-1.294084321	-1.489252591	-1.098529386	High SDI
DALYs (Disability-Adjusted Life Years)	Male	35 to 39	37.5	-1.639347264	-1.80316028	-1.475260973	High SDI
DALYs (Disability-Adjusted Life Years)	Male	40 to 44	42.5	-1.897079064	-2.037706494	-1.75624976	High SDI
DALYs (Disability-Adjusted Life Years)	Male	45 to 49	47.5	-1.834295849	-1.956632122	-1.711806927	High SDI
DALYs (Disability-Adjusted Life Years)	Male	50 to 54	52.5	-1.689646068	-1.797556654	-1.581616904	High SDI

DALYs (Disability-Adjusted Life Years)	Male	55 to 59	57.5	-1.632612098		-1.732615517	-1.53250691	High SDI
DALYs (Disability-Adjusted Life Years)	Male	60 to 64	62.5	-1.724338574		-1.837233546	-1.611313764	High SDI
DALYs (Disability-Adjusted Life Years)	Male	15 to 19	17.5	-0.025759351		-0.231511696	0.180417317	Low-middle SDI
DALYs (Disability-Adjusted Life Years)	Male	20 to 24	22.5	0.234843525		0.071516021	0.398437598	Low-middle SDI
DALYs (Disability-Adjusted Life Years)	Male	25 to 29	27.5	0.453536871		0.306118703	0.601171697	Low-middle SDI
DALYs (Disability-Adjusted Life Years)	Male	30 to 34	32.5	0.519582347		0.382406975	0.656945173	Low-middle SDI
DALYs (Disability-Adjusted Life Years)	Male	35 to 39	37.5	0.494105469		0.366292035	0.622081669	Low-middle SDI
DALYs (Disability-Adjusted Life Years)	Male	40 to 44	42.5	0.538638994		0.416471562	0.660955056	Low-middle SDI
DALYs (Disability-Adjusted Life Years)	Male	45 to 49	47.5	0.480634999		0.362954018	0.598453967	Low-middle SDI
DALYs (Disability-Adjusted Life Years)	Male	50 to 54	52.5	0.513019831		0.399190799	0.626977917	Low-middle SDI
DALYs (Disability-Adjusted Life Years)	Male	55 to 59	57.5	0.630642542		0.511682625	0.749743253	Low-middle SDI
DALYs (Disability-Adjusted Life Years)	Male	60 to 64	62.5	0.418455061		0.263638643	0.573510529	Low-middle SDI
DALYs (Disability-Adjusted Life Years)	Male	15 to 19	17.5	-0.888989708		-1.258763819	-0.517830836	High-middle SDI
DALYs (Disability-Adjusted Life Years)	Male	20 to 24	22.5	-0.71739991		-0.982937887	-0.45114983	High-middle SDI
DALYs (Disability-Adjusted Life Years)	Male	25 to 29	27.5	-0.578041716		-0.794013686	-0.361599575	High-middle SDI
DALYs (Disability-Adjusted Life Years)	Male	30 to 34	32.5	-0.521643851		-0.705740146	-0.337206234	High-middle SDI
DALYs (Disability-Adjusted Life Years)	Male	35 to 39	37.5	-0.336664011		-0.497435416	-0.175632839	High-middle SDI
DALYs (Disability-Adjusted Life Years)	Male	40 to 44	42.5	-0.536309977		-0.679988247	-0.39242386	High-middle SDI
DALYs (Disability-Adjusted Life Years)	Male	45 to 49	47.5	-0.956242985		-1.087555591	-0.824756053	High-middle SDI
DALYs (Disability-Adjusted Life Years)	Male	50 to 54	52.5	-0.99637411		-1.116094607	-0.876508665	High-middle SDI
DALYs (Disability-Adjusted Life Years)	Male	55 to 59	57.5	-0.732123221		-0.844399116	-0.619720194	High-middle SDI
DALYs (Disability-Adjusted Life Years)	Male	60 to 64	62.5	-0.773001998		-0.905025815	-0.640802286	High-middle SDI
DALYs (Disability-Adjusted Life Years)	Male	15 to 19	17.5	-0.025620077		-0.195778057	0.144828009	Low SDI
DALYs (Disability-Adjusted Life Years)	Male	20 to 24	22.5	0.184248882		0.039337255	0.329370421	Low SDI

DALYs (Disability-Adjusted Life Years)	Male	25 to 29	27.5	0.350585927	0.213887471	0.487470849	Low SDI
DALYs (Disability-Adjusted Life Years)	Male	30 to 34	32.5	0.354672761	0.225095162	0.484417886	Low SDI
DALYs (Disability-Adjusted Life Years)	Male	35 to 39	37.5	0.298171918	0.177882096	0.41860618	Low SDI
DALYs (Disability-Adjusted Life Years)	Male	40 to 44	42.5	0.261493521	0.146541691	0.376577296	Low SDI
DALYs (Disability-Adjusted Life Years)	Male	45 to 49	47.5	0.108910968	-0.001470452	0.21941423	Low SDI
DALYs (Disability-Adjusted Life Years)	Male	50 to 54	52.5	0.014625555	-0.092798579	0.122165195	Low SDI
DALYs (Disability-Adjusted Life Years)	Male	55 to 59	57.5	0.05197265	-0.058943615	0.163012011	Low SDI
DALYs (Disability-Adjusted Life Years)	Male	60 to 64	62.5	0.040426088	-0.104561362	0.185623972	Low SDI
DALYs (Disability-Adjusted Life Years)	Male	15 to 19	17.5	-1.171381566	-1.414236454	-0.927928433	Middle SDI
DALYs (Disability-Adjusted Life Years)	Male	20 to 24	22.5	-0.7527451	-0.935141497	-0.570012879	Middle SDI
DALYs (Disability-Adjusted Life Years)	Male	25 to 29	27.5	-0.584100916	-0.741142489	-0.426810881	Middle SDI
DALYs (Disability-Adjusted Life Years)	Male	30 to 34	32.5	-0.609184674	-0.750355039	-0.467813512	Middle SDI
DALYs (Disability-Adjusted Life Years)	Male	35 to 39	37.5	-0.629153944	-0.758109313	-0.500031011	Middle SDI
DALYs (Disability-Adjusted Life Years)	Male	40 to 44	42.5	-0.701498863	-0.822848435	-0.580000813	Middle SDI
DALYs (Disability-Adjusted Life Years)	Male	45 to 49	47.5	-0.805391824	-0.921118039	-0.689530439	Middle SDI
DALYs (Disability-Adjusted Life Years)	Male	50 to 54	52.5	-0.766529441	-0.878616565	-0.654315569	Middle SDI
DALYs (Disability-Adjusted Life Years)	Male	55 to 59	57.5	-0.430723054	-0.547955465	-0.313352451	Middle SDI
DALYs (Disability-Adjusted Life Years)	Male	60 to 64	62.5	-0.307305517	-0.458234411	-0.156147779	Middle SDI
DALYs (Disability-Adjusted Life Years)	Male	15 to 19	17.5	-0.712199125	-0.953474408	-0.470336101	Global
DALYs (Disability-Adjusted Life Years)	Male	20 to 24	22.5	-0.439878677	-0.623355184	-0.256063422	Global
DALYs (Disability-Adjusted Life Years)	Male	25 to 29	27.5	-0.328847468	-0.486273273	-0.171172624	Global
DALYs (Disability-Adjusted Life Years)	Male	30 to 34	32.5	-0.461809303	-0.601154279	-0.322268982	Global
DALYs (Disability-Adjusted Life Years)	Male	35 to 39	37.5	-0.639655676	-0.7636858	-0.515470533	Global
DALYs (Disability-Adjusted Life Years)	Male	40 to 44	42.5	-0.882572439	-0.995504702	-0.769511357	Global

DALYs (Disability-Adjusted Life Years)	Male	45 to 49	47.5	-1.09565343	-1.199924657	-0.991272158	Global
DALYs (Disability-Adjusted Life Years)	Male	50 to 54	52.5	-1.082504791	-1.179530153	-0.985384167	Global
DALYs (Disability-Adjusted Life Years)	Male	55 to 59	57.5	-0.913106585	-1.007932938	-0.818189397	Global
DALYs (Disability-Adjusted Life Years)	Male	60 to 64	62.5	-1.045187857	-1.158419644	-0.931826353	Global
DALYs (Disability-Adjusted Life Years)	Female	15 to 19	17.5	-1.145545855	-1.614205225	-0.674654033	High SDI
DALYs (Disability-Adjusted Life Years)	Female	20 to 24	22.5	-0.686516356	-1.004970281	-0.367038008	High SDI
DALYs (Disability-Adjusted Life Years)	Female	25 to 29	27.5	-0.418526207	-0.662012968	-0.174442637	High SDI
DALYs (Disability-Adjusted Life Years)	Female	30 to 34	32.5	-0.552922468	-0.75136645	-0.354081704	High SDI
DALYs (Disability-Adjusted Life Years)	Female	35 to 39	37.5	-0.862731315	-1.031533372	-0.693641348	High SDI
DALYs (Disability-Adjusted Life Years)	Female	40 to 44	42.5	-1.070160204	-1.21739913	-0.922701814	High SDI
DALYs (Disability-Adjusted Life Years)	Female	45 to 49	47.5	-1.1239504	-1.250961666	-0.996775772	High SDI
DALYs (Disability-Adjusted Life Years)	Female	50 to 54	52.5	-1.253642533	-1.362454969	-1.144710061	High SDI
DALYs (Disability-Adjusted Life Years)	Female	55 to 59	57.5	-1.502180973	-1.597589249	-1.406680192	High SDI
DALYs (Disability-Adjusted Life Years)	Female	60 to 64	62.5	-1.932246161	-2.033909944	-1.830476878	High SDI
DALYs (Disability-Adjusted Life Years)	Female	15 to 19	17.5	-0.28931117	-0.514455327	-0.063657493	Low-middle SDI
DALYs (Disability-Adjusted Life Years)	Female	20 to 24	22.5	-0.027348268	-0.212109169	0.157754725	Low-middle SDI
DALYs (Disability-Adjusted Life Years)	Female	25 to 29	27.5	0.24472701	0.071939123	0.417813239	Low-middle SDI
DALYs (Disability-Adjusted Life Years)	Female	30 to 34	32.5	0.301392942	0.137140127	0.465915177	Low-middle SDI
DALYs (Disability-Adjusted Life Years)	Female	35 to 39	37.5	0.430667413	0.270219079	0.59137249	Low-middle SDI
DALYs (Disability-Adjusted Life Years)	Female	40 to 44	42.5	0.475332086	0.318391269	0.632518426	Low-middle SDI
DALYs (Disability-Adjusted Life Years)	Female	45 to 49	47.5	0.453731144	0.302381231	0.605309434	Low-middle SDI
DALYs (Disability-Adjusted Life Years)	Female	50 to 54	52.5	0.482433134	0.339708341	0.625360941	Low-middle SDI
DALYs (Disability-Adjusted Life Years)	Female	55 to 59	57.5	0.587381157	0.449817868	0.725132835	Low-middle SDI
DALYs (Disability-Adjusted Life Years)	Female	60 to 64	62.5	0.413394595	0.239503946	0.587586901	Low-middle SDI

DALYs (Disability-Adjusted Life Years)	Female	15 to 19	17.5	0.320206626	-0.025116075	0.666722104	High-middle SDI
DALYs (Disability-Adjusted Life Years)	Female	20 to 24	22.5	0.195592198	-0.069342926	0.461229714	High-middle SDI
DALYs (Disability-Adjusted Life Years)	Female	25 to 29	27.5	0.283377506	0.061181409	0.506067012	High-middle SDI
DALYs (Disability-Adjusted Life Years)	Female	30 to 34	32.5	0.238654375	0.047782696	0.4298902	High-middle SDI
DALYs (Disability-Adjusted Life Years)	Female	35 to 39	37.5	0.276550119	0.1067751	0.446613065	High-middle SDI
DALYs (Disability-Adjusted Life Years)	Female	40 to 44	42.5	0.000224767	-0.152566938	0.153250283	High-middle SDI
DALYs (Disability-Adjusted Life Years)	Female	45 to 49	47.5	-0.616325077	-0.753871115	-0.478588412	High-middle SDI
DALYs (Disability-Adjusted Life Years)	Female	50 to 54	52.5	-0.953789034	-1.076389291	-0.831036833	High-middle SDI
DALYs (Disability-Adjusted Life Years)	Female	55 to 59	57.5	-0.845470207	-0.95410415	-0.736717113	High-middle SDI
DALYs (Disability-Adjusted Life Years)	Female	60 to 64	62.5	-0.936776766	-1.055533295	-0.817877701	High-middle SDI
DALYs (Disability-Adjusted Life Years)	Female	15 to 19	17.5	-0.295716946	-0.479993265	-0.111099411	Low SDI
DALYs (Disability-Adjusted Life Years)	Female	20 to 24	22.5	-0.196561392	-0.351159566	-0.04172337	Low SDI
DALYs (Disability-Adjusted Life Years)	Female	25 to 29	27.5	-0.162568656	-0.318466579	-0.006426916	Low SDI
DALYs (Disability-Adjusted Life Years)	Female	30 to 34	32.5	-0.265796418	-0.423123725	-0.108220541	Low SDI
DALYs (Disability-Adjusted Life Years)	Female	35 to 39	37.5	-0.340219645	-0.499675618	-0.180508132	Low SDI
DALYs (Disability-Adjusted Life Years)	Female	40 to 44	42.5	-0.328864265	-0.487343434	-0.170132709	Low SDI
DALYs (Disability-Adjusted Life Years)	Female	45 to 49	47.5	-0.293484984	-0.447158971	-0.13957378	Low SDI
DALYs (Disability-Adjusted Life Years)	Female	50 to 54	52.5	-0.202199296	-0.347061455	-0.057126556	Low SDI
DALYs (Disability-Adjusted Life Years)	Female	55 to 59	57.5	-0.073769224	-0.209195523	0.061840862	Low SDI
DALYs (Disability-Adjusted Life Years)	Female	60 to 64	62.5	-0.146918228	-0.314603685	0.0210493	Low SDI
DALYs (Disability-Adjusted Life Years)	Female	15 to 19	17.5	-1.07008405	-1.409380207	-0.729620218	Middle SDI
DALYs (Disability-Adjusted Life Years)	Female	20 to 24	22.5	-0.655505653	-0.917958906	-0.3923572	Middle SDI
DALYs (Disability-Adjusted Life Years)	Female	25 to 29	27.5	-0.53889067	-0.769010451	-0.308237234	Middle SDI
DALYs (Disability-Adjusted Life Years)	Female	30 to 34	32.5	-0.632877959	-0.840210807	-0.4251116	Middle SDI

DALYs (Disability-Adjusted Life Years)	Female	35 to 39	37.5	-0.621766974		-0.815705993	-0.427448739	Middle SDI
DALYs (Disability-Adjusted Life Years)	Female	40 to 44	42.5	-0.621417569		-0.806827569	-0.435661004	Middle SDI
DALYs (Disability-Adjusted Life Years)	Female	45 to 49	47.5	-0.732963182		-0.910010524	-0.555599505	Middle SDI
DALYs (Disability-Adjusted Life Years)	Female	50 to 54	52.5	-0.721032604		-0.889686809	-0.552091404	Middle SDI
DALYs (Disability-Adjusted Life Years)	Female	55 to 59	57.5	-0.445050952		-0.611560772	-0.278262172	Middle SDI
DALYs (Disability-Adjusted Life Years)	Female	60 to 64	62.5	-0.320451952		-0.52978807	-0.110675283	Middle SDI
DALYs (Disability-Adjusted Life Years)	Female	15 to 19	17.5	-0.192548271		-0.429931588	0.045400989	Global
DALYs (Disability-Adjusted Life Years)	Female	20 to 24	22.5	-0.014250604		-0.202304314	0.174157464	Global
DALYs (Disability-Adjusted Life Years)	Female	25 to 29	27.5	0.035382294		-0.133433181	0.204483136	Global
DALYs (Disability-Adjusted Life Years)	Female	30 to 34	32.5	-0.145289281		-0.298807802	0.008465625	Global
DALYs (Disability-Adjusted Life Years)	Female	35 to 39	37.5	-0.288011754		-0.430709499	-0.145109502	Global
DALYs (Disability-Adjusted Life Years)	Female	40 to 44	42.5	-0.4743932		-0.607757148	-0.340850306	Global
DALYs (Disability-Adjusted Life Years)	Female	45 to 49	47.5	-0.732482076		-0.855922728	-0.608887733	Global
DALYs (Disability-Adjusted Life Years)	Female	50 to 54	52.5	-0.856985134		-0.969482309	-0.744360164	Global
DALYs (Disability-Adjusted Life Years)	Female	55 to 59	57.5	-0.802760066		-0.906498747	-0.698912783	Global
DALYs (Disability-Adjusted Life Years)	Female	60 to 64	62.5	-1.084293199		-1.202871439	-0.965572639	Global
DALYs (Disability-Adjusted Life Years)	Both	15 to 19	17.5	-1.754691116		-2.15119783	-1.356577661	High SDI
DALYs (Disability-Adjusted Life Years)	Both	20 to 24	22.5	-1.12790854		-1.386415736	-0.868723689	High SDI
DALYs (Disability-Adjusted Life Years)	Both	25 to 29	27.5	-0.908724772		-1.104192728	-0.712870473	High SDI
DALYs (Disability-Adjusted Life Years)	Both	30 to 34	32.5	-1.052099039		-1.21060625	-0.893337504	High SDI
DALYs (Disability-Adjusted Life Years)	Both	35 to 39	37.5	-1.38886644		-1.522402572	-1.255149232	High SDI
DALYs (Disability-Adjusted Life Years)	Both	40 to 44	42.5	-1.63455099		-1.749704711	-1.519262305	High SDI
DALYs (Disability-Adjusted Life Years)	Both	45 to 49	47.5	-1.613863481		-1.713764306	-1.513861115	High SDI
DALYs (Disability-Adjusted Life Years)	Both	50 to 54	52.5	-1.553811165		-1.641152077	-1.466392696	High SDI

DALYs (Disability-Adjusted Life Years)	Both	55 to 59	57.5	-1.583168692	-1.662771166	-1.503501781	High SDI
DALYs (Disability-Adjusted Life Years)	Both	60 to 64	62.5	-1.76372349	-1.851987496	-1.675380108	High SDI
DALYs (Disability-Adjusted Life Years)	Both	15 to 19	17.5	-0.121373539	-0.312388078	0.070007009	Low-middle SDI
DALYs (Disability-Adjusted Life Years)	Both	20 to 24	22.5	0.145997485	-0.007639033	0.299870064	Low-middle SDI
DALYs (Disability-Adjusted Life Years)	Both	25 to 29	27.5	0.380344683	0.239832865	0.521053464	Low-middle SDI
DALYs (Disability-Adjusted Life Years)	Both	30 to 34	32.5	0.427538684	0.295816722	0.559433641	Low-middle SDI
DALYs (Disability-Adjusted Life Years)	Both	35 to 39	37.5	0.437255062	0.312441992	0.562223431	Low-middle SDI
DALYs (Disability-Adjusted Life Years)	Both	40 to 44	42.5	0.467055079	0.346783835	0.587470474	Low-middle SDI
DALYs (Disability-Adjusted Life Years)	Both	45 to 49	47.5	0.421959636	0.306070377	0.537982787	Low-middle SDI
DALYs (Disability-Adjusted Life Years)	Both	50 to 54	52.5	0.460833149	0.349759204	0.572030039	Low-middle SDI
DALYs (Disability-Adjusted Life Years)	Both	55 to 59	57.5	0.585994472	0.473255401	0.698860045	Low-middle SDI
DALYs (Disability-Adjusted Life Years)	Both	60 to 64	62.5	0.387454834	0.242225164	0.532894912	Low-middle SDI
DALYs (Disability-Adjusted Life Years)	Both	15 to 19	17.5	-0.46097546	-0.798159546	-0.122645296	High-middle SDI
DALYs (Disability-Adjusted Life Years)	Both	20 to 24	22.5	-0.401578433	-0.648830141	-0.153711399	High-middle SDI
DALYs (Disability-Adjusted Life Years)	Both	25 to 29	27.5	-0.308671555	-0.511519825	-0.105409696	High-middle SDI
DALYs (Disability-Adjusted Life Years)	Both	30 to 34	32.5	-0.303278926	-0.476514566	-0.129741743	High-middle SDI
DALYs (Disability-Adjusted Life Years)	Both	35 to 39	37.5	-0.166970165	-0.319032559	-0.014675801	High-middle SDI
DALYs (Disability-Adjusted Life Years)	Both	40 to 44	42.5	-0.390364842	-0.5265151	-0.254028235	High-middle SDI
DALYs (Disability-Adjusted Life Years)	Both	45 to 49	47.5	-0.871056739	-0.994946665	-0.747011783	High-middle SDI
DALYs (Disability-Adjusted Life Years)	Both	50 to 54	52.5	-0.991400944	-1.103593189	-0.879081423	High-middle SDI
DALYs (Disability-Adjusted Life Years)	Both	55 to 59	57.5	-0.765496318	-0.868933766	-0.661950939	High-middle SDI
DALYs (Disability-Adjusted Life Years)	Both	60 to 64	62.5	-0.811905525	-0.93087765	-0.692790525	High-middle SDI
DALYs (Disability-Adjusted Life Years)	Both	15 to 19	17.5	-0.152108745	-0.280950252	-0.023100769	Low SDI
DALYs (Disability-Adjusted Life Years)	Both	20 to 24	22.5	9.99E-05	-0.108854586	0.109173188	Low SDI

DALYs (Disability-Adjusted Life Years)	Both	25 to 29	27.5	0.123312531	0.017682599	0.229054021	Low SDI
DALYs (Disability-Adjusted Life Years)	Both	30 to 34	32.5	0.105607379	0.002977881	0.208342201	Low SDI
DALYs (Disability-Adjusted Life Years)	Both	35 to 39	37.5	0.058330636	-0.040419106	0.157177933	Low SDI
DALYs (Disability-Adjusted Life Years)	Both	40 to 44	42.5	0.049379758	-0.046509572	0.145361077	Low SDI
DALYs (Disability-Adjusted Life Years)	Both	45 to 49	47.5	-0.034581839	-0.127051619	0.057973557	Low SDI
DALYs (Disability-Adjusted Life Years)	Both	50 to 54	52.5	-0.072357705	-0.161275648	0.01663943	Low SDI
DALYs (Disability-Adjusted Life Years)	Both	55 to 59	57.5	-0.01243973	-0.100887588	0.076086436	Low SDI
DALYs (Disability-Adjusted Life Years)	Both	60 to 64	62.5	-0.059326848	-0.172550586	0.054025308	Low SDI
DALYs (Disability-Adjusted Life Years)	Both	15 to 19	17.5	-1.12206676	-1.381000322	-0.862453343	Middle SDI
DALYs (Disability-Adjusted Life Years)	Both	20 to 24	22.5	-0.713895167	-0.910504442	-0.516895788	Middle SDI
DALYs (Disability-Adjusted Life Years)	Both	25 to 29	27.5	-0.571531738	-0.741919675	-0.40085131	Middle SDI
DALYs (Disability-Adjusted Life Years)	Both	30 to 34	32.5	-0.628504905	-0.781770718	-0.475002336	Middle SDI
DALYs (Disability-Adjusted Life Years)	Both	35 to 39	37.5	-0.650669188	-0.79183233	-0.509305186	Middle SDI
DALYs (Disability-Adjusted Life Years)	Both	40 to 44	42.5	-0.706068142	-0.839616817	-0.572339604	Middle SDI
DALYs (Disability-Adjusted Life Years)	Both	45 to 49	47.5	-0.817786105	-0.945171311	-0.690237081	Middle SDI
DALYs (Disability-Adjusted Life Years)	Both	50 to 54	52.5	-0.794455118	-0.917083069	-0.671675398	Middle SDI
DALYs (Disability-Adjusted Life Years)	Both	55 to 59	57.5	-0.474276851	-0.599916987	-0.348477907	Middle SDI
DALYs (Disability-Adjusted Life Years)	Both	60 to 64	62.5	-0.33983695	-0.500260098	-0.179155153	Middle SDI
DALYs (Disability-Adjusted Life Years)	Both	15 to 19	17.5	-0.506231898	-0.740148067	-0.27176448	Global
DALYs (Disability-Adjusted Life Years)	Both	20 to 24	22.5	-0.274983804	-0.455633463	-0.094006308	Global
DALYs (Disability-Adjusted Life Years)	Both	25 to 29	27.5	-0.19864993	-0.356202407	-0.040848338	Global
DALYs (Disability-Adjusted Life Years)	Both	30 to 34	32.5	-0.361277333	-0.501986096	-0.220369583	Global
DALYs (Disability-Adjusted Life Years)	Both	35 to 39	37.5	-0.544017305	-0.671085645	-0.41678641	Global
DALYs (Disability-Adjusted Life Years)	Both	40 to 44	42.5	-0.777314136	-0.893989592	-0.66050132	Global

DALYs (Disability-Adjusted Life Years)	Both	45 to 49	47.5	-1.00843684			-1.116231894	-0.900524277	Global
DALYs (Disability-Adjusted Life Years)	Both	50 to 54	52.5	-1.039867203			-1.139478379	-0.940155659	Global
DALYs (Disability-Adjusted Life Years)	Both	55 to 59	57.5	-0.898011675			-0.993532623	-0.802398569	Global
DALYs (Disability-Adjusted Life Years)	Both	60 to 64	62.5	-1.069115394			-1.181556429	-0.956546418	Global
Prevalence	Male	15 to 19	17.5	0.72045081			-0.064858907	1.511931643	High SDI
Prevalence	Male	20 to 24	22.5	0.56687242			0.270409885	0.864211485	High SDI
Prevalence	Male	25 to 29	27.5	0.407912631			0.238796514	0.577314069	High SDI
Prevalence	Male	30 to 34	32.5	0.2886522			0.173501107	0.403935661	High SDI
Prevalence	Male	35 to 39	37.5	0.311977769			0.226172149	0.397856849	High SDI
Prevalence	Male	40 to 44	42.5	0.330534322			0.262822514	0.398291859	High SDI
Prevalence	Male	45 to 49	47.5	0.295945972			0.241908844	0.350012231	High SDI
Prevalence	Male	50 to 54	52.5	0.213195596			0.169416133	0.256994193	High SDI
Prevalence	Male	55 to 59	57.5	0.245778791			0.209460942	0.282109801	High SDI
Prevalence	Male	60 to 64	62.5	0.461646227			0.421907705	0.501400473	High SDI
Prevalence	Male	15 to 19	17.5	0.488676057			-0.09698918	1.077774663	Low-middle SDI
Prevalence	Male	20 to 24	22.5	0.588627037			0.337507483	0.84037508	Low-middle SDI
Prevalence	Male	25 to 29	27.5	0.70606854			0.545019432	0.867375611	Low-middle SDI
Prevalence	Male	30 to 34	32.5	0.780040472			0.660386269	0.899836907	Low-middle SDI
Prevalence	Male	35 to 39	37.5	0.818675961			0.723631813	0.913809794	Low-middle SDI
Prevalence	Male	40 to 44	42.5	0.85909873			0.780323789	0.937935245	Low-middle SDI
Prevalence	Male	45 to 49	47.5	0.972202923			0.9062034	1.038245614	Low-middle SDI
Prevalence	Male	50 to 54	52.5	1.19298938			1.136994529	1.249015234	Low-middle SDI
Prevalence	Male	55 to 59	57.5	1.402284628			1.351712471	1.45288202	Low-middle SDI
Prevalence	Male	60 to 64	62.5	1.564623502			1.503152051	1.62613218	Low-middle SDI

Prevalence	Male	15 to 19	17.5	0.903385796	0.334941595	1.475050498	High-middle SDI
Prevalence	Male	20 to 24	22.5	0.817301457	0.600125351	1.034946404	High-middle SDI
Prevalence	Male	25 to 29	27.5	1.01038703	0.888035164	1.132887278	High-middle SDI
Prevalence	Male	30 to 34	32.5	1.076559109	0.994373123	1.158811975	High-middle SDI
Prevalence	Male	35 to 39	37.5	1.077657197	1.016282433	1.13906925	High-middle SDI
Prevalence	Male	40 to 44	42.5	0.845428232	0.798070699	0.892808016	High-middle SDI
Prevalence	Male	45 to 49	47.5	0.464864156	0.42752927	0.502212922	High-middle SDI
Prevalence	Male	50 to 54	52.5	0.367358101	0.337494804	0.397230286	High-middle SDI
Prevalence	Male	55 to 59	57.5	0.580856123	0.557063999	0.604653876	High-middle SDI
Prevalence	Male	60 to 64	62.5	0.778444694	0.752383719	0.80451241	High-middle SDI
Prevalence	Male	15 to 19	17.5	0.375164437	-0.472800343	1.230353817	Low SDI
Prevalence	Male	20 to 24	22.5	0.387630464	-0.010286152	0.787130619	Low SDI
Prevalence	Male	25 to 29	27.5	0.425781673	0.155250579	0.697043503	Low SDI
Prevalence	Male	30 to 34	32.5	0.461465703	0.25231193	0.671055828	Low SDI
Prevalence	Male	35 to 39	37.5	0.478907507	0.308330859	0.649774225	Low SDI
Prevalence	Male	40 to 44	42.5	0.465435925	0.32036934	0.610712282	Low SDI
Prevalence	Male	45 to 49	47.5	0.438262238	0.312842691	0.563838595	Low SDI
Prevalence	Male	50 to 54	52.5	0.440972834	0.329695736	0.552373351	Low SDI
Prevalence	Male	55 to 59	57.5	0.497698228	0.3899064	0.605605795	Low SDI
Prevalence	Male	60 to 64	62.5	0.594769232	0.458973942	0.730748084	Low SDI
Prevalence	Male	15 to 19	17.5	0.787170785	0.267279181	1.309758057	Middle SDI
Prevalence	Male	20 to 24	22.5	1.011672465	0.802445641	1.221333563	Middle SDI
Prevalence	Male	25 to 29	27.5	1.155221707	1.029283273	1.281317131	Middle SDI
Prevalence	Male	30 to 34	32.5	1.134916828	1.045310565	1.224602553	Middle SDI

Prevalence	Male	35 to 39	37.5	1.118040948	1.048393859	1.18773604	Middle SDI
Prevalence	Male	40 to 44	42.5	1.082452755	1.026561965	1.138374465	Middle SDI
Prevalence	Male	45 to 49	47.5	1.091800122	1.046603004	1.137017455	Middle SDI
Prevalence	Male	50 to 54	52.5	1.304481345	1.267056564	1.341919956	Middle SDI
Prevalence	Male	55 to 59	57.5	1.671993394	1.639244071	1.704753268	Middle SDI
Prevalence	Male	60 to 64	62.5	1.886541284	1.847468993	1.925628564	Middle SDI
Prevalence	Male	15 to 19	17.5	0.193727161	-0.136728725	0.525276552	Global
Prevalence	Male	20 to 24	22.5	0.206416883	0.07626951	0.336733511	Global
Prevalence	Male	25 to 29	27.5	0.263308982	0.186888371	0.339787886	Global
Prevalence	Male	30 to 34	32.5	0.1696162	0.116708827	0.222551532	Global
Prevalence	Male	35 to 39	37.5	0.106076263	0.066073379	0.14609514	Global
Prevalence	Male	40 to 44	42.5	0.024466947	-0.007147083	0.056090971	Global
Prevalence	Male	45 to 49	47.5	-0.044182613	-0.069503475	-0.018855336	Global
Prevalence	Male	50 to 54	52.5	0.08508402	0.064448526	0.10572377	Global
Prevalence	Male	55 to 59	57.5	0.378943901	0.361737142	0.39615361	Global
Prevalence	Male	60 to 64	62.5	0.640345907	0.62113952	0.659555961	Global
Prevalence	Female	15 to 19	17.5	1.178857578	-0.124596404	2.499322677	High SDI
Prevalence	Female	20 to 24	22.5	1.038151149	0.543369944	1.535367208	High SDI
Prevalence	Female	25 to 29	27.5	0.902368721	0.620717292	1.184808532	High SDI
Prevalence	Female	30 to 34	32.5	0.765418883	0.573658833	0.957544555	High SDI
Prevalence	Female	35 to 39	37.5	0.700614993	0.558100364	0.843331598	High SDI
Prevalence	Female	40 to 44	42.5	0.593004651	0.480939584	0.705194703	High SDI
Prevalence	Female	45 to 49	47.5	0.392336289	0.303770106	0.480980674	High SDI
Prevalence	Female	50 to 54	52.5	0.168133091	0.097561292	0.238754644	High SDI

Prevalence	Female	55 to 59	57.5	0.109385241	0.052774584	0.166027928	High SDI
Prevalence	Female	60 to 64	62.5	0.319583894	0.260684203	0.378518186	High SDI
Prevalence	Female	15 to 19	17.5	0.356480953	-0.47404941	1.193941977	Low-middle SDI
Prevalence	Female	20 to 24	22.5	0.537721995	0.192199576	0.88443598	Low-middle SDI
Prevalence	Female	25 to 29	27.5	0.70311528	0.48515967	0.921543643	Low-middle SDI
Prevalence	Female	30 to 34	32.5	0.784140744	0.623242612	0.945296155	Low-middle SDI
Prevalence	Female	35 to 39	37.5	0.810277556	0.682825296	0.937891155	Low-middle SDI
Prevalence	Female	40 to 44	42.5	0.851879868	0.746049875	0.957821031	Low-middle SDI
Prevalence	Female	45 to 49	47.5	0.984681655	0.895603478	1.073838477	Low-middle SDI
Prevalence	Female	50 to 54	52.5	1.221749126	1.146285562	1.297268992	Low-middle SDI
Prevalence	Female	55 to 59	57.5	1.407970623	1.340993179	1.474992334	Low-middle SDI
Prevalence	Female	60 to 64	62.5	1.453695762	1.373695072	1.533759586	Low-middle SDI
Prevalence	Female	15 to 19	17.5	1.199758086	0.405494683	2.000304555	High-middle SDI
Prevalence	Female	20 to 24	22.5	1.065688526	0.764039344	1.368240731	High-middle SDI
Prevalence	Female	25 to 29	27.5	1.113267595	0.944036859	1.282782043	High-middle SDI
Prevalence	Female	30 to 34	32.5	1.108339379	0.995090706	1.22171504	High-middle SDI
Prevalence	Female	35 to 39	37.5	1.062427338	0.978054533	1.14687064	High-middle SDI
Prevalence	Female	40 to 44	42.5	0.792233677	0.727378215	0.857130898	High-middle SDI
Prevalence	Female	45 to 49	47.5	0.389807256	0.339024427	0.440615787	High-middle SDI
Prevalence	Female	50 to 54	52.5	0.26060084	0.220613919	0.300603716	High-middle SDI
Prevalence	Female	55 to 59	57.5	0.4912753	0.460599909	0.521960058	High-middle SDI
Prevalence	Female	60 to 64	62.5	0.795504315	0.763899512	0.82711903	High-middle SDI
Prevalence	Female	15 to 19	17.5	0.252426937	-1.157311368	1.682271555	Low SDI
Prevalence	Female	20 to 24	22.5	0.344876424	-0.295752757	0.989621838	Low SDI

Prevalence	Female	25 to 29	27.5	0.436054		0.007243198	0.866703456	Low SDI
Prevalence	Female	30 to 34	32.5	0.520993751		0.191878478	0.851190119	Low SDI
Prevalence	Female	35 to 39	37.5	0.577249885		0.309989908	0.845221934	Low SDI
Prevalence	Female	40 to 44	42.5	0.611239493		0.384589336	0.838401384	Low SDI
Prevalence	Female	45 to 49	47.5	0.661783505		0.46767683	0.8562652	Low SDI
Prevalence	Female	50 to 54	52.5	0.699267044		0.532608969	0.866201396	Low SDI
Prevalence	Female	55 to 59	57.5	0.74505834		0.595067029	0.895273295	Low SDI
Prevalence	Female	60 to 64	62.5	0.825332872		0.645664874	1.005321606	Low SDI
Prevalence	Female	15 to 19	17.5	0.756263892		0.070863204	1.446358996	Middle SDI
Prevalence	Female	20 to 24	22.5	0.958214841		0.687934255	1.229220951	Middle SDI
Prevalence	Female	25 to 29	27.5	1.11670845		0.955794784	1.277878597	Middle SDI
Prevalence	Female	30 to 34	32.5	1.155969041		1.042306029	1.269759912	Middle SDI
Prevalence	Female	35 to 39	37.5	1.180181382		1.09192705	1.268512761	Middle SDI
Prevalence	Female	40 to 44	42.5	1.140905529		1.069451991	1.212409582	Middle SDI
Prevalence	Female	45 to 49	47.5	1.123797857		1.065229996	1.182399658	Middle SDI
Prevalence	Female	50 to 54	52.5	1.312232223		1.263261162	1.361226966	Middle SDI
Prevalence	Female	55 to 59	57.5	1.64385464		1.60090345	1.686823988	Middle SDI
Prevalence	Female	60 to 64	62.5	1.800962835		1.750239848	1.851711107	Middle SDI
Prevalence	Female	15 to 19	17.5	0.372006731		-0.261104564	1.009136818	Global
Prevalence	Female	20 to 24	22.5	0.434968654		0.187495453	0.683053138	Global
Prevalence	Female	25 to 29	27.5	0.492813783		0.348011623	0.637824892	Global
Prevalence	Female	30 to 34	32.5	0.393999427		0.293776308	0.494322697	Global
Prevalence	Female	35 to 39	37.5	0.29341879		0.21764278	0.369252095	Global
Prevalence	Female	40 to 44	42.5	0.148806712		0.08889922	0.208750062	Global

Prevalence	Female	45 to 49	47.5	0.006986805	-0.040866306	0.054862824	Global
Prevalence	Female	50 to 54	52.5	0.044778388	0.006171348	0.083400332	Global
Prevalence	Female	55 to 59	57.5	0.262356884	0.230998194	0.293725386	Global
Prevalence	Female	60 to 64	62.5	0.475753027	0.442245988	0.509271243	Global
Prevalence	Both	15 to 19	17.5	0.858911632	-0.010057969	1.735433073	High SDI
Prevalence	Both	20 to 24	22.5	0.71956003	0.391028185	1.049167003	High SDI
Prevalence	Both	25 to 29	27.5	0.575735038	0.38842113	0.763398454	High SDI
Prevalence	Both	30 to 34	32.5	0.46076827	0.333217997	0.588480694	High SDI
Prevalence	Both	35 to 39	37.5	0.458347432	0.363378385	0.553406343	High SDI
Prevalence	Both	40 to 44	42.5	0.439356647	0.364494038	0.514275096	High SDI
Prevalence	Both	45 to 49	47.5	0.345240229	0.285678279	0.404837554	High SDI
Prevalence	Both	50 to 54	52.5	0.215315339	0.167310714	0.26334297	High SDI
Prevalence	Both	55 to 59	57.5	0.220250322	0.180853141	0.259662996	High SDI
Prevalence	Both	60 to 64	62.5	0.437764713	0.395370648	0.48017668	High SDI
Prevalence	Both	15 to 19	17.5	0.456143119	-0.022954958	0.937537073	Low-middle SDI
Prevalence	Both	20 to 24	22.5	0.591021646	0.387772828	0.794681968	Low-middle SDI
Prevalence	Both	25 to 29	27.5	0.717642785	0.588067876	0.847384609	Low-middle SDI
Prevalence	Both	30 to 34	32.5	0.777261334	0.681232439	0.873381821	Low-middle SDI
Prevalence	Both	35 to 39	37.5	0.791518873	0.715335228	0.867760145	Low-middle SDI
Prevalence	Both	40 to 44	42.5	0.818255431	0.755084497	0.881465972	Low-middle SDI
Prevalence	Both	45 to 49	47.5	0.933433494	0.880424498	0.986470344	Low-middle SDI
Prevalence	Both	50 to 54	52.5	1.164060313	1.119111331	1.209029275	Low-middle SDI
Prevalence	Both	55 to 59	57.5	1.373064724	1.332721465	1.413424045	Low-middle SDI
Prevalence	Both	60 to 64	62.5	1.48471292	1.435984784	1.533464465	Low-middle SDI

Prevalence	Both	15 to 19	17.5	1.038982864	0.498975755	1.581891572	High-middle SDI
Prevalence	Both	20 to 24	22.5	0.943151387	0.737592352	1.149129874	High-middle SDI
Prevalence	Both	25 to 29	27.5	1.068399491	0.952767661	1.184163766	High-middle SDI
Prevalence	Both	30 to 34	32.5	1.096655293	1.01909049	1.174279652	High-middle SDI
Prevalence	Both	35 to 39	37.5	1.077998457	1.020123338	1.135906733	High-middle SDI
Prevalence	Both	40 to 44	42.5	0.832106647	0.787504606	0.876728425	High-middle SDI
Prevalence	Both	45 to 49	47.5	0.43826712	0.403180077	0.473366424	High-middle SDI
Prevalence	Both	50 to 54	52.5	0.330781452	0.30285516	0.358715519	High-middle SDI
Prevalence	Both	55 to 59	57.5	0.555979339	0.534005492	0.57795799	High-middle SDI
Prevalence	Both	60 to 64	62.5	0.797036411	0.773447566	0.820630778	High-middle SDI
Prevalence	Both	15 to 19	17.5	0.345585647	-0.382007996	1.078493517	Low SDI
Prevalence	Both	20 to 24	22.5	0.372841427	0.034505369	0.712321803	Low SDI
Prevalence	Both	25 to 29	27.5	0.412371997	0.183502122	0.641764727	Low SDI
Prevalence	Both	30 to 34	32.5	0.450251088	0.273757952	0.627054872	Low SDI
Prevalence	Both	35 to 39	37.5	0.475279477	0.331573802	0.619190983	Low SDI
Prevalence	Both	40 to 44	42.5	0.487149078	0.365019458	0.609427313	Low SDI
Prevalence	Both	45 to 49	47.5	0.489748009	0.384453887	0.595152575	Low SDI
Prevalence	Both	50 to 54	52.5	0.507652868	0.415227985	0.600162821	Low SDI
Prevalence	Both	55 to 59	57.5	0.561030113	0.47365231	0.648483904	Low SDI
Prevalence	Both	60 to 64	62.5	0.639272601	0.531000285	0.747661527	Low SDI
Prevalence	Both	15 to 19	17.5	0.78635251	0.311992984	1.262955207	Middle SDI
Prevalence	Both	20 to 24	22.5	0.997557369	0.808171989	1.187298542	Middle SDI
Prevalence	Both	25 to 29	27.5	1.140525058	1.027005264	1.254172409	Middle SDI
Prevalence	Both	30 to 34	32.5	1.136502841	1.055959451	1.217110426	Middle SDI

Prevalence	Both	35 to 39	37.5	1.126757483	1.064187098	1.189366606	Middle SDI
Prevalence	Both	40 to 44	42.5	1.080930066	1.030554801	1.13133045	Middle SDI
Prevalence	Both	45 to 49	47.5	1.070858096	1.029917713	1.11181507	Middle SDI
Prevalence	Both	50 to 54	52.5	1.266688748	1.232668702	1.300720226	Middle SDI
Prevalence	Both	55 to 59	57.5	1.62395704	1.594160036	1.653762782	Middle SDI
Prevalence	Both	60 to 64	62.5	1.824013016	1.788593017	1.85944534	Middle SDI
Prevalence	Both	15 to 19	17.5	0.263413373	-0.127107478	0.655461231	Global
Prevalence	Both	20 to 24	22.5	0.290154489	0.136735916	0.443808114	Global
Prevalence	Both	25 to 29	27.5	0.340201497	0.250227231	0.430256515	Global
Prevalence	Both	30 to 34	32.5	0.236358532	0.174079754	0.298676029	Global
Prevalence	Both	35 to 39	37.5	0.152487563	0.105404818	0.199592452	Global
Prevalence	Both	40 to 44	42.5	0.046152196	0.008940672	0.083377566	Global
Prevalence	Both	45 to 49	47.5	-0.05151396	-0.081290497	-0.021728549	Global
Prevalence	Both	50 to 54	52.5	0.046874134	0.022687866	0.071066252	Global
Prevalence	Both	55 to 59	57.5	0.32203645	0.302043995	0.34203289	Global
Prevalence	Both	60 to 64	62.5	0.570027005	0.548038814	0.592020005	Global
Incidence	Male	15 to 19	17.5	0.619277309	0.126591043	1.114387904	High SDI
Incidence	Male	20 to 24	22.5	0.504613887	0.24132936	0.768589933	High SDI
Incidence	Male	25 to 29	27.5	0.317817103	0.134095653	0.501875636	High SDI
Incidence	Male	30 to 34	32.5	0.122041874	-0.02150428	0.265794127	High SDI
Incidence	Male	35 to 39	37.5	0.064377777	-0.051856517	0.180747246	High SDI
Incidence	Male	40 to 44	42.5	0.03961887	-0.053509035	0.132833549	High SDI
Incidence	Male	45 to 49	47.5	0.06835986	-0.005357648	0.142131713	High SDI
Incidence	Male	50 to 54	52.5	0.182719658	0.124759739	0.240713129	High SDI

Incidence	Male	55 to 59	57.5	0.399544158		0.352100651	0.447010093	High SDI
Incidence	Male	60 to 64	62.5	0.879627897		0.827370512	0.931912366	High SDI
Incidence	Male	15 to 19	17.5	0.42290929		-0.339682493	1.191336356	Low-middle SDI
Incidence	Male	20 to 24	22.5	0.476707796		0.028884877	0.926535589	Low-middle SDI
Incidence	Male	25 to 29	27.5	0.58457984		0.242263286	0.928065369	Low-middle SDI
Incidence	Male	30 to 34	32.5	0.673631003		0.388127448	0.959946528	Low-middle SDI
Incidence	Male	35 to 39	37.5	0.769181494		0.527568029	1.011375667	Low-middle SDI
Incidence	Male	40 to 44	42.5	0.880113353		0.679245015	1.081382449	Low-middle SDI
Incidence	Male	45 to 49	47.5	1.026529088		0.856576685	1.196767877	Low-middle SDI
Incidence	Male	50 to 54	52.5	1.252582593		1.102314353	1.403074176	Low-middle SDI
Incidence	Male	55 to 59	57.5	1.449125012		1.301339824	1.5971258	Low-middle SDI
Incidence	Male	60 to 64	62.5	1.554376834		1.362306099	1.746811523	Low-middle SDI
Incidence	Male	15 to 19	17.5	0.887532911		0.205569081	1.574137947	High-middle SDI
Incidence	Male	20 to 24	22.5	0.758492682		0.391665053	1.126660686	High-middle SDI
Incidence	Male	25 to 29	27.5	0.924574013		0.671990728	1.177791022	High-middle SDI
Incidence	Male	30 to 34	32.5	0.984631235		0.79108432	1.178549814	High-middle SDI
Incidence	Male	35 to 39	37.5	0.988520997		0.834481017	1.142796297	High-middle SDI
Incidence	Male	40 to 44	42.5	0.793861476		0.677504472	0.910352959	High-middle SDI
Incidence	Male	45 to 49	47.5	0.465315735		0.374806964	0.55590612	High-middle SDI
Incidence	Male	50 to 54	52.5	0.452664127		0.380046	0.525334788	High-middle SDI
Incidence	Male	55 to 59	57.5	0.774323885		0.713561289	0.835123139	High-middle SDI
Incidence	Male	60 to 64	62.5	1.081049287		1.00904287	1.153107035	High-middle SDI
Incidence	Male	15 to 19	17.5	0.250365152		-0.833218908	1.345789412	Low SDI
Incidence	Male	20 to 24	22.5	0.240183377		-0.436330714	0.921294239	Low SDI

Incidence	Male	25 to 29	27.5	0.247356216	-0.292917642	0.790557608	Low SDI
Incidence	Male	30 to 34	32.5	0.289004941	-0.178612586	0.758813042	Low SDI
Incidence	Male	35 to 39	37.5	0.311403635	-0.0989695	0.723462499	Low SDI
Incidence	Male	40 to 44	42.5	0.321621127	-0.036641324	0.681167568	Low SDI
Incidence	Male	45 to 49	47.5	0.329355013	0.010321877	0.649405865	Low SDI
Incidence	Male	50 to 54	52.5	0.361916088	0.057634254	0.667123263	Low SDI
Incidence	Male	55 to 59	57.5	0.426574004	0.101706794	0.752495528	Low SDI
Incidence	Male	60 to 64	62.5	0.540867384	0.116115991	0.967420823	Low SDI
Incidence	Male	15 to 19	17.5	0.706006446	0.026159403	1.390474201	Middle SDI
Incidence	Male	20 to 24	22.5	0.868036273	0.489072369	1.248429323	Middle SDI
Incidence	Male	25 to 29	27.5	0.983793455	0.70897428	1.259362569	Middle SDI
Incidence	Male	30 to 34	32.5	0.99058898	0.77021909	1.211440787	Middle SDI
Incidence	Male	35 to 39	37.5	1.049018261	0.867590778	1.230772072	Middle SDI
Incidence	Male	40 to 44	42.5	1.103103597	0.959790337	1.246620291	Middle SDI
Incidence	Male	45 to 49	47.5	1.179335877	1.063273311	1.295531731	Middle SDI
Incidence	Male	50 to 54	52.5	1.448834372	1.349667656	1.548098118	Middle SDI
Incidence	Male	55 to 59	57.5	1.838964716	1.744374734	1.933642636	Middle SDI
Incidence	Male	60 to 64	62.5	2.032145379	1.908475361	2.155965475	Middle SDI
Incidence	Male	15 to 19	17.5	0.192947656	-0.104964346	0.491748106	Global
Incidence	Male	20 to 24	22.5	0.193396409	0.029039614	0.358023257	Global
Incidence	Male	25 to 29	27.5	0.240544209	0.122977614	0.358248854	Global
Incidence	Male	30 to 34	32.5	0.150598055	0.057647001	0.243635459	Global
Incidence	Male	35 to 39	37.5	0.099251703	0.023692141	0.174868343	Global
Incidence	Male	40 to 44	42.5	0.038217246	-0.021455936	0.097926044	Global

Incidence	Male	45 to 49	47.5	-0.013828417	-0.061377971	0.033743761	Global
Incidence	Male	50 to 54	52.5	0.166539713	0.127881591	0.205212761	Global
Incidence	Male	55 to 59	57.5	0.526990447	0.493875906	0.560115899	Global
Incidence	Male	60 to 64	62.5	0.918126888	0.879564928	0.956703589	Global
Incidence	Female	15 to 19	17.5	1.147930891	0.39097008	1.910599284	High SDI
Incidence	Female	20 to 24	22.5	1.029857962	0.62368964	1.437665786	High SDI
Incidence	Female	25 to 29	27.5	0.859366781	0.575968087	1.143564024	High SDI
Incidence	Female	30 to 34	32.5	0.63564115	0.414340879	0.857429138	High SDI
Incidence	Female	35 to 39	37.5	0.47146945	0.293434186	0.649820752	High SDI
Incidence	Female	40 to 44	42.5	0.26199917	0.121275213	0.40292092	High SDI
Incidence	Female	45 to 49	47.5	0.039361946	-0.070003165	0.148846748	High SDI
Incidence	Female	50 to 54	52.5	-0.09689871	-0.181003443	-0.012723113	High SDI
Incidence	Female	55 to 59	57.5	0.045914283	-0.019768705	0.111640423	High SDI
Incidence	Female	60 to 64	62.5	0.628552761	0.563264468	0.693883441	High SDI
Incidence	Female	15 to 19	17.5	0.324020543	-0.762918959	1.422865247	Low-middle SDI
Incidence	Female	20 to 24	22.5	0.460290394	-0.168140364	1.092677056	Low-middle SDI
Incidence	Female	25 to 29	27.5	0.622559661	0.146087332	1.101298936	Low-middle SDI
Incidence	Female	30 to 34	32.5	0.733571047	0.337227842	1.131479853	Low-middle SDI
Incidence	Female	35 to 39	37.5	0.821633526	0.487570917	1.156806699	Low-middle SDI
Incidence	Female	40 to 44	42.5	0.929513856	0.652410394	1.207380204	Low-middle SDI
Incidence	Female	45 to 49	47.5	1.066579272	0.83213819	1.301565445	Low-middle SDI
Incidence	Female	50 to 54	52.5	1.264853595	1.0603265	1.469794615	Low-middle SDI
Incidence	Female	55 to 59	57.5	1.390034135	1.196715744	1.583721827	Low-middle SDI
Incidence	Female	60 to 64	62.5	1.358200477	1.117960701	1.599011022	Low-middle SDI

Incidence	Female	15 to 19	17.5	1.171094176	0.10591938	2.24760294	High-middle SDI
Incidence	Female	20 to 24	22.5	1.017828085	0.447745952	1.591145668	High-middle SDI
Incidence	Female	25 to 29	27.5	1.037584096	0.645981167	1.430710711	High-middle SDI
Incidence	Female	30 to 34	32.5	1.045309603	0.745787995	1.345721702	High-middle SDI
Incidence	Female	35 to 39	37.5	1.027260204	0.789487771	1.265593565	High-middle SDI
Incidence	Female	40 to 44	42.5	0.786871969	0.608463979	0.965596328	High-middle SDI
Incidence	Female	45 to 49	47.5	0.407252672	0.270601141	0.544090435	High-middle SDI
Incidence	Female	50 to 54	52.5	0.323320551	0.217276848	0.429476463	High-middle SDI
Incidence	Female	55 to 59	57.5	0.663384573	0.580366254	0.746471414	High-middle SDI
Incidence	Female	60 to 64	62.5	1.209239078	1.120233363	1.298323135	High-middle SDI
Incidence	Female	15 to 19	17.5	0.182067747	-1.619522292	2.016649358	Low SDI
Incidence	Female	20 to 24	22.5	0.158686827	-0.951799955	1.281623919	Low SDI
Incidence	Female	25 to 29	27.5	0.24105624	-0.635870445	1.125722141	Low SDI
Incidence	Female	30 to 34	32.5	0.347243507	-0.407200483	1.107402627	Low SDI
Incidence	Female	35 to 39	37.5	0.459802689	-0.195794285	1.119706168	Low SDI
Incidence	Female	40 to 44	42.5	0.555156607	-0.009352542	1.122852759	Low SDI
Incidence	Female	45 to 49	47.5	0.633743763	0.142168887	1.127731667	Low SDI
Incidence	Female	50 to 54	52.5	0.667197892	0.23032625	1.105973716	Low SDI
Incidence	Female	55 to 59	57.5	0.693061662	0.27627593	1.111579712	Low SDI
Incidence	Female	60 to 64	62.5	0.739244479	0.229332887	1.25175022	Low SDI
Incidence	Female	15 to 19	17.5	0.724394538	-0.181119658	1.638123172	Middle SDI
Incidence	Female	20 to 24	22.5	0.871986408	0.371656924	1.374809919	Middle SDI
Incidence	Female	25 to 29	27.5	1.023649658	0.662329907	1.386266338	Middle SDI
Incidence	Female	30 to 34	32.5	1.114804465	0.825873468	1.404563436	Middle SDI

Incidence	Female	35 to 39	37.5	1.206523671		0.968676636	1.444930991	Middle SDI
Incidence	Female	40 to 44	42.5	1.242128625		1.052667167	1.431945299	Middle SDI
Incidence	Female	45 to 49	47.5	1.258515608		1.103588788	1.413679831	Middle SDI
Incidence	Female	50 to 54	52.5	1.448798548		1.315962429	1.58180883	Middle SDI
Incidence	Female	55 to 59	57.5	1.763317472		1.638592446	1.888195553	Middle SDI
Incidence	Female	60 to 64	62.5	1.886036468		1.729293743	2.0430207	Middle SDI
Incidence	Female	15 to 19	17.5	0.42433486		-0.039626501	0.890449676	Global
Incidence	Female	20 to 24	22.5	0.446894862		0.191927405	0.702511158	Global
Incidence	Female	25 to 29	27.5	0.493878482		0.311762496	0.676325099	Global
Incidence	Female	30 to 34	32.5	0.411928704		0.267970409	0.556093685	Global
Incidence	Female	35 to 39	37.5	0.336474802		0.219843574	0.453241761	Global
Incidence	Female	40 to 44	42.5	0.189496399		0.098060134	0.281016187	Global
Incidence	Female	45 to 49	47.5	0.000464152		-0.07167894	0.072659327	Global
Incidence	Female	50 to 54	52.5	-0.008021084		-0.065605895	0.049596909	Global
Incidence	Female	55 to 59	57.5	0.251504802		0.204360398	0.298671386	Global
Incidence	Female	60 to 64	62.5	0.660530825		0.610202151	0.710884675	Global
Incidence	Both	15 to 19	17.5	0.78339549		0.341115024	1.227625427	High SDI
Incidence	Both	20 to 24	22.5	0.675879943		0.439283274	0.913033945	High SDI
Incidence	Both	25 to 29	27.5	0.501152006		0.336044135	0.66653157	High SDI
Incidence	Both	30 to 34	32.5	0.306895309		0.177918675	0.436037998	High SDI
Incidence	Both	35 to 39	37.5	0.220022438		0.115793888	0.324359499	High SDI
Incidence	Both	40 to 44	42.5	0.140321541		0.057158084	0.22355412	High SDI
Incidence	Both	45 to 49	47.5	0.080495877		0.01504571	0.145988876	High SDI
Incidence	Both	50 to 54	52.5	0.106979993		0.055895091	0.158090978	High SDI

Incidence	Both	55 to 59	57.5	0.294868239	0.253733229	0.336020126	High SDI
Incidence	Both	60 to 64	62.5	0.804521272	0.760810858	0.848250648	High SDI
Incidence	Both	15 to 19	17.5	0.404321772	-0.220785085	1.033344862	Low-middle SDI
Incidence	Both	20 to 24	22.5	0.500163179	0.135226513	0.866429834	Low-middle SDI
Incidence	Both	25 to 29	27.5	0.609486398	0.331310845	0.888433212	Low-middle SDI
Incidence	Both	30 to 34	32.5	0.687119912	0.455396093	0.919378255	Low-middle SDI
Incidence	Both	35 to 39	37.5	0.757758878	0.561982166	0.953916732	Low-middle SDI
Incidence	Both	40 to 44	42.5	0.854333192	0.69173482	1.01719413	Low-middle SDI
Incidence	Both	45 to 49	47.5	0.995614126	0.8580563	1.133359564	Low-middle SDI
Incidence	Both	50 to 54	52.5	1.217221154	1.096191421	1.338395779	Low-middle SDI
Incidence	Both	55 to 59	57.5	1.396314898	1.278984293	1.513781429	Low-middle SDI
Incidence	Both	60 to 64	62.5	1.443559476	1.293560919	1.593780156	Low-middle SDI
Incidence	Both	15 to 19	17.5	1.031196334	0.455989786	1.609696488	High-middle SDI
Incidence	Both	20 to 24	22.5	0.886589936	0.57780345	1.196324436	High-middle SDI
Incidence	Both	25 to 29	27.5	0.985478345	0.773089111	1.19831521	High-middle SDI
Incidence	Both	30 to 34	32.5	1.013405164	0.850793165	1.176279359	High-middle SDI
Incidence	Both	35 to 39	37.5	1.005719619	0.876431707	1.135173231	High-middle SDI
Incidence	Both	40 to 44	42.5	0.796323583	0.698897927	0.893843498	High-middle SDI
Incidence	Both	45 to 49	47.5	0.443386618	0.36796535	0.51886456	High-middle SDI
Incidence	Both	50 to 54	52.5	0.406126361	0.346266917	0.466021512	High-middle SDI
Incidence	Both	55 to 59	57.5	0.740463297	0.691541666	0.789408696	High-middle SDI
Incidence	Both	60 to 64	62.5	1.142148929	1.086167973	1.198160888	High-middle SDI
Incidence	Both	15 to 19	17.5	0.224741089	-0.704718134	1.162900569	Low SDI
Incidence	Both	20 to 24	22.5	0.227412805	-0.350895972	0.80907777	Low SDI

Incidence	Both	25 to 29	27.5	0.253028504	-0.207168933	0.715348154	Low SDI
Incidence	Both	30 to 34	32.5	0.283237977	-0.114628436	0.682689185	Low SDI
Incidence	Both	35 to 39	37.5	0.334878187	-0.01288925	0.683855202	Low SDI
Incidence	Both	40 to 44	42.5	0.379240156	0.076849954	0.682544054	Low SDI
Incidence	Both	45 to 49	47.5	0.415571778	0.148190772	0.683666652	Low SDI
Incidence	Both	50 to 54	52.5	0.458979174	0.210107492	0.708468929	Low SDI
Incidence	Both	55 to 59	57.5	0.514805363	0.259211826	0.771050492	Low SDI
Incidence	Both	60 to 64	62.5	0.588148141	0.262044434	0.915312506	Low SDI
Incidence	Both	15 to 19	17.5	0.700978137	0.155686582	1.249238498	Middle SDI
Incidence	Both	20 to 24	22.5	0.871573976	0.569309299	1.17474712	Middle SDI
Incidence	Both	25 to 29	27.5	0.997825078	0.779009103	1.217116156	Middle SDI
Incidence	Both	30 to 34	32.5	1.027896724	0.852631922	1.203466106	Middle SDI
Incidence	Both	35 to 39	37.5	1.089313711	0.94507135	1.233762182	Middle SDI
Incidence	Both	40 to 44	42.5	1.125875741	1.011596537	1.240284234	Middle SDI
Incidence	Both	45 to 49	47.5	1.17161509	1.078755788	1.2645597	Middle SDI
Incidence	Both	50 to 54	52.5	1.405240818	1.325810261	1.484733642	Middle SDI
Incidence	Both	55 to 59	57.5	1.772658442	1.697322433	1.848050259	Middle SDI
Incidence	Both	60 to 64	62.5	1.94764424	1.850568364	2.04481264	Middle SDI
Incidence	Both	15 to 19	17.5	0.282801347	-0.011829639	0.578300509	Global
Incidence	Both	20 to 24	22.5	0.286253706	0.123936427	0.448834127	Global
Incidence	Both	25 to 29	27.5	0.325215218	0.209172862	0.44139195	Global
Incidence	Both	30 to 34	32.5	0.229451209	0.137724146	0.321262296	Global
Incidence	Both	35 to 39	37.5	0.163048143	0.088575244	0.237576455	Global
Incidence	Both	40 to 44	42.5	0.070660497	0.011990121	0.12936529	Global

Incidence	Both	45 to 49	47.5	-0.031745711		-0.078344286	0.014874595	Global
Incidence	Both	50 to 54	52.5	0.082553884		0.044905209	0.120216726	Global
Incidence	Both	55 to 59	57.5	0.412659271		0.380925839	0.444402734	Global
Incidence	Both	60 to 64	62.5	0.809366637		0.773538606	0.845207406	Global

Abbreviation: CI: confidence interval; SDI: Sociodemographic index.
