# Supplementary material for: Trends in the global burden of aortic valve calcification disease in the working-age population from 1992 to 2021
Source: Front Cardiovasc Med. 2025 Aug 12;12:1544273. doi: 10.3389/fcvm.2025.1544273 (PMC12379075; doi:10.3389/fcvm.2025.1544273)
Supplement: Supplementary file 3 [file Datasheet3.zip › Supplementary Table 6.PDF]

Supplementary

Table S6. Working-age cohort cycle model of aortic valve calcification from 1992 to 2021

Measure	Sex	Label	Age	Rate	CI Low	CI High	Location
Deaths	Male	15 to 19	17.5	0.067634032	0.053853811	0.084940364	High SDI
Deaths	Male	20 to 24	22.5	0.093619752	0.078001318	0.11236551	High SDI
Deaths	Male	25 to 29	27.5	0.130648476	0.112195993	0.152135773	High SDI
Deaths	Male	30 to 34	32.5	0.17280311	0.151246184	0.197432517	High SDI
Deaths	Male	35 to 39	37.5	0.247581743	0.219473826	0.279289428	High SDI
Deaths	Male	40 to 44	42.5	0.370988081	0.331252495	0.415490173	High SDI
Deaths	Male	45 to 49	47.5	0.515708184	0.455793883	0.583498246	High SDI
Deaths	Male	50 to 54	52.5	0.792224734	0.701118017	0.895170305	High SDI
Deaths	Male	55 to 59	57.5	1.211637729	1.072656758	1.368626054	High SDI
Deaths	Male	60 to 64	62.5	2.004849418	1.774709223	2.264833663	High SDI
Deaths	Male	15 to 19	17.5	0.062058059	0.052524898	0.073321469	Low-middle SDI
Deaths	Male	20 to 24	22.5	0.076125533	0.065506491	0.088465993	Low-middle SDI
Deaths	Male	25 to 29	27.5	0.093170292	0.081007823	0.107158828	Low-middle SDI
Deaths	Male	30 to 34	32.5	0.116422137	0.102072441	0.132789163	Low-middle SDI
Deaths	Male	35 to 39	37.5	0.200746817	0.178530888	0.22572724	Low-middle SDI
Deaths	Male	40 to 44	42.5	0.292629173	0.261309276	0.327702996	Low-middle SDI
Deaths	Male	45 to 49	47.5	0.440365134	0.386304296	0.501991443	Low-middle SDI
Deaths	Male	50 to 54	52.5	0.733336276	0.642062382	0.837585425	Low-middle SDI
Deaths	Male	55 to 59	57.5	1.283304087	1.12180348	1.468055154	Low-middle SDI
Deaths	Male	60 to 64	62.5	1.76717192	1.539354039	2.028705884	Low-middle SDI
Deaths	Male	15 to 19	17.5	0.05895063	0.047349529	0.073394114	High-middle SDI

Deaths	Male	20 to 24	22.5	0.065964272	0.054471884	0.079881305	High-middle SDI
Deaths	Male	25 to 29	27.5	0.081534791	0.068839609	0.096571179	High-middle SDI
Deaths	Male	30 to 34	32.5	0.105614373	0.090691422	0.122992842	High-middle SDI
Deaths	Male	35 to 39	37.5	0.152250153	0.132489047	0.174958682	High-middle SDI
Deaths	Male	40 to 44	42.5	0.22679551	0.198685404	0.258882646	High-middle SDI
Deaths	Male	45 to 49	47.5	0.322819312	0.278670305	0.373962729	High-middle SDI
Deaths	Male	50 to 54	52.5	0.530345382	0.458053755	0.614046325	High-middle SDI
Deaths	Male	55 to 59	57.5	0.902936849	0.779651269	1.04571747	High-middle SDI
Deaths	Male	60 to 64	62.5	1.444021829	1.24549984	1.67418652	High-middle SDI
Deaths	Male	15 to 19	17.5	0.060904084	0.047480691	0.078122439	Low SDI
Deaths	Male	20 to 24	22.5	0.066440756	0.052565847	0.083977989	Low SDI
Deaths	Male	25 to 29	27.5	0.081640492	0.065502569	0.10175433	Low SDI
Deaths	Male	30 to 34	32.5	0.103308096	0.083872176	0.127247952	Low SDI
Deaths	Male	35 to 39	37.5	0.205390249	0.171851642	0.245474259	Low SDI
Deaths	Male	40 to 44	42.5	0.285407507	0.239778678	0.339719303	Low SDI
Deaths	Male	45 to 49	47.5	0.444167201	0.362196578	0.544689029	Low SDI
Deaths	Male	50 to 54	52.5	0.718036932	0.582833026	0.884605045	Low SDI
Deaths	Male	55 to 59	57.5	1.274708363	1.032751034	1.573352489	Low SDI
Deaths	Male	60 to 64	62.5	1.73598861	1.39896406	2.154205774	Low SDI
Deaths	Male	15 to 19	17.5	0.059414399	0.050858198	0.069410065	Middle SDI
Deaths	Male	20 to 24	22.5	0.063328328	0.054879283	0.073078163	Middle SDI
Deaths	Male	25 to 29	27.5	0.072316834	0.063359873	0.082540009	Middle SDI
Deaths	Male	30 to 34	32.5	0.089687806	0.079343855	0.101380284	Middle SDI
Deaths	Male	35 to 39	37.5	0.137545288	0.123034546	0.153767432	Middle SDI

Deaths	Male	40 to 44	42.5	0.183676466	0.164818566	0.204692014	Middle SDI
Deaths	Male	45 to 49	47.5	0.247759586	0.219293466	0.279920846	Middle SDI
Deaths	Male	50 to 54	52.5	0.382533828	0.338421532	0.432396039	Middle SDI
Deaths	Male	55 to 59	57.5	0.605859229	0.53538425	0.685611139	Middle SDI
Deaths	Male	60 to 64	62.5	0.857759586	0.756068245	0.973128433	Middle SDI
Deaths	Male	15 to 19	17.5	0.061694273	0.056622175	0.067220719	Global
Deaths	Male	20 to 24	22.5	0.071330534	0.06606	0.077021572	Global
Deaths	Male	25 to 29	27.5	0.088043398	0.082139456	0.094371698	Global
Deaths	Male	30 to 34	32.5	0.112379632	0.105468969	0.119743104	Global
Deaths	Male	35 to 39	37.5	0.176714983	0.166907532	0.187098718	Global
Deaths	Male	40 to 44	42.5	0.254139056	0.240618594	0.268419239	Global
Deaths	Male	45 to 49	47.5	0.361405708	0.339921391	0.384247915	Global
Deaths	Male	50 to 54	52.5	0.574429453	0.54031876	0.610693577	Global
Deaths	Male	55 to 59	57.5	0.935900483	0.880182186	0.995145923	Global
Deaths	Male	60 to 64	62.5	1.43931368	1.352864243	1.531287325	Global
Deaths	Female	15 to 19	17.5	0.030436073	0.021519856	0.043046501	High SDI
Deaths	Female	20 to 24	22.5	0.037983954	0.02846028	0.050694539	High SDI
Deaths	Female	25 to 29	27.5	0.051858724	0.040647667	0.066161911	High SDI
Deaths	Female	30 to 34	32.5	0.0777086	0.063072285	0.095741364	High SDI
Deaths	Female	35 to 39	37.5	0.108803371	0.089959198	0.131594923	High SDI
Deaths	Female	40 to 44	42.5	0.151823126	0.126494209	0.182223847	High SDI
Deaths	Female	45 to 49	47.5	0.238140341	0.195587371	0.289951348	High SDI
Deaths	Female	50 to 54	52.5	0.380583448	0.313063967	0.462665066	High SDI
Deaths	Female	55 to 59	57.5	0.628623063	0.517422351	0.763722239	High SDI

Deaths	Female	60 to 64	62.5	1.168826212	0.962476568	1.419416077	High SDI
Deaths	Female	15 to 19	17.5	0.047281001	0.038558367	0.057976862	Low-middle SDI
Deaths	Female	20 to 24	22.5	0.049554427	0.04098978	0.059908621	Low-middle SDI
Deaths	Female	25 to 29	27.5	0.05073065	0.04213291	0.061082864	Low-middle SDI
Deaths	Female	30 to 34	32.5	0.077025992	0.065153668	0.091061693	Low-middle SDI
Deaths	Female	35 to 39	37.5	0.088579092	0.074936441	0.104705472	Low-middle SDI
Deaths	Female	40 to 44	42.5	0.135403022	0.115318935	0.158984979	Low-middle SDI
Deaths	Female	45 to 49	47.5	0.207233519	0.1724846	0.248982989	Low-middle SDI
Deaths	Female	50 to 54	52.5	0.332138404	0.275539926	0.40036274	Low-middle SDI
Deaths	Female	55 to 59	57.5	0.77864234	0.646966482	0.937117934	Low-middle SDI
Deaths	Female	60 to 64	62.5	1.174396161	0.971912032	1.419064996	Low-middle SDI
Deaths	Female	15 to 19	17.5	0.025041817	0.017843635	0.035143768	High-middle SDI
Deaths	Female	20 to 24	22.5	0.024196926	0.017715071	0.03305046	High-middle SDI
Deaths	Female	25 to 29	27.5	0.02764359	0.020730477	0.036862059	High-middle SDI
Deaths	Female	30 to 34	32.5	0.045442545	0.035649434	0.057925881	High-middle SDI
Deaths	Female	35 to 39	37.5	0.058212581	0.046345915	0.073117653	High-middle SDI
Deaths	Female	40 to 44	42.5	0.083663362	0.067164595	0.104214998	High-middle SDI
Deaths	Female	45 to 49	47.5	0.135198038	0.106194308	0.17212325	High-middle SDI
Deaths	Female	50 to 54	52.5	0.215594997	0.169205125	0.274703281	High-middle SDI
Deaths	Female	55 to 59	57.5	0.400009514	0.314087759	0.509435999	High-middle SDI
Deaths	Female	60 to 64	62.5	0.74440457	0.584284047	0.948405431	High-middle SDI
Deaths	Female	15 to 19	17.5	0.054454006	0.041325685	0.071752926	Low SDI
Deaths	Female	20 to 24	22.5	0.070766503	0.055174217	0.090765183	Low SDI
Deaths	Female	25 to 29	27.5	0.05507186	0.042340197	0.071631925	Low SDI

Deaths	Female	30 to 34	32.5	0.076591899	0.059854304	0.098009977	Low SDI
Deaths	Female	35 to 39	37.5	0.073814568	0.056838082	0.095861617	Low SDI
Deaths	Female	40 to 44	42.5	0.123883455	0.096919145	0.158349627	Low SDI
Deaths	Female	45 to 49	47.5	0.190905266	0.144369761	0.252440819	Low SDI
Deaths	Female	50 to 54	52.5	0.279804567	0.209819367	0.373133314	Low SDI
Deaths	Female	55 to 59	57.5	0.754156953	0.569386522	0.99888685	Low SDI
Deaths	Female	60 to 64	62.5	1.159806304	0.870601778	1.545081455	Low SDI
Deaths	Female	15 to 19	17.5	0.0396023	0.032376515	0.048440735	Middle SDI
Deaths	Female	20 to 24	22.5	0.035680082	0.029445378	0.04323491	Middle SDI
Deaths	Female	25 to 29	27.5	0.038717411	0.032343773	0.046347032	Middle SDI
Deaths	Female	30 to 34	32.5	0.058083863	0.04950924	0.068143545	Middle SDI
Deaths	Female	35 to 39	37.5	0.068898407	0.058947192	0.080529543	Middle SDI
Deaths	Female	40 to 44	42.5	0.089962171	0.077294886	0.104705403	Middle SDI
Deaths	Female	45 to 49	47.5	0.126540747	0.10685343	0.149855372	Middle SDI
Deaths	Female	50 to 54	52.5	0.192475778	0.162399951	0.228121529	Middle SDI
Deaths	Female	55 to 59	57.5	0.364631383	0.307813287	0.431937317	Middle SDI
Deaths	Female	60 to 64	62.5	0.54902651	0.46213497	0.65225557	Middle SDI
Deaths	Female	15 to 19	17.5	0.039318327	0.035168268	0.043958116	Global
Deaths	Female	20 to 24	22.5	0.04176951	0.037701897	0.046275974	Global
Deaths	Female	25 to 29	27.5	0.043007508	0.038985383	0.047444596	Global
Deaths	Female	30 to 34	32.5	0.064602897	0.059211109	0.070485664	Global
Deaths	Female	35 to 39	37.5	0.078148326	0.071813177	0.085042343	Global
Deaths	Female	40 to 44	42.5	0.111679702	0.102960849	0.121136877	Global
Deaths	Female	45 to 49	47.5	0.170027821	0.155376508	0.186060686	Global

Deaths	Female	50 to 54	52.5	0.266998587	0.243908249	0.292274845	Global
Deaths	Female	55 to 59	57.5	0.516422464	0.471993249	0.565033848	Global
Deaths	Female	60 to 64	62.5	0.87019883	0.794864007	0.952673662	Global
Deaths	Both	15 to 19	17.5	0.049335438	0.04079528	0.059663409	High SDI
Deaths	Both	20 to 24	22.5	0.066688533	0.057152449	0.077815746	High SDI
Deaths	Both	25 to 29	27.5	0.091998484	0.080834895	0.104703805	High SDI
Deaths	Both	30 to 34	32.5	0.126530498	0.113084278	0.141575534	High SDI
Deaths	Both	35 to 39	37.5	0.179923962	0.162501386	0.199214497	High SDI
Deaths	Both	40 to 44	42.5	0.264035167	0.239777646	0.290746743	High SDI
Deaths	Both	45 to 49	47.5	0.37872626	0.341082824	0.420524195	High SDI
Deaths	Both	50 to 54	52.5	0.585963516	0.528274843	0.649951907	High SDI
Deaths	Both	55 to 59	57.5	0.912856097	0.823242333	1.012224736	High SDI
Deaths	Both	60 to 64	62.5	1.561290563	1.408072784	1.731180554	High SDI
Deaths	Both	15 to 19	17.5	0.054732326	0.048109919	0.062266319	Low-middle SDI
Deaths	Both	20 to 24	22.5	0.062914303	0.055930383	0.070770291	Low-middle SDI
Deaths	Both	25 to 29	27.5	0.071564633	0.063998542	0.080025209	Low-middle SDI
Deaths	Both	30 to 34	32.5	0.096260726	0.086811545	0.106738421	Low-middle SDI
Deaths	Both	35 to 39	37.5	0.144050427	0.130869091	0.158559408	Low-middle SDI
Deaths	Both	40 to 44	42.5	0.213958634	0.195062214	0.234685622	Low-middle SDI
Deaths	Both	45 to 49	47.5	0.323780649	0.291050019	0.360192069	Low-middle SDI
Deaths	Both	50 to 54	52.5	0.529476937	0.475141377	0.590026127	Low-middle SDI
Deaths	Both	55 to 59	57.5	1.022799806	0.91740485	1.140302935	Low-middle SDI
Deaths	Both	60 to 64	62.5	1.454364053	1.301116086	1.625661863	Low-middle SDI
Deaths	Both	15 to 19	17.5	0.042128594	0.035046973	0.050641132	High-middle SDI

Deaths	Both	20 to 24	22.5	0.045137311	0.038335788	0.053145557	High-middle SDI
Deaths	Both	25 to 29	27.5	0.054970583	0.047510626	0.063601877	High-middle SDI
Deaths	Both	30 to 34	32.5	0.076077396	0.0668821	0.08653691	High-middle SDI
Deaths	Both	35 to 39	37.5	0.10604525	0.09416269	0.119427292	High-middle SDI
Deaths	Both	40 to 44	42.5	0.156132712	0.139402944	0.174870223	High-middle SDI
Deaths	Both	45 to 49	47.5	0.228914603	0.201896141	0.259548771	High-middle SDI
Deaths	Both	50 to 54	52.5	0.371520997	0.327743598	0.421145834	High-middle SDI
Deaths	Both	55 to 59	57.5	0.642791033	0.567010376	0.728699737	High-middle SDI
Deaths	Both	60 to 64	62.5	1.070276602	0.943476916	1.21411768	High-middle SDI
Deaths	Both	15 to 19	17.5	0.057770354	0.048053762	0.069451666	Low SDI
Deaths	Both	20 to 24	22.5	0.069233108	0.058475624	0.081969596	Low SDI
Deaths	Both	25 to 29	27.5	0.068331665	0.057748309	0.080854599	Low SDI
Deaths	Both	30 to 34	32.5	0.089621391	0.076463815	0.105043069	Low SDI
Deaths	Both	35 to 39	37.5	0.139480576	0.120535924	0.161402763	Low SDI
Deaths	Both	40 to 44	42.5	0.206198769	0.179009217	0.237518117	Low SDI
Deaths	Both	45 to 49	47.5	0.319422649	0.271052667	0.376424369	Low SDI
Deaths	Both	50 to 54	52.5	0.499578878	0.42217748	0.591170934	Low SDI
Deaths	Both	55 to 59	57.5	1.026412922	0.867775223	1.214051126	Low SDI
Deaths	Both	60 to 64	62.5	1.472443496	1.240209146	1.748164699	Low SDI
Deaths	Both	15 to 19	17.5	0.049377067	0.043651921	0.055853091	Middle SDI
Deaths	Both	20 to 24	22.5	0.049442899	0.044078105	0.055460647	Middle SDI
Deaths	Both	25 to 29	27.5	0.05550467	0.049892139	0.061748574	Middle SDI
Deaths	Both	30 to 34	32.5	0.073922081	0.067076621	0.081466149	Middle SDI
Deaths	Both	35 to 39	37.5	0.103311939	0.094346317	0.113129554	Middle SDI

Deaths	Both	40 to 44	42.5	0.13701699	0.125451718	0.149648453	Middle SDI
Deaths	Both	45 to 49	47.5	0.186950609	0.169326721	0.206408829	Middle SDI
Deaths	Both	50 to 54	52.5	0.286510024	0.259394633	0.316459877	Middle SDI
Deaths	Both	55 to 59	57.5	0.481651554	0.435862182	0.532251314	Middle SDI
Deaths	Both	60 to 64	62.5	0.695005605	0.627740397	0.769478582	Middle SDI
Deaths	Both	15 to 19	17.5	0.050712394	0.047381288	0.054277691	Global
Deaths	Both	20 to 24	22.5	0.056613411	0.053241149	0.06019927	Global
Deaths	Both	25 to 29	27.5	0.065570588	0.0619592	0.069392473	Global
Deaths	Both	30 to 34	32.5	0.088511703	0.084089092	0.093166919	Global
Deaths	Both	35 to 39	37.5	0.127723318	0.121823667	0.133908677	Global
Deaths	Both	40 to 44	42.5	0.183422509	0.175292051	0.191930078	Global
Deaths	Both	45 to 49	47.5	0.265830024	0.252698212	0.279644249	Global
Deaths	Both	50 to 54	52.5	0.419324714	0.398603657	0.441122937	Global
Deaths	Both	55 to 59	57.5	0.718210083	0.682723789	0.755540866	Global
Deaths	Both	60 to 64	62.5	1.134550216	1.078057368	1.194003427	Global
DALYs (Disability-Adjusted Life Years)	Male	15 to 19	17.5	4.884559545	4.57753133	5.212181027	High SDI
DALYs (Disability-Adjusted Life Years)	Male	20 to 24	22.5	6.319557557	5.987278245	6.670277559	High SDI
DALYs (Disability-Adjusted Life Years)	Male	25 to 29	27.5	8.183131556	7.81040538	8.573644875	High SDI
DALYs (Disability-Adjusted Life Years)	Male	30 to 34	32.5	10.00831992	9.596286008	10.43804524	High SDI
DALYs (Disability-Adjusted Life Years)	Male	35 to 39	37.5	13.13895639	12.63656407	13.66132234	High SDI
DALYs (Disability-Adjusted Life Years)	Male	40 to 44	42.5	17.89326799	17.23783554	18.57362189	High SDI
DALYs (Disability-Adjusted Life Years)	Male	45 to 49	47.5	22.49383085	21.59347828	23.43172414	High SDI
DALYs (Disability-Adjusted Life Years)	Male	50 to 54	52.5	30.98146954	29.74775862	32.26634541	High SDI
DALYs (Disability-Adjusted Life Years)	Male	55 to 59	57.5	42.2824482	40.59492645	44.04011984	High SDI

DALYs (Disability-Adjusted Life Years)	Male	60 to 64	62.5	61.7464152	59.2660611	64.33057502	High SDI
DALYs (Disability-Adjusted Life Years)	Male	15 to 19	17.5	4.482888942	4.33403467	4.636855677	Low-middle SDI
DALYs (Disability-Adjusted Life Years)	Male	20 to 24	22.5	5.126821688	4.969806649	5.288797427	Low-middle SDI
DALYs (Disability-Adjusted Life Years)	Male	25 to 29	27.5	5.822005869	5.651271787	5.997898104	Low-middle SDI
DALYs (Disability-Adjusted Life Years)	Male	30 to 34	32.5	6.717823785	6.526930811	6.9142998	Low-middle SDI
DALYs (Disability-Adjusted Life Years)	Male	35 to 39	37.5	10.60297711	10.32567556	10.88772575	Low-middle SDI
DALYs (Disability-Adjusted Life Years)	Male	40 to 44	42.5	14.03465867	13.67065037	14.40835941	Low-middle SDI
DALYs (Disability-Adjusted Life Years)	Male	45 to 49	47.5	19.05710534	18.48267364	19.64939007	Low-middle SDI
DALYs (Disability-Adjusted Life Years)	Male	50 to 54	52.5	28.33973166	27.46181569	29.24571338	Low-middle SDI
DALYs (Disability-Adjusted Life Years)	Male	55 to 59	57.5	43.82177196	42.43384551	45.25509471	Low-middle SDI
DALYs (Disability-Adjusted Life Years)	Male	60 to 64	62.5	52.48775347	50.74623694	54.28903561	Low-middle SDI
DALYs (Disability-Adjusted Life Years)	Male	15 to 19	17.5	4.235879091	4.028679754	4.453734912	High-middle SDI
DALYs (Disability-Adjusted Life Years)	Male	20 to 24	22.5	4.419647255	4.224927382	4.623341443	High-middle SDI
DALYs (Disability-Adjusted Life Years)	Male	25 to 29	27.5	5.116893504	4.911309099	5.331083547	High-middle SDI
DALYs (Disability-Adjusted Life Years)	Male	30 to 34	32.5	6.101890084	5.873900712	6.338728628	High-middle SDI
DALYs (Disability-Adjusted Life Years)	Male	35 to 39	37.5	8.050598317	7.767702027	8.343797564	High-middle SDI
DALYs (Disability-Adjusted Life Years)	Male	40 to 44	42.5	10.92792605	10.55486978	11.3141678	High-middle SDI
DALYs (Disability-Adjusted Life Years)	Male	45 to 49	47.5	14.075068	13.53919281	14.6321529	High-middle SDI
DALYs (Disability-Adjusted Life Years)	Male	50 to 54	52.5	20.87733453	20.07927823	21.70710978	High-middle SDI
DALYs (Disability-Adjusted Life Years)	Male	55 to 59	57.5	31.84312975	30.61667377	33.11871564	High-middle SDI
DALYs (Disability-Adjusted Life Years)	Male	60 to 64	62.5	45.42588993	43.64814718	47.27603827	High-middle SDI
DALYs (Disability-Adjusted Life Years)	Male	15 to 19	17.5	4.423394882	4.291559721	4.559279972	Low SDI
DALYs (Disability-Adjusted Life Years)	Male	20 to 24	22.5	4.518231949	4.388770894	4.651511877	Low SDI
DALYs (Disability-Adjusted Life Years)	Male	25 to 29	27.5	5.102378813	4.960302767	5.248524289	Low SDI

DALYs (Disability-Adjusted Life Years)	Male	30 to 34	32.5	5.907076134	5.746252682	6.072400639	Low SDI
DALYs (Disability-Adjusted Life Years)	Male	35 to 39	37.5	10.8861169	10.62541846	11.15321166	Low SDI
DALYs (Disability-Adjusted Life Years)	Male	40 to 44	42.5	13.783138	13.45113798	14.12333242	Low SDI
DALYs (Disability-Adjusted Life Years)	Male	45 to 49	47.5	19.26179884	18.71559881	19.82393929	Low SDI
DALYs (Disability-Adjusted Life Years)	Male	50 to 54	52.5	27.74152052	26.92544142	28.58233404	Low SDI
DALYs (Disability-Adjusted Life Years)	Male	55 to 59	57.5	43.27052284	41.97205145	44.60916447	Low SDI
DALYs (Disability-Adjusted Life Years)	Male	60 to 64	62.5	51.19382695	49.58416679	52.85574182	Low SDI
DALYs (Disability-Adjusted Life Years)	Male	15 to 19	17.5	4.289898607	4.141031538	4.444117339	Middle SDI
DALYs (Disability-Adjusted Life Years)	Male	20 to 24	22.5	4.268024529	4.128176391	4.41261023	Middle SDI
DALYs (Disability-Adjusted Life Years)	Male	25 to 29	27.5	4.541080359	4.399514367	4.687201611	Middle SDI
DALYs (Disability-Adjusted Life Years)	Male	30 to 34	32.5	5.178295577	5.024001984	5.337327725	Middle SDI
DALYs (Disability-Adjusted Life Years)	Male	35 to 39	37.5	7.267961049	7.065131573	7.476613459	Middle SDI
DALYs (Disability-Adjusted Life Years)	Male	40 to 44	42.5	8.825449047	8.580412402	9.07748337	Middle SDI
DALYs (Disability-Adjusted Life Years)	Male	45 to 49	47.5	10.74831776	10.4103004	11.09731038	Middle SDI
DALYs (Disability-Adjusted Life Years)	Male	50 to 54	52.5	14.90343968	14.42759062	15.39498314	Middle SDI
DALYs (Disability-Adjusted Life Years)	Male	55 to 59	57.5	21.00087302	20.31668372	21.70810324	Middle SDI
DALYs (Disability-Adjusted Life Years)	Male	60 to 64	62.5	26.20737264	25.32061995	27.12518028	Middle SDI
DALYs (Disability-Adjusted Life Years)	Male	15 to 19	17.5	4.453688719	4.291771581	4.621714561	Global
DALYs (Disability-Adjusted Life Years)	Male	20 to 24	22.5	4.814770605	4.653808604	4.981299823	Global
DALYs (Disability-Adjusted Life Years)	Male	25 to 29	27.5	5.514894173	5.342887182	5.692438696	Global
DALYs (Disability-Adjusted Life Years)	Male	30 to 34	32.5	6.493955731	6.302801097	6.690907802	Global
DALYs (Disability-Adjusted Life Years)	Male	35 to 39	37.5	9.351142978	9.096241107	9.613187906	Global
DALYs (Disability-Adjusted Life Years)	Male	40 to 44	42.5	12.2172783	11.89093977	12.55257297	Global
DALYs (Disability-Adjusted Life Years)	Male	45 to 49	47.5	15.69513225	15.22322762	16.18166545	Global

DALYs (Disability-Adjusted Life Years)	Male	50 to 54	52.5	22.37600138	21.69772875	23.07547687	Global
DALYs (Disability-Adjusted Life Years)	Male	55 to 59	57.5	32.45060861	31.45666362	33.47595956	Global
DALYs (Disability-Adjusted Life Years)	Male	60 to 64	62.5	44.04343756	42.66651437	45.46479648	Global
DALYs (Disability-Adjusted Life Years)	Female	15 to 19	17.5	2.164436606	2.030152196	2.307603259	High SDI
DALYs (Disability-Adjusted Life Years)	Female	20 to 24	22.5	2.627781087	2.4880776	2.775328808	High SDI
DALYs (Disability-Adjusted Life Years)	Female	25 to 29	27.5	3.284765742	3.131286419	3.445767822	High SDI
DALYs (Disability-Adjusted Life Years)	Female	30 to 34	32.5	4.519710837	4.332505042	4.715005719	High SDI
DALYs (Disability-Adjusted Life Years)	Female	35 to 39	37.5	5.792458958	5.567687242	6.026304878	High SDI
DALYs (Disability-Adjusted Life Years)	Female	40 to 44	42.5	7.339862668	7.060910429	7.629835349	High SDI
DALYs (Disability-Adjusted Life Years)	Female	45 to 49	47.5	10.43470627	10.00699526	10.88069815	High SDI
DALYs (Disability-Adjusted Life Years)	Female	50 to 54	52.5	14.95456769	14.34307846	15.59212658	High SDI
DALYs (Disability-Adjusted Life Years)	Female	55 to 59	57.5	22.03739668	21.13591882	22.97732389	High SDI
DALYs (Disability-Adjusted Life Years)	Female	60 to 64	62.5	36.1830273	34.70146095	37.72784858	High SDI
DALYs (Disability-Adjusted Life Years)	Female	15 to 19	17.5	3.428395355	3.30239182	3.559206583	Low-middle SDI
DALYs (Disability-Adjusted Life Years)	Female	20 to 24	22.5	3.353042013	3.235602027	3.474744622	Low-middle SDI
DALYs (Disability-Adjusted Life Years)	Female	25 to 29	27.5	3.18472408	3.072802659	3.300722042	Low-middle SDI
DALYs (Disability-Adjusted Life Years)	Female	30 to 34	32.5	4.426323971	4.281237758	4.576326989	Low-middle SDI
DALYs (Disability-Adjusted Life Years)	Female	35 to 39	37.5	4.674650693	4.516979389	4.83782573	Low-middle SDI
DALYs (Disability-Adjusted Life Years)	Female	40 to 44	42.5	6.513981113	6.297623442	6.737771848	Low-middle SDI
DALYs (Disability-Adjusted Life Years)	Female	45 to 49	47.5	8.996952488	8.653655109	9.353868746	Low-middle SDI
DALYs (Disability-Adjusted Life Years)	Female	50 to 54	52.5	12.86320825	12.35665642	13.39052579	Low-middle SDI
DALYs (Disability-Adjusted Life Years)	Female	55 to 59	57.5	26.53427382	25.49520264	27.61569293	Low-middle SDI
DALYs (Disability-Adjusted Life Years)	Female	60 to 64	62.5	34.80754899	33.39265816	36.28239061	Low-middle SDI
DALYs (Disability-Adjusted Life Years)	Female	15 to 19	17.5	1.828467033	1.740385499	1.921006405	High-middle SDI

DALYs (Disability-Adjusted Life Years)	Female	20 to 24	22.5	1.661993661	1.5861942	1.74141535	High-middle SDI
DALYs (Disability-Adjusted Life Years)	Female	25 to 29	27.5	1.763641731	1.687282441	1.843456719	High-middle SDI
DALYs (Disability-Adjusted Life Years)	Female	30 to 34	32.5	2.640134614	2.539968065	2.744251345	High-middle SDI
DALYs (Disability-Adjusted Life Years)	Female	35 to 39	37.5	3.094561153	2.980658582	3.212816384	High-middle SDI
DALYs (Disability-Adjusted Life Years)	Female	40 to 44	42.5	4.045440392	3.898780651	4.197617006	High-middle SDI
DALYs (Disability-Adjusted Life Years)	Female	45 to 49	47.5	5.935017805	5.698516875	6.181334043	High-middle SDI
DALYs (Disability-Adjusted Life Years)	Female	50 to 54	52.5	8.555659634	8.210909932	8.914884267	High-middle SDI
DALYs (Disability-Adjusted Life Years)	Female	55 to 59	57.5	14.31318127	13.7357682	14.91486717	High-middle SDI
DALYs (Disability-Adjusted Life Years)	Female	60 to 64	62.5	23.75817851	22.79386002	24.76329351	High-middle SDI
DALYs (Disability-Adjusted Life Years)	Female	15 to 19	17.5	3.964430522	3.833220179	4.100132169	Low SDI
DALYs (Disability-Adjusted Life Years)	Female	20 to 24	22.5	4.830854815	4.683880703	4.982440785	Low SDI
DALYs (Disability-Adjusted Life Years)	Female	25 to 29	27.5	3.502473419	3.387653141	3.621185386	Low SDI
DALYs (Disability-Adjusted Life Years)	Female	30 to 34	32.5	4.455994201	4.314609666	4.602011735	Low SDI
DALYs (Disability-Adjusted Life Years)	Female	35 to 39	37.5	4.000446749	3.862374414	4.143454899	Low SDI
DALYs (Disability-Adjusted Life Years)	Female	40 to 44	42.5	6.057033978	5.854408235	6.266672759	Low SDI
DALYs (Disability-Adjusted Life Years)	Female	45 to 49	47.5	8.420191171	8.097768655	8.755451332	Low SDI
DALYs (Disability-Adjusted Life Years)	Female	50 to 54	52.5	11.0405221	10.59698747	11.50262077	Low SDI
DALYs (Disability-Adjusted Life Years)	Female	55 to 59	57.5	26.00877288	24.98811064	27.07112501	Low SDI
DALYs (Disability-Adjusted Life Years)	Female	60 to 64	62.5	34.50646485	33.10078748	35.97183655	Low SDI
DALYs (Disability-Adjusted Life Years)	Female	15 to 19	17.5	2.823726027	2.685785359	2.968751262	Middle SDI
DALYs (Disability-Adjusted Life Years)	Female	20 to 24	22.5	2.389559141	2.27534463	2.509506828	Middle SDI
DALYs (Disability-Adjusted Life Years)	Female	25 to 29	27.5	2.40374087	2.292910705	2.519928123	Middle SDI
DALYs (Disability-Adjusted Life Years)	Female	30 to 34	32.5	3.339646105	3.198479011	3.487043706	Middle SDI
DALYs (Disability-Adjusted Life Years)	Female	35 to 39	37.5	3.643755122	3.489393171	3.804945657	Middle SDI

DALYs (Disability-Adjusted Life Years)	Female	40 to 44	42.5	4.329111034	4.14614583	4.520150307	Middle SDI
DALYs (Disability-Adjusted Life Years)	Female	45 to 49	47.5	5.503351205	5.243168401	5.776445113	Middle SDI
DALYs (Disability-Adjusted Life Years)	Female	50 to 54	52.5	7.527659632	7.165952522	7.907624194	Middle SDI
DALYs (Disability-Adjusted Life Years)	Female	55 to 59	57.5	12.65399245	12.04403463	13.29484097	Middle SDI
DALYs (Disability-Adjusted Life Years)	Female	60 to 64	62.5	16.74305745	15.91033575	17.61936247	Middle SDI
DALYs (Disability-Adjusted Life Years)	Female	15 to 19	17.5	2.841112702	2.735553118	2.950745621	Global
DALYs (Disability-Adjusted Life Years)	Female	20 to 24	22.5	2.818381743	2.719699364	2.920644743	Global
DALYs (Disability-Adjusted Life Years)	Female	25 to 29	27.5	2.70383664	2.610722218	2.800272094	Global
DALYs (Disability-Adjusted Life Years)	Female	30 to 34	32.5	3.735274413	3.617282898	3.857114673	Global
DALYs (Disability-Adjusted Life Years)	Female	35 to 39	37.5	4.13817504	4.00770539	4.272892091	Global
DALYs (Disability-Adjusted Life Years)	Female	40 to 44	42.5	5.377650884	5.210598827	5.550058639	Global
DALYs (Disability-Adjusted Life Years)	Female	45 to 49	47.5	7.397036018	7.141639625	7.661565793	Global
DALYs (Disability-Adjusted Life Years)	Female	50 to 54	52.5	10.43583224	10.07056318	10.81434997	Global
DALYs (Disability-Adjusted Life Years)	Female	55 to 59	57.5	17.90890468	17.28287117	18.55761485	Global
DALYs (Disability-Adjusted Life Years)	Female	60 to 64	62.5	26.5869929	25.64332291	27.56538979	Global
DALYs (Disability-Adjusted Life Years)	Both	15 to 19	17.5	3.56291761	3.381553269	3.754009145	High SDI
DALYs (Disability-Adjusted Life Years)	Both	20 to 24	22.5	4.524686988	4.33081666	4.727235981	High SDI
DALYs (Disability-Adjusted Life Years)	Both	25 to 29	27.5	5.797506519	5.581575878	6.021790722	High SDI
DALYs (Disability-Adjusted Life Years)	Both	30 to 34	32.5	7.342515244	7.096820558	7.596715974	High SDI
DALYs (Disability-Adjusted Life Years)	Both	35 to 39	37.5	9.573023278	9.274830135	9.880803568	High SDI
DALYs (Disability-Adjusted Life Years)	Both	40 to 44	42.5	12.77675449	12.39323162	13.1721459	High SDI
DALYs (Disability-Adjusted Life Years)	Both	45 to 49	47.5	16.57121914	16.02946065	17.13128781	High SDI
DALYs (Disability-Adjusted Life Years)	Both	50 to 54	52.5	23.00609039	22.25735051	23.78001796	High SDI
DALYs (Disability-Adjusted Life Years)	Both	55 to 59	57.5	31.99142792	30.9486778	33.06931129	High SDI

DALYs (Disability-Adjusted Life Years)	Both	60 to 64	62.5	48.30025201	46.71889825	49.93513186	High SDI
DALYs (Disability-Adjusted Life Years)	Both	15 to 19	17.5	3.95552838	3.832897951	4.082082269	Low-middle SDI
DALYs (Disability-Adjusted Life Years)	Both	20 to 24	22.5	4.231503712	4.108900969	4.357764717	Low-middle SDI
DALYs (Disability-Adjusted Life Years)	Both	25 to 29	27.5	4.483461363	4.356843666	4.613758798	Low-middle SDI
DALYs (Disability-Adjusted Life Years)	Both	30 to 34	32.5	5.557715919	5.407914374	5.711667031	Low-middle SDI
DALYs (Disability-Adjusted Life Years)	Both	35 to 39	37.5	7.616444179	7.41984834	7.818249009	Low-middle SDI
DALYs (Disability-Adjusted Life Years)	Both	40 to 44	42.5	10.26436924	10.00208322	10.53353323	Low-middle SDI
DALYs (Disability-Adjusted Life Years)	Both	45 to 49	47.5	14.00280687	13.58842189	14.42982871	Low-middle SDI
DALYs (Disability-Adjusted Life Years)	Both	50 to 54	52.5	20.49130748	19.86718396	21.13503771	Low-middle SDI
DALYs (Disability-Adjusted Life Years)	Both	55 to 59	57.5	34.84006577	33.76590598	35.94839669	Low-middle SDI
DALYs (Disability-Adjusted Life Years)	Both	60 to 64	62.5	43.06466717	41.67962299	44.49573737	Low-middle SDI
DALYs (Disability-Adjusted Life Years)	Both	15 to 19	17.5	3.050080669	2.911540275	3.195213259	High-middle SDI
DALYs (Disability-Adjusted Life Years)	Both	20 to 24	22.5	3.052071095	2.925358918	3.184271821	High-middle SDI
DALYs (Disability-Adjusted Life Years)	Both	25 to 29	27.5	3.463695575	3.331178981	3.60148377	High-middle SDI
DALYs (Disability-Adjusted Life Years)	Both	30 to 34	32.5	4.399188609	4.245201723	4.558761086	High-middle SDI
DALYs (Disability-Adjusted Life Years)	Both	35 to 39	37.5	5.610826279	5.424494454	5.803558618	High-middle SDI
DALYs (Disability-Adjusted Life Years)	Both	40 to 44	42.5	7.526819262	7.28311839	7.778674624	High-middle SDI
DALYs (Disability-Adjusted Life Years)	Both	45 to 49	47.5	10.00091457	9.641141648	10.37411293	High-middle SDI
DALYs (Disability-Adjusted Life Years)	Both	50 to 54	52.5	14.63988765	14.11009385	15.1895737	High-middle SDI
DALYs (Disability-Adjusted Life Years)	Both	55 to 59	57.5	22.75935341	21.93117228	23.61880892	High-middle SDI
DALYs (Disability-Adjusted Life Years)	Both	60 to 64	62.5	33.80213909	32.55683882	35.09507214	High-middle SDI
DALYs (Disability-Adjusted Life Years)	Both	15 to 19	17.5	4.201396243	4.105128126	4.299921914	Low SDI
DALYs (Disability-Adjusted Life Years)	Both	20 to 24	22.5	4.676370096	4.57594967	4.778994274	Low SDI
DALYs (Disability-Adjusted Life Years)	Both	25 to 29	27.5	4.288065864	4.193915246	4.384330101	Low SDI

DALYs (Disability-Adjusted Life Years)	Both	30 to 34	32.5	5.180604324	5.069919277	5.293705817	Low SDI
DALYs (Disability-Adjusted Life Years)	Both	35 to 39	37.5	7.373529629	7.224078306	7.526072792	Low SDI
DALYs (Disability-Adjusted Life Years)	Both	40 to 44	42.5	9.888927458	9.689106559	10.09286931	Low SDI
DALYs (Disability-Adjusted Life Years)	Both	45 to 49	47.5	13.81477046	13.48914301	14.14825854	Low SDI
DALYs (Disability-Adjusted Life Years)	Both	50 to 54	52.5	19.27876155	18.80547346	19.76396117	Low SDI
DALYs (Disability-Adjusted Life Years)	Both	55 to 59	57.5	34.75042979	33.89341864	35.62911088	Low SDI
DALYs (Disability-Adjusted Life Years)	Both	60 to 64	62.5	43.1034062	41.99467166	44.24141332	Low SDI
DALYs (Disability-Adjusted Life Years)	Both	15 to 19	17.5	3.57198058	3.439296691	3.709783253	Middle SDI
DALYs (Disability-Adjusted Life Years)	Both	20 to 24	22.5	3.335800075	3.21723524	3.458734384	Middle SDI
DALYs (Disability-Adjusted Life Years)	Both	25 to 29	27.5	3.476291582	3.358174289	3.598563422	Middle SDI
DALYs (Disability-Adjusted Life Years)	Both	30 to 34	32.5	4.260203735	4.124011284	4.400893841	Middle SDI
DALYs (Disability-Adjusted Life Years)	Both	35 to 39	37.5	5.463621513	5.295969817	5.636580469	Middle SDI
DALYs (Disability-Adjusted Life Years)	Both	40 to 44	42.5	6.586210525	6.384971567	6.79379205	Middle SDI
DALYs (Disability-Adjusted Life Years)	Both	45 to 49	47.5	8.120213606	7.840278458	8.410143767	Middle SDI
DALYs (Disability-Adjusted Life Years)	Both	50 to 54	52.5	11.17262751	10.78142492	11.57802484	Middle SDI
DALYs (Disability-Adjusted Life Years)	Both	55 to 59	57.5	16.69159014	16.0989383	17.30605932	Middle SDI
DALYs (Disability-Adjusted Life Years)	Both	60 to 64	62.5	21.20618441	20.42638556	22.01575291	Middle SDI
DALYs (Disability-Adjusted Life Years)	Both	15 to 19	17.5	3.663276456	3.532243093	3.799170681	Global
DALYs (Disability-Adjusted Life Years)	Both	20 to 24	22.5	3.819569468	3.692757676	3.950736063	Global
DALYs (Disability-Adjusted Life Years)	Both	25 to 29	27.5	4.107097286	3.977481056	4.240937387	Global
DALYs (Disability-Adjusted Life Years)	Both	30 to 34	32.5	5.115110317	4.964291757	5.270510848	Global
DALYs (Disability-Adjusted Life Years)	Both	35 to 39	37.5	6.760927949	6.571694756	6.955610148	Global
DALYs (Disability-Adjusted Life Years)	Both	40 to 44	42.5	8.825649251	8.583168131	9.074980649	Global
DALYs (Disability-Adjusted Life Years)	Both	45 to 49	47.5	11.55216016	11.1961979	11.91943958	Global

DALYs (Disability-Adjusted Life Years)	Both	50 to 54	52.5	16.35471716	15.84581174	16.87996663	Global
DALYs (Disability-Adjusted Life Years)	Both	55 to 59	57.5	24.91074189	24.13058559	25.71612111	Global
DALYs (Disability-Adjusted Life Years)	Both	60 to 64	62.5	34.70256396	33.5957343	35.84585873	Global
Prevalence	Male	15 to 19	17.5	0.885070244	0.791505083	0.989695901	High SDI
Prevalence	Male	20 to 24	22.5	5.981409994	5.718404794	6.256511529	High SDI
Prevalence	Male	25 to 29	27.5	16.18300071	15.72402752	16.655371	High SDI
Prevalence	Male	30 to 34	32.5	31.85444501	31.16149727	32.56280205	High SDI
Prevalence	Male	35 to 39	37.5	53.84507646	52.83249416	54.87706582	High SDI
Prevalence	Male	40 to 44	42.5	89.0111476	87.4489266	90.60127671	High SDI
Prevalence	Male	45 to 49	47.5	160.1509285	157.0602685	163.3024072	High SDI
Prevalence	Male	50 to 54	52.5	295.0801216	289.4256764	300.8450365	High SDI
Prevalence	Male	55 to 59	57.5	578.9071094	567.8241709	590.2063675	High SDI
Prevalence	Male	60 to 64	62.5	1108.620553	1087.350243	1130.306944	High SDI
Prevalence	Male	15 to 19	17.5	0.122748898	0.112089861	0.134421542	Low-middle SDI
Prevalence	Male	20 to 24	22.5	0.793098879	0.760603159	0.826982934	Low-middle SDI
Prevalence	Male	25 to 29	27.5	2.050792037	1.991516717	2.111831621	Low-middle SDI
Prevalence	Male	30 to 34	32.5	3.94457721	3.85236697	4.038994593	Low-middle SDI
Prevalence	Male	35 to 39	37.5	6.706729148	6.569296865	6.847036568	Low-middle SDI
Prevalence	Male	40 to 44	42.5	12.24390211	12.01638435	12.47572769	Low-middle SDI
Prevalence	Male	45 to 49	47.5	27.06158723	26.49109329	27.64436693	Low-middle SDI
Prevalence	Male	50 to 54	52.5	55.02744423	53.86270484	56.2173702	Low-middle SDI
Prevalence	Male	55 to 59	57.5	101.8880768	99.70415092	104.1198395	Low-middle SDI
Prevalence	Male	60 to 64	62.5	171.036238	167.3277999	174.8268652	Low-middle SDI
Prevalence	Male	15 to 19	17.5	0.333936986	0.308575947	0.36138238	High-middle SDI

Prevalence	Male	20 to 24	22.5	2.321052358	2.248729733	2.39570099	High-middle SDI
Prevalence	Male	25 to 29	27.5	6.441252212	6.311600763	6.573566931	High-middle SDI
Prevalence	Male	30 to 34	32.5	13.03199154	12.83048896	13.23665872	High-middle SDI
Prevalence	Male	35 to 39	37.5	22.77730239	22.47529426	23.0833687	High-middle SDI
Prevalence	Male	40 to 44	42.5	41.77910245	41.27730177	42.28700344	High-middle SDI
Prevalence	Male	45 to 49	47.5	92.02576864	90.79726282	93.27089641	High-middle SDI
Prevalence	Male	50 to 54	52.5	196.1521521	193.5490892	198.7902238	High-middle SDI
Prevalence	Male	55 to 59	57.5	410.957326	405.4863412	416.5021276	High-middle SDI
Prevalence	Male	60 to 64	62.5	773.9100773	763.5551155	784.4054681	High-middle SDI
Prevalence	Male	15 to 19	17.5	0.103929101	0.090078057	0.11990998	Low SDI
Prevalence	Male	20 to 24	22.5	0.63174191	0.58907004	0.677504903	Low SDI
Prevalence	Male	25 to 29	27.5	1.579120627	1.501500078	1.660753795	Low SDI
Prevalence	Male	30 to 34	32.5	2.979231467	2.858880888	3.104648455	Low SDI
Prevalence	Male	35 to 39	37.5	5.007086857	4.828385493	5.192402064	Low SDI
Prevalence	Male	40 to 44	42.5	8.822172417	8.533680063	9.120417636	Low SDI
Prevalence	Male	45 to 49	47.5	17.79616993	17.12626872	18.49227461	Low SDI
Prevalence	Male	50 to 54	52.5	31.95868874	30.73926258	33.22648952	Low SDI
Prevalence	Male	55 to 59	57.5	49.43944599	47.51463276	51.44223323	Low SDI
Prevalence	Male	60 to 64	62.5	71.97991379	69.13215637	74.94497875	Low SDI
Prevalence	Male	15 to 19	17.5	0.137632567	0.127716927	0.148318035	Middle SDI
Prevalence	Male	20 to 24	22.5	0.921674366	0.89214995	0.95217585	Middle SDI
Prevalence	Male	25 to 29	27.5	2.423259098	2.370343917	2.477355549	Middle SDI
Prevalence	Male	30 to 34	32.5	4.711238269	4.629219516	4.7947102	Middle SDI
Prevalence	Male	35 to 39	37.5	8.062641237	7.940316417	8.186850536	Middle SDI

Prevalence	Male	40 to 44	42.5	15.10209049	14.8956771	15.31136421	Middle SDI
Prevalence	Male	45 to 49	47.5	35.5605979	35.01709416	36.11253741	Middle SDI
Prevalence	Male	50 to 54	52.5	76.95016548	75.77426423	78.14431492	Middle SDI
Prevalence	Male	55 to 59	57.5	154.8432569	152.4515367	157.2724993	Middle SDI
Prevalence	Male	60 to 64	62.5	279.4335266	275.0682838	283.8680445	Middle SDI
Prevalence	Male	15 to 19	17.5	0.255269163	0.243260255	0.26787091	Global
Prevalence	Male	20 to 24	22.5	1.783097419	1.747520811	1.819398308	Global
Prevalence	Male	25 to 29	27.5	4.924414888	4.85972534	4.989965542	Global
Prevalence	Male	30 to 34	32.5	9.909110259	9.808059082	10.01120255	Global
Prevalence	Male	35 to 39	37.5	17.19000831	17.03849687	17.34286704	Global
Prevalence	Male	40 to 44	42.5	30.42761253	30.18131803	30.6759169	Global
Prevalence	Male	45 to 49	47.5	62.1642612	61.60616627	62.72741195	Global
Prevalence	Male	50 to 54	52.5	124.9491237	123.833264	126.0750385	Global
Prevalence	Male	55 to 59	57.5	252.0698369	249.8126332	254.3474357	Global
Prevalence	Male	60 to 64	62.5	474.8070467	470.5357186	479.1171481	Global
Prevalence	Female	15 to 19	17.5	0.355490019	0.293684029	0.43030312	High SDI
Prevalence	Female	20 to 24	22.5	2.47460459	2.292309205	2.671396975	High SDI
Prevalence	Female	25 to 29	27.5	6.889654148	6.562554825	7.233057179	High SDI
Prevalence	Female	30 to 34	32.5	13.90874406	13.40384217	14.43266483	High SDI
Prevalence	Female	35 to 39	37.5	23.90558472	23.15984086	24.67534143	High SDI
Prevalence	Female	40 to 44	42.5	39.9845484	38.82834982	41.17517531	High SDI
Prevalence	Female	45 to 49	47.5	73.4256816	71.08575177	75.84263491	High SDI
Prevalence	Female	50 to 54	52.5	137.8569714	133.502858	142.3530916	High SDI
Prevalence	Female	55 to 59	57.5	276.3455648	267.6383512	285.3360545	High SDI

Prevalence	Female	60 to 64	62.5	567.2073593	549.3267415	585.6699924	High SDI
Prevalence	Female	15 to 19	17.5	0.063861747	0.05617419	0.072601363	Low-middle SDI
Prevalence	Female	20 to 24	22.5	0.421528626	0.398187879	0.446237547	Low-middle SDI
Prevalence	Female	25 to 29	27.5	1.107264411	1.064522906	1.151722023	Low-middle SDI
Prevalence	Female	30 to 34	32.5	2.166171423	2.098635026	2.235881215	Low-middle SDI
Prevalence	Female	35 to 39	37.5	3.740146211	3.638092032	3.84506317	Low-middle SDI
Prevalence	Female	40 to 44	42.5	6.873719643	6.703761855	7.047986302	Low-middle SDI
Prevalence	Female	45 to 49	47.5	15.20479834	14.77690826	15.64507869	Low-middle SDI
Prevalence	Female	50 to 54	52.5	31.1156586	30.23685492	32.02000382	Low-middle SDI
Prevalence	Female	55 to 59	57.5	58.21370881	56.54882098	59.9276136	Low-middle SDI
Prevalence	Female	60 to 64	62.5	99.64422776	96.76236731	102.6119183	Low-middle SDI
Prevalence	Female	15 to 19	17.5	0.14288907	0.128036949	0.159464017	High-middle SDI
Prevalence	Female	20 to 24	22.5	0.998367949	0.955612821	1.043035987	High-middle SDI
Prevalence	Female	25 to 29	27.5	2.806745522	2.729009845	2.886695494	High-middle SDI
Prevalence	Female	30 to 34	32.5	5.709800934	5.588167112	5.834082277	High-middle SDI
Prevalence	Female	35 to 39	37.5	9.971845963	9.789859409	10.15721552	High-middle SDI
Prevalence	Female	40 to 44	42.5	18.06842879	17.76960549	18.37227725	High-middle SDI
Prevalence	Female	45 to 49	47.5	39.15184695	38.43297983	39.88416009	High-middle SDI
Prevalence	Female	50 to 54	52.5	84.41827826	82.8801743	85.9849266	High-middle SDI
Prevalence	Female	55 to 59	57.5	187.2251224	183.809904	190.7037962	High-middle SDI
Prevalence	Female	60 to 64	62.5	387.7447836	380.6494048	394.9724215	High-middle SDI
Prevalence	Female	15 to 19	17.5	0.037882145	0.029858861	0.048061341	Low SDI
Prevalence	Female	20 to 24	22.5	0.234514776	0.209598289	0.26239327	Low SDI
Prevalence	Female	25 to 29	27.5	0.594110022	0.548506806	0.643504719	Low SDI

Prevalence	Female	30 to 34	32.5	1.143675303	1.071438142	1.220782748	Low SDI
Prevalence	Female	35 to 39	37.5	1.951958433	1.842901894	2.067468559	Low SDI
Prevalence	Female	40 to 44	42.5	3.552842525	3.371954373	3.743434404	Low SDI
Prevalence	Female	45 to 49	47.5	7.619591608	7.172939814	8.094055963	Low SDI
Prevalence	Female	50 to 54	52.5	14.99832157	14.11190077	15.94042175	Low SDI
Prevalence	Female	55 to 59	57.5	27.28699058	25.6536934	29.02427512	Low SDI
Prevalence	Female	60 to 64	62.5	46.73845591	43.91280902	49.74592403	Low SDI
Prevalence	Female	15 to 19	17.5	0.08440754	0.07652619	0.093100581	Middle SDI
Prevalence	Female	20 to 24	22.5	0.574075037	0.550640788	0.598506603	Middle SDI
Prevalence	Female	25 to 29	27.5	1.530774688	1.488571096	1.574174826	Middle SDI
Prevalence	Female	30 to 34	32.5	3.020754607	2.954662895	3.088324698	Middle SDI
Prevalence	Female	35 to 39	37.5	5.215313479	5.11592979	5.316627827	Middle SDI
Prevalence	Female	40 to 44	42.5	9.605433847	9.440012443	9.773754001	Middle SDI
Prevalence	Female	45 to 49	47.5	21.91352932	21.48992215	22.34548658	Middle SDI
Prevalence	Female	50 to 54	52.5	46.43732462	45.53915359	47.35321033	Middle SDI
Prevalence	Female	55 to 59	57.5	91.66682054	89.87187969	93.49761034	Middle SDI
Prevalence	Female	60 to 64	62.5	164.9578764	161.6883268	168.2935406	Middle SDI
Prevalence	Female	15 to 19	17.5	0.116627518	0.106279901	0.127982599	Global
Prevalence	Female	20 to 24	22.5	0.813215375	0.782484972	0.845152648	Global
Prevalence	Female	25 to 29	27.5	2.267773772	2.211347294	2.325640073	Global
Prevalence	Female	30 to 34	32.5	4.628845992	4.539364161	4.720091726	Global
Prevalence	Female	35 to 39	37.5	8.126962186	7.991487992	8.264732981	Global
Prevalence	Female	40 to 44	42.5	14.51330219	14.29209463	14.73793351	Global
Prevalence	Female	45 to 49	47.5	30.00122267	29.49255986	30.51865846	Global

Prevalence	Female	50 to 54	52.5	60.97839423	59.95098185	62.02341392	Global
Prevalence	Female	55 to 59	57.5	125.6159594	123.4954901	127.7728381	Global
Prevalence	Female	60 to 64	62.5	251.241864	246.9868578	255.570174	Global
Prevalence	Both	15 to 19	17.5	0.627650371	0.554112174	0.710948084	High SDI
Prevalence	Both	20 to 24	22.5	4.278166947	4.069100867	4.497974621	High SDI
Prevalence	Both	25 to 29	27.5	11.66536149	11.29798112	12.04468809	High SDI
Prevalence	Both	30 to 34	32.5	23.15453758	22.59589512	23.72699146	High SDI
Prevalence	Both	35 to 39	37.5	39.33883306	38.51987755	40.17520005	High SDI
Prevalence	Both	40 to 44	42.5	65.19545023	63.93138019	66.48451383	High SDI
Prevalence	Both	45 to 49	47.5	117.7766606	115.2638813	120.3442191	High SDI
Prevalence	Both	50 to 54	52.5	217.6508044	213.042636	222.3586487	High SDI
Prevalence	Both	55 to 59	57.5	428.2290358	419.1751514	437.4784776	High SDI
Prevalence	Both	60 to 64	62.5	834.7556802	817.0789968	852.814781	High SDI
Prevalence	Both	15 to 19	17.5	0.093631904	0.086942433	0.100836071	Low-middle SDI
Prevalence	Both	20 to 24	22.5	0.605371762	0.585298447	0.626133509	Low-middle SDI
Prevalence	Both	25 to 29	27.5	1.570779354	1.534266607	1.608161037	Low-middle SDI
Prevalence	Both	30 to 34	32.5	3.044593845	2.987435251	3.102846055	Low-middle SDI
Prevalence	Both	35 to 39	37.5	5.214704109	5.128978394	5.301862644	Low-middle SDI
Prevalence	Both	40 to 44	42.5	9.557511744	9.41511268	9.702064527	Low-middle SDI
Prevalence	Both	45 to 49	47.5	21.12020709	20.76265704	21.48391445	Low-middle SDI
Prevalence	Both	50 to 54	52.5	42.91685749	42.18747389	43.65885149	Low-middle SDI
Prevalence	Both	55 to 59	57.5	79.35983533	77.99400405	80.74958505	Low-middle SDI
Prevalence	Both	60 to 64	62.5	133.5229385	131.1986115	135.8884435	Low-middle SDI
Prevalence	Both	15 to 19	17.5	0.239617269	0.222372767	0.258199043	High-middle SDI

Prevalence	Both	20 to 24	22.5	1.665475771	1.616413899	1.716026783	High-middle SDI
Prevalence	Both	25 to 29	27.5	4.651294482	4.562832251	4.741471781	High-middle SDI
Prevalence	Both	30 to 34	32.5	9.432172142	9.294374889	9.57201236	High-middle SDI
Prevalence	Both	35 to 39	37.5	16.46973101	16.26349802	16.67857918	High-middle SDI
Prevalence	Both	40 to 44	42.5	30.04390621	29.70312028	30.38860199	High-middle SDI
Prevalence	Both	45 to 49	47.5	65.66726866	64.83957344	66.50552963	High-middle SDI
Prevalence	Both	50 to 54	52.5	139.8325401	138.0814282	141.6058591	High-middle SDI
Prevalence	Both	55 to 59	57.5	296.4731916	292.7511737	300.242531	High-middle SDI
Prevalence	Both	60 to 64	62.5	572.7434604	565.5219548	580.0571819	High-middle SDI
Prevalence	Both	15 to 19	17.5	0.071165114	0.062954849	0.080446121	Low SDI
Prevalence	Both	20 to 24	22.5	0.428937121	0.404211196	0.45517555	Low SDI
Prevalence	Both	25 to 29	27.5	1.071676328	1.026956234	1.118343814	Low SDI
Prevalence	Both	30 to 34	32.5	2.038959856	1.969113567	2.111283657	Low SDI
Prevalence	Both	35 to 39	37.5	3.457752898	3.353151068	3.565617792	Low SDI
Prevalence	Both	40 to 44	42.5	6.180290987	6.009343348	6.356101569	Low SDI
Prevalence	Both	45 to 49	47.5	12.71833483	12.31303485	13.1369758	Low SDI
Prevalence	Both	50 to 54	52.5	23.44103909	22.68507305	24.22219724	Low SDI
Prevalence	Both	55 to 59	57.5	38.11489107	36.86403048	39.40819554	Low SDI
Prevalence	Both	60 to 64	62.5	58.73726349	56.78357641	60.75816883	Low SDI
Prevalence	Both	15 to 19	17.5	0.111723376	0.104376743	0.119587107	Middle SDI
Prevalence	Both	20 to 24	22.5	0.749445751	0.7277569	0.77178098	Middle SDI
Prevalence	Both	25 to 29	27.5	1.979228184	1.940362111	2.018872758	Middle SDI
Prevalence	Both	30 to 34	32.5	3.87119399	3.810721903	3.932625706	Middle SDI
Prevalence	Both	35 to 39	37.5	6.647363893	6.556912651	6.739062892	Middle SDI

Prevalence	Both	40 to 44	42.5	12.36767409	12.21590705	12.52132664	Middle SDI
Prevalence	Both	45 to 49	47.5	28.73643114	28.34147732	29.13688886	Middle SDI
Prevalence	Both	50 to 54	52.5	61.53767543	60.69181816	62.39532135	Middle SDI
Prevalence	Both	55 to 59	57.5	122.556315	120.8525585	124.2840907	Middle SDI
Prevalence	Both	60 to 64	62.5	219.9372303	216.8439172	223.0746699	Middle SDI
Prevalence	Both	15 to 19	17.5	0.187135166	0.176759611	0.198119752	Global
Prevalence	Both	20 to 24	22.5	1.300135396	1.269567277	1.331439521	Global
Prevalence	Both	25 to 29	27.5	3.597751504	3.542077462	3.654300626	Global
Prevalence	Both	30 to 34	32.5	7.281049073	7.193575728	7.369586088	Global
Prevalence	Both	35 to 39	37.5	12.6891747	12.55752233	12.82220731	Global
Prevalence	Both	40 to 44	42.5	22.52763311	22.31329096	22.74403424	Global
Prevalence	Both	45 to 49	47.5	46.12010549	45.63293226	46.61247975	Global
Prevalence	Both	50 to 54	52.5	92.70486064	91.73107533	93.68898332	Global
Prevalence	Both	55 to 59	57.5	187.3900923	185.4169391	189.3842432	Global
Prevalence	Both	60 to 64	62.5	358.2489749	354.4612259	362.0771996	Global
Incidence	Male	15 to 19	17.5	0.543069779	0.505889186	0.582982979	High SDI
Incidence	Male	20 to 24	22.5	1.633360218	1.564779428	1.704946752	High SDI
Incidence	Male	25 to 29	27.5	2.754150288	2.662747442	2.848690677	High SDI
Incidence	Male	30 to 34	32.5	3.904232884	3.792243106	4.019529863	High SDI
Incidence	Male	35 to 39	37.5	5.096699998	4.961195623	5.235905383	High SDI
Incidence	Male	40 to 44	42.5	9.568250269	9.336203858	9.806064071	High SDI
Incidence	Male	45 to 49	47.5	17.53109866	17.06814094	18.00661369	High SDI
Incidence	Male	50 to 54	52.5	37.47263199	36.49600618	38.47539212	High SDI
Incidence	Male	55 to 59	57.5	70.36338986	68.52563412	72.25043149	High SDI

Incidence	Male	60 to 64	62.5	141.0676078	137.3768001	144.8575739	High SDI
Incidence	Male	15 to 19	17.5	0.083142081	0.073726289	0.09376039	Low-middle SDI
Incidence	Male	20 to 24	22.5	0.257632137	0.238132346	0.278728695	Low-middle SDI
Incidence	Male	25 to 29	27.5	0.438069787	0.410412116	0.46759131	Low-middle SDI
Incidence	Male	30 to 34	32.5	0.625382627	0.58991962	0.662977491	Low-middle SDI
Incidence	Male	35 to 39	37.5	0.827835792	0.783508344	0.874671091	Low-middle SDI
Incidence	Male	40 to 44	42.5	2.03030855	1.938095687	2.126908819	Low-middle SDI
Incidence	Male	45 to 49	47.5	4.387900887	4.161043223	4.627126698	Low-middle SDI
Incidence	Male	50 to 54	52.5	7.674482861	7.271404853	8.099904816	Low-middle SDI
Incidence	Male	55 to 59	57.5	12.00022773	11.35703822	12.67984335	Low-middle SDI
Incidence	Male	60 to 64	62.5	16.98601965	16.05850077	17.96711086	Low-middle SDI
Incidence	Male	15 to 19	17.5	0.207490149	0.188575794	0.228301636	High-middle SDI
Incidence	Male	20 to 24	22.5	0.652036769	0.615934049	0.690255634	High-middle SDI
Incidence	Male	25 to 29	27.5	1.141615396	1.091833479	1.193667109	High-middle SDI
Incidence	Male	30 to 34	32.5	1.6755614	1.612554485	1.741030167	High-middle SDI
Incidence	Male	35 to 39	37.5	2.270037787	2.191540154	2.351347085	High-middle SDI
Incidence	Male	40 to 44	42.5	5.961744125	5.788950485	6.139695461	High-middle SDI
Incidence	Male	45 to 49	47.5	13.28186189	12.85283344	13.72521133	High-middle SDI
Incidence	Male	50 to 54	52.5	28.0913876	27.18662507	29.02626035	High-middle SDI
Incidence	Male	55 to 59	57.5	52.24819614	50.55393846	53.99923494	High-middle SDI
Incidence	Male	60 to 64	62.5	83.72593519	80.98945002	86.55488119	High-middle SDI
Incidence	Male	15 to 19	17.5	0.075875726	0.063091578	0.091250305	Low SDI
Incidence	Male	20 to 24	22.5	0.230510902	0.203510439	0.261093613	Low SDI
Incidence	Male	25 to 29	27.5	0.389741103	0.350941462	0.432830383	Low SDI

Incidence	Male	30 to 34	32.5	0.549921362	0.50003664	0.60478269	Low SDI
Incidence	Male	35 to 39	37.5	0.713419014	0.651511684	0.781208844	Low SDI
Incidence	Male	40 to 44	42.5	1.480563247	1.366690184	1.60392425	Low SDI
Incidence	Male	45 to 49	47.5	2.891435195	2.637267176	3.170098791	Low SDI
Incidence	Male	50 to 54	52.5	4.069130682	3.698956345	4.476350343	Low SDI
Incidence	Male	55 to 59	57.5	5.000322945	4.527972428	5.521948279	Low SDI
Incidence	Male	60 to 64	62.5	6.825366049	6.16526251	7.556145683	Low SDI
Incidence	Male	15 to 19	17.5	0.090891908	0.082302365	0.100377904	Middle SDI
Incidence	Male	20 to 24	22.5	0.284615301	0.267360098	0.302984142	Middle SDI
Incidence	Male	25 to 29	27.5	0.483660482	0.459784788	0.508775991	Middle SDI
Incidence	Male	30 to 34	32.5	0.690836529	0.660595132	0.722462346	Middle SDI
Incidence	Male	35 to 39	37.5	0.916871993	0.879312261	0.956036085	Middle SDI
Incidence	Male	40 to 44	42.5	2.555449045	2.469261832	2.644644539	Middle SDI
Incidence	Male	45 to 49	47.5	5.846884134	5.624455616	6.078108961	Middle SDI
Incidence	Male	50 to 54	52.5	11.25015062	10.81852162	11.69900042	Middle SDI
Incidence	Male	55 to 59	57.5	19.28601925	18.53385477	20.06870903	Middle SDI
Incidence	Male	60 to 64	62.5	27.83000917	26.72516649	28.98052705	Middle SDI
Incidence	Male	15 to 19	17.5	0.162156793	0.155175768	0.16945188	Global
Incidence	Male	20 to 24	22.5	0.517100245	0.503225872	0.531357146	Global
Incidence	Male	25 to 29	27.5	0.905697676	0.886263472	0.925558038	Global
Incidence	Male	30 to 34	32.5	1.324467643	1.29969709	1.349710291	Global
Incidence	Male	35 to 39	37.5	1.779480305	1.748601136	1.81090478	Global
Incidence	Male	40 to 44	42.5	4.06759653	4.006321563	4.129808672	Global
Incidence	Male	45 to 49	47.5	8.441076195	8.300031126	8.584518088	Global

Incidence	Male	50 to 54	52.5	17.43947484	17.14921489	17.7346476	Global
Incidence	Male	55 to 59	57.5	32.08783601	31.55021617	32.63461697	Global
Incidence	Male	60 to 64	62.5	57.20453311	56.24063692	58.18494932	Global
Incidence	Female	15 to 19	17.5	0.214753405	0.19209008	0.240090611	High SDI
Incidence	Female	20 to 24	22.5	0.666200678	0.622881629	0.712532401	High SDI
Incidence	Female	25 to 29	27.5	1.156279253	1.096984261	1.218779302	High SDI
Incidence	Female	30 to 34	32.5	1.682408138	1.608149782	1.760095467	High SDI
Incidence	Female	35 to 39	37.5	2.233671066	2.142861436	2.328329004	High SDI
Incidence	Female	40 to 44	42.5	4.378262407	4.217888019	4.54473462	High SDI
Incidence	Female	45 to 49	47.5	8.199725669	7.871793787	8.5413189	High SDI
Incidence	Female	50 to 54	52.5	18.01856926	17.30829967	18.75798571	High SDI
Incidence	Female	55 to 59	57.5	34.1086203	32.76309355	35.50940563	High SDI
Incidence	Female	60 to 64	62.5	85.51199097	82.1450694	89.01691427	High SDI
Incidence	Female	15 to 19	17.5	0.042379116	0.035729787	0.050265887	Low-middle SDI
Incidence	Female	20 to 24	22.5	0.130929061	0.117340565	0.146091157	Low-middle SDI
Incidence	Female	25 to 29	27.5	0.222728222	0.203511364	0.243759659	Low-middle SDI
Incidence	Female	30 to 34	32.5	0.321032549	0.296091919	0.348073996	Low-middle SDI
Incidence	Female	35 to 39	37.5	0.429752911	0.398246212	0.463752218	Low-middle SDI
Incidence	Female	40 to 44	42.5	1.082279818	1.015425455	1.153535789	Low-middle SDI
Incidence	Female	45 to 49	47.5	2.373276109	2.20577052	2.553502024	Low-middle SDI
Incidence	Female	50 to 54	52.5	4.287094171	3.98012989	4.617732823	Low-middle SDI
Incidence	Female	55 to 59	57.5	6.877942592	6.37592862	7.419483045	Low-middle SDI
Incidence	Female	60 to 64	62.5	10.78539943	9.987379717	11.64718317	Low-middle SDI
Incidence	Female	15 to 19	17.5	0.087876498	0.075774644	0.10191112	High-middle SDI

Incidence	Female	20 to 24	22.5	0.275024421	0.251773486	0.300422549	High-middle SDI
Incidence	Female	25 to 29	27.5	0.485523181	0.453048722	0.520325404	High-middle SDI
Incidence	Female	30 to 34	32.5	0.716871831	0.675428994	0.760857509	High-middle SDI
Incidence	Female	35 to 39	37.5	0.97170673	0.920091593	1.026217365	High-middle SDI
Incidence	Female	40 to 44	42.5	2.508398776	2.396326085	2.625712944	High-middle SDI
Incidence	Female	45 to 49	47.5	5.547017984	5.271571998	5.836856355	High-middle SDI
Incidence	Female	50 to 54	52.5	12.79859874	12.16745409	13.46248183	High-middle SDI
Incidence	Female	55 to 59	57.5	25.63549281	24.36536278	26.97183283	High-middle SDI
Incidence	Female	60 to 64	62.5	52.28874428	49.68979772	55.02362464	High-middle SDI
Incidence	Female	15 to 19	17.5	0.027078406	0.01987079	0.036900397	Low SDI
Incidence	Female	20 to 24	22.5	0.082427879	0.067199274	0.10110757	Low SDI
Incidence	Female	25 to 29	27.5	0.140515157	0.118528419	0.166580383	Low SDI
Incidence	Female	30 to 34	32.5	0.198216231	0.169777005	0.231419291	Low SDI
Incidence	Female	35 to 39	37.5	0.260490073	0.224732093	0.301937641	Low SDI
Incidence	Female	40 to 44	42.5	0.591958779	0.521245825	0.672264754	Low SDI
Incidence	Female	45 to 49	47.5	1.220057425	1.052941548	1.413696821	Low SDI
Incidence	Female	50 to 54	52.5	2.11044499	1.815953829	2.452693446	Low SDI
Incidence	Female	55 to 59	57.5	3.303949113	2.833673128	3.852272033	Low SDI
Incidence	Female	60 to 64	62.5	5.530340367	4.734924085	6.459378022	Low SDI
Incidence	Female	15 to 19	17.5	0.054139907	0.047463998	0.061754797	Middle SDI
Incidence	Female	20 to 24	22.5	0.168387596	0.15511904	0.182791117	Middle SDI
Incidence	Female	25 to 29	27.5	0.287170245	0.268775461	0.306823955	Middle SDI
Incidence	Female	30 to 34	32.5	0.414606762	0.391090271	0.439537313	Middle SDI
Incidence	Female	35 to 39	37.5	0.555771882	0.526278835	0.58691774	Middle SDI

Incidence	Female	40 to 44	42.5	1.514473152	1.448069495	1.58392186	Middle SDI
Incidence	Female	45 to 49	47.5	3.455653448	3.284302182	3.635944592	Middle SDI
Incidence	Female	50 to 54	52.5	6.585584083	6.255824959	6.932725579	Middle SDI
Incidence	Female	55 to 59	57.5	11.17588193	10.60613406	11.77623593	Middle SDI
Incidence	Female	60 to 64	62.5	17.48882838	16.58316283	18.44395555	Middle SDI
Incidence	Female	15 to 19	17.5	0.072823169	0.06797994	0.078011453	Global
Incidence	Female	20 to 24	22.5	0.230657548	0.221097359	0.240631117	Global
Incidence	Female	25 to 29	27.5	0.407148702	0.393653193	0.421106873	Global
Incidence	Female	30 to 34	32.5	0.60408705	0.586629679	0.622063931	Global
Incidence	Female	35 to 39	37.5	0.822462224	0.800459365	0.845069893	Global
Incidence	Female	40 to 44	42.5	1.940591118	1.895950782	1.986282514	Global
Incidence	Female	45 to 49	47.5	4.089263528	3.984563175	4.196715038	Global
Incidence	Female	50 to 54	52.5	8.728033709	8.505743027	8.956133777	Global
Incidence	Female	55 to 59	57.5	16.42457635	16.00390559	16.85630465	Global
Incidence	Female	60 to 64	62.5	35.63353568	34.71950113	36.57163333	Global
Incidence	Both	15 to 19	17.5	0.383097072	0.359334432	0.408431126	High SDI
Incidence	Both	20 to 24	22.5	1.162903057	1.118750617	1.208798009	High SDI
Incidence	Both	25 to 29	27.5	1.976800102	1.917582148	2.0378468	High SDI
Incidence	Both	30 to 34	32.5	2.826691082	2.753637308	2.901682967	High SDI
Incidence	Both	35 to 39	37.5	3.710314912	3.621624379	3.801177402	High SDI
Incidence	Both	40 to 44	42.5	7.047497349	6.894436191	7.20395657	High SDI
Incidence	Both	45 to 49	47.5	12.97369283	12.66655386	13.2882793	High SDI
Incidence	Both	50 to 54	52.5	27.89242344	27.24103945	28.55938324	High SDI
Incidence	Both	55 to 59	57.5	52.31363887	51.08986497	53.56672627	High SDI

Incidence	Both	60 to 64	62.5	113.0458295	110.4006739	115.754362	High SDI
Incidence	Both	15 to 19	17.5	0.063047751	0.057149436	0.069554822	Low-middle SDI
Incidence	Both	20 to 24	22.5	0.19326768	0.181292307	0.206034094	Low-middle SDI
Incidence	Both	25 to 29	27.5	0.328544875	0.311631665	0.346376017	Low-middle SDI
Incidence	Both	30 to 34	32.5	0.47140029	0.449607767	0.494249097	Low-middle SDI
Incidence	Both	35 to 39	37.5	0.627250994	0.599886984	0.655863221	Low-middle SDI
Incidence	Both	40 to 44	42.5	1.555234506	1.497901037	1.614762464	Low-middle SDI
Incidence	Both	45 to 49	47.5	3.375967718	3.233965292	3.524205426	Low-middle SDI
Incidence	Both	50 to 54	52.5	5.952798619	5.698527254	6.21841571	Low-middle SDI
Incidence	Both	55 to 59	57.5	9.345213834	8.937962244	9.771021538	Low-middle SDI
Incidence	Both	60 to 64	62.5	13.68548465	13.07906074	14.32002602	Low-middle SDI
Incidence	Both	15 to 19	17.5	0.14855376	0.137095486	0.160969704	High-middle SDI
Incidence	Both	20 to 24	22.5	0.465492747	0.443741037	0.488310702	High-middle SDI
Incidence	Both	25 to 29	27.5	0.818527895	0.788413997	0.849792009	High-middle SDI
Incidence	Both	30 to 34	32.5	1.20455116	1.166352778	1.24400055	High-middle SDI
Incidence	Both	35 to 39	37.5	1.631581984	1.584036397	1.680554674	High-middle SDI
Incidence	Both	40 to 44	42.5	4.253595539	4.149689203	4.360103643	High-middle SDI
Incidence	Both	45 to 49	47.5	9.429318609	9.172686487	9.693130749	High-middle SDI
Incidence	Both	50 to 54	52.5	20.39006745	19.8374588	20.95807002	High-middle SDI
Incidence	Both	55 to 59	57.5	38.64484652	37.59113482	39.72809466	High-middle SDI
Incidence	Both	60 to 64	62.5	67.41949037	65.57053328	69.32058432	High-middle SDI
Incidence	Both	15 to 19	17.5	0.051880189	0.044289664	0.060771605	Low SDI
Incidence	Both	20 to 24	22.5	0.155225915	0.139569853	0.172638175	Low SDI
Incidence	Both	25 to 29	27.5	0.26059906	0.238337333	0.284940128	Low SDI

Incidence	Both	30 to 34	32.5	0.370514289	0.341695034	0.401764219	Low SDI
Incidence	Both	35 to 39	37.5	0.483452197	0.447475013	0.522321962	Low SDI
Incidence	Both	40 to 44	42.5	1.03683079	0.968924092	1.109496704	Low SDI
Incidence	Both	45 to 49	47.5	2.062382289	1.90756395	2.229765722	Low SDI
Incidence	Both	50 to 54	52.5	3.089140899	2.850309027	3.347984868	Low SDI
Incidence	Both	55 to 59	57.5	4.123103414	3.79437785	4.480308085	Low SDI
Incidence	Both	60 to 64	62.5	6.105176894	5.610073158	6.64397484	Low SDI
Incidence	Both	15 to 19	17.5	0.073029186	0.067467488	0.079049363	Middle SDI
Incidence	Both	20 to 24	22.5	0.227173058	0.21615146	0.238756648	Middle SDI
Incidence	Both	25 to 29	27.5	0.385680123	0.370477079	0.401507046	Middle SDI
Incidence	Both	30 to 34	32.5	0.553268686	0.533958721	0.573276972	Middle SDI
Incidence	Both	35 to 39	37.5	0.737338952	0.713270911	0.762219126	Middle SDI
Incidence	Both	40 to 44	42.5	2.037157319	1.98239663	2.093430687	Middle SDI
Incidence	Both	45 to 49	47.5	4.648819243	4.507636815	4.794423606	Middle SDI
Incidence	Both	50 to 54	52.5	8.888122618	8.615755878	9.169099589	Middle SDI
Incidence	Both	55 to 59	57.5	15.12740905	14.65587575	15.6141133	Middle SDI
Incidence	Both	60 to 64	62.5	22.39891576	21.68871862	23.13236831	Middle SDI
Incidence	Both	15 to 19	17.5	0.118174555	0.113137249	0.123436142	Global
Incidence	Both	20 to 24	22.5	0.374399644	0.364469924	0.384599892	Global
Incidence	Both	25 to 29	27.5	0.656637207	0.642716508	0.670859418	Global
Incidence	Both	30 to 34	32.5	0.965769441	0.947926845	0.983947883	Global
Incidence	Both	35 to 39	37.5	1.304358281	1.282019114	1.327086708	Global
Incidence	Both	40 to 44	42.5	3.011977767	2.967339247	3.057287797	Global
Incidence	Both	45 to 49	47.5	6.270413779	6.167209072	6.375345557	Global

Incidence	Both	50 to 54	52.5	13.04467873	12.83091689	13.26200182	Global
Incidence	Both	55 to 59	57.5	24.06747962	23.67059889	24.47101479	Global
Incidence	Both	60 to 64	62.5	45.86997228	45.11056671	46.64216192	Global

Abbreviation: CI: confidence interval; SDI: Sociodemographic index.
