# Supplementary material for: Trends in the global burden of aortic valve calcification disease in the working-age population from 1992 to 2021
Source: Front Cardiovasc Med. 2025 Aug 12;12:1544273. doi: 10.3389/fcvm.2025.1544273 (PMC12379075; doi:10.3389/fcvm.2025.1544273)
Supplement: Supplementary file 3 [file Datasheet3.zip › Supplementary Table 5.PDF]

Supplementary

Table S5. Decomposition analysis of aortic valve calcification disease in the working age from 1992 to 2021

Location	Sex	Measure	Overll difference	Population size	Population age	Prevalence	Case fatality and disease severity
High SDI	Male	Deaths	-399.96	566.42(-141.62%)	713.06(-178.28%)	184.55(-46.14%)	-1864(466.04%)
High SDI	Female	Deaths	-131.95	218.47(-165.57%)	322.21(-244.2%)	59.55(-45.13%)	-732.18(554.9%)
High SDI	Both	Deaths	-531.91	765.39(-143.89%)	1022.42(-192.22%)	273.25(-51.37%)	-2592.97(487.48%)
Low-middle SDI	Male	Deaths	1072.07	770.24(71.85%)	125.47(11.7%)	412.52(38.48%)	-236.17(-22.03%)
Low-middle SDI	Female	Deaths	644.45	466.26(72.35%)	104.82(16.26%)	238.61(37.03%)	-165.23(-25.64%)
Low-middle SDI	Both	Deaths	1716.52	1242.1(72.36%)	241.92(14.09%)	633.99(36.93%)	-401.49(-23.39%)
High-middle SDI	Male	Deaths	366.29	302.08(82.47%)	384.98(105.1%)	231.22(63.12%)	-551.98(-150.69%)
High-middle SDI	Female	Deaths	164.64	119.5(72.58%)	170.27(103.42%)	99.21(60.26%)	-224.34(-136.26%)
High-middle SDI	Both	Deaths	530.93	414.47(78.06%)	554.31(104.4%)	337.27(63.52%)	-775.11(-145.99%)
Low SDI	Male	Deaths	467.29	463.96(99.29%)	-33.65(-7.2%)	78.02(16.7%)	-41.04(-8.78%)
Low SDI	Female	Deaths	281.72	305.72(108.52%)	-11.07(-3.93%)	68.85(24.44%)	-81.77(-29.02%)
Low SDI	Both	Deaths	749.01	770.4(102.86%)	-41.68(-5.57%)	137.56(18.37%)	-117.27(-15.66%)
Middle SDI	Male	Deaths	820.78	645.09(78.6%)	473.63(57.71%)	621.57(75.73%)	-919.52(-112.03%)
Middle SDI	Female	Deaths	553.23	393.81(71.18%)	308.28(55.72%)	360.87(65.23%)	-509.72(-92.14%)
Middle SDI	Both	Deaths	1374.01	1047.21(76.22%)	793.85(57.78%)	964.63(70.21%)	-1431.68(-104.2%)
Global	Male	Deaths	2331.71	3236.52(138.8%)	1515.31(64.99%)	390.59(16.75%)	-2810.71(-120.54%)
Global	Female	Deaths	1514.9	1670.73(110.29%)	819.15(54.07%)	150.62(9.94%)	-1125.59(-74.3%)
Global	Both	Deaths	3846.62	4924.92(128.03%)	2365.76(61.5%)	497.46(12.93%)	-3941.52(-102.47%)
High SDI	Male	DALYs (Disability-Adjusted Life Years)	-17202.26	21380.44(-124.29%)	22495.66(-130.77%)	6285.94(-36.54%)	-67364.3(391.6%)
High SDI	Female	DALYs (Disability-Adjusted Life Years)	-4559.07	8016.57(-175.84%)	10123.36(-222.05%)	2345.7(-51.45%)	-25044.7(549.34%)

High SDI	Both	DALYs (Disability-Adjusted Life Years)	-21761.33	28645.04(-131.63%)	32174.1(-147.85%)	9734.67(-44.73%)	-92315.14(424.22%)
Low-middle SDI	Male	DALYs (Disability-Adjusted Life Years)	44155.51	32395.95(73.37%)	4168.64(9.44%)	16065.04(36.38%)	-8474.12(-19.19%)
Low-middle SDI	Female	DALYs (Disability-Adjusted Life Years)	25757.83	19473.26(75.6%)	3219.67(12.5%)	9122.65(35.42%)	-6057.74(-23.52%)
Low-middle SDI	Both	DALYs (Disability-Adjusted Life Years)	69913.34	52112.17(74.54%)	7774.28(11.12%)	24606.52(35.2%)	-14579.62(-20.85%)
High-middle SDI	Male	DALYs (Disability-Adjusted Life Years)	12865.97	11943.58(92.83%)	12744(99.05%)	9549.87(74.23%)	-21371.48(-166.11%)
High-middle SDI	Female	DALYs (Disability-Adjusted Life Years)	6171.39	4616.02(74.8%)	5597.82(90.71%)	3979.94(64.49%)	-8022.38(-129.99%)
High-middle SDI	Both	DALYs (Disability-Adjusted Life Years)	19037.36	16273.8(85.48%)	18296.96(96.11%)	13816.07(72.57%)	-29349.47(-154.17%)
Low SDI	Male	DALYs (Disability-Adjusted Life Years)	20567.2	19751.72(96.04%)	-950.66(-4.62%)	3220.77(15.66%)	-1454.62(-7.07%)
Low SDI	Female	DALYs (Disability-Adjusted Life Years)	12366.64	13173.41(106.52%)	-314.82(-2.55%)	2803.64(22.67%)	-3295.58(-26.65%)
Low SDI	Both	DALYs (Disability-Adjusted Life Years)	32933.85	32958.42(100.07%)	-1160.8(-3.52%)	5679.54(17.25%)	-4543.32(-13.8%)
Middle SDI	Male	DALYs (Disability-Adjusted Life Years)	29642.5	26983.62(91.03%)	15275.74(51.53%)	24626.24(83.08%)	-37243.1(-125.64%)
Middle SDI	Female	DALYs (Disability-Adjusted Life Years)	19655.15	16262.48(82.74%)	9695.26(49.33%)	14130.36(71.89%)	-20432.95(-103.96%)
Middle SDI	Both	DALYs (Disability-Adjusted Life Years)	49297.65	43602.56(88.45%)	25359.95(51.44%)	38134.58(77.36%)	-57799.44(-117.25%)
Global	Male	DALYs (Disability-Adjusted Life Years)	90224.15	129576.05(143.62%)	49588.72(54.96%)	11390.29(12.62%)	-100330.91(-111.2%)
Global	Female	DALYs (Disability-Adjusted Life Years)	59501.93	66253.48(111.35%)	25914.19(43.55%)	5216.65(8.77%)	-37882.39(-63.67%)
Global	Both	DALYs (Disability-Adjusted Life Years)	149726.08	196548.66(131.27%)	76565.87(51.14%)	15007.98(10.02%)	-138396.42(-92.43%)

Abbreviation: SDI:Sociodemographic index.
