# Supplementary material for: Trends in the global burden of aortic valve calcification disease in the working-age population from 1992 to 2021
Source: Front Cardiovasc Med. 2025 Aug 12;12:1544273. doi: 10.3389/fcvm.2025.1544273 (PMC12379075; doi:10.3389/fcvm.2025.1544273)
Supplement: Supplementary file 3 [file Datasheet3.zip › Supplementary Table 4.PDF]

Supplementary

Table S4. Frontier analysis of aortic valve calcification disease in the working-age range of different countries and regions from 1992 to 2021

Location	Measure	SDI	Rate of Deaths	Frontier Deaths	Effective difference	Effective difference rank (Age-standardized Deaths rank)
	Deaths					
Afghanistan		0.337199998	0.35(0.1 to 0.8)	0.03	0.33	100 (107)
Albania		0.706849791	0.18(0.11 to 0.27)	0	0.17	42 (37)
Algeria		0.659500924	0.28(0.08 to 0.52)	0	0.27	72 (65)
American Samoa		0.723727533	0.62(0.37 to 0.97)	0	0.62	181 (181)
Andorra		0.869444113	0.24(0.13 to 0.41)	0	0.24	58 (54)
Angola		0.453721949	0.45(0.24 to 0.75)	0.02	0.42	138 (140)
Antigua and Barbuda		0.749886887	0.24(0.2 to 0.29)	0	0.23	56 (52)
Argentina		0.723122973	0.66(0.57 to 0.77)	0	0.66	185 (185)
Armenia		0.701833194	0.09(0.07 to 0.11)	0	0.09	22 (18)
Australia		0.844252814	0.31(0.27 to 0.36)	0	0.31	92 (89)
Austria		0.853837004	0.38(0.33 to 0.45)	0	0.38	121 (118)
Azerbaijan		0.694851274	0.02(0.01 to 0.03)	0	0.02	5 (2)
Bahamas		0.805020668	0.39(0.3 to 0.52)	0	0.39	128 (126)
Bahrain		0.753043204	0.32(0.21 to 0.47)	0	0.31	95 (92)
Bangladesh		0.492420885	0.25(0.13 to 0.42)	0	0.24	61 (56)
Barbados		0.746748764	0.48(0.36 to 0.65)	0	0.48	153 (153)
Belarus		0.784484711	0.08(0.06 to 0.1)	0	0.08	19 (15)
Belgium		0.853654016	0.53(0.46 to 0.62)	0	0.53	163 (162)

Belize	0.610229002	0.14(0.11 to 0.18)	0	0.14	32 (27)
Benin	0.373486574	0.22(0.1 to 0.38)	0.02	0.19	47 (49)
Bermuda	0.821365422	1.56(1.22 to 1.97)	0	1.55	204 (204)
Bhutan	0.473062378	0.24(0.13 to 0.4)	0	0.24	57 (53)
Bolivia (Plurinational State of)	0.599010799	0.43(0.25 to 0.71)	0	0.43	139 (138)
Bosnia and Herzegovina	0.723077893	0.28(0.15 to 0.45)	0	0.28	74 (68)
Botswana	0.642721629	0.21(0.11 to 0.36)	0	0.21	51 (47)
Brazil	0.653043887	0.54(0.51 to 0.57)	0	0.54	165 (165)
Brunei Darussalam	0.810234367	0.39(0.25 to 0.58)	0	0.39	125 (122)
Bulgaria	0.768150939	0.29(0.24 to 0.36)	0	0.29	82 (78)
Burkina Faso	0.285118402	0.28(0.13 to 0.5)	0.04	0.24	60 (66)
Burundi	0.289374365	0.29(0.15 to 0.51)	0.04	0.25	63 (81)
Cabo Verde	0.533534539	0.27(0.14 to 0.52)	0	0.27	71 (64)
Cambodia	0.473621491	0.03(0.01 to 0.07)	0	0.02	7 (3)
Cameroon	0.479691223	0.34(0.17 to 0.59)	0	0.34	107 (102)
Canada	0.87317068	0.32(0.27 to 0.37)	0	0.31	94 (91)
Central African Republic	0.30916769	0.47(0.23 to 0.87)	0.03	0.44	141 (149)
Chad	0.240436019	0.25(0.12 to 0.47)	0.18	0.07	18 (59)
Chile	0.771514716	0.29(0.24 to 0.35)	0	0.29	81 (76)
China	0.72162976	0.03(0.02 to 0.04)	0	0.02	8 (4)
Colombia	0.655442913	0.59(0.46 to 0.74)	0	0.58	176 (174)
Comoros	0.475978688	0.31(0.16 to 0.56)	0	0.31	93 (90)
Congo	0.583075236	0.51(0.28 to 0.84)	0	0.5	157 (157)
Cook Islands	0.779109955	0.21(0.1 to 0.41)	0	0.2	50 (46)

Costa Rica	0.700340477	0.69(0.56 to 0.83)	0	0.68	188 (188)
Coted'Ivoire	0.425941883	0.32(0.15 to 0.59)	0.02	0.3	84 (93)
Croatia	0.798341027	0.65(0.53 to 0.78)	0	0.65	183 (183)
Cuba	0.668729864	0.5(0.4 to 0.61)	0	0.5	155 (155)
Cyprus	0.835630545	0.66(0.45 to 0.97)	0	0.66	186 (186)
Czechia	0.828450433	0.59(0.49 to 0.7)	0	0.58	175 (173)
Democratic People's Republic of Korea	0.569854634	0.05(0.02 to 0.1)	0	0.05	13 (10)
Democratic Republic of the Congo	0.383179849	0.4(0.21 to 0.68)	0.02	0.38	120 (130)
Denmark	0.896424204	0.48(0.41 to 0.56)	0	0.48	152 (152)
Djibouti	0.487958371	0.32(0.16 to 0.58)	0	0.32	97 (96)
Dominica	0.746967185	0.42(0.25 to 0.68)	0	0.42	136 (136)
Dominican Republic	0.619388201	0.37(0.23 to 0.58)	0	0.37	118 (116)
Ecuador	0.661017053	0.39(0.28 to 0.51)	0	0.38	122 (119)
Egypt	0.606787094	0.43(0.27 to 0.66)	0	0.43	140 (139)
El Salvador	0.563775188	0.16(0.1 to 0.23)	0	0.15	36 (31)
Equatorial Guinea	0.657857456	0.4(0.19 to 0.75)	0	0.4	133 (132)
Eritrea	0.403863943	0.36(0.19 to 0.63)	0.02	0.34	106 (110)
Estonia	0.844917787	1.05(0.87 to 1.26)	0	1.05	199 (199)
Eswatini	0.585459713	0.37(0.18 to 0.67)	0	0.37	116 (115)
Ethiopia	0.358823295	0.2(0.11 to 0.3)	0.03	0.18	43 (42)
Fiji	0.675051631	0.16(0.09 to 0.25)	0	0.15	34 (29)
Finland	0.859831368	0.57(0.48 to 0.67)	0	0.57	170 (170)
France	0.838364875	0.4(0.34 to 0.47)	0	0.39	130 (128)
Gabon	0.634691393	0.46(0.26 to 0.78)	0	0.46	145 (144)

Gambia	0.40971416	0.35(0.17 to 0.61)	0.02	0.33	99 (104)
Georgia	0.732473604	0.37(0.29 to 0.45)	0	0.36	113 (113)
Germany	0.902957091	0.6(0.51 to 0.71)	0	0.6	179 (179)
Ghana	0.56493039	0.35(0.19 to 0.61)	0	0.35	108 (105)
Greece	0.791854408	0.47(0.4 to 0.54)	0	0.47	149 (148)
Greenland	0.826210336	0.82(0.48 to 1.25)	0	0.82	193 (193)
Grenada	0.668993028	0.68(0.53 to 0.87)	0	0.68	187 (187)
Guam	0.803982203	0.8(0.57 to 1.11)	0	0.8	192 (192)
Guatemala	0.539972424	0.21(0.17 to 0.27)	0	0.21	52 (48)
Guinea	0.336401293	0.28(0.13 to 0.52)	0.03	0.25	64 (69)
Guinea-Bissau	0.353109621	0.42(0.21 to 0.73)	0.03	0.39	126 (135)
Guyana	0.650812335	1.06(0.75 to 1.44)	0	1.06	200 (200)
Haiti	0.448278285	0.6(0.29 to 1.06)	0.02	0.58	174 (178)
Honduras	0.513037248	0.48(0.29 to 0.74)	0	0.48	151 (151)
Hungary	0.790754768	1.28(1.11 to 1.47)	0	1.28	202 (202)
Iceland	0.87636168	0.58(0.48 to 0.69)	0	0.58	172 (172)
India	0.575401649	0.27(0.2 to 0.35)	0	0.27	70 (63)
Indonesia	0.656868336	0.04(0.01 to 0.08)	0	0.03	11 (8)
Iran (Islamic Republic of)	0.697207398	0.28(0.22 to 0.41)	0	0.28	75 (70)
Iraq	0.662626231	0.45(0.21 to 0.77)	0	0.45	143 (142)
Ireland	0.87375385	0.34(0.28 to 0.41)	0	0.34	105 (101)
Israel	0.809011652	0.4(0.34 to 0.47)	0	0.4	134 (133)
Italy	0.805773534	0.38(0.35 to 0.4)	0	0.37	119 (117)
Jamaica	0.683263064	0.1(0.07 to 0.14)	0	0.1	24 (20)

Japan	0.871241813	0.2(0.19 to 0.21)	0	0.2	48 (43)
Jordan	0.725307227	0.2(0.13 to 0.29)	0	0.19	46 (41)
Kazakhstan	0.725144495	0.08(0.07 to 0.11)	0	0.08	20 (16)
Kenya	0.523768077	0.25(0.18 to 0.35)	0	0.25	62 (60)
Kiribati	0.527186583	1.35(0.74 to 2.27)	0	1.35	203 (203)
Kuwait	0.846651055	0.24(0.19 to 0.31)	0	0.24	59 (55)
Kyrgyzstan	0.603979328	0.05(0.04 to 0.07)	0	0.05	14 (11)
Lao People's Democratic Republic	0.489136091	0.04(0.01 to 0.09)	0	0.03	10 (7)
Latvia	0.830663516	0.74(0.61 to 0.89)	0	0.74	191 (191)
Lebanon	0.744746351	0.65(0.42 to 0.97)	0	0.65	184 (184)
Lesotho	0.510393066	0.36(0.18 to 0.63)	0	0.36	109 (108)
Liberia	0.352442452	0.3(0.14 to 0.56)	0.03	0.27	73 (83)
Libya	0.725771399	0.47(0.11 to 1.03)	0	0.46	146 (145)
Lithuania	0.856484049	0.56(0.46 to 0.66)	0	0.55	166 (166)
Luxembourg	0.884428955	0.58(0.49 to 0.68)	0	0.58	173 (171)
Madagascar	0.400246943	0.6(0.32 to 0.99)	0.02	0.57	171 (175)
Malawi	0.384553634	0.39(0.21 to 0.66)	0.02	0.37	115 (125)
Malaysia	0.742523828	0.16(0.1 to 0.23)	0	0.15	37 (32)
Maldives	0.650886627	0.12(0.07 to 0.19)	0	0.12	27 (23)
Mali	0.268579941	0.2(0.09 to 0.38)	0.18	0.02	6 (45)
Malta	0.801585034	0.34(0.28 to 0.4)	0	0.34	104 (100)
Marshall Islands	0.574091128	0.64(0.31 to 1.18)	0	0.64	182 (182)
Mauritania	0.4989451	0.27(0.14 to 0.48)	0	0.27	69 (62)
Mauritius	0.718260446	0.56(0.47 to 0.66)	0	0.56	168 (168)

Mexico	0.664575304	0.4(0.35 to 0.46)	0	0.4	132 (131)
Micronesia (Federated States of)	0.587534967	0.61(0.33 to 1.03)	0	0.61	180 (180)
Monaco	0.908262831	0.19(0.11 to 0.31)	0	0.18	45 (39)
Mongolia	0.617621565	0.12(0.07 to 0.19)	0	0.12	28 (24)
Montenegro	0.795800584	0.16(0.11 to 0.25)	0	0.16	39 (34)
Morocco	0.562698301	0.34(0.11 to 0.64)	0	0.33	103 (99)
Mozambique	0.326462614	0.54(0.27 to 0.94)	0.03	0.51	159 (164)
Myanmar	0.53390084	0.04(0.01 to 0.1)	0	0.04	12 (9)
Namibia	0.617564872	0.29(0.15 to 0.5)	0	0.29	80 (75)
Nauru	0.625177834	0.86(0.44 to 1.52)	0	0.86	195 (195)
Nepal	0.433174635	0.25(0.14 to 0.41)	0.02	0.23	54 (58)
Netherlands	0.888464256	0.41(0.35 to 0.48)	0	0.41	135 (134)
New Zealand	0.849442499	0.51(0.45 to 0.57)	0	0.5	158 (158)
Nicaragua	0.523958472	0.16(0.11 to 0.23)	0	0.16	38 (33)
Niger	0.168072774	0.19(0.07 to 0.35)	0.18	0.01	3 (40)
Nigeria	0.503390833	0.23(0.12 to 0.43)	0	0.23	53 (50)
Niue	0.72622205	0.57(0.33 to 0.99)	0	0.57	169 (169)
North Macedonia	0.750629703	0.18(0.12 to 0.27)	0	0.18	44 (38)
Northern Mariana Islands	0.771535213	0.91(0.58 to 1.36)	0	0.91	197 (197)
Norway	0.91613281	0.4(0.37 to 0.43)	0	0.39	131 (129)
Oman	0.773391602	0.11(0.06 to 0.18)	0	0.11	25 (21)
Pakistan	0.504028689	0.31(0.19 to 0.47)	0	0.3	88 (86)
Palau	0.754046931	0.49(0.27 to 0.83)	0	0.48	154 (154)
Palestine	0.631011665	0.16(0.1 to 0.24)	0	0.15	35 (30)

Panama	0.708864828	0.39(0.29 to 0.5)	0	0.39	124 (121)
Papua New Guinea	0.417797443	0.39(0.19 to 0.74)	0.02	0.37	117 (124)
Paraguay	0.635718099	0.86(0.56 to 1.27)	0	0.86	196 (196)
Peru	0.662054037	0.26(0.16 to 0.4)	0	0.25	65 (61)
Philippines	0.651219329	0.06(0.04 to 0.08)	0	0.05	15 (12)
Poland	0.812042809	0.74(0.67 to 0.82)	0	0.73	190 (190)
Portugal	0.744151851	0.46(0.4 to 0.53)	0	0.46	144 (143)
Puerto Rico	0.825525847	0.74(0.57 to 0.93)	0	0.73	189 (189)
Qatar	0.846860584	0.24(0.13 to 0.4)	0	0.23	55 (51)
Republic of Korea	0.886675267	0.1(0.07 to 0.14)	0	0.09	23 (19)
Republic of Moldova	0.732214875	0.09(0.07 to 0.11)	0	0.09	21 (17)
Romania	0.768453864	0.29(0.24 to 0.35)	0	0.29	83 (80)
Russian Federation	0.808536005	0.4(0.36 to 0.44)	0	0.39	129 (127)
Rwanda	0.435588706	0.31(0.16 to 0.54)	0.02	0.29	79 (88)
Saint Kitts and Nevis	0.754987055	0.32(0.24 to 0.43)	0	0.32	96 (95)
Saint Lucia	0.672509735	0.36(0.28 to 0.46)	0	0.36	111 (111)
Saint Vincent and the Grenadines	0.637195963	0.5(0.4 to 0.62)	0	0.5	156 (156)
Samoa	0.593392769	0.47(0.26 to 0.78)	0	0.46	147 (146)
San Marino	0.888005474	0.13(0.07 to 0.22)	0	0.13	31 (26)
Sao Tome and Principe	0.505413747	0.31(0.15 to 0.61)	0	0.3	89 (85)
Saudi Arabia	0.815143493	0.3(0.17 to 0.5)	0	0.3	85 (82)
Senegal	0.408054193	0.29(0.14 to 0.52)	0.02	0.27	67 (74)
Serbia	0.792416294	0.53(0.34 to 0.77)	0	0.53	162 (161)
Seychelles	0.730150775	0.15(0.06 to 0.23)	0	0.14	33 (28)

Sierra Leone	0.358665881	0.29(0.13 to 0.58)	0.03	0.27	68 (77)
Singapore	0.856097766	0.08(0.07 to 0.09)	0	0.07	17 (14)
Slovakia	0.81061053	0.37(0.25 to 0.51)	0	0.36	114 (114)
Slovenia	0.842430731	1.22(0.98 to 1.47)	0	1.22	201 (201)
Solomon Islands	0.429360316	0.32(0.16 to 0.57)	0.02	0.3	86 (94)
Somalia	0.077688109	0.25(0.09 to 0.49)	0.25	0	1.5 (57)
South Africa	0.679626598	0.33(0.25 to 0.45)	0	0.33	102 (98)
South Sudan	0.278371125	0.29(0.13 to 0.53)	0.17	0.12	29 (79)
Spain	0.769283698	0.45(0.39 to 0.51)	0	0.44	142 (141)
Sri Lanka	0.701534935	0.13(0.07 to 0.22)	0	0.13	30 (25)
Sudan	0.541949735	0.36(0.11 to 0.7)	0	0.36	110 (109)
Suriname	0.633665739	0.36(0.2 to 0.6)	0	0.36	112 (112)
Sweden	0.886880299	0.28(0.23 to 0.34)	0	0.28	76 (71)
Switzerland	0.933059111	0.29(0.24 to 0.33)	0	0.28	78 (73)
Syrian Arab Republic	0.623004075	0.84(0.45 to 1.38)	0	0.84	194 (194)
Taiwan (Province of China)	0.874747053	0.2(0.17 to 0.24)	0	0.2	49 (44)
Tajikistan	0.541511187	0(0 to 0.01)	0	0	1.5 (1)
Thailand	0.682547933	0.17(0.1 to 0.27)	0	0.16	40 (35)
Timor-Leste	0.444667619	0.03(0.01 to 0.08)	0.02	0.01	4 (6)
Togo	0.408533695	0.33(0.17 to 0.58)	0.02	0.31	91 (97)
Tokelau	0.686425621	0.6(0.34 to 1.04)	0	0.6	177 (176)
Tonga	0.626349936	0.31(0.17 to 0.55)	0	0.3	90 (87)
Trinidad and Tobago	0.768763254	0.3(0.22 to 0.41)	0	0.3	87 (84)
Tunisia	0.682432216	0.28(0.08 to 0.56)	0	0.28	77 (72)

Turkey		0.712692673	0.39(0.24 to 0.61)	0	0.39	127 (123)
Turkmenistan		0.682160776	0.07(0.05 to 0.09)	0	0.06	16 (13)
Tuvalu		0.576620529	0.56(0.32 to 0.94)	0	0.55	167 (167)
Uganda		0.423261181	0.28(0.14 to 0.48)	0.02	0.26	66 (67)
Ukraine		0.760773913	0.17(0.12 to 0.25)	0	0.17	41 (36)
United Arab Emirates		0.849317734	0.6(0.38 to 0.91)	0	0.6	178 (177)
United Kingdom		0.859000182	0.52(0.5 to 0.54)	0	0.52	160 (159)
United Republic of Tanzania		0.446568273	0.35(0.18 to 0.64)	0.02	0.32	98 (103)
United States Virgin Islands		0.821830853	0.54(0.29 to 0.93)	0	0.53	164 (163)
United States of America		0.862448354	0.47(0.45 to 0.49)	0	0.47	148 (147)
Uruguay		0.719283445	1.03(0.88 to 1.19)	0	1.02	198 (198)
Uzbekistan		0.662621694	0.11(0.09 to 0.14)	0	0.11	26 (22)
Vanuatu		0.473100706	0.48(0.25 to 0.82)	0	0.47	150 (150)
Venezuela (Bolivarian Republic of)		0.596513059	0.52(0.37 to 0.72)	0	0.52	161 (160)
Viet Nam		0.627933721	0.03(0.01 to 0.07)	0	0.03	9 (5)
Yemen		0.450376375	0.35(0.1 to 0.7)	0.02	0.33	101 (106)
Zambia		0.505948954	0.42(0.23 to 0.72)	0	0.42	137 (137)
Zimbabwe		0.473819486	0.39(0.2 to 0.68)	0	0.39	123 (120)
Location	Measure	SDI	Rate of Prevalence	Frontier Prevalence	Effective difference	Effective difference rank (Age-standardized Prevalence rank)
	Prevalence					
Afghanistan		0.337199998	16.48(11.6 to 22.79)	5.25	11.23	73 (73)
Albania		0.706849791	131.64(95.9 to 177.35)	5.25	126.39	178 (178)
Algeria		0.659500924	34.22(24.35 to 46.73)	5.21	29.02	93 (93)

American Samoa	0.723727533	20.92(14.25 to 29.95)	5.24	15.68	84 (84)
Andorra	0.869444113	111.34(80.46 to 152.64)	5.25	106.09	169 (170)
Angola	0.453721949	8.81(5.81 to 12.71)	5.26	3.55	26 (26)
Antigua and Barbuda	0.749886887	66.2(47.66 to 90.05)	5.25	60.95	125 (125)
Argentina	0.723122973	94.97(69.27 to 124.21)	5.25	89.72	151 (151)
Armenia	0.701833194	91.43(66.22 to 123.06)	5.19	86.24	147 (147)
Australia	0.844252814	88.8(63.83 to 120.42)	5.2	83.6	145 (145)
Austria	0.853837004	256.68(182.47 to 354.05)	5.2	251.48	199 (199)
Azerbaijan	0.694851274	80.86(57.51 to 109.82)	5.24	75.62	142 (142)
Bahamas	0.805020668	68.35(49.95 to 90.61)	5.24	63.11	128 (128)
Bahrain	0.753043204	40.22(28.38 to 55.46)	5.26	34.97	102 (102)
Bangladesh	0.492420885	13.4(8.95 to 19.53)	5.24	8.16	60 (60)
Barbados	0.746748764	80.44(57.83 to 110.73)	5.25	75.19	141 (141)
Belarus	0.784484711	118.1(83.71 to 160.4)	5.24	112.87	173 (173)
Belgium	0.853654016	127.4(92.2 to 173.85)	5.24	122.16	176 (176)
Belize	0.610229002	59.93(43.38 to 81.43)	5.23	54.7	117 (117)
Benin	0.373486574	9.15(6.44 to 12.5)	5.21	3.94	32 (31)
Bermuda	0.821365422	184.99(133.69 to 251.3)	5.25	179.75	194 (194)
Bhutan	0.473062378	15.86(10.55 to 22.79)	5.24	10.62	70 (70)
Bolivia (Plurinational State of)	0.599010799	64.24(45.77 to 88.33)	5.25	58.99	121 (121)
Bosnia and Herzegovina	0.723077893	158.89(115.17 to 213.29)	5.21	153.68	189 (189)

Botswana	0.642721629	13.63(9.07 to 19.33)	5.2	8.43	62 (62)
Brazil	0.653043887	71.08(50.62 to 95.81)	5.23	65.85	130 (130)
Brunei Darussalam	0.810234367	100.12(72.41 to 136.99)	5.25	94.87	154 (154)
Bulgaria	0.768150939	128.66(94.07 to 168.57)	5.2	123.46	177 (177)
Burkina Faso	0.285118402	8.61(6 to 11.93)	5.34	3.26	21 (23)
Burundi	0.289374365	6.69(4.31 to 9.8)	5.29	1.4	5 (5)
Cabo Verde	0.533534539	13.65(9.77 to 18.65)	5.2	8.45	63 (63)
Cambodia	0.473621491	7.68(4.8 to 11.27)	5.21	2.47	11 (10)
Cameroon	0.479691223	11.44(8.1 to 15.82)	5.2	6.24	48 (48)
Canada	0.87317068	121.38(87.75 to 161.99)	5.2	116.18	174 (174)
Central African Republic	0.30916769	6.34(4.11 to 9.23)	5.25	1.09	2 (2)
Chad	0.240436019	7.76(5.46 to 10.82)	5.73	2.03	8 (12)
Chile	0.771514716	104.35(75.46 to 138.88)	5.26	99.09	160 (160)
China	0.72162976	15.95(10.73 to 22.84)	5.2	10.75	71 (71)
Colombia	0.655442913	100.44(73.97 to 129.57)	5.2	95.24	155 (155)
Comoros	0.475978688	8.42(5.42 to 12.44)	5.27	3.15	17 (19)
Congo	0.583075236	10.14(6.69 to 14.82)	5.26	4.89	39 (39)
Cook Islands	0.779109955	18.78(12.49 to 27.41)	5.26	13.53	80 (80)
Costa Rica	0.700340477	117.56(86.25 to 152.26)	5.21	112.35	172 (172)

Coted'Ivoire	0.425941883	10.24(7.26 to 13.87)	5.19	5.05	40 (40)
Croatia	0.798341027	259.46(190.41 to 336.61)	5.24	254.23	200 (200)
Cuba	0.668729864	87.48(64.69 to 116.71)	5.23	82.25	144 (144)
Cyprus	0.835630545	94.69(69.81 to 126.83)	5.25	89.44	150 (150)
Czechia	0.828450433	263.97(190.76 to 357.05)	5.26	258.72	201 (201)
Democratic People's Republic of Korea	0.569854634	10.75(6.94 to 15.81)	5.24	5.51	42 (42)
Democratic Republic of the Congo	0.383179849	8.55(5.69 to 12.38)	5.26	3.29	22 (22)
Denmark	0.896424204	146.33(106.86 to 193.77)	5.25	141.08	185 (185)
Djibouti	0.487958371	9.16(6.07 to 13.2)	5.25	3.92	31 (32)
Dominica	0.746967185	72.85(52.92 to 99.08)	5.22	67.63	132 (132)
Dominican Republic	0.619388201	57.69(42.12 to 77.17)	5.23	52.46	115 (115)
Ecuador	0.661017053	80.16(65.05 to 97.35)	5.23	74.93	140 (140)
Egypt	0.606787094	36.45(25.89 to 50.82)	5.24	31.21	97 (97)
El Salvador	0.563775188	59.67(42.64 to 80.9)	5.23	54.44	116 (116)
Equatorial Guinea	0.657857456	12.47(8.27 to 17.67)	5.24	7.23	52 (52)
Eritrea	0.403863943	6.54(4.27 to 9.54)	5.2	1.34	4 (3)
Estonia	0.844917787	235.25(168.4 to 327.27)	5.25	230	198 (198)
Eswatini	0.585459713	13.33(8.86 to 18.64)	5.25	8.08	59 (59)
Ethiopia	0.358823295	6.58(4.2 to 9.74)	5.25	1.33	3 (4)
Fiji	0.675051631	12.83(8.32 to 18.81)	5.23	7.6	54 (54)
Finland	0.859831368	186.51(134.75 to	5.25	181.26	195 (195)

		253.89)			
France	0.838364875	109.96(77.68 to 152.06)	5.25	104.71	167 (167)
Gabon	0.634691393	13.27(8.86 to 18.93)	5.19	8.08	58 (58)
Gambia	0.40971416	9.78(6.86 to 13.37)	5.24	4.54	36 (36)
Georgia	0.732473604	106.92(77.69 to 144.22)	5.26	101.65	162 (162)
Germany	0.902957091	165.25(120.02 to 219.61)	5.25	160	192 (192)
Ghana	0.56493039	10.41(7.26 to 14.28)	5.24	5.17	41 (41)
Greece	0.791854408	104.22(79.72 to 134.4)	5.24	98.98	159 (159)
Greenland	0.826210336	110.09(80.38 to 146.01)	5.24	104.85	168 (168)
Grenada	0.668993028	76.56(56.34 to 108.11)	5.25	71.31	136 (136)
Guam	0.803982203	24.45(17.12 to 34.44)	5.2	19.25	86 (86)
Guatemala	0.539972424	47.36(33.62 to 65.24)	5.27	42.09	105 (105)
Guinea	0.336401293	8.85(6.28 to 12.18)	5.24	3.61	27 (27)
Guinea-Bissau	0.353109621	7.97(5.57 to 11.08)	5.26	2.71	13 (13)
Guyana	0.650812335	76.55(57.24 to 98.62)	5.25	71.3	135 (135)
Haiti	0.448278285	35.76(25.52 to 48.21)	5.19	30.56	96 (96)
Honduras	0.513037248	53.97(38.96 to 72.21)	5.19	48.78	111 (111)
Hungary	0.790754768	267.13(196.41 to 348.2)	5.26	261.87	202 (202)
Iceland	0.87636168	186.79(134.03 to 253.76)	5.2	181.59	196 (196)

India	0.575401649	12.87(8.7 to 18.56)	5.25	7.62	55 (55)
Indonesia	0.656868336	9.47(6.11 to 13.8)	5.19	4.28	34 (34)
Iran (Islamic Republic of)	0.697207398	34.28(24.62 to 46.27)	5.24	29.04	94 (94)
Iraq	0.662626231	25.19(17.23 to 35.11)	5.25	19.95	89 (89)
Ireland	0.87375385	123.56(88.81 to 165.99)	5.26	118.31	175 (175)
Israel	0.809011652	63.32(46.35 to 84.55)	5.24	58.08	120 (120)
Italy	0.805773534	135.72(104.63 to 173.15)	5.23	130.49	181 (181)
Jamaica	0.683263064	56.66(40.84 to 77)	5.27	51.39	112 (112)
Japan	0.871241813	104.43(77.21 to 138.67)	5.2	99.24	161 (161)
Jordan	0.725307227	34.91(24.43 to 48.42)	5.19	29.72	95 (95)
Kazakhstan	0.725144495	84.92(59.82 to 115.92)	5.24	79.67	143 (143)
Kenya	0.523768077	9.12(6.04 to 13.32)	5.24	3.89	29 (29)
Kiribati	0.527186583	12.35(8.2 to 17.92)	5.26	7.09	51 (51)
Kuwait	0.846651055	48.86(34.42 to 67.14)	5.25	43.61	106 (106)
Kyrgyzstan	0.603979328	65.48(46.57 to 89.06)	5.2	60.28	123 (123)
Lao People's Democratic Republic	0.489136091	7.68(4.88 to 11.66)	5.2	2.48	12 (9)
Latvia	0.830663516	191.48(136.23 to 260.29)	5.25	186.24	197 (197)
Lebanon	0.744746351	42.8(31.72 to 57.58)	5.25	37.54	103 (103)
Lesotho	0.510393066	10.8(7.3 to 15.21)	5.25	5.55	43 (44)
Liberia	0.352442452	9.85(6.92 to 13.42)	5.25	4.6	38 (38)
Libya	0.725771399	34.17(25.04 to 46.35)	5.23	28.94	92 (92)

Lithuania	0.856484049	147.58(106.55 to 200.8)	5.24	142.34	186 (186)
Luxembourg	0.884428955	170.9(121.84 to 230.35)	5.25	165.65	193 (193)
Madagascar	0.400246943	9.29(6.27 to 13.09)	5.2	4.09	33 (33)
Malawi	0.384553634	8.41(5.52 to 12.42)	5.25	3.16	19 (17)
Malaysia	0.742523828	14.71(9.55 to 21.62)	5.25	9.47	66 (66)
Maldives	0.650886627	16.64(11.13 to 24.27)	5.21	11.43	75 (75)
Mali	0.268579941	7.41(4.93 to 10.38)	5.73	1.68	7 (8)
Malta	0.801585034	92.14(67.81 to 121.08)	5.26	86.87	148 (148)
Marshall Islands	0.574091128	13.25(8.9 to 19.2)	5.25	7.99	57 (57)
Mauritania	0.4989451	11.21(7.8 to 15.33)	5.24	5.98	46 (46)
Mauritius	0.718260446	17.79(11.78 to 25.49)	5.25	12.53	77 (77)
Mexico	0.664575304	79.7(56.86 to 108.83)	5.19	74.51	137 (137)
Micronesia (Federated States of)	0.587534967	15.58(10.56 to 22.23)	5.19	10.39	69 (69)
Monaco	0.908262831	108.31(78.65 to 149.04)	5.21	103.11	164 (164)
Mongolia	0.617621565	65.62(46.23 to 88.31)	5.2	60.43	124 (124)
Montenegro	0.795800584	155.1(112.03 to 206.64)	5.26	149.83	187 (187)
Morocco	0.562698301	30.14(21.36 to 41.59)	5.26	24.89	91 (91)
Mozambique	0.326462614	8.77(5.85 to 12.77)	5.25	3.52	25 (25)
Myanmar	0.53390084	8.24(5.21 to 12.2)	5.24	3.01	15 (15)
Namibia	0.617564872	12.51(8.35 to 17.83)	5.21	7.3	53 (53)
Nauru	0.625177834	15.39(10.37 to 22.16)	5.26	10.13	68 (68)

Nepal	0.433174635	13.82(9.27 to 19.79)	5.26	8.56	64 (64)
Netherlands	0.888464256	51.42(36.82 to 68.85)	5.2	46.22	109 (109)
New Zealand	0.849442499	93.86(69.8 to 123.51)	5.23	88.62	149 (149)
Nicaragua	0.523958472	60.6(43.44 to 81.93)	5.26	55.34	118 (118)
Niger	0.168072774	7.17(4.96 to 10.05)	5.73	1.45	6 (6)
Nigeria	0.503390833	8.41(5.78 to 11.75)	5.26	3.16	18 (18)
Niue	0.72622205	18.12(12.02 to 26.42)	5.23	12.88	79 (79)
North Macedonia	0.750629703	143.15(104.84 to 190.39)	5.25	137.9	183 (183)
Northern Mariana Islands	0.771535213	29.06(20.57 to 40.26)	5.2	23.86	90 (90)
Norway	0.91613281	112.5(80.8 to 151.74)	5.24	107.26	171 (171)
Oman	0.773391602	36.96(26.48 to 49.86)	5.26	31.7	98 (98)
Pakistan	0.504028689	13.55(9.16 to 19.38)	5.21	8.33	61 (61)
Palau	0.754046931	19.39(13.04 to 28.24)	5.25	14.14	83 (83)
Palestine	0.631011665	24.78(17.26 to 33.97)	5.24	19.54	88 (88)
Panama	0.708864828	73.81(53.82 to 98.89)	5.21	68.6	133 (133)
Papua New Guinea	0.417797443	9.09(5.83 to 13.11)	5.19	3.89	30 (28)
Paraguay	0.635718099	89.56(65.96 to 121.26)	5.19	84.37	146 (146)
Peru	0.662054037	67.09(48.02 to 89.48)	5.26	61.83	127 (127)
Philippines	0.651219329	8.44(5.42 to 12.42)	5.25	3.19	20 (20)
Poland	0.812042809	57.55(42.5 to 75.04)	5.2	52.35	114 (114)
Portugal	0.744151851	62.49(45.61 to 82.67)	5.24	57.25	119 (119)
Puerto Rico	0.825525847	96.55(68.95 to 132.74)	5.2	91.34	152 (152)
Qatar	0.846860584	50.33(35.38 to 69.86)	5.19	45.14	108 (108)

Republic of Korea	0.886675267	96.79(69 to 132.55)	5.21	91.58	153 (153)
Republic of Moldova	0.732214875	111.33(79.65 to 151.95)	5.22	106.11	170 (169)
Romania	0.768453864	378.61(265.4 to 505.17)	5.22	373.39	204 (204)
Russian Federation	0.808536005	143.05(102.1 to 192.59)	5.26	137.79	182 (182)
Rwanda	0.435588706	8.23(5.34 to 12.21)	5.26	2.97	14 (14)
Saint Kitts and Nevis	0.754987055	68.46(49.63 to 91.43)	5.21	63.25	129 (129)
Saint Lucia	0.672509735	71.3(52.39 to 95.66)	5.22	66.08	131 (131)
Saint Vincent and the Grenadines	0.637195963	66.52(48.22 to 89.56)	5.21	61.32	126 (126)
Samoa	0.593392769	16.62(11.1 to 23.9)	5.25	11.37	74 (74)
San Marino	0.888005474	107.63(77.76 to 146.12)	5.21	102.42	163 (163)
Sao Tome and Principe	0.505413747	11.53(8.2 to 15.96)	5.23	6.3	49 (49)
Saudi Arabia	0.815143493	37.84(26.38 to 52.42)	5.27	32.57	101 (101)
Senegal	0.408054193	8.62(6.02 to 11.97)	5.26	3.36	24 (24)
Serbia	0.792416294	162.77(118.32 to 217.15)	5.24	157.53	191 (191)
Seychelles	0.730150775	17.93(12.06 to 25.65)	5.25	12.67	78 (78)
Sierra Leone	0.358665881	8.33(5.79 to 11.52)	5.21	3.12	16 (16)
Singapore	0.856097766	102.03(74.55 to 138.46)	5.22	96.82	156 (156)
Slovakia	0.81061053	135(101.94 to 180.46)	5.23	129.76	180 (180)
Slovenia	0.842430731	287.2(205.67 to	5.25	281.94	203 (203)

		396.55)			
Solomon Islands	0.429360316	11.2(7.31 to 16.69)	5.24	5.96	45 (45)
Somalia	0.077688109	5.74(3.69 to 8.53)	5.73	0.01	1 (1)
South Africa	0.679626598	15.99(10.94 to 22.36)	5.24	10.75	72 (72)
South Sudan	0.278371125	7.72(4.98 to 11.4)	5.63	2.09	9 (11)
Spain	0.769283698	146.29(106.21 to 192.93)	5.21	141.08	184 (184)
Sri Lanka	0.701534935	21.05(14.77 to 28.91)	5.21	15.84	85 (85)
Sudan	0.541949735	24.58(17.28 to 33.83)	5.24	19.34	87 (87)
Suriname	0.633665739	57.18(41.91 to 77.44)	5.24	51.95	113 (113)
Sweden	0.886880299	159.41(113.98 to 213.06)	5.26	154.16	190 (190)
Switzerland	0.933059111	155.87(111.58 to 212.33)	5.23	150.64	188 (188)
Syrian Arab Republic	0.623004075	45.66(33.29 to 60.97)	5.2	40.46	104 (104)
Taiwan (Province of China)	0.874747053	18.91(12.49 to 28.28)	5.21	13.7	81 (81)
Tajikistan	0.541511187	49.28(35.23 to 66.91)	5.25	44.02	107 (107)
Thailand	0.682547933	13.13(8.46 to 19.77)	5.25	7.88	56 (56)
Timor-Leste	0.444667619	7.38(4.74 to 10.96)	5.24	2.15	10 (7)
Togo	0.408533695	9.13(6.45 to 12.37)	5.25	3.88	28 (30)
Tokelau	0.686425621	15.32(10.09 to 22.55)	5.2	10.12	67 (67)
Tonga	0.626349936	17.42(11.58 to 25.46)	5.24	12.17	76 (76)
Trinidad and Tobago	0.768763254	65.29(46.44 to 89.64)	5.25	60.05	122 (122)
Tunisia	0.682432216	36.98(26.63 to 50.23)	5.24	31.74	99 (99)
Turkey	0.712692673	37.46(26.43 to 51.38)	5.24	32.23	100 (100)

Turkmenistan		0.682160776	76.08(53.63 to 103.19)	5.25	70.83	134 (134)
Tuvalu		0.576620529	14.14(9.43 to 20.06)	5.2	8.94	65 (65)
Uganda		0.423261181	8.52(5.66 to 12.31)	5.2	3.32	23 (21)
Ukraine		0.760773913	103.54(72.38 to 141.67)	5.23	98.31	157 (157)
United Arab Emirates		0.849317734	52.72(37.62 to 73.17)	5.26	47.45	110 (110)
United Kingdom		0.859000182	103.85(76.67 to 137.05)	5.24	98.61	158 (158)
United Republic of Tanzania		0.446568273	9.83(6.55 to 14.04)	5.25	4.58	37 (37)
United States Virgin Islands		0.821830853	108.45(78.06 to 143.67)	5.24	103.22	165 (165)
United States of America		0.862448354	133.86(102.05 to 170.66)	5.24	128.61	179 (179)
Uruguay		0.719283445	109.32(81.4 to 144.93)	5.26	104.07	166 (166)
Uzbekistan		0.662621694	79.79(56.62 to 108.46)	5.26	74.54	138 (138)
Vanuatu		0.473100706	11.98(7.92 to 17.44)	5.24	6.74	50 (50)
Venezuela (Bolivarian Republic of)		0.596513059	80.01(59.17 to 106.76)	5.25	74.76	139 (139)
Viet Nam		0.627933721	10.77(6.92 to 15.83)	5.21	5.56	44 (43)
Yemen		0.450376375	19.32(13.77 to 26.21)	5.24	14.08	82 (82)
Zambia		0.505948954	9.61(6.37 to 13.75)	5.21	4.4	35 (35)
Zimbabwe		0.473819486	11.36(7.79 to 16.01)	5.23	6.13	47 (47)
Location	Measure	SDI	Rate of Incidence	Frontier Incidence	Effective difference	Effective difference rank (Age-standardized Incidence rank)
	Incidence					

Afghanistan	0.337199998	2.04(1.34 to 2.93)	0.75	1.29	69 (69)
Albania	0.706849791	15.18(10.13 to 21.73)	0.75	14.43	172 (172)
Algeria	0.659500924	3.77(2.42 to 5.51)	0.75	3.03	95 (95)
American Samoa	0.723727533	2.98(1.93 to 4.42)	0.75	2.23	88 (88)
Andorra	0.869444113	14.17(9.32 to 20.37)	0.75	13.42	165 (165)
Angola	0.453721949	1.24(0.78 to 1.83)	0.75	0.5	37 (37)
Antigua and Barbuda	0.749886887	8.1(5.46 to 11.48)	0.75	7.35	123 (123)
Argentina	0.723122973	12.88(8.8 to 17.55)	0.75	12.13	155 (155)
Armenia	0.701833194	10.62(6.98 to 15.26)	0.75	9.87	145 (144)
Australia	0.844252814	12.12(8.06 to 17.46)	0.75	11.37	151 (151)
Austria	0.853837004	39.05(25.65 to 56.55)	0.75	38.3	202 (202)
Azerbaijan	0.694851274	9.09(5.81 to 13.3)	0.75	8.34	134 (134)
Bahamas	0.805020668	8.24(5.59 to 11.38)	0.75	7.49	127 (127)
Bahrain	0.753043204	4.42(2.79 to 6.58)	0.75	3.67	101 (101)
Bangladesh	0.492420885	1.68(1.05 to 2.57)	0.75	0.93	55 (55)
Barbados	0.746748764	9.9(6.64 to 14.04)	0.75	9.15	140 (140)
Belarus	0.784484711	14.79(9.7 to 21.34)	0.75	14.04	170 (170)
Belgium	0.853654016	18.05(12.18 to 25.69)	0.75	17.3	182 (182)
Belize	0.610229002	7.12(4.77 to 10.38)	0.75	6.37	115 (115)
Benin	0.373486574	1.09(0.7 to 1.54)	0.75	0.34	19 (20)
Bermuda	0.821365422	24.08(16.45 to 34.11)	0.75	23.33	192 (192)
Bhutan	0.473062378	2.04(1.25 to 3.06)	0.75	1.29	68 (68)
Bolivia (Plurinational State of)	0.599010799	8.2(5.42 to 11.62)	0.75	7.45	125 (125)
Bosnia and Herzegovina	0.723077893	19.7(13.44 to 27.67)	0.75	18.95	184 (184)

Botswana	0.642721629	1.57(0.96 to 2.33)	0.75	0.82	51 (51)
Brazil	0.653043887	8.94(5.87 to 12.93)	0.75	8.19	133 (133)
Brunei Darussalam	0.810234367	12.85(8.64 to 18.21)	0.75	12.1	154 (154)
Bulgaria	0.768150939	14.31(9.5 to 19.72)	0.75	13.56	169 (169)
Burkina Faso	0.285118402	1.08(0.7 to 1.54)	0.77	0.31	14 (16)
Burundi	0.289374365	0.91(0.54 to 1.39)	0.77	0.14	5 (5)
Cabo Verde	0.533534539	1.5(0.98 to 2.13)	0.75	0.75	50 (50)
Cambodia	0.473621491	1.07(0.63 to 1.64)	0.75	0.32	15 (14)
Cameroon	0.479691223	1.38(0.92 to 1.95)	0.75	0.63	45 (45)
Canada	0.87317068	14.29(9.68 to 20.29)	0.75	13.54	168 (168)
Central African Republic	0.30916769	0.99(0.62 to 1.42)	0.75	0.24	9 (8)
Chad	0.240436019	0.97(0.63 to 1.39)	0.8	0.17	6 (7)
Chile	0.771514716	12.89(8.76 to 18.2)	0.75	12.14	156 (156)
China	0.72162976	1.99(1.21 to 3.04)	0.75	1.24	67 (67)
Colombia	0.655442913	13.19(9.26 to 17.63)	0.75	12.44	157 (157)
Comoros	0.475978688	1.09(0.65 to 1.65)	0.75	0.34	20 (18)
Congo	0.583075236	1.42(0.88 to 2.09)	0.75	0.67	49 (49)
Cook Islands	0.779109955	2.51(1.56 to 3.86)	0.75	1.76	81 (81)
Costa Rica	0.700340477	15.68(11.08 to 20.91)	0.75	14.93	174 (174)
Coted'Ivoire	0.425941883	1.23(0.81 to 1.73)	0.75	0.49	36 (36)
Croatia	0.798341027	36.06(25.22 to 48.51)	0.75	35.31	199 (199)
Cuba	0.668729864	10.62(7.35 to 14.9)	0.75	9.87	144 (145)
Cyprus	0.835630545	14.24(9.67 to 20.2)	0.75	13.49	167 (167)
Czechia	0.828450433	35.09(24.04 to 48.62)	0.75	34.34	198 (198)

Democratic People's Republic of Korea	0.569854634	1.4(0.85 to 2.16)	0.75	0.65	46 (46)
Democratic Republic of the Congo	0.383179849	1.19(0.75 to 1.72)	0.75	0.44	31 (31)
Denmark	0.896424204	20.96(14.32 to 28.96)	0.75	20.21	188 (188)
Djibouti	0.487958371	1.17(0.72 to 1.75)	0.75	0.42	28 (28)
Dominica	0.746967185	9.18(6.29 to 13.12)	0.75	8.43	135 (135)
Dominican Republic	0.619388201	6.94(4.75 to 9.78)	0.75	6.19	112 (112)
Ecuador	0.661017053	10.48(8.05 to 13.1)	0.75	9.73	143 (143)
Egypt	0.606787094	4.16(2.67 to 6.07)	0.75	3.41	98 (98)
El Salvador	0.563775188	7.26(4.8 to 10.26)	0.75	6.51	118 (118)
Equatorial Guinea	0.657857456	1.72(1.08 to 2.52)	0.75	0.97	57 (57)
Eritrea	0.403863943	0.93(0.58 to 1.39)	0.75	0.18	7 (6)
Estonia	0.844917787	36.28(25.47 to 51.73)	0.75	35.53	201 (201)
Eswatini	0.585459713	1.59(0.98 to 2.36)	0.75	0.84	52 (52)
Ethiopia	0.358823295	0.88(0.52 to 1.34)	0.75	0.13	4 (3)
Fiji	0.675051631	1.79(1.09 to 2.71)	0.75	1.04	58 (58)
Finland	0.859831368	26.47(18.14 to 37.47)	0.75	25.72	196 (196)
France	0.838364875	15.73(10.27 to 22.33)	0.75	14.98	175 (175)
Gabon	0.634691393	1.79(1.14 to 2.62)	0.75	1.04	61 (61)
Gambia	0.40971416	1.16(0.74 to 1.68)	0.75	0.41	27 (27)
Georgia	0.732473604	13.65(9.32 to 19.18)	0.75	12.9	159 (159)
Germany	0.902957091	24.44(16.82 to 33.62)	0.75	23.69	194 (194)
Ghana	0.56493039	1.25(0.8 to 1.76)	0.75	0.5	38 (38)
Greece	0.791854408	14.04(9.98 to 18.95)	0.75	13.29	163 (163)
Greenland	0.826210336	14.23(9.5 to 20.2)	0.75	13.48	166 (166)

Grenada	0.668993028	9.66(6.57 to 14.01)	0.75	8.91	137 (137)
Guam	0.803982203	3.07(1.94 to 4.62)	0.75	2.32	89 (89)
Guatemala	0.539972424	5.94(3.99 to 8.5)	0.75	5.19	109 (109)
Guinea	0.336401293	1.09(0.71 to 1.53)	0.75	0.34	18 (19)
Guinea-Bissau	0.353109621	1.08(0.72 to 1.52)	0.75	0.33	16 (15)
Guyana	0.650812335	9.7(6.98 to 12.74)	0.75	8.95	138 (138)
Haiti	0.448278285	4.51(3 to 6.37)	0.75	3.76	103 (103)
Honduras	0.513037248	6.98(4.75 to 9.73)	0.75	6.23	114 (114)
Hungary	0.790754768	36.25(25.82 to 48.5)	0.75	35.5	200 (200)
Iceland	0.87636168	26.1(17.71 to 37.07)	0.75	25.35	195 (195)
India	0.575401649	1.63(1 to 2.48)	0.75	0.88	54 (54)
Indonesia	0.656868336	1.3(0.79 to 1.99)	0.75	0.55	41 (41)
Iran (Islamic Republic of)	0.697207398	3.63(2.3 to 5.35)	0.75	2.88	91 (91)
Iraq	0.662626231	2.72(1.72 to 4.02)	0.75	1.97	84 (84)
Ireland	0.87375385	16.44(11.1 to 23.26)	0.75	15.69	179 (179)
Israel	0.809011652	8.15(5.5 to 11.53)	0.75	7.4	124 (124)
Italy	0.805773534	20.13(14.7 to 26.48)	0.75	19.38	185 (185)
Jamaica	0.683263064	6.25(4.05 to 9.13)	0.75	5.5	110 (110)
Japan	0.871241813	12.67(8.33 to 17.94)	0.75	11.92	153 (153)
Jordan	0.725307227	3.64(2.27 to 5.43)	0.75	2.89	93 (93)
Kazakhstan	0.725144495	9.8(6.32 to 14.34)	0.75	9.05	139 (139)
Kenya	0.523768077	1.17(0.71 to 1.79)	0.75	0.42	30 (29)
Kiribati	0.527186583	1.79(1.14 to 2.64)	0.75	1.04	60 (60)
Kuwait	0.846651055	5.22(3.25 to 7.72)	0.75	4.47	105 (105)

Kyrgyzstan	0.603979328	7.24(4.63 to 10.46)	0.75	6.49	116 (116)
Lao People's Democratic Republic	0.489136091	1.08(0.64 to 1.68)	0.75	0.33	17 (17)
Latvia	0.830663516	27.01(17.99 to 37.68)	0.75	26.26	197 (197)
Lebanon	0.744746351	4.45(2.95 to 6.51)	0.75	3.7	102 (102)
Lesotho	0.510393066	1.32(0.83 to 1.95)	0.75	0.57	43 (43)
Liberia	0.352442452	1.15(0.74 to 1.62)	0.75	0.4	26 (26)
Libya	0.725771399	3.64(2.39 to 5.31)	0.75	2.89	92 (92)
Lithuania	0.856484049	20.18(14.15 to 27.94)	0.75	19.43	186 (186)
Luxembourg	0.884428955	24.43(16.19 to 34.27)	0.75	23.68	193 (193)
Madagascar	0.400246943	1.22(0.77 to 1.78)	0.75	0.47	34 (34)
Malawi	0.384553634	1.13(0.69 to 1.72)	0.75	0.38	24 (24)
Malaysia	0.742523828	2.05(1.27 to 3.11)	0.75	1.3	70 (70)
Maldives	0.650886627	2.33(1.45 to 3.52)	0.75	1.58	77 (77)
Mali	0.268579941	0.88(0.53 to 1.3)	0.8	0.08	3 (4)
Malta	0.801585034	11.59(7.81 to 16.18)	0.75	10.84	149 (149)
Marshall Islands	0.574091128	1.88(1.21 to 2.81)	0.75	1.13	65 (65)
Mauritania	0.4989451	1.2(0.75 to 1.71)	0.75	0.45	32 (32)
Mauritius	0.718260446	2.16(1.31 to 3.26)	0.75	1.41	73 (73)
Mexico	0.664575304	10.22(6.73 to 14.73)	0.75	9.47	141 (141)
Micronesia (Federated States of)	0.587534967	2.23(1.41 to 3.31)	0.75	1.48	74 (74)
Monaco	0.908262831	13.33(9 to 19.54)	0.75	12.58	158 (158)
Mongolia	0.617621565	7.61(4.88 to 10.99)	0.75	6.86	121 (121)
Montenegro	0.795800584	17.74(11.73 to 25.21)	0.75	16.99	181 (181)
Morocco	0.562698301	3.4(2.22 to 4.98)	0.75	2.65	90 (90)

Mozambique	0.326462614	1.22(0.79 to 1.8)	0.75	0.47	33 (33)
Myanmar	0.53390084	1.17(0.7 to 1.79)	0.75	0.42	29 (30)
Namibia	0.617564872	1.41(0.85 to 2.15)	0.75	0.66	47 (47)
Nauru	0.625177834	2.14(1.37 to 3.22)	0.75	1.39	72 (72)
Nepal	0.433174635	1.82(1.15 to 2.71)	0.75	1.07	63 (63)
Netherlands	0.888464256	6.94(4.61 to 9.89)	0.75	6.19	111 (111)
New Zealand	0.849442499	14.11(9.57 to 19.74)	0.75	13.36	164 (164)
Nicaragua	0.523958472	7.35(4.79 to 10.45)	0.75	6.6	119 (119)
Niger	0.168072774	0.87(0.55 to 1.27)	0.8	0.07	2 (2)
Nigeria	0.503390833	0.99(0.61 to 1.46)	0.75	0.24	10 (9)
Niue	0.72622205	2.49(1.55 to 3.79)	0.75	1.74	80 (80)
North Macedonia	0.750629703	16.43(11.1 to 23)	0.75	15.69	178 (178)
Northern Mariana Islands	0.771535213	3.74(2.41 to 5.57)	0.75	2.99	94 (94)
Norway	0.91613281	16.25(10.5 to 23.49)	0.75	15.5	177 (177)
Oman	0.773391602	4.28(2.84 to 6.15)	0.75	3.53	100 (100)
Pakistan	0.504028689	1.79(1.11 to 2.71)	0.75	1.04	59 (59)
Palau	0.754046931	2.69(1.69 to 4.03)	0.75	1.94	83 (83)
Palestine	0.631011665	2.46(1.55 to 3.64)	0.75	1.71	79 (79)
Panama	0.708864828	8.88(5.98 to 12.5)	0.75	8.13	132 (132)
Papua New Guinea	0.417797443	1.26(0.77 to 1.89)	0.75	0.51	39 (39)
Paraguay	0.635718099	11.43(7.76 to 16.24)	0.75	10.68	148 (148)
Peru	0.662054037	8.07(5.38 to 11.35)	0.75	7.32	122 (122)
Philippines	0.651219329	1.11(0.65 to 1.72)	0.75	0.36	21 (21)
Poland	0.812042809	7.24(4.94 to 10)	0.75	6.49	117 (117)

Portugal	0.744151851	8.4(5.92 to 11.72)	0.75	7.65	128 (128)
Puerto Rico	0.825525847	12.1(8.23 to 17.07)	0.75	11.35	150 (150)
Qatar	0.846860584	5.75(3.66 to 8.59)	0.75	5	108 (108)
Republic of Korea	0.886675267	11.01(7.2 to 15.83)	0.75	10.26	147 (147)
Republic of Moldova	0.732214875	13.75(9.03 to 19.71)	0.75	13	161 (161)
Romania	0.768453864	47.82(31.23 to 66.01)	0.75	47.07	204 (204)
Russian Federation	0.808536005	18.63(12.43 to 26.69)	0.75	17.88	183 (183)
Rwanda	0.435588706	1.14(0.69 to 1.71)	0.75	0.39	25 (25)
Saint Kitts and Nevis	0.754987055	8.67(5.9 to 12.31)	0.75	7.92	130 (130)
Saint Lucia	0.672509735	8.75(5.96 to 12.13)	0.75	8	131 (131)
Saint Vincent and the Grenadines	0.637195963	8.22(5.64 to 11.54)	0.75	7.47	126 (126)
Samoa	0.593392769	2.26(1.43 to 3.42)	0.75	1.51	75 (75)
San Marino	0.888005474	13.66(9.02 to 19.65)	0.75	12.91	160 (160)
Sao Tome and Principe	0.505413747	1.23(0.79 to 1.78)	0.75	0.48	35 (35)
Saudi Arabia	0.815143493	4.24(2.67 to 6.29)	0.75	3.5	99 (99)
Senegal	0.408054193	1(0.62 to 1.45)	0.75	0.25	12 (11)
Serbia	0.792416294	21.8(15.13 to 30.06)	0.75	21.05	190 (190)
Seychelles	0.730150775	2.52(1.61 to 3.71)	0.75	1.78	82 (82)
Sierra Leone	0.358665881	1.01(0.64 to 1.46)	0.75	0.26	13 (12)
Singapore	0.856097766	10.74(7.17 to 15.6)	0.75	9.99	146 (146)
Slovakia	0.81061053	16.09(11.37 to 22.39)	0.75	15.34	176 (176)
Slovenia	0.842430731	46.79(34.16 to 64.12)	0.75	46.04	203 (203)
Solomon Islands	0.429360316	1.62(1.02 to 2.46)	0.75	0.87	53 (53)
Somalia	0.077688109	0.84(0.51 to 1.27)	0.84	0	1 (1)

South Africa	0.679626598	1.84(1.12 to 2.77)	0.75	1.09	64 (64)
South Sudan	0.278371125	1.02(0.61 to 1.54)	0.79	0.23	8 (13)
Spain	0.769283698	20.22(13.89 to 27.5)	0.75	19.47	187 (187)
Sri Lanka	0.701534935	2.94(1.95 to 4.17)	0.75	2.19	87 (87)
Sudan	0.541949735	2.8(1.81 to 4.09)	0.75	2.05	86 (86)
Suriname	0.633665739	6.95(4.69 to 9.78)	0.75	6.2	113 (113)
Sweden	0.886880299	22.29(14.35 to 31.91)	0.75	21.54	191 (191)
Switzerland	0.933059111	21.37(14.1 to 30.54)	0.75	20.62	189 (189)
Syrian Arab Republic	0.623004075	4.97(3.26 to 7.17)	0.75	4.22	104 (104)
Taiwan (Province of China)	0.874747053	2.78(1.72 to 4.27)	0.75	2.03	85 (85)
Tajikistan	0.541511187	5.32(3.42 to 7.82)	0.75	4.57	106 (106)
Thailand	0.682547933	1.81(1.09 to 2.82)	0.75	1.06	62 (62)
Timor-Leste	0.444667619	0.99(0.6 to 1.5)	0.75	0.24	11 (10)
Togo	0.408533695	1.12(0.74 to 1.57)	0.75	0.37	23 (23)
Tokelau	0.686425621	2.11(1.31 to 3.22)	0.75	1.36	71 (71)
Tonga	0.626349936	2.38(1.5 to 3.64)	0.75	1.63	78 (78)
Trinidad and Tobago	0.768763254	7.6(4.91 to 11.16)	0.75	6.85	120 (120)
Tunisia	0.682432216	3.93(2.53 to 5.71)	0.75	3.18	96 (96)
Turkey	0.712692673	4.09(2.6 to 6.01)	0.75	3.35	97 (97)
Turkmenistan	0.682160776	8.61(5.52 to 12.52)	0.75	7.86	129 (129)
Tuvalu	0.576620529	1.97(1.24 to 2.91)	0.75	1.22	66 (66)
Uganda	0.423261181	1.12(0.69 to 1.67)	0.75	0.37	22 (22)
Ukraine	0.760773913	12.66(8.08 to 18.48)	0.75	11.91	152 (152)
United Arab Emirates	0.849317734	5.72(3.63 to 8.59)	0.75	4.97	107 (107)

United Kingdom		0.859000182	14.88(10.24 to 20.52)	0.75	14.13	171 (171)
United Republic of Tanzania		0.446568273	1.29(0.8 to 1.9)	0.75	0.54	40 (40)
United States Virgin Islands		0.821830853	13.96(9.36 to 19.41)	0.75	13.21	162 (162)
United States of America		0.862448354	17.38(11.97 to 23.89)	0.75	16.63	180 (180)
Uruguay		0.719283445	15.18(10.41 to 20.82)	0.75	14.43	173 (173)
Uzbekistan		0.662621694	9.54(6.28 to 13.86)	0.75	8.79	136 (136)
Vanuatu		0.473100706	1.68(1.06 to 2.52)	0.75	0.93	56 (56)
Venezuela (Bolivarian Republic of)		0.596513059	10.46(7.39 to 14.42)	0.75	9.71	142 (142)
Viet Nam		0.627933721	1.41(0.86 to 2.17)	0.75	0.66	48 (48)
Yemen		0.450376375	2.26(1.48 to 3.24)	0.75	1.51	76 (76)
Zambia		0.505948954	1.33(0.83 to 1.96)	0.75	0.58	44 (44)
Zimbabwe		0.473819486	1.32(0.83 to 1.96)	0.75	0.57	42 (42)
Location	Measure	SDI	Rate of DALYs	Frontier DALYs	Effective difference	Effective difference rank (Age-standardized DALYs rank)
	DALYs					
Afghanistan		0.337199998	15.52(4.25 to 34.93)	1.09	14.44	112 (118)
Albania		0.706849791	7.3(4.67 to 11.04)	0.39	6.91	40 (37)
Algeria		0.659500924	12.02(3.41 to 22.64)	0.39	11.63	80 (78)
American Samoa		0.723727533	28.75(16.59 to 45.17)	0.39	28.36	185 (185)
Andorra		0.869444113	9.52(5.44 to 15.82)	0.39	9.13	56 (52)
Angola		0.453721949	16.96(9.11 to 28.73)	0.93	16.03	133 (135)
Antigua and Barbuda		0.749886887	9.3(7.61 to 11.26)	0.39	8.91	53 (50)
Argentina		0.723122973	24.85(21.3 to 28.8)	0.39	24.46	176 (175)
Armenia		0.701833194	3.95(3.2 to 4.75)	0.39	3.57	21 (17)

Australia	0.844252814	12.1(10.28 to 14.13)	0.39	11.71	85 (83)
Austria	0.853837004	15.41(13.03 to 18.13)	0.39	15.03	117 (116)
Azerbaijan	0.694851274	1.35(0.86 to 2.12)	0.39	0.97	8 (6)
Bahamas	0.805020668	16.58(12.52 to 21.65)	0.39	16.19	134 (133)
Bahrain	0.753043204	13.41(9.01 to 19.75)	0.39	13.03	98 (97)
Bangladesh	0.492420885	9.6(5.22 to 16.29)	0.39	9.21	58 (53)
Barbados	0.746748764	19.72(14.56 to 26.48)	0.39	19.33	152 (151)
Belarus	0.784484711	3.6(2.76 to 4.74)	0.39	3.21	19 (15)
Belgium	0.853654016	20.98(18.02 to 24.35)	0.39	20.59	158 (157)
Belize	0.610229002	5.87(4.55 to 7.43)	0.39	5.49	31 (27)
Benin	0.373486574	8.46(4.05 to 15.01)	0.92	7.54	46 (46)
Bermuda	0.821365422	62.29(48.71 to 78.73)	0.39	61.9	203 (203)
Bhutan	0.473062378	9.39(5.13 to 15.77)	0.39	9	55 (51)
Bolivia (Plurinational State of)	0.599010799	17.17(9.94 to 28.51)	0.39	16.78	138 (138)
Bosnia and Herzegovina	0.723077893	10.94(6.13 to 17.63)	0.39	10.55	73 (67)
Botswana	0.642721629	8.9(4.53 to 15.6)	0.39	8.51	51 (48)
Brazil	0.653043887	21.37(20.12 to 22.76)	0.39	20.98	160 (159)
Brunei Darussalam	0.810234367	15.76(10.41 to 23.08)	0.39	15.37	123 (122)
Bulgaria	0.768150939	11.75(9.54 to 14.35)	0.39	11.36	77 (75)
Burkina Faso	0.285118402	10.47(4.85 to 18.95)	1.31	9.17	57 (60)
Burundi	0.289374365	11.91(5.99 to 20.92)	1.59	10.32	67 (77)
Cabo Verde	0.533534539	10.8(5.46 to 20.74)	0.39	10.41	69 (65)
Cambodia	0.473621491	1.12(0.43 to 2.79)	0.39	0.73	5 (2)
Cameroon	0.479691223	13.5(6.79 to 23.18)	0.39	13.12	100 (98)

Canada	0.87317068	12.63(10.74 to 14.82)	0.39	12.25	89 (88)
Central African Republic	0.30916769	18.01(8.54 to 33.51)	1.14	16.87	139 (143)
Chad	0.240436019	9.75(4.53 to 18.08)	6.79	2.96	18 (54)
Chile	0.771514716	11.26(9.43 to 13.33)	0.39	10.87	75 (70)
China	0.72162976	1.24(0.93 to 1.68)	0.39	0.85	6 (3)
Colombia	0.655442913	23.22(18.2 to 29.13)	0.39	22.84	169 (169)
Comoros	0.475978688	12.83(6.51 to 22.73)	0.39	12.44	90 (89)
Congo	0.583075236	19.39(10.55 to 32.69)	0.39	19	150 (149)
Cook Islands	0.779109955	10.58(5.3 to 21.38)	0.39	10.19	64 (62)
Costa Rica	0.700340477	27.21(22.18 to 32.75)	0.39	26.82	181 (180)
Coted'Ivoire	0.425941883	12.54(6.03 to 23.14)	0.92	11.62	79 (87)
Croatia	0.798341027	24.9(20.43 to 30.06)	0.39	24.51	177 (176)
Cuba	0.668729864	19.64(15.77 to 23.96)	0.39	19.25	151 (150)
Cyprus	0.835630545	24.62(16.62 to 35.92)	0.39	24.24	174 (174)
Czechia	0.828450433	22.93(18.94 to 27.25)	0.39	22.54	167 (167)
Democratic People's Republic of Korea	0.569854634	2.13(1.01 to 4.16)	0.39	1.74	13 (10)
Democratic Republic of the Congo	0.383179849	15.06(7.86 to 25.66)	0.94	14.12	110 (111)
Denmark	0.896424204	18.53(15.62 to 21.67)	0.39	18.14	146 (146)
Djibouti	0.487958371	12.89(6.34 to 23.39)	0.39	12.5	92 (91)
Dominica	0.746967185	17.02(10 to 27.48)	0.39	16.64	136 (136)
Dominican Republic	0.619388201	16.26(10 to 25.13)	0.39	15.87	131 (130)
Ecuador	0.661017053	15.92(11.72 to 21)	0.39	15.53	126 (124)
Egypt	0.606787094	20.84(12.85 to 31.79)	0.39	20.45	156 (156)
El Salvador	0.563775188	6.59(4.38 to 9.5)	0.39	6.2	35 (31)

Equatorial Guinea	0.657857456	15.53(7.29 to 29.11)	0.39	15.14	120 (119)
Eritrea	0.403863943	14.76(7.56 to 25.85)	0.94	13.82	107 (110)
Estonia	0.844917787	40.33(33.42 to 48.6)	0.39	39.94	197 (197)
Eswatini	0.585459713	16.15(7.52 to 29.23)	0.39	15.76	130 (129)
Ethiopia	0.358823295	8.14(4.66 to 12.13)	1.04	7.1	42 (41)
Fiji	0.675051631	7.07(4.06 to 11.51)	0.39	6.68	39 (35)
Finland	0.859831368	22.54(19 to 26.5)	0.39	22.15	164 (164)
France	0.838364875	16.05(13.54 to 18.91)	0.39	15.67	128 (127)
Gabon	0.634691393	17.65(9.68 to 29.94)	0.39	17.26	143 (142)
Gambia	0.40971416	13.7(6.79 to 24.14)	0.94	12.76	96 (99)
Georgia	0.732473604	14.42(11.47 to 17.83)	0.39	14.03	108 (105)
Germany	0.902957091	22.9(19.37 to 26.77)	0.39	22.51	166 (166)
Ghana	0.56493039	13.71(7.35 to 23.59)	0.39	13.32	101 (100)
Greece	0.791854408	18.36(15.87 to 21.09)	0.39	17.97	144 (144)
Greenland	0.826210336	31.5(18.48 to 47.98)	0.39	31.12	189 (189)
Grenada	0.668993028	27.25(20.95 to 34.9)	0.39	26.87	182 (182)
Guam	0.803982203	39.16(27.71 to 54.27)	0.39	38.77	196 (196)
Guatemala	0.539972424	8.78(6.97 to 10.92)	0.39	8.39	50 (47)
Guinea	0.336401293	10.96(5.17 to 20.42)	1.09	9.86	61 (68)
Guinea-Bissau	0.353109621	16.46(8.19 to 29.41)	1.05	15.4	124 (132)
Guyana	0.650812335	42.88(30.48 to 58.41)	0.39	42.49	199 (199)
Haiti	0.448278285	24.31(11.44 to 43)	0.93	23.38	172 (172)
Honduras	0.513037248	17.61(10.45 to 27.14)	0.39	17.22	142 (141)
Hungary	0.790754768	48.96(42.09 to 56.7)	0.39	48.57	202 (202)

Iceland	0.87636168	23.39(19.2 to 27.87)	0.39	23	171 (171)
India	0.575401649	10.55(7.78 to 13.56)	0.39	10.16	63 (61)
Indonesia	0.656868336	1.49(0.54 to 3.33)	0.39	1.1	9 (7)
Iran (Islamic Republic of)	0.697207398	12.07(9.33 to 17.13)	0.39	11.68	83 (81)
Iraq	0.662626231	18.83(8.93 to 32.38)	0.39	18.44	147 (147)
Ireland	0.87375385	13.27(10.99 to 15.91)	0.39	12.88	97 (96)
Israel	0.809011652	15.13(12.73 to 17.69)	0.39	14.75	113 (112)
Italy	0.805773534	15.53(14.39 to 16.76)	0.39	15.15	121 (120)
Jamaica	0.683263064	4.32(3.05 to 5.96)	0.39	3.93	24 (20)
Japan	0.871241813	8.38(7.94 to 8.88)	0.39	7.99	48 (44)
Jordan	0.725307227	8.43(5.52 to 12.56)	0.39	8.04	49 (45)
Kazakhstan	0.725144495	3.65(2.82 to 4.68)	0.39	3.26	20 (16)
Kenya	0.523768077	10.23(7.3 to 14.19)	0.39	9.84	60 (58)
Kiribati	0.527186583	66.53(35.79 to 112.47)	0.39	66.14	204 (204)
Kuwait	0.846651055	10.72(8.53 to 13.38)	0.39	10.33	68 (64)
Kyrgyzstan	0.603979328	2.39(1.8 to 3.15)	0.39	2	15 (12)
Lao People's Democratic Republic	0.489136091	1.5(0.54 to 3.77)	0.39	1.11	10 (8)
Latvia	0.830663516	29.43(24.26 to 35.3)	0.39	29.04	186 (186)
Lebanon	0.744746351	28.16(18.23 to 41.72)	0.39	27.78	184 (184)
Lesotho	0.510393066	15.26(7.54 to 27)	0.39	14.87	115 (114)
Liberia	0.352442452	11.89(5.59 to 21.92)	1.05	10.84	74 (76)
Libya	0.725771399	21.08(4.91 to 46.36)	0.39	20.69	159 (158)
Lithuania	0.856484049	21.94(18.13 to 26.03)	0.39	21.55	163 (163)
Luxembourg	0.884428955	21.54(18.18 to 25.35)	0.39	21.15	162 (161)

Madagascar	0.400246943	25.32(13.34 to 42.11)	0.95	24.36	175 (177)
Malawi	0.384553634	16(8.43 to 27.1)	0.92	15.08	118 (125)
Malaysia	0.742523828	6.19(4.01 to 9.15)	0.39	5.8	33 (29)
Maldives	0.650886627	4.92(2.98 to 7.78)	0.39	4.53	27 (23)
Mali	0.268579941	8.17(3.57 to 15.48)	6.99	1.17	11 (42)
Malta	0.801585034	13.07(10.93 to 15.51)	0.39	12.69	95 (95)
Marshall Islands	0.574091128	31.64(15.12 to 58.45)	0.39	31.25	191 (191)
Mauritania	0.4989451	10.45(5.51 to 18.39)	0.39	10.06	62 (59)
Mauritius	0.718260446	23.19(19.38 to 27.18)	0.39	22.8	168 (168)
Mexico	0.664575304	16.03(14.01 to 18.26)	0.39	15.64	127 (126)
Micronesia (Federated States of)	0.587534967	29.8(16.12 to 50.58)	0.39	29.41	187 (187)
Monaco	0.908262831	7.8(4.46 to 12.7)	0.39	7.41	45 (40)
Mongolia	0.617621565	5.19(3.25 to 7.81)	0.39	4.8	28 (24)
Montenegro	0.795800584	6.75(4.52 to 9.9)	0.39	6.37	37 (33)
Morocco	0.562698301	14.1(4.37 to 27.27)	0.39	13.71	103 (102)
Mozambique	0.326462614	21.67(10.66 to 38.1)	1.15	20.52	157 (162)
Myanmar	0.53390084	1.69(0.65 to 3.99)	0.39	1.3	12 (9)
Namibia	0.617564872	12.08(6.14 to 21.42)	0.39	11.69	84 (82)
Nauru	0.625177834	43.08(22.18 to 76.18)	0.39	42.69	200 (200)
Nepal	0.433174635	9.86(5.45 to 16.17)	0.92	8.94	54 (56)
Netherlands	0.888464256	15.9(13.53 to 18.54)	0.39	15.51	125 (123)
New Zealand	0.849442499	19.03(16.79 to 21.55)	0.39	18.64	148 (148)
Nicaragua	0.523958472	6.54(4.47 to 9.22)	0.39	6.15	34 (30)
Niger	0.168072774	7.17(2.68 to 13.59)	6.76	0.41	4 (36)

Nigeria	0.503390833	9.13(4.81 to 16.91)	0.39	8.74	52 (49)
Niue	0.72622205	29.81(17.1 to 52.28)	0.39	29.43	188 (188)
North Macedonia	0.750629703	7.32(4.92 to 10.6)	0.39	6.93	41 (38)
Northern Mariana Islands	0.771535213	42.63(27.09 to 64.11)	0.39	42.24	198 (198)
Norway	0.91613281	14.43(13.24 to 15.71)	0.39	14.04	109 (107)
Oman	0.773391602	4.73(2.66 to 7.78)	0.39	4.35	25 (21)
Pakistan	0.504028689	12.31(7.64 to 19.05)	0.39	11.92	86 (84)
Palau	0.754046931	25.64(14.25 to 43.95)	0.39	25.26	178 (178)
Palestine	0.631011665	6.6(4.2 to 10.05)	0.39	6.21	36 (32)
Panama	0.708864828	15.51(11.57 to 19.83)	0.39	15.12	119 (117)
Papua New Guinea	0.417797443	19.83(9.32 to 37.66)	0.94	18.89	149 (153)
Paraguay	0.635718099	33.83(22.03 to 49.68)	0.39	33.44	193 (193)
Peru	0.662054037	10.65(6.53 to 16.72)	0.39	10.27	65 (63)
Philippines	0.651219329	2.36(1.82 to 3.28)	0.39	1.98	14 (11)
Poland	0.812042809	27.54(24.9 to 30.4)	0.39	27.15	183 (183)
Portugal	0.744151851	17.15(14.81 to 19.74)	0.39	16.77	137 (137)
Puerto Rico	0.825525847	31.52(24.52 to 39.56)	0.39	31.13	190 (190)
Qatar	0.846860584	10.08(5.6 to 16.68)	0.39	9.69	59 (57)
Republic of Korea	0.886675267	4.2(2.98 to 5.82)	0.39	3.82	23 (19)
Republic of Moldova	0.732214875	4.02(3.14 to 5.17)	0.39	3.62	22 (18)
Romania	0.768453864	12.91(10.28 to 16.07)	0.39	12.53	93 (92)
Russian Federation	0.808536005	16.1(14.54 to 17.78)	0.39	15.72	129 (128)
Rwanda	0.435588706	12.41(6.27 to 21.7)	0.93	11.49	78 (85)
Saint Kitts and Nevis	0.754987055	12.43(9.2 to 16.57)	0.39	12.04	88 (86)

Saint Lucia	0.672509735	15.15(11.58 to 19.38)	0.39	14.76	114 (113)
Saint Vincent and the Grenadines	0.637195963	20.79(16.63 to 25.55)	0.39	20.4	155 (155)
Samoa	0.593392769	22.72(12.6 to 38.24)	0.39	22.33	165 (165)
San Marino	0.888005474	5.31(2.86 to 8.71)	0.39	4.92	29 (25)
Sao Tome and Principe	0.505413747	12.07(5.86 to 24.21)	0.39	11.68	81 (80)
Saudi Arabia	0.815143493	12.97(7.22 to 21.75)	0.39	12.58	94 (94)
Senegal	0.408054193	11.21(5.4 to 20.11)	0.93	10.28	66 (69)
Serbia	0.792416294	19.75(13.11 to 28.64)	0.39	19.36	153 (152)
Seychelles	0.730150775	5.98(2.45 to 9.44)	0.39	5.59	32 (28)
Sierra Leone	0.358665881	11.47(5.25 to 22.73)	1.04	10.44	71 (73)
Singapore	0.856097766	3.31(2.78 to 3.95)	0.39	2.92	17 (14)
Slovakia	0.81061053	14.16(9.73 to 19.84)	0.39	13.77	105 (103)
Slovenia	0.842430731	45.71(36.82 to 55.09)	0.39	45.33	201 (201)
Solomon Islands	0.429360316	14.64(7.38 to 26.57)	0.93	13.71	104 (108)
Somalia	0.077688109	9.84(3.65 to 19.88)	9.84	0	1 (55)
South Africa	0.679626598	14.2(10.69 to 19.27)	0.39	13.81	106 (104)
South Sudan	0.278371125	11.64(5.25 to 21.26)	4.36	7.28	43 (74)
Spain	0.769283698	17.37(14.93 to 20.07)	0.39	16.98	141 (140)
Sri Lanka	0.701534935	5.46(3.05 to 8.91)	0.39	5.07	30 (26)
Sudan	0.541949735	15.63(4.6 to 30.51)	0.39	15.24	122 (121)
Suriname	0.633665739	15.28(8.63 to 24.94)	0.39	14.89	116 (115)
Sweden	0.886880299	10.91(8.99 to 13.12)	0.39	10.52	72 (66)
Switzerland	0.933059111	11.36(9.55 to 13.38)	0.39	10.97	76 (72)
Syrian Arab Republic	0.623004075	35.63(19.06 to 58.48)	0.39	35.24	194 (194)

Taiwan (Province of China)	0.874747053	8.3(7 to 9.68)	0.39	7.91	47 (43)
Tajikistan	0.541511187	0.41(0.2 to 0.78)	0.39	0.03	2 (1)
Thailand	0.682547933	7.04(4.12 to 11.31)	0.39	6.65	38 (34)
Timor-Leste	0.444667619	1.31(0.43 to 3.21)	0.94	0.37	3 (5)
Togo	0.408533695	12.94(6.58 to 22.5)	0.96	11.98	87 (93)
Tokelau	0.686425621	32.13(18.41 to 55.64)	0.39	31.74	192 (192)
Tonga	0.626349936	14.75(8.05 to 26.37)	0.39	14.36	111 (109)
Trinidad and Tobago	0.768763254	12.86(9.29 to 17.42)	0.39	12.47	91 (90)
Tunisia	0.682432216	12.07(3.55 to 23.86)	0.39	11.68	82 (79)
Turkey	0.712692673	16.38(9.89 to 25.28)	0.39	15.99	132 (131)
Turkmenistan	0.682160776	3.19(2.31 to 4.28)	0.39	2.81	16 (13)
Tuvalu	0.576620529	27.21(15.54 to 46.01)	0.39	26.82	180 (181)
Uganda	0.423261181	11.35(5.68 to 19.63)	0.92	10.43	70 (71)
Ukraine	0.760773913	7.71(5.3 to 10.64)	0.39	7.32	44 (39)
United Arab Emirates	0.849317734	26.27(16.57 to 39.9)	0.39	25.88	179 (179)
United Kingdom	0.859000182	19.91(19.09 to 20.77)	0.39	19.52	154 (154)
United Republic of Tanzania	0.446568273	14(7.21 to 25.85)	0.94	13.06	99 (101)
United States Virgin Islands	0.821830853	24.6(13.07 to 43.63)	0.39	24.21	173 (173)
United States of America	0.862448354	18.36(17.34 to 19.42)	0.39	17.97	145 (145)
Uruguay	0.719283445	39.12(33.38 to 45.55)	0.39	38.73	195 (195)
Uzbekistan	0.662621694	4.87(3.82 to 6.1)	0.39	4.48	26 (22)
Vanuatu	0.473100706	23.25(12.1 to 40.16)	0.39	22.86	170 (170)
Venezuela (Bolivarian Republic of)	0.596513059	21.41(15.3 to 29.17)	0.39	21.02	161 (160)
Viet Nam	0.627933721	1.27(0.54 to 2.67)	0.39	0.88	7 (4)

Yemen	0.450376375	14.43(3.8 to 29.07)	0.92	13.5	102 (106)
Zambia	0.505948954	16.92(9.17 to 29.01)	0.39	16.53	135 (134)
Zimbabwe	0.473819486	17.26(8.7 to 31.13)	0.39	16.87	140 (139)

Abbreviation: SDI: Sociodemographic index; DALYs: Disability-Adjusted Life Years.
