# Supplementary material for: Trends in the global burden of aortic valve calcification disease in the working-age population from 1992 to 2021
Source: Front Cardiovasc Med. 2025 Aug 12;12:1544273. doi: 10.3389/fcvm.2025.1544273 (PMC12379075; doi:10.3389/fcvm.2025.1544273)
Supplement: Supplementary file 3 [file Datasheet3.zip › Supplementary Table 3.PDF]

Supplementary

Table S3. Concentration index of health inequality analysis of aortic valve calcification disease in the working-age range from 1992 to 2021

Measure	Age	Year	Concentration index	SE	Up CI	Low CI
DALYs (Disability-Adjusted Life Years)	Age-standardized	1992	0.24	0.03	0.31	0.17
DALYs (Disability-Adjusted Life Years)	Age-standardized	2021	0.00	0.03	0.05	-0.06
Deaths	Age-standardized	1992	0.25	0.03	0.32	0.18
Deaths	Age-standardized	2021	-0.01	0.03	0.05	-0.06
Incidence	Age-standardized	1992	0.52	0.03	0.58	0.46
Incidence	Age-standardized	2021	0.44	0.04	0.52	0.36
Prevalence	Age-standardized	1992	0.52	0.03	0.57	0.46
Prevalence	Age-standardized	2021	0.43	0.04	0.50	0.36
DALYs (Disability-Adjusted Life Years)	All ages	1992	0.32	0.04	0.39	0.25
DALYs (Disability-Adjusted Life Years)	All ages	2021	0.11	0.03	0.17	0.05
Deaths	All ages	1992	0.35	0.04	0.42	0.28
Deaths	All ages	2021	0.14	0.03	0.20	0.07
Incidence	All ages	1992	0.58	0.04	0.65	0.51
Incidence	All ages	2021	0.53	0.05	0.62	0.44
Prevalence	All ages	1992	0.59	0.03	0.65	0.52
Prevalence	All ages	2021	0.52	0.04	0.61	0.44

Abbreviation: CI: confidence interval； SE:Standard Error.
