# Supplementary material for: Trends in the global burden of aortic valve calcification disease in the working-age population from 1992 to 2021
Source: Front Cardiovasc Med. 2025 Aug 12;12:1544273. doi: 10.3389/fcvm.2025.1544273 (PMC12379075; doi:10.3389/fcvm.2025.1544273)
Supplement: Supplementary file 3 [file Datasheet3.zip › Supplementary Table 2.PDF]

**Supplementary**

**Table S2. Slope index of age-standardized aortic valve calcification analysis in the working-age population from 1992 to 2021**

| Measure | Location | Year | value | upper | lower | SDI | pop | pop_global | cummu | half | midpoint | weighted_order |
|---|---|---|---|---|---|---|---|---|---|---|---|---|
| DALYs | Somalia | 1992 | 12.68 | 26.06 | 4.60 | 0.05 | 3816581 | 3376967464 | 3816581 | 1908290 | 1908290.3 | 0.00 |
| DALYs | Somalia | 2021 | 9.84 | 19.88 | 3.65 | 0.08 | 10890630 | 5105255047 | 10890631 | 5445315 | 5445315.5 | 0.00 |
| DALYs | Niger | 1992 | 7.69 | 14.84 | 3.18 | 0.08 | 4059013 | 3376967464 | 7875594 | 2029507 | 5846087.2 | 0.00 |
| DALYs | Chad | 1992 | 8.36 | 15.76 | 3.70 | 0.12 | 3037794 | 3376967464 | 10913388 | 1518897 | 9394490.9 | 0.00 |
| DALYs | Mali | 1992 | 8.47 | 16.44 | 3.70 | 0.13 | 4416030 | 3376967464 | 15329418 | 2208015 | 13121403 | 0.00 |
| DALYs | Burkina Faso | 1992 | 9.47 | 17.65 | 4.23 | 0.14 | 4697926 | 3376967464 | 20027344 | 2348963 | 17678381.3 | 0.01 |
| DALYs | Ethiopia | 1992 | 11.11 | 20.03 | 5.44 | 0.15 | 27028040 | 3376967464 | 47055389 | 13514020 | 33541366.7 | 0.01 |
| DALYs | Niger | 2021 | 7.17 | 13.59 | 2.68 | 0.17 | 11701660 | 5105255047 | 22592286 | 5850828 | 16741458.7 | 0.00 |
| DALYs | Mozambique | 1992 | 13.36 | 23.59 | 6.40 | 0.18 | 6841201 | 3376967464 | 53896590 | 3420600 | 50475989.3 | 0.01 |
| DALYs | Afghanistan | 1992 | 15.61 | 40.79 | 3.42 | 0.18 | 5317936 | 3376967464 | 59214526 | 2658968 | 56555557.9 | 0.02 |
| DALYs | Guinea | 1992 | 9.52 | 17.72 | 4.27 | 0.18 | 3194335 | 3376967464 | 62408861 | 1597167 | 60811693.6 | 0.02 |
| DALYs | Uganda | 1992 | 12.68 | 24.06 | 5.55 | 0.19 | 8871780 | 3376967464 | 71280641 | 4435890 | 66844751.1 | 0.02 |
| DALYs | Malawi | 1992 | 11.91 | 21.08 | 5.88 | 0.21 | 5373904 | 3376967464 | 76654545 | 2686952 | 73967593.3 | 0.02 |
| DALYs | Burundi | 1992 | 19.85 | 38.66 | 8.83 | 0.21 | 2868701 | 3376967464 | 79523246 | 1434350 | 78088895.8 | 0.02 |
| DALYs | South Sudan | 1992 | 13.57 | 25.90 | 5.92 | 0.21 | 3170246 | 3376967464 | 82693492 | 1585123 | 81108369 | 0.02 |
| DALYs | Nepal | 1992 | 8.06 | 14.79 | 3.76 | 0.21 | 10843820 | 3376967464 | 93537312 | 5421910 | 88115402.1 | 0.03 |
| DALYs | Sierra Leone | 1992 | 10.29 | 19.22 | 4.68 | 0.21 | 2160724 | 3376967464 | 95698036 | 1080362 | 94617674.1 | 0.03 |
| DALYs | Guinea-Bissau | 1992 | 16.31 | 30.70 | 7.18 | 0.22 | 518730.4 | 3376967464 | 96216766 | 259365.2 | 95957401.1 | 0.03 |
| DALYs | Central African Republic | 1992 | 19.35 | 37.24 | 8.36 | 0.22 | 1511635 | 3376967464 | 97728401 | 755817.3 | 96972583.7 | 0.03 |
| DALYs | Benin | 1992 | 8.50 | 14.97 | 4.13 | 0.23 | 2437709 | 3376967464 | 100166110 | 1218855 | 98947255.7 | 0.03 |

| | | | | | | | | | | | | |
|---|---|---|---|---|---|---|---|---|---|---|---|---|
| DALYs | Bhutan | 1992 | 8.20 | 15.96 | 3.39 | 0.23 | 362111 | 3376967464 | 100528221 | 181055.5 | 100347165.9 | 0.03 |
| DALYs | Eritrea | 1992 | 17.19 | 32.90 | 7.29 | 0.23 | 1636011 | 3376967464 | 102164232 | 818005.3 | 101346226.7 | 0.03 |
| DALYs | Yemen | 1992 | 14.51 | 33.32 | 3.41 | 0.23 | 6649314 | 3376967464 | 108813546 | 3324657 | 105488888.8 | 0.03 |
| DALYs | Liberia | 1992 | 10.63 | 18.83 | 5.22 | 0.24 | 970267.7 | 3376967464 | 109783813 | 485133.8 | 109298679.5 | 0.03 |
| DALYs | Chad | 2021 | 9.75 | 18.08 | 4.53 | 0.24 | 8325851 | 5105255047 | 30918137 | 4162925 | 26755211.9 | 0.01 |
| DALYs | Bangladesh | 1992 | 9.34 | 17.19 | 4.62 | 0.25 | 59911290 | 3376967464 | 169695106 | 29955650 | 139739459.7 | 0.04 |
| DALYs | Senegal | 1992 | 11.86 | 21.19 | 5.88 | 0.25 | 3982520 | 3376967464 | 173677626 | 1991260 | 171686366 | 0.05 |
| DALYs | Gambia | 1992 | 11.78 | 22.08 | 5.37 | 0.25 | 540094.8 | 3376967464 | 174217721 | 270047.4 | 173947673.4 | 0.05 |
| DALYs | United Republic of Tanzania | 1992 | 14.09 | 24.29 | 7.29 | 0.26 | 13815680 | 3376967464 | 188033398 | 6907839 | 181125559.4 | 0.05 |
| DALYs | Mali | 2021 | 8.17 | 15.48 | 3.57 | 0.27 | 11895330 | 5105255047 | 42813466 | 5947664 | 36865801.5 | 0.01 |
| DALYs | Lao People's Democratic Republic | 1992 | 1.79 | 4.47 | 0.63 | 0.27 | 2290031 | 3376967464 | 190323429 | 1145016 | 189178413.7 | 0.06 |
| DALYs | Rwanda | 1992 | 25.21 | 49.02 | 10.90 | 0.28 | 3779329 | 3376967464 | 194102758 | 1889664 | 192213093.7 | 0.06 |
| DALYs | South Sudan | 2021 | 11.64 | 21.26 | 5.25 | 0.28 | 5135653 | 5105255047 | 47949119 | 2567827 | 45381292.2 | 0.01 |
| DALYs | Togo | 1992 | 12.37 | 21.60 | 6.12 | 0.28 | 1939975 | 3376967464 | 196042733 | 969987.5 | 195072745.7 | 0.06 |
| DALYs | Angola | 1992 | 16.87 | 30.43 | 8.46 | 0.28 | 5702682 | 3376967464 | 201745415 | 2851341 | 198894074.2 | 0.06 |
| DALYs | Timor-Leste | 1992 | 1.18 | 2.83 | 0.36 | 0.28 | 447494.9 | 3376967464 | 202192910 | 223747.4 | 201969162.6 | 0.06 |
| DALYs | Madagascar | 1992 | 25.99 | 45.85 | 13.68 | 0.28 | 6432226 | 3376967464 | 208625136 | 3216113 | 205409023.2 | 0.06 |
| DALYs | Equatorial Guinea | 1992 | 18.17 | 32.68 | 8.89 | 0.28 | 229117.5 | 3376967464 | 208854254 | 114558.8 | 208739695 | 0.06 |
| DALYs | Burkina Faso | 2021 | 10.47 | 18.95 | 4.85 | 0.29 | 11698660 | 5105255047 | 59647781 | 5849331 | 53798449.8 | 0.01 |
| DALYs | Cabo Verde | 1992 | 11.27 | 22.63 | 5.17 | 0.29 | 183931.3 | 3376967464 | 209038185 | 91965.67 | 208946219.4 | 0.06 |
| DALYs | Comoros | 1992 | 16.98 | 29.44 | 8.20 | 0.29 | 249116.1 | 3376967464 | 209287301 | 124558 | 209162743.1 | 0.06 |
| DALYs | Burundi | 2021 | 11.91 | 20.92 | 5.99 | 0.29 | 7040681 | 5105255047 | 66688462 | 3520341 | 63168121.4 | 0.01 |
| DALYs | Democratic Republic of the | 1992 | 14.62 | 24.90 | 7.33 | 0.29 | 20457730 | 3376967464 | 229745032 | 10228870 | 219516166.6 | 0.07 |

| | | | | | | | | | | | | | |
|---|---|---|---|---|---|---|---|---|---|---|---|---|---|
| | Congo | | | | | | | | | | | | |
| DALYs | Coted'Ivoire | 1992 | 12.83 | 23.35 | 6.20 | 0.29 | 6800901 | 3376967464 | 236545933 | 3400450 | 233145482.4 | 0.07 |
| DALYs | Cambodia | 1992 | 1.23 | 3.30 | 0.45 | 0.30 | 5566488 | 3376967464 | 242112421 | 2783244 | 239329176.7 | 0.07 |
| DALYs | Sudan | 1992 | 16.52 | 35.44 | 4.22 | 0.30 | 11202790 | 3376967464 | 253315211 | 5601395 | 247713815.6 | 0.07 |
| DALYs | Zambia | 1992 | 15.32 | 27.99 | 7.58 | 0.31 | 4197849 | 3376967464 | 257513060 | 2098925 | 255414135.1 | 0.08 |
| DALYs | Central African Republic | 2021 | 18.01 | 33.51 | 8.54 | 0.31 | 3075108 | 5105255047 | 69763570 | 1537554 | 68226015.8 | 0.01 |
| DALYs | Nigeria | 1992 | 10.79 | 16.59 | 6.11 | 0.31 | 50478270 | 3376967464 | 307991334 | 25239140 | 282752197 | 0.08 |
| DALYs | Solomon Islands | 1992 | 13.80 | 26.82 | 6.29 | 0.31 | 187733.3 | 3376967464 | 308179068 | 93866.66 | 308085200.9 | 0.09 |
| DALYs | Sao Tome and Principe | 1992 | 9.37 | 17.75 | 4.44 | 0.31 | 61668.72 | 3376967464 | 308240736 | 30834.36 | 308209902 | 0.09 |
| DALYs | Cameroon | 1992 | 12.85 | 22.57 | 6.55 | 0.32 | 5631620 | 3376967464 | 313872356 | 2815810 | 311056546.2 | 0.09 |
| DALYs | Guatemala | 1992 | 10.03 | 12.79 | 7.40 | 0.32 | 4330417 | 3376967464 | 318202773 | 2165209 | 316037564.7 | 0.09 |
| DALYs | Papua New Guinea | 1992 | 20.13 | 40.55 | 8.37 | 0.32 | 2419850 | 3376967464 | 320622623 | 1209925 | 319412698.1 | 0.09 |
| DALYs | Haiti | 1992 | 24.17 | 43.99 | 10.19 | 0.32 | 3615033 | 3376967464 | 324237656 | 1807516 | 322430139.3 | 0.10 |
| DALYs | Pakistan | 1992 | 9.09 | 14.80 | 5.00 | 0.32 | 60357000 | 3376967464 | 384594651 | 30178500 | 354416153.3 | 0.10 |
| DALYs | Myanmar | 1992 | 2.05 | 4.67 | 0.73 | 0.33 | 24926270 | 3376967464 | 409520920 | 12463130 | 397057785.4 | 0.12 |
| DALYs | Mozambique | 2021 | 21.67 | 38.10 | 10.66 | 0.33 | 16039550 | 5105255047 | 85803118 | 8019774 | 77783343.8 | 0.02 |
| DALYs | Guinea | 2021 | 10.96 | 20.42 | 5.17 | 0.34 | 6958615 | 5105255047 | 92761733 | 3479307 | 89282425.3 | 0.02 |
| DALYs | Afghanistan | 2021 | 15.52 | 34.93 | 4.25 | 0.34 | 16398400 | 5105255047 | 109160135 | 8199201 | 100960934 | 0.02 |
| DALYs | Djibouti | 1992 | 14.24 | 25.11 | 7.33 | 0.34 | 281064.1 | 3376967464 | 409801984 | 140532.1 | 409661452 | 0.12 |
| DALYs | Honduras | 1992 | 16.29 | 25.80 | 9.53 | 0.34 | 2511178 | 3376967464 | 412313162 | 1255589 | 411057573.1 | 0.12 |
| DALYs | India | 1992 | 9.41 | 14.07 | 6.00 | 0.34 | 515839400 | 3376967464 | 928152513 | 257919700 | 670232837.4 | 0.20 |
| DALYs | Kenya | 1992 | 8.67 | 12.83 | 5.75 | 0.35 | 12235470 | 3376967464 | 940387986 | 6117737 | 934270249.4 | 0.28 |
| DALYs | Mauritania | 1992 | 13.19 | 23.35 | 7.10 | 0.35 | 1105278 | 3376967464 | 941493264 | 552639.1 | 940940625.2 | 0.28 |
| DALYs | Lesotho | 1992 | 7.82 | 13.95 | 3.73 | 0.35 | 821010.8 | 3376967464 | 942314275 | 410505.4 | 941903769.7 | 0.28 |

| | | | | | | | | | | | |
|---|---|---|---|---|---|---|---|---|---|---|---|
| DALYs | Liberia | 2021 | 11.89 | 21.92 | 5.59 | 0.35 | 3135229 | 5105255047 | 112295364 | 1567614 | 110727749.6 | 0.02 |
| DALYs | Guinea-Bissau | 2021 | 16.46 | 29.41 | 8.19 | 0.35 | 1119372 | 5105255047 | 113414736 | 559686.2 | 112855050.1 | 0.02 |
| DALYs | Nicaragua | 1992 | 6.25 | 8.55 | 4.35 | 0.36 | 2103741 | 3376967464 | 944418017 | 1051871 | 943366145.8 | 0.28 |
| DALYs | Maldives | 1992 | 5.79 | 10.80 | 2.39 | 0.36 | 118284.3 | 3376967464 | 944536301 | 59142.14 | 944477158.7 | 0.28 |
| DALYs | Sierra Leone | 2021 | 11.47 | 22.73 | 5.25 | 0.36 | 5014942 | 5105255047 | 118429679 | 2507471 | 115922207.5 | 0.02 |
| DALYs | Ethiopia | 2021 | 8.14 | 12.13 | 4.66 | 0.36 | 61363610 | 5105255047 | 179793292 | 30681810 | 149111485.3 | 0.03 |
| DALYs | Vanuatu | 1992 | 24.56 | 46.94 | 11.63 | 0.36 | 84103.98 | 3376967464 | 944620405 | 42051.99 | 944578352.8 | 0.28 |
| DALYs | Morocco | 1992 | 15.93 | 30.79 | 4.31 | 0.37 | 15160090 | 3376967464 | 959780492 | 7580044 | 952200448.5 | 0.28 |
| DALYs | Benin | 2021 | 8.46 | 15.01 | 4.05 | 0.37 | 7048559 | 5105255047 | 186841851 | 3524280 | 183317571.5 | 0.04 |
| DALYs | El Salvador | 1992 | 6.28 | 8.70 | 4.42 | 0.38 | 3000660 | 3376967464 | 962781152 | 1500330 | 961280822.3 | 0.28 |
| DALYs | Democratic Republic of the Congo | 2021 | 15.06 | 25.67 | 7.86 | 0.38 | 49675100 | 5105255047 | 236516952 | 24837550 | 211679401.7 | 0.04 |
| DALYs | Malawi | 2021 | 16.00 | 27.10 | 8.43 | 0.38 | 10785330 | 5105255047 | 247302286 | 5392667 | 241909619.1 | 0.05 |
| DALYs | Ghana | 1992 | 16.45 | 27.30 | 9.03 | 0.38 | 8290370 | 3376967464 | 971071522 | 4145185 | 966926337.2 | 0.29 |
| DALYs | Madagascar | 2021 | 25.32 | 42.11 | 13.34 | 0.40 | 16136660 | 5105255047 | 263438948 | 8068331 | 255370616.9 | 0.05 |
| DALYs | Eritrea | 2021 | 14.76 | 25.85 | 7.56 | 0.40 | 3903951 | 5105255047 | 267342899 | 1951975 | 265390923.2 | 0.05 |
| DALYs | Senegal | 2021 | 11.21 | 20.11 | 5.40 | 0.41 | 8916317 | 5105255047 | 276259216 | 4458159 | 271801057.2 | 0.05 |
| DALYs | Togo | 2021 | 12.94 | 22.50 | 6.58 | 0.41 | 4808399 | 5105255047 | 281067615 | 2404199 | 278663415.3 | 0.05 |
| DALYs | Gambia | 2021 | 13.70 | 24.14 | 6.79 | 0.41 | 1328594 | 5105255047 | 282396209 | 664296.9 | 281731911.6 | 0.06 |
| DALYs | Palestine | 1992 | 8.79 | 14.60 | 4.96 | 0.41 | 1092733 | 3376967464 | 972164255 | 546366.4 | 971617888.3 | 0.29 |
| DALYs | Zimbabwe | 1992 | 8.88 | 17.25 | 4.22 | 0.41 | 5556213 | 3376967464 | 977720467 | 2778106 | 974942361.1 | 0.29 |
| DALYs | Kiribati | 1992 | 61.99 | 100.30 | 34.66 | 0.42 | 43348.47 | 3376967464 | 977763816 | 21674.24 | 977742141.7 | 0.29 |
| DALYs | Eswatini | 1992 | 12.47 | 23.01 | 5.74 | 0.42 | 429553.2 | 3376967464 | 978193369 | 214776.6 | 977978592.6 | 0.29 |
| DALYs | Papua New Guinea | 2021 | 19.83 | 37.66 | 9.32 | 0.42 | 6230283 | 5105255047 | 288626492 | 3115142 | 285511350.2 | 0.06 |

| | | | | | | | | | | | | |
|---|---|---|---|---|---|---|---|---|---|---|---|---|
| DALYs | Viet Nam | 1992 | 1.20 | 2.60 | 0.46 | 0.42 | 40268370 | 3376967464 | 1018461738 | 20134180 | 998327553.6 | 0.30 |
| DALYs | Iraq | 1992 | 24.72 | 41.45 | 12.29 | 0.42 | 10043140 | 3376967464 | 1028504876 | 5021569 | 1023483307 | 0.30 |
| DALYs | Tuvalu | 1992 | 33.27 | 60.46 | 16.60 | 0.42 | 5474.875 | 3376967464 | 1028510350 | 2737.437 | 1028507613 | 0.30 |
| DALYs | Uganda | 2021 | 11.35 | 19.63 | 5.68 | 0.42 | 22465620 | 5105255047 | 311092115 | 11232810 | 299859303.5 | 0.06 |
| DALYs | Coted'Ivoire | 2021 | 12.54 | 23.14 | 6.03 | 0.43 | 15562160 | 5105255047 | 326654276 | 7781080 | 318873195.4 | 0.06 |
| DALYs | Solomon Islands | 2021 | 14.64 | 26.57 | 7.38 | 0.43 | 400945.3 | 5105255047 | 327055221 | 200472.7 | 326854748.6 | 0.06 |
| DALYs | Congo | 1992 | 23.32 | 41.13 | 11.87 | 0.43 | 1365818 | 3376967464 | 1029876169 | 682909 | 1029193260 | 0.30 |
| DALYs | Nepal | 2021 | 9.86 | 16.17 | 5.45 | 0.43 | 19987970 | 5105255047 | 347043194 | 9993986 | 337049207.6 | 0.07 |
| DALYs | Bolivia (Plurinational State of) | 1992 | 21.25 | 33.74 | 11.85 | 0.43 | 3645533 | 3376967464 | 1033521701 | 1822766 | 1031698935 | 0.31 |
| DALYs | Rwanda | 2021 | 12.41 | 21.70 | 6.27 | 0.44 | 7848418 | 5105255047 | 354891612 | 3924209 | 350967402.9 | 0.07 |
| DALYs | Egypt | 1992 | 20.72 | 34.24 | 11.17 | 0.44 | 33112790 | 3376967464 | 1066634488 | 16556390 | 1050078095 | 0.31 |
| DALYs | Botswana | 1992 | 13.80 | 24.95 | 6.70 | 0.44 | 747651.5 | 3376967464 | 1067382140 | 373825.7 | 1067008314 | 0.32 |
| DALYs | Marshall Islands | 1992 | 30.81 | 57.06 | 13.92 | 0.44 | 23628.24 | 3376967464 | 1067405768 | 11814.12 | 1067393954 | 0.32 |
| DALYs | Belize | 1992 | 5.62 | 7.36 | 4.43 | 0.44 | 103326.3 | 3376967464 | 1067509094 | 51663.13 | 1067457431 | 0.32 |
| DALYs | Timor-Leste | 2021 | 1.31 | 3.21 | 0.43 | 0.44 | 802737.4 | 5105255047 | 355694349 | 401368.7 | 355292980.6 | 0.07 |
| DALYs | Syrian Arab Republic | 1992 | 54.06 | 89.48 | 28.78 | 0.45 | 6931728 | 3376967464 | 1074440822 | 3465864 | 1070974958 | 0.32 |
| DALYs | United Republic of Tanzania | 2021 | 14.00 | 25.85 | 7.21 | 0.45 | 32205890 | 5105255047 | 387900240 | 16102950 | 371797294.8 | 0.07 |
| DALYs | Haiti | 2021 | 24.31 | 43.00 | 11.44 | 0.45 | 8005224 | 5105255047 | 395905464 | 4002612 | 391902852.4 | 0.08 |
| DALYs | Dominican Republic | 1992 | 15.55 | 23.39 | 9.87 | 0.45 | 4367623 | 3376967464 | 1078808445 | 2183812 | 1076624634 | 0.32 |
| DALYs | Yemen | 2021 | 14.43 | 29.07 | 3.80 | 0.45 | 18839690 | 5105255047 | 414745156 | 9419846 | 405325310.2 | 0.08 |
| DALYs | Angola | 2021 | 16.96 | 28.73 | 9.11 | 0.45 | 16719890 | 5105255047 | 431465050 | 8359947 | 423105102.9 | 0.08 |
| DALYs | Grenada | 1992 | 18.95 | 25.73 | 13.37 | 0.46 | 48427.92 | 3376967464 | 1078856873 | 24213.96 | 1078832659 | 0.32 |
| DALYs | Oman | 1992 | 6.95 | 12.93 | 3.20 | 0.46 | 1153630 | 3376967464 | 1080010503 | 576815.1 | 1079433688 | 0.32 |
| DALYs | Namibia | 1992 | 11.95 | 20.13 | 6.49 | 0.46 | 804903.9 | 3376967464 | 1080815407 | 402451.9 | 1080412955 | 0.32 |

| | | | | | | | | | | | | |
|---|---|---|---|---|---|---|---|---|---|---|---|---|
| DALYs | Guyana | 1992 | 35.72 | 45.14 | 27.55 | 0.47 | 459165.3 | 3376967464 | 1081274573 | 229582.7 | 1081044990 | 0.32 |
| DALYs | Gabon | 1992 | 19.51 | 32.48 | 10.90 | 0.47 | 557425.5 | 3376967464 | 1081831998 | 278712.8 | 1081553285 | 0.32 |
| DALYs | Bhutan | 2021 | 9.39 | 15.77 | 5.13 | 0.47 | 519639.6 | 5105255047 | 431984689 | 259819.8 | 431724869.5 | 0.08 |
| DALYs | Vanuatu | 2021 | 23.25 | 40.16 | 12.10 | 0.47 | 184213.1 | 5105255047 | 432168902 | 92106.55 | 432076795.9 | 0.08 |
| DALYs | Cambodia | 2021 | 1.12 | 2.79 | 0.43 | 0.47 | 10996940 | 5105255047 | 443165844 | 5498471 | 437667373 | 0.09 |
| DALYs | Micronesia (Federated States of) | 1992 | 33.10 | 57.73 | 16.91 | 0.47 | 55679.86 | 3376967464 | 1081887678 | 27839.93 | 1081859838 | 0.32 |
| DALYs | Zimbabwe | 2021 | 17.26 | 31.13 | 8.70 | 0.47 | 8807361 | 5105255047 | 451973205 | 4403681 | 447569524.4 | 0.09 |
| DALYs | Tajikistan | 1992 | 0.41 | 0.73 | 0.21 | 0.48 | 2943035 | 3376967464 | 1084830713 | 1471517 | 1083359195 | 0.32 |
| DALYs | Indonesia | 1992 | 1.48 | 3.69 | 0.57 | 0.48 | 115716600 | 3376967464 | 1200547263 | 57858280 | 1142688988 | 0.34 |
| DALYs | China | 1992 | 1.86 | 2.78 | 1.17 | 0.48 | 815239200 | 3376967464 | 2015786424 | 407619600 | 1608166844 | 0.48 |
| DALYs | Algeria | 1992 | 14.84 | 28.16 | 3.87 | 0.48 | 14528470 | 3376967464 | 2030314898 | 7264237 | 2023050661 | 0.60 |
| DALYs | Comoros | 2021 | 12.83 | 22.73 | 6.51 | 0.48 | 467233.1 | 5105255047 | 452440438 | 233616.6 | 452206821.7 | 0.09 |
| DALYs | Turkey | 1992 | 25.95 | 44.24 | 12.59 | 0.48 | 36334360 | 3376967464 | 2066649254 | 18167180 | 2048482076 | 0.61 |
| DALYs | Mongolia | 1992 | 2.41 | 4.52 | 1.28 | 0.48 | 1235917 | 3376967464 | 2067885171 | 617958.5 | 2067267212 | 0.61 |
| DALYs | Paraguay | 1992 | 27.63 | 40.38 | 18.33 | 0.48 | 2324234 | 3376967464 | 2070209405 | 1162117 | 2069047288 | 0.61 |
| DALYs | Cameroon | 2021 | 13.50 | 23.18 | 6.79 | 0.48 | 17450670 | 5105255047 | 469891104 | 8725333 | 461165771 | 0.09 |
| DALYs | Iran (Islamic Republic of) | 1992 | 15.52 | 22.25 | 10.79 | 0.48 | 32234900 | 3376967464 | 2102444303 | 16117450 | 2086326854 | 0.62 |
| DALYs | Colombia | 1992 | 34.56 | 39.87 | 29.83 | 0.48 | 20427380 | 3376967464 | 2122871685 | 10213690 | 2112657994 | 0.63 |
| DALYs | Saint Vincent and the Grenadines | 1992 | 28.34 | 34.78 | 22.98 | 0.49 | 63082.62 | 3376967464 | 2122934768 | 31541.31 | 2122903226 | 0.63 |
| DALYs | Djibouti | 2021 | 12.89 | 23.39 | 6.34 | 0.49 | 805751.6 | 5105255047 | 470696855 | 402875.8 | 470293979.5 | 0.09 |
| DALYs | Tunisia | 1992 | 14.15 | 26.12 | 3.86 | 0.49 | 5133459 | 3376967464 | 2128068226 | 2566729 | 2125501497 | 0.63 |
| DALYs | Lao People's Democratic Republic | 2021 | 1.50 | 3.77 | 0.54 | 0.49 | 4754006 | 5105255047 | 475450861 | 2377003 | 473073858.3 | 0.09 |
| DALYs | Bangladesh | 2021 | 9.60 | 16.29 | 5.22 | 0.49 | 107272700 | 5105255047 | 582723583 | 53636360 | 529087222.1 | 0.10 |

| | | | | | | | | | | | | |
|---|---|---|---|---|---|---|---|---|---|---|---|---|
| DALYs | Samoa | 1992 | 22.76 | 38.74 | 12.58 | 0.49 | 92361.26 | 3376967464 | 2128160588 | 46180.63 | 2128114407 | 0.63 |
| DALYs | Mauritania | 2021 | 10.45 | 18.39 | 5.51 | 0.50 | 2374531 | 5105255047 | 585098113 | 1187265 | 583910848.2 | 0.11 |
| DALYs | Democratic People's Republic of Korea | 1992 | 2.47 | 4.90 | 1.03 | 0.50 | 13875620 | 3376967464 | 2142036206 | 6937809 | 2135098397 | 0.63 |
| DALYs | Uzbekistan | 1992 | 1.99 | 2.75 | 1.39 | 0.50 | 12096580 | 3376967464 | 2154132781 | 6048288 | 2148084494 | 0.64 |
| DALYs | Nigeria | 2021 | 9.13 | 16.91 | 4.81 | 0.50 | 123377300 | 5105255047 | 708475425 | 61688660 | 646786769.3 | 0.13 |
| DALYs | Tonga | 1992 | 14.85 | 25.11 | 8.56 | 0.50 | 53374.01 | 3376967464 | 2154186155 | 26687 | 2154159468 | 0.64 |
| DALYs | Pakistan | 2021 | 12.31 | 19.05 | 7.64 | 0.50 | 141565600 | 5105255047 | 850041000 | 70782790 | 779258212.8 | 0.15 |
| DALYs | Sao Tome and Principe | 2021 | 12.07 | 24.21 | 5.86 | 0.51 | 131024.1 | 5105255047 | 850172025 | 65512.04 | 850106512.5 | 0.17 |
| DALYs | Zambia | 2021 | 16.92 | 29.01 | 9.17 | 0.51 | 10789070 | 5105255047 | 860961092 | 5394534 | 855566558.4 | 0.17 |
| DALYs | Brazil | 1992 | 38.02 | 40.29 | 35.83 | 0.51 | 93825900 | 3376967464 | 2248012056 | 46912950 | 2201099106 | 0.65 |
| DALYs | Lesotho | 2021 | 15.26 | 27.00 | 7.54 | 0.51 | 1159988 | 5105255047 | 862121080 | 579994.1 | 861541086.2 | 0.17 |
| DALYs | Suriname | 1992 | 17.12 | 26.16 | 10.25 | 0.51 | 240092.4 | 3376967464 | 2248252148 | 120046.2 | 2248132102 | 0.67 |
| DALYs | Honduras | 2021 | 17.61 | 27.14 | 10.45 | 0.51 | 6325127 | 5105255047 | 868446208 | 3162564 | 865283644 | 0.17 |
| DALYs | Peru | 1992 | 12.43 | 18.72 | 7.81 | 0.51 | 13017280 | 3376967464 | 2261269426 | 6508639 | 2254760787 | 0.67 |
| DALYs | Mexico | 1992 | 16.67 | 17.46 | 15.92 | 0.51 | 51527500 | 3376967464 | 2312796928 | 25763750 | 2287033177 | 0.68 |
| DALYs | Saint Lucia | 1992 | 24.72 | 29.47 | 20.41 | 0.52 | 80360.32 | 3376967464 | 2312877288 | 40180.16 | 2312837108 | 0.68 |
| DALYs | Philippines | 1992 | 1.61 | 2.16 | 1.28 | 0.52 | 37716100 | 3376967464 | 2350593387 | 18858050 | 2331735338 | 0.69 |
| DALYs | Ecuador | 1992 | 15.08 | 17.70 | 12.62 | 0.52 | 6008273 | 3376967464 | 2356601661 | 3004137 | 2353597524 | 0.70 |
| DALYs | Kenya | 2021 | 10.23 | 14.19 | 7.30 | 0.52 | 29742370 | 5105255047 | 898188583 | 14871190 | 883317395.1 | 0.17 |
| DALYs | Nicaragua | 2021 | 6.54 | 9.22 | 4.47 | 0.52 | 4297839 | 5105255047 | 902486421 | 2148919 | 900337502 | 0.18 |
| DALYs | Thailand | 1992 | 3.72 | 6.12 | 1.92 | 0.53 | 39057400 | 3376967464 | 2395659056 | 19528700 | 2376130359 | 0.70 |
| DALYs | Kiribati | 2021 | 66.53 | 112.47 | 35.79 | 0.53 | 74513.75 | 5105255047 | 902560935 | 37256.87 | 902523678.2 | 0.18 |
| DALYs | Kyrgyzstan | 1992 | 1.14 | 1.57 | 0.85 | 0.53 | 2635720 | 3376967464 | 2398294776 | 1317860 | 2396976916 | 0.71 |

| | | | | | | | | | | | | |
|---|---|---|---|---|---|---|---|---|---|---|---|---|
| DALYs | Venezuela (Bolivarian Republic of) | 1992 | 21.77 | 24.96 | 18.88 | 0.53 | 11607490 | 3376967464 | 2409902266 | 5803745 | 2404098521 | 0.71 |
| DALYs | Tokelau | 1992 | 28.43 | 52.35 | 14.57 | 0.53 | 834.3087 | 3376967464 | 2409903100 | 417.1543 | 2409902683 | 0.71 |
| DALYs | Sri Lanka | 1992 | 9.38 | 13.31 | 6.24 | 0.53 | 11121210 | 3376967464 | 2421024309 | 5560605 | 2415463705 | 0.72 |
| DALYs | Cabo Verde | 2021 | 10.80 | 20.74 | 5.46 | 0.53 | 382357.4 | 5105255047 | 902943292 | 191178.7 | 902752113.8 | 0.18 |
| DALYs | Myanmar | 2021 | 1.69 | 3.99 | 0.65 | 0.53 | 36996570 | 5105255047 | 939939864 | 18498290 | 921441578.5 | 0.18 |
| DALYs | Nauru | 1992 | 47.07 | 83.90 | 22.80 | 0.54 | 5770.117 | 3376967464 | 2421030079 | 2885.058 | 2421027194 | 0.72 |
| DALYs | Bosnia and Herzegovina | 1992 | 11.60 | 20.16 | 5.72 | 0.54 | 3112775 | 3376967464 | 2424142855 | 1556388 | 2422586467 | 0.72 |
| DALYs | Guatemala | 2021 | 8.78 | 10.92 | 6.97 | 0.54 | 9913414 | 5105255047 | 949853279 | 4956707 | 944896571.5 | 0.19 |
| DALYs | Tajikistan | 2021 | 0.41 | 0.78 | 0.20 | 0.54 | 6207893 | 5105255047 | 956061172 | 3103947 | 952957225.2 | 0.19 |
| DALYs | Lebanon | 1992 | 62.37 | 106.35 | 29.28 | 0.54 | 1853583 | 3376967464 | 2425996438 | 926791.5 | 2425069646 | 0.72 |
| DALYs | Sudan | 2021 | 15.63 | 30.51 | 4.60 | 0.54 | 25440510 | 5105255047 | 981501682 | 12720260 | 968781427 | 0.19 |
| DALYs | Fiji | 1992 | 7.93 | 12.53 | 4.72 | 0.54 | 465040.9 | 3376967464 | 2426461479 | 232520.5 | 2426228958 | 0.72 |
| DALYs | Costa Rica | 1992 | 23.50 | 27.18 | 20.34 | 0.54 | 1893843 | 3376967464 | 2428355322 | 946921.6 | 2427408400 | 0.72 |
| DALYs | Jamaica | 1992 | 2.83 | 3.74 | 2.17 | 0.55 | 1395283 | 3376967464 | 2429750605 | 697641.5 | 2429052964 | 0.72 |
| DALYs | Jordan | 1992 | 15.58 | 22.88 | 9.71 | 0.55 | 2205439 | 3376967464 | 2431956044 | 1102720 | 2430853325 | 0.72 |
| DALYs | Armenia | 1992 | 2.97 | 3.75 | 2.27 | 0.55 | 2180135 | 3376967464 | 2434136179 | 1090067 | 2433046112 | 0.72 |
| DALYs | South Africa | 1992 | 18.79 | 26.34 | 13.49 | 0.55 | 22998760 | 3376967464 | 2457134939 | 11499380 | 2445635559 | 0.72 |
| DALYs | Albania | 1992 | 5.41 | 7.80 | 3.41 | 0.55 | 2048832 | 3376967464 | 2459183771 | 1024416 | 2458159355 | 0.73 |
| DALYs | Mauritius | 1992 | 25.61 | 29.78 | 21.76 | 0.55 | 729511 | 3376967464 | 2459913282 | 364755.5 | 2459548527 | 0.73 |
| DALYs | Libya | 1992 | 13.59 | 26.10 | 3.71 | 0.55 | 2388892 | 3376967464 | 2462302174 | 1194446 | 2461107728 | 0.73 |
| DALYs | Panama | 1992 | 12.22 | 14.29 | 10.32 | 0.55 | 1507409 | 3376967464 | 2463809583 | 753704.7 | 2463055878 | 0.73 |
| DALYs | Malaysia | 1992 | 7.15 | 10.40 | 4.65 | 0.56 | 11151630 | 3376967464 | 2474961212 | 5575814 | 2469385397 | 0.73 |
| DALYs | Saudi Arabia | 1992 | 10.80 | 18.65 | 5.68 | 0.56 | 9752944 | 3376967464 | 2484714156 | 4876472 | 2479837684 | 0.73 |

| | | | | | | | | | | | | |
|---|---|---|---|---|---|---|---|---|---|---|---|---|
| DALYs | Morocco | 2021 | 14.10 | 27.27 | 4.37 | 0.56 | 24647580 | 5105255047 | 1006149263 | 12323790 | 993825472.4 | 0.19 |
| DALYs | El Salvador | 2021 | 6.59 | 9.50 | 4.38 | 0.56 | 4074428 | 5105255047 | 1010223691 | 2037214 | 1008186477 | 0.20 |
| DALYs | Ghana | 2021 | 13.71 | 23.59 | 7.35 | 0.56 | 20161140 | 5105255047 | 1030384835 | 10080570 | 1020304263 | 0.20 |
| DALYs | Turkmenistan | 1992 | 0.66 | 1.03 | 0.43 | 0.57 | 2146841 | 3376967464 | 2486860997 | 1073420 | 2485787576 | 0.74 |
| DALYs | Cuba | 1992 | 21.44 | 25.35 | 17.88 | 0.57 | 7554381 | 3376967464 | 2494415377 | 3777190 | 2490638187 | 0.74 |
| DALYs | Dominica | 1992 | 13.87 | 21.74 | 8.57 | 0.57 | 41551.19 | 3376967464 | 2494456928 | 20775.6 | 2494436153 | 0.74 |
| DALYs | Democratic People's Republic of Korea | 2021 | 2.13 | 4.16 | 1.01 | 0.57 | 18946650 | 5105255047 | 1049331482 | 9473323 | 1039858159 | 0.20 |
| DALYs | Marshall Islands | 2021 | 31.64 | 58.45 | 15.12 | 0.57 | 36532.79 | 5105255047 | 1049368015 | 18266.39 | 1049349748 | 0.21 |
| DALYs | India | 2021 | 10.55 | 13.56 | 7.78 | 0.58 | 950604900 | 5105255047 | 1999972931 | 475302500 | 1524670473 | 0.30 |
| DALYs | Tuvalu | 2021 | 27.21 | 46.01 | 15.54 | 0.58 | 7770.842 | 5105255047 | 1999980701 | 3885.421 | 1999976816 | 0.39 |
| DALYs | Cook Islands | 1992 | 15.59 | 28.62 | 8.44 | 0.58 | 11553.59 | 3376967464 | 2494468482 | 5776.796 | 2494462705 | 0.74 |
| DALYs | Congo | 2021 | 19.39 | 32.69 | 10.55 | 0.58 | 3289800 | 5105255047 | 2003270502 | 1644900 | 2001625602 | 0.39 |
| DALYs | Eswatini | 2021 | 16.15 | 29.23 | 7.52 | 0.59 | 702572.3 | 5105255047 | 2003973074 | 351286.1 | 2003621788 | 0.39 |
| DALYs | Micronesia (Federated States of) | 2021 | 29.80 | 50.58 | 16.12 | 0.59 | 67172.81 | 5105255047 | 2004040247 | 33586.4 | 2004006661 | 0.39 |
| DALYs | Uruguay | 1992 | 39.81 | 45.54 | 34.37 | 0.59 | 1978724 | 3376967464 | 2496447206 | 989362.1 | 2495457844 | 0.74 |
| DALYs | Seychelles | 1992 | 9.03 | 14.12 | 3.81 | 0.59 | 45508.48 | 3376967464 | 2496492715 | 22754.24 | 2496469961 | 0.74 |
| DALYs | Samoa | 2021 | 22.72 | 38.24 | 12.60 | 0.59 | 122652 | 5105255047 | 2004162899 | 61325.99 | 2004101573 | 0.39 |
| DALYs | Azerbaijan | 1992 | 1.82 | 2.83 | 1.12 | 0.59 | 4664389 | 3376967464 | 2501157104 | 2332195 | 2498824909 | 0.74 |
| DALYs | Bahrain | 1992 | 18.50 | 27.60 | 12.14 | 0.60 | 353670.8 | 3376967464 | 2501510775 | 176835.4 | 2501333939 | 0.74 |
| DALYs | Kazakhstan | 1992 | 3.08 | 3.87 | 2.53 | 0.60 | 10145610 | 3376967464 | 2511656388 | 5072806 | 2506583581 | 0.74 |
| DALYs | Venezuela (Bolivarian Republic of) | 2021 | 21.41 | 29.17 | 15.30 | 0.60 | 17426450 | 5105255047 | 2021589354 | 8713227 | 2012876126 | 0.39 |
| DALYs | Argentina | 1992 | 43.60 | 50.13 | 37.85 | 0.60 | 20617890 | 3376967464 | 2532274273 | 10308940 | 2521965330 | 0.75 |

| | | | | | | | | | | | | |
|---|---|---|---|---|---|---|---|---|---|---|---|---|
| DALYs | Bolivia (Plurinational State of) | 2021 | 17.17 | 28.51 | 9.94 | 0.60 | 7560756 | 5105255047 | 2029150109 | 3780378 | 2025369731 | 0.40 |
| DALYs | Chile | 1992 | 17.60 | 20.70 | 14.94 | 0.60 | 8721318 | 3376967464 | 2540995591 | 4360659 | 2536634932 | 0.75 |
| DALYs | Saint Kitts and Nevis | 1992 | 19.13 | 23.02 | 14.99 | 0.60 | 23875.01 | 3376967464 | 2541019466 | 11937.5 | 2541007529 | 0.75 |
| DALYs | Niue | 1992 | 27.53 | 47.46 | 14.99 | 0.60 | 1295.984 | 3376967464 | 2541020762 | 647.9919 | 2541020114 | 0.75 |
| DALYs | Kyrgyzstan | 2021 | 2.39 | 3.15 | 1.80 | 0.60 | 4248862 | 5105255047 | 2033398972 | 2124431 | 2031274540 | 0.40 |
| DALYs | Egypt | 2021 | 20.84 | 31.79 | 12.85 | 0.61 | 64397140 | 5105255047 | 2097796116 | 32198570 | 2065597544 | 0.40 |
| DALYs | Republic of Moldova | 1992 | 1.35 | 1.93 | 0.96 | 0.61 | 2854706 | 3376967464 | 2543875468 | 1427353 | 2542448115 | 0.75 |
| DALYs | Belize | 2021 | 5.87 | 7.43 | 4.55 | 0.61 | 283479.5 | 5105255047 | 2098079595 | 141739.7 | 2097937856 | 0.41 |
| DALYs | North Macedonia | 1992 | 4.39 | 6.96 | 2.94 | 0.61 | 1323446 | 3376967464 | 2545198915 | 661723.2 | 2544537192 | 0.75 |
| DALYs | Portugal | 1992 | 18.52 | 21.58 | 15.86 | 0.61 | 6780922 | 3376967464 | 2551979837 | 3390461 | 2548589376 | 0.75 |
| DALYs | Namibia | 2021 | 12.08 | 21.42 | 6.14 | 0.62 | 1504481 | 5105255047 | 2099584076 | 752240.5 | 2098831836 | 0.41 |
| DALYs | Mongolia | 2021 | 5.19 | 7.81 | 3.25 | 0.62 | 2105152 | 5105255047 | 2101689228 | 1052576 | 2100636652 | 0.41 |
| DALYs | American Samoa | 1992 | 31.98 | 51.34 | 18.57 | 0.62 | 29749.7 | 3376967464 | 2552009587 | 14874.85 | 2551994712 | 0.76 |
| DALYs | Dominican Republic | 2021 | 16.26 | 25.13 | 10.00 | 0.62 | 7231298 | 5105255047 | 2108920526 | 3615649 | 2105304877 | 0.41 |
| DALYs | Syrian Arab Republic | 2021 | 35.63 | 58.48 | 19.06 | 0.62 | 9352995 | 5105255047 | 2118273521 | 4676498 | 2113597023 | 0.41 |
| DALYs | Antigua and Barbuda | 1992 | 12.99 | 15.80 | 10.48 | 0.62 | 38493.21 | 3376967464 | 2552048080 | 19246.61 | 2552028833 | 0.76 |
| DALYs | Nauru | 2021 | 43.08 | 76.18 | 22.18 | 0.63 | 6647.105 | 5105255047 | 2118280168 | 3323.553 | 2118276845 | 0.41 |
| DALYs | Tonga | 2021 | 14.75 | 26.37 | 8.05 | 0.63 | 60598.31 | 5105255047 | 2118340767 | 30299.16 | 2118310467 | 0.41 |
| DALYs | Viet Nam | 2021 | 1.27 | 2.67 | 0.54 | 0.63 | 67836850 | 5105255047 | 2186177612 | 33918420 | 2152259189 | 0.42 |
| DALYs | Romania | 1992 | 8.97 | 11.03 | 7.26 | 0.63 | 15484770 | 3376967464 | 2567532852 | 7742386 | 2559790466 | 0.76 |
| DALYs | Palestine | 2021 | 6.60 | 10.05 | 4.20 | 0.63 | 3092703 | 5105255047 | 2189270315 | 1546351 | 2187723963 | 0.43 |
| DALYs | Belarus | 1992 | 1.36 | 1.95 | 0.98 | 0.63 | 6938289 | 3376967464 | 2574471141 | 3469144 | 2571001997 | 0.76 |
| DALYs | Suriname | 2021 | 15.28 | 24.94 | 8.63 | 0.63 | 384154.9 | 5105255047 | 2189654470 | 192077.4 | 2189462392 | 0.43 |
| DALYs | Trinidad and Tobago | 1992 | 11.30 | 13.55 | 9.39 | 0.63 | 745716.6 | 3376967464 | 2575216858 | 372858.3 | 2574843999 | 0.76 |

| | | | | | | | | | | | | |
|---|---|---|---|---|---|---|---|---|---|---|---|---|
| DALYs | Gabon | 2021 | 17.65 | 29.94 | 9.68 | 0.63 | 1102224 | 5105255047 | 2190756693 | 551111.8 | 2190205581 | 0.43 |
| DALYs | Paraguay | 2021 | 33.83 | 49.68 | 22.03 | 0.64 | 4680073 | 5105255047 | 2195436766 | 2340037 | 2193096730 | 0.43 |
| DALYs | Saint Vincent and the Grenadines | 2021 | 20.79 | 25.55 | 16.63 | 0.64 | 76552.59 | 5105255047 | 2195513319 | 38276.3 | 2195475043 | 0.43 |
| DALYs | Poland | 1992 | 7.38 | 7.84 | 6.99 | 0.64 | 24967360 | 3376967464 | 2600184220 | 12483680 | 2587700539 | 0.77 |
| DALYs | Serbia | 1992 | 17.62 | 27.25 | 10.64 | 0.64 | 6574516 | 3376967464 | 2606758736 | 3287258 | 2603471478 | 0.77 |
| DALYs | Botswana | 2021 | 8.90 | 15.60 | 4.53 | 0.64 | 1590402 | 5105255047 | 2197103721 | 795200.9 | 2196308520 | 0.43 |
| DALYs | Guyana | 2021 | 42.88 | 58.41 | 30.48 | 0.65 | 501391.8 | 5105255047 | 2197605113 | 250695.9 | 2197354417 | 0.43 |
| DALYs | Maldives | 2021 | 4.92 | 7.78 | 2.98 | 0.65 | 394937.9 | 5105255047 | 2198000050 | 197468.9 | 2197802582 | 0.43 |
| DALYs | Bulgaria | 1992 | 3.10 | 3.93 | 2.51 | 0.65 | 5699135 | 3376967464 | 2612457871 | 2849568 | 2609608303 | 0.77 |
| DALYs | Philippines | 2021 | 2.36 | 3.28 | 1.82 | 0.65 | 73079830 | 5105255047 | 2271079884 | 36539920 | 2234539967 | 0.44 |
| DALYs | Spain | 1992 | 41.62 | 48.07 | 36.01 | 0.65 | 26113210 | 3376967464 | 2638571077 | 13056600 | 2625514474 | 0.78 |
| DALYs | Brazil | 2021 | 21.37 | 22.76 | 20.12 | 0.65 | 150405800 | 5105255047 | 2421485716 | 75202920 | 2346282800 | 0.46 |
| DALYs | Colombia | 2021 | 23.22 | 29.13 | 18.20 | 0.66 | 33609590 | 5105255047 | 2455095302 | 16804790 | 2438290509 | 0.48 |
| DALYs | Indonesia | 2021 | 1.49 | 3.33 | 0.54 | 0.66 | 194150400 | 5105255047 | 2649245676 | 97075190 | 2552170489 | 0.50 |
| DALYs | Ukraine | 1992 | 1.99 | 2.65 | 1.54 | 0.66 | 34804310 | 3376967464 | 2673375387 | 17402160 | 2655973232 | 0.79 |
| DALYs | Equatorial Guinea | 2021 | 15.53 | 29.11 | 7.29 | 0.66 | 893663 | 5105255047 | 2650139339 | 446831.5 | 2649692507 | 0.52 |
| DALYs | Algeria | 2021 | 12.02 | 22.64 | 3.41 | 0.66 | 28054400 | 5105255047 | 2678193741 | 14027200 | 2664166540 | 0.52 |
| DALYs | Hungary | 1992 | 43.68 | 50.21 | 38.07 | 0.66 | 6927328 | 3376967464 | 2680302715 | 3463664 | 2676839051 | 0.79 |
| DALYs | Qatar | 1992 | 22.09 | 34.55 | 13.29 | 0.66 | 335643.3 | 3376967464 | 2680638358 | 167821.6 | 2680470537 | 0.79 |
| DALYs | Ecuador | 2021 | 15.92 | 21.00 | 11.72 | 0.66 | 11574520 | 5105255047 | 2689768262 | 5787261 | 2683981001 | 0.53 |
| DALYs | Peru | 2021 | 10.65 | 16.72 | 6.53 | 0.66 | 23885430 | 5105255047 | 2713653696 | 11942720 | 2701710979 | 0.53 |
| DALYs | Barbados | 1992 | 21.55 | 25.51 | 17.99 | 0.66 | 162757.9 | 3376967464 | 2680801116 | 81378.93 | 2680719737 | 0.79 |
| DALYs | Uzbekistan | 2021 | 4.87 | 6.10 | 3.82 | 0.66 | 22329360 | 5105255047 | 2735983057 | 11164680 | 2724818377 | 0.53 |
| DALYs | Iraq | 2021 | 18.83 | 32.38 | 8.93 | 0.66 | 26081420 | 5105255047 | 2762064476 | 13040710 | 2749023766 | 0.54 |

| | | | | | | | | | | | | |
|---|---|---|---|---|---|---|---|---|---|---|---|---|
| DALYs | Mexico | 2021 | 16.03 | 18.26 | 14.01 | 0.66 | 86646750 | 5105255047 | 2848711229 | 43323380 | 2805387853 | 0.55 |
| DALYs | Georgia | 1992 | 1.91 | 2.62 | 1.34 | 0.67 | 3570243 | 3376967464 | 2684371360 | 1785122 | 2682586238 | 0.79 |
| DALYs | Slovakia | 1992 | 9.78 | 14.26 | 6.61 | 0.67 | 3469779 | 3376967464 | 2687841139 | 1734890 | 2686106249 | 0.80 |
| DALYs | Cyprus | 1992 | 41.32 | 62.78 | 26.57 | 0.67 | 522214.5 | 3376967464 | 2688363353 | 261107.2 | 2688102246 | 0.80 |
| DALYs | Malta | 1992 | 11.62 | 13.56 | 9.90 | 0.67 | 249227.5 | 3376967464 | 2688612581 | 124613.8 | 2688487967 | 0.80 |
| DALYs | Guam | 1992 | 52.62 | 74.54 | 36.20 | 0.67 | 93009.67 | 3376967464 | 2688705591 | 46504.84 | 2688659086 | 0.80 |
| DALYs | Puerto Rico | 1992 | 32.69 | 38.36 | 27.84 | 0.67 | 2339319 | 3376967464 | 2691044910 | 1169660 | 2689875250 | 0.80 |
| DALYs | Cuba | 2021 | 19.64 | 23.96 | 15.77 | 0.67 | 7721598 | 5105255047 | 2856432827 | 3860799 | 2852572028 | 0.56 |
| DALYs | Grenada | 2021 | 27.25 | 34.90 | 20.95 | 0.67 | 71465.1 | 5105255047 | 2856504292 | 35732.55 | 2856468560 | 0.56 |
| DALYs | Kuwait | 1992 | 15.11 | 17.78 | 12.86 | 0.67 | 1142046 | 3376967464 | 2692186956 | 571023.1 | 2691615933 | 0.80 |
| DALYs | United States Virgin Islands | 1992 | 32.56 | 50.10 | 19.90 | 0.67 | 68428.68 | 3376967464 | 2692255385 | 34214.34 | 2692221171 | 0.80 |
| DALYs | Saint Lucia | 2021 | 15.15 | 19.38 | 11.58 | 0.67 | 127254.6 | 5105255047 | 2856631547 | 63627.3 | 2856567920 | 0.56 |
| DALYs | Montenegro | 1992 | 6.82 | 9.82 | 4.58 | 0.67 | 417608.2 | 3376967464 | 2692672993 | 208804.1 | 2692464189 | 0.80 |
| DALYs | Palau | 1992 | 25.49 | 43.45 | 12.75 | 0.67 | 10631.73 | 3376967464 | 2692683625 | 5315.866 | 2692678309 | 0.80 |
| DALYs | Fiji | 2021 | 7.07 | 11.51 | 4.06 | 0.68 | 595530.4 | 5105255047 | 2857227077 | 297765.2 | 2856929312 | 0.56 |
| DALYs | United Arab Emirates | 1992 | 60.98 | 96.56 | 35.65 | 0.68 | 1447269 | 3376967464 | 2694130893 | 723634.3 | 2693407259 | 0.80 |
| DALYs | Brunei Darussalam | 1992 | 16.57 | 25.13 | 10.38 | 0.68 | 172733 | 3376967464 | 2694303626 | 86366.52 | 2694217260 | 0.80 |
| DALYs | Croatia | 1992 | 11.15 | 13.01 | 9.55 | 0.68 | 3329713 | 3376967464 | 2697633339 | 1664857 | 2695968483 | 0.80 |
| DALYs | South Africa | 2021 | 14.20 | 19.27 | 10.69 | 0.68 | 37983600 | 5105255047 | 2895210682 | 18991800 | 2876218879 | 0.56 |
| DALYs | Lithuania | 1992 | 7.76 | 9.36 | 6.50 | 0.68 | 2447071 | 3376967464 | 2700080410 | 1223536 | 2698856875 | 0.80 |
| DALYs | Turkmenistan | 2021 | 3.19 | 4.28 | 2.31 | 0.68 | 3350351 | 5105255047 | 2898561033 | 1675176 | 2896885857 | 0.57 |
| DALYs | Tunisia | 2021 | 12.07 | 23.86 | 3.55 | 0.68 | 7946659 | 5105255047 | 2906507692 | 3973329 | 2902534362 | 0.57 |
| DALYs | Thailand | 2021 | 7.04 | 11.31 | 4.12 | 0.68 | 47274310 | 5105255047 | 2953782004 | 23637160 | 2930144848 | 0.57 |
| DALYs | Jamaica | 2021 | 4.32 | 5.96 | 3.05 | 0.68 | 1946551 | 5105255047 | 2955728554 | 973275.3 | 2954755279 | 0.58 |

| | | | | | | | | | | | | |
|---|---|---|---|---|---|---|---|---|---|---|---|---|
| DALYs | Taiwan (Province of China) | 1992 | 12.17 | 14.13 | 10.43 | 0.68 | 14026120 | 3376967464 | 2714106526 | 7013058 | 2707093468 | 0.80 |
| DALYs | Tokelau | 2021 | 32.13 | 55.64 | 18.41 | 0.69 | 843.5528 | 5105255047 | 2955729398 | 421.7764 | 2955728976 | 0.58 |
| DALYs | Greece | 1992 | 17.01 | 19.53 | 14.77 | 0.69 | 7122789 | 3376967464 | 2721229315 | 3561394 | 2717667921 | 0.80 |
| DALYs | Bahamas | 1992 | 15.92 | 19.03 | 13.06 | 0.69 | 171499.1 | 3376967464 | 2721400814 | 85749.56 | 2721315065 | 0.81 |
| DALYs | Russian Federation | 1992 | 4.63 | 5.19 | 4.27 | 0.69 | 101681600 | 3376967464 | 2823082430 | 50840810 | 2772241622 | 0.82 |
| DALYs | Azerbaijan | 2021 | 1.35 | 2.12 | 0.86 | 0.69 | 7440503 | 5105255047 | 2963169901 | 3720252 | 2959449649 | 0.58 |
| DALYs | Estonia | 1992 | 20.85 | 25.35 | 17.46 | 0.70 | 1018565 | 3376967464 | 2824100995 | 509282.4 | 2823591712 | 0.84 |
| DALYs | Latvia | 1992 | 12.42 | 14.48 | 10.68 | 0.70 | 1746906 | 3376967464 | 2825847901 | 873453 | 2824974448 | 0.84 |
| DALYs | Iran (Islamic Republic of) | 2021 | 12.07 | 17.13 | 9.33 | 0.70 | 59167520 | 5105255047 | 3022337423 | 29583760 | 2992753662 | 0.59 |
| DALYs | Czechia | 1992 | 7.62 | 8.88 | 6.55 | 0.70 | 6884471 | 3376967464 | 2832732372 | 3442236 | 2829290136 | 0.84 |
| DALYs | Costa Rica | 2021 | 27.21 | 32.75 | 22.18 | 0.70 | 3249833 | 5105255047 | 3025587256 | 1624917 | 3023962339 | 0.59 |
| DALYs | Sri Lanka | 2021 | 5.46 | 8.91 | 3.05 | 0.70 | 14714740 | 5105255047 | 3040301997 | 7357370 | 3032944626 | 0.59 |
| DALYs | Armenia | 2021 | 3.95 | 4.75 | 3.20 | 0.70 | 2004496 | 5105255047 | 3042306493 | 1002248 | 3041304245 | 0.60 |
| DALYs | Singapore | 1992 | 9.92 | 11.63 | 8.44 | 0.70 | 2369939 | 3376967464 | 2835102310 | 1184969 | 2833917341 | 0.84 |
| DALYs | Bermuda | 1992 | 109.48 | 129.48 | 90.02 | 0.70 | 42685.4 | 3376967464 | 2835144996 | 21342.7 | 2835123653 | 0.84 |
| DALYs | Albania | 2021 | 7.30 | 11.04 | 4.67 | 0.71 | 1808384 | 5105255047 | 3044114877 | 904192 | 3043210685 | 0.60 |
| DALYs | Panama | 2021 | 15.51 | 19.83 | 11.57 | 0.71 | 2749507 | 5105255047 | 3046864384 | 1374753 | 3045489630 | 0.60 |
| DALYs | Republic of Korea | 1992 | 7.39 | 11.00 | 4.60 | 0.71 | 31435440 | 3376967464 | 2866580440 | 15717720 | 2850862718 | 0.84 |
| DALYs | Turkey | 2021 | 16.38 | 25.28 | 9.89 | 0.71 | 56921960 | 5105255047 | 3103786340 | 28460980 | 3075325362 | 0.60 |
| DALYs | Northern Mariana Islands | 1992 | 46.94 | 73.29 | 28.28 | 0.72 | 33889.48 | 3376967464 | 2866614329 | 16944.74 | 2866597384 | 0.85 |
| DALYs | Italy | 1992 | 21.67 | 23.05 | 20.42 | 0.72 | 38983200 | 3376967464 | 2905597533 | 19491600 | 2886105931 | 0.85 |
| DALYs | Israel | 1992 | 19.93 | 23.21 | 16.89 | 0.72 | 3155772 | 3376967464 | 2908753305 | 1577886 | 2907175419 | 0.86 |
| DALYs | Mauritius | 2021 | 23.19 | 27.18 | 19.38 | 0.72 | 900058.5 | 5105255047 | 3104686398 | 450029.2 | 3104236369 | 0.61 |
| DALYs | Uruguay | 2021 | 39.12 | 45.55 | 33.38 | 0.72 | 2214747 | 5105255047 | 3106901145 | 1107373 | 3105793772 | 0.61 |

| | | | | | | | | | | | |
|---|---|---|---|---|---|---|---|---|---|---|---|
| DALYs | China | 2021 | 1.24 | 1.68 | 0.93 | 0.72 | 967106700 | 5105255047 | 4074007884 | 483553400 | 3590454514 | 0.70 |
| DALYs | Bosnia and Herzegovina | 2021 | 10.94 | 17.63 | 6.13 | 0.72 | 2206873 | 5105255047 | 4076214757 | 1103437 | 4075111320 | 0.80 |
| DALYs | Argentina | 2021 | 24.85 | 28.80 | 21.30 | 0.72 | 30052210 | 5105255047 | 4106266964 | 15026100 | 4091240861 | 0.80 |
| DALYs | American Samoa | 2021 | 28.75 | 45.17 | 16.59 | 0.72 | 31908.17 | 5105255047 | 4106298872 | 15954.09 | 4106282918 | 0.80 |
| DALYs | Kazakhstan | 2021 | 3.65 | 4.68 | 2.82 | 0.73 | 12126520 | 5105255047 | 4118425393 | 6063260 | 4112362132 | 0.81 |
| DALYs | Jordan | 2021 | 8.43 | 12.56 | 5.52 | 0.73 | 8180007 | 5105255047 | 4126605399 | 4090003 | 4122515396 | 0.81 |
| DALYs | Libya | 2021 | 21.08 | 46.36 | 4.91 | 0.73 | 5029432 | 5105255047 | 4131634831 | 2514716 | 4129120115 | 0.81 |
| DALYs | Niue | 2021 | 29.81 | 52.28 | 17.10 | 0.73 | 1099.18 | 5105255047 | 4131635930 | 549.5901 | 4131635381 | 0.81 |
| DALYs | Greenland | 1992 | 59.82 | 95.58 | 29.49 | 0.73 | 38395.59 | 3376967464 | 2908791700 | 19197.79 | 2908772503 | 0.86 |
| DALYs | Seychelles | 2021 | 5.98 | 9.44 | 2.45 | 0.73 | 72957.22 | 5105255047 | 4131708887 | 36478.61 | 4131672409 | 0.81 |
| DALYs | Ireland | 1992 | 20.28 | 23.71 | 17.30 | 0.73 | 2251651 | 3376967464 | 2911043351 | 1125825 | 2909917526 | 0.86 |
| DALYs | Republic of Moldova | 2021 | 4.02 | 5.17 | 3.14 | 0.73 | 2516382 | 5105255047 | 4134225269 | 1258191 | 4132967078 | 0.81 |
| DALYs | Georgia | 2021 | 14.42 | 17.83 | 11.47 | 0.73 | 2299321 | 5105255047 | 4136524590 | 1149660 | 4135374929 | 0.81 |
| DALYs | Australia | 1992 | 21.22 | 24.47 | 18.39 | 0.73 | 11484530 | 3376967464 | 2922527885 | 5742267 | 2916785618 | 0.86 |
| DALYs | Slovenia | 1992 | 34.81 | 39.83 | 30.29 | 0.74 | 1357830 | 3376967464 | 2923885715 | 678914.9 | 2923206800 | 0.87 |
| DALYs | Malaysia | 2021 | 6.19 | 9.15 | 4.01 | 0.74 | 21856660 | 5105255047 | 4158381246 | 10928330 | 4147452918 | 0.81 |
| DALYs | France | 1992 | 36.30 | 42.19 | 31.06 | 0.74 | 38242170 | 3376967464 | 2962127885 | 19121090 | 2943006800 | 0.87 |
| DALYs | Portugal | 2021 | 17.15 | 19.74 | 14.81 | 0.74 | 6825510 | 5105255047 | 4165206755 | 3412755 | 4161794000 | 0.82 |
| DALYs | Lebanon | 2021 | 28.16 | 41.72 | 18.23 | 0.74 | 3716347 | 5105255047 | 4168923102 | 1858174 | 4167064929 | 0.82 |
| DALYs | Barbados | 2021 | 19.72 | 26.48 | 14.56 | 0.75 | 202783.5 | 5105255047 | 4169125886 | 101391.7 | 4169024494 | 0.82 |
| DALYs | Dominica | 2021 | 17.02 | 27.48 | 10.00 | 0.75 | 46130.66 | 5105255047 | 4169172016 | 23065.33 | 4169148951 | 0.82 |
| DALYs | Belgium | 1992 | 29.79 | 34.56 | 25.45 | 0.75 | 6685776 | 3376967464 | 2968813661 | 3342888 | 2965470773 | 0.88 |
| DALYs | Antigua and Barbuda | 2021 | 9.30 | 11.26 | 7.61 | 0.75 | 63578.22 | 5105255047 | 4169235595 | 31789.11 | 4169203806 | 0.82 |
| DALYs | North Macedonia | 2021 | 7.32 | 10.60 | 4.92 | 0.75 | 1541196 | 5105255047 | 4170776791 | 770598.2 | 4170006193 | 0.82 |

| | | | | | | | | | | | |
|---|---|---|---|---|---|---|---|---|---|---|---|
| DALYs | Bahrain | 2021 | 13.41 | 19.75 | 9.01 | 0.75 | 1178377 | 5105255047 | 4171955168 | 589188.4 | 4171365980 | 0.82 |
| DALYs | Palau | 2021 | 25.64 | 43.95 | 14.25 | 0.75 | 13185.18 | 5105255047 | 4171968353 | 6592.589 | 4171961761 | 0.82 |
| DALYs | Austria | 1992 | 25.87 | 30.07 | 22.10 | 0.75 | 5270737 | 3376967464 | 2974084397 | 2635368 | 2971449029 | 0.88 |
| DALYs | United Kingdom | 1992 | 22.88 | 23.69 | 22.16 | 0.75 | 37397090 | 3376967464 | 3011481485 | 18698540 | 2992782941 | 0.89 |
| DALYs | Saint Kitts and Nevis | 2021 | 12.43 | 16.57 | 9.20 | 0.75 | 43420.68 | 5105255047 | 4172011774 | 21710.34 | 4171990064 | 0.82 |
| DALYs | New Zealand | 1992 | 27.20 | 30.85 | 23.92 | 0.76 | 2285123 | 3376967464 | 3013766608 | 1142561 | 3012624047 | 0.89 |
| DALYs | Ukraine | 2021 | 7.71 | 10.64 | 5.30 | 0.76 | 29287980 | 5105255047 | 4201299752 | 14643990 | 4186655763 | 0.82 |
| DALYs | Finland | 1992 | 29.07 | 33.94 | 24.68 | 0.76 | 3388185 | 3376967464 | 3017154793 | 1694093 | 3015460701 | 0.89 |
| DALYs | Andorra | 1992 | 16.07 | 25.71 | 9.33 | 0.77 | 45683.08 | 3376967464 | 3017200476 | 22841.54 | 3017177635 | 0.89 |
| DALYs | Bulgaria | 2021 | 11.75 | 14.35 | 9.54 | 0.77 | 4337506 | 5105255047 | 4205637258 | 2168753 | 4203468505 | 0.82 |
| DALYs | Romania | 2021 | 12.91 | 16.07 | 10.28 | 0.77 | 12142540 | 5105255047 | 4217779801 | 6071271 | 4211708529 | 0.82 |
| DALYs | Trinidad and Tobago | 2021 | 12.86 | 17.42 | 9.29 | 0.77 | 942563.1 | 5105255047 | 4218722364 | 471281.5 | 4218251082 | 0.83 |
| DALYs | Spain | 2021 | 17.37 | 20.07 | 14.93 | 0.77 | 29873650 | 5105255047 | 4248596015 | 14936830 | 4233659190 | 0.83 |
| DALYs | United States of America | 1992 | 32.78 | 34.14 | 31.55 | 0.77 | 169626400 | 3376967464 | 3186826882 | 84813200 | 3102013679 | 0.92 |
| DALYs | Chile | 2021 | 11.26 | 13.33 | 9.43 | 0.77 | 12819950 | 5105255047 | 4261415966 | 6409975 | 4255005991 | 0.83 |
| DALYs | Northern Mariana Islands | 2021 | 42.63 | 64.11 | 27.09 | 0.77 | 33644 | 5105255047 | 4261449610 | 16822 | 4261432788 | 0.83 |
| DALYs | Iceland | 1992 | 22.04 | 25.73 | 18.77 | 0.77 | 167194.9 | 3376967464 | 3186994077 | 83597.46 | 3186910480 | 0.94 |
| DALYs | Oman | 2021 | 4.73 | 7.78 | 2.66 | 0.77 | 3365550 | 5105255047 | 4264815160 | 1682775 | 4263132385 | 0.84 |
| DALYs | Cook Islands | 2021 | 10.58 | 21.38 | 5.30 | 0.78 | 11633.24 | 5105255047 | 4264826793 | 5816.619 | 4264820977 | 0.84 |
| DALYs | Belarus | 2021 | 3.60 | 4.74 | 2.76 | 0.78 | 6254502 | 5105255047 | 4271081295 | 3127251 | 4267954044 | 0.84 |
| DALYs | Canada | 1992 | 19.05 | 21.94 | 16.35 | 0.79 | 18901940 | 3376967464 | 3205896013 | 9450968 | 3196445045 | 0.95 |
| DALYs | Luxembourg | 1992 | 34.03 | 39.30 | 29.34 | 0.79 | 268190.4 | 3376967464 | 3206164203 | 134095.2 | 3206030108 | 0.95 |
| DALYs | Hungary | 2021 | 48.96 | 56.70 | 42.09 | 0.79 | 6199148 | 5105255047 | 4277280443 | 3099574 | 4274180869 | 0.84 |
| DALYs | Greece | 2021 | 18.36 | 21.09 | 15.87 | 0.79 | 6469340 | 5105255047 | 4283749783 | 3234670 | 4280515113 | 0.84 |

| | | | | | | | | | | | | |
|---|---|---|---|---|---|---|---|---|---|---|---|---|
| DALYs | Serbia | 2021 | 19.75 | 28.64 | 13.11 | 0.79 | 5934126 | 5105255047 | 4289683909 | 2967063 | 4286716846 | 0.84 |
| DALYs | Sweden | 1992 | 21.50 | 24.39 | 18.81 | 0.80 | 5553552 | 3376967464 | 3211717756 | 2776776 | 3208940980 | 0.95 |
| DALYs | Montenegro | 2021 | 6.75 | 9.90 | 4.52 | 0.80 | 412912.2 | 5105255047 | 4290096822 | 206456.1 | 4289890365 | 0.84 |
| DALYs | Croatia | 2021 | 24.90 | 30.06 | 20.43 | 0.80 | 2715472 | 5105255047 | 4292812293 | 1357736 | 4291454558 | 0.84 |
| DALYs | Japan | 1992 | 25.55 | 26.69 | 24.47 | 0.80 | 88370150 | 3376967464 | 3300087906 | 44185070 | 3255902831 | 0.96 |
| DALYs | Malta | 2021 | 13.07 | 15.51 | 10.93 | 0.80 | 278137.3 | 5105255047 | 4293090431 | 139068.6 | 4292951362 | 0.84 |
| DALYs | Netherlands | 1992 | 26.26 | 30.63 | 22.39 | 0.80 | 10396300 | 3376967464 | 3310484202 | 5198148 | 3305286054 | 0.98 |
| DALYs | Guam | 2021 | 39.16 | 54.27 | 27.71 | 0.80 | 103477.4 | 5105255047 | 4293193908 | 51738.72 | 4293142170 | 0.84 |
| DALYs | Bahamas | 2021 | 16.58 | 21.65 | 12.52 | 0.81 | 274938.4 | 5105255047 | 4293468847 | 137469.2 | 4293331377 | 0.84 |
| DALYs | Norway | 1992 | 22.82 | 24.19 | 21.52 | 0.81 | 2768970 | 3376967464 | 3313253172 | 1384485 | 3311868687 | 0.98 |
| DALYs | Italy | 2021 | 15.53 | 16.76 | 14.39 | 0.81 | 38186670 | 5105255047 | 4331655515 | 19093330 | 4312562181 | 0.84 |
| DALYs | Denmark | 1992 | 23.30 | 27.23 | 19.84 | 0.81 | 3484450 | 3376967464 | 3316737622 | 1742225 | 3314995397 | 0.98 |
| DALYs | Russian Federation | 2021 | 16.10 | 17.78 | 14.54 | 0.81 | 96028800 | 5105255047 | 4427684315 | 48014400 | 4379669915 | 0.86 |
| DALYs | Israel | 2021 | 15.13 | 17.69 | 12.73 | 0.81 | 5769267 | 5105255047 | 4433453582 | 2884633 | 4430568949 | 0.87 |
| DALYs | Brunei Darussalam | 2021 | 15.76 | 23.08 | 10.41 | 0.81 | 332019.1 | 5105255047 | 4433785601 | 166009.6 | 4433619592 | 0.87 |
| DALYs | Slovakia | 2021 | 14.16 | 19.84 | 9.73 | 0.81 | 3635982 | 5105255047 | 4437421583 | 1817991 | 4435603592 | 0.87 |
| DALYs | Poland | 2021 | 27.54 | 30.40 | 24.90 | 0.81 | 25179790 | 5105255047 | 4462601375 | 12589900 | 4450011479 | 0.87 |
| DALYs | Saudi Arabia | 2021 | 12.97 | 21.75 | 7.22 | 0.82 | 29117100 | 5105255047 | 4491718473 | 14558550 | 4477159924 | 0.88 |
| DALYs | Bermuda | 2021 | 62.29 | 78.73 | 48.71 | 0.82 | 41969.77 | 5105255047 | 4491760443 | 20984.88 | 4491739458 | 0.88 |
| DALYs | United States Virgin Islands | 2021 | 24.60 | 43.63 | 13.07 | 0.82 | 53866.94 | 5105255047 | 4491814310 | 26933.47 | 4491787376 | 0.88 |
| DALYs | San Marino | 1992 | 7.67 | 11.19 | 5.04 | 0.82 | 16789.73 | 3376967464 | 3316754411 | 8394.865 | 3316746017 | 0.98 |
| DALYs | Puerto Rico | 2021 | 31.52 | 39.56 | 24.52 | 0.83 | 2124836 | 5105255047 | 4493939146 | 1062418 | 4492876728 | 0.88 |
| DALYs | Greenland | 2021 | 31.50 | 47.98 | 18.48 | 0.83 | 39064.81 | 5105255047 | 4493978210 | 19532.41 | 4493958678 | 0.88 |
| DALYs | Germany | 1992 | 36.65 | 42.96 | 31.20 | 0.83 | 55437780 | 3376967464 | 3372192189 | 27718890 | 3344473300 | 0.99 |

| | | | | | | | | | | | | |
|---|---|---|---|---|---|---|---|---|---|---|---|---|
| DALYs | Czechia | 2021 | 22.93 | 27.25 | 18.94 | 0.83 | 6708178 | 5105255047 | 4500686388 | 3354089 | 4497332299 | 0.88 |
| DALYs | Latvia | 2021 | 29.43 | 35.30 | 24.26 | 0.83 | 1180840 | 5105255047 | 4501867228 | 590420 | 4501276808 | 0.88 |
| DALYs | Cyprus | 2021 | 24.62 | 35.92 | 16.62 | 0.84 | 940775.7 | 5105255047 | 4502808004 | 470387.8 | 4502337616 | 0.88 |
| DALYs | France | 2021 | 16.05 | 18.91 | 13.54 | 0.84 | 41021790 | 5105255047 | 4543829797 | 20510900 | 4523318900 | 0.89 |
| DALYs | Slovenia | 2021 | 45.71 | 55.09 | 36.82 | 0.84 | 1320405 | 5105255047 | 4545150201 | 660202.5 | 4544489999 | 0.89 |
| DALYs | Australia | 2021 | 12.10 | 14.13 | 10.28 | 0.84 | 16656050 | 5105255047 | 4561806249 | 8328024 | 4553478225 | 0.89 |
| DALYs | Estonia | 2021 | 40.33 | 48.60 | 33.42 | 0.84 | 825205.3 | 5105255047 | 4562631454 | 412602.6 | 4562218851 | 0.89 |
| DALYs | Kuwait | 2021 | 10.72 | 13.38 | 8.53 | 0.85 | 3633714 | 5105255047 | 4566265168 | 1816857 | 4564448311 | 0.89 |
| DALYs | Qatar | 2021 | 10.08 | 16.68 | 5.60 | 0.85 | 2446107 | 5105255047 | 4568711276 | 1223054 | 4567488222 | 0.89 |
| DALYs | United Arab Emirates | 2021 | 26.27 | 39.90 | 16.57 | 0.85 | 8129433 | 5105255047 | 4576840709 | 4064717 | 4572775992 | 0.90 |
| DALYs | New Zealand | 2021 | 19.03 | 21.55 | 16.79 | 0.85 | 3378026 | 5105255047 | 4580218735 | 1689013 | 4578529722 | 0.90 |
| DALYs | Monaco | 1992 | 9.48 | 14.77 | 5.66 | 0.85 | 19942.64 | 3376967464 | 3372212132 | 9971.318 | 3372202160 | 1.00 |
| DALYs | Belgium | 2021 | 20.98 | 24.35 | 18.02 | 0.85 | 7314275 | 5105255047 | 4587533010 | 3657137 | 4583875872 | 0.90 |
| DALYs | Austria | 2021 | 15.41 | 18.13 | 13.03 | 0.85 | 5970418 | 5105255047 | 4593503427 | 2985209 | 4590518219 | 0.90 |
| DALYs | Singapore | 2021 | 3.31 | 3.95 | 2.78 | 0.86 | 4146887 | 5105255047 | 4597650315 | 2073444 | 4595576871 | 0.90 |
| DALYs | Lithuania | 2021 | 21.94 | 26.03 | 18.13 | 0.86 | 1763517 | 5105255047 | 4599413832 | 881758.6 | 4598532074 | 0.90 |
| DALYs | United Kingdom | 2021 | 19.91 | 20.77 | 19.09 | 0.86 | 43558640 | 5105255047 | 4642972474 | 21779320 | 4621193153 | 0.91 |
| DALYs | Finland | 2021 | 22.54 | 26.50 | 19.00 | 0.86 | 3397926 | 5105255047 | 4646370400 | 1698963 | 4644671437 | 0.91 |
| DALYs | United States of America | 2021 | 18.36 | 19.42 | 17.34 | 0.86 | 216097500 | 5105255047 | 4862467891 | 108048700 | 4754419146 | 0.93 |
| DALYs | Switzerland | 1992 | 19.12 | 22.41 | 16.12 | 0.87 | 4755332 | 3376967464 | 3376967464 | 2377666 | 3374589798 | 1.00 |
| DALYs | Andorra | 2021 | 9.52 | 15.82 | 5.44 | 0.87 | 61742.59 | 5105255047 | 4862529634 | 30871.3 | 4862498763 | 0.95 |
| DALYs | Japan | 2021 | 8.38 | 8.88 | 7.94 | 0.87 | 75404150 | 5105255047 | 4937933788 | 37702080 | 4900231711 | 0.96 |
| DALYs | Canada | 2021 | 12.63 | 14.82 | 10.74 | 0.87 | 24262340 | 5105255047 | 4962196124 | 12131170 | 4950064956 | 0.97 |
| DALYs | Ireland | 2021 | 13.27 | 15.91 | 10.99 | 0.87 | 3192765 | 5105255047 | 4965388890 | 1596383 | 4963792507 | 0.97 |

| | | | | | | | | | | | | |
|---|---|---|---|---|---|---|---|---|---|---|---|---|
| DALYs | Taiwan (Province of China) | 2021 | 8.30 | 9.68 | 7.00 | 0.87 | 16681750 | 5105255047 | 4982070642 | 8340876 | 4973729766 | 0.97 |
| DALYs | Iceland | 2021 | 23.39 | 27.87 | 19.20 | 0.88 | 227618.6 | 5105255047 | 4982298260 | 113809.3 | 4982184451 | 0.98 |
| DALYs | Luxembourg | 2021 | 21.54 | 25.35 | 18.18 | 0.88 | 447039.5 | 5105255047 | 4982745300 | 223519.8 | 4982521780 | 0.98 |
| DALYs | Republic of Korea | 2021 | 4.20 | 5.82 | 2.98 | 0.89 | 36999740 | 5105255047 | 5019745039 | 18499870 | 5001245169 | 0.98 |
| DALYs | Sweden | 2021 | 10.91 | 13.12 | 8.99 | 0.89 | 6417083 | 5105255047 | 5026162122 | 3208541 | 5022953580 | 0.98 |
| DALYs | San Marino | 2021 | 5.31 | 8.71 | 2.86 | 0.89 | 21290.2 | 5105255047 | 5026183412 | 10645.1 | 5026172767 | 0.98 |
| DALYs | Netherlands | 2021 | 15.90 | 18.54 | 13.53 | 0.89 | 11069920 | 5105255047 | 5037253336 | 5534962 | 5031718374 | 0.99 |
| DALYs | Denmark | 2021 | 18.53 | 21.67 | 15.62 | 0.90 | 3719018 | 5105255047 | 5040972354 | 1859509 | 5039112845 | 0.99 |
| DALYs | Germany | 2021 | 22.90 | 26.77 | 19.37 | 0.90 | 54851780 | 5105255047 | 5095824131 | 27425890 | 5068398243 | 0.99 |
| DALYs | Monaco | 2021 | 7.80 | 12.70 | 4.46 | 0.91 | 23218.3 | 5105255047 | 5095847350 | 11609.15 | 5095835740 | 1.00 |
| DALYs | Norway | 2021 | 14.43 | 15.71 | 13.24 | 0.92 | 3522494 | 5105255047 | 5099369844 | 1761247 | 5097608597 | 1.00 |
| DALYs | Switzerland | 2021 | 11.36 | 13.38 | 9.55 | 0.93 | 5885204 | 5105255047 | 5105255047 | 2942602 | 5102312446 | 1.00 |
| DALYs | Somalia | 1992 | 9.76 | 20.26 | 3.48 | 0.05 | 3816581 | 3376967464 | 3816581 | 1908290 | 1908290.3 | 0.00 |
| DALYs | Somalia | 2021 | 6.93 | 14.27 | 2.54 | 0.08 | 10890630 | 5105255047 | 10890631 | 5445315 | 5445315.5 | 0.00 |
| DALYs | Niger | 1992 | 5.45 | 10.70 | 2.19 | 0.08 | 4059013 | 3376967464 | 7875594 | 2029507 | 5846087.2 | 0.00 |
| DALYs | Chad | 1992 | 6.15 | 11.74 | 2.68 | 0.12 | 3037794 | 3376967464 | 10913388 | 1518897 | 9394490.9 | 0.00 |
| DALYs | Mali | 1992 | 7.05 | 13.92 | 3.01 | 0.13 | 4416030 | 3376967464 | 15329418 | 2208015 | 13121403 | 0.00 |
| DALYs | Burkina Faso | 1992 | 7.27 | 13.68 | 3.20 | 0.14 | 4697926 | 3376967464 | 20027344 | 2348963 | 17678381.3 | 0.01 |
| DALYs | Ethiopia | 1992 | 8.77 | 16.17 | 4.25 | 0.15 | 27028040 | 3376967464 | 47055389 | 13514020 | 33541366.7 | 0.01 |
| DALYs | Niger | 2021 | 5.04 | 9.74 | 1.87 | 0.17 | 11701660 | 5105255047 | 22592286 | 5850828 | 16741458.7 | 0.00 |
| DALYs | Mozambique | 1992 | 10.73 | 19.11 | 5.10 | 0.18 | 6841201 | 3376967464 | 53896590 | 3420600 | 50475989.3 | 0.01 |
| DALYs | Afghanistan | 1992 | 15.66 | 40.76 | 3.40 | 0.18 | 5317936 | 3376967464 | 59214526 | 2658968 | 56555557.9 | 0.02 |
| DALYs | Guinea | 1992 | 7.80 | 14.65 | 3.45 | 0.18 | 3194335 | 3376967464 | 62408861 | 1597167 | 60811693.6 | 0.02 |
| DALYs | Uganda | 1992 | 9.38 | 18.12 | 3.98 | 0.19 | 8871780 | 3376967464 | 71280641 | 4435890 | 66844751.1 | 0.02 |

| | | | | | | | | | | | | |
|---|---|---|---|---|---|---|---|---|---|---|---|---|
| DALYs | Malawi | 1992 | 9.08 | 16.39 | 4.42 | 0.21 | 5373904 | 3376967464 | 76654545 | 2686952 | 73967593.3 | 0.02 |
| DALYs | Burundi | 1992 | 15.77 | 31.19 | 6.77 | 0.21 | 2868701 | 3376967464 | 79523246 | 1434350 | 78088895.8 | 0.02 |
| DALYs | South Sudan | 1992 | 9.89 | 19.06 | 4.19 | 0.21 | 3170246 | 3376967464 | 82693492 | 1585123 | 81108369 | 0.02 |
| DALYs | Nepal | 1992 | 6.36 | 11.90 | 2.88 | 0.21 | 10843820 | 3376967464 | 93537312 | 5421910 | 88115402.1 | 0.03 |
| DALYs | Sierra Leone | 1992 | 7.73 | 14.58 | 3.46 | 0.21 | 2160724 | 3376967464 | 95698036 | 1080362 | 94617674.1 | 0.03 |
| DALYs | Guinea-Bissau | 1992 | 11.99 | 22.92 | 5.15 | 0.22 | 518730.4 | 3376967464 | 96216766 | 259365.2 | 95957401.1 | 0.03 |
| DALYs | Central African Republic | 1992 | 14.39 | 28.00 | 6.11 | 0.22 | 1511635 | 3376967464 | 97728401 | 755817.3 | 96972583.7 | 0.03 |
| DALYs | Benin | 1992 | 6.06 | 10.83 | 2.91 | 0.23 | 2437709 | 3376967464 | 100166110 | 1218855 | 98947255.7 | 0.03 |
| DALYs | Bhutan | 1992 | 5.71 | 11.36 | 2.24 | 0.23 | 362111 | 3376967464 | 100528221 | 181055.5 | 100347165.9 | 0.03 |
| DALYs | Eritrea | 1992 | 13.29 | 25.83 | 5.50 | 0.23 | 1636011 | 3376967464 | 102164232 | 818005.3 | 101346226.7 | 0.03 |
| DALYs | Yemen | 1992 | 10.70 | 24.97 | 2.34 | 0.23 | 6649314 | 3376967464 | 108813546 | 3324657 | 105488888.8 | 0.03 |
| DALYs | Liberia | 1992 | 8.96 | 16.08 | 4.36 | 0.24 | 970267.7 | 3376967464 | 109783813 | 485133.8 | 109298679.5 | 0.03 |
| DALYs | Chad | 2021 | 6.79 | 12.86 | 3.11 | 0.24 | 8325851 | 5105255047 | 30918137 | 4162925 | 26755211.9 | 0.01 |
| DALYs | Bangladesh | 1992 | 6.52 | 12.19 | 3.14 | 0.25 | 59911290 | 3376967464 | 169695106 | 29955650 | 139739459.7 | 0.04 |
| DALYs | Senegal | 1992 | 8.94 | 16.25 | 4.36 | 0.25 | 3982520 | 3376967464 | 173677626 | 1991260 | 171686366 | 0.05 |
| DALYs | Gambia | 1992 | 8.14 | 15.49 | 3.67 | 0.25 | 540094.8 | 3376967464 | 174217721 | 270047.4 | 173947673.4 | 0.05 |
| DALYs | United Republic of Tanzania | 1992 | 10.92 | 19.08 | 5.55 | 0.26 | 13815680 | 3376967464 | 188033398 | 6907839 | 181125559.4 | 0.05 |
| DALYs | Mali | 2021 | 6.32 | 12.27 | 2.72 | 0.27 | 11895330 | 5105255047 | 42813466 | 5947664 | 36865801.5 | 0.01 |
| DALYs | Lao People's Democratic Republic | 1992 | 1.45 | 3.61 | 0.50 | 0.27 | 2290031 | 3376967464 | 190323429 | 1145016 | 189178413.7 | 0.06 |
| DALYs | Rwanda | 1992 | 19.91 | 39.77 | 8.25 | 0.28 | 3779329 | 3376967464 | 194102758 | 1889664 | 192213093.7 | 0.06 |
| DALYs | South Sudan | 2021 | 9.42 | 17.47 | 4.18 | 0.28 | 5135653 | 5105255047 | 47949119 | 2567827 | 45381292.2 | 0.01 |
| DALYs | Togo | 1992 | 8.62 | 15.39 | 4.21 | 0.28 | 1939975 | 3376967464 | 196042733 | 969987.5 | 195072745.7 | 0.06 |
| DALYs | Angola | 1992 | 12.02 | 21.96 | 5.89 | 0.28 | 5702682 | 3376967464 | 201745415 | 2851341 | 198894074.2 | 0.06 |

| | | | | | | | | | | | | |
|---|---|---|---|---|---|---|---|---|---|---|---|---|
| DALYs | Timor-Leste | 1992 | 0.86 | 2.02 | 0.26 | 0.28 | 447494.9 | 3376967464 | 202192910 | 223747.4 | 201969162.6 | 0.06 |
| DALYs | Madagascar | 1992 | 21.61 | 38.67 | 11.10 | 0.28 | 6432226 | 3376967464 | 208625136 | 3216113 | 205409023.2 | 0.06 |
| DALYs | Equatorial Guinea | 1992 | 14.13 | 25.72 | 6.81 | 0.28 | 229117.5 | 3376967464 | 208854254 | 114558.8 | 208739695 | 0.06 |
| DALYs | Burkina Faso | 2021 | 7.47 | 13.70 | 3.42 | 0.29 | 11698660 | 5105255047 | 59647781 | 5849331 | 53798449.8 | 0.01 |
| DALYs | Cabo Verde | 1992 | 9.54 | 19.13 | 4.36 | 0.29 | 183931.3 | 3376967464 | 209038185 | 91965.67 | 208946219.4 | 0.06 |
| DALYs | Comoros | 1992 | 13.98 | 24.70 | 6.49 | 0.29 | 249116.1 | 3376967464 | 209287301 | 124558 | 209162743.1 | 0.06 |
| DALYs | Burundi | 2021 | 9.28 | 16.50 | 4.60 | 0.29 | 7040681 | 5105255047 | 66688462 | 3520341 | 63168121.4 | 0.01 |
| DALYs | Democratic Republic of the Congo | 1992 | 10.88 | 18.77 | 5.40 | 0.29 | 20457730 | 3376967464 | 229745032 | 10228870 | 219516166.6 | 0.07 |
| DALYs | Coted'Ivoire | 1992 | 8.93 | 16.51 | 4.24 | 0.29 | 6800901 | 3376967464 | 236545933 | 3400450 | 233145482.4 | 0.07 |
| DALYs | Cambodia | 1992 | 0.93 | 2.48 | 0.33 | 0.30 | 5566488 | 3376967464 | 242112421 | 2783244 | 239329176.7 | 0.07 |
| DALYs | Sudan | 1992 | 13.69 | 29.57 | 3.28 | 0.30 | 11202790 | 3376967464 | 253315211 | 5601395 | 247713815.6 | 0.07 |
| DALYs | Zambia | 1992 | 11.71 | 21.91 | 5.60 | 0.31 | 4197849 | 3376967464 | 257513060 | 2098925 | 255414135.1 | 0.08 |
| DALYs | Central African Republic | 2021 | 13.72 | 25.85 | 6.35 | 0.31 | 3075108 | 5105255047 | 69763570 | 1537554 | 68226015.8 | 0.01 |
| DALYs | Nigeria | 1992 | 8.35 | 12.92 | 4.72 | 0.31 | 50478270 | 3376967464 | 307991334 | 25239140 | 282752197 | 0.08 |
| DALYs | Solomon Islands | 1992 | 11.78 | 23.26 | 5.26 | 0.31 | 187733.3 | 3376967464 | 308179068 | 93866.66 | 308085200.9 | 0.09 |
| DALYs | Sao Tome and Principe | 1992 | 7.50 | 14.54 | 3.44 | 0.31 | 61668.72 | 3376967464 | 308240736 | 30834.36 | 308209902 | 0.09 |
| DALYs | Cameroon | 1992 | 9.81 | 17.40 | 4.89 | 0.32 | 5631620 | 3376967464 | 313872356 | 2815810 | 311056546.2 | 0.09 |
| DALYs | Guatemala | 1992 | 7.97 | 10.23 | 5.86 | 0.32 | 4330417 | 3376967464 | 318202773 | 2165209 | 316037564.7 | 0.09 |
| DALYs | Papua New Guinea | 1992 | 19.07 | 39.56 | 7.42 | 0.32 | 2419850 | 3376967464 | 320622623 | 1209925 | 319412698.1 | 0.09 |
| DALYs | Haiti | 1992 | 19.89 | 36.71 | 8.20 | 0.32 | 3615033 | 3376967464 | 324237656 | 1807516 | 322430139.3 | 0.10 |
| DALYs | Pakistan | 1992 | 7.09 | 11.82 | 3.73 | 0.32 | 60357000 | 3376967464 | 384594651 | 30178500 | 354416153.3 | 0.10 |
| DALYs | Myanmar | 1992 | 1.73 | 3.95 | 0.61 | 0.33 | 24926270 | 3376967464 | 409520920 | 12463130 | 397057785.4 | 0.12 |
| DALYs | Mozambique | 2021 | 16.10 | 28.72 | 7.80 | 0.33 | 16039550 | 5105255047 | 85803118 | 8019774 | 77783343.8 | 0.02 |

| | | | | | | | | | | | | |
|---|---|---|---|---|---|---|---|---|---|---|---|---|
| DALYs | Guinea | 2021 | 8.21 | 15.45 | 3.83 | 0.34 | 6958615 | 5105255047 | 92761733 | 3479307 | 89282425.3 | 0.02 |
| DALYs | Afghanistan | 2021 | 12.72 | 28.50 | 3.25 | 0.34 | 16398400 | 5105255047 | 109160135 | 8199201 | 100960934 | 0.02 |
| DALYs | Djibouti | 1992 | 9.93 | 17.89 | 4.96 | 0.34 | 281064.1 | 3376967464 | 409801984 | 140532.1 | 409661452 | 0.12 |
| DALYs | Honduras | 1992 | 11.75 | 18.90 | 6.72 | 0.34 | 2511178 | 3376967464 | 412313162 | 1255589 | 411057573.1 | 0.12 |
| DALYs | India | 1992 | 7.80 | 11.75 | 4.91 | 0.34 | 515839400 | 3376967464 | 928152513 | 257919700 | 670232837.4 | 0.20 |
| DALYs | Kenya | 1992 | 6.63 | 9.85 | 4.36 | 0.35 | 12235470 | 3376967464 | 940387986 | 6117737 | 934270249.4 | 0.28 |
| DALYs | Mauritania | 1992 | 9.85 | 17.70 | 5.19 | 0.35 | 1105278 | 3376967464 | 941493264 | 552639.1 | 940940625.2 | 0.28 |
| DALYs | Lesotho | 1992 | 6.74 | 12.13 | 3.17 | 0.35 | 821010.8 | 3376967464 | 942314275 | 410505.4 | 941903769.7 | 0.28 |
| DALYs | Liberia | 2021 | 8.92 | 16.52 | 4.16 | 0.35 | 3135229 | 5105255047 | 112295364 | 1567614 | 110727749.6 | 0.02 |
| DALYs | Guinea-Bissau | 2021 | 12.00 | 21.86 | 5.90 | 0.35 | 1119372 | 5105255047 | 113414736 | 559686.2 | 112855050.1 | 0.02 |
| DALYs | Nicaragua | 1992 | 4.44 | 6.13 | 3.07 | 0.36 | 2103741 | 3376967464 | 944418017 | 1051871 | 943366145.8 | 0.28 |
| DALYs | Maldives | 1992 | 4.49 | 8.42 | 1.84 | 0.36 | 118284.3 | 3376967464 | 944536301 | 59142.14 | 944477158.7 | 0.28 |
| DALYs | Sierra Leone | 2021 | 8.48 | 16.92 | 3.84 | 0.36 | 5014942 | 5105255047 | 118429679 | 2507471 | 115922207.5 | 0.02 |
| DALYs | Ethiopia | 2021 | 6.15 | 9.37 | 3.47 | 0.36 | 61363610 | 5105255047 | 179793292 | 30681810 | 149111485.3 | 0.03 |
| DALYs | Vanuatu | 1992 | 22.72 | 44.02 | 10.48 | 0.36 | 84103.98 | 3376967464 | 944620405 | 42051.99 | 944578352.8 | 0.28 |
| DALYs | Morocco | 1992 | 13.81 | 27.09 | 3.56 | 0.37 | 15160090 | 3376967464 | 959780492 | 7580044 | 952200448.5 | 0.28 |
| DALYs | Benin | 2021 | 6.10 | 10.93 | 2.90 | 0.37 | 7048559 | 5105255047 | 186841851 | 3524280 | 183317571.5 | 0.04 |
| DALYs | El Salvador | 1992 | 5.09 | 7.08 | 3.55 | 0.38 | 3000660 | 3376967464 | 962781152 | 1500330 | 961280822.3 | 0.28 |
| DALYs | Democratic Republic of the Congo | 2021 | 11.03 | 18.96 | 5.70 | 0.38 | 49675100 | 5105255047 | 236516952 | 24837550 | 211679401.7 | 0.04 |
| DALYs | Malawi | 2021 | 11.91 | 20.40 | 6.17 | 0.38 | 10785330 | 5105255047 | 247302286 | 5392667 | 241909619.1 | 0.05 |
| DALYs | Ghana | 1992 | 12.67 | 21.39 | 6.85 | 0.38 | 8290370 | 3376967464 | 971071522 | 4145185 | 966926337.2 | 0.29 |
| DALYs | Madagascar | 2021 | 21.47 | 36.07 | 11.22 | 0.40 | 16136660 | 5105255047 | 263438948 | 8068331 | 255370616.9 | 0.05 |
| DALYs | Eritrea | 2021 | 11.91 | 21.17 | 6.01 | 0.40 | 3903951 | 5105255047 | 267342899 | 1951975 | 265390923.2 | 0.05 |

| | | | | | | | | | | | |
|---|---|---|---|---|---|---|---|---|---|---|---|
| DALYs | Senegal | 2021 | 8.75 | 15.81 | 4.19 | 0.41 | 8916317 | 5105255047 | 276259216 | 4458159 | 271801057.2 | 0.05 |
| DALYs | Togo | 2021 | 10.23 | 17.88 | 5.14 | 0.41 | 4808399 | 5105255047 | 281067615 | 2404199 | 278663415.3 | 0.05 |
| DALYs | Gambia | 2021 | 10.00 | 17.72 | 4.89 | 0.41 | 1328594 | 5105255047 | 282396209 | 664296.9 | 281731911.6 | 0.06 |
| DALYs | Palestine | 1992 | 7.21 | 12.03 | 4.04 | 0.41 | 1092733 | 3376967464 | 972164255 | 546366.4 | 971617888.3 | 0.29 |
| DALYs | Zimbabwe | 1992 | 7.05 | 14.40 | 3.17 | 0.41 | 5556213 | 3376967464 | 977720467 | 2778106 | 974942361.1 | 0.29 |
| DALYs | Kiribati | 1992 | 59.39 | 96.66 | 32.71 | 0.42 | 43348.47 | 3376967464 | 977763816 | 21674.24 | 977742141.7 | 0.29 |
| DALYs | Eswatini | 1992 | 9.83 | 18.27 | 4.41 | 0.42 | 429553.2 | 3376967464 | 978193369 | 214776.6 | 977978592.6 | 0.29 |
| DALYs | Papua New Guinea | 2021 | 19.99 | 38.26 | 9.14 | 0.42 | 6230283 | 5105255047 | 288626492 | 3115142 | 285511350.2 | 0.06 |
| DALYs | Viet Nam | 1992 | 0.95 | 2.05 | 0.36 | 0.42 | 40268370 | 3376967464 | 1018461738 | 20134180 | 998327553.6 | 0.30 |
| DALYs | Iraq | 1992 | 19.30 | 32.59 | 9.56 | 0.42 | 10043140 | 3376967464 | 1028504876 | 5021569 | 1023483307 | 0.30 |
| DALYs | Tuvalu | 1992 | 32.46 | 59.27 | 16.01 | 0.42 | 5474.875 | 3376967464 | 1028510350 | 2737.437 | 1028507613 | 0.30 |
| DALYs | Uganda | 2021 | 8.53 | 14.92 | 4.25 | 0.42 | 22465620 | 5105255047 | 311092115 | 11232810 | 299859303.5 | 0.06 |
| DALYs | Coted'Ivoire | 2021 | 9.61 | 17.85 | 4.56 | 0.43 | 15562160 | 5105255047 | 326654276 | 7781080 | 318873195.4 | 0.06 |
| DALYs | Solomon Islands | 2021 | 13.67 | 24.98 | 6.78 | 0.43 | 400945.3 | 5105255047 | 327055221 | 200472.7 | 326854748.6 | 0.06 |
| DALYs | Congo | 1992 | 16.97 | 30.10 | 8.49 | 0.43 | 1365818 | 3376967464 | 1029876169 | 682909 | 1029193260 | 0.30 |
| DALYs | Nepal | 2021 | 8.62 | 14.24 | 4.74 | 0.43 | 19987970 | 5105255047 | 347043194 | 9993986 | 337049207.6 | 0.07 |
| DALYs | Bolivia (Plurinational State of) | 1992 | 17.34 | 27.74 | 9.59 | 0.43 | 3645533 | 3376967464 | 1033521701 | 1822766 | 1031698935 | 0.31 |
| DALYs | Rwanda | 2021 | 10.02 | 17.64 | 5.04 | 0.44 | 7848418 | 5105255047 | 354891612 | 3924209 | 350967402.9 | 0.07 |
| DALYs | Egypt | 1992 | 20.13 | 33.32 | 10.64 | 0.44 | 33112790 | 3376967464 | 1066634488 | 16556390 | 1050078095 | 0.31 |
| DALYs | Botswana | 1992 | 11.25 | 20.72 | 5.36 | 0.44 | 747651.5 | 3376967464 | 1067382140 | 373825.7 | 1067008314 | 0.32 |
| DALYs | Marshall Islands | 1992 | 27.61 | 51.91 | 12.12 | 0.44 | 23628.24 | 3376967464 | 1067405768 | 11814.12 | 1067393954 | 0.32 |
| DALYs | Belize | 1992 | 4.47 | 5.95 | 3.49 | 0.44 | 103326.3 | 3376967464 | 1067509094 | 51663.13 | 1067457431 | 0.32 |
| DALYs | Timor-Leste | 2021 | 1.11 | 2.71 | 0.36 | 0.44 | 802737.4 | 5105255047 | 355694349 | 401368.7 | 355292980.6 | 0.07 |
| DALYs | Syrian Arab Republic | 1992 | 45.69 | 75.52 | 24.38 | 0.45 | 6931728 | 3376967464 | 1074440822 | 3465864 | 1070974958 | 0.32 |

| | | | | | | | | | | | | | |
|---|---|---|---|---|---|---|---|---|---|---|---|---|---|
| DALYs | United Republic of Tanzania | 2021 | 11.12 | 20.54 | 5.64 | 0.45 | 32205890 | 5105255047 | 387900240 | 16102950 | 371797294.8 | 0.07 |
| DALYs | Haiti | 2021 | 20.60 | 36.78 | 9.47 | 0.45 | 8005224 | 5105255047 | 395905464 | 4002612 | 391902852.4 | 0.08 |
| DALYs | Dominican Republic | 1992 | 13.29 | 20.13 | 8.31 | 0.45 | 4367623 | 3376967464 | 1078808445 | 2183812 | 1076624634 | 0.32 |
| DALYs | Yemen | 2021 | 11.17 | 23.03 | 2.69 | 0.45 | 18839690 | 5105255047 | 414745156 | 9419846 | 405325310.2 | 0.08 |
| DALYs | Angola | 2021 | 12.41 | 21.27 | 6.56 | 0.45 | 16719890 | 5105255047 | 431465050 | 8359947 | 423105102.9 | 0.08 |
| DALYs | Grenada | 1992 | 16.31 | 22.21 | 11.45 | 0.46 | 48427.92 | 3376967464 | 1078856873 | 24213.96 | 1078832659 | 0.32 |
| DALYs | Oman | 1992 | 5.23 | 9.79 | 2.36 | 0.46 | 1153630 | 3376967464 | 1080010503 | 576815.1 | 1079433688 | 0.32 |
| DALYs | Namibia | 1992 | 9.52 | 16.37 | 5.05 | 0.46 | 804903.9 | 3376967464 | 1080815407 | 402451.9 | 1080412955 | 0.32 |
| DALYs | Guyana | 1992 | 27.42 | 35.29 | 20.71 | 0.47 | 459165.3 | 3376967464 | 1081274573 | 229582.7 | 1081044990 | 0.32 |
| DALYs | Gabon | 1992 | 15.68 | 26.36 | 8.67 | 0.47 | 557425.5 | 3376967464 | 1081831998 | 278712.8 | 1081553285 | 0.32 |
| DALYs | Bhutan | 2021 | 8.14 | 13.77 | 4.40 | 0.47 | 519639.6 | 5105255047 | 431984689 | 259819.8 | 431724869.5 | 0.08 |
| DALYs | Vanuatu | 2021 | 22.94 | 39.78 | 11.84 | 0.47 | 184213.1 | 5105255047 | 432168902 | 92106.55 | 432076795.9 | 0.08 |
| DALYs | Cambodia | 2021 | 1.03 | 2.56 | 0.39 | 0.47 | 10996940 | 5105255047 | 443165844 | 5498471 | 437667373 | 0.09 |
| DALYs | Micronesia (Federated States of) | 1992 | 30.44 | 53.96 | 15.14 | 0.47 | 55679.86 | 3376967464 | 1081887678 | 27839.93 | 1081859838 | 0.32 |
| DALYs | Zimbabwe | 2021 | 15.24 | 28.27 | 7.42 | 0.47 | 8807361 | 5105255047 | 451973205 | 4403681 | 447569524.4 | 0.09 |
| DALYs | Tajikistan | 1992 | 0.32 | 0.56 | 0.16 | 0.48 | 2943035 | 3376967464 | 1084830713 | 1471517 | 1083359195 | 0.32 |
| DALYs | Indonesia | 1992 | 1.22 | 3.01 | 0.48 | 0.48 | 115716600 | 3376967464 | 1200547263 | 57858280 | 1142688988 | 0.34 |
| DALYs | China | 1992 | 1.69 | 2.53 | 1.05 | 0.48 | 815239200 | 3376967464 | 2015786424 | 407619600 | 1608166844 | 0.48 |
| DALYs | Algeria | 1992 | 12.73 | 24.66 | 3.19 | 0.48 | 14528470 | 3376967464 | 2030314898 | 7264237 | 2023050661 | 0.60 |
| DALYs | Comoros | 2021 | 11.46 | 20.38 | 5.76 | 0.48 | 467233.1 | 5105255047 | 452440438 | 233616.6 | 452206821.7 | 0.09 |
| DALYs | Turkey | 1992 | 22.96 | 39.37 | 11.06 | 0.48 | 36334360 | 3376967464 | 2066649254 | 18167180 | 2048482076 | 0.61 |
| DALYs | Mongolia | 1992 | 1.74 | 3.33 | 0.89 | 0.48 | 1235917 | 3376967464 | 2067885171 | 617958.5 | 2067267212 | 0.61 |
| DALYs | Paraguay | 1992 | 22.60 | 33.23 | 14.92 | 0.48 | 2324234 | 3376967464 | 2070209405 | 1162117 | 2069047288 | 0.61 |
| DALYs | Cameroon | 2021 | 10.04 | 17.47 | 5.00 | 0.48 | 17450670 | 5105255047 | 469891104 | 8725333 | 461165771 | 0.09 |

| | | | | | | | | | | | | |
|---|---|---|---|---|---|---|---|---|---|---|---|---|
| DALYs | Iran (Islamic Republic of) | 1992 | 13.23 | 18.73 | 9.07 | 0.48 | 32234900 | 3376967464 | 2102444303 | 16117450 | 2086326854 | 0.62 |
| DALYs | Colombia | 1992 | 27.49 | 31.91 | 23.55 | 0.48 | 20427380 | 3376967464 | 2122871685 | 10213690 | 2112657994 | 0.63 |
| DALYs | Saint Vincent and the Grenadines | 1992 | 23.74 | 29.16 | 19.25 | 0.49 | 63082.62 | 3376967464 | 2122934768 | 31541.31 | 2122903226 | 0.63 |
| DALYs | Djibouti | 2021 | 10.85 | 19.89 | 5.23 | 0.49 | 805751.6 | 5105255047 | 470696855 | 402875.8 | 470293979.5 | 0.09 |
| DALYs | Tunisia | 1992 | 12.43 | 23.28 | 3.27 | 0.49 | 5133459 | 3376967464 | 2128068226 | 2566729 | 2125501497 | 0.63 |
| DALYs | Lao People's Democratic Republic | 2021 | 1.33 | 3.36 | 0.47 | 0.49 | 4754006 | 5105255047 | 475450861 | 2377003 | 473073858.3 | 0.09 |
| DALYs | Bangladesh | 2021 | 8.85 | 15.06 | 4.79 | 0.49 | 107272700 | 5105255047 | 582723583 | 53636360 | 529087222.1 | 0.10 |
| DALYs | Samoa | 1992 | 21.27 | 36.65 | 11.61 | 0.49 | 92361.26 | 3376967464 | 2128160588 | 46180.63 | 2128114407 | 0.63 |
| DALYs | Mauritania | 2021 | 8.08 | 14.37 | 4.21 | 0.50 | 2374531 | 5105255047 | 585098113 | 1187265 | 583910848.2 | 0.11 |
| DALYs | Democratic People's Republic of Korea | 1992 | 2.45 | 4.86 | 1.02 | 0.50 | 13875620 | 3376967464 | 2142036206 | 6937809 | 2135098397 | 0.63 |
| DALYs | Uzbekistan | 1992 | 1.73 | 2.40 | 1.20 | 0.50 | 12096580 | 3376967464 | 2154132781 | 6048288 | 2148084494 | 0.64 |
| DALYs | Nigeria | 2021 | 6.95 | 12.91 | 3.62 | 0.50 | 123377300 | 5105255047 | 708475425 | 61688660 | 646786769.3 | 0.13 |
| DALYs | Tonga | 1992 | 13.61 | 23.10 | 7.78 | 0.50 | 53374.01 | 3376967464 | 2154186155 | 26687 | 2154159468 | 0.64 |
| DALYs | Pakistan | 2021 | 10.11 | 15.86 | 6.15 | 0.50 | 141565600 | 5105255047 | 850041000 | 70782790 | 779258212.8 | 0.15 |
| DALYs | Sao Tome and Principe | 2021 | 9.82 | 19.93 | 4.65 | 0.51 | 131024.1 | 5105255047 | 850172025 | 65512.04 | 850106512.5 | 0.17 |
| DALYs | Zambia | 2021 | 12.43 | 21.88 | 6.53 | 0.51 | 10789070 | 5105255047 | 860961092 | 5394534 | 855566558.4 | 0.17 |
| DALYs | Brazil | 1992 | 32.13 | 34.06 | 30.25 | 0.51 | 93825900 | 3376967464 | 2248012056 | 46912950 | 2201099106 | 0.65 |
| DALYs | Lesotho | 2021 | 12.96 | 23.15 | 6.35 | 0.51 | 1159988 | 5105255047 | 862121080 | 579994.1 | 861541086.2 | 0.17 |
| DALYs | Suriname | 1992 | 15.34 | 23.59 | 9.12 | 0.51 | 240092.4 | 3376967464 | 2248252148 | 120046.2 | 2248132102 | 0.67 |
| DALYs | Honduras | 2021 | 13.33 | 20.83 | 7.76 | 0.51 | 6325127 | 5105255047 | 868446208 | 3162564 | 865283644 | 0.17 |
| DALYs | Peru | 1992 | 10.46 | 15.92 | 6.48 | 0.51 | 13017280 | 3376967464 | 2261269426 | 6508639 | 2254760787 | 0.67 |
| DALYs | Mexico | 1992 | 12.57 | 13.16 | 12.00 | 0.51 | 51527500 | 3376967464 | 2312796928 | 25763750 | 2287033177 | 0.68 |

| | | | | | | | | | | | | |
|---|---|---|---|---|---|---|---|---|---|---|---|---|
| DALYs | Saint Lucia | 1992 | 19.76 | 23.65 | 16.28 | 0.52 | 80360.32 | 3376967464 | 2312877288 | 40180.16 | 2312837108 | 0.68 |
| DALYs | Philippines | 1992 | 1.33 | 1.80 | 1.03 | 0.52 | 37716100 | 3376967464 | 2350593387 | 18858050 | 2331735338 | 0.69 |
| DALYs | Ecuador | 1992 | 12.10 | 14.29 | 10.06 | 0.52 | 6008273 | 3376967464 | 2356601661 | 3004137 | 2353597524 | 0.70 |
| DALYs | Kenya | 2021 | 8.04 | 11.28 | 5.67 | 0.52 | 29742370 | 5105255047 | 898188583 | 14871190 | 883317395.1 | 0.17 |
| DALYs | Nicaragua | 2021 | 5.69 | 8.05 | 3.88 | 0.52 | 4297839 | 5105255047 | 902486421 | 2148919 | 900337502 | 0.18 |
| DALYs | Thailand | 1992 | 3.31 | 5.47 | 1.68 | 0.53 | 39057400 | 3376967464 | 2395659056 | 19528700 | 2376130359 | 0.70 |
| DALYs | Kiribati | 2021 | 65.76 | 111.30 | 35.22 | 0.53 | 74513.75 | 5105255047 | 902560935 | 37256.87 | 902523678.2 | 0.18 |
| DALYs | Kyrgyzstan | 1992 | 1.02 | 1.40 | 0.76 | 0.53 | 2635720 | 3376967464 | 2398294776 | 1317860 | 2396976916 | 0.71 |
| DALYs | Venezuela (Bolivarian Republic of) | 1992 | 16.45 | 18.90 | 14.22 | 0.53 | 11607490 | 3376967464 | 2409902266 | 5803745 | 2404098521 | 0.71 |
| DALYs | Tokelau | 1992 | 28.23 | 52.04 | 14.44 | 0.53 | 834.3087 | 3376967464 | 2409903100 | 417.1543 | 2409902683 | 0.71 |
| DALYs | Sri Lanka | 1992 | 8.14 | 11.58 | 5.37 | 0.53 | 11121210 | 3376967464 | 2421024309 | 5560605 | 2415463705 | 0.72 |
| DALYs | Cabo Verde | 2021 | 10.00 | 19.24 | 5.03 | 0.53 | 382357.4 | 5105255047 | 902943292 | 191178.7 | 902752113.8 | 0.18 |
| DALYs | Myanmar | 2021 | 1.68 | 3.95 | 0.65 | 0.53 | 36996570 | 5105255047 | 939939864 | 18498290 | 921441578.5 | 0.18 |
| DALYs | Nauru | 1992 | 44.82 | 80.75 | 21.51 | 0.54 | 5770.117 | 3376967464 | 2421030079 | 2885.058 | 2421027194 | 0.72 |
| DALYs | Bosnia and Herzegovina | 1992 | 12.39 | 21.49 | 6.15 | 0.54 | 3112775 | 3376967464 | 2424142855 | 1556388 | 2422586467 | 0.72 |
| DALYs | Guatemala | 2021 | 7.40 | 9.19 | 5.88 | 0.54 | 9913414 | 5105255047 | 949853279 | 4956707 | 944896571.5 | 0.19 |
| DALYs | Tajikistan | 2021 | 0.35 | 0.67 | 0.17 | 0.54 | 6207893 | 5105255047 | 956061172 | 3103947 | 952957225.2 | 0.19 |
| DALYs | Lebanon | 1992 | 58.85 | 100.30 | 27.63 | 0.54 | 1853583 | 3376967464 | 2425996438 | 926791.5 | 2425069646 | 0.72 |
| DALYs | Sudan | 2021 | 13.19 | 26.17 | 3.67 | 0.54 | 25440510 | 5105255047 | 981501682 | 12720260 | 968781427 | 0.19 |
| DALYs | Fiji | 1992 | 7.17 | 11.44 | 4.17 | 0.54 | 465040.9 | 3376967464 | 2426461479 | 232520.5 | 2426228958 | 0.72 |
| DALYs | Costa Rica | 1992 | 17.93 | 20.78 | 15.46 | 0.54 | 1893843 | 3376967464 | 2428355322 | 946921.6 | 2427408400 | 0.72 |
| DALYs | Jamaica | 1992 | 2.31 | 3.08 | 1.75 | 0.55 | 1395283 | 3376967464 | 2429750605 | 697641.5 | 2429052964 | 0.72 |
| DALYs | Jordan | 1992 | 12.24 | 18.06 | 7.66 | 0.55 | 2205439 | 3376967464 | 2431956044 | 1102720 | 2430853325 | 0.72 |

| | | | | | | | | | | | | | |
|---|---|---|---|---|---|---|---|---|---|---|---|---|---|
| DALYs | Armenia | 1992 | 3.05 | 3.86 | 2.33 | 0.55 | 2180135 | 3376967464 | 2434136179 | 1090067 | 2433046112 | 0.72 |
| DALYs | South Africa | 1992 | 16.91 | 24.03 | 12.09 | 0.55 | 22998760 | 3376967464 | 2457134939 | 11499380 | 2445635559 | 0.72 |
| DALYs | Albania | 1992 | 4.65 | 6.74 | 2.92 | 0.55 | 2048832 | 3376967464 | 2459183771 | 1024416 | 2458159355 | 0.73 |
| DALYs | Mauritius | 1992 | 20.64 | 24.05 | 17.52 | 0.55 | 729511 | 3376967464 | 2459913282 | 364755.5 | 2459548527 | 0.73 |
| DALYs | Libya | 1992 | 12.64 | 24.54 | 3.28 | 0.55 | 2388892 | 3376967464 | 2462302174 | 1194446 | 2461107728 | 0.73 |
| DALYs | Panama | 1992 | 9.66 | 11.32 | 8.14 | 0.55 | 1507409 | 3376967464 | 2463809583 | 753704.7 | 2463055878 | 0.73 |
| DALYs | Malaysia | 1992 | 5.66 | 8.32 | 3.62 | 0.56 | 11151630 | 3376967464 | 2474961212 | 5575814 | 2469385397 | 0.73 |
| DALYs | Saudi Arabia | 1992 | 8.58 | 14.95 | 4.45 | 0.56 | 9752944 | 3376967464 | 2484714156 | 4876472 | 2479837684 | 0.73 |
| DALYs | Morocco | 2021 | 14.30 | 27.61 | 4.45 | 0.56 | 24647580 | 5105255047 | 1006149263 | 12323790 | 993825472.4 | 0.19 |
| DALYs | El Salvador | 2021 | 6.17 | 8.89 | 4.09 | 0.56 | 4074428 | 5105255047 | 1010223691 | 2037214 | 1008186477 | 0.20 |
| DALYs | Ghana | 2021 | 10.74 | 18.53 | 5.70 | 0.56 | 20161140 | 5105255047 | 1030384835 | 10080570 | 1020304263 | 0.20 |
| DALYs | Turkmenistan | 1992 | 0.53 | 0.80 | 0.35 | 0.57 | 2146841 | 3376967464 | 2486860997 | 1073420 | 2485787576 | 0.74 |
| DALYs | Cuba | 1992 | 19.70 | 23.31 | 16.39 | 0.57 | 7554381 | 3376967464 | 2494415377 | 3777190 | 2490638187 | 0.74 |
| DALYs | Dominica | 1992 | 11.99 | 18.92 | 7.35 | 0.57 | 41551.19 | 3376967464 | 2494456928 | 20775.6 | 2494436153 | 0.74 |
| DALYs | Democratic People's Republic of Korea | 2021 | 2.33 | 4.55 | 1.12 | 0.57 | 18946650 | 5105255047 | 1049331482 | 9473323 | 1039858159 | 0.20 |
| DALYs | Marshall Islands | 2021 | 31.31 | 58.05 | 14.90 | 0.57 | 36532.79 | 5105255047 | 1049368015 | 18266.39 | 1049349748 | 0.21 |
| DALYs | India | 2021 | 9.69 | 12.46 | 7.13 | 0.58 | 950604900 | 5105255047 | 1999972931 | 475302500 | 1524670473 | 0.30 |
| DALYs | Tuvalu | 2021 | 27.07 | 45.74 | 15.42 | 0.58 | 7770.842 | 5105255047 | 1999980701 | 3885.421 | 1999976816 | 0.39 |
| DALYs | Cook Islands | 1992 | 15.47 | 28.65 | 8.29 | 0.58 | 11553.59 | 3376967464 | 2494468482 | 5776.796 | 2494462705 | 0.74 |
| DALYs | Congo | 2021 | 15.70 | 26.79 | 8.46 | 0.58 | 3289800 | 5105255047 | 2003270502 | 1644900 | 2001625602 | 0.39 |
| DALYs | Eswatini | 2021 | 13.73 | 25.10 | 6.29 | 0.59 | 702572.3 | 5105255047 | 2003973074 | 351286.1 | 2003621788 | 0.39 |
| DALYs | Micronesia (Federated States of) | 2021 | 29.74 | 50.36 | 16.13 | 0.59 | 67172.81 | 5105255047 | 2004040247 | 33586.4 | 2004006661 | 0.39 |
| DALYs | Uruguay | 1992 | 43.59 | 49.80 | 37.67 | 0.59 | 1978724 | 3376967464 | 2496447206 | 989362.1 | 2495457844 | 0.74 |

| | | | | | | | | | | | | |
|---|---|---|---|---|---|---|---|---|---|---|---|---|
| DALYs | Seychelles | 1992 | 7.62 | 12.04 | 3.19 | 0.59 | 45508.48 | 3376967464 | 2496492715 | 22754.24 | 2496469961 | 0.74 |
| DALYs | Samoa | 2021 | 22.49 | 38.01 | 12.47 | 0.59 | 122652 | 5105255047 | 2004162899 | 61325.99 | 2004101573 | 0.39 |
| DALYs | Azerbaijan | 1992 | 1.75 | 2.75 | 1.07 | 0.59 | 4664389 | 3376967464 | 2501157104 | 2332195 | 2498824909 | 0.74 |
| DALYs | Bahrain | 1992 | 13.03 | 19.31 | 8.56 | 0.60 | 353670.8 | 3376967464 | 2501510775 | 176835.4 | 2501333939 | 0.74 |
| DALYs | Kazakhstan | 1992 | 2.95 | 3.71 | 2.42 | 0.60 | 10145610 | 3376967464 | 2511656388 | 5072806 | 2506583581 | 0.74 |
| DALYs | Venezuela (Bolivarian Republic of) | 2021 | 23.57 | 32.10 | 16.85 | 0.60 | 17426450 | 5105255047 | 2021589354 | 8713227 | 2012876126 | 0.39 |
| DALYs | Argentina | 1992 | 43.56 | 50.07 | 37.82 | 0.60 | 20617890 | 3376967464 | 2532274273 | 10308940 | 2521965330 | 0.75 |
| DALYs | Bolivia (Plurinational State of) | 2021 | 15.60 | 26.15 | 8.96 | 0.60 | 7560756 | 5105255047 | 2029150109 | 3780378 | 2025369731 | 0.40 |
| DALYs | Chile | 1992 | 15.09 | 17.79 | 12.78 | 0.60 | 8721318 | 3376967464 | 2540995591 | 4360659 | 2536634932 | 0.75 |
| DALYs | Saint Kitts and Nevis | 1992 | 15.83 | 19.21 | 12.29 | 0.60 | 23875.01 | 3376967464 | 2541019466 | 11937.5 | 2541007529 | 0.75 |
| DALYs | Niue | 1992 | 27.61 | 47.55 | 15.03 | 0.60 | 1295.984 | 3376967464 | 2541020762 | 647.9919 | 2541020114 | 0.75 |
| DALYs | Kyrgyzstan | 2021 | 2.28 | 3.00 | 1.71 | 0.60 | 4248862 | 5105255047 | 2033398972 | 2124431 | 2031274540 | 0.40 |
| DALYs | Egypt | 2021 | 20.39 | 31.17 | 12.57 | 0.61 | 64397140 | 5105255047 | 2097796116 | 32198570 | 2065597544 | 0.40 |
| DALYs | Republic of Moldova | 1992 | 1.44 | 2.08 | 1.01 | 0.61 | 2854706 | 3376967464 | 2543875468 | 1427353 | 2542448115 | 0.75 |
| DALYs | Belize | 2021 | 5.13 | 6.51 | 3.97 | 0.61 | 283479.5 | 5105255047 | 2098079595 | 141739.7 | 2097937856 | 0.41 |
| DALYs | North Macedonia | 1992 | 4.58 | 7.23 | 3.07 | 0.61 | 1323446 | 3376967464 | 2545198915 | 661723.2 | 2544537192 | 0.75 |
| DALYs | Portugal | 1992 | 20.76 | 24.15 | 17.83 | 0.61 | 6780922 | 3376967464 | 2551979837 | 3390461 | 2548589376 | 0.75 |
| DALYs | Namibia | 2021 | 10.36 | 18.76 | 5.14 | 0.62 | 1504481 | 5105255047 | 2099584076 | 752240.5 | 2098831836 | 0.41 |
| DALYs | Mongolia | 2021 | 5.07 | 7.65 | 3.17 | 0.62 | 2105152 | 5105255047 | 2101689228 | 1052576 | 2100636652 | 0.41 |
| DALYs | American Samoa | 1992 | 27.42 | 45.13 | 15.42 | 0.62 | 29749.7 | 3376967464 | 2552009587 | 14874.85 | 2551994712 | 0.76 |
| DALYs | Dominican Republic | 2021 | 15.72 | 24.37 | 9.64 | 0.62 | 7231298 | 5105255047 | 2108920526 | 3615649 | 2105304877 | 0.41 |
| DALYs | Syrian Arab Republic | 2021 | 37.92 | 61.97 | 20.45 | 0.62 | 9352995 | 5105255047 | 2118273521 | 4676498 | 2113597023 | 0.41 |
| DALYs | Antigua and Barbuda | 1992 | 10.85 | 13.27 | 8.67 | 0.62 | 38493.21 | 3376967464 | 2552048080 | 19246.61 | 2552028833 | 0.76 |

| | | | | | | | | | | | | |
|---|---|---|---|---|---|---|---|---|---|---|---|---|
| DALYs | Nauru | 2021 | 43.06 | 76.41 | 22.23 | 0.63 | 6647.105 | 5105255047 | 2118280168 | 3323.553 | 2118276845 | 0.41 |
| DALYs | Tonga | 2021 | 14.61 | 26.10 | 7.96 | 0.63 | 60598.31 | 5105255047 | 2118340767 | 30299.16 | 2118310467 | 0.41 |
| DALYs | Viet Nam | 2021 | 1.37 | 2.88 | 0.59 | 0.63 | 67836850 | 5105255047 | 2186177612 | 33918420 | 2152259189 | 0.42 |
| DALYs | Romania | 1992 | 10.58 | 13.06 | 8.58 | 0.63 | 15484770 | 3376967464 | 2567532852 | 7742386 | 2559790466 | 0.76 |
| DALYs | Palestine | 2021 | 5.65 | 8.70 | 3.55 | 0.63 | 3092703 | 5105255047 | 2189270315 | 1546351 | 2187723963 | 0.43 |
| DALYs | Belarus | 1992 | 1.63 | 2.38 | 1.16 | 0.63 | 6938289 | 3376967464 | 2574471141 | 3469144 | 2571001997 | 0.76 |
| DALYs | Suriname | 2021 | 16.55 | 26.97 | 9.36 | 0.63 | 384154.9 | 5105255047 | 2189654470 | 192077.4 | 2189462392 | 0.43 |
| DALYs | Trinidad and Tobago | 1992 | 9.74 | 11.68 | 8.08 | 0.63 | 745716.6 | 3376967464 | 2575216858 | 372858.3 | 2574843999 | 0.76 |
| DALYs | Gabon | 2021 | 14.78 | 25.23 | 8.02 | 0.63 | 1102224 | 5105255047 | 2190756693 | 551111.8 | 2190205581 | 0.43 |
| DALYs | Paraguay | 2021 | 30.78 | 45.25 | 20.03 | 0.64 | 4680073 | 5105255047 | 2195436766 | 2340037 | 2193096730 | 0.43 |
| DALYs | Saint Vincent and the Grenadines | 2021 | 23.36 | 28.67 | 18.72 | 0.64 | 76552.59 | 5105255047 | 2195513319 | 38276.3 | 2195475043 | 0.43 |
| DALYs | Poland | 1992 | 8.09 | 8.60 | 7.66 | 0.64 | 24967360 | 3376967464 | 2600184220 | 12483680 | 2587700539 | 0.77 |
| DALYs | Serbia | 1992 | 21.37 | 32.81 | 12.92 | 0.64 | 6574516 | 3376967464 | 2606758736 | 3287258 | 2603471478 | 0.77 |
| DALYs | Botswana | 2021 | 8.10 | 14.40 | 4.05 | 0.64 | 1590402 | 5105255047 | 2197103721 | 795200.9 | 2196308520 | 0.43 |
| DALYs | Guyana | 2021 | 41.84 | 56.97 | 29.75 | 0.65 | 501391.8 | 5105255047 | 2197605113 | 250695.9 | 2197354417 | 0.43 |
| DALYs | Maldives | 2021 | 4.43 | 7.04 | 2.64 | 0.65 | 394937.9 | 5105255047 | 2198000050 | 197468.9 | 2197802582 | 0.43 |
| DALYs | Bulgaria | 1992 | 3.87 | 4.96 | 3.11 | 0.65 | 5699135 | 3376967464 | 2612457871 | 2849568 | 2609608303 | 0.77 |
| DALYs | Philippines | 2021 | 2.21 | 3.09 | 1.70 | 0.65 | 73079830 | 5105255047 | 2271079884 | 36539920 | 2234539967 | 0.44 |
| DALYs | Spain | 1992 | 46.49 | 53.57 | 40.33 | 0.65 | 26113210 | 3376967464 | 2638571077 | 13056600 | 2625514474 | 0.78 |
| DALYs | Brazil | 2021 | 22.90 | 24.38 | 21.55 | 0.65 | 150405800 | 5105255047 | 2421485716 | 75202920 | 2346282800 | 0.46 |
| DALYs | Colombia | 2021 | 24.05 | 30.17 | 18.86 | 0.66 | 33609590 | 5105255047 | 2455095302 | 16804790 | 2438290509 | 0.48 |
| DALYs | Indonesia | 2021 | 1.49 | 3.35 | 0.54 | 0.66 | 194150400 | 5105255047 | 2649245676 | 97075190 | 2552170489 | 0.50 |
| DALYs | Ukraine | 1992 | 2.40 | 3.25 | 1.84 | 0.66 | 34804310 | 3376967464 | 2673375387 | 17402160 | 2655973232 | 0.79 |
| DALYs | Equatorial Guinea | 2021 | 10.13 | 19.35 | 4.65 | 0.66 | 893663 | 5105255047 | 2650139339 | 446831.5 | 2649692507 | 0.52 |

| | | | | | | | | | | | | |
|---|---|---|---|---|---|---|---|---|---|---|---|---|
| DALYs | Algeria | 2021 | 11.92 | 22.45 | 3.37 | 0.66 | 28054400 | 5105255047 | 2678193741 | 14027200 | 2664166540 | 0.52 |
| DALYs | Hungary | 1992 | 52.48 | 60.19 | 45.91 | 0.66 | 6927328 | 3376967464 | 2680302715 | 3463664 | 2676839051 | 0.79 |
| DALYs | Qatar | 1992 | 15.97 | 25.13 | 9.44 | 0.66 | 335643.3 | 3376967464 | 2680638358 | 167821.6 | 2680470537 | 0.79 |
| DALYs | Ecuador | 2021 | 15.20 | 20.04 | 11.20 | 0.66 | 11574520 | 5105255047 | 2689768262 | 5787261 | 2683981001 | 0.53 |
| DALYs | Peru | 2021 | 10.22 | 16.08 | 6.26 | 0.66 | 23885430 | 5105255047 | 2713653696 | 11942720 | 2701710979 | 0.53 |
| DALYs | Barbados | 1992 | 19.98 | 23.70 | 16.64 | 0.66 | 162757.9 | 3376967464 | 2680801116 | 81378.93 | 2680719737 | 0.79 |
| DALYs | Uzbekistan | 2021 | 4.80 | 6.01 | 3.77 | 0.66 | 22329360 | 5105255047 | 2735983057 | 11164680 | 2724818377 | 0.53 |
| DALYs | Iraq | 2021 | 16.60 | 28.82 | 7.87 | 0.66 | 26081420 | 5105255047 | 2762064476 | 13040710 | 2749023766 | 0.54 |
| DALYs | Mexico | 2021 | 16.11 | 18.35 | 14.07 | 0.66 | 86646750 | 5105255047 | 2848711229 | 43323380 | 2805387853 | 0.55 |
| DALYs | Georgia | 1992 | 2.16 | 2.97 | 1.52 | 0.67 | 3570243 | 3376967464 | 2684371360 | 1785122 | 2682586238 | 0.79 |
| DALYs | Slovakia | 1992 | 10.17 | 14.82 | 6.88 | 0.67 | 3469779 | 3376967464 | 2687841139 | 1734890 | 2686106249 | 0.80 |
| DALYs | Cyprus | 1992 | 40.75 | 61.90 | 26.19 | 0.67 | 522214.5 | 3376967464 | 2688363353 | 261107.2 | 2688102246 | 0.80 |
| DALYs | Malta | 1992 | 12.15 | 14.17 | 10.37 | 0.67 | 249227.5 | 3376967464 | 2688612581 | 124613.8 | 2688487967 | 0.80 |
| DALYs | Guam | 1992 | 50.54 | 71.60 | 34.78 | 0.67 | 93009.67 | 3376967464 | 2688705591 | 46504.84 | 2688659086 | 0.80 |
| DALYs | Puerto Rico | 1992 | 32.19 | 37.79 | 27.41 | 0.67 | 2339319 | 3376967464 | 2691044910 | 1169660 | 2689875250 | 0.80 |
| DALYs | Cuba | 2021 | 26.18 | 31.91 | 21.05 | 0.67 | 7721598 | 5105255047 | 2856432827 | 3860799 | 2852572028 | 0.56 |
| DALYs | Grenada | 2021 | 29.64 | 37.86 | 22.86 | 0.67 | 71465.1 | 5105255047 | 2856504292 | 35732.55 | 2856468560 | 0.56 |
| DALYs | Kuwait | 1992 | 12.31 | 14.57 | 10.42 | 0.67 | 1142046 | 3376967464 | 2692186956 | 571023.1 | 2691615933 | 0.80 |
| DALYs | United States Virgin Islands | 1992 | 32.77 | 50.48 | 20.01 | 0.67 | 68428.68 | 3376967464 | 2692255385 | 34214.34 | 2692221171 | 0.80 |
| DALYs | Saint Lucia | 2021 | 17.36 | 22.20 | 13.27 | 0.67 | 127254.6 | 5105255047 | 2856631547 | 63627.3 | 2856567920 | 0.56 |
| DALYs | Montenegro | 1992 | 7.26 | 10.43 | 4.89 | 0.67 | 417608.2 | 3376967464 | 2692672993 | 208804.1 | 2692464189 | 0.80 |
| DALYs | Palau | 1992 | 24.18 | 41.88 | 11.81 | 0.67 | 10631.73 | 3376967464 | 2692683625 | 5315.866 | 2692678309 | 0.80 |
| DALYs | Fiji | 2021 | 7.17 | 11.65 | 4.13 | 0.68 | 595530.4 | 5105255047 | 2857227077 | 297765.2 | 2856929312 | 0.56 |
| DALYs | United Arab Emirates | 1992 | 37.29 | 59.79 | 21.64 | 0.68 | 1447269 | 3376967464 | 2694130893 | 723634.3 | 2693407259 | 0.80 |

| | | | | | | | | | | | | |
|---|---|---|---|---|---|---|---|---|---|---|---|---|
| DALYs | Brunei Darussalam | 1992 | 11.91 | 18.42 | 7.24 | 0.68 | 172733 | 3376967464 | 2694303626 | 86366.52 | 2694217260 | 0.80 |
| DALYs | Croatia | 1992 | 13.67 | 15.91 | 11.73 | 0.68 | 3329713 | 3376967464 | 2697633339 | 1664857 | 2695968483 | 0.80 |
| DALYs | South Africa | 2021 | 13.73 | 18.71 | 10.32 | 0.68 | 37983600 | 5105255047 | 2895210682 | 18991800 | 2876218879 | 0.56 |
| DALYs | Lithuania | 1992 | 8.56 | 10.33 | 7.18 | 0.68 | 2447071 | 3376967464 | 2700080410 | 1223536 | 2698856875 | 0.80 |
| DALYs | Turkmenistan | 2021 | 3.14 | 4.21 | 2.27 | 0.68 | 3350351 | 5105255047 | 2898561033 | 1675176 | 2896885857 | 0.57 |
| DALYs | Tunisia | 2021 | 13.06 | 25.64 | 3.90 | 0.68 | 7946659 | 5105255047 | 2906507692 | 3973329 | 2902534362 | 0.57 |
| DALYs | Thailand | 2021 | 8.56 | 13.70 | 5.08 | 0.68 | 47274310 | 5105255047 | 2953782004 | 23637160 | 2930144848 | 0.57 |
| DALYs | Jamaica | 2021 | 4.37 | 6.03 | 3.09 | 0.68 | 1946551 | 5105255047 | 2955728554 | 973275.3 | 2954755279 | 0.58 |
| DALYs | Taiwan (Province of China) | 1992 | 11.35 | 13.21 | 9.70 | 0.68 | 14026120 | 3376967464 | 2714106526 | 7013058 | 2707093468 | 0.80 |
| DALYs | Tokelau | 2021 | 31.97 | 55.21 | 18.35 | 0.69 | 843.5528 | 5105255047 | 2955729398 | 421.7764 | 2955728976 | 0.58 |
| DALYs | Greece | 1992 | 20.48 | 23.47 | 17.81 | 0.69 | 7122789 | 3376967464 | 2721229315 | 3561394 | 2717667921 | 0.80 |
| DALYs | Bahamas | 1992 | 13.34 | 16.01 | 10.88 | 0.69 | 171499.1 | 3376967464 | 2721400814 | 85749.56 | 2721315065 | 0.81 |
| DALYs | Russian Federation | 1992 | 5.15 | 5.83 | 4.72 | 0.69 | 101681600 | 3376967464 | 2823082430 | 50840810 | 2772241622 | 0.82 |
| DALYs | Azerbaijan | 2021 | 1.54 | 2.41 | 0.97 | 0.69 | 7440503 | 5105255047 | 2963169901 | 3720252 | 2959449649 | 0.58 |
| DALYs | Estonia | 1992 | 23.85 | 28.91 | 20.04 | 0.70 | 1018565 | 3376967464 | 2824100995 | 509282.4 | 2823591712 | 0.84 |
| DALYs | Latvia | 1992 | 14.46 | 16.84 | 12.47 | 0.70 | 1746906 | 3376967464 | 2825847901 | 873453 | 2824974448 | 0.84 |
| DALYs | Iran (Islamic Republic of) | 2021 | 11.97 | 16.94 | 9.36 | 0.70 | 59167520 | 5105255047 | 3022337423 | 29583760 | 2992753662 | 0.59 |
| DALYs | Czechia | 1992 | 8.46 | 9.86 | 7.27 | 0.70 | 6884471 | 3376967464 | 2832732372 | 3442236 | 2829290136 | 0.84 |
| DALYs | Costa Rica | 2021 | 28.99 | 34.89 | 23.64 | 0.70 | 3249833 | 5105255047 | 3025587256 | 1624917 | 3023962339 | 0.59 |
| DALYs | Sri Lanka | 2021 | 5.99 | 9.79 | 3.34 | 0.70 | 14714740 | 5105255047 | 3040301997 | 7357370 | 3032944626 | 0.59 |
| DALYs | Armenia | 2021 | 4.98 | 6.02 | 4.01 | 0.70 | 2004496 | 5105255047 | 3042306493 | 1002248 | 3041304245 | 0.60 |
| DALYs | Singapore | 1992 | 8.39 | 9.86 | 7.12 | 0.70 | 2369939 | 3376967464 | 2835102310 | 1184969 | 2833917341 | 0.84 |
| DALYs | Bermuda | 1992 | 110.39 | 130.70 | 90.65 | 0.70 | 42685.4 | 3376967464 | 2835144996 | 21342.7 | 2835123653 | 0.84 |
| DALYs | Albania | 2021 | 9.46 | 14.25 | 6.13 | 0.71 | 1808384 | 5105255047 | 3044114877 | 904192 | 3043210685 | 0.60 |

| | | | | | | | | | | | | |
|---|---|---|---|---|---|---|---|---|---|---|---|---|
| DALYs | Panama | 2021 | 15.59 | 19.92 | 11.62 | 0.71 | 2749507 | 5105255047 | 3046864384 | 1374753 | 3045489630 | 0.60 |
| DALYs | Republic of Korea | 1992 | 6.52 | 9.73 | 4.01 | 0.71 | 31435440 | 3376967464 | 2866580440 | 15717720 | 2850862718 | 0.84 |
| DALYs | Turkey | 2021 | 17.34 | 26.72 | 10.51 | 0.71 | 56921960 | 5105255047 | 3103786340 | 28460980 | 3075325362 | 0.60 |
| DALYs | Northern Mariana Islands | 1992 | 41.42 | 65.50 | 24.39 | 0.72 | 33889.48 | 3376967464 | 2866614329 | 16944.74 | 2866597384 | 0.85 |
| DALYs | Italy | 1992 | 25.11 | 26.71 | 23.67 | 0.72 | 38983200 | 3376967464 | 2905597533 | 19491600 | 2886105931 | 0.85 |
| DALYs | Israel | 1992 | 18.46 | 21.52 | 15.63 | 0.72 | 3155772 | 3376967464 | 2908753305 | 1577886 | 2907175419 | 0.86 |
| DALYs | Mauritius | 2021 | 27.10 | 31.77 | 22.63 | 0.72 | 900058.5 | 5105255047 | 3104686398 | 450029.2 | 3104236369 | 0.61 |
| DALYs | Uruguay | 2021 | 45.86 | 53.23 | 39.25 | 0.72 | 2214747 | 5105255047 | 3106901145 | 1107373 | 3105793772 | 0.61 |
| DALYs | China | 2021 | 1.49 | 2.00 | 1.11 | 0.72 | 967106700 | 5105255047 | 4074007884 | 483553400 | 3590454514 | 0.70 |
| DALYs | Bosnia and Herzegovina | 2021 | 15.95 | 25.45 | 9.04 | 0.72 | 2206873 | 5105255047 | 4076214757 | 1103437 | 4075111320 | 0.80 |
| DALYs | Argentina | 2021 | 25.96 | 30.07 | 22.25 | 0.72 | 30052210 | 5105255047 | 4106266964 | 15026100 | 4091240861 | 0.80 |
| DALYs | American Samoa | 2021 | 29.97 | 46.80 | 17.47 | 0.72 | 31908.17 | 5105255047 | 4106298872 | 15954.09 | 4106282918 | 0.80 |
| DALYs | Kazakhstan | 2021 | 3.96 | 5.08 | 3.06 | 0.73 | 12126520 | 5105255047 | 4118425393 | 6063260 | 4112362132 | 0.81 |
| DALYs | Jordan | 2021 | 7.69 | 11.47 | 5.03 | 0.73 | 8180007 | 5105255047 | 4126605399 | 4090003 | 4122515396 | 0.81 |
| DALYs | Libya | 2021 | 20.73 | 45.33 | 4.82 | 0.73 | 5029432 | 5105255047 | 4131634831 | 2514716 | 4129120115 | 0.81 |
| DALYs | Niue | 2021 | 29.39 | 50.99 | 16.98 | 0.73 | 1099.18 | 5105255047 | 4131635930 | 549.5901 | 4131635381 | 0.81 |
| DALYs | Greenland | 1992 | 53.28 | 85.86 | 25.98 | 0.73 | 38395.59 | 3376967464 | 2908791700 | 19197.79 | 2908772503 | 0.86 |
| DALYs | Seychelles | 2021 | 6.85 | 10.75 | 2.84 | 0.73 | 72957.22 | 5105255047 | 4131708887 | 36478.61 | 4131672409 | 0.81 |
| DALYs | Ireland | 1992 | 19.90 | 23.25 | 16.98 | 0.73 | 2251651 | 3376967464 | 2911043351 | 1125825 | 2909917526 | 0.86 |
| DALYs | Republic of Moldova | 2021 | 5.32 | 6.92 | 4.12 | 0.73 | 2516382 | 5105255047 | 4134225269 | 1258191 | 4132967078 | 0.81 |
| DALYs | Georgia | 2021 | 19.21 | 23.74 | 15.35 | 0.73 | 2299321 | 5105255047 | 4136524590 | 1149660 | 4135374929 | 0.81 |
| DALYs | Australia | 1992 | 21.18 | 24.42 | 18.36 | 0.73 | 11484530 | 3376967464 | 2922527885 | 5742267 | 2916785618 | 0.86 |
| DALYs | Slovenia | 1992 | 39.60 | 45.25 | 34.51 | 0.74 | 1357830 | 3376967464 | 2923885715 | 678914.9 | 2923206800 | 0.87 |
| DALYs | Malaysia | 2021 | 5.88 | 8.71 | 3.80 | 0.74 | 21856660 | 5105255047 | 4158381246 | 10928330 | 4147452918 | 0.81 |

| | | | | | | | | | | | | | |
|---|---|---|---|---|---|---|---|---|---|---|---|---|---|
| DALYs | France | 1992 | 40.05 | 46.52 | 34.31 | 0.74 | 38242170 | 3376967464 | 2962127885 | 19121090 | 2943006800 | 0.87 |
| DALYs | Portugal | 2021 | 24.56 | 28.17 | 21.26 | 0.74 | 6825510 | 5105255047 | 4165206755 | 3412755 | 4161794000 | 0.82 |
| DALYs | Lebanon | 2021 | 27.67 | 41.04 | 17.89 | 0.74 | 3716347 | 5105255047 | 4168923102 | 1858174 | 4167064929 | 0.82 |
| DALYs | Barbados | 2021 | 24.88 | 33.26 | 18.44 | 0.75 | 202783.5 | 5105255047 | 4169125886 | 101391.7 | 4169024494 | 0.82 |
| DALYs | Dominica | 2021 | 19.27 | 30.94 | 11.42 | 0.75 | 46130.66 | 5105255047 | 4169172016 | 23065.33 | 4169148951 | 0.82 |
| DALYs | Belgium | 1992 | 34.82 | 40.34 | 29.80 | 0.75 | 6685776 | 3376967464 | 2968813661 | 3342888 | 2965470773 | 0.88 |
| DALYs | Antigua and Barbuda | 2021 | 10.58 | 12.79 | 8.69 | 0.75 | 63578.22 | 5105255047 | 4169235595 | 31789.11 | 4169203806 | 0.82 |
| DALYs | North Macedonia | 2021 | 9.46 | 13.57 | 6.40 | 0.75 | 1541196 | 5105255047 | 4170776791 | 770598.2 | 4170006193 | 0.82 |
| DALYs | Bahrain | 2021 | 12.58 | 18.51 | 8.45 | 0.75 | 1178377 | 5105255047 | 4171955168 | 589188.4 | 4171365980 | 0.82 |
| DALYs | Palau | 2021 | 24.31 | 41.12 | 13.76 | 0.75 | 13185.18 | 5105255047 | 4171968353 | 6592.589 | 4171961761 | 0.82 |
| DALYs | Austria | 1992 | 28.91 | 33.57 | 24.75 | 0.75 | 5270737 | 3376967464 | 2974084397 | 2635368 | 2971449029 | 0.88 |
| DALYs | United Kingdom | 1992 | 26.23 | 27.17 | 25.41 | 0.75 | 37397090 | 3376967464 | 3011481485 | 18698540 | 2992782941 | 0.89 |
| DALYs | Saint Kitts and Nevis | 2021 | 15.11 | 19.99 | 11.28 | 0.75 | 43420.68 | 5105255047 | 4172011774 | 21710.34 | 4171990064 | 0.82 |
| DALYs | New Zealand | 1992 | 26.84 | 30.46 | 23.58 | 0.76 | 2285123 | 3376967464 | 3013766608 | 1142561 | 3012624047 | 0.89 |
| DALYs | Ukraine | 2021 | 9.87 | 13.60 | 6.81 | 0.76 | 29287980 | 5105255047 | 4201299752 | 14643990 | 4186655763 | 0.82 |
| DALYs | Finland | 1992 | 33.41 | 39.02 | 28.39 | 0.76 | 3388185 | 3376967464 | 3017154793 | 1694093 | 3015460701 | 0.89 |
| DALYs | Andorra | 1992 | 14.93 | 23.87 | 8.67 | 0.77 | 45683.08 | 3376967464 | 3017200476 | 22841.54 | 3017177635 | 0.89 |
| DALYs | Bulgaria | 2021 | 16.22 | 19.85 | 13.17 | 0.77 | 4337506 | 5105255047 | 4205637258 | 2168753 | 4203468505 | 0.82 |
| DALYs | Romania | 2021 | 17.57 | 21.91 | 13.94 | 0.77 | 12142540 | 5105255047 | 4217779801 | 6071271 | 4211708529 | 0.82 |
| DALYs | Trinidad and Tobago | 2021 | 14.74 | 19.97 | 10.64 | 0.77 | 942563.1 | 5105255047 | 4218722364 | 471281.5 | 4218251082 | 0.83 |
| DALYs | Spain | 2021 | 24.00 | 27.59 | 20.75 | 0.77 | 29873650 | 5105255047 | 4248596015 | 14936830 | 4233659190 | 0.83 |
| DALYs | United States of America | 1992 | 32.94 | 34.31 | 31.71 | 0.77 | 169626400 | 3376967464 | 3186826882 | 84813200 | 3102013679 | 0.92 |
| DALYs | Chile | 2021 | 13.25 | 15.66 | 11.12 | 0.77 | 12819950 | 5105255047 | 4261415966 | 6409975 | 4255005991 | 0.83 |
| DALYs | Northern Mariana Islands | 2021 | 46.23 | 69.32 | 29.49 | 0.77 | 33644 | 5105255047 | 4261449610 | 16822 | 4261432788 | 0.83 |

| | | | | | | | | | | | | |
|---|---|---|---|---|---|---|---|---|---|---|---|---|
| DALYs | Iceland | 1992 | 21.54 | 25.17 | 18.32 | 0.77 | 167194.9 | 3376967464 | 3186994077 | 83597.46 | 3186910480 | 0.94 |
| DALYs | Oman | 2021 | 3.85 | 6.40 | 2.13 | 0.77 | 3365550 | 5105255047 | 4264815160 | 1682775 | 4263132385 | 0.84 |
| DALYs | Cook Islands | 2021 | 10.49 | 20.82 | 5.40 | 0.78 | 11633.24 | 5105255047 | 4264826793 | 5816.619 | 4264820977 | 0.84 |
| DALYs | Belarus | 2021 | 5.15 | 6.81 | 3.93 | 0.78 | 6254502 | 5105255047 | 4271081295 | 3127251 | 4267954044 | 0.84 |
| DALYs | Canada | 1992 | 19.46 | 22.42 | 16.70 | 0.79 | 18901940 | 3376967464 | 3205896013 | 9450968 | 3196445045 | 0.95 |
| DALYs | Luxembourg | 1992 | 38.88 | 44.81 | 33.59 | 0.79 | 268190.4 | 3376967464 | 3206164203 | 134095.2 | 3206030108 | 0.95 |
| DALYs | Hungary | 2021 | 65.54 | 75.63 | 56.58 | 0.79 | 6199148 | 5105255047 | 4277280443 | 3099574 | 4274180869 | 0.84 |
| DALYs | Greece | 2021 | 25.19 | 28.87 | 21.83 | 0.79 | 6469340 | 5105255047 | 4283749783 | 3234670 | 4280515113 | 0.84 |
| DALYs | Serbia | 2021 | 26.54 | 38.25 | 17.63 | 0.79 | 5934126 | 5105255047 | 4289683909 | 2967063 | 4286716846 | 0.84 |
| DALYs | Sweden | 1992 | 24.94 | 28.30 | 21.84 | 0.80 | 5553552 | 3376967464 | 3211717756 | 2776776 | 3208940980 | 0.95 |
| DALYs | Montenegro | 2021 | 8.90 | 12.92 | 6.01 | 0.80 | 412912.2 | 5105255047 | 4290096822 | 206456.1 | 4289890365 | 0.84 |
| DALYs | Croatia | 2021 | 35.76 | 43.11 | 29.36 | 0.80 | 2715472 | 5105255047 | 4292812293 | 1357736 | 4291454558 | 0.84 |
| DALYs | Japan | 1992 | 30.37 | 31.74 | 29.09 | 0.80 | 88370150 | 3376967464 | 3300087906 | 44185070 | 3255902831 | 0.96 |
| DALYs | Malta | 2021 | 17.72 | 20.94 | 14.85 | 0.80 | 278137.3 | 5105255047 | 4293090431 | 139068.6 | 4292951362 | 0.84 |
| DALYs | Netherlands | 1992 | 27.83 | 32.45 | 23.72 | 0.80 | 10396300 | 3376967464 | 3310484202 | 5198148 | 3305286054 | 0.98 |
| DALYs | Guam | 2021 | 39.90 | 55.38 | 28.24 | 0.80 | 103477.4 | 5105255047 | 4293193908 | 51738.72 | 4293142170 | 0.84 |
| DALYs | Bahamas | 2021 | 17.62 | 22.99 | 13.31 | 0.81 | 274938.4 | 5105255047 | 4293468847 | 137469.2 | 4293331377 | 0.84 |
| DALYs | Norway | 1992 | 24.30 | 25.75 | 22.92 | 0.81 | 2768970 | 3376967464 | 3313253172 | 1384485 | 3311868687 | 0.98 |
| DALYs | Italy | 2021 | 21.08 | 22.78 | 19.50 | 0.81 | 38186670 | 5105255047 | 4331655515 | 19093330 | 4312562181 | 0.84 |
| DALYs | Denmark | 1992 | 25.59 | 29.88 | 21.82 | 0.81 | 3484450 | 3376967464 | 3316737622 | 1742225 | 3314995397 | 0.98 |
| DALYs | Russian Federation | 2021 | 21.14 | 23.41 | 19.08 | 0.81 | 96028800 | 5105255047 | 4427684315 | 48014400 | 4379669915 | 0.86 |
| DALYs | Israel | 2021 | 16.04 | 18.75 | 13.50 | 0.81 | 5769267 | 5105255047 | 4433453582 | 2884633 | 4430568949 | 0.87 |
| DALYs | Brunei Darussalam | 2021 | 15.16 | 22.23 | 9.99 | 0.81 | 332019.1 | 5105255047 | 4433785601 | 166009.6 | 4433619592 | 0.87 |
| DALYs | Slovakia | 2021 | 18.80 | 26.23 | 12.98 | 0.81 | 3635982 | 5105255047 | 4437421583 | 1817991 | 4435603592 | 0.87 |

| | | | | | | | | | | | | | |
|---|---|---|---|---|---|---|---|---|---|---|---|---|---|
| DALYs | Poland | 2021 | 36.92 | 40.76 | 33.39 | 0.81 | 25179790 | 5105255047 | 4462601375 | 12589900 | 4450011479 | 0.87 |
| DALYs | Saudi Arabia | 2021 | 12.32 | 21.09 | 6.75 | 0.82 | 29117100 | 5105255047 | 4491718473 | 14558550 | 4477159924 | 0.88 |
| DALYs | Bermuda | 2021 | 87.58 | 110.32 | 68.95 | 0.82 | 41969.77 | 5105255047 | 4491760443 | 20984.88 | 4491739458 | 0.88 |
| DALYs | United States Virgin Islands | 2021 | 29.25 | 49.35 | 16.39 | 0.82 | 53866.94 | 5105255047 | 4491814310 | 26933.47 | 4491787376 | 0.88 |
| DALYs | San Marino | 1992 | 8.49 | 12.35 | 5.60 | 0.82 | 16789.73 | 3376967464 | 3316754411 | 8394.865 | 3316746017 | 0.98 |
| DALYs | Puerto Rico | 2021 | 37.27 | 46.85 | 28.99 | 0.83 | 2124836 | 5105255047 | 4493939146 | 1062418 | 4492876728 | 0.88 |
| DALYs | Greenland | 2021 | 40.97 | 61.96 | 24.33 | 0.83 | 39064.81 | 5105255047 | 4493978210 | 19532.41 | 4493958678 | 0.88 |
| DALYs | Germany | 1992 | 44.12 | 51.63 | 37.64 | 0.83 | 55437780 | 3376967464 | 3372192189 | 27718890 | 3344473300 | 0.99 |
| DALYs | Czechia | 2021 | 30.49 | 36.30 | 25.10 | 0.83 | 6708178 | 5105255047 | 4500686388 | 3354089 | 4497332299 | 0.88 |
| DALYs | Latvia | 2021 | 40.99 | 48.89 | 34.00 | 0.83 | 1180840 | 5105255047 | 4501867228 | 590420 | 4501276808 | 0.88 |
| DALYs | Cyprus | 2021 | 29.20 | 42.47 | 19.79 | 0.84 | 940775.7 | 5105255047 | 4502808004 | 470387.8 | 4502337616 | 0.88 |
| DALYs | France | 2021 | 20.36 | 23.97 | 17.19 | 0.84 | 41021790 | 5105255047 | 4543829797 | 20510900 | 4523318900 | 0.89 |
| DALYs | Slovenia | 2021 | 67.53 | 81.11 | 54.55 | 0.84 | 1320405 | 5105255047 | 4545150201 | 660202.5 | 4544489999 | 0.89 |
| DALYs | Australia | 2021 | 15.00 | 17.46 | 12.79 | 0.84 | 16656050 | 5105255047 | 4561806249 | 8328024 | 4553478225 | 0.89 |
| DALYs | Estonia | 2021 | 54.33 | 65.31 | 45.11 | 0.84 | 825205.3 | 5105255047 | 4562631454 | 412602.6 | 4562218851 | 0.89 |
| DALYs | Kuwait | 2021 | 10.25 | 12.78 | 8.16 | 0.85 | 3633714 | 5105255047 | 4566265168 | 1816857 | 4564448311 | 0.89 |
| DALYs | Qatar | 2021 | 7.91 | 13.17 | 4.40 | 0.85 | 2446107 | 5105255047 | 4568711276 | 1223054 | 4567488222 | 0.89 |
| DALYs | United Arab Emirates | 2021 | 21.62 | 32.80 | 13.55 | 0.85 | 8129433 | 5105255047 | 4576840709 | 4064717 | 4572775992 | 0.90 |
| DALYs | New Zealand | 2021 | 23.71 | 26.76 | 20.96 | 0.85 | 3378026 | 5105255047 | 4580218735 | 1689013 | 4578529722 | 0.90 |
| DALYs | Monaco | 1992 | 12.37 | 18.98 | 7.49 | 0.85 | 19942.64 | 3376967464 | 3372212132 | 9971.318 | 3372202160 | 1.00 |
| DALYs | Belgium | 2021 | 27.57 | 31.88 | 23.75 | 0.85 | 7314275 | 5105255047 | 4587533010 | 3657137 | 4583875872 | 0.90 |
| DALYs | Austria | 2021 | 20.89 | 24.52 | 17.75 | 0.85 | 5970418 | 5105255047 | 4593503427 | 2985209 | 4590518219 | 0.90 |
| DALYs | Singapore | 2021 | 4.13 | 4.93 | 3.48 | 0.86 | 4146887 | 5105255047 | 4597650315 | 2073444 | 4595576871 | 0.90 |
| DALYs | Lithuania | 2021 | 31.07 | 36.84 | 25.60 | 0.86 | 1763517 | 5105255047 | 4599413832 | 881758.6 | 4598532074 | 0.90 |

| | | | | | | | | | | | | |
|---|---|---|---|---|---|---|---|---|---|---|---|---|
| DALYs | United Kingdom | 2021 | 25.77 | 26.89 | 24.71 | 0.86 | 43558640 | 5105255047 | 4642972474 | 21779320 | 4621193153 | 0.91 |
| DALYs | Finland | 2021 | 30.06 | 35.18 | 25.44 | 0.86 | 3397926 | 5105255047 | 4646370400 | 1698963 | 4644671437 | 0.91 |
| DALYs | United States of America | 2021 | 23.43 | 24.81 | 22.15 | 0.86 | 216097500 | 5105255047 | 4862467891 | 108048700 | 4754419146 | 0.93 |
| DALYs | Switzerland | 1992 | 20.96 | 24.54 | 17.70 | 0.87 | 4755332 | 3376967464 | 3376967464 | 2377666 | 3374589798 | 1.00 |
| DALYs | Andorra | 2021 | 13.28 | 22.10 | 7.61 | 0.87 | 61742.59 | 5105255047 | 4862529634 | 30871.3 | 4862498763 | 0.95 |
| DALYs | Japan | 2021 | 10.92 | 11.61 | 10.31 | 0.87 | 75404150 | 5105255047 | 4937933788 | 37702080 | 4900231711 | 0.96 |
| DALYs | Canada | 2021 | 16.77 | 19.63 | 14.30 | 0.87 | 24262340 | 5105255047 | 4962196124 | 12131170 | 4950064956 | 0.97 |
| DALYs | Ireland | 2021 | 16.43 | 19.64 | 13.66 | 0.87 | 3192765 | 5105255047 | 4965388890 | 1596383 | 4963792507 | 0.97 |
| DALYs | Taiwan (Province of China) | 2021 | 10.54 | 12.27 | 8.92 | 0.87 | 16681750 | 5105255047 | 4982070642 | 8340876 | 4973729766 | 0.97 |
| DALYs | Iceland | 2021 | 28.27 | 33.63 | 23.26 | 0.88 | 227618.6 | 5105255047 | 4982298260 | 113809.3 | 4982184451 | 0.98 |
| DALYs | Luxembourg | 2021 | 27.87 | 32.74 | 23.57 | 0.88 | 447039.5 | 5105255047 | 4982745300 | 223519.8 | 4982521780 | 0.98 |
| DALYs | Republic of Korea | 2021 | 5.83 | 8.01 | 4.18 | 0.89 | 36999740 | 5105255047 | 5019745039 | 18499870 | 5001245169 | 0.98 |
| DALYs | Sweden | 2021 | 14.25 | 17.12 | 11.75 | 0.89 | 6417083 | 5105255047 | 5026162122 | 3208541 | 5022953580 | 0.98 |
| DALYs | San Marino | 2021 | 7.84 | 12.76 | 4.27 | 0.89 | 21290.2 | 5105255047 | 5026183412 | 10645.1 | 5026172767 | 0.98 |
| DALYs | Netherlands | 2021 | 21.39 | 24.88 | 18.29 | 0.89 | 11069920 | 5105255047 | 5037253336 | 5534962 | 5031718374 | 0.99 |
| DALYs | Denmark | 2021 | 24.49 | 28.57 | 20.69 | 0.90 | 3719018 | 5105255047 | 5040972354 | 1859509 | 5039112845 | 0.99 |
| DALYs | Germany | 2021 | 33.68 | 39.25 | 28.57 | 0.90 | 54851780 | 5105255047 | 5095824131 | 27425890 | 5068398243 | 0.99 |
| DALYs | Monaco | 2021 | 11.43 | 18.40 | 6.76 | 0.91 | 23218.3 | 5105255047 | 5095847350 | 11609.15 | 5095835740 | 1.00 |
| DALYs | Norway | 2021 | 19.13 | 20.83 | 17.54 | 0.92 | 3522494 | 5105255047 | 5099369844 | 1761247 | 5097608597 | 1.00 |
| DALYs | Switzerland | 2021 | 15.34 | 18.04 | 12.93 | 0.93 | 5885204 | 5105255047 | 5105255047 | 2942602 | 5102312446 | 1.00 |
| Deaths | Somalia | 1992 | 0.32 | 0.65 | 0.12 | 0.05 | 3816581 | 3376967464 | 3816581 | 1908290 | 1908290.3 | 0.00 |
| Deaths | Somalia | 2021 | 0.25 | 0.49 | 0.09 | 0.08 | 10890630 | 5105255047 | 10890631 | 5445315 | 5445315.5 | 0.00 |
| Deaths | Niger | 1992 | 0.20 | 0.39 | 0.08 | 0.08 | 4059013 | 3376967464 | 7875594 | 2029507 | 5846087.2 | 0.00 |
| Deaths | Chad | 1992 | 0.22 | 0.41 | 0.10 | 0.12 | 3037794 | 3376967464 | 10913388 | 1518897 | 9394490.9 | 0.00 |

| | | | | | | | | | | | | |
|---|---|---|---|---|---|---|---|---|---|---|---|---|
| Deaths | Mali | 1992 | 0.21 | 0.41 | 0.10 | 0.13 | 4416030 | 3376967464 | 15329418 | 2208015 | 13121403 | 0.00 |
| Deaths | Burkina Faso | 1992 | 0.25 | 0.46 | 0.11 | 0.14 | 4697926 | 3376967464 | 20027344 | 2348963 | 17678381.3 | 0.01 |
| Deaths | Ethiopia | 1992 | 0.28 | 0.49 | 0.14 | 0.15 | 27028040 | 3376967464 | 47055389 | 13514020 | 33541366.7 | 0.01 |
| Deaths | Niger | 2021 | 0.19 | 0.35 | 0.07 | 0.17 | 11701660 | 5105255047 | 22592286 | 5850828 | 16741458.7 | 0.00 |
| Deaths | Mozambique | 1992 | 0.34 | 0.60 | 0.16 | 0.18 | 6841201 | 3376967464 | 53896590 | 3420600 | 50475989.3 | 0.01 |
| Deaths | Afghanistan | 1992 | 0.38 | 0.98 | 0.08 | 0.18 | 5317936 | 3376967464 | 59214526 | 2658968 | 56555557.9 | 0.02 |
| Deaths | Guinea | 1992 | 0.25 | 0.46 | 0.11 | 0.18 | 3194335 | 3376967464 | 62408861 | 1597167 | 60811693.6 | 0.02 |
| Deaths | Uganda | 1992 | 0.32 | 0.60 | 0.14 | 0.19 | 8871780 | 3376967464 | 71280641 | 4435890 | 66844751.1 | 0.02 |
| Deaths | Malawi | 1992 | 0.29 | 0.51 | 0.15 | 0.21 | 5373904 | 3376967464 | 76654545 | 2686952 | 73967593.3 | 0.02 |
| Deaths | Burundi | 1992 | 0.49 | 0.93 | 0.22 | 0.21 | 2868701 | 3376967464 | 79523246 | 1434350 | 78088895.8 | 0.02 |
| Deaths | South Sudan | 1992 | 0.34 | 0.65 | 0.15 | 0.21 | 3170246 | 3376967464 | 82693492 | 1585123 | 81108369 | 0.02 |
| Deaths | Nepal | 1992 | 0.21 | 0.38 | 0.10 | 0.21 | 10843820 | 3376967464 | 93537312 | 5421910 | 88115402.1 | 0.03 |
| Deaths | Sierra Leone | 1992 | 0.27 | 0.50 | 0.12 | 0.21 | 2160724 | 3376967464 | 95698036 | 1080362 | 94617674.1 | 0.03 |
| Deaths | Guinea-Bissau | 1992 | 0.42 | 0.78 | 0.19 | 0.22 | 518730.4 | 3376967464 | 96216766 | 259365.2 | 95957401.1 | 0.03 |
| Deaths | Central African Republic | 1992 | 0.51 | 0.97 | 0.22 | 0.22 | 1511635 | 3376967464 | 97728401 | 755817.3 | 96972583.7 | 0.03 |
| Deaths | Benin | 1992 | 0.22 | 0.39 | 0.11 | 0.23 | 2437709 | 3376967464 | 100166110 | 1218855 | 98947255.7 | 0.03 |
| Deaths | Bhutan | 1992 | 0.21 | 0.41 | 0.09 | 0.23 | 362111 | 3376967464 | 100528221 | 181055.5 | 100347165.9 | 0.03 |
| Deaths | Eritrea | 1992 | 0.43 | 0.81 | 0.18 | 0.23 | 1636011 | 3376967464 | 102164232 | 818005.3 | 101346226.7 | 0.03 |
| Deaths | Yemen | 1992 | 0.36 | 0.82 | 0.09 | 0.23 | 6649314 | 3376967464 | 108813546 | 3324657 | 105488888.8 | 0.03 |
| Deaths | Liberia | 1992 | 0.28 | 0.49 | 0.14 | 0.24 | 970267.7 | 3376967464 | 109783813 | 485133.8 | 109298679.5 | 0.03 |
| Deaths | Chad | 2021 | 0.25 | 0.47 | 0.12 | 0.24 | 8325851 | 5105255047 | 30918137 | 4162925 | 26755211.9 | 0.01 |
| Deaths | Bangladesh | 1992 | 0.25 | 0.45 | 0.12 | 0.25 | 59911290 | 3376967464 | 169695106 | 29955650 | 139739459.7 | 0.04 |
| Deaths | Senegal | 1992 | 0.30 | 0.54 | 0.15 | 0.25 | 3982520 | 3376967464 | 173677626 | 1991260 | 171686366 | 0.05 |
| Deaths | Gambia | 1992 | 0.30 | 0.57 | 0.14 | 0.25 | 540094.8 | 3376967464 | 174217721 | 270047.4 | 173947673.4 | 0.05 |

| | | | | | | | | | | | | |
|---|---|---|---|---|---|---|---|---|---|---|---|---|
| Deaths | United Republic of Tanzania | 1992 | 0.35 | 0.61 | 0.19 | 0.26 | 13815680 | 3376967464 | 188033398 | 6907839 | 181125559.4 | 0.05 |
| Deaths | Mali | 2021 | 0.20 | 0.38 | 0.09 | 0.27 | 11895330 | 5105255047 | 42813466 | 5947664 | 36865801.5 | 0.01 |
| Deaths | Lao People's Democratic Republic | 1992 | 0.04 | 0.11 | 0.02 | 0.27 | 2290031 | 3376967464 | 190323429 | 1145016 | 189178413.7 | 0.06 |
| Deaths | Rwanda | 1992 | 0.61 | 1.17 | 0.27 | 0.28 | 3779329 | 3376967464 | 194102758 | 1889664 | 192213093.7 | 0.06 |
| Deaths | South Sudan | 2021 | 0.29 | 0.53 | 0.13 | 0.28 | 5135653 | 5105255047 | 47949119 | 2567827 | 45381292.2 | 0.01 |
| Deaths | Togo | 1992 | 0.32 | 0.55 | 0.16 | 0.28 | 1939975 | 3376967464 | 196042733 | 969987.5 | 195072745.7 | 0.06 |
| Deaths | Angola | 1992 | 0.45 | 0.80 | 0.23 | 0.28 | 5702682 | 3376967464 | 201745415 | 2851341 | 198894074.2 | 0.06 |
| Deaths | Timor-Leste | 1992 | 0.03 | 0.07 | 0.01 | 0.28 | 447494.9 | 3376967464 | 202192910 | 223747.4 | 201969162.6 | 0.06 |
| Deaths | Madagascar | 1992 | 0.62 | 1.08 | 0.33 | 0.28 | 6432226 | 3376967464 | 208625136 | 3216113 | 205409023.2 | 0.06 |
| Deaths | Equatorial Guinea | 1992 | 0.48 | 0.86 | 0.24 | 0.28 | 229117.5 | 3376967464 | 208854254 | 114558.8 | 208739695 | 0.06 |
| Deaths | Burkina Faso | 2021 | 0.28 | 0.50 | 0.13 | 0.29 | 11698660 | 5105255047 | 59647781 | 5849331 | 53798449.8 | 0.01 |
| Deaths | Cabo Verde | 1992 | 0.27 | 0.55 | 0.12 | 0.29 | 183931.3 | 3376967464 | 209038185 | 91965.67 | 208946219.4 | 0.06 |
| Deaths | Comoros | 1992 | 0.41 | 0.71 | 0.20 | 0.29 | 249116.1 | 3376967464 | 209287301 | 124558 | 209162743.1 | 0.06 |
| Deaths | Burundi | 2021 | 0.29 | 0.51 | 0.15 | 0.29 | 7040681 | 5105255047 | 66688462 | 3520341 | 63168121.4 | 0.01 |
| Deaths | Democratic Republic of the Congo | 1992 | 0.39 | 0.65 | 0.19 | 0.29 | 20457730 | 3376967464 | 229745032 | 10228870 | 219516166.6 | 0.07 |
| Deaths | Coted'Ivoire | 1992 | 0.33 | 0.60 | 0.16 | 0.29 | 6800901 | 3376967464 | 236545933 | 3400450 | 233145482.4 | 0.07 |
| Deaths | Cambodia | 1992 | 0.03 | 0.09 | 0.01 | 0.30 | 5566488 | 3376967464 | 242112421 | 2783244 | 239329176.7 | 0.07 |
| Deaths | Sudan | 1992 | 0.38 | 0.82 | 0.10 | 0.30 | 11202790 | 3376967464 | 253315211 | 5601395 | 247713815.6 | 0.07 |
| Deaths | Zambia | 1992 | 0.38 | 0.68 | 0.19 | 0.31 | 4197849 | 3376967464 | 257513060 | 2098925 | 255414135.1 | 0.08 |
| Deaths | Central African Republic | 2021 | 0.47 | 0.87 | 0.23 | 0.31 | 3075108 | 5105255047 | 69763570 | 1537554 | 68226015.8 | 0.01 |
| Deaths | Nigeria | 1992 | 0.28 | 0.42 | 0.16 | 0.31 | 50478270 | 3376967464 | 307991334 | 25239140 | 282752197 | 0.08 |
| Deaths | Solomon Islands | 1992 | 0.32 | 0.61 | 0.15 | 0.31 | 187733.3 | 3376967464 | 308179068 | 93866.66 | 308085200.9 | 0.09 |

| | | | | | | | | | | | | |
|---|---|---|---|---|---|---|---|---|---|---|---|---|
| Deaths | Sao Tome and Principe | 1992 | 0.24 | 0.44 | 0.12 | 0.31 | 61668.72 | 3376967464 | 308240736 | 30834.36 | 308209902 | 0.09 |
| Deaths | Cameroon | 1992 | 0.33 | 0.58 | 0.17 | 0.32 | 5631620 | 3376967464 | 313872356 | 2815810 | 311056546.2 | 0.09 |
| Deaths | Guatemala | 1992 | 0.24 | 0.31 | 0.18 | 0.32 | 4330417 | 3376967464 | 318202773 | 2165209 | 316037564.7 | 0.09 |
| Deaths | Papua New Guinea | 1992 | 0.42 | 0.83 | 0.19 | 0.32 | 2419850 | 3376967464 | 320622623 | 1209925 | 319412698.1 | 0.09 |
| Deaths | Haiti | 1992 | 0.60 | 1.09 | 0.26 | 0.32 | 3615033 | 3376967464 | 324237656 | 1807516 | 322430139.3 | 0.10 |
| Deaths | Pakistan | 1992 | 0.23 | 0.37 | 0.13 | 0.32 | 60357000 | 3376967464 | 384594651 | 30178500 | 354416153.3 | 0.10 |
| Deaths | Myanmar | 1992 | 0.05 | 0.12 | 0.02 | 0.33 | 24926270 | 3376967464 | 409520920 | 12463130 | 397057785.4 | 0.12 |
| Deaths | Mozambique | 2021 | 0.54 | 0.94 | 0.27 | 0.33 | 16039550 | 5105255047 | 85803118 | 8019774 | 77783343.8 | 0.02 |
| Deaths | Guinea | 2021 | 0.28 | 0.52 | 0.13 | 0.34 | 6958615 | 5105255047 | 92761733 | 3479307 | 89282425.3 | 0.02 |
| Deaths | Afghanistan | 2021 | 0.35 | 0.80 | 0.10 | 0.34 | 16398400 | 5105255047 | 109160135 | 8199201 | 100960934 | 0.02 |
| Deaths | Djibouti | 1992 | 0.36 | 0.63 | 0.19 | 0.34 | 281064.1 | 3376967464 | 409801984 | 140532.1 | 409661452 | 0.12 |
| Deaths | Honduras | 1992 | 0.42 | 0.66 | 0.25 | 0.34 | 2511178 | 3376967464 | 412313162 | 1255589 | 411057573.1 | 0.12 |
| Deaths | India | 1992 | 0.24 | 0.36 | 0.16 | 0.34 | 515839400 | 3376967464 | 928152513 | 257919700 | 670232837.4 | 0.20 |
| Deaths | Kenya | 1992 | 0.21 | 0.31 | 0.14 | 0.35 | 12235470 | 3376967464 | 940387986 | 6117737 | 934270249.4 | 0.28 |
| Deaths | Mauritania | 1992 | 0.35 | 0.61 | 0.19 | 0.35 | 1105278 | 3376967464 | 941493264 | 552639.1 | 940940625.2 | 0.28 |
| Deaths | Lesotho | 1992 | 0.19 | 0.34 | 0.09 | 0.35 | 821010.8 | 3376967464 | 942314275 | 410505.4 | 941903769.7 | 0.28 |
| Deaths | Liberia | 2021 | 0.30 | 0.56 | 0.14 | 0.35 | 3135229 | 5105255047 | 112295364 | 1567614 | 110727749.6 | 0.02 |
| Deaths | Guinea-Bissau | 2021 | 0.42 | 0.73 | 0.21 | 0.35 | 1119372 | 5105255047 | 113414736 | 559686.2 | 112855050.1 | 0.02 |
| Deaths | Nicaragua | 1992 | 0.15 | 0.21 | 0.11 | 0.36 | 2103741 | 3376967464 | 944418017 | 1051871 | 943366145.8 | 0.28 |
| Deaths | Maldives | 1992 | 0.15 | 0.28 | 0.06 | 0.36 | 118284.3 | 3376967464 | 944536301 | 59142.14 | 944477158.7 | 0.28 |
| Deaths | Sierra Leone | 2021 | 0.29 | 0.58 | 0.13 | 0.36 | 5014942 | 5105255047 | 118429679 | 2507471 | 115922207.5 | 0.02 |
| Deaths | Ethiopia | 2021 | 0.20 | 0.30 | 0.11 | 0.36 | 61363610 | 5105255047 | 179793292 | 30681810 | 149111485.3 | 0.03 |
| Deaths | Vanuatu | 1992 | 0.52 | 0.99 | 0.26 | 0.36 | 84103.98 | 3376967464 | 944620405 | 42051.99 | 944578352.8 | 0.28 |
| Deaths | Morocco | 1992 | 0.37 | 0.71 | 0.10 | 0.37 | 15160090 | 3376967464 | 959780492 | 7580044 | 952200448.5 | 0.28 |

| | | | | | | | | | | | | | |
|---|---|---|---|---|---|---|---|---|---|---|---|---|---|
| Deaths | Benin | 2021 | 0.22 | 0.38 | 0.10 | 0.37 | 7048559 | 5105255047 | 186841851 | 3524280 | 183317571.5 | 0.04 |
| Deaths | El Salvador | 1992 | 0.15 | 0.21 | 0.10 | 0.38 | 3000660 | 3376967464 | 962781152 | 1500330 | 961280822.3 | 0.28 |
| Deaths | Democratic Republic of the Congo | 2021 | 0.40 | 0.68 | 0.21 | 0.38 | 49675100 | 5105255047 | 236516952 | 24837550 | 211679401.7 | 0.04 |
| Deaths | Malawi | 2021 | 0.39 | 0.66 | 0.21 | 0.38 | 10785330 | 5105255047 | 247302286 | 5392667 | 241909619.1 | 0.05 |
| Deaths | Ghana | 1992 | 0.42 | 0.69 | 0.23 | 0.38 | 8290370 | 3376967464 | 971071522 | 4145185 | 966926337.2 | 0.29 |
| Deaths | Madagascar | 2021 | 0.60 | 0.99 | 0.32 | 0.40 | 16136660 | 5105255047 | 263438948 | 8068331 | 255370616.9 | 0.05 |
| Deaths | Eritrea | 2021 | 0.36 | 0.63 | 0.19 | 0.40 | 3903951 | 5105255047 | 267342899 | 1951975 | 265390923.2 | 0.05 |
| Deaths | Senegal | 2021 | 0.29 | 0.52 | 0.14 | 0.41 | 8916317 | 5105255047 | 276259216 | 4458159 | 271801057.2 | 0.05 |
| Deaths | Togo | 2021 | 0.33 | 0.58 | 0.17 | 0.41 | 4808399 | 5105255047 | 281067615 | 2404199 | 278663415.3 | 0.05 |
| Deaths | Gambia | 2021 | 0.35 | 0.61 | 0.17 | 0.41 | 1328594 | 5105255047 | 282396209 | 664296.9 | 281731911.6 | 0.06 |
| Deaths | Palestine | 1992 | 0.20 | 0.34 | 0.11 | 0.41 | 1092733 | 3376967464 | 972164255 | 546366.4 | 971617888.3 | 0.29 |
| Deaths | Zimbabwe | 1992 | 0.21 | 0.39 | 0.10 | 0.41 | 5556213 | 3376967464 | 977720467 | 2778106 | 974942361.1 | 0.29 |
| Deaths | Kiribati | 1992 | 1.29 | 2.07 | 0.73 | 0.42 | 43348.47 | 3376967464 | 977763816 | 21674.24 | 977742141.7 | 0.29 |
| Deaths | Eswatini | 1992 | 0.30 | 0.54 | 0.14 | 0.42 | 429553.2 | 3376967464 | 978193369 | 214776.6 | 977978592.6 | 0.29 |
| Deaths | Papua New Guinea | 2021 | 0.39 | 0.74 | 0.19 | 0.42 | 6230283 | 5105255047 | 288626492 | 3115142 | 285511350.2 | 0.06 |
| Deaths | Viet Nam | 1992 | 0.03 | 0.07 | 0.01 | 0.42 | 40268370 | 3376967464 | 1018461738 | 20134180 | 998327553.6 | 0.30 |
| Deaths | Iraq | 1992 | 0.59 | 0.98 | 0.29 | 0.42 | 10043140 | 3376967464 | 1028504876 | 5021569 | 1023483307 | 0.30 |
| Deaths | Tuvalu | 1992 | 0.71 | 1.27 | 0.36 | 0.42 | 5474.875 | 3376967464 | 1028510350 | 2737.437 | 1028507613 | 0.30 |
| Deaths | Uganda | 2021 | 0.28 | 0.48 | 0.14 | 0.42 | 22465620 | 5105255047 | 311092115 | 11232810 | 299859303.5 | 0.06 |
| Deaths | Coted'Ivoire | 2021 | 0.32 | 0.59 | 0.15 | 0.43 | 15562160 | 5105255047 | 326654276 | 7781080 | 318873195.4 | 0.06 |
| Deaths | Solomon Islands | 2021 | 0.32 | 0.57 | 0.16 | 0.43 | 400945.3 | 5105255047 | 327055221 | 200472.7 | 326854748.6 | 0.06 |
| Deaths | Congo | 1992 | 0.61 | 1.07 | 0.31 | 0.43 | 1365818 | 3376967464 | 1029876169 | 682909 | 1029193260 | 0.30 |
| Deaths | Nepal | 2021 | 0.25 | 0.41 | 0.14 | 0.43 | 19987970 | 5105255047 | 347043194 | 9993986 | 337049207.6 | 0.07 |

| | | | | | | | | | | | | |
|---|---|---|---|---|---|---|---|---|---|---|---|---|
| Deaths | Bolivia (Plurinational State of) | 1992 | 0.53 | 0.84 | 0.29 | 0.43 | 3645533 | 3376967464 | 1033521701 | 1822766 | 1031698935 | 0.31 |
| Deaths | Rwanda | 2021 | 0.31 | 0.54 | 0.16 | 0.44 | 7848418 | 5105255047 | 354891612 | 3924209 | 350967402.9 | 0.07 |
| Deaths | Egypt | 1992 | 0.43 | 0.71 | 0.24 | 0.44 | 33112790 | 3376967464 | 1066634488 | 16556390 | 1050078095 | 0.31 |
| Deaths | Botswana | 1992 | 0.32 | 0.58 | 0.16 | 0.44 | 747651.5 | 3376967464 | 1067382140 | 373825.7 | 1067008314 | 0.32 |
| Deaths | Marshall Islands | 1992 | 0.66 | 1.21 | 0.30 | 0.44 | 23628.24 | 3376967464 | 1067405768 | 11814.12 | 1067393954 | 0.32 |
| Deaths | Belize | 1992 | 0.13 | 0.17 | 0.10 | 0.44 | 103326.3 | 3376967464 | 1067509094 | 51663.13 | 1067457431 | 0.32 |
| Deaths | Timor-Leste | 2021 | 0.03 | 0.08 | 0.01 | 0.44 | 802737.4 | 5105255047 | 355694349 | 401368.7 | 355292980.6 | 0.07 |
| Deaths | Syrian Arab Republic | 1992 | 1.24 | 2.06 | 0.66 | 0.45 | 6931728 | 3376967464 | 1074440822 | 3465864 | 1070974958 | 0.32 |
| Deaths | United Republic of Tanzania | 2021 | 0.35 | 0.64 | 0.18 | 0.45 | 32205890 | 5105255047 | 387900240 | 16102950 | 371797294.8 | 0.07 |
| Deaths | Haiti | 2021 | 0.60 | 1.06 | 0.29 | 0.45 | 8005224 | 5105255047 | 395905464 | 4002612 | 391902852.4 | 0.08 |
| Deaths | Dominican Republic | 1992 | 0.36 | 0.54 | 0.23 | 0.45 | 4367623 | 3376967464 | 1078808445 | 2183812 | 1076624634 | 0.32 |
| Deaths | Yemen | 2021 | 0.35 | 0.70 | 0.10 | 0.45 | 18839690 | 5105255047 | 414745156 | 9419846 | 405325310.2 | 0.08 |
| Deaths | Angola | 2021 | 0.45 | 0.75 | 0.24 | 0.45 | 16719890 | 5105255047 | 431465050 | 8359947 | 423105102.9 | 0.08 |
| Deaths | Grenada | 1992 | 0.45 | 0.61 | 0.32 | 0.46 | 48427.92 | 3376967464 | 1078856873 | 24213.96 | 1078832659 | 0.32 |
| Deaths | Oman | 1992 | 0.17 | 0.31 | 0.08 | 0.46 | 1153630 | 3376967464 | 1080010503 | 576815.1 | 1079433688 | 0.32 |
| Deaths | Namibia | 1992 | 0.29 | 0.48 | 0.16 | 0.46 | 804903.9 | 3376967464 | 1080815407 | 402451.9 | 1080412955 | 0.32 |
| Deaths | Guyana | 1992 | 0.88 | 1.10 | 0.69 | 0.47 | 459165.3 | 3376967464 | 1081274573 | 229582.7 | 1081044990 | 0.32 |
| Deaths | Gabon | 1992 | 0.51 | 0.84 | 0.29 | 0.47 | 557425.5 | 3376967464 | 1081831998 | 278712.8 | 1081553285 | 0.32 |
| Deaths | Bhutan | 2021 | 0.24 | 0.40 | 0.13 | 0.47 | 519639.6 | 5105255047 | 431984689 | 259819.8 | 431724869.5 | 0.08 |
| Deaths | Vanuatu | 2021 | 0.48 | 0.82 | 0.25 | 0.47 | 184213.1 | 5105255047 | 432168902 | 92106.55 | 432076795.9 | 0.08 |
| Deaths | Cambodia | 2021 | 0.03 | 0.07 | 0.01 | 0.47 | 10996940 | 5105255047 | 443165844 | 5498471 | 437667373 | 0.09 |
| Deaths | Micronesia (Federated States of) | 1992 | 0.70 | 1.21 | 0.37 | 0.47 | 55679.86 | 3376967464 | 1081887678 | 27839.93 | 1081859838 | 0.32 |
| Deaths | Zimbabwe | 2021 | 0.39 | 0.68 | 0.20 | 0.47 | 8807361 | 5105255047 | 451973205 | 4403681 | 447569524.4 | 0.09 |
| Deaths | Tajikistan | 1992 | 0.00 | 0.01 | 0.00 | 0.48 | 2943035 | 3376967464 | 1084830713 | 1471517 | 1083359195 | 0.32 |

| | | | | | | | | | | | | |
|---|---|---|---|---|---|---|---|---|---|---|---|---|
| Deaths | Indonesia | 1992 | 0.04 | 0.09 | 0.01 | 0.48 | 115716600 | 3376967464 | 1200547263 | 57858280 | 1142688988 | 0.34 |
| Deaths | China | 1992 | 0.04 | 0.06 | 0.03 | 0.48 | 815239200 | 3376967464 | 2015786424 | 407619600 | 1608166844 | 0.48 |
| Deaths | Algeria | 1992 | 0.34 | 0.64 | 0.09 | 0.48 | 14528470 | 3376967464 | 2030314898 | 7264237 | 2023050661 | 0.60 |
| Deaths | Comoros | 2021 | 0.31 | 0.56 | 0.16 | 0.48 | 467233.1 | 5105255047 | 452440438 | 233616.6 | 452206821.7 | 0.09 |
| Deaths | Turkey | 1992 | 0.62 | 1.06 | 0.30 | 0.48 | 36334360 | 3376967464 | 2066649254 | 18167180 | 2048482076 | 0.61 |
| Deaths | Mongolia | 1992 | 0.06 | 0.11 | 0.03 | 0.48 | 1235917 | 3376967464 | 2067885171 | 617958.5 | 2067267212 | 0.61 |
| Deaths | Paraguay | 1992 | 0.69 | 1.00 | 0.45 | 0.48 | 2324234 | 3376967464 | 2070209405 | 1162117 | 2069047288 | 0.61 |
| Deaths | Cameroon | 2021 | 0.34 | 0.59 | 0.17 | 0.48 | 17450670 | 5105255047 | 469891104 | 8725333 | 461165771 | 0.09 |
| Deaths | Iran (Islamic Republic of) | 1992 | 0.37 | 0.53 | 0.25 | 0.48 | 32234900 | 3376967464 | 2102444303 | 16117450 | 2086326854 | 0.62 |
| Deaths | Colombia | 1992 | 0.88 | 1.01 | 0.76 | 0.48 | 20427380 | 3376967464 | 2122871685 | 10213690 | 2112657994 | 0.63 |
| Deaths | Saint Vincent and the Grenadines | 1992 | 0.66 | 0.81 | 0.54 | 0.49 | 63082.62 | 3376967464 | 2122934768 | 31541.31 | 2122903226 | 0.63 |
| Deaths | Djibouti | 2021 | 0.32 | 0.58 | 0.16 | 0.49 | 805751.6 | 5105255047 | 470696855 | 402875.8 | 470293979.5 | 0.09 |
| Deaths | Tunisia | 1992 | 0.33 | 0.60 | 0.09 | 0.49 | 5133459 | 3376967464 | 2128068226 | 2566729 | 2125501497 | 0.63 |
| Deaths | Lao People's Democratic Republic | 2021 | 0.04 | 0.09 | 0.01 | 0.49 | 4754006 | 5105255047 | 475450861 | 2377003 | 473073858.3 | 0.09 |
| Deaths | Bangladesh | 2021 | 0.25 | 0.42 | 0.13 | 0.49 | 107272700 | 5105255047 | 582723583 | 53636360 | 529087222.1 | 0.10 |
| Deaths | Samoa | 1992 | 0.49 | 0.82 | 0.27 | 0.49 | 92361.26 | 3376967464 | 2128160588 | 46180.63 | 2128114407 | 0.63 |
| Deaths | Mauritania | 2021 | 0.27 | 0.48 | 0.14 | 0.50 | 2374531 | 5105255047 | 585098113 | 1187265 | 583910848.2 | 0.11 |
| Deaths | Democratic People's Republic of Korea | 1992 | 0.06 | 0.11 | 0.02 | 0.50 | 13875620 | 3376967464 | 2142036206 | 6937809 | 2135098397 | 0.63 |
| Deaths | Uzbekistan | 1992 | 0.04 | 0.05 | 0.03 | 0.50 | 12096580 | 3376967464 | 2154132781 | 6048288 | 2148084494 | 0.64 |
| Deaths | Nigeria | 2021 | 0.23 | 0.43 | 0.12 | 0.50 | 123377300 | 5105255047 | 708475425 | 61688660 | 646786769.3 | 0.13 |
| Deaths | Tonga | 1992 | 0.33 | 0.55 | 0.19 | 0.50 | 53374.01 | 3376967464 | 2154186155 | 26687 | 2154159468 | 0.64 |
| Deaths | Pakistan | 2021 | 0.31 | 0.47 | 0.19 | 0.50 | 141565600 | 5105255047 | 850041000 | 70782790 | 779258212.8 | 0.15 |

| | | | | | | | | | | | | |
|---|---|---|---|---|---|---|---|---|---|---|---|---|
| Deaths | Sao Tome and Principe | 2021 | 0.31 | 0.61 | 0.15 | 0.51 | 131024.1 | 5105255047 | 850172025 | 65512.04 | 850106512.5 | 0.17 |
| Deaths | Zambia | 2021 | 0.42 | 0.72 | 0.23 | 0.51 | 10789070 | 5105255047 | 860961092 | 5394534 | 855566558.4 | 0.17 |
| Deaths | Brazil | 1992 | 0.93 | 0.99 | 0.88 | 0.51 | 93825900 | 3376967464 | 2248012056 | 46912950 | 2201099106 | 0.65 |
| Deaths | Lesotho | 2021 | 0.36 | 0.63 | 0.18 | 0.51 | 1159988 | 5105255047 | 862121080 | 579994.1 | 861541086.2 | 0.17 |
| Deaths | Suriname | 1992 | 0.41 | 0.62 | 0.25 | 0.51 | 240092.4 | 3376967464 | 2248252148 | 120046.2 | 2248132102 | 0.67 |
| Deaths | Honduras | 2021 | 0.48 | 0.74 | 0.29 | 0.51 | 6325127 | 5105255047 | 868446208 | 3162564 | 865283644 | 0.17 |
| Deaths | Peru | 1992 | 0.30 | 0.45 | 0.19 | 0.51 | 13017280 | 3376967464 | 2261269426 | 6508639 | 2254760787 | 0.67 |
| Deaths | Mexico | 1992 | 0.42 | 0.44 | 0.40 | 0.51 | 51527500 | 3376967464 | 2312796928 | 25763750 | 2287033177 | 0.68 |
| Deaths | Saint Lucia | 1992 | 0.61 | 0.72 | 0.50 | 0.52 | 80360.32 | 3376967464 | 2312877288 | 40180.16 | 2312837108 | 0.68 |
| Deaths | Philippines | 1992 | 0.04 | 0.05 | 0.03 | 0.52 | 37716100 | 3376967464 | 2350593387 | 18858050 | 2331735338 | 0.69 |
| Deaths | Ecuador | 1992 | 0.37 | 0.44 | 0.31 | 0.52 | 6008273 | 3376967464 | 2356601661 | 3004137 | 2353597524 | 0.70 |
| Deaths | Kenya | 2021 | 0.25 | 0.35 | 0.18 | 0.52 | 29742370 | 5105255047 | 898188583 | 14871190 | 883317395.1 | 0.17 |
| Deaths | Nicaragua | 2021 | 0.16 | 0.23 | 0.11 | 0.52 | 4297839 | 5105255047 | 902486421 | 2148919 | 900337502 | 0.18 |
| Deaths | Thailand | 1992 | 0.09 | 0.15 | 0.05 | 0.53 | 39057400 | 3376967464 | 2395659056 | 19528700 | 2376130359 | 0.70 |
| Deaths | Kiribati | 2021 | 1.35 | 2.27 | 0.74 | 0.53 | 74513.75 | 5105255047 | 902560935 | 37256.87 | 902523678.2 | 0.18 |
| Deaths | Kyrgyzstan | 1992 | 0.02 | 0.02 | 0.02 | 0.53 | 2635720 | 3376967464 | 2398294776 | 1317860 | 2396976916 | 0.71 |
| Deaths | Venezuela (Bolivarian Republic of) | 1992 | 0.56 | 0.64 | 0.49 | 0.53 | 11607490 | 3376967464 | 2409902266 | 5803745 | 2404098521 | 0.71 |
| Deaths | Tokelau | 1992 | 0.60 | 1.09 | 0.31 | 0.53 | 834.3087 | 3376967464 | 2409903100 | 417.1543 | 2409902683 | 0.71 |
| Deaths | Sri Lanka | 1992 | 0.23 | 0.33 | 0.15 | 0.53 | 11121210 | 3376967464 | 2421024309 | 5560605 | 2415463705 | 0.72 |
| Deaths | Cabo Verde | 2021 | 0.27 | 0.52 | 0.14 | 0.53 | 382357.4 | 5105255047 | 902943292 | 191178.7 | 902752113.8 | 0.18 |
| Deaths | Myanmar | 2021 | 0.04 | 0.10 | 0.01 | 0.53 | 36996570 | 5105255047 | 939939864 | 18498290 | 921441578.5 | 0.18 |
| Deaths | Nauru | 1992 | 0.99 | 1.74 | 0.48 | 0.54 | 5770.117 | 3376967464 | 2421030079 | 2885.058 | 2421027194 | 0.72 |
| Deaths | Bosnia and Herzegovina | 1992 | 0.29 | 0.52 | 0.14 | 0.54 | 3112775 | 3376967464 | 2424142855 | 1556388 | 2422586467 | 0.72 |

| | | | | | | | | | | | | |
|---|---|---|---|---|---|---|---|---|---|---|---|---|
| Deaths | Guatemala | 2021 | 0.21 | 0.27 | 0.17 | 0.54 | 9913414 | 5105255047 | 949853279 | 4956707 | 944896571.5 | 0.19 |
| Deaths | Tajikistan | 2021 | 0.00 | 0.01 | 0.00 | 0.54 | 6207893 | 5105255047 | 956061172 | 3103947 | 952957225.2 | 0.19 |
| Deaths | Lebanon | 1992 | 1.53 | 2.60 | 0.73 | 0.54 | 1853583 | 3376967464 | 2425996438 | 926791.5 | 2425069646 | 0.72 |
| Deaths | Sudan | 2021 | 0.36 | 0.70 | 0.11 | 0.54 | 25440510 | 5105255047 | 981501682 | 12720260 | 968781427 | 0.19 |
| Deaths | Fiji | 1992 | 0.18 | 0.28 | 0.11 | 0.54 | 465040.9 | 3376967464 | 2426461479 | 232520.5 | 2426228958 | 0.72 |
| Deaths | Costa Rica | 1992 | 0.61 | 0.70 | 0.53 | 0.54 | 1893843 | 3376967464 | 2428355322 | 946921.6 | 2427408400 | 0.72 |
| Deaths | Jamaica | 1992 | 0.07 | 0.09 | 0.05 | 0.55 | 1395283 | 3376967464 | 2429750605 | 697641.5 | 2429052964 | 0.72 |
| Deaths | Jordan | 1992 | 0.37 | 0.54 | 0.23 | 0.55 | 2205439 | 3376967464 | 2431956044 | 1102720 | 2430853325 | 0.72 |
| Deaths | Armenia | 1992 | 0.06 | 0.08 | 0.05 | 0.55 | 2180135 | 3376967464 | 2434136179 | 1090067 | 2433046112 | 0.72 |
| Deaths | South Africa | 1992 | 0.41 | 0.57 | 0.30 | 0.55 | 22998760 | 3376967464 | 2457134939 | 11499380 | 2445635559 | 0.72 |
| Deaths | Albania | 1992 | 0.13 | 0.19 | 0.08 | 0.55 | 2048832 | 3376967464 | 2459183771 | 1024416 | 2458159355 | 0.73 |
| Deaths | Mauritius | 1992 | 0.68 | 0.79 | 0.58 | 0.55 | 729511 | 3376967464 | 2459913282 | 364755.5 | 2459548527 | 0.73 |
| Deaths | Libya | 1992 | 0.29 | 0.56 | 0.08 | 0.55 | 2388892 | 3376967464 | 2462302174 | 1194446 | 2461107728 | 0.73 |
| Deaths | Panama | 1992 | 0.31 | 0.37 | 0.26 | 0.55 | 1507409 | 3376967464 | 2463809583 | 753704.7 | 2463055878 | 0.73 |
| Deaths | Malaysia | 1992 | 0.18 | 0.26 | 0.12 | 0.56 | 11151630 | 3376967464 | 2474961212 | 5575814 | 2469385397 | 0.73 |
| Deaths | Saudi Arabia | 1992 | 0.25 | 0.44 | 0.13 | 0.56 | 9752944 | 3376967464 | 2484714156 | 4876472 | 2479837684 | 0.73 |
| Deaths | Morocco | 2021 | 0.34 | 0.64 | 0.11 | 0.56 | 24647580 | 5105255047 | 1006149263 | 12323790 | 993825472.4 | 0.19 |
| Deaths | El Salvador | 2021 | 0.16 | 0.23 | 0.10 | 0.56 | 4074428 | 5105255047 | 1010223691 | 2037214 | 1008186477 | 0.20 |
| Deaths | Ghana | 2021 | 0.35 | 0.61 | 0.19 | 0.56 | 20161140 | 5105255047 | 1030384835 | 10080570 | 1020304263 | 0.20 |
| Deaths | Turkmenistan | 1992 | 0.01 | 0.01 | 0.01 | 0.57 | 2146841 | 3376967464 | 2486860997 | 1073420 | 2485787576 | 0.74 |
| Deaths | Cuba | 1992 | 0.52 | 0.62 | 0.44 | 0.57 | 7554381 | 3376967464 | 2494415377 | 3777190 | 2490638187 | 0.74 |
| Deaths | Dominica | 1992 | 0.34 | 0.54 | 0.21 | 0.57 | 41551.19 | 3376967464 | 2494456928 | 20775.6 | 2494436153 | 0.74 |
| Deaths | Democratic People's Republic of Korea | 2021 | 0.05 | 0.10 | 0.02 | 0.57 | 18946650 | 5105255047 | 1049331482 | 9473323 | 1039858159 | 0.20 |

| | | | | | | | | | | | | | |
|---|---|---|---|---|---|---|---|---|---|---|---|---|---|
| Deaths | Marshall Islands | 2021 | 0.64 | 1.18 | 0.31 | 0.57 | 36532.79 | 5105255047 | 1049368015 | 18266.39 | 1049349748 | 0.21 |
| Deaths | India | 2021 | 0.27 | 0.35 | 0.20 | 0.58 | 950604900 | 5105255047 | 1999972931 | 475302500 | 1524670473 | 0.30 |
| Deaths | Tuvalu | 2021 | 0.56 | 0.94 | 0.32 | 0.58 | 7770.842 | 5105255047 | 1999980701 | 3885.421 | 1999976816 | 0.39 |
| Deaths | Cook Islands | 1992 | 0.31 | 0.56 | 0.17 | 0.58 | 11553.59 | 3376967464 | 2494468482 | 5776.796 | 2494462705 | 0.74 |
| Deaths | Congo | 2021 | 0.51 | 0.84 | 0.28 | 0.58 | 3289800 | 5105255047 | 2003270502 | 1644900 | 2001625602 | 0.39 |
| Deaths | Eswatini | 2021 | 0.37 | 0.67 | 0.18 | 0.59 | 702572.3 | 5105255047 | 2003973074 | 351286.1 | 2003621788 | 0.39 |
| Deaths | Micronesia (Federated States of) | 2021 | 0.61 | 1.03 | 0.33 | 0.59 | 67172.81 | 5105255047 | 2004040247 | 33586.4 | 2004006661 | 0.39 |
| Deaths | Uruguay | 1992 | 1.06 | 1.21 | 0.92 | 0.59 | 1978724 | 3376967464 | 2496447206 | 989362.1 | 2495457844 | 0.74 |
| Deaths | Seychelles | 1992 | 0.23 | 0.35 | 0.10 | 0.59 | 45508.48 | 3376967464 | 2496492715 | 22754.24 | 2496469961 | 0.74 |
| Deaths | Samoa | 2021 | 0.47 | 0.78 | 0.26 | 0.59 | 122652 | 5105255047 | 2004162899 | 61325.99 | 2004101573 | 0.39 |
| Deaths | Azerbaijan | 1992 | 0.04 | 0.05 | 0.02 | 0.59 | 4664389 | 3376967464 | 2501157104 | 2332195 | 2498824909 | 0.74 |
| Deaths | Bahrain | 1992 | 0.46 | 0.70 | 0.30 | 0.60 | 353670.8 | 3376967464 | 2501510775 | 176835.4 | 2501333939 | 0.74 |
| Deaths | Kazakhstan | 1992 | 0.07 | 0.08 | 0.06 | 0.60 | 10145610 | 3376967464 | 2511656388 | 5072806 | 2506583581 | 0.74 |
| Deaths | Venezuela (Bolivarian Republic of) | 2021 | 0.52 | 0.72 | 0.37 | 0.60 | 17426450 | 5105255047 | 2021589354 | 8713227 | 2012876126 | 0.39 |
| Deaths | Argentina | 1992 | 1.15 | 1.32 | 1.00 | 0.60 | 20617890 | 3376967464 | 2532274273 | 10308940 | 2521965330 | 0.75 |
| Deaths | Bolivia (Plurinational State of) | 2021 | 0.43 | 0.71 | 0.25 | 0.60 | 7560756 | 5105255047 | 2029150109 | 3780378 | 2025369731 | 0.40 |
| Deaths | Chile | 1992 | 0.47 | 0.55 | 0.40 | 0.60 | 8721318 | 3376967464 | 2540995591 | 4360659 | 2536634932 | 0.75 |
| Deaths | Saint Kitts and Nevis | 1992 | 0.47 | 0.56 | 0.37 | 0.60 | 23875.01 | 3376967464 | 2541019466 | 11937.5 | 2541007529 | 0.75 |
| Deaths | Niue | 1992 | 0.58 | 0.98 | 0.32 | 0.60 | 1295.984 | 3376967464 | 2541020762 | 647.9919 | 2541020114 | 0.75 |
| Deaths | Kyrgyzstan | 2021 | 0.05 | 0.07 | 0.04 | 0.60 | 4248862 | 5105255047 | 2033398972 | 2124431 | 2031274540 | 0.40 |
| Deaths | Egypt | 2021 | 0.43 | 0.66 | 0.27 | 0.61 | 64397140 | 5105255047 | 2097796116 | 32198570 | 2065597544 | 0.40 |
| Deaths | Republic of Moldova | 1992 | 0.02 | 0.03 | 0.02 | 0.61 | 2854706 | 3376967464 | 2543875468 | 1427353 | 2542448115 | 0.75 |
| Deaths | Belize | 2021 | 0.14 | 0.18 | 0.11 | 0.61 | 283479.5 | 5105255047 | 2098079595 | 141739.7 | 2097937856 | 0.41 |

| | | | | | | | | | | | | |
|---|---|---|---|---|---|---|---|---|---|---|---|---|
| Deaths | North Macedonia | 1992 | 0.10 | 0.17 | 0.07 | 0.61 | 1323446 | 3376967464 | 2545198915 | 661723.2 | 2544537192 | 0.75 |
| Deaths | Portugal | 1992 | 0.48 | 0.55 | 0.41 | 0.61 | 6780922 | 3376967464 | 2551979837 | 3390461 | 2548589376 | 0.75 |
| Deaths | Namibia | 2021 | 0.29 | 0.50 | 0.15 | 0.62 | 1504481 | 5105255047 | 2099584076 | 752240.5 | 2098831836 | 0.41 |
| Deaths | Mongolia | 2021 | 0.12 | 0.19 | 0.07 | 0.62 | 2105152 | 5105255047 | 2101689228 | 1052576 | 2100636652 | 0.41 |
| Deaths | American Samoa | 1992 | 0.74 | 1.17 | 0.44 | 0.62 | 29749.7 | 3376967464 | 2552009587 | 14874.85 | 2551994712 | 0.76 |
| Deaths | Dominican Republic | 2021 | 0.37 | 0.58 | 0.23 | 0.62 | 7231298 | 5105255047 | 2108920526 | 3615649 | 2105304877 | 0.41 |
| Deaths | Syrian Arab Republic | 2021 | 0.84 | 1.38 | 0.45 | 0.62 | 9352995 | 5105255047 | 2118273521 | 4676498 | 2113597023 | 0.41 |
| Deaths | Antigua and Barbuda | 1992 | 0.33 | 0.39 | 0.26 | 0.62 | 38493.21 | 3376967464 | 2552048080 | 19246.61 | 2552028833 | 0.76 |
| Deaths | Nauru | 2021 | 0.86 | 1.52 | 0.44 | 0.63 | 6647.105 | 5105255047 | 2118280168 | 3323.553 | 2118276845 | 0.41 |
| Deaths | Tonga | 2021 | 0.31 | 0.55 | 0.17 | 0.63 | 60598.31 | 5105255047 | 2118340767 | 30299.16 | 2118310467 | 0.41 |
| Deaths | Viet Nam | 2021 | 0.03 | 0.07 | 0.01 | 0.63 | 67836850 | 5105255047 | 2186177612 | 33918420 | 2152259189 | 0.42 |
| Deaths | Romania | 1992 | 0.21 | 0.25 | 0.17 | 0.63 | 15484770 | 3376967464 | 2567532852 | 7742386 | 2559790466 | 0.76 |
| Deaths | Palestine | 2021 | 0.16 | 0.24 | 0.10 | 0.63 | 3092703 | 5105255047 | 2189270315 | 1546351 | 2187723963 | 0.43 |
| Deaths | Belarus | 1992 | 0.02 | 0.03 | 0.02 | 0.63 | 6938289 | 3376967464 | 2574471141 | 3469144 | 2571001997 | 0.76 |
| Deaths | Suriname | 2021 | 0.36 | 0.60 | 0.20 | 0.63 | 384154.9 | 5105255047 | 2189654470 | 192077.4 | 2189462392 | 0.43 |
| Deaths | Trinidad and Tobago | 1992 | 0.27 | 0.33 | 0.23 | 0.63 | 745716.6 | 3376967464 | 2575216858 | 372858.3 | 2574843999 | 0.76 |
| Deaths | Gabon | 2021 | 0.46 | 0.78 | 0.26 | 0.63 | 1102224 | 5105255047 | 2190756693 | 551111.8 | 2190205581 | 0.43 |
| Deaths | Paraguay | 2021 | 0.86 | 1.27 | 0.56 | 0.64 | 4680073 | 5105255047 | 2195436766 | 2340037 | 2193096730 | 0.43 |
| Deaths | Saint Vincent and the Grenadines | 2021 | 0.50 | 0.62 | 0.40 | 0.64 | 76552.59 | 5105255047 | 2195513319 | 38276.3 | 2195475043 | 0.43 |
| Deaths | Poland | 1992 | 0.19 | 0.20 | 0.18 | 0.64 | 24967360 | 3376967464 | 2600184220 | 12483680 | 2587700539 | 0.77 |
| Deaths | Serbia | 1992 | 0.47 | 0.72 | 0.28 | 0.64 | 6574516 | 3376967464 | 2606758736 | 3287258 | 2603471478 | 0.77 |
| Deaths | Botswana | 2021 | 0.21 | 0.36 | 0.11 | 0.64 | 1590402 | 5105255047 | 2197103721 | 795200.9 | 2196308520 | 0.43 |
| Deaths | Guyana | 2021 | 1.06 | 1.44 | 0.75 | 0.65 | 501391.8 | 5105255047 | 2197605113 | 250695.9 | 2197354417 | 0.43 |
| Deaths | Maldives | 2021 | 0.12 | 0.19 | 0.07 | 0.65 | 394937.9 | 5105255047 | 2198000050 | 197468.9 | 2197802582 | 0.43 |

| | | | | | | | | | | | | | | |
|---|---|---|---|---|---|---|---|---|---|---|---|---|---|---|
| Deaths | Bulgaria | 1992 | 0.07 | 0.08 | 0.06 | 0.65 | 5699135 | 3376967464 | 2612457871 | 2849568 | 2609608303 | 0.77 |
| Deaths | Philippines | 2021 | 0.06 | 0.08 | 0.04 | 0.65 | 73079830 | 5105255047 | 2271079884 | 36539920 | 2234539967 | 0.44 |
| Deaths | Spain | 1992 | 1.08 | 1.24 | 0.93 | 0.65 | 26113210 | 3376967464 | 2638571077 | 13056600 | 2625514474 | 0.78 |
| Deaths | Brazil | 2021 | 0.54 | 0.57 | 0.51 | 0.65 | 150405800 | 5105255047 | 2421485716 | 75202920 | 2346282800 | 0.46 |
| Deaths | Colombia | 2021 | 0.59 | 0.74 | 0.46 | 0.66 | 33609590 | 5105255047 | 2455095302 | 16804790 | 2438290509 | 0.48 |
| Deaths | Indonesia | 2021 | 0.04 | 0.08 | 0.01 | 0.66 | 194150400 | 5105255047 | 2649245676 | 97075190 | 2552170489 | 0.50 |
| Deaths | Ukraine | 1992 | 0.04 | 0.05 | 0.03 | 0.66 | 34804310 | 3376967464 | 2673375387 | 17402160 | 2655973232 | 0.79 |
| Deaths | Equatorial Guinea | 2021 | 0.40 | 0.75 | 0.19 | 0.66 | 893663 | 5105255047 | 2650139339 | 446831.5 | 2649692507 | 0.52 |
| Deaths | Algeria | 2021 | 0.28 | 0.52 | 0.08 | 0.66 | 28054400 | 5105255047 | 2678193741 | 14027200 | 2664166540 | 0.52 |
| Deaths | Hungary | 1992 | 1.18 | 1.34 | 1.03 | 0.66 | 6927328 | 3376967464 | 2680302715 | 3463664 | 2676839051 | 0.79 |
| Deaths | Qatar | 1992 | 0.53 | 0.83 | 0.32 | 0.66 | 335643.3 | 3376967464 | 2680638358 | 167821.6 | 2680470537 | 0.79 |
| Deaths | Ecuador | 2021 | 0.39 | 0.51 | 0.28 | 0.66 | 11574520 | 5105255047 | 2689768262 | 5787261 | 2683981001 | 0.53 |
| Deaths | Peru | 2021 | 0.26 | 0.40 | 0.16 | 0.66 | 23885430 | 5105255047 | 2713653696 | 11942720 | 2701710979 | 0.53 |
| Deaths | Barbados | 1992 | 0.52 | 0.61 | 0.43 | 0.66 | 162757.9 | 3376967464 | 2680801116 | 81378.93 | 2680719737 | 0.79 |
| Deaths | Uzbekistan | 2021 | 0.11 | 0.14 | 0.09 | 0.66 | 22329360 | 5105255047 | 2735983057 | 11164680 | 2724818377 | 0.53 |
| Deaths | Iraq | 2021 | 0.45 | 0.77 | 0.21 | 0.66 | 26081420 | 5105255047 | 2762064476 | 13040710 | 2749023766 | 0.54 |
| Deaths | Mexico | 2021 | 0.40 | 0.46 | 0.35 | 0.66 | 86646750 | 5105255047 | 2848711229 | 43323380 | 2805387853 | 0.55 |
| Deaths | Georgia | 1992 | 0.04 | 0.05 | 0.03 | 0.67 | 3570243 | 3376967464 | 2684371360 | 1785122 | 2682586238 | 0.79 |
| Deaths | Slovakia | 1992 | 0.25 | 0.36 | 0.17 | 0.67 | 3469779 | 3376967464 | 2687841139 | 1734890 | 2686106249 | 0.80 |
| Deaths | Cyprus | 1992 | 1.11 | 1.69 | 0.72 | 0.67 | 522214.5 | 3376967464 | 2688363353 | 261107.2 | 2688102246 | 0.80 |
| Deaths | Malta | 1992 | 0.30 | 0.35 | 0.26 | 0.67 | 249227.5 | 3376967464 | 2688612581 | 124613.8 | 2688487967 | 0.80 |
| Deaths | Guam | 1992 | 1.09 | 1.54 | 0.75 | 0.67 | 93009.67 | 3376967464 | 2688705591 | 46504.84 | 2688659086 | 0.80 |
| Deaths | Puerto Rico | 1992 | 0.75 | 0.88 | 0.64 | 0.67 | 2339319 | 3376967464 | 2691044910 | 1169660 | 2689875250 | 0.80 |
| Deaths | Cuba | 2021 | 0.50 | 0.61 | 0.40 | 0.67 | 7721598 | 5105255047 | 2856432827 | 3860799 | 2852572028 | 0.56 |

| | | | | | | | | | | | | |
|---|---|---|---|---|---|---|---|---|---|---|---|---|
| Deaths | Grenada | 2021 | 0.68 | 0.87 | 0.53 | 0.67 | 71465.1 | 5105255047 | 2856504292 | 35732.55 | 2856468560 | 0.56 |
| Deaths | Kuwait | 1992 | 0.35 | 0.41 | 0.29 | 0.67 | 1142046 | 3376967464 | 2692186956 | 571023.1 | 2691615933 | 0.80 |
| Deaths | United States Virgin Islands | 1992 | 0.77 | 1.18 | 0.48 | 0.67 | 68428.68 | 3376967464 | 2692255385 | 34214.34 | 2692221171 | 0.80 |
| Deaths | Saint Lucia | 2021 | 0.36 | 0.46 | 0.28 | 0.67 | 127254.6 | 5105255047 | 2856631547 | 63627.3 | 2856567920 | 0.56 |
| Deaths | Montenegro | 1992 | 0.16 | 0.24 | 0.11 | 0.67 | 417608.2 | 3376967464 | 2692672993 | 208804.1 | 2692464189 | 0.80 |
| Deaths | Palau | 1992 | 0.53 | 0.90 | 0.27 | 0.67 | 10631.73 | 3376967464 | 2692683625 | 5315.866 | 2692678309 | 0.80 |
| Deaths | Fiji | 2021 | 0.16 | 0.25 | 0.09 | 0.68 | 595530.4 | 5105255047 | 2857227077 | 297765.2 | 2856929312 | 0.56 |
| Deaths | United Arab Emirates | 1992 | 1.56 | 2.46 | 0.92 | 0.68 | 1447269 | 3376967464 | 2694130893 | 723634.3 | 2693407259 | 0.80 |
| Deaths | Brunei Darussalam | 1992 | 0.42 | 0.63 | 0.26 | 0.68 | 172733 | 3376967464 | 2694303626 | 86366.52 | 2694217260 | 0.80 |
| Deaths | Croatia | 1992 | 0.28 | 0.33 | 0.24 | 0.68 | 3329713 | 3376967464 | 2697633339 | 1664857 | 2695968483 | 0.80 |
| Deaths | South Africa | 2021 | 0.33 | 0.45 | 0.25 | 0.68 | 37983600 | 5105255047 | 2895210682 | 18991800 | 2876218879 | 0.56 |
| Deaths | Lithuania | 1992 | 0.18 | 0.22 | 0.15 | 0.68 | 2447071 | 3376967464 | 2700080410 | 1223536 | 2698856875 | 0.80 |
| Deaths | Turkmenistan | 2021 | 0.07 | 0.09 | 0.05 | 0.68 | 3350351 | 5105255047 | 2898561033 | 1675176 | 2896885857 | 0.57 |
| Deaths | Tunisia | 2021 | 0.28 | 0.56 | 0.08 | 0.68 | 7946659 | 5105255047 | 2906507692 | 3973329 | 2902534362 | 0.57 |
| Deaths | Thailand | 2021 | 0.17 | 0.27 | 0.10 | 0.68 | 47274310 | 5105255047 | 2953782004 | 23637160 | 2930144848 | 0.57 |
| Deaths | Jamaica | 2021 | 0.10 | 0.14 | 0.07 | 0.68 | 1946551 | 5105255047 | 2955728554 | 973275.3 | 2954755279 | 0.58 |
| Deaths | Taiwan (Province of China) | 1992 | 0.29 | 0.34 | 0.25 | 0.68 | 14026120 | 3376967464 | 2714106526 | 7013058 | 2707093468 | 0.80 |
| Deaths | Tokelau | 2021 | 0.60 | 1.04 | 0.34 | 0.69 | 843.5528 | 5105255047 | 2955729398 | 421.7764 | 2955728976 | 0.58 |
| Deaths | Greece | 1992 | 0.44 | 0.50 | 0.38 | 0.69 | 7122789 | 3376967464 | 2721229315 | 3561394 | 2717667921 | 0.80 |
| Deaths | Bahamas | 1992 | 0.38 | 0.45 | 0.31 | 0.69 | 171499.1 | 3376967464 | 2721400814 | 85749.56 | 2721315065 | 0.81 |
| Deaths | Russian Federation | 1992 | 0.10 | 0.11 | 0.10 | 0.69 | 101681600 | 3376967464 | 2823082430 | 50840810 | 2772241622 | 0.82 |
| Deaths | Azerbaijan | 2021 | 0.02 | 0.03 | 0.01 | 0.69 | 7440503 | 5105255047 | 2963169901 | 3720252 | 2959449649 | 0.58 |
| Deaths | Estonia | 1992 | 0.50 | 0.61 | 0.42 | 0.70 | 1018565 | 3376967464 | 2824100995 | 509282.4 | 2823591712 | 0.84 |
| Deaths | Latvia | 1992 | 0.29 | 0.34 | 0.25 | 0.70 | 1746906 | 3376967464 | 2825847901 | 873453 | 2824974448 | 0.84 |

| | | | | | | | | | | | | |
|---|---|---|---|---|---|---|---|---|---|---|---|---|
| Deaths | Iran (Islamic Republic of) | 2021 | 0.28 | 0.41 | 0.22 | 0.70 | 59167520 | 5105255047 | 3022337423 | 29583760 | 2992753662 | 0.59 |
| Deaths | Czechia | 1992 | 0.18 | 0.21 | 0.15 | 0.70 | 6884471 | 3376967464 | 2832732372 | 3442236 | 2829290136 | 0.84 |
| Deaths | Costa Rica | 2021 | 0.69 | 0.83 | 0.56 | 0.70 | 3249833 | 5105255047 | 3025587256 | 1624917 | 3023962339 | 0.59 |
| Deaths | Sri Lanka | 2021 | 0.13 | 0.22 | 0.07 | 0.70 | 14714740 | 5105255047 | 3040301997 | 7357370 | 3032944626 | 0.59 |
| Deaths | Armenia | 2021 | 0.09 | 0.11 | 0.07 | 0.70 | 2004496 | 5105255047 | 3042306493 | 1002248 | 3041304245 | 0.60 |
| Deaths | Singapore | 1992 | 0.25 | 0.29 | 0.21 | 0.70 | 2369939 | 3376967464 | 2835102310 | 1184969 | 2833917341 | 0.84 |
| Deaths | Bermuda | 1992 | 2.73 | 3.21 | 2.25 | 0.70 | 42685.4 | 3376967464 | 2835144996 | 21342.7 | 2835123653 | 0.84 |
| Deaths | Albania | 2021 | 0.18 | 0.27 | 0.11 | 0.71 | 1808384 | 5105255047 | 3044114877 | 904192 | 3043210685 | 0.60 |
| Deaths | Panama | 2021 | 0.39 | 0.50 | 0.29 | 0.71 | 2749507 | 5105255047 | 3046864384 | 1374753 | 3045489630 | 0.60 |
| Deaths | Republic of Korea | 1992 | 0.18 | 0.26 | 0.11 | 0.71 | 31435440 | 3376967464 | 2866580440 | 15717720 | 2850862718 | 0.84 |
| Deaths | Turkey | 2021 | 0.39 | 0.61 | 0.24 | 0.71 | 56921960 | 5105255047 | 3103786340 | 28460980 | 3075325362 | 0.60 |
| Deaths | Northern Mariana Islands | 1992 | 1.03 | 1.60 | 0.63 | 0.72 | 33889.48 | 3376967464 | 2866614329 | 16944.74 | 2866597384 | 0.85 |
| Deaths | Italy | 1992 | 0.54 | 0.57 | 0.52 | 0.72 | 38983200 | 3376967464 | 2905597533 | 19491600 | 2886105931 | 0.85 |
| Deaths | Israel | 1992 | 0.53 | 0.62 | 0.45 | 0.72 | 3155772 | 3376967464 | 2908753305 | 1577886 | 2907175419 | 0.86 |
| Deaths | Mauritius | 2021 | 0.56 | 0.66 | 0.47 | 0.72 | 900058.5 | 5105255047 | 3104686398 | 450029.2 | 3104236369 | 0.61 |
| Deaths | Uruguay | 2021 | 1.03 | 1.19 | 0.88 | 0.72 | 2214747 | 5105255047 | 3106901145 | 1107373 | 3105793772 | 0.61 |
| Deaths | China | 2021 | 0.03 | 0.04 | 0.02 | 0.72 | 967106700 | 5105255047 | 4074007884 | 483553400 | 3590454514 | 0.70 |
| Deaths | Bosnia and Herzegovina | 2021 | 0.28 | 0.45 | 0.15 | 0.72 | 2206873 | 5105255047 | 4076214757 | 1103437 | 4075111320 | 0.80 |
| Deaths | Argentina | 2021 | 0.66 | 0.77 | 0.57 | 0.72 | 30052210 | 5105255047 | 4106266964 | 15026100 | 4091240861 | 0.80 |
| Deaths | American Samoa | 2021 | 0.62 | 0.97 | 0.37 | 0.72 | 31908.17 | 5105255047 | 4106298872 | 15954.09 | 4106282918 | 0.80 |
| Deaths | Kazakhstan | 2021 | 0.08 | 0.11 | 0.07 | 0.73 | 12126520 | 5105255047 | 4118425393 | 6063260 | 4112362132 | 0.81 |
| Deaths | Jordan | 2021 | 0.20 | 0.29 | 0.13 | 0.73 | 8180007 | 5105255047 | 4126605399 | 4090003 | 4122515396 | 0.81 |
| Deaths | Libya | 2021 | 0.47 | 1.03 | 0.11 | 0.73 | 5029432 | 5105255047 | 4131634831 | 2514716 | 4129120115 | 0.81 |
| Deaths | Niue | 2021 | 0.57 | 0.99 | 0.33 | 0.73 | 1099.18 | 5105255047 | 4131635930 | 549.5901 | 4131635381 | 0.81 |

| | | | | | | | | | | | | |
|---|---|---|---|---|---|---|---|---|---|---|---|---|
| Deaths | Greenland | 1992 | 1.54 | 2.44 | 0.76 | 0.73 | 38395.59 | 3376967464 | 2908791700 | 19197.79 | 2908772503 | 0.86 |
| Deaths | Seychelles | 2021 | 0.15 | 0.23 | 0.06 | 0.73 | 72957.22 | 5105255047 | 4131708887 | 36478.61 | 4131672409 | 0.81 |
| Deaths | Ireland | 1992 | 0.54 | 0.62 | 0.46 | 0.73 | 2251651 | 3376967464 | 2911043351 | 1125825 | 2909917526 | 0.86 |
| Deaths | Republic of Moldova | 2021 | 0.09 | 0.11 | 0.07 | 0.73 | 2516382 | 5105255047 | 4134225269 | 1258191 | 4132967078 | 0.81 |
| Deaths | Georgia | 2021 | 0.37 | 0.45 | 0.29 | 0.73 | 2299321 | 5105255047 | 4136524590 | 1149660 | 4135374929 | 0.81 |
| Deaths | Australia | 1992 | 0.58 | 0.67 | 0.51 | 0.73 | 11484530 | 3376967464 | 2922527885 | 5742267 | 2916785618 | 0.86 |
| Deaths | Slovenia | 1992 | 0.91 | 1.03 | 0.80 | 0.74 | 1357830 | 3376967464 | 2923885715 | 678914.9 | 2923206800 | 0.87 |
| Deaths | Malaysia | 2021 | 0.16 | 0.23 | 0.10 | 0.74 | 21856660 | 5105255047 | 4158381246 | 10928330 | 4147452918 | 0.81 |
| Deaths | France | 1992 | 0.95 | 1.10 | 0.81 | 0.74 | 38242170 | 3376967464 | 2962127885 | 19121090 | 2943006800 | 0.87 |
| Deaths | Portugal | 2021 | 0.46 | 0.53 | 0.40 | 0.74 | 6825510 | 5105255047 | 4165206755 | 3412755 | 4161794000 | 0.82 |
| Deaths | Lebanon | 2021 | 0.65 | 0.97 | 0.42 | 0.74 | 3716347 | 5105255047 | 4168923102 | 1858174 | 4167064929 | 0.82 |
| Deaths | Barbados | 2021 | 0.48 | 0.65 | 0.36 | 0.75 | 202783.5 | 5105255047 | 4169125886 | 101391.7 | 4169024494 | 0.82 |
| Deaths | Dominica | 2021 | 0.42 | 0.68 | 0.25 | 0.75 | 46130.66 | 5105255047 | 4169172016 | 23065.33 | 4169148951 | 0.82 |
| Deaths | Belgium | 1992 | 0.78 | 0.90 | 0.67 | 0.75 | 6685776 | 3376967464 | 2968813661 | 3342888 | 2965470773 | 0.88 |
| Deaths | Antigua and Barbuda | 2021 | 0.24 | 0.29 | 0.20 | 0.75 | 63578.22 | 5105255047 | 4169235595 | 31789.11 | 4169203806 | 0.82 |
| Deaths | North Macedonia | 2021 | 0.18 | 0.27 | 0.12 | 0.75 | 1541196 | 5105255047 | 4170776791 | 770598.2 | 4170006193 | 0.82 |
| Deaths | Bahrain | 2021 | 0.32 | 0.47 | 0.21 | 0.75 | 1178377 | 5105255047 | 4171955168 | 589188.4 | 4171365980 | 0.82 |
| Deaths | Palau | 2021 | 0.49 | 0.83 | 0.27 | 0.75 | 13185.18 | 5105255047 | 4171968353 | 6592.589 | 4171961761 | 0.82 |
| Deaths | Austria | 1992 | 0.68 | 0.79 | 0.58 | 0.75 | 5270737 | 3376967464 | 2974084397 | 2635368 | 2971449029 | 0.88 |
| Deaths | United Kingdom | 1992 | 0.63 | 0.65 | 0.61 | 0.75 | 37397090 | 3376967464 | 3011481485 | 18698540 | 2992782941 | 0.89 |
| Deaths | Saint Kitts and Nevis | 2021 | 0.32 | 0.43 | 0.24 | 0.75 | 43420.68 | 5105255047 | 4172011774 | 21710.34 | 4171990064 | 0.82 |
| Deaths | New Zealand | 1992 | 0.74 | 0.84 | 0.65 | 0.76 | 2285123 | 3376967464 | 3013766608 | 1142561 | 3012624047 | 0.89 |
| Deaths | Ukraine | 2021 | 0.17 | 0.25 | 0.12 | 0.76 | 29287980 | 5105255047 | 4201299752 | 14643990 | 4186655763 | 0.82 |
| Deaths | Finland | 1992 | 0.77 | 0.89 | 0.65 | 0.76 | 3388185 | 3376967464 | 3017154793 | 1694093 | 3015460701 | 0.89 |

| | | | | | | | | | | | | |
|---|---|---|---|---|---|---|---|---|---|---|---|---|
| Deaths | Andorra | 1992 | 0.42 | 0.67 | 0.24 | 0.77 | 45683.08 | 3376967464 | 3017200476 | 22841.54 | 3017177635 | 0.89 |
| Deaths | Bulgaria | 2021 | 0.29 | 0.36 | 0.24 | 0.77 | 4337506 | 5105255047 | 4205637258 | 2168753 | 4203468505 | 0.82 |
| Deaths | Romania | 2021 | 0.29 | 0.35 | 0.24 | 0.77 | 12142540 | 5105255047 | 4217779801 | 6071271 | 4211708529 | 0.82 |
| Deaths | Trinidad and Tobago | 2021 | 0.30 | 0.41 | 0.22 | 0.77 | 942563.1 | 5105255047 | 4218722364 | 471281.5 | 4218251082 | 0.83 |
| Deaths | Spain | 2021 | 0.45 | 0.51 | 0.39 | 0.77 | 29873650 | 5105255047 | 4248596015 | 14936830 | 4233659190 | 0.83 |
| Deaths | United States of America | 1992 | 0.85 | 0.89 | 0.82 | 0.77 | 169626400 | 3376967464 | 3186826882 | 84813200 | 3102013679 | 0.92 |
| Deaths | Chile | 2021 | 0.29 | 0.35 | 0.24 | 0.77 | 12819950 | 5105255047 | 4261415966 | 6409975 | 4255005991 | 0.83 |
| Deaths | Northern Mariana Islands | 2021 | 0.91 | 1.36 | 0.58 | 0.77 | 33644 | 5105255047 | 4261449610 | 16822 | 4261432788 | 0.83 |
| Deaths | Iceland | 1992 | 0.57 | 0.66 | 0.48 | 0.77 | 167194.9 | 3376967464 | 3186994077 | 83597.46 | 3186910480 | 0.94 |
| Deaths | Oman | 2021 | 0.11 | 0.18 | 0.06 | 0.77 | 3365550 | 5105255047 | 4264815160 | 1682775 | 4263132385 | 0.84 |
| Deaths | Cook Islands | 2021 | 0.21 | 0.41 | 0.10 | 0.78 | 11633.24 | 5105255047 | 4264826793 | 5816.619 | 4264820977 | 0.84 |
| Deaths | Belarus | 2021 | 0.08 | 0.10 | 0.06 | 0.78 | 6254502 | 5105255047 | 4271081295 | 3127251 | 4267954044 | 0.84 |
| Deaths | Canada | 1992 | 0.51 | 0.59 | 0.44 | 0.79 | 18901940 | 3376967464 | 3205896013 | 9450968 | 3196445045 | 0.95 |
| Deaths | Luxembourg | 1992 | 0.87 | 1.00 | 0.76 | 0.79 | 268190.4 | 3376967464 | 3206164203 | 134095.2 | 3206030108 | 0.95 |
| Deaths | Hungary | 2021 | 1.28 | 1.47 | 1.11 | 0.79 | 6199148 | 5105255047 | 4277280443 | 3099574 | 4274180869 | 0.84 |
| Deaths | Greece | 2021 | 0.47 | 0.54 | 0.40 | 0.79 | 6469340 | 5105255047 | 4283749783 | 3234670 | 4280515113 | 0.84 |
| Deaths | Serbia | 2021 | 0.53 | 0.77 | 0.34 | 0.79 | 5934126 | 5105255047 | 4289683909 | 2967063 | 4286716846 | 0.84 |
| Deaths | Sweden | 1992 | 0.58 | 0.66 | 0.51 | 0.80 | 5553552 | 3376967464 | 3211717756 | 2776776 | 3208940980 | 0.95 |
| Deaths | Montenegro | 2021 | 0.16 | 0.25 | 0.11 | 0.80 | 412912.2 | 5105255047 | 4290096822 | 206456.1 | 4289890365 | 0.84 |
| Deaths | Croatia | 2021 | 0.65 | 0.78 | 0.53 | 0.80 | 2715472 | 5105255047 | 4292812293 | 1357736 | 4291454558 | 0.84 |
| Deaths | Japan | 1992 | 0.65 | 0.68 | 0.63 | 0.80 | 88370150 | 3376967464 | 3300087906 | 44185070 | 3255902831 | 0.96 |
| Deaths | Malta | 2021 | 0.34 | 0.40 | 0.28 | 0.80 | 278137.3 | 5105255047 | 4293090431 | 139068.6 | 4292951362 | 0.84 |
| Deaths | Netherlands | 1992 | 0.73 | 0.86 | 0.63 | 0.80 | 10396300 | 3376967464 | 3310484202 | 5198148 | 3305286054 | 0.98 |
| Deaths | Guam | 2021 | 0.80 | 1.11 | 0.57 | 0.80 | 103477.4 | 5105255047 | 4293193908 | 51738.72 | 4293142170 | 0.84 |

| | | | | | | | | | | | | |
|---|---|---|---|---|---|---|---|---|---|---|---|---|
| Deaths | Bahamas | 2021 | 0.39 | 0.52 | 0.30 | 0.81 | 274938.4 | 5105255047 | 4293468847 | 137469.2 | 4293331377 | 0.84 |
| Deaths | Norway | 1992 | 0.64 | 0.68 | 0.61 | 0.81 | 2768970 | 3376967464 | 3313253172 | 1384485 | 3311868687 | 0.98 |
| Deaths | Italy | 2021 | 0.38 | 0.40 | 0.35 | 0.81 | 38186670 | 5105255047 | 4331655515 | 19093330 | 4312562181 | 0.84 |
| Deaths | Denmark | 1992 | 0.62 | 0.72 | 0.53 | 0.81 | 3484450 | 3376967464 | 3316737622 | 1742225 | 3314995397 | 0.98 |
| Deaths | Russian Federation | 2021 | 0.40 | 0.44 | 0.36 | 0.81 | 96028800 | 5105255047 | 4427684315 | 48014400 | 4379669915 | 0.86 |
| Deaths | Israel | 2021 | 0.40 | 0.47 | 0.34 | 0.81 | 5769267 | 5105255047 | 4433453582 | 2884633 | 4430568949 | 0.87 |
| Deaths | Brunei Darussalam | 2021 | 0.39 | 0.58 | 0.25 | 0.81 | 332019.1 | 5105255047 | 4433785601 | 166009.6 | 4433619592 | 0.87 |
| Deaths | Slovakia | 2021 | 0.37 | 0.51 | 0.25 | 0.81 | 3635982 | 5105255047 | 4437421583 | 1817991 | 4435603592 | 0.87 |
| Deaths | Poland | 2021 | 0.74 | 0.82 | 0.67 | 0.81 | 25179790 | 5105255047 | 4462601375 | 12589900 | 4450011479 | 0.87 |
| Deaths | Saudi Arabia | 2021 | 0.30 | 0.50 | 0.17 | 0.82 | 29117100 | 5105255047 | 4491718473 | 14558550 | 4477159924 | 0.88 |
| Deaths | Bermuda | 2021 | 1.56 | 1.97 | 1.22 | 0.82 | 41969.77 | 5105255047 | 4491760443 | 20984.88 | 4491739458 | 0.88 |
| Deaths | United States Virgin Islands | 2021 | 0.54 | 0.93 | 0.29 | 0.82 | 53866.94 | 5105255047 | 4491814310 | 26933.47 | 4491787376 | 0.88 |
| Deaths | San Marino | 1992 | 0.20 | 0.30 | 0.13 | 0.82 | 16789.73 | 3376967464 | 3316754411 | 8394.865 | 3316746017 | 0.98 |
| Deaths | Puerto Rico | 2021 | 0.74 | 0.93 | 0.57 | 0.83 | 2124836 | 5105255047 | 4493939146 | 1062418 | 4492876728 | 0.88 |
| Deaths | Greenland | 2021 | 0.82 | 1.25 | 0.48 | 0.83 | 39064.81 | 5105255047 | 4493978210 | 19532.41 | 4493958678 | 0.88 |
| Deaths | Germany | 1992 | 0.98 | 1.14 | 0.84 | 0.83 | 55437780 | 3376967464 | 3372192189 | 27718890 | 3344473300 | 0.99 |
| Deaths | Czechia | 2021 | 0.59 | 0.70 | 0.49 | 0.83 | 6708178 | 5105255047 | 4500686388 | 3354089 | 4497332299 | 0.88 |
| Deaths | Latvia | 2021 | 0.74 | 0.89 | 0.61 | 0.83 | 1180840 | 5105255047 | 4501867228 | 590420 | 4501276808 | 0.88 |
| Deaths | Cyprus | 2021 | 0.66 | 0.97 | 0.45 | 0.84 | 940775.7 | 5105255047 | 4502808004 | 470387.8 | 4502337616 | 0.88 |
| Deaths | France | 2021 | 0.40 | 0.47 | 0.34 | 0.84 | 41021790 | 5105255047 | 4543829797 | 20510900 | 4523318900 | 0.89 |
| Deaths | Slovenia | 2021 | 1.22 | 1.47 | 0.98 | 0.84 | 1320405 | 5105255047 | 4545150201 | 660202.5 | 4544489999 | 0.89 |
| Deaths | Australia | 2021 | 0.31 | 0.36 | 0.27 | 0.84 | 16656050 | 5105255047 | 4561806249 | 8328024 | 4553478225 | 0.89 |
| Deaths | Estonia | 2021 | 1.05 | 1.26 | 0.87 | 0.84 | 825205.3 | 5105255047 | 4562631454 | 412602.6 | 4562218851 | 0.89 |
| Deaths | Kuwait | 2021 | 0.24 | 0.31 | 0.19 | 0.85 | 3633714 | 5105255047 | 4566265168 | 1816857 | 4564448311 | 0.89 |

| | | | | | | | | | | | | |
|---|---|---|---|---|---|---|---|---|---|---|---|---|
| Deaths | Qatar | 2021 | 0.24 | 0.40 | 0.13 | 0.85 | 2446107 | 5105255047 | 4568711276 | 1223054 | 4567488222 | 0.89 |
| Deaths | United Arab Emirates | 2021 | 0.60 | 0.91 | 0.38 | 0.85 | 8129433 | 5105255047 | 4576840709 | 4064717 | 4572775992 | 0.90 |
| Deaths | New Zealand | 2021 | 0.51 | 0.57 | 0.45 | 0.85 | 3378026 | 5105255047 | 4580218735 | 1689013 | 4578529722 | 0.90 |
| Deaths | Monaco | 1992 | 0.23 | 0.36 | 0.14 | 0.85 | 19942.64 | 3376967464 | 3372212132 | 9971.318 | 3372202160 | 1.00 |
| Deaths | Belgium | 2021 | 0.53 | 0.62 | 0.46 | 0.85 | 7314275 | 5105255047 | 4587533010 | 3657137 | 4583875872 | 0.90 |
| Deaths | Austria | 2021 | 0.38 | 0.45 | 0.33 | 0.85 | 5970418 | 5105255047 | 4593503427 | 2985209 | 4590518219 | 0.90 |
| Deaths | Singapore | 2021 | 0.08 | 0.09 | 0.07 | 0.86 | 4146887 | 5105255047 | 4597650315 | 2073444 | 4595576871 | 0.90 |
| Deaths | Lithuania | 2021 | 0.56 | 0.66 | 0.46 | 0.86 | 1763517 | 5105255047 | 4599413832 | 881758.6 | 4598532074 | 0.90 |
| Deaths | United Kingdom | 2021 | 0.52 | 0.54 | 0.50 | 0.86 | 43558640 | 5105255047 | 4642972474 | 21779320 | 4621193153 | 0.91 |
| Deaths | Finland | 2021 | 0.57 | 0.67 | 0.48 | 0.86 | 3397926 | 5105255047 | 4646370400 | 1698963 | 4644671437 | 0.91 |
| Deaths | United States of America | 2021 | 0.47 | 0.49 | 0.45 | 0.86 | 216097500 | 5105255047 | 4862467891 | 108048700 | 4754419146 | 0.93 |
| Deaths | Switzerland | 1992 | 0.48 | 0.56 | 0.41 | 0.87 | 4755332 | 3376967464 | 3376967464 | 2377666 | 3374589798 | 1.00 |
| Deaths | Andorra | 2021 | 0.24 | 0.41 | 0.13 | 0.87 | 61742.59 | 5105255047 | 4862529634 | 30871.3 | 4862498763 | 0.95 |
| Deaths | Japan | 2021 | 0.20 | 0.21 | 0.19 | 0.87 | 75404150 | 5105255047 | 4937933788 | 37702080 | 4900231711 | 0.96 |
| Deaths | Canada | 2021 | 0.32 | 0.37 | 0.27 | 0.87 | 24262340 | 5105255047 | 4962196124 | 12131170 | 4950064956 | 0.97 |
| Deaths | Ireland | 2021 | 0.34 | 0.41 | 0.28 | 0.87 | 3192765 | 5105255047 | 4965388890 | 1596383 | 4963792507 | 0.97 |
| Deaths | Taiwan (Province of China) | 2021 | 0.20 | 0.24 | 0.17 | 0.87 | 16681750 | 5105255047 | 4982070642 | 8340876 | 4973729766 | 0.97 |
| Deaths | Iceland | 2021 | 0.58 | 0.69 | 0.48 | 0.88 | 227618.6 | 5105255047 | 4982298260 | 113809.3 | 4982184451 | 0.98 |
| Deaths | Luxembourg | 2021 | 0.58 | 0.68 | 0.49 | 0.88 | 447039.5 | 5105255047 | 4982745300 | 223519.8 | 4982521780 | 0.98 |
| Deaths | Republic of Korea | 2021 | 0.10 | 0.14 | 0.07 | 0.89 | 36999740 | 5105255047 | 5019745039 | 18499870 | 5001245169 | 0.98 |
| Deaths | Sweden | 2021 | 0.28 | 0.34 | 0.23 | 0.89 | 6417083 | 5105255047 | 5026162122 | 3208541 | 5022953580 | 0.98 |
| Deaths | San Marino | 2021 | 0.13 | 0.22 | 0.07 | 0.89 | 21290.2 | 5105255047 | 5026183412 | 10645.1 | 5026172767 | 0.98 |
| Deaths | Netherlands | 2021 | 0.41 | 0.48 | 0.35 | 0.89 | 11069920 | 5105255047 | 5037253336 | 5534962 | 5031718374 | 0.99 |
| Deaths | Denmark | 2021 | 0.48 | 0.56 | 0.41 | 0.90 | 3719018 | 5105255047 | 5040972354 | 1859509 | 5039112845 | 0.99 |

| | | | | | | | | | | | |
|---|---|---|---|---|---|---|---|---|---|---|---|
| Deaths | Germany | 2021 | 0.60 | 0.71 | 0.51 | 0.90 | 54851780 | 5105255047 | 5095824131 | 27425890 | 5068398243 | 0.99 |
| Deaths | Monaco | 2021 | 0.19 | 0.31 | 0.11 | 0.91 | 23218.3 | 5105255047 | 5095847350 | 11609.15 | 5095835740 | 1.00 |
| Deaths | Norway | 2021 | 0.40 | 0.43 | 0.37 | 0.92 | 3522494 | 5105255047 | 5099369844 | 1761247 | 5097608597 | 1.00 |
| Deaths | Switzerland | 2021 | 0.29 | 0.33 | 0.24 | 0.93 | 5885204 | 5105255047 | 5105255047 | 2942602 | 5102312446 | 1.00 |
| Deaths | Somalia | 1992 | 0.22 | 0.46 | 0.08 | 0.05 | 3816581 | 3376967464 | 3816581 | 1908290 | 1908290.3 | 0.00 |
| Deaths | Somalia | 2021 | 0.15 | 0.31 | 0.06 | 0.08 | 10890630 | 5105255047 | 10890631 | 5445315 | 5445315.5 | 0.00 |
| Deaths | Niger | 1992 | 0.13 | 0.25 | 0.05 | 0.08 | 4059013 | 3376967464 | 7875594 | 2029507 | 5846087.2 | 0.00 |
| Deaths | Chad | 1992 | 0.15 | 0.29 | 0.07 | 0.12 | 3037794 | 3376967464 | 10913388 | 1518897 | 9394490.9 | 0.00 |
| Deaths | Mali | 1992 | 0.17 | 0.32 | 0.07 | 0.13 | 4416030 | 3376967464 | 15329418 | 2208015 | 13121403 | 0.00 |
| Deaths | Burkina Faso | 1992 | 0.18 | 0.34 | 0.08 | 0.14 | 4697926 | 3376967464 | 20027344 | 2348963 | 17678381.3 | 0.01 |
| Deaths | Ethiopia | 1992 | 0.20 | 0.36 | 0.10 | 0.15 | 27028040 | 3376967464 | 47055389 | 13514020 | 33541366.7 | 0.01 |
| Deaths | Niger | 2021 | 0.12 | 0.23 | 0.04 | 0.17 | 11701660 | 5105255047 | 22592286 | 5850828 | 16741458.7 | 0.00 |
| Deaths | Mozambique | 1992 | 0.26 | 0.45 | 0.12 | 0.18 | 6841201 | 3376967464 | 53896590 | 3420600 | 50475989.3 | 0.01 |
| Deaths | Afghanistan | 1992 | 0.37 | 0.96 | 0.08 | 0.18 | 5317936 | 3376967464 | 59214526 | 2658968 | 56555557.9 | 0.02 |
| Deaths | Guinea | 1992 | 0.19 | 0.36 | 0.09 | 0.18 | 3194335 | 3376967464 | 62408861 | 1597167 | 60811693.6 | 0.02 |
| Deaths | Uganda | 1992 | 0.21 | 0.40 | 0.09 | 0.19 | 8871780 | 3376967464 | 71280641 | 4435890 | 66844751.1 | 0.02 |
| Deaths | Malawi | 1992 | 0.20 | 0.35 | 0.10 | 0.21 | 5373904 | 3376967464 | 76654545 | 2686952 | 73967593.3 | 0.02 |
| Deaths | Burundi | 1992 | 0.35 | 0.68 | 0.15 | 0.21 | 2868701 | 3376967464 | 79523246 | 1434350 | 78088895.8 | 0.02 |
| Deaths | South Sudan | 1992 | 0.22 | 0.43 | 0.10 | 0.21 | 3170246 | 3376967464 | 82693492 | 1585123 | 81108369 | 0.02 |
| Deaths | Nepal | 1992 | 0.16 | 0.29 | 0.07 | 0.21 | 10843820 | 3376967464 | 93537312 | 5421910 | 88115402.1 | 0.03 |
| Deaths | Sierra Leone | 1992 | 0.19 | 0.35 | 0.08 | 0.21 | 2160724 | 3376967464 | 95698036 | 1080362 | 94617674.1 | 0.03 |
| Deaths | Guinea-Bissau | 1992 | 0.28 | 0.53 | 0.12 | 0.22 | 518730.4 | 3376967464 | 96216766 | 259365.2 | 95957401.1 | 0.03 |
| Deaths | Central African Republic | 1992 | 0.35 | 0.68 | 0.15 | 0.22 | 1511635 | 3376967464 | 97728401 | 755817.3 | 96972583.7 | 0.03 |
| Deaths | Benin | 1992 | 0.14 | 0.25 | 0.07 | 0.23 | 2437709 | 3376967464 | 100166110 | 1218855 | 98947255.7 | 0.03 |

| | | | | | | | | | | | | |
|---|---|---|---|---|---|---|---|---|---|---|---|---|
| Deaths | Bhutan | 1992 | 0.14 | 0.27 | 0.06 | 0.23 | 362111 | 3376967464 | 100528221 | 181055.5 | 100347165.9 | 0.03 |
| Deaths | Eritrea | 1992 | 0.30 | 0.57 | 0.13 | 0.23 | 1636011 | 3376967464 | 102164232 | 818005.3 | 101346226.7 | 0.03 |
| Deaths | Yemen | 1992 | 0.25 | 0.57 | 0.06 | 0.23 | 6649314 | 3376967464 | 108813546 | 3324657 | 105488888.8 | 0.03 |
| Deaths | Liberia | 1992 | 0.23 | 0.40 | 0.11 | 0.24 | 970267.7 | 3376967464 | 109783813 | 485133.8 | 109298679.5 | 0.03 |
| Deaths | Chad | 2021 | 0.16 | 0.30 | 0.07 | 0.24 | 8325851 | 5105255047 | 30918137 | 4162925 | 26755211.9 | 0.01 |
| Deaths | Bangladesh | 1992 | 0.16 | 0.30 | 0.08 | 0.25 | 59911290 | 3376967464 | 169695106 | 29955650 | 139739459.7 | 0.04 |
| Deaths | Senegal | 1992 | 0.21 | 0.37 | 0.10 | 0.25 | 3982520 | 3376967464 | 173677626 | 1991260 | 171686366 | 0.05 |
| Deaths | Gambia | 1992 | 0.19 | 0.35 | 0.08 | 0.25 | 540094.8 | 3376967464 | 174217721 | 270047.4 | 173947673.4 | 0.05 |
| Deaths | United Republic of Tanzania | 1992 | 0.25 | 0.43 | 0.13 | 0.26 | 13815680 | 3376967464 | 188033398 | 6907839 | 181125559.4 | 0.05 |
| Deaths | Mali | 2021 | 0.14 | 0.27 | 0.06 | 0.27 | 11895330 | 5105255047 | 42813466 | 5947664 | 36865801.5 | 0.01 |
| Deaths | Lao People's Democratic Republic | 1992 | 0.03 | 0.09 | 0.01 | 0.27 | 2290031 | 3376967464 | 190323429 | 1145016 | 189178413.7 | 0.06 |
| Deaths | Rwanda | 1992 | 0.44 | 0.85 | 0.19 | 0.28 | 3779329 | 3376967464 | 194102758 | 1889664 | 192213093.7 | 0.06 |
| Deaths | South Sudan | 2021 | 0.22 | 0.40 | 0.10 | 0.28 | 5135653 | 5105255047 | 47949119 | 2567827 | 45381292.2 | 0.01 |
| Deaths | Togo | 1992 | 0.20 | 0.35 | 0.10 | 0.28 | 1939975 | 3376967464 | 196042733 | 969987.5 | 195072745.7 | 0.06 |
| Deaths | Angola | 1992 | 0.29 | 0.53 | 0.15 | 0.28 | 5702682 | 3376967464 | 201745415 | 2851341 | 198894074.2 | 0.06 |
| Deaths | Timor-Leste | 1992 | 0.02 | 0.05 | 0.01 | 0.28 | 447494.9 | 3376967464 | 202192910 | 223747.4 | 201969162.6 | 0.06 |
| Deaths | Madagascar | 1992 | 0.46 | 0.82 | 0.24 | 0.28 | 6432226 | 3376967464 | 208625136 | 3216113 | 205409023.2 | 0.06 |
| Deaths | Equatorial Guinea | 1992 | 0.35 | 0.63 | 0.17 | 0.28 | 229117.5 | 3376967464 | 208854254 | 114558.8 | 208739695 | 0.06 |
| Deaths | Burkina Faso | 2021 | 0.18 | 0.33 | 0.08 | 0.29 | 11698660 | 5105255047 | 59647781 | 5849331 | 53798449.8 | 0.01 |
| Deaths | Cabo Verde | 1992 | 0.22 | 0.45 | 0.10 | 0.29 | 183931.3 | 3376967464 | 209038185 | 91965.67 | 208946219.4 | 0.06 |
| Deaths | Comoros | 1992 | 0.31 | 0.54 | 0.15 | 0.29 | 249116.1 | 3376967464 | 209287301 | 124558 | 209162743.1 | 0.06 |
| Deaths | Burundi | 2021 | 0.21 | 0.36 | 0.10 | 0.29 | 7040681 | 5105255047 | 66688462 | 3520341 | 63168121.4 | 0.01 |
| Deaths | Democratic Republic of the | 1992 | 0.27 | 0.46 | 0.13 | 0.29 | 20457730 | 3376967464 | 229745032 | 10228870 | 219516166.6 | 0.07 |

| | | | | | | | | | | | | |
|---|---|---|---|---|---|---|---|---|---|---|---|---|
| | Congo | | | | | | | | | | | |
| Deaths | Coted'Ivoire | 1992 | 0.21 | 0.38 | 0.10 | 0.29 | 6800901 | 3376967464 | 236545933 | 3400450 | 233145482.4 | 0.07 |
| Deaths | Cambodia | 1992 | 0.02 | 0.06 | 0.01 | 0.30 | 5566488 | 3376967464 | 242112421 | 2783244 | 239329176.7 | 0.07 |
| Deaths | Sudan | 1992 | 0.29 | 0.62 | 0.07 | 0.30 | 11202790 | 3376967464 | 253315211 | 5601395 | 247713815.6 | 0.07 |
| Deaths | Zambia | 1992 | 0.26 | 0.47 | 0.13 | 0.31 | 4197849 | 3376967464 | 257513060 | 2098925 | 255414135.1 | 0.08 |
| Deaths | Central African Republic | 2021 | 0.33 | 0.62 | 0.16 | 0.31 | 3075108 | 5105255047 | 69763570 | 1537554 | 68226015.8 | 0.01 |
| Deaths | Nigeria | 1992 | 0.20 | 0.31 | 0.11 | 0.31 | 50478270 | 3376967464 | 307991334 | 25239140 | 282752197 | 0.08 |
| Deaths | Solomon Islands | 1992 | 0.25 | 0.48 | 0.11 | 0.31 | 187733.3 | 3376967464 | 308179068 | 93866.66 | 308085200.9 | 0.09 |
| Deaths | Sao Tome and Principe | 1992 | 0.18 | 0.34 | 0.09 | 0.31 | 61668.72 | 3376967464 | 308240736 | 30834.36 | 308209902 | 0.09 |
| Deaths | Cameroon | 1992 | 0.24 | 0.42 | 0.12 | 0.32 | 5631620 | 3376967464 | 313872356 | 2815810 | 311056546.2 | 0.09 |
| Deaths | Guatemala | 1992 | 0.18 | 0.23 | 0.13 | 0.32 | 4330417 | 3376967464 | 318202773 | 2165209 | 316037564.7 | 0.09 |
| Deaths | Papua New Guinea | 1992 | 0.37 | 0.74 | 0.15 | 0.32 | 2419850 | 3376967464 | 320622623 | 1209925 | 319412698.1 | 0.09 |
| Deaths | Haiti | 1992 | 0.47 | 0.86 | 0.20 | 0.32 | 3615033 | 3376967464 | 324237656 | 1807516 | 322430139.3 | 0.10 |
| Deaths | Pakistan | 1992 | 0.17 | 0.28 | 0.09 | 0.32 | 60357000 | 3376967464 | 384594651 | 30178500 | 354416153.3 | 0.10 |
| Deaths | Myanmar | 1992 | 0.04 | 0.09 | 0.01 | 0.33 | 24926270 | 3376967464 | 409520920 | 12463130 | 397057785.4 | 0.12 |
| Deaths | Mozambique | 2021 | 0.36 | 0.64 | 0.18 | 0.33 | 16039550 | 5105255047 | 85803118 | 8019774 | 77783343.8 | 0.02 |
| Deaths | Guinea | 2021 | 0.19 | 0.36 | 0.09 | 0.34 | 6958615 | 5105255047 | 92761733 | 3479307 | 89282425.3 | 0.02 |
| Deaths | Afghanistan | 2021 | 0.25 | 0.57 | 0.07 | 0.34 | 16398400 | 5105255047 | 109160135 | 8199201 | 100960934 | 0.02 |
| Deaths | Djibouti | 1992 | 0.22 | 0.39 | 0.11 | 0.34 | 281064.1 | 3376967464 | 409801984 | 140532.1 | 409661452 | 0.12 |
| Deaths | Honduras | 1992 | 0.29 | 0.45 | 0.17 | 0.34 | 2511178 | 3376967464 | 412313162 | 1255589 | 411057573.1 | 0.12 |
| Deaths | India | 1992 | 0.19 | 0.29 | 0.12 | 0.34 | 515839400 | 3376967464 | 928152513 | 257919700 | 670232837.4 | 0.20 |
| Deaths | Kenya | 1992 | 0.14 | 0.21 | 0.09 | 0.35 | 12235470 | 3376967464 | 940387986 | 6117737 | 934270249.4 | 0.28 |
| Deaths | Mauritania | 1992 | 0.24 | 0.42 | 0.13 | 0.35 | 1105278 | 3376967464 | 941493264 | 552639.1 | 940940625.2 | 0.28 |
| Deaths | Lesotho | 1992 | 0.16 | 0.28 | 0.07 | 0.35 | 821010.8 | 3376967464 | 942314275 | 410505.4 | 941903769.7 | 0.28 |

| | | | | | | | | | | | | |
|---|---|---|---|---|---|---|---|---|---|---|---|---|
| Deaths | Liberia | 2021 | 0.20 | 0.37 | 0.09 | 0.35 | 3135229 | 5105255047 | 112295364 | 1567614 | 110727749.6 | 0.02 |
| Deaths | Guinea-Bissau | 2021 | 0.27 | 0.49 | 0.13 | 0.35 | 1119372 | 5105255047 | 113414736 | 559686.2 | 112855050.1 | 0.02 |
| Deaths | Nicaragua | 1992 | 0.10 | 0.14 | 0.07 | 0.36 | 2103741 | 3376967464 | 944418017 | 1051871 | 943366145.8 | 0.28 |
| Deaths | Maldives | 1992 | 0.11 | 0.20 | 0.05 | 0.36 | 118284.3 | 3376967464 | 944536301 | 59142.14 | 944477158.7 | 0.28 |
| Deaths | Sierra Leone | 2021 | 0.19 | 0.39 | 0.09 | 0.36 | 5014942 | 5105255047 | 118429679 | 2507471 | 115922207.5 | 0.02 |
| Deaths | Ethiopia | 2021 | 0.13 | 0.20 | 0.08 | 0.36 | 61363610 | 5105255047 | 179793292 | 30681810 | 149111485.3 | 0.03 |
| Deaths | Vanuatu | 1992 | 0.44 | 0.84 | 0.21 | 0.36 | 84103.98 | 3376967464 | 944620405 | 42051.99 | 944578352.8 | 0.28 |
| Deaths | Morocco | 1992 | 0.30 | 0.58 | 0.08 | 0.37 | 15160090 | 3376967464 | 959780492 | 7580044 | 952200448.5 | 0.28 |
| Deaths | Benin | 2021 | 0.14 | 0.25 | 0.07 | 0.37 | 7048559 | 5105255047 | 186841851 | 3524280 | 183317571.5 | 0.04 |
| Deaths | El Salvador | 1992 | 0.11 | 0.16 | 0.08 | 0.38 | 3000660 | 3376967464 | 962781152 | 1500330 | 961280822.3 | 0.28 |
| Deaths | Democratic Republic of the Congo | 2021 | 0.27 | 0.46 | 0.14 | 0.38 | 49675100 | 5105255047 | 236516952 | 24837550 | 211679401.7 | 0.04 |
| Deaths | Malawi | 2021 | 0.26 | 0.44 | 0.14 | 0.38 | 10785330 | 5105255047 | 247302286 | 5392667 | 241909619.1 | 0.05 |
| Deaths | Ghana | 1992 | 0.30 | 0.50 | 0.16 | 0.38 | 8290370 | 3376967464 | 971071522 | 4145185 | 966926337.2 | 0.29 |
| Deaths | Madagascar | 2021 | 0.46 | 0.76 | 0.24 | 0.40 | 16136660 | 5105255047 | 263438948 | 8068331 | 255370616.9 | 0.05 |
| Deaths | Eritrea | 2021 | 0.27 | 0.47 | 0.14 | 0.40 | 3903951 | 5105255047 | 267342899 | 1951975 | 265390923.2 | 0.05 |
| Deaths | Senegal | 2021 | 0.21 | 0.38 | 0.10 | 0.41 | 8916317 | 5105255047 | 276259216 | 4458159 | 271801057.2 | 0.05 |
| Deaths | Togo | 2021 | 0.24 | 0.43 | 0.12 | 0.41 | 4808399 | 5105255047 | 281067615 | 2404199 | 278663415.3 | 0.05 |
| Deaths | Gambia | 2021 | 0.23 | 0.40 | 0.11 | 0.41 | 1328594 | 5105255047 | 282396209 | 664296.9 | 281731911.6 | 0.06 |
| Deaths | Palestine | 1992 | 0.15 | 0.25 | 0.08 | 0.41 | 1092733 | 3376967464 | 972164255 | 546366.4 | 971617888.3 | 0.29 |
| Deaths | Zimbabwe | 1992 | 0.15 | 0.29 | 0.07 | 0.41 | 5556213 | 3376967464 | 977720467 | 2778106 | 974942361.1 | 0.29 |
| Deaths | Kiribati | 1992 | 1.15 | 1.86 | 0.64 | 0.42 | 43348.47 | 3376967464 | 977763816 | 21674.24 | 977742141.7 | 0.29 |
| Deaths | Eswatini | 1992 | 0.21 | 0.39 | 0.09 | 0.42 | 429553.2 | 3376967464 | 978193369 | 214776.6 | 977978592.6 | 0.29 |
| Deaths | Papua New Guinea | 2021 | 0.37 | 0.71 | 0.18 | 0.42 | 6230283 | 5105255047 | 288626492 | 3115142 | 285511350.2 | 0.06 |

| | | | | | | | | | | | | |
|---|---|---|---|---|---|---|---|---|---|---|---|---|
| Deaths | Viet Nam | 1992 | 0.02 | 0.05 | 0.01 | 0.42 | 40268370 | 3376967464 | 1018461738 | 20134180 | 998327553.6 | 0.30 |
| Deaths | Iraq | 1992 | 0.42 | 0.70 | 0.21 | 0.42 | 10043140 | 3376967464 | 1028504876 | 5021569 | 1023483307 | 0.30 |
| Deaths | Tuvalu | 1992 | 0.67 | 1.21 | 0.34 | 0.42 | 5474.875 | 3376967464 | 1028510350 | 2737.437 | 1028507613 | 0.30 |
| Deaths | Uganda | 2021 | 0.18 | 0.32 | 0.09 | 0.42 | 22465620 | 5105255047 | 311092115 | 11232810 | 299859303.5 | 0.06 |
| Deaths | Coted'Ivoire | 2021 | 0.22 | 0.41 | 0.11 | 0.43 | 15562160 | 5105255047 | 326654276 | 7781080 | 318873195.4 | 0.06 |
| Deaths | Solomon Islands | 2021 | 0.28 | 0.51 | 0.14 | 0.43 | 400945.3 | 5105255047 | 327055221 | 200472.7 | 326854748.6 | 0.06 |
| Deaths | Congo | 1992 | 0.41 | 0.73 | 0.21 | 0.43 | 1365818 | 3376967464 | 1029876169 | 682909 | 1029193260 | 0.30 |
| Deaths | Nepal | 2021 | 0.22 | 0.35 | 0.12 | 0.43 | 19987970 | 5105255047 | 347043194 | 9993986 | 337049207.6 | 0.07 |
| Deaths | Bolivia (Plurinational State of) | 1992 | 0.40 | 0.64 | 0.22 | 0.43 | 3645533 | 3376967464 | 1033521701 | 1822766 | 1031698935 | 0.31 |
| Deaths | Rwanda | 2021 | 0.23 | 0.40 | 0.12 | 0.44 | 7848418 | 5105255047 | 354891612 | 3924209 | 350967402.9 | 0.07 |
| Deaths | Egypt | 1992 | 0.39 | 0.65 | 0.21 | 0.44 | 33112790 | 3376967464 | 1066634488 | 16556390 | 1050078095 | 0.31 |
| Deaths | Botswana | 1992 | 0.24 | 0.43 | 0.12 | 0.44 | 747651.5 | 3376967464 | 1067382140 | 373825.7 | 1067008314 | 0.32 |
| Deaths | Marshall Islands | 1992 | 0.52 | 0.97 | 0.23 | 0.44 | 23628.24 | 3376967464 | 1067405768 | 11814.12 | 1067393954 | 0.32 |
| Deaths | Belize | 1992 | 0.09 | 0.13 | 0.07 | 0.44 | 103326.3 | 3376967464 | 1067509094 | 51663.13 | 1067457431 | 0.32 |
| Deaths | Timor-Leste | 2021 | 0.02 | 0.06 | 0.01 | 0.44 | 802737.4 | 5105255047 | 355694349 | 401368.7 | 355292980.6 | 0.07 |
| Deaths | Syrian Arab Republic | 1992 | 0.95 | 1.57 | 0.50 | 0.45 | 6931728 | 3376967464 | 1074440822 | 3465864 | 1070974958 | 0.32 |
| Deaths | United Republic of Tanzania | 2021 | 0.25 | 0.46 | 0.13 | 0.45 | 32205890 | 5105255047 | 387900240 | 16102950 | 371797294.8 | 0.07 |
| Deaths | Haiti | 2021 | 0.48 | 0.85 | 0.23 | 0.45 | 8005224 | 5105255047 | 395905464 | 4002612 | 391902852.4 | 0.08 |
| Deaths | Dominican Republic | 1992 | 0.28 | 0.42 | 0.18 | 0.45 | 4367623 | 3376967464 | 1078808445 | 2183812 | 1076624634 | 0.32 |
| Deaths | Yemen | 2021 | 0.25 | 0.51 | 0.06 | 0.45 | 18839690 | 5105255047 | 414745156 | 9419846 | 405325310.2 | 0.08 |
| Deaths | Angola | 2021 | 0.30 | 0.51 | 0.16 | 0.45 | 16719890 | 5105255047 | 431465050 | 8359947 | 423105102.9 | 0.08 |
| Deaths | Grenada | 1992 | 0.38 | 0.51 | 0.27 | 0.46 | 48427.92 | 3376967464 | 1078856873 | 24213.96 | 1078832659 | 0.32 |
| Deaths | Oman | 1992 | 0.11 | 0.21 | 0.05 | 0.46 | 1153630 | 3376967464 | 1080010503 | 576815.1 | 1079433688 | 0.32 |
| Deaths | Namibia | 1992 | 0.21 | 0.35 | 0.11 | 0.46 | 804903.9 | 3376967464 | 1080815407 | 402451.9 | 1080412955 | 0.32 |

| | | | | | | | | | | | | |
|---|---|---|---|---|---|---|---|---|---|---|---|---|
| Deaths | Guyana | 1992 | 0.62 | 0.78 | 0.47 | 0.47 | 459165.3 | 3376967464 | 1081274573 | 229582.7 | 1081044990 | 0.32 |
| Deaths | Gabon | 1992 | 0.40 | 0.65 | 0.22 | 0.47 | 557425.5 | 3376967464 | 1081831998 | 278712.8 | 1081553285 | 0.32 |
| Deaths | Bhutan | 2021 | 0.20 | 0.34 | 0.11 | 0.47 | 519639.6 | 5105255047 | 431984689 | 259819.8 | 431724869.5 | 0.08 |
| Deaths | Vanuatu | 2021 | 0.45 | 0.77 | 0.23 | 0.47 | 184213.1 | 5105255047 | 432168902 | 92106.55 | 432076795.9 | 0.08 |
| Deaths | Cambodia | 2021 | 0.02 | 0.06 | 0.01 | 0.47 | 10996940 | 5105255047 | 443165844 | 5498471 | 437667373 | 0.09 |
| Deaths | Micronesia (Federated States of) | 1992 | 0.58 | 1.02 | 0.30 | 0.47 | 55679.86 | 3376967464 | 1081887678 | 27839.93 | 1081859838 | 0.32 |
| Deaths | Zimbabwe | 2021 | 0.31 | 0.57 | 0.16 | 0.47 | 8807361 | 5105255047 | 451973205 | 4403681 | 447569524.4 | 0.09 |
| Deaths | Tajikistan | 1992 | 0.00 | 0.01 | 0.00 | 0.48 | 2943035 | 3376967464 | 1084830713 | 1471517 | 1083359195 | 0.32 |
| Deaths | Indonesia | 1992 | 0.03 | 0.07 | 0.01 | 0.48 | 115716600 | 3376967464 | 1200547263 | 57858280 | 1142688988 | 0.34 |
| Deaths | China | 1992 | 0.04 | 0.06 | 0.02 | 0.48 | 815239200 | 3376967464 | 2015786424 | 407619600 | 1608166844 | 0.48 |
| Deaths | Algeria | 1992 | 0.26 | 0.51 | 0.07 | 0.48 | 14528470 | 3376967464 | 2030314898 | 7264237 | 2023050661 | 0.60 |
| Deaths | Comoros | 2021 | 0.27 | 0.47 | 0.14 | 0.48 | 467233.1 | 5105255047 | 452440438 | 233616.6 | 452206821.7 | 0.09 |
| Deaths | Turkey | 1992 | 0.53 | 0.90 | 0.26 | 0.48 | 36334360 | 3376967464 | 2066649254 | 18167180 | 2048482076 | 0.61 |
| Deaths | Mongolia | 1992 | 0.04 | 0.08 | 0.02 | 0.48 | 1235917 | 3376967464 | 2067885171 | 617958.5 | 2067267212 | 0.61 |
| Deaths | Paraguay | 1992 | 0.53 | 0.77 | 0.35 | 0.48 | 2324234 | 3376967464 | 2070209405 | 1162117 | 2069047288 | 0.61 |
| Deaths | Cameroon | 2021 | 0.23 | 0.40 | 0.12 | 0.48 | 17450670 | 5105255047 | 469891104 | 8725333 | 461165771 | 0.09 |
| Deaths | Iran (Islamic Republic of) | 1992 | 0.29 | 0.41 | 0.20 | 0.48 | 32234900 | 3376967464 | 2102444303 | 16117450 | 2086326854 | 0.62 |
| Deaths | Colombia | 1992 | 0.65 | 0.75 | 0.56 | 0.48 | 20427380 | 3376967464 | 2122871685 | 10213690 | 2112657994 | 0.63 |
| Deaths | Saint Vincent and the Grenadines | 1992 | 0.52 | 0.64 | 0.42 | 0.49 | 63082.62 | 3376967464 | 2122934768 | 31541.31 | 2122903226 | 0.63 |
| Deaths | Djibouti | 2021 | 0.25 | 0.46 | 0.12 | 0.49 | 805751.6 | 5105255047 | 470696855 | 402875.8 | 470293979.5 | 0.09 |
| Deaths | Tunisia | 1992 | 0.27 | 0.51 | 0.07 | 0.49 | 5133459 | 3376967464 | 2128068226 | 2566729 | 2125501497 | 0.63 |
| Deaths | Lao People's Democratic Republic | 2021 | 0.03 | 0.08 | 0.01 | 0.49 | 4754006 | 5105255047 | 475450861 | 2377003 | 473073858.3 | 0.09 |
| Deaths | Bangladesh | 2021 | 0.22 | 0.38 | 0.12 | 0.49 | 107272700 | 5105255047 | 582723583 | 53636360 | 529087222.1 | 0.10 |

| | | | | | | | | | | | | | |
|---|---|---|---|---|---|---|---|---|---|---|---|---|---|
| Deaths | Samoa | 1992 | 0.42 | 0.71 | 0.23 | 0.49 | 92361.26 | 3376967464 | 2128160588 | 46180.63 | 2128114407 | 0.63 |
| Deaths | Mauritania | 2021 | 0.20 | 0.35 | 0.10 | 0.50 | 2374531 | 5105255047 | 585098113 | 1187265 | 583910848.2 | 0.11 |
| Deaths | Democratic People's Republic of Korea | 1992 | 0.06 | 0.11 | 0.02 | 0.50 | 13875620 | 3376967464 | 2142036206 | 6937809 | 2135098397 | 0.63 |
| Deaths | Uzbekistan | 1992 | 0.03 | 0.05 | 0.02 | 0.50 | 12096580 | 3376967464 | 2154132781 | 6048288 | 2148084494 | 0.64 |
| Deaths | Nigeria | 2021 | 0.16 | 0.30 | 0.08 | 0.50 | 123377300 | 5105255047 | 708475425 | 61688660 | 646786769.3 | 0.13 |
| Deaths | Tonga | 1992 | 0.28 | 0.48 | 0.16 | 0.50 | 53374.01 | 3376967464 | 2154186155 | 26687 | 2154159468 | 0.64 |
| Deaths | Pakistan | 2021 | 0.24 | 0.36 | 0.15 | 0.50 | 141565600 | 5105255047 | 850041000 | 70782790 | 779258212.8 | 0.15 |
| Deaths | Sao Tome and Principe | 2021 | 0.23 | 0.47 | 0.11 | 0.51 | 131024.1 | 5105255047 | 850172025 | 65512.04 | 850106512.5 | 0.17 |
| Deaths | Zambia | 2021 | 0.28 | 0.48 | 0.15 | 0.51 | 10789070 | 5105255047 | 860961092 | 5394534 | 855566558.4 | 0.17 |
| Deaths | Brazil | 1992 | 0.75 | 0.79 | 0.71 | 0.51 | 93825900 | 3376967464 | 2248012056 | 46912950 | 2201099106 | 0.65 |
| Deaths | Lesotho | 2021 | 0.28 | 0.50 | 0.14 | 0.51 | 1159988 | 5105255047 | 862121080 | 579994.1 | 861541086.2 | 0.17 |
| Deaths | Suriname | 1992 | 0.35 | 0.54 | 0.21 | 0.51 | 240092.4 | 3376967464 | 2248252148 | 120046.2 | 2248132102 | 0.67 |
| Deaths | Honduras | 2021 | 0.35 | 0.54 | 0.21 | 0.51 | 6325127 | 5105255047 | 868446208 | 3162564 | 865283644 | 0.17 |
| Deaths | Peru | 1992 | 0.23 | 0.35 | 0.15 | 0.51 | 13017280 | 3376967464 | 2261269426 | 6508639 | 2254760787 | 0.67 |
| Deaths | Mexico | 1992 | 0.30 | 0.31 | 0.28 | 0.51 | 51527500 | 3376967464 | 2312796928 | 25763750 | 2287033177 | 0.68 |
| Deaths | Saint Lucia | 1992 | 0.46 | 0.55 | 0.38 | 0.52 | 80360.32 | 3376967464 | 2312877288 | 40180.16 | 2312837108 | 0.68 |
| Deaths | Philippines | 1992 | 0.03 | 0.04 | 0.02 | 0.52 | 37716100 | 3376967464 | 2350593387 | 18858050 | 2331735338 | 0.69 |
| Deaths | Ecuador | 1992 | 0.28 | 0.32 | 0.23 | 0.52 | 6008273 | 3376967464 | 2356601661 | 3004137 | 2353597524 | 0.70 |
| Deaths | Kenya | 2021 | 0.18 | 0.25 | 0.13 | 0.52 | 29742370 | 5105255047 | 898188583 | 14871190 | 883317395.1 | 0.17 |
| Deaths | Nicaragua | 2021 | 0.14 | 0.19 | 0.09 | 0.52 | 4297839 | 5105255047 | 902486421 | 2148919 | 900337502 | 0.18 |
| Deaths | Thailand | 1992 | 0.07 | 0.12 | 0.04 | 0.53 | 39057400 | 3376967464 | 2395659056 | 19528700 | 2376130359 | 0.70 |
| Deaths | Kiribati | 2021 | 1.29 | 2.18 | 0.70 | 0.53 | 74513.75 | 5105255047 | 902560935 | 37256.87 | 902523678.2 | 0.18 |
| Deaths | Kyrgyzstan | 1992 | 0.02 | 0.02 | 0.01 | 0.53 | 2635720 | 3376967464 | 2398294776 | 1317860 | 2396976916 | 0.71 |

| | | | | | | | | | | | | |
|---|---|---|---|---|---|---|---|---|---|---|---|---|
| Deaths | Venezuela (Bolivarian Republic of) | 1992 | 0.39 | 0.45 | 0.34 | 0.53 | 11607490 | 3376967464 | 2409902266 | 5803745 | 2404098521 | 0.71 |
| Deaths | Tokelau | 1992 | 0.59 | 1.06 | 0.30 | 0.53 | 834.3087 | 3376967464 | 2409903100 | 417.1543 | 2409902683 | 0.71 |
| Deaths | Sri Lanka | 1992 | 0.19 | 0.27 | 0.13 | 0.53 | 11121210 | 3376967464 | 2421024309 | 5560605 | 2415463705 | 0.72 |
| Deaths | Cabo Verde | 2021 | 0.25 | 0.48 | 0.13 | 0.53 | 382357.4 | 5105255047 | 902943292 | 191178.7 | 902752113.8 | 0.18 |
| Deaths | Myanmar | 2021 | 0.04 | 0.10 | 0.01 | 0.53 | 36996570 | 5105255047 | 939939864 | 18498290 | 921441578.5 | 0.18 |
| Deaths | Nauru | 1992 | 0.86 | 1.54 | 0.41 | 0.54 | 5770.117 | 3376967464 | 2421030079 | 2885.058 | 2421027194 | 0.72 |
| Deaths | Bosnia and Herzegovina | 1992 | 0.32 | 0.56 | 0.16 | 0.54 | 3112775 | 3376967464 | 2424142855 | 1556388 | 2422586467 | 0.72 |
| Deaths | Guatemala | 2021 | 0.17 | 0.22 | 0.14 | 0.54 | 9913414 | 5105255047 | 949853279 | 4956707 | 944896571.5 | 0.19 |
| Deaths | Tajikistan | 2021 | 0.00 | 0.00 | 0.00 | 0.54 | 6207893 | 5105255047 | 956061172 | 3103947 | 952957225.2 | 0.19 |
| Deaths | Lebanon | 1992 | 1.43 | 2.43 | 0.68 | 0.54 | 1853583 | 3376967464 | 2425996438 | 926791.5 | 2425069646 | 0.72 |
| Deaths | Sudan | 2021 | 0.28 | 0.54 | 0.08 | 0.54 | 25440510 | 5105255047 | 981501682 | 12720260 | 968781427 | 0.19 |
| Deaths | Fiji | 1992 | 0.15 | 0.23 | 0.09 | 0.54 | 465040.9 | 3376967464 | 2426461479 | 232520.5 | 2426228958 | 0.72 |
| Deaths | Costa Rica | 1992 | 0.44 | 0.51 | 0.38 | 0.54 | 1893843 | 3376967464 | 2428355322 | 946921.6 | 2427408400 | 0.72 |
| Deaths | Jamaica | 1992 | 0.05 | 0.07 | 0.04 | 0.55 | 1395283 | 3376967464 | 2429750605 | 697641.5 | 2429052964 | 0.72 |
| Deaths | Jordan | 1992 | 0.26 | 0.38 | 0.16 | 0.55 | 2205439 | 3376967464 | 2431956044 | 1102720 | 2430853325 | 0.72 |
| Deaths | Armenia | 1992 | 0.07 | 0.08 | 0.05 | 0.55 | 2180135 | 3376967464 | 2434136179 | 1090067 | 2433046112 | 0.72 |
| Deaths | South Africa | 1992 | 0.35 | 0.48 | 0.25 | 0.55 | 22998760 | 3376967464 | 2457134939 | 11499380 | 2445635559 | 0.72 |
| Deaths | Albania | 1992 | 0.11 | 0.16 | 0.06 | 0.55 | 2048832 | 3376967464 | 2459183771 | 1024416 | 2458159355 | 0.73 |
| Deaths | Mauritius | 1992 | 0.53 | 0.61 | 0.45 | 0.55 | 729511 | 3376967464 | 2459913282 | 364755.5 | 2459548527 | 0.73 |
| Deaths | Libya | 1992 | 0.24 | 0.47 | 0.06 | 0.55 | 2388892 | 3376967464 | 2462302174 | 1194446 | 2461107728 | 0.73 |
| Deaths | Panama | 1992 | 0.23 | 0.27 | 0.20 | 0.55 | 1507409 | 3376967464 | 2463809583 | 753704.7 | 2463055878 | 0.73 |
| Deaths | Malaysia | 1992 | 0.13 | 0.20 | 0.09 | 0.56 | 11151630 | 3376967464 | 2474961212 | 5575814 | 2469385397 | 0.73 |
| Deaths | Saudi Arabia | 1992 | 0.18 | 0.31 | 0.09 | 0.56 | 9752944 | 3376967464 | 2484714156 | 4876472 | 2479837684 | 0.73 |

| | | | | | | | | | | | | |
|---|---|---|---|---|---|---|---|---|---|---|---|---|
| Deaths | Morocco | 2021 | 0.34 | 0.65 | 0.11 | 0.56 | 24647580 | 5105255047 | 1006149263 | 12323790 | 993825472.4 | 0.19 |
| Deaths | El Salvador | 2021 | 0.14 | 0.21 | 0.09 | 0.56 | 4074428 | 5105255047 | 1010223691 | 2037214 | 1008186477 | 0.20 |
| Deaths | Ghana | 2021 | 0.26 | 0.44 | 0.14 | 0.56 | 20161140 | 5105255047 | 1030384835 | 10080570 | 1020304263 | 0.20 |
| Deaths | Turkmenistan | 1992 | 0.01 | 0.01 | 0.01 | 0.57 | 2146841 | 3376967464 | 2486860997 | 1073420 | 2485787576 | 0.74 |
| Deaths | Cuba | 1992 | 0.47 | 0.55 | 0.39 | 0.57 | 7554381 | 3376967464 | 2494415377 | 3777190 | 2490638187 | 0.74 |
| Deaths | Dominica | 1992 | 0.29 | 0.45 | 0.18 | 0.57 | 41551.19 | 3376967464 | 2494456928 | 20775.6 | 2494436153 | 0.74 |
| Deaths | Democratic People's Republic of Korea | 2021 | 0.06 | 0.11 | 0.03 | 0.57 | 18946650 | 5105255047 | 1049331482 | 9473323 | 1039858159 | 0.20 |
| Deaths | Marshall Islands | 2021 | 0.62 | 1.13 | 0.30 | 0.57 | 36532.79 | 5105255047 | 1049368015 | 18266.39 | 1049349748 | 0.21 |
| Deaths | India | 2021 | 0.25 | 0.32 | 0.18 | 0.58 | 950604900 | 5105255047 | 1999972931 | 475302500 | 1524670473 | 0.30 |
| Deaths | Tuvalu | 2021 | 0.54 | 0.92 | 0.31 | 0.58 | 7770.842 | 5105255047 | 1999980701 | 3885.421 | 1999976816 | 0.39 |
| Deaths | Cook Islands | 1992 | 0.30 | 0.54 | 0.16 | 0.58 | 11553.59 | 3376967464 | 2494468482 | 5776.796 | 2494462705 | 0.74 |
| Deaths | Congo | 2021 | 0.38 | 0.65 | 0.21 | 0.58 | 3289800 | 5105255047 | 2003270502 | 1644900 | 2001625602 | 0.39 |
| Deaths | Eswatini | 2021 | 0.29 | 0.53 | 0.14 | 0.59 | 702572.3 | 5105255047 | 2003973074 | 351286.1 | 2003621788 | 0.39 |
| Deaths | Micronesia (Federated States of) | 2021 | 0.60 | 1.01 | 0.33 | 0.59 | 67172.81 | 5105255047 | 2004040247 | 33586.4 | 2004006661 | 0.39 |
| Deaths | Uruguay | 1992 | 1.19 | 1.36 | 1.03 | 0.59 | 1978724 | 3376967464 | 2496447206 | 989362.1 | 2495457844 | 0.74 |
| Deaths | Seychelles | 1992 | 0.18 | 0.29 | 0.08 | 0.59 | 45508.48 | 3376967464 | 2496492715 | 22754.24 | 2496469961 | 0.74 |
| Deaths | Samoa | 2021 | 0.45 | 0.75 | 0.25 | 0.59 | 122652 | 5105255047 | 2004162899 | 61325.99 | 2004101573 | 0.39 |
| Deaths | Azerbaijan | 1992 | 0.03 | 0.05 | 0.02 | 0.59 | 4664389 | 3376967464 | 2501157104 | 2332195 | 2498824909 | 0.74 |
| Deaths | Bahrain | 1992 | 0.29 | 0.43 | 0.19 | 0.60 | 353670.8 | 3376967464 | 2501510775 | 176835.4 | 2501333939 | 0.74 |
| Deaths | Kazakhstan | 1992 | 0.06 | 0.08 | 0.05 | 0.60 | 10145610 | 3376967464 | 2511656388 | 5072806 | 2506583581 | 0.74 |
| Deaths | Venezuela (Bolivarian Republic of) | 2021 | 0.59 | 0.81 | 0.42 | 0.60 | 17426450 | 5105255047 | 2021589354 | 8713227 | 2012876126 | 0.39 |
| Deaths | Argentina | 1992 | 1.16 | 1.33 | 1.01 | 0.60 | 20617890 | 3376967464 | 2532274273 | 10308940 | 2521965330 | 0.75 |

| | | | | | | | | | | | | | |
|---|---|---|---|---|---|---|---|---|---|---|---|---|---|
| Deaths | Bolivia (Plurinational State of) | 2021 | 0.38 | 0.63 | 0.22 | 0.60 | 7560756 | 5105255047 | 2029150109 | 3780378 | 2025369731 | 0.40 |
| Deaths | Chile | 1992 | 0.39 | 0.46 | 0.33 | 0.60 | 8721318 | 3376967464 | 2540995591 | 4360659 | 2536634932 | 0.75 |
| Deaths | Saint Kitts and Nevis | 1992 | 0.37 | 0.44 | 0.29 | 0.60 | 23875.01 | 3376967464 | 2541019466 | 11937.5 | 2541007529 | 0.75 |
| Deaths | Niue | 1992 | 0.57 | 0.97 | 0.32 | 0.60 | 1295.984 | 3376967464 | 2541020762 | 647.9919 | 2541020114 | 0.75 |
| Deaths | Kyrgyzstan | 2021 | 0.05 | 0.06 | 0.04 | 0.60 | 4248862 | 5105255047 | 2033398972 | 2124431 | 2031274540 | 0.40 |
| Deaths | Egypt | 2021 | 0.41 | 0.63 | 0.25 | 0.61 | 64397140 | 5105255047 | 2097796116 | 32198570 | 2065597544 | 0.40 |
| Deaths | Republic of Moldova | 1992 | 0.02 | 0.03 | 0.02 | 0.61 | 2854706 | 3376967464 | 2543875468 | 1427353 | 2542448115 | 0.75 |
| Deaths | Belize | 2021 | 0.12 | 0.15 | 0.09 | 0.61 | 283479.5 | 5105255047 | 2098079595 | 141739.7 | 2097937856 | 0.41 |
| Deaths | North Macedonia | 1992 | 0.11 | 0.18 | 0.07 | 0.61 | 1323446 | 3376967464 | 2545198915 | 661723.2 | 2544537192 | 0.75 |
| Deaths | Portugal | 1992 | 0.55 | 0.64 | 0.48 | 0.61 | 6780922 | 3376967464 | 2551979837 | 3390461 | 2548589376 | 0.75 |
| Deaths | Namibia | 2021 | 0.23 | 0.41 | 0.12 | 0.62 | 1504481 | 5105255047 | 2099584076 | 752240.5 | 2098831836 | 0.41 |
| Deaths | Mongolia | 2021 | 0.12 | 0.18 | 0.07 | 0.62 | 2105152 | 5105255047 | 2101689228 | 1052576 | 2100636652 | 0.41 |
| Deaths | American Samoa | 1992 | 0.58 | 0.94 | 0.34 | 0.62 | 29749.7 | 3376967464 | 2552009587 | 14874.85 | 2551994712 | 0.76 |
| Deaths | Dominican Republic | 2021 | 0.36 | 0.55 | 0.22 | 0.62 | 7231298 | 5105255047 | 2108920526 | 3615649 | 2105304877 | 0.41 |
| Deaths | Syrian Arab Republic | 2021 | 0.91 | 1.49 | 0.49 | 0.62 | 9352995 | 5105255047 | 2118273521 | 4676498 | 2113597023 | 0.41 |
| Deaths | Antigua and Barbuda | 1992 | 0.26 | 0.32 | 0.21 | 0.62 | 38493.21 | 3376967464 | 2552048080 | 19246.61 | 2552028833 | 0.76 |
| Deaths | Nauru | 2021 | 0.80 | 1.42 | 0.41 | 0.63 | 6647.105 | 5105255047 | 2118280168 | 3323.553 | 2118276845 | 0.41 |
| Deaths | Tonga | 2021 | 0.30 | 0.53 | 0.16 | 0.63 | 60598.31 | 5105255047 | 2118340767 | 30299.16 | 2118310467 | 0.41 |
| Deaths | Viet Nam | 2021 | 0.03 | 0.07 | 0.01 | 0.63 | 67836850 | 5105255047 | 2186177612 | 33918420 | 2152259189 | 0.42 |
| Deaths | Romania | 1992 | 0.25 | 0.30 | 0.21 | 0.63 | 15484770 | 3376967464 | 2567532852 | 7742386 | 2559790466 | 0.76 |
| Deaths | Palestine | 2021 | 0.12 | 0.19 | 0.08 | 0.63 | 3092703 | 5105255047 | 2189270315 | 1546351 | 2187723963 | 0.43 |
| Deaths | Belarus | 1992 | 0.03 | 0.03 | 0.02 | 0.63 | 6938289 | 3376967464 | 2574471141 | 3469144 | 2571001997 | 0.76 |
| Deaths | Suriname | 2021 | 0.41 | 0.66 | 0.23 | 0.63 | 384154.9 | 5105255047 | 2189654470 | 192077.4 | 2189462392 | 0.43 |
| Deaths | Trinidad and Tobago | 1992 | 0.22 | 0.27 | 0.19 | 0.63 | 745716.6 | 3376967464 | 2575216858 | 372858.3 | 2574843999 | 0.76 |

| | | | | | | | | | | | | |
|---|---|---|---|---|---|---|---|---|---|---|---|---|
| Deaths | Gabon | 2021 | 0.37 | 0.63 | 0.21 | 0.63 | 1102224 | 5105255047 | 2190756693 | 551111.8 | 2190205581 | 0.43 |
| Deaths | Paraguay | 2021 | 0.77 | 1.13 | 0.50 | 0.64 | 4680073 | 5105255047 | 2195436766 | 2340037 | 2193096730 | 0.43 |
| Deaths | Saint Vincent and the Grenadines | 2021 | 0.58 | 0.72 | 0.47 | 0.64 | 76552.59 | 5105255047 | 2195513319 | 38276.3 | 2195475043 | 0.43 |
| Deaths | Poland | 1992 | 0.21 | 0.22 | 0.20 | 0.64 | 24967360 | 3376967464 | 2600184220 | 12483680 | 2587700539 | 0.77 |
| Deaths | Serbia | 1992 | 0.59 | 0.90 | 0.35 | 0.64 | 6574516 | 3376967464 | 2606758736 | 3287258 | 2603471478 | 0.77 |
| Deaths | Botswana | 2021 | 0.18 | 0.31 | 0.09 | 0.64 | 1590402 | 5105255047 | 2197103721 | 795200.9 | 2196308520 | 0.43 |
| Deaths | Guyana | 2021 | 1.03 | 1.40 | 0.73 | 0.65 | 501391.8 | 5105255047 | 2197605113 | 250695.9 | 2197354417 | 0.43 |
| Deaths | Maldives | 2021 | 0.10 | 0.16 | 0.06 | 0.65 | 394937.9 | 5105255047 | 2198000050 | 197468.9 | 2197802582 | 0.43 |
| Deaths | Bulgaria | 1992 | 0.08 | 0.10 | 0.07 | 0.65 | 5699135 | 3376967464 | 2612457871 | 2849568 | 2609608303 | 0.77 |
| Deaths | Philippines | 2021 | 0.05 | 0.07 | 0.04 | 0.65 | 73079830 | 5105255047 | 2271079884 | 36539920 | 2234539967 | 0.44 |
| Deaths | Spain | 1992 | 1.25 | 1.43 | 1.08 | 0.65 | 26113210 | 3376967464 | 2638571077 | 13056600 | 2625514474 | 0.78 |
| Deaths | Brazil | 2021 | 0.59 | 0.62 | 0.55 | 0.65 | 150405800 | 5105255047 | 2421485716 | 75202920 | 2346282800 | 0.46 |
| Deaths | Colombia | 2021 | 0.61 | 0.77 | 0.48 | 0.66 | 33609590 | 5105255047 | 2455095302 | 16804790 | 2438290509 | 0.48 |
| Deaths | Indonesia | 2021 | 0.04 | 0.08 | 0.01 | 0.66 | 194150400 | 5105255047 | 2649245676 | 97075190 | 2552170489 | 0.50 |
| Deaths | Ukraine | 1992 | 0.05 | 0.06 | 0.04 | 0.66 | 34804310 | 3376967464 | 2673375387 | 17402160 | 2655973232 | 0.79 |
| Deaths | Equatorial Guinea | 2021 | 0.23 | 0.43 | 0.11 | 0.66 | 893663 | 5105255047 | 2650139339 | 446831.5 | 2649692507 | 0.52 |
| Deaths | Algeria | 2021 | 0.27 | 0.51 | 0.08 | 0.66 | 28054400 | 5105255047 | 2678193741 | 14027200 | 2664166540 | 0.52 |
| Deaths | Hungary | 1992 | 1.45 | 1.65 | 1.27 | 0.66 | 6927328 | 3376967464 | 2680302715 | 3463664 | 2676839051 | 0.79 |
| Deaths | Qatar | 1992 | 0.33 | 0.52 | 0.20 | 0.66 | 335643.3 | 3376967464 | 2680638358 | 167821.6 | 2680470537 | 0.79 |
| Deaths | Ecuador | 2021 | 0.36 | 0.48 | 0.27 | 0.66 | 11574520 | 5105255047 | 2689768262 | 5787261 | 2683981001 | 0.53 |
| Deaths | Peru | 2021 | 0.24 | 0.38 | 0.15 | 0.66 | 23885430 | 5105255047 | 2713653696 | 11942720 | 2701710979 | 0.53 |
| Deaths | Barbados | 1992 | 0.47 | 0.56 | 0.39 | 0.66 | 162757.9 | 3376967464 | 2680801116 | 81378.93 | 2680719737 | 0.79 |
| Deaths | Uzbekistan | 2021 | 0.11 | 0.14 | 0.09 | 0.66 | 22329360 | 5105255047 | 2735983057 | 11164680 | 2724818377 | 0.53 |
| Deaths | Iraq | 2021 | 0.38 | 0.65 | 0.18 | 0.66 | 26081420 | 5105255047 | 2762064476 | 13040710 | 2749023766 | 0.54 |

| | | | | | | | | | | | | |
|---|---|---|---|---|---|---|---|---|---|---|---|---|
| Deaths | Mexico | 2021 | 0.40 | 0.46 | 0.35 | 0.66 | 86646750 | 5105255047 | 2848711229 | 43323380 | 2805387853 | 0.55 |
| Deaths | Georgia | 1992 | 0.04 | 0.06 | 0.03 | 0.67 | 3570243 | 3376967464 | 2684371360 | 1785122 | 2682586238 | 0.79 |
| Deaths | Slovakia | 1992 | 0.26 | 0.38 | 0.18 | 0.67 | 3469779 | 3376967464 | 2687841139 | 1734890 | 2686106249 | 0.80 |
| Deaths | Cyprus | 1992 | 1.09 | 1.66 | 0.70 | 0.67 | 522214.5 | 3376967464 | 2688363353 | 261107.2 | 2688102246 | 0.80 |
| Deaths | Malta | 1992 | 0.32 | 0.37 | 0.27 | 0.67 | 249227.5 | 3376967464 | 2688612581 | 124613.8 | 2688487967 | 0.80 |
| Deaths | Guam | 1992 | 0.98 | 1.39 | 0.67 | 0.67 | 93009.67 | 3376967464 | 2688705591 | 46504.84 | 2688659086 | 0.80 |
| Deaths | Puerto Rico | 1992 | 0.74 | 0.86 | 0.63 | 0.67 | 2339319 | 3376967464 | 2691044910 | 1169660 | 2689875250 | 0.80 |
| Deaths | Cuba | 2021 | 0.71 | 0.86 | 0.57 | 0.67 | 7721598 | 5105255047 | 2856432827 | 3860799 | 2852572028 | 0.56 |
| Deaths | Grenada | 2021 | 0.76 | 0.96 | 0.59 | 0.67 | 71465.1 | 5105255047 | 2856504292 | 35732.55 | 2856468560 | 0.56 |
| Deaths | Kuwait | 1992 | 0.25 | 0.30 | 0.21 | 0.67 | 1142046 | 3376967464 | 2692186956 | 571023.1 | 2691615933 | 0.80 |
| Deaths | United States Virgin Islands | 1992 | 0.77 | 1.18 | 0.48 | 0.67 | 68428.68 | 3376967464 | 2692255385 | 34214.34 | 2692221171 | 0.80 |
| Deaths | Saint Lucia | 2021 | 0.43 | 0.55 | 0.33 | 0.67 | 127254.6 | 5105255047 | 2856631547 | 63627.3 | 2856567920 | 0.56 |
| Deaths | Montenegro | 1992 | 0.18 | 0.26 | 0.12 | 0.67 | 417608.2 | 3376967464 | 2692672993 | 208804.1 | 2692464189 | 0.80 |
| Deaths | Palau | 1992 | 0.47 | 0.81 | 0.23 | 0.67 | 10631.73 | 3376967464 | 2692683625 | 5315.866 | 2692678309 | 0.80 |
| Deaths | Fiji | 2021 | 0.16 | 0.26 | 0.09 | 0.68 | 595530.4 | 5105255047 | 2857227077 | 297765.2 | 2856929312 | 0.56 |
| Deaths | United Arab Emirates | 1992 | 0.79 | 1.27 | 0.46 | 0.68 | 1447269 | 3376967464 | 2694130893 | 723634.3 | 2693407259 | 0.80 |
| Deaths | Brunei Darussalam | 1992 | 0.26 | 0.40 | 0.16 | 0.68 | 172733 | 3376967464 | 2694303626 | 86366.52 | 2694217260 | 0.80 |
| Deaths | Croatia | 1992 | 0.36 | 0.42 | 0.31 | 0.68 | 3329713 | 3376967464 | 2697633339 | 1664857 | 2695968483 | 0.80 |
| Deaths | South Africa | 2021 | 0.32 | 0.43 | 0.24 | 0.68 | 37983600 | 5105255047 | 2895210682 | 18991800 | 2876218879 | 0.56 |
| Deaths | Lithuania | 1992 | 0.21 | 0.25 | 0.18 | 0.68 | 2447071 | 3376967464 | 2700080410 | 1223536 | 2698856875 | 0.80 |
| Deaths | Turkmenistan | 2021 | 0.06 | 0.09 | 0.05 | 0.68 | 3350351 | 5105255047 | 2898561033 | 1675176 | 2896885857 | 0.57 |
| Deaths | Tunisia | 2021 | 0.31 | 0.62 | 0.09 | 0.68 | 7946659 | 5105255047 | 2906507692 | 3973329 | 2902534362 | 0.57 |
| Deaths | Thailand | 2021 | 0.22 | 0.35 | 0.13 | 0.68 | 47274310 | 5105255047 | 2953782004 | 23637160 | 2930144848 | 0.57 |
| Deaths | Jamaica | 2021 | 0.10 | 0.14 | 0.07 | 0.68 | 1946551 | 5105255047 | 2955728554 | 973275.3 | 2954755279 | 0.58 |

| | | | | | | | | | | | | |
|---|---|---|---|---|---|---|---|---|---|---|---|---|
| Deaths | Taiwan (Province of China) | 1992 | 0.27 | 0.31 | 0.23 | 0.68 | 14026120 | 3376967464 | 2714106526 | 7013058 | 2707093468 | 0.80 |
| Deaths | Tokelau | 2021 | 0.60 | 1.03 | 0.34 | 0.69 | 843.5528 | 5105255047 | 2955729398 | 421.7764 | 2955728976 | 0.58 |
| Deaths | Greece | 1992 | 0.56 | 0.63 | 0.49 | 0.69 | 7122789 | 3376967464 | 2721229315 | 3561394 | 2717667921 | 0.80 |
| Deaths | Bahamas | 1992 | 0.29 | 0.35 | 0.24 | 0.69 | 171499.1 | 3376967464 | 2721400814 | 85749.56 | 2721315065 | 0.81 |
| Deaths | Russian Federation | 1992 | 0.12 | 0.12 | 0.11 | 0.69 | 101681600 | 3376967464 | 2823082430 | 50840810 | 2772241622 | 0.82 |
| Deaths | Azerbaijan | 2021 | 0.03 | 0.04 | 0.02 | 0.69 | 7440503 | 5105255047 | 2963169901 | 3720252 | 2959449649 | 0.58 |
| Deaths | Estonia | 1992 | 0.60 | 0.73 | 0.51 | 0.70 | 1018565 | 3376967464 | 2824100995 | 509282.4 | 2823591712 | 0.84 |
| Deaths | Latvia | 1992 | 0.36 | 0.41 | 0.31 | 0.70 | 1746906 | 3376967464 | 2825847901 | 873453 | 2824974448 | 0.84 |
| Deaths | Iran (Islamic Republic of) | 2021 | 0.28 | 0.41 | 0.22 | 0.70 | 59167520 | 5105255047 | 3022337423 | 29583760 | 2992753662 | 0.59 |
| Deaths | Czechia | 1992 | 0.20 | 0.23 | 0.17 | 0.70 | 6884471 | 3376967464 | 2832732372 | 3442236 | 2829290136 | 0.84 |
| Deaths | Costa Rica | 2021 | 0.74 | 0.89 | 0.61 | 0.70 | 3249833 | 5105255047 | 3025587256 | 1624917 | 3023962339 | 0.59 |
| Deaths | Sri Lanka | 2021 | 0.15 | 0.24 | 0.08 | 0.70 | 14714740 | 5105255047 | 3040301997 | 7357370 | 3032944626 | 0.59 |
| Deaths | Armenia | 2021 | 0.12 | 0.14 | 0.10 | 0.70 | 2004496 | 5105255047 | 3042306493 | 1002248 | 3041304245 | 0.60 |
| Deaths | Singapore | 1992 | 0.20 | 0.24 | 0.17 | 0.70 | 2369939 | 3376967464 | 2835102310 | 1184969 | 2833917341 | 0.84 |
| Deaths | Bermuda | 1992 | 2.74 | 3.22 | 2.26 | 0.70 | 42685.4 | 3376967464 | 2835144996 | 21342.7 | 2835123653 | 0.84 |
| Deaths | Albania | 2021 | 0.24 | 0.36 | 0.15 | 0.71 | 1808384 | 5105255047 | 3044114877 | 904192 | 3043210685 | 0.60 |
| Deaths | Panama | 2021 | 0.39 | 0.50 | 0.29 | 0.71 | 2749507 | 5105255047 | 3046864384 | 1374753 | 3045489630 | 0.60 |
| Deaths | Republic of Korea | 1992 | 0.15 | 0.22 | 0.09 | 0.71 | 31435440 | 3376967464 | 2866580440 | 15717720 | 2850862718 | 0.84 |
| Deaths | Turkey | 2021 | 0.42 | 0.65 | 0.26 | 0.71 | 56921960 | 5105255047 | 3103786340 | 28460980 | 3075325362 | 0.60 |
| Deaths | Northern Mariana Islands | 1992 | 0.82 | 1.29 | 0.49 | 0.72 | 33889.48 | 3376967464 | 2866614329 | 16944.74 | 2866597384 | 0.85 |
| Deaths | Italy | 1992 | 0.66 | 0.69 | 0.62 | 0.72 | 38983200 | 3376967464 | 2905597533 | 19491600 | 2886105931 | 0.85 |
| Deaths | Israel | 1992 | 0.49 | 0.57 | 0.41 | 0.72 | 3155772 | 3376967464 | 2908753305 | 1577886 | 2907175419 | 0.86 |
| Deaths | Mauritius | 2021 | 0.70 | 0.82 | 0.58 | 0.72 | 900058.5 | 5105255047 | 3104686398 | 450029.2 | 3104236369 | 0.61 |
| Deaths | Uruguay | 2021 | 1.24 | 1.44 | 1.07 | 0.72 | 2214747 | 5105255047 | 3106901145 | 1107373 | 3105793772 | 0.61 |

| | | | | | | | | | | | | |
|---|---|---|---|---|---|---|---|---|---|---|---|---|
| Deaths | China | 2021 | 0.04 | 0.05 | 0.03 | 0.72 | 967106700 | 5105255047 | 4074007884 | 483553400 | 3590454514 | 0.70 |
| Deaths | Bosnia and Herzegovina | 2021 | 0.43 | 0.69 | 0.23 | 0.72 | 2206873 | 5105255047 | 4076214757 | 1103437 | 4075111320 | 0.80 |
| Deaths | Argentina | 2021 | 0.70 | 0.81 | 0.60 | 0.72 | 30052210 | 5105255047 | 4106266964 | 15026100 | 4091240861 | 0.80 |
| Deaths | American Samoa | 2021 | 0.67 | 1.04 | 0.40 | 0.72 | 31908.17 | 5105255047 | 4106298872 | 15954.09 | 4106282918 | 0.80 |
| Deaths | Kazakhstan | 2021 | 0.09 | 0.12 | 0.07 | 0.73 | 12126520 | 5105255047 | 4118425393 | 6063260 | 4112362132 | 0.81 |
| Deaths | Jordan | 2021 | 0.17 | 0.26 | 0.11 | 0.73 | 8180007 | 5105255047 | 4126605399 | 4090003 | 4122515396 | 0.81 |
| Deaths | Libya | 2021 | 0.45 | 0.98 | 0.10 | 0.73 | 5029432 | 5105255047 | 4131634831 | 2514716 | 4129120115 | 0.81 |
| Deaths | Niue | 2021 | 0.60 | 1.03 | 0.34 | 0.73 | 1099.18 | 5105255047 | 4131635930 | 549.5901 | 4131635381 | 0.81 |
| Deaths | Greenland | 1992 | 1.31 | 2.09 | 0.64 | 0.73 | 38395.59 | 3376967464 | 2908791700 | 19197.79 | 2908772503 | 0.86 |
| Deaths | Seychelles | 2021 | 0.17 | 0.27 | 0.07 | 0.73 | 72957.22 | 5105255047 | 4131708887 | 36478.61 | 4131672409 | 0.81 |
| Deaths | Ireland | 1992 | 0.53 | 0.61 | 0.45 | 0.73 | 2251651 | 3376967464 | 2911043351 | 1125825 | 2909917526 | 0.86 |
| Deaths | Republic of Moldova | 2021 | 0.12 | 0.16 | 0.10 | 0.73 | 2516382 | 5105255047 | 4134225269 | 1258191 | 4132967078 | 0.81 |
| Deaths | Georgia | 2021 | 0.52 | 0.64 | 0.41 | 0.73 | 2299321 | 5105255047 | 4136524590 | 1149660 | 4135374929 | 0.81 |
| Deaths | Australia | 1992 | 0.58 | 0.67 | 0.51 | 0.73 | 11484530 | 3376967464 | 2922527885 | 5742267 | 2916785618 | 0.86 |
| Deaths | Slovenia | 1992 | 1.06 | 1.20 | 0.93 | 0.74 | 1357830 | 3376967464 | 2923885715 | 678914.9 | 2923206800 | 0.87 |
| Deaths | Malaysia | 2021 | 0.15 | 0.22 | 0.10 | 0.74 | 21856660 | 5105255047 | 4158381246 | 10928330 | 4147452918 | 0.81 |
| Deaths | France | 1992 | 1.07 | 1.24 | 0.92 | 0.74 | 38242170 | 3376967464 | 2962127885 | 19121090 | 2943006800 | 0.87 |
| Deaths | Portugal | 2021 | 0.70 | 0.80 | 0.60 | 0.74 | 6825510 | 5105255047 | 4165206755 | 3412755 | 4161794000 | 0.82 |
| Deaths | Lebanon | 2021 | 0.63 | 0.94 | 0.41 | 0.74 | 3716347 | 5105255047 | 4168923102 | 1858174 | 4167064929 | 0.82 |
| Deaths | Barbados | 2021 | 0.65 | 0.87 | 0.48 | 0.75 | 202783.5 | 5105255047 | 4169125886 | 101391.7 | 4169024494 | 0.82 |
| Deaths | Dominica | 2021 | 0.50 | 0.79 | 0.29 | 0.75 | 46130.66 | 5105255047 | 4169172016 | 23065.33 | 4169148951 | 0.82 |
| Deaths | Belgium | 1992 | 0.94 | 1.09 | 0.81 | 0.75 | 6685776 | 3376967464 | 2968813661 | 3342888 | 2965470773 | 0.88 |
| Deaths | Antigua and Barbuda | 2021 | 0.28 | 0.33 | 0.23 | 0.75 | 63578.22 | 5105255047 | 4169235595 | 31789.11 | 4169203806 | 0.82 |
| Deaths | North Macedonia | 2021 | 0.24 | 0.35 | 0.16 | 0.75 | 1541196 | 5105255047 | 4170776791 | 770598.2 | 4170006193 | 0.82 |

| | | | | | | | | | | | | |
|---|---|---|---|---|---|---|---|---|---|---|---|---|
| Deaths | Bahrain | 2021 | 0.29 | 0.42 | 0.19 | 0.75 | 1178377 | 5105255047 | 4171955168 | 589188.4 | 4171365980 | 0.82 |
| Deaths | Palau | 2021 | 0.51 | 0.85 | 0.29 | 0.75 | 13185.18 | 5105255047 | 4171968353 | 6592.589 | 4171961761 | 0.82 |
| Deaths | Austria | 1992 | 0.77 | 0.89 | 0.66 | 0.75 | 5270737 | 3376967464 | 2974084397 | 2635368 | 2971449029 | 0.88 |
| Deaths | United Kingdom | 1992 | 0.74 | 0.76 | 0.72 | 0.75 | 37397090 | 3376967464 | 3011481485 | 18698540 | 2992782941 | 0.89 |
| Deaths | Saint Kitts and Nevis | 2021 | 0.41 | 0.53 | 0.30 | 0.75 | 43420.68 | 5105255047 | 4172011774 | 21710.34 | 4171990064 | 0.82 |
| Deaths | New Zealand | 1992 | 0.73 | 0.83 | 0.65 | 0.76 | 2285123 | 3376967464 | 3013766608 | 1142561 | 3012624047 | 0.89 |
| Deaths | Ukraine | 2021 | 0.23 | 0.33 | 0.16 | 0.76 | 29287980 | 5105255047 | 4201299752 | 14643990 | 4186655763 | 0.82 |
| Deaths | Finland | 1992 | 0.90 | 1.04 | 0.76 | 0.76 | 3388185 | 3376967464 | 3017154793 | 1694093 | 3015460701 | 0.89 |
| Deaths | Andorra | 1992 | 0.38 | 0.62 | 0.22 | 0.77 | 45683.08 | 3376967464 | 3017200476 | 22841.54 | 3017177635 | 0.89 |
| Deaths | Bulgaria | 2021 | 0.43 | 0.53 | 0.34 | 0.77 | 4337506 | 5105255047 | 4205637258 | 2168753 | 4203468505 | 0.82 |
| Deaths | Romania | 2021 | 0.41 | 0.49 | 0.33 | 0.77 | 12142540 | 5105255047 | 4217779801 | 6071271 | 4211708529 | 0.82 |
| Deaths | Trinidad and Tobago | 2021 | 0.36 | 0.49 | 0.26 | 0.77 | 942563.1 | 5105255047 | 4218722364 | 471281.5 | 4218251082 | 0.83 |
| Deaths | Spain | 2021 | 0.65 | 0.75 | 0.57 | 0.77 | 29873650 | 5105255047 | 4248596015 | 14936830 | 4233659190 | 0.83 |
| Deaths | United States of America | 1992 | 0.85 | 0.89 | 0.82 | 0.77 | 169626400 | 3376967464 | 3186826882 | 84813200 | 3102013679 | 0.92 |
| Deaths | Chile | 2021 | 0.35 | 0.42 | 0.29 | 0.77 | 12819950 | 5105255047 | 4261415966 | 6409975 | 4255005991 | 0.83 |
| Deaths | Northern Mariana Islands | 2021 | 1.05 | 1.57 | 0.67 | 0.77 | 33644 | 5105255047 | 4261449610 | 16822 | 4261432788 | 0.83 |
| Deaths | Iceland | 1992 | 0.56 | 0.65 | 0.47 | 0.77 | 167194.9 | 3376967464 | 3186994077 | 83597.46 | 3186910480 | 0.94 |
| Deaths | Oman | 2021 | 0.08 | 0.14 | 0.05 | 0.77 | 3365550 | 5105255047 | 4264815160 | 1682775 | 4263132385 | 0.84 |
| Deaths | Cook Islands | 2021 | 0.22 | 0.43 | 0.11 | 0.78 | 11633.24 | 5105255047 | 4264826793 | 5816.619 | 4264820977 | 0.84 |
| Deaths | Belarus | 2021 | 0.12 | 0.15 | 0.09 | 0.78 | 6254502 | 5105255047 | 4271081295 | 3127251 | 4267954044 | 0.84 |
| Deaths | Canada | 1992 | 0.52 | 0.60 | 0.45 | 0.79 | 18901940 | 3376967464 | 3205896013 | 9450968 | 3196445045 | 0.95 |
| Deaths | Luxembourg | 1992 | 1.03 | 1.18 | 0.90 | 0.79 | 268190.4 | 3376967464 | 3206164203 | 134095.2 | 3206030108 | 0.95 |
| Deaths | Hungary | 2021 | 1.80 | 2.06 | 1.55 | 0.79 | 6199148 | 5105255047 | 4277280443 | 3099574 | 4274180869 | 0.84 |
| Deaths | Greece | 2021 | 0.69 | 0.79 | 0.59 | 0.79 | 6469340 | 5105255047 | 4283749783 | 3234670 | 4280515113 | 0.84 |

| | | | | | | | | | | | | |
|---|---|---|---|---|---|---|---|---|---|---|---|---|
| Deaths | Serbia | 2021 | 0.74 | 1.08 | 0.48 | 0.79 | 5934126 | 5105255047 | 4289683909 | 2967063 | 4286716846 | 0.84 |
| Deaths | Sweden | 1992 | 0.68 | 0.78 | 0.60 | 0.80 | 5553552 | 3376967464 | 3211717756 | 2776776 | 3208940980 | 0.95 |
| Deaths | Montenegro | 2021 | 0.23 | 0.33 | 0.15 | 0.80 | 412912.2 | 5105255047 | 4290096822 | 206456.1 | 4289890365 | 0.84 |
| Deaths | Croatia | 2021 | 0.98 | 1.19 | 0.80 | 0.80 | 2715472 | 5105255047 | 4292812293 | 1357736 | 4291454558 | 0.84 |
| Deaths | Japan | 1992 | 0.81 | 0.84 | 0.77 | 0.80 | 88370150 | 3376967464 | 3300087906 | 44185070 | 3255902831 | 0.96 |
| Deaths | Malta | 2021 | 0.49 | 0.57 | 0.41 | 0.80 | 278137.3 | 5105255047 | 4293090431 | 139068.6 | 4292951362 | 0.84 |
| Deaths | Netherlands | 1992 | 0.78 | 0.91 | 0.67 | 0.80 | 10396300 | 3376967464 | 3310484202 | 5198148 | 3305286054 | 0.98 |
| Deaths | Guam | 2021 | 0.86 | 1.20 | 0.61 | 0.80 | 103477.4 | 5105255047 | 4293193908 | 51738.72 | 4293142170 | 0.84 |
| Deaths | Bahamas | 2021 | 0.43 | 0.56 | 0.32 | 0.81 | 274938.4 | 5105255047 | 4293468847 | 137469.2 | 4293331377 | 0.84 |
| Deaths | Norway | 1992 | 0.69 | 0.73 | 0.65 | 0.81 | 2768970 | 3376967464 | 3313253172 | 1384485 | 3311868687 | 0.98 |
| Deaths | Italy | 2021 | 0.55 | 0.59 | 0.51 | 0.81 | 38186670 | 5105255047 | 4331655515 | 19093330 | 4312562181 | 0.84 |
| Deaths | Denmark | 1992 | 0.69 | 0.80 | 0.59 | 0.81 | 3484450 | 3376967464 | 3316737622 | 1742225 | 3314995397 | 0.98 |
| Deaths | Russian Federation | 2021 | 0.55 | 0.60 | 0.49 | 0.81 | 96028800 | 5105255047 | 4427684315 | 48014400 | 4379669915 | 0.86 |
| Deaths | Israel | 2021 | 0.43 | 0.51 | 0.36 | 0.81 | 5769267 | 5105255047 | 4433453582 | 2884633 | 4430568949 | 0.87 |
| Deaths | Brunei Darussalam | 2021 | 0.37 | 0.54 | 0.24 | 0.81 | 332019.1 | 5105255047 | 4433785601 | 166009.6 | 4433619592 | 0.87 |
| Deaths | Slovakia | 2021 | 0.51 | 0.71 | 0.35 | 0.81 | 3635982 | 5105255047 | 4437421583 | 1817991 | 4435603592 | 0.87 |
| Deaths | Poland | 2021 | 1.04 | 1.15 | 0.94 | 0.81 | 25179790 | 5105255047 | 4462601375 | 12589900 | 4450011479 | 0.87 |
| Deaths | Saudi Arabia | 2021 | 0.27 | 0.45 | 0.15 | 0.82 | 29117100 | 5105255047 | 4491718473 | 14558550 | 4477159924 | 0.88 |
| Deaths | Bermuda | 2021 | 2.37 | 2.99 | 1.86 | 0.82 | 41969.77 | 5105255047 | 4491760443 | 20984.88 | 4491739458 | 0.88 |
| Deaths | United States Virgin Islands | 2021 | 0.72 | 1.20 | 0.40 | 0.82 | 53866.94 | 5105255047 | 4491814310 | 26933.47 | 4491787376 | 0.88 |
| Deaths | San Marino | 1992 | 0.23 | 0.33 | 0.15 | 0.82 | 16789.73 | 3376967464 | 3316754411 | 8394.865 | 3316746017 | 0.98 |
| Deaths | Puerto Rico | 2021 | 0.93 | 1.18 | 0.72 | 0.83 | 2124836 | 5105255047 | 4493939146 | 1062418 | 4492876728 | 0.88 |
| Deaths | Greenland | 2021 | 1.12 | 1.70 | 0.66 | 0.83 | 39064.81 | 5105255047 | 4493978210 | 19532.41 | 4493958678 | 0.88 |
| Deaths | Germany | 1992 | 1.21 | 1.41 | 1.04 | 0.83 | 55437780 | 3376967464 | 3372192189 | 27718890 | 3344473300 | 0.99 |

| | | | | | | | | | | | | |
|---|---|---|---|---|---|---|---|---|---|---|---|---|
| Deaths | Czechia | 2021 | 0.81 | 0.97 | 0.67 | 0.83 | 6708178 | 5105255047 | 4500686388 | 3354089 | 4497332299 | 0.88 |
| Deaths | Latvia | 2021 | 1.10 | 1.30 | 0.91 | 0.83 | 1180840 | 5105255047 | 4501867228 | 590420 | 4501276808 | 0.88 |
| Deaths | Cyprus | 2021 | 0.81 | 1.17 | 0.55 | 0.84 | 940775.7 | 5105255047 | 4502808004 | 470387.8 | 4502337616 | 0.88 |
| Deaths | France | 2021 | 0.54 | 0.63 | 0.45 | 0.84 | 41021790 | 5105255047 | 4543829797 | 20510900 | 4523318900 | 0.89 |
| Deaths | Slovenia | 2021 | 1.90 | 2.28 | 1.53 | 0.84 | 1320405 | 5105255047 | 4545150201 | 660202.5 | 4544489999 | 0.89 |
| Deaths | Australia | 2021 | 0.40 | 0.47 | 0.35 | 0.84 | 16656050 | 5105255047 | 4561806249 | 8328024 | 4553478225 | 0.89 |
| Deaths | Estonia | 2021 | 1.49 | 1.79 | 1.23 | 0.84 | 825205.3 | 5105255047 | 4562631454 | 412602.6 | 4562218851 | 0.89 |
| Deaths | Kuwait | 2021 | 0.23 | 0.28 | 0.18 | 0.85 | 3633714 | 5105255047 | 4566265168 | 1816857 | 4564448311 | 0.89 |
| Deaths | Qatar | 2021 | 0.17 | 0.28 | 0.09 | 0.85 | 2446107 | 5105255047 | 4568711276 | 1223054 | 4567488222 | 0.89 |
| Deaths | United Arab Emirates | 2021 | 0.49 | 0.74 | 0.31 | 0.85 | 8129433 | 5105255047 | 4576840709 | 4064717 | 4572775992 | 0.90 |
| Deaths | New Zealand | 2021 | 0.65 | 0.74 | 0.58 | 0.85 | 3378026 | 5105255047 | 4580218735 | 1689013 | 4578529722 | 0.90 |
| Deaths | Monaco | 1992 | 0.32 | 0.50 | 0.19 | 0.85 | 19942.64 | 3376967464 | 3372212132 | 9971.318 | 3372202160 | 1.00 |
| Deaths | Belgium | 2021 | 0.74 | 0.86 | 0.64 | 0.85 | 7314275 | 5105255047 | 4587533010 | 3657137 | 4583875872 | 0.90 |
| Deaths | Austria | 2021 | 0.54 | 0.63 | 0.46 | 0.85 | 5970418 | 5105255047 | 4593503427 | 2985209 | 4590518219 | 0.90 |
| Deaths | Singapore | 2021 | 0.10 | 0.11 | 0.08 | 0.86 | 4146887 | 5105255047 | 4597650315 | 2073444 | 4595576871 | 0.90 |
| Deaths | Lithuania | 2021 | 0.84 | 1.00 | 0.69 | 0.86 | 1763517 | 5105255047 | 4599413832 | 881758.6 | 4598532074 | 0.90 |
| Deaths | United Kingdom | 2021 | 0.70 | 0.73 | 0.68 | 0.86 | 43558640 | 5105255047 | 4642972474 | 21779320 | 4621193153 | 0.91 |
| Deaths | Finland | 2021 | 0.81 | 0.95 | 0.69 | 0.86 | 3397926 | 5105255047 | 4646370400 | 1698963 | 4644671437 | 0.91 |
| Deaths | United States of America | 2021 | 0.63 | 0.66 | 0.60 | 0.86 | 216097500 | 5105255047 | 4862467891 | 108048700 | 4754419146 | 0.93 |
| Deaths | Switzerland | 1992 | 0.54 | 0.62 | 0.46 | 0.87 | 4755332 | 3376967464 | 3376967464 | 2377666 | 3374589798 | 1.00 |
| Deaths | Andorra | 2021 | 0.35 | 0.59 | 0.19 | 0.87 | 61742.59 | 5105255047 | 4862529634 | 30871.3 | 4862498763 | 0.95 |
| Deaths | Japan | 2021 | 0.28 | 0.29 | 0.27 | 0.87 | 75404150 | 5105255047 | 4937933788 | 37702080 | 4900231711 | 0.96 |
| Deaths | Canada | 2021 | 0.45 | 0.52 | 0.38 | 0.87 | 24262340 | 5105255047 | 4962196124 | 12131170 | 4950064956 | 0.97 |
| Deaths | Ireland | 2021 | 0.44 | 0.52 | 0.36 | 0.87 | 3192765 | 5105255047 | 4965388890 | 1596383 | 4963792507 | 0.97 |

| | | | | | | | | | | | | |
|---|---|---|---|---|---|---|---|---|---|---|---|---|
| Deaths | Taiwan (Province of China) | 2021 | 0.28 | 0.32 | 0.23 | 0.87 | 16681750 | 5105255047 | 4982070642 | 8340876 | 4973729766 | 0.97 |
| Deaths | Iceland | 2021 | 0.74 | 0.88 | 0.61 | 0.88 | 227618.6 | 5105255047 | 4982298260 | 113809.3 | 4982184451 | 0.98 |
| Deaths | Luxembourg | 2021 | 0.77 | 0.90 | 0.66 | 0.88 | 447039.5 | 5105255047 | 4982745300 | 223519.8 | 4982521780 | 0.98 |
| Deaths | Republic of Korea | 2021 | 0.14 | 0.20 | 0.10 | 0.89 | 36999740 | 5105255047 | 5019745039 | 18499870 | 5001245169 | 0.98 |
| Deaths | Sweden | 2021 | 0.38 | 0.46 | 0.32 | 0.89 | 6417083 | 5105255047 | 5026162122 | 3208541 | 5022953580 | 0.98 |
| Deaths | San Marino | 2021 | 0.21 | 0.35 | 0.10 | 0.89 | 21290.2 | 5105255047 | 5026183412 | 10645.1 | 5026172767 | 0.98 |
| Deaths | Netherlands | 2021 | 0.59 | 0.69 | 0.51 | 0.89 | 11069920 | 5105255047 | 5037253336 | 5534962 | 5031718374 | 0.99 |
| Deaths | Denmark | 2021 | 0.67 | 0.78 | 0.57 | 0.90 | 3719018 | 5105255047 | 5040972354 | 1859509 | 5039112845 | 0.99 |
| Deaths | Germany | 2021 | 0.94 | 1.10 | 0.80 | 0.90 | 54851780 | 5105255047 | 5095824131 | 27425890 | 5068398243 | 0.99 |
| Deaths | Monaco | 2021 | 0.30 | 0.49 | 0.17 | 0.91 | 23218.3 | 5105255047 | 5095847350 | 11609.15 | 5095835740 | 1.00 |
| Deaths | Norway | 2021 | 0.54 | 0.59 | 0.50 | 0.92 | 3522494 | 5105255047 | 5099369844 | 1761247 | 5097608597 | 1.00 |
| Deaths | Switzerland | 2021 | 0.41 | 0.47 | 0.34 | 0.93 | 5885204 | 5105255047 | 5105255047 | 2942602 | 5102312446 | 1.00 |
| Incidence | Somalia | 1992 | 0.95 | 1.43 | 0.59 | 0.05 | 3816581 | 3376967464 | 3816581 | 1908290 | 1908290.3 | 0.00 |
| Incidence | Somalia | 2021 | 0.84 | 1.27 | 0.51 | 0.08 | 10890630 | 5105255047 | 10890631 | 5445315 | 5445315.5 | 0.00 |
| Incidence | Niger | 1992 | 0.89 | 1.30 | 0.56 | 0.08 | 4059013 | 3376967464 | 7875594 | 2029507 | 5846087.2 | 0.00 |
| Incidence | Chad | 1992 | 0.86 | 1.24 | 0.55 | 0.12 | 3037794 | 3376967464 | 10913388 | 1518897 | 9394490.9 | 0.00 |
| Incidence | Mali | 1992 | 0.80 | 1.21 | 0.49 | 0.13 | 4416030 | 3376967464 | 15329418 | 2208015 | 13121403 | 0.00 |
| Incidence | Burkina Faso | 1992 | 0.90 | 1.31 | 0.58 | 0.14 | 4697926 | 3376967464 | 20027344 | 2348963 | 17678381.3 | 0.01 |
| Incidence | Ethiopia | 1992 | 0.89 | 1.35 | 0.53 | 0.15 | 27028040 | 3376967464 | 47055389 | 13514020 | 33541366.7 | 0.01 |
| Incidence | Niger | 2021 | 0.87 | 1.27 | 0.55 | 0.17 | 11701660 | 5105255047 | 22592286 | 5850828 | 16741458.7 | 0.00 |
| Incidence | Mozambique | 1992 | 0.96 | 1.44 | 0.58 | 0.18 | 6841201 | 3376967464 | 53896590 | 3420600 | 50475989.3 | 0.01 |
| Incidence | Afghanistan | 1992 | 1.90 | 2.76 | 1.22 | 0.18 | 5317936 | 3376967464 | 59214526 | 2658968 | 56555557.9 | 0.02 |
| Incidence | Guinea | 1992 | 0.98 | 1.41 | 0.63 | 0.18 | 3194335 | 3376967464 | 62408861 | 1597167 | 60811693.6 | 0.02 |
| Incidence | Uganda | 1992 | 0.96 | 1.47 | 0.58 | 0.19 | 8871780 | 3376967464 | 71280641 | 4435890 | 66844751.1 | 0.02 |

| | | | | | | | | | | | | |
|---|---|---|---|---|---|---|---|---|---|---|---|---|
| Incidence | Malawi | 1992 | 0.98 | 1.48 | 0.59 | 0.21 | 5373904 | 3376967464 | 76654545 | 2686952 | 73967593.3 | 0.02 |
| Incidence | Burundi | 1992 | 1.01 | 1.51 | 0.62 | 0.21 | 2868701 | 3376967464 | 79523246 | 1434350 | 78088895.8 | 0.02 |
| Incidence | South Sudan | 1992 | 1.08 | 1.64 | 0.65 | 0.21 | 3170246 | 3376967464 | 82693492 | 1585123 | 81108369 | 0.02 |
| Incidence | Nepal | 1992 | 1.25 | 1.92 | 0.75 | 0.21 | 10843820 | 3376967464 | 93537312 | 5421910 | 88115402.1 | 0.03 |
| Incidence | Sierra Leone | 1992 | 0.91 | 1.33 | 0.58 | 0.21 | 2160724 | 3376967464 | 95698036 | 1080362 | 94617674.1 | 0.03 |
| Incidence | Guinea-Bissau | 1992 | 0.98 | 1.39 | 0.64 | 0.22 | 518730.4 | 3376967464 | 96216766 | 259365.2 | 95957401.1 | 0.03 |
| Incidence | Central African Republic | 1992 | 1.01 | 1.50 | 0.65 | 0.22 | 1511635 | 3376967464 | 97728401 | 755817.3 | 96972583.7 | 0.03 |
| Incidence | Benin | 1992 | 0.97 | 1.41 | 0.62 | 0.23 | 2437709 | 3376967464 | 100166110 | 1218855 | 98947255.7 | 0.03 |
| Incidence | Bhutan | 1992 | 1.39 | 2.14 | 0.83 | 0.23 | 362111 | 3376967464 | 100528221 | 181055.5 | 100347165.9 | 0.03 |
| Incidence | Eritrea | 1992 | 0.93 | 1.38 | 0.58 | 0.23 | 1636011 | 3376967464 | 102164232 | 818005.3 | 101346226.7 | 0.03 |
| Incidence | Yemen | 1992 | 2.05 | 2.99 | 1.32 | 0.23 | 6649314 | 3376967464 | 108813546 | 3324657 | 105488888.8 | 0.03 |
| Incidence | Liberia | 1992 | 1.01 | 1.47 | 0.65 | 0.24 | 970267.7 | 3376967464 | 109783813 | 485133.8 | 109298679.5 | 0.03 |
| Incidence | Chad | 2021 | 0.97 | 1.39 | 0.63 | 0.24 | 8325851 | 5105255047 | 30918137 | 4162925 | 26755211.9 | 0.01 |
| Incidence | Bangladesh | 1992 | 1.28 | 1.95 | 0.76 | 0.25 | 59911290 | 3376967464 | 169695106 | 29955650 | 139739459.7 | 0.04 |
| Incidence | Senegal | 1992 | 0.90 | 1.32 | 0.55 | 0.25 | 3982520 | 3376967464 | 173677626 | 1991260 | 171686366 | 0.05 |
| Incidence | Gambia | 1992 | 0.99 | 1.44 | 0.62 | 0.25 | 540094.8 | 3376967464 | 174217721 | 270047.4 | 173947673.4 | 0.05 |
| Incidence | United Republic of Tanzania | 1992 | 1.15 | 1.73 | 0.71 | 0.26 | 13815680 | 3376967464 | 188033398 | 6907839 | 181125559.4 | 0.05 |
| Incidence | Mali | 2021 | 0.88 | 1.30 | 0.53 | 0.27 | 11895330 | 5105255047 | 42813466 | 5947664 | 36865801.5 | 0.01 |
| Incidence | Lao People's Democratic Republic | 1992 | 0.76 | 1.19 | 0.45 | 0.27 | 2290031 | 3376967464 | 190323429 | 1145016 | 189178413.7 | 0.06 |
| Incidence | Rwanda | 1992 | 1.14 | 1.68 | 0.72 | 0.28 | 3779329 | 3376967464 | 194102758 | 1889664 | 192213093.7 | 0.06 |
| Incidence | South Sudan | 2021 | 1.02 | 1.54 | 0.61 | 0.28 | 5135653 | 5105255047 | 47949119 | 2567827 | 45381292.2 | 0.01 |
| Incidence | Togo | 1992 | 0.96 | 1.40 | 0.61 | 0.28 | 1939975 | 3376967464 | 196042733 | 969987.5 | 195072745.7 | 0.06 |
| Incidence | Angola | 1992 | 1.03 | 1.53 | 0.63 | 0.28 | 5702682 | 3376967464 | 201745415 | 2851341 | 198894074.2 | 0.06 |

| | | | | | | | | | | | | |
|---|---|---|---|---|---|---|---|---|---|---|---|---|
| Incidence | Timor-Leste | 1992 | 0.77 | 1.21 | 0.45 | 0.28 | 447494.9 | 3376967464 | 202192910 | 223747.4 | 201969162.6 | 0.06 |
| Incidence | Madagascar | 1992 | 1.18 | 1.73 | 0.75 | 0.28 | 6432226 | 3376967464 | 208625136 | 3216113 | 205409023.2 | 0.06 |
| Incidence | Equatorial Guinea | 1992 | 1.06 | 1.57 | 0.66 | 0.28 | 229117.5 | 3376967464 | 208854254 | 114558.8 | 208739695 | 0.06 |
| Incidence | Burkina Faso | 2021 | 1.08 | 1.54 | 0.70 | 0.29 | 11698660 | 5105255047 | 59647781 | 5849331 | 53798449.8 | 0.01 |
| Incidence | Cabo Verde | 1992 | 1.01 | 1.47 | 0.64 | 0.29 | 183931.3 | 3376967464 | 209038185 | 91965.67 | 208946219.4 | 0.06 |
| Incidence | Comoros | 1992 | 1.09 | 1.66 | 0.66 | 0.29 | 249116.1 | 3376967464 | 209287301 | 124558 | 209162743.1 | 0.06 |
| Incidence | Burundi | 2021 | 0.91 | 1.39 | 0.54 | 0.29 | 7040681 | 5105255047 | 66688462 | 3520341 | 63168121.4 | 0.01 |
| Incidence | Democratic Republic of the Congo | 1992 | 1.10 | 1.64 | 0.69 | 0.29 | 20457730 | 3376967464 | 229745032 | 10228870 | 219516166.6 | 0.07 |
| Incidence | Coted'Ivoire | 1992 | 1.09 | 1.56 | 0.70 | 0.29 | 6800901 | 3376967464 | 236545933 | 3400450 | 233145482.4 | 0.07 |
| Incidence | Cambodia | 1992 | 0.75 | 1.17 | 0.43 | 0.30 | 5566488 | 3376967464 | 242112421 | 2783244 | 239329176.7 | 0.07 |
| Incidence | Sudan | 1992 | 2.12 | 3.14 | 1.34 | 0.30 | 11202790 | 3376967464 | 253315211 | 5601395 | 247713815.6 | 0.07 |
| Incidence | Zambia | 1992 | 1.02 | 1.54 | 0.62 | 0.31 | 4197849 | 3376967464 | 257513060 | 2098925 | 255414135.1 | 0.08 |
| Incidence | Central African Republic | 2021 | 0.99 | 1.42 | 0.62 | 0.31 | 3075108 | 5105255047 | 69763570 | 1537554 | 68226015.8 | 0.01 |
| Incidence | Nigeria | 1992 | 1.01 | 1.50 | 0.63 | 0.31 | 50478270 | 3376967464 | 307991334 | 25239140 | 282752197 | 0.08 |
| Incidence | Solomon Islands | 1992 | 1.27 | 1.92 | 0.79 | 0.31 | 187733.3 | 3376967464 | 308179068 | 93866.66 | 308085200.9 | 0.09 |
| Incidence | Sao Tome and Principe | 1992 | 0.93 | 1.39 | 0.57 | 0.31 | 61668.72 | 3376967464 | 308240736 | 30834.36 | 308209902 | 0.09 |
| Incidence | Cameroon | 1992 | 1.14 | 1.64 | 0.74 | 0.32 | 5631620 | 3376967464 | 313872356 | 2815810 | 311056546.2 | 0.09 |
| Incidence | Guatemala | 1992 | 4.33 | 6.33 | 2.79 | 0.32 | 4330417 | 3376967464 | 318202773 | 2165209 | 316037564.7 | 0.09 |
| Incidence | Papua New Guinea | 1992 | 1.09 | 1.67 | 0.66 | 0.32 | 2419850 | 3376967464 | 320622623 | 1209925 | 319412698.1 | 0.09 |
| Incidence | Haiti | 1992 | 3.98 | 5.73 | 2.69 | 0.32 | 3615033 | 3376967464 | 324237656 | 1807516 | 322430139.3 | 0.10 |
| Incidence | Pakistan | 1992 | 1.48 | 2.26 | 0.89 | 0.32 | 60357000 | 3376967464 | 384594651 | 30178500 | 354416153.3 | 0.10 |
| Incidence | Myanmar | 1992 | 0.82 | 1.26 | 0.48 | 0.33 | 24926270 | 3376967464 | 409520920 | 12463130 | 397057785.4 | 0.12 |
| Incidence | Mozambique | 2021 | 1.22 | 1.80 | 0.79 | 0.33 | 16039550 | 5105255047 | 85803118 | 8019774 | 77783343.8 | 0.02 |

| | | | | | | | | | | | | |
|---|---|---|---|---|---|---|---|---|---|---|---|---|
| Incidence | Guinea | 2021 | 1.09 | 1.53 | 0.71 | 0.34 | 6958615 | 5105255047 | 92761733 | 3479307 | 89282425.3 | 0.02 |
| Incidence | Afghanistan | 2021 | 2.04 | 2.93 | 1.34 | 0.34 | 16398400 | 5105255047 | 109160135 | 8199201 | 100960934 | 0.02 |
| Incidence | Djibouti | 1992 | 1.07 | 1.61 | 0.65 | 0.34 | 281064.1 | 3376967464 | 409801984 | 140532.1 | 409661452 | 0.12 |
| Incidence | Honduras | 1992 | 4.68 | 6.76 | 3.09 | 0.34 | 2511178 | 3376967464 | 412313162 | 1255589 | 411057573.1 | 0.12 |
| Incidence | India | 1992 | 1.27 | 1.96 | 0.76 | 0.34 | 515839400 | 3376967464 | 928152513 | 257919700 | 670232837.4 | 0.20 |
| Incidence | Kenya | 1992 | 1.00 | 1.54 | 0.59 | 0.35 | 12235470 | 3376967464 | 940387986 | 6117737 | 934270249.4 | 0.28 |
| Incidence | Mauritania | 1992 | 1.09 | 1.58 | 0.69 | 0.35 | 1105278 | 3376967464 | 941493264 | 552639.1 | 940940625.2 | 0.28 |
| Incidence | Lesotho | 1992 | 1.05 | 1.60 | 0.62 | 0.35 | 821010.8 | 3376967464 | 942314275 | 410505.4 | 941903769.7 | 0.28 |
| Incidence | Liberia | 2021 | 1.15 | 1.62 | 0.74 | 0.35 | 3135229 | 5105255047 | 112295364 | 1567614 | 110727749.6 | 0.02 |
| Incidence | Guinea-Bissau | 2021 | 1.08 | 1.52 | 0.72 | 0.35 | 1119372 | 5105255047 | 113414736 | 559686.2 | 112855050.1 | 0.02 |
| Incidence | Nicaragua | 1992 | 5.44 | 7.76 | 3.64 | 0.36 | 2103741 | 3376967464 | 944418017 | 1051871 | 943366145.8 | 0.28 |
| Incidence | Maldives | 1992 | 1.28 | 2.00 | 0.78 | 0.36 | 118284.3 | 3376967464 | 944536301 | 59142.14 | 944477158.7 | 0.28 |
| Incidence | Sierra Leone | 2021 | 1.01 | 1.46 | 0.64 | 0.36 | 5014942 | 5105255047 | 118429679 | 2507471 | 115922207.5 | 0.02 |
| Incidence | Ethiopia | 2021 | 0.88 | 1.34 | 0.52 | 0.36 | 61363610 | 5105255047 | 179793292 | 30681810 | 149111485.3 | 0.03 |
| Incidence | Vanuatu | 1992 | 1.31 | 1.96 | 0.80 | 0.36 | 84103.98 | 3376967464 | 944620405 | 42051.99 | 944578352.8 | 0.28 |
| Incidence | Morocco | 1992 | 2.51 | 3.67 | 1.60 | 0.37 | 15160090 | 3376967464 | 959780492 | 7580044 | 952200448.5 | 0.28 |
| Incidence | Benin | 2021 | 1.09 | 1.54 | 0.70 | 0.37 | 7048559 | 5105255047 | 186841851 | 3524280 | 183317571.5 | 0.04 |
| Incidence | El Salvador | 1992 | 4.97 | 7.22 | 3.27 | 0.38 | 3000660 | 3376967464 | 962781152 | 1500330 | 961280822.3 | 0.28 |
| Incidence | Democratic Republic of the Congo | 2021 | 1.19 | 1.72 | 0.75 | 0.38 | 49675100 | 5105255047 | 236516952 | 24837550 | 211679401.7 | 0.04 |
| Incidence | Malawi | 2021 | 1.13 | 1.72 | 0.69 | 0.38 | 10785330 | 5105255047 | 247302286 | 5392667 | 241909619.1 | 0.05 |
| Incidence | Ghana | 1992 | 1.01 | 1.48 | 0.64 | 0.38 | 8290370 | 3376967464 | 971071522 | 4145185 | 966926337.2 | 0.29 |
| Incidence | Madagascar | 2021 | 1.22 | 1.78 | 0.77 | 0.40 | 16136660 | 5105255047 | 263438948 | 8068331 | 255370616.9 | 0.05 |
| Incidence | Eritrea | 2021 | 0.93 | 1.39 | 0.58 | 0.40 | 3903951 | 5105255047 | 267342899 | 1951975 | 265390923.2 | 0.05 |

| | | | | | | | | | | | | |
|---|---|---|---|---|---|---|---|---|---|---|---|---|
| Incidence | Senegal | 2021 | 1.00 | 1.45 | 0.62 | 0.41 | 8916317 | 5105255047 | 276259216 | 4458159 | 271801057.2 | 0.05 |
| Incidence | Togo | 2021 | 1.12 | 1.57 | 0.74 | 0.41 | 4808399 | 5105255047 | 281067615 | 2404199 | 278663415.3 | 0.05 |
| Incidence | Gambia | 2021 | 1.16 | 1.68 | 0.74 | 0.41 | 1328594 | 5105255047 | 282396209 | 664296.9 | 281731911.6 | 0.06 |
| Incidence | Palestine | 1992 | 2.26 | 3.44 | 1.36 | 0.41 | 1092733 | 3376967464 | 972164255 | 546366.4 | 971617888.3 | 0.29 |
| Incidence | Zimbabwe | 1992 | 1.27 | 1.95 | 0.77 | 0.41 | 5556213 | 3376967464 | 977720467 | 2778106 | 974942361.1 | 0.29 |
| Incidence | Kiribati | 1992 | 1.39 | 2.10 | 0.87 | 0.42 | 43348.47 | 3376967464 | 977763816 | 21674.24 | 977742141.7 | 0.29 |
| Incidence | Eswatini | 1992 | 1.35 | 2.04 | 0.82 | 0.42 | 429553.2 | 3376967464 | 978193369 | 214776.6 | 977978592.6 | 0.29 |
| Incidence | Papua New Guinea | 2021 | 1.26 | 1.89 | 0.77 | 0.42 | 6230283 | 5105255047 | 288626492 | 3115142 | 285511350.2 | 0.06 |
| Incidence | Viet Nam | 1992 | 0.93 | 1.46 | 0.55 | 0.42 | 40268370 | 3376967464 | 1018461738 | 20134180 | 998327553.6 | 0.30 |
| Incidence | Iraq | 1992 | 2.48 | 3.76 | 1.52 | 0.42 | 10043140 | 3376967464 | 1028504876 | 5021569 | 1023483307 | 0.30 |
| Incidence | Tuvalu | 1992 | 1.36 | 2.07 | 0.84 | 0.42 | 5474.875 | 3376967464 | 1028510350 | 2737.437 | 1028507613 | 0.30 |
| Incidence | Uganda | 2021 | 1.12 | 1.67 | 0.69 | 0.42 | 22465620 | 5105255047 | 311092115 | 11232810 | 299859303.5 | 0.06 |
| Incidence | Coted'Ivoire | 2021 | 1.23 | 1.73 | 0.81 | 0.43 | 15562160 | 5105255047 | 326654276 | 7781080 | 318873195.4 | 0.06 |
| Incidence | Solomon Islands | 2021 | 1.62 | 2.46 | 1.02 | 0.43 | 400945.3 | 5105255047 | 327055221 | 200472.7 | 326854748.6 | 0.06 |
| Incidence | Congo | 1992 | 1.24 | 1.87 | 0.78 | 0.43 | 1365818 | 3376967464 | 1029876169 | 682909 | 1029193260 | 0.30 |
| Incidence | Nepal | 2021 | 1.82 | 2.71 | 1.15 | 0.43 | 19987970 | 5105255047 | 347043194 | 9993986 | 337049207.6 | 0.07 |
| Incidence | Bolivia (Plurinational State of) | 1992 | 4.77 | 6.90 | 3.13 | 0.43 | 3645533 | 3376967464 | 1033521701 | 1822766 | 1031698935 | 0.31 |
| Incidence | Rwanda | 2021 | 1.14 | 1.71 | 0.69 | 0.44 | 7848418 | 5105255047 | 354891612 | 3924209 | 350967402.9 | 0.07 |
| Incidence | Egypt | 1992 | 2.91 | 4.27 | 1.84 | 0.44 | 33112790 | 3376967464 | 1066634488 | 16556390 | 1050078095 | 0.31 |
| Incidence | Botswana | 1992 | 1.21 | 1.82 | 0.73 | 0.44 | 747651.5 | 3376967464 | 1067382140 | 373825.7 | 1067008314 | 0.32 |
| Incidence | Marshall Islands | 1992 | 1.42 | 2.16 | 0.86 | 0.44 | 23628.24 | 3376967464 | 1067405768 | 11814.12 | 1067393954 | 0.32 |
| Incidence | Belize | 1992 | 5.24 | 7.58 | 3.44 | 0.44 | 103326.3 | 3376967464 | 1067509094 | 51663.13 | 1067457431 | 0.32 |
| Incidence | Timor-Leste | 2021 | 0.99 | 1.50 | 0.60 | 0.44 | 802737.4 | 5105255047 | 355694349 | 401368.7 | 355292980.6 | 0.07 |
| Incidence | Syrian Arab Republic | 1992 | 4.49 | 6.57 | 2.92 | 0.45 | 6931728 | 3376967464 | 1074440822 | 3465864 | 1070974958 | 0.32 |

| | | | | | | | | | | | | |
|---|---|---|---|---|---|---|---|---|---|---|---|---|
| Incidence | United Republic of Tanzania | 2021 | 1.29 | 1.90 | 0.80 | 0.45 | 32205890 | 5105255047 | 387900240 | 16102950 | 371797294.8 | 0.07 |
| Incidence | Haiti | 2021 | 4.51 | 6.37 | 3.00 | 0.45 | 8005224 | 5105255047 | 395905464 | 4002612 | 391902852.4 | 0.08 |
| Incidence | Dominican Republic | 1992 | 4.54 | 6.61 | 2.93 | 0.45 | 4367623 | 3376967464 | 1078808445 | 2183812 | 1076624634 | 0.32 |
| Incidence | Yemen | 2021 | 2.26 | 3.24 | 1.48 | 0.45 | 18839690 | 5105255047 | 414745156 | 9419846 | 405325310.2 | 0.08 |
| Incidence | Angola | 2021 | 1.24 | 1.83 | 0.78 | 0.45 | 16719890 | 5105255047 | 431465050 | 8359947 | 423105102.9 | 0.08 |
| Incidence | Grenada | 1992 | 5.82 | 8.23 | 3.95 | 0.46 | 48427.92 | 3376967464 | 1078856873 | 24213.96 | 1078832659 | 0.32 |
| Incidence | Oman | 1992 | 2.33 | 3.49 | 1.44 | 0.46 | 1153630 | 3376967464 | 1080010503 | 576815.1 | 1079433688 | 0.32 |
| Incidence | Namibia | 1992 | 1.20 | 1.81 | 0.72 | 0.46 | 804903.9 | 3376967464 | 1080815407 | 402451.9 | 1080412955 | 0.32 |
| Incidence | Guyana | 1992 | 7.91 | 10.77 | 5.52 | 0.47 | 459165.3 | 3376967464 | 1081274573 | 229582.7 | 1081044990 | 0.32 |
| Incidence | Gabon | 1992 | 1.40 | 2.09 | 0.89 | 0.47 | 557425.5 | 3376967464 | 1081831998 | 278712.8 | 1081553285 | 0.32 |
| Incidence | Bhutan | 2021 | 2.04 | 3.06 | 1.25 | 0.47 | 519639.6 | 5105255047 | 431984689 | 259819.8 | 431724869.5 | 0.08 |
| Incidence | Vanuatu | 2021 | 1.68 | 2.52 | 1.06 | 0.47 | 184213.1 | 5105255047 | 432168902 | 92106.55 | 432076795.9 | 0.08 |
| Incidence | Cambodia | 2021 | 1.07 | 1.64 | 0.63 | 0.47 | 10996940 | 5105255047 | 443165844 | 5498471 | 437667373 | 0.09 |
| Incidence | Micronesia (Federated States of) | 1992 | 1.58 | 2.41 | 0.99 | 0.47 | 55679.86 | 3376967464 | 1081887678 | 27839.93 | 1081859838 | 0.32 |
| Incidence | Zimbabwe | 2021 | 1.32 | 1.96 | 0.83 | 0.47 | 8807361 | 5105255047 | 451973205 | 4403681 | 447569524.4 | 0.09 |
| Incidence | Tajikistan | 1992 | 4.19 | 6.28 | 2.65 | 0.48 | 2943035 | 3376967464 | 1084830713 | 1471517 | 1083359195 | 0.32 |
| Incidence | Indonesia | 1992 | 0.85 | 1.32 | 0.49 | 0.48 | 115716600 | 3376967464 | 1200547263 | 57858280 | 1142688988 | 0.34 |
| Incidence | China | 1992 | 1.17 | 1.81 | 0.68 | 0.48 | 815239200 | 3376967464 | 2015786424 | 407619600 | 1608166844 | 0.48 |
| Incidence | Algeria | 1992 | 2.90 | 4.29 | 1.87 | 0.48 | 14528470 | 3376967464 | 2030314898 | 7264237 | 2023050661 | 0.60 |
| Incidence | Comoros | 2021 | 1.09 | 1.65 | 0.65 | 0.48 | 467233.1 | 5105255047 | 452440438 | 233616.6 | 452206821.7 | 0.09 |
| Incidence | Turkey | 1992 | 3.22 | 4.80 | 2.03 | 0.48 | 36334360 | 3376967464 | 2066649254 | 18167180 | 2048482076 | 0.61 |
| Incidence | Mongolia | 1992 | 4.99 | 7.37 | 3.21 | 0.48 | 1235917 | 3376967464 | 2067885171 | 617958.5 | 2067267212 | 0.61 |
| Incidence | Paraguay | 1992 | 6.99 | 9.71 | 4.72 | 0.48 | 2324234 | 3376967464 | 2070209405 | 1162117 | 2069047288 | 0.61 |
| Incidence | Cameroon | 2021 | 1.38 | 1.95 | 0.92 | 0.48 | 17450670 | 5105255047 | 469891104 | 8725333 | 461165771 | 0.09 |

| | | | | | | | | | | | | |
|---|---|---|---|---|---|---|---|---|---|---|---|---|
| Incidence | Iran (Islamic Republic of) | 1992 | 2.87 | 4.28 | 1.78 | 0.48 | 32234900 | 3376967464 | 2102444303 | 16117450 | 2086326854 | 0.62 |
| Incidence | Colombia | 1992 | 8.57 | 11.78 | 5.93 | 0.48 | 20427380 | 3376967464 | 2122871685 | 10213690 | 2112657994 | 0.63 |
| Incidence | Saint Vincent and the Grenadines | 1992 | 5.75 | 8.20 | 3.78 | 0.49 | 63082.62 | 3376967464 | 2122934768 | 31541.31 | 2122903226 | 0.63 |
| Incidence | Djibouti | 2021 | 1.17 | 1.75 | 0.72 | 0.49 | 805751.6 | 5105255047 | 470696855 | 402875.8 | 470293979.5 | 0.09 |
| Incidence | Tunisia | 1992 | 3.07 | 4.54 | 1.95 | 0.49 | 5133459 | 3376967464 | 2128068226 | 2566729 | 2125501497 | 0.63 |
| Incidence | Lao People's Democratic Republic | 2021 | 1.08 | 1.68 | 0.64 | 0.49 | 4754006 | 5105255047 | 475450861 | 2377003 | 473073858.3 | 0.09 |
| Incidence | Bangladesh | 2021 | 1.68 | 2.57 | 1.05 | 0.49 | 107272700 | 5105255047 | 582723583 | 53636360 | 529087222.1 | 0.10 |
| Incidence | Samoa | 1992 | 1.82 | 2.80 | 1.12 | 0.49 | 92361.26 | 3376967464 | 2128160588 | 46180.63 | 2128114407 | 0.63 |
| Incidence | Mauritania | 2021 | 1.20 | 1.71 | 0.75 | 0.50 | 2374531 | 5105255047 | 585098113 | 1187265 | 583910848.2 | 0.11 |
| Incidence | Democratic People's Republic of Korea | 1992 | 1.11 | 1.69 | 0.66 | 0.50 | 13875620 | 3376967464 | 2142036206 | 6937809 | 2135098397 | 0.63 |
| Incidence | Uzbekistan | 1992 | 5.17 | 7.75 | 3.26 | 0.50 | 12096580 | 3376967464 | 2154132781 | 6048288 | 2148084494 | 0.64 |
| Incidence | Nigeria | 2021 | 0.99 | 1.46 | 0.61 | 0.50 | 123377300 | 5105255047 | 708475425 | 61688660 | 646786769.3 | 0.13 |
| Incidence | Tonga | 1992 | 1.78 | 2.69 | 1.09 | 0.50 | 53374.01 | 3376967464 | 2154186155 | 26687 | 2154159468 | 0.64 |
| Incidence | Pakistan | 2021 | 1.79 | 2.71 | 1.11 | 0.50 | 141565600 | 5105255047 | 850041000 | 70782790 | 779258212.8 | 0.15 |
| Incidence | Sao Tome and Principe | 2021 | 1.23 | 1.78 | 0.79 | 0.51 | 131024.1 | 5105255047 | 850172025 | 65512.04 | 850106512.5 | 0.17 |
| Incidence | Zambia | 2021 | 1.33 | 1.96 | 0.83 | 0.51 | 10789070 | 5105255047 | 860961092 | 5394534 | 855566558.4 | 0.17 |
| Incidence | Brazil | 1992 | 7.66 | 11.06 | 4.99 | 0.51 | 93825900 | 3376967464 | 2248012056 | 46912950 | 2201099106 | 0.65 |
| Incidence | Lesotho | 2021 | 1.32 | 1.95 | 0.83 | 0.51 | 1159988 | 5105255047 | 862121080 | 579994.1 | 861541086.2 | 0.17 |
| Incidence | Suriname | 1992 | 5.12 | 7.39 | 3.43 | 0.51 | 240092.4 | 3376967464 | 2248252148 | 120046.2 | 2248132102 | 0.67 |
| Incidence | Honduras | 2021 | 6.98 | 9.73 | 4.75 | 0.51 | 6325127 | 5105255047 | 868446208 | 3162564 | 865283644 | 0.17 |
| Incidence | Peru | 1992 | 4.75 | 6.84 | 3.14 | 0.51 | 13017280 | 3376967464 | 2261269426 | 6508639 | 2254760787 | 0.67 |
| Incidence | Mexico | 1992 | 6.89 | 10.12 | 4.47 | 0.51 | 51527500 | 3376967464 | 2312796928 | 25763750 | 2287033177 | 0.68 |

| | | | | | | | | | | | | |
|---|---|---|---|---|---|---|---|---|---|---|---|---|
| Incidence | Saint Lucia | 1992 | 6.18 | 8.67 | 4.20 | 0.52 | 80360.32 | 3376967464 | 2312877288 | 40180.16 | 2312837108 | 0.68 |
| Incidence | Philippines | 1992 | 0.80 | 1.28 | 0.45 | 0.52 | 37716100 | 3376967464 | 2350593387 | 18858050 | 2331735338 | 0.69 |
| Incidence | Ecuador | 1992 | 6.47 | 8.87 | 4.49 | 0.52 | 6008273 | 3376967464 | 2356601661 | 3004137 | 2353597524 | 0.70 |
| Incidence | Kenya | 2021 | 1.17 | 1.79 | 0.71 | 0.52 | 29742370 | 5105255047 | 898188583 | 14871190 | 883317395.1 | 0.17 |
| Incidence | Nicaragua | 2021 | 7.35 | 10.45 | 4.79 | 0.52 | 4297839 | 5105255047 | 902486421 | 2148919 | 900337502 | 0.18 |
| Incidence | Thailand | 1992 | 1.20 | 1.84 | 0.72 | 0.53 | 39057400 | 3376967464 | 2395659056 | 19528700 | 2376130359 | 0.70 |
| Incidence | Kiribati | 2021 | 1.79 | 2.64 | 1.14 | 0.53 | 74513.75 | 5105255047 | 902560935 | 37256.87 | 902523678.2 | 0.18 |
| Incidence | Kyrgyzstan | 1992 | 5.24 | 7.73 | 3.31 | 0.53 | 2635720 | 3376967464 | 2398294776 | 1317860 | 2396976916 | 0.71 |
| Incidence | Venezuela (Bolivarian Republic of) | 1992 | 7.91 | 10.86 | 5.52 | 0.53 | 11607490 | 3376967464 | 2409902266 | 5803745 | 2404098521 | 0.71 |
| Incidence | Tokelau | 1992 | 1.42 | 2.16 | 0.86 | 0.53 | 834.3087 | 3376967464 | 2409903100 | 417.1543 | 2409902683 | 0.71 |
| Incidence | Sri Lanka | 1992 | 1.92 | 2.78 | 1.22 | 0.53 | 11121210 | 3376967464 | 2421024309 | 5560605 | 2415463705 | 0.72 |
| Incidence | Cabo Verde | 2021 | 1.50 | 2.13 | 0.98 | 0.53 | 382357.4 | 5105255047 | 902943292 | 191178.7 | 902752113.8 | 0.18 |
| Incidence | Myanmar | 2021 | 1.17 | 1.79 | 0.70 | 0.53 | 36996570 | 5105255047 | 939939864 | 18498290 | 921441578.5 | 0.18 |
| Incidence | Nauru | 1992 | 1.74 | 2.66 | 1.06 | 0.54 | 5770.117 | 3376967464 | 2421030079 | 2885.058 | 2421027194 | 0.72 |
| Incidence | Bosnia and Herzegovina | 1992 | 11.16 | 15.59 | 7.33 | 0.54 | 3112775 | 3376967464 | 2424142855 | 1556388 | 2422586467 | 0.72 |
| Incidence | Guatemala | 2021 | 5.94 | 8.50 | 3.99 | 0.54 | 9913414 | 5105255047 | 949853279 | 4956707 | 944896571.5 | 0.19 |
| Incidence | Tajikistan | 2021 | 5.32 | 7.82 | 3.42 | 0.54 | 6207893 | 5105255047 | 956061172 | 3103947 | 952957225.2 | 0.19 |
| Incidence | Lebanon | 1992 | 3.40 | 4.97 | 2.17 | 0.54 | 1853583 | 3376967464 | 2425996438 | 926791.5 | 2425069646 | 0.72 |
| Incidence | Sudan | 2021 | 2.80 | 4.09 | 1.81 | 0.54 | 25440510 | 5105255047 | 981501682 | 12720260 | 968781427 | 0.19 |
| Incidence | Fiji | 1992 | 1.35 | 2.06 | 0.81 | 0.54 | 465040.9 | 3376967464 | 2426461479 | 232520.5 | 2426228958 | 0.72 |
| Incidence | Costa Rica | 1992 | 9.66 | 13.43 | 6.76 | 0.54 | 1893843 | 3376967464 | 2428355322 | 946921.6 | 2427408400 | 0.72 |
| Incidence | Jamaica | 1992 | 4.84 | 7.08 | 3.10 | 0.55 | 1395283 | 3376967464 | 2429750605 | 697641.5 | 2429052964 | 0.72 |
| Incidence | Jordan | 1992 | 2.96 | 4.49 | 1.84 | 0.55 | 2205439 | 3376967464 | 2431956044 | 1102720 | 2430853325 | 0.72 |

| | | | | | | | | | | | | | |
|---|---|---|---|---|---|---|---|---|---|---|---|---|---|
| Incidence | Armenia | 1992 | 5.75 | 8.59 | 3.67 | 0.55 | 2180135 | 3376967464 | 2434136179 | 1090067 | 2433046112 | 0.72 |
| Incidence | South Africa | 1992 | 1.62 | 2.43 | 0.99 | 0.55 | 22998760 | 3376967464 | 2457134939 | 11499380 | 2445635559 | 0.72 |
| Incidence | Albania | 1992 | 9.11 | 13.03 | 5.99 | 0.55 | 2048832 | 3376967464 | 2459183771 | 1024416 | 2458159355 | 0.73 |
| Incidence | Mauritius | 1992 | 1.60 | 2.45 | 0.98 | 0.55 | 729511 | 3376967464 | 2459913282 | 364755.5 | 2459548527 | 0.73 |
| Incidence | Libya | 1992 | 3.05 | 4.60 | 1.91 | 0.55 | 2388892 | 3376967464 | 2462302174 | 1194446 | 2461107728 | 0.73 |
| Incidence | Panama | 1992 | 7.14 | 9.98 | 4.81 | 0.55 | 1507409 | 3376967464 | 2463809583 | 753704.7 | 2463055878 | 0.73 |
| Incidence | Malaysia | 1992 | 1.34 | 2.04 | 0.82 | 0.56 | 11151630 | 3376967464 | 2474961212 | 5575814 | 2469385397 | 0.73 |
| Incidence | Saudi Arabia | 1992 | 2.61 | 3.96 | 1.63 | 0.56 | 9752944 | 3376967464 | 2484714156 | 4876472 | 2479837684 | 0.73 |
| Incidence | Morocco | 2021 | 3.40 | 4.98 | 2.22 | 0.56 | 24647580 | 5105255047 | 1006149263 | 12323790 | 993825472.4 | 0.19 |
| Incidence | El Salvador | 2021 | 7.26 | 10.26 | 4.80 | 0.56 | 4074428 | 5105255047 | 1010223691 | 2037214 | 1008186477 | 0.20 |
| Incidence | Ghana | 2021 | 1.25 | 1.76 | 0.80 | 0.56 | 20161140 | 5105255047 | 1030384835 | 10080570 | 1020304263 | 0.20 |
| Incidence | Turkmenistan | 1992 | 5.13 | 7.64 | 3.26 | 0.57 | 2146841 | 3376967464 | 2486860997 | 1073420 | 2485787576 | 0.74 |
| Incidence | Cuba | 1992 | 7.11 | 9.95 | 4.90 | 0.57 | 7554381 | 3376967464 | 2494415377 | 3777190 | 2490638187 | 0.74 |
| Incidence | Dominica | 1992 | 5.79 | 8.21 | 3.93 | 0.57 | 41551.19 | 3376967464 | 2494456928 | 20775.6 | 2494436153 | 0.74 |
| Incidence | Democratic People's Republic of Korea | 2021 | 1.40 | 2.16 | 0.85 | 0.57 | 18946650 | 5105255047 | 1049331482 | 9473323 | 1039858159 | 0.20 |
| Incidence | Marshall Islands | 2021 | 1.88 | 2.81 | 1.21 | 0.57 | 36532.79 | 5105255047 | 1049368015 | 18266.39 | 1049349748 | 0.21 |
| Incidence | India | 2021 | 1.63 | 2.48 | 1.00 | 0.58 | 950604900 | 5105255047 | 1999972931 | 475302500 | 1524670473 | 0.30 |
| Incidence | Tuvalu | 2021 | 1.97 | 2.91 | 1.24 | 0.58 | 7770.842 | 5105255047 | 1999980701 | 3885.421 | 1999976816 | 0.39 |
| Incidence | Cook Islands | 1992 | 1.82 | 2.78 | 1.12 | 0.58 | 11553.59 | 3376967464 | 2494468482 | 5776.796 | 2494462705 | 0.74 |
| Incidence | Congo | 2021 | 1.42 | 2.09 | 0.88 | 0.58 | 3289800 | 5105255047 | 2003270502 | 1644900 | 2001625602 | 0.39 |
| Incidence | Eswatini | 2021 | 1.59 | 2.36 | 0.98 | 0.59 | 702572.3 | 5105255047 | 2003973074 | 351286.1 | 2003621788 | 0.39 |
| Incidence | Micronesia (Federated States of) | 2021 | 2.23 | 3.31 | 1.41 | 0.59 | 67172.81 | 5105255047 | 2004040247 | 33586.4 | 2004006661 | 0.39 |
| Incidence | Uruguay | 1992 | 9.92 | 13.44 | 7.04 | 0.59 | 1978724 | 3376967464 | 2496447206 | 989362.1 | 2495457844 | 0.74 |

| | | | | | | | | | | | | |
|---|---|---|---|---|---|---|---|---|---|---|---|---|
| Incidence | Seychelles | 1992 | 1.74 | 2.63 | 1.10 | 0.59 | 45508.48 | 3376967464 | 2496492715 | 22754.24 | 2496469961 | 0.74 |
| Incidence | Samoa | 2021 | 2.26 | 3.42 | 1.43 | 0.59 | 122652 | 5105255047 | 2004162899 | 61325.99 | 2004101573 | 0.39 |
| Incidence | Azerbaijan | 1992 | 5.48 | 8.19 | 3.47 | 0.59 | 4664389 | 3376967464 | 2501157104 | 2332195 | 2498824909 | 0.74 |
| Incidence | Bahrain | 1992 | 3.49 | 5.22 | 2.19 | 0.60 | 353670.8 | 3376967464 | 2501510775 | 176835.4 | 2501333939 | 0.74 |
| Incidence | Kazakhstan | 1992 | 5.89 | 8.71 | 3.72 | 0.60 | 10145610 | 3376967464 | 2511656388 | 5072806 | 2506583581 | 0.74 |
| Incidence | Venezuela (Bolivarian Republic of) | 2021 | 10.46 | 14.42 | 7.39 | 0.60 | 17426450 | 5105255047 | 2021589354 | 8713227 | 2012876126 | 0.39 |
| Incidence | Argentina | 1992 | 9.74 | 13.02 | 7.00 | 0.60 | 20617890 | 3376967464 | 2532274273 | 10308940 | 2521965330 | 0.75 |
| Incidence | Bolivia (Plurinational State of) | 2021 | 8.20 | 11.62 | 5.42 | 0.60 | 7560756 | 5105255047 | 2029150109 | 3780378 | 2025369731 | 0.40 |
| Incidence | Chile | 1992 | 8.61 | 12.02 | 6.06 | 0.60 | 8721318 | 3376967464 | 2540995591 | 4360659 | 2536634932 | 0.75 |
| Incidence | Saint Kitts and Nevis | 1992 | 5.98 | 8.55 | 4.02 | 0.60 | 23875.01 | 3376967464 | 2541019466 | 11937.5 | 2541007529 | 0.75 |
| Incidence | Niue | 1992 | 1.75 | 2.64 | 1.07 | 0.60 | 1295.984 | 3376967464 | 2541020762 | 647.9919 | 2541020114 | 0.75 |
| Incidence | Kyrgyzstan | 2021 | 7.24 | 10.46 | 4.63 | 0.60 | 4248862 | 5105255047 | 2033398972 | 2124431 | 2031274540 | 0.40 |
| Incidence | Egypt | 2021 | 4.16 | 6.07 | 2.67 | 0.61 | 64397140 | 5105255047 | 2097796116 | 32198570 | 2065597544 | 0.40 |
| Incidence | Republic of Moldova | 1992 | 8.98 | 13.05 | 5.91 | 0.61 | 2854706 | 3376967464 | 2543875468 | 1427353 | 2542448115 | 0.75 |
| Incidence | Belize | 2021 | 7.12 | 10.38 | 4.77 | 0.61 | 283479.5 | 5105255047 | 2098079595 | 141739.7 | 2097937856 | 0.41 |
| Incidence | North Macedonia | 1992 | 9.71 | 13.74 | 6.40 | 0.61 | 1323446 | 3376967464 | 2545198915 | 661723.2 | 2544537192 | 0.75 |
| Incidence | Portugal | 1992 | 4.97 | 6.98 | 3.49 | 0.61 | 6780922 | 3376967464 | 2551979837 | 3390461 | 2548589376 | 0.75 |
| Incidence | Namibia | 2021 | 1.41 | 2.15 | 0.85 | 0.62 | 1504481 | 5105255047 | 2099584076 | 752240.5 | 2098831836 | 0.41 |
| Incidence | Mongolia | 2021 | 7.61 | 10.99 | 4.88 | 0.62 | 2105152 | 5105255047 | 2101689228 | 1052576 | 2100636652 | 0.41 |
| Incidence | American Samoa | 1992 | 2.51 | 3.83 | 1.54 | 0.62 | 29749.7 | 3376967464 | 2552009587 | 14874.85 | 2551994712 | 0.76 |
| Incidence | Dominican Republic | 2021 | 6.94 | 9.78 | 4.75 | 0.62 | 7231298 | 5105255047 | 2108920526 | 3615649 | 2105304877 | 0.41 |
| Incidence | Syrian Arab Republic | 2021 | 4.97 | 7.17 | 3.26 | 0.62 | 9352995 | 5105255047 | 2118273521 | 4676498 | 2113597023 | 0.41 |
| Incidence | Antigua and Barbuda | 1992 | 5.90 | 8.49 | 3.99 | 0.62 | 38493.21 | 3376967464 | 2552048080 | 19246.61 | 2552028833 | 0.76 |

| | | | | | | | | | | | | |
|---|---|---|---|---|---|---|---|---|---|---|---|---|
| Incidence | Nauru | 2021 | 2.14 | 3.22 | 1.37 | 0.63 | 6647.105 | 5105255047 | 2118280168 | 3323.553 | 2118276845 | 0.41 |
| Incidence | Tonga | 2021 | 2.38 | 3.64 | 1.50 | 0.63 | 60598.31 | 5105255047 | 2118340767 | 30299.16 | 2118310467 | 0.41 |
| Incidence | Viet Nam | 2021 | 1.41 | 2.17 | 0.86 | 0.63 | 67836850 | 5105255047 | 2186177612 | 33918420 | 2152259189 | 0.42 |
| Incidence | Romania | 1992 | 26.20 | 36.78 | 17.27 | 0.63 | 15484770 | 3376967464 | 2567532852 | 7742386 | 2559790466 | 0.76 |
| Incidence | Palestine | 2021 | 2.46 | 3.64 | 1.55 | 0.63 | 3092703 | 5105255047 | 2189270315 | 1546351 | 2187723963 | 0.43 |
| Incidence | Belarus | 1992 | 9.80 | 14.17 | 6.51 | 0.63 | 6938289 | 3376967464 | 2574471141 | 3469144 | 2571001997 | 0.76 |
| Incidence | Suriname | 2021 | 6.95 | 9.78 | 4.69 | 0.63 | 384154.9 | 5105255047 | 2189654470 | 192077.4 | 2189462392 | 0.43 |
| Incidence | Trinidad and Tobago | 1992 | 5.57 | 8.19 | 3.65 | 0.63 | 745716.6 | 3376967464 | 2575216858 | 372858.3 | 2574843999 | 0.76 |
| Incidence | Gabon | 2021 | 1.79 | 2.62 | 1.14 | 0.63 | 1102224 | 5105255047 | 2190756693 | 551111.8 | 2190205581 | 0.43 |
| Incidence | Paraguay | 2021 | 11.43 | 16.24 | 7.76 | 0.64 | 4680073 | 5105255047 | 2195436766 | 2340037 | 2193096730 | 0.43 |
| Incidence | Saint Vincent and the Grenadines | 2021 | 8.22 | 11.54 | 5.64 | 0.64 | 76552.59 | 5105255047 | 2195513319 | 38276.3 | 2195475043 | 0.43 |
| Incidence | Poland | 1992 | 4.97 | 7.47 | 3.10 | 0.64 | 24967360 | 3376967464 | 2600184220 | 12483680 | 2587700539 | 0.77 |
| Incidence | Serbia | 1992 | 10.91 | 15.12 | 7.28 | 0.64 | 6574516 | 3376967464 | 2606758736 | 3287258 | 2603471478 | 0.77 |
| Incidence | Botswana | 2021 | 1.57 | 2.33 | 0.96 | 0.64 | 1590402 | 5105255047 | 2197103721 | 795200.9 | 2196308520 | 0.43 |
| Incidence | Guyana | 2021 | 9.70 | 12.74 | 6.98 | 0.65 | 501391.8 | 5105255047 | 2197605113 | 250695.9 | 2197354417 | 0.43 |
| Incidence | Maldives | 2021 | 2.33 | 3.52 | 1.45 | 0.65 | 394937.9 | 5105255047 | 2198000050 | 197468.9 | 2197802582 | 0.43 |
| Incidence | Bulgaria | 1992 | 12.38 | 18.09 | 8.08 | 0.65 | 5699135 | 3376967464 | 2612457871 | 2849568 | 2609608303 | 0.77 |
| Incidence | Philippines | 2021 | 1.11 | 1.72 | 0.65 | 0.65 | 73079830 | 5105255047 | 2271079884 | 36539920 | 2234539967 | 0.44 |
| Incidence | Spain | 1992 | 14.62 | 19.68 | 10.25 | 0.65 | 26113210 | 3376967464 | 2638571077 | 13056600 | 2625514474 | 0.78 |
| Incidence | Brazil | 2021 | 8.94 | 12.93 | 5.87 | 0.65 | 150405800 | 5105255047 | 2421485716 | 75202920 | 2346282800 | 0.46 |
| Incidence | Colombia | 2021 | 13.19 | 17.63 | 9.26 | 0.66 | 33609590 | 5105255047 | 2455095302 | 16804790 | 2438290509 | 0.48 |
| Incidence | Indonesia | 2021 | 1.30 | 1.99 | 0.79 | 0.66 | 194150400 | 5105255047 | 2649245676 | 97075190 | 2552170489 | 0.50 |
| Incidence | Ukraine | 1992 | 9.26 | 13.63 | 5.86 | 0.66 | 34804310 | 3376967464 | 2673375387 | 17402160 | 2655973232 | 0.79 |
| Incidence | Equatorial Guinea | 2021 | 1.72 | 2.52 | 1.08 | 0.66 | 893663 | 5105255047 | 2650139339 | 446831.5 | 2649692507 | 0.52 |

| | | | | | | | | | | | | |
|---|---|---|---|---|---|---|---|---|---|---|---|---|
| Incidence | Algeria | 2021 | 3.77 | 5.51 | 2.42 | 0.66 | 28054400 | 5105255047 | 2678193741 | 14027200 | 2664166540 | 0.52 |
| Incidence | Hungary | 1992 | 20.65 | 28.20 | 14.43 | 0.66 | 6927328 | 3376967464 | 2680302715 | 3463664 | 2676839051 | 0.79 |
| Incidence | Qatar | 1992 | 5.23 | 7.84 | 3.33 | 0.66 | 335643.3 | 3376967464 | 2680638358 | 167821.6 | 2680470537 | 0.79 |
| Incidence | Ecuador | 2021 | 10.48 | 13.10 | 8.05 | 0.66 | 11574520 | 5105255047 | 2689768262 | 5787261 | 2683981001 | 0.53 |
| Incidence | Peru | 2021 | 8.07 | 11.35 | 5.38 | 0.66 | 23885430 | 5105255047 | 2713653696 | 11942720 | 2701710979 | 0.53 |
| Incidence | Barbados | 1992 | 7.60 | 10.90 | 5.23 | 0.66 | 162757.9 | 3376967464 | 2680801116 | 81378.93 | 2680719737 | 0.79 |
| Incidence | Uzbekistan | 2021 | 9.54 | 13.86 | 6.28 | 0.66 | 22329360 | 5105255047 | 2735983057 | 11164680 | 2724818377 | 0.53 |
| Incidence | Iraq | 2021 | 2.72 | 4.02 | 1.72 | 0.66 | 26081420 | 5105255047 | 2762064476 | 13040710 | 2749023766 | 0.54 |
| Incidence | Mexico | 2021 | 10.22 | 14.73 | 6.73 | 0.66 | 86646750 | 5105255047 | 2848711229 | 43323380 | 2805387853 | 0.55 |
| Incidence | Georgia | 1992 | 6.60 | 9.88 | 4.13 | 0.67 | 3570243 | 3376967464 | 2684371360 | 1785122 | 2682586238 | 0.79 |
| Incidence | Slovakia | 1992 | 7.36 | 10.26 | 5.09 | 0.67 | 3469779 | 3376967464 | 2687841139 | 1734890 | 2686106249 | 0.80 |
| Incidence | Cyprus | 1992 | 12.60 | 18.29 | 8.53 | 0.67 | 522214.5 | 3376967464 | 2688363353 | 261107.2 | 2688102246 | 0.80 |
| Incidence | Malta | 1992 | 8.23 | 11.45 | 5.69 | 0.67 | 249227.5 | 3376967464 | 2688612581 | 124613.8 | 2688487967 | 0.80 |
| Incidence | Guam | 1992 | 2.30 | 3.54 | 1.41 | 0.67 | 93009.67 | 3376967464 | 2688705591 | 46504.84 | 2688659086 | 0.80 |
| Incidence | Puerto Rico | 1992 | 8.83 | 12.66 | 5.94 | 0.67 | 2339319 | 3376967464 | 2691044910 | 1169660 | 2689875250 | 0.80 |
| Incidence | Cuba | 2021 | 10.62 | 14.90 | 7.35 | 0.67 | 7721598 | 5105255047 | 2856432827 | 3860799 | 2852572028 | 0.56 |
| Incidence | Grenada | 2021 | 9.66 | 14.01 | 6.57 | 0.67 | 71465.1 | 5105255047 | 2856504292 | 35732.55 | 2856468560 | 0.56 |
| Incidence | Kuwait | 1992 | 3.91 | 5.92 | 2.40 | 0.67 | 1142046 | 3376967464 | 2692186956 | 571023.1 | 2691615933 | 0.80 |
| Incidence | United States Virgin Islands | 1992 | 9.22 | 12.85 | 6.04 | 0.67 | 68428.68 | 3376967464 | 2692255385 | 34214.34 | 2692221171 | 0.80 |
| Incidence | Saint Lucia | 2021 | 8.75 | 12.13 | 5.96 | 0.67 | 127254.6 | 5105255047 | 2856631547 | 63627.3 | 2856567920 | 0.56 |
| Incidence | Montenegro | 1992 | 12.21 | 17.35 | 8.05 | 0.67 | 417608.2 | 3376967464 | 2692672993 | 208804.1 | 2692464189 | 0.80 |
| Incidence | Palau | 1992 | 1.96 | 3.00 | 1.21 | 0.67 | 10631.73 | 3376967464 | 2692683625 | 5315.866 | 2692678309 | 0.80 |
| Incidence | Fiji | 2021 | 1.79 | 2.71 | 1.09 | 0.68 | 595530.4 | 5105255047 | 2857227077 | 297765.2 | 2856929312 | 0.56 |
| Incidence | United Arab Emirates | 1992 | 4.31 | 6.30 | 2.74 | 0.68 | 1447269 | 3376967464 | 2694130893 | 723634.3 | 2693407259 | 0.80 |

| | | | | | | | | | | | | |
|---|---|---|---|---|---|---|---|---|---|---|---|---|
| Incidence | Brunei Darussalam | 1992 | 8.95 | 12.79 | 5.97 | 0.68 | 172733 | 3376967464 | 2694303626 | 86366.52 | 2694217260 | 0.80 |
| Incidence | Croatia | 1992 | 13.11 | 17.87 | 9.17 | 0.68 | 3329713 | 3376967464 | 2697633339 | 1664857 | 2695968483 | 0.80 |
| Incidence | South Africa | 2021 | 1.84 | 2.77 | 1.12 | 0.68 | 37983600 | 5105255047 | 2895210682 | 18991800 | 2876218879 | 0.56 |
| Incidence | Lithuania | 1992 | 9.83 | 13.38 | 6.91 | 0.68 | 2447071 | 3376967464 | 2700080410 | 1223536 | 2698856875 | 0.80 |
| Incidence | Turkmenistan | 2021 | 8.61 | 12.52 | 5.52 | 0.68 | 3350351 | 5105255047 | 2898561033 | 1675176 | 2896885857 | 0.57 |
| Incidence | Tunisia | 2021 | 3.93 | 5.71 | 2.53 | 0.68 | 7946659 | 5105255047 | 2906507692 | 3973329 | 2902534362 | 0.57 |
| Incidence | Thailand | 2021 | 1.81 | 2.82 | 1.09 | 0.68 | 47274310 | 5105255047 | 2953782004 | 23637160 | 2930144848 | 0.57 |
| Incidence | Jamaica | 2021 | 6.25 | 9.13 | 4.05 | 0.68 | 1946551 | 5105255047 | 2955728554 | 973275.3 | 2954755279 | 0.58 |
| Incidence | Taiwan (Province of China) | 1992 | 1.77 | 2.75 | 1.07 | 0.68 | 14026120 | 3376967464 | 2714106526 | 7013058 | 2707093468 | 0.80 |
| Incidence | Tokelau | 2021 | 2.11 | 3.22 | 1.31 | 0.69 | 843.5528 | 5105255047 | 2955729398 | 421.7764 | 2955728976 | 0.58 |
| Incidence | Greece | 1992 | 10.27 | 14.30 | 7.10 | 0.69 | 7122789 | 3376967464 | 2721229315 | 3561394 | 2717667921 | 0.80 |
| Incidence | Bahamas | 1992 | 6.15 | 8.73 | 4.15 | 0.69 | 171499.1 | 3376967464 | 2721400814 | 85749.56 | 2721315065 | 0.81 |
| Incidence | Russian Federation | 1992 | 9.52 | 14.04 | 6.08 | 0.69 | 101681600 | 3376967464 | 2823082430 | 50840810 | 2772241622 | 0.82 |
| Incidence | Azerbaijan | 2021 | 9.09 | 13.30 | 5.81 | 0.69 | 7440503 | 5105255047 | 2963169901 | 3720252 | 2959449649 | 0.58 |
| Incidence | Estonia | 1992 | 12.88 | 18.29 | 8.66 | 0.70 | 1018565 | 3376967464 | 2824100995 | 509282.4 | 2823591712 | 0.84 |
| Incidence | Latvia | 1992 | 14.59 | 20.50 | 9.71 | 0.70 | 1746906 | 3376967464 | 2825847901 | 873453 | 2824974448 | 0.84 |
| Incidence | Iran (Islamic Republic of) | 2021 | 3.63 | 5.35 | 2.30 | 0.70 | 59167520 | 5105255047 | 3022337423 | 29583760 | 2992753662 | 0.59 |
| Incidence | Czechia | 1992 | 15.44 | 22.57 | 10.01 | 0.70 | 6884471 | 3376967464 | 2832732372 | 3442236 | 2829290136 | 0.84 |
| Incidence | Costa Rica | 2021 | 15.68 | 20.91 | 11.08 | 0.70 | 3249833 | 5105255047 | 3025587256 | 1624917 | 3023962339 | 0.59 |
| Incidence | Sri Lanka | 2021 | 2.94 | 4.17 | 1.95 | 0.70 | 14714740 | 5105255047 | 3040301997 | 7357370 | 3032944626 | 0.59 |
| Incidence | Armenia | 2021 | 10.62 | 15.26 | 6.98 | 0.70 | 2004496 | 5105255047 | 3042306493 | 1002248 | 3041304245 | 0.60 |
| Incidence | Singapore | 1992 | 9.42 | 13.64 | 6.23 | 0.70 | 2369939 | 3376967464 | 2835102310 | 1184969 | 2833917341 | 0.84 |
| Incidence | Bermuda | 1992 | 16.49 | 23.62 | 10.83 | 0.70 | 42685.4 | 3376967464 | 2835144996 | 21342.7 | 2835123653 | 0.84 |
| Incidence | Albania | 2021 | 15.18 | 21.73 | 10.13 | 0.71 | 1808384 | 5105255047 | 3044114877 | 904192 | 3043210685 | 0.60 |

| | | | | | | | | | | | | |
|---|---|---|---|---|---|---|---|---|---|---|---|---|
| Incidence | Panama | 2021 | 8.88 | 12.50 | 5.98 | 0.71 | 2749507 | 5105255047 | 3046864384 | 1374753 | 3045489630 | 0.60 |
| Incidence | Republic of Korea | 1992 | 8.19 | 11.92 | 5.25 | 0.71 | 31435440 | 3376967464 | 2866580440 | 15717720 | 2850862718 | 0.84 |
| Incidence | Turkey | 2021 | 4.09 | 6.01 | 2.60 | 0.71 | 56921960 | 5105255047 | 3103786340 | 28460980 | 3075325362 | 0.60 |
| Incidence | Northern Mariana Islands | 1992 | 3.19 | 4.81 | 2.02 | 0.72 | 33889.48 | 3376967464 | 2866614329 | 16944.74 | 2866597384 | 0.85 |
| Incidence | Italy | 1992 | 14.97 | 22.11 | 9.68 | 0.72 | 38983200 | 3376967464 | 2905597533 | 19491600 | 2886105931 | 0.85 |
| Incidence | Israel | 1992 | 6.79 | 9.59 | 4.66 | 0.72 | 3155772 | 3376967464 | 2908753305 | 1577886 | 2907175419 | 0.86 |
| Incidence | Mauritius | 2021 | 2.16 | 3.26 | 1.31 | 0.72 | 900058.5 | 5105255047 | 3104686398 | 450029.2 | 3104236369 | 0.61 |
| Incidence | Uruguay | 2021 | 15.18 | 20.82 | 10.41 | 0.72 | 2214747 | 5105255047 | 3106901145 | 1107373 | 3105793772 | 0.61 |
| Incidence | China | 2021 | 1.99 | 3.04 | 1.21 | 0.72 | 967106700 | 5105255047 | 4074007884 | 483553400 | 3590454514 | 0.70 |
| Incidence | Bosnia and Herzegovina | 2021 | 19.70 | 27.67 | 13.44 | 0.72 | 2206873 | 5105255047 | 4076214757 | 1103437 | 4075111320 | 0.80 |
| Incidence | Argentina | 2021 | 12.88 | 17.55 | 8.80 | 0.72 | 30052210 | 5105255047 | 4106266964 | 15026100 | 4091240861 | 0.80 |
| Incidence | American Samoa | 2021 | 2.98 | 4.42 | 1.93 | 0.72 | 31908.17 | 5105255047 | 4106298872 | 15954.09 | 4106282918 | 0.80 |
| Incidence | Kazakhstan | 2021 | 9.80 | 14.34 | 6.32 | 0.73 | 12126520 | 5105255047 | 4118425393 | 6063260 | 4112362132 | 0.81 |
| Incidence | Jordan | 2021 | 3.64 | 5.43 | 2.27 | 0.73 | 8180007 | 5105255047 | 4126605399 | 4090003 | 4122515396 | 0.81 |
| Incidence | Libya | 2021 | 3.64 | 5.31 | 2.39 | 0.73 | 5029432 | 5105255047 | 4131634831 | 2514716 | 4129120115 | 0.81 |
| Incidence | Niue | 2021 | 2.49 | 3.79 | 1.55 | 0.73 | 1099.18 | 5105255047 | 4131635930 | 549.5901 | 4131635381 | 0.81 |
| Incidence | Greenland | 1992 | 10.32 | 14.70 | 6.98 | 0.73 | 38395.59 | 3376967464 | 2908791700 | 19197.79 | 2908772503 | 0.86 |
| Incidence | Seychelles | 2021 | 2.52 | 3.71 | 1.61 | 0.73 | 72957.22 | 5105255047 | 4131708887 | 36478.61 | 4131672409 | 0.81 |
| Incidence | Ireland | 1992 | 13.96 | 19.45 | 9.88 | 0.73 | 2251651 | 3376967464 | 2911043351 | 1125825 | 2909917526 | 0.86 |
| Incidence | Republic of Moldova | 2021 | 13.75 | 19.71 | 9.03 | 0.73 | 2516382 | 5105255047 | 4134225269 | 1258191 | 4132967078 | 0.81 |
| Incidence | Georgia | 2021 | 13.65 | 19.18 | 9.32 | 0.73 | 2299321 | 5105255047 | 4136524590 | 1149660 | 4135374929 | 0.81 |
| Incidence | Australia | 1992 | 9.52 | 13.51 | 6.48 | 0.73 | 11484530 | 3376967464 | 2922527885 | 5742267 | 2916785618 | 0.86 |
| Incidence | Slovenia | 1992 | 23.59 | 32.91 | 16.65 | 0.74 | 1357830 | 3376967464 | 2923885715 | 678914.9 | 2923206800 | 0.87 |
| Incidence | Malaysia | 2021 | 2.05 | 3.11 | 1.27 | 0.74 | 21856660 | 5105255047 | 4158381246 | 10928330 | 4147452918 | 0.81 |

| | | | | | | | | | | | |
|---|---|---|---|---|---|---|---|---|---|---|---|
| Incidence | France | 1992 | 14.46 | 19.57 | 10.32 | 0.74 | 38242170 | 3376967464 | 2962127885 | 19121090 | 2943006800 | 0.87 |
| Incidence | Portugal | 2021 | 8.40 | 11.72 | 5.92 | 0.74 | 6825510 | 5105255047 | 4165206755 | 3412755 | 4161794000 | 0.82 |
| Incidence | Lebanon | 2021 | 4.45 | 6.51 | 2.95 | 0.74 | 3716347 | 5105255047 | 4168923102 | 1858174 | 4167064929 | 0.82 |
| Incidence | Barbados | 2021 | 9.90 | 14.04 | 6.64 | 0.75 | 202783.5 | 5105255047 | 4169125886 | 101391.7 | 4169024494 | 0.82 |
| Incidence | Dominica | 2021 | 9.18 | 13.12 | 6.29 | 0.75 | 46130.66 | 5105255047 | 4169172016 | 23065.33 | 4169148951 | 0.82 |
| Incidence | Belgium | 1992 | 14.62 | 20.49 | 10.11 | 0.75 | 6685776 | 3376967464 | 2968813661 | 3342888 | 2965470773 | 0.88 |
| Incidence | Antigua and Barbuda | 2021 | 8.10 | 11.48 | 5.46 | 0.75 | 63578.22 | 5105255047 | 4169235595 | 31789.11 | 4169203806 | 0.82 |
| Incidence | North Macedonia | 2021 | 16.43 | 23.00 | 11.10 | 0.75 | 1541196 | 5105255047 | 4170776791 | 770598.2 | 4170006193 | 0.82 |
| Incidence | Bahrain | 2021 | 4.42 | 6.58 | 2.79 | 0.75 | 1178377 | 5105255047 | 4171955168 | 589188.4 | 4171365980 | 0.82 |
| Incidence | Palau | 2021 | 2.69 | 4.03 | 1.69 | 0.75 | 13185.18 | 5105255047 | 4171968353 | 6592.589 | 4171961761 | 0.82 |
| Incidence | Austria | 1992 | 27.99 | 34.88 | 22.09 | 0.75 | 5270737 | 3376967464 | 2974084397 | 2635368 | 2971449029 | 0.88 |
| Incidence | United Kingdom | 1992 | 13.64 | 19.19 | 9.36 | 0.75 | 37397090 | 3376967464 | 3011481485 | 18698540 | 2992782941 | 0.89 |
| Incidence | Saint Kitts and Nevis | 2021 | 8.67 | 12.31 | 5.90 | 0.75 | 43420.68 | 5105255047 | 4172011774 | 21710.34 | 4171990064 | 0.82 |
| Incidence | New Zealand | 1992 | 11.63 | 16.81 | 7.52 | 0.76 | 2285123 | 3376967464 | 3013766608 | 1142561 | 3012624047 | 0.89 |
| Incidence | Ukraine | 2021 | 12.66 | 18.48 | 8.08 | 0.76 | 29287980 | 5105255047 | 4201299752 | 14643990 | 4186655763 | 0.82 |
| Incidence | Finland | 1992 | 24.54 | 36.51 | 16.97 | 0.76 | 3388185 | 3376967464 | 3017154793 | 1694093 | 3015460701 | 0.89 |
| Incidence | Andorra | 1992 | 11.41 | 16.32 | 7.45 | 0.77 | 45683.08 | 3376967464 | 3017200476 | 22841.54 | 3017177635 | 0.89 |
| Incidence | Bulgaria | 2021 | 14.31 | 19.72 | 9.50 | 0.77 | 4337506 | 5105255047 | 4205637258 | 2168753 | 4203468505 | 0.82 |
| Incidence | Romania | 2021 | 47.82 | 66.01 | 31.23 | 0.77 | 12142540 | 5105255047 | 4217779801 | 6071271 | 4211708529 | 0.82 |
| Incidence | Trinidad and Tobago | 2021 | 7.60 | 11.16 | 4.91 | 0.77 | 942563.1 | 5105255047 | 4218722364 | 471281.5 | 4218251082 | 0.83 |
| Incidence | Spain | 2021 | 20.22 | 27.50 | 13.89 | 0.77 | 29873650 | 5105255047 | 4248596015 | 14936830 | 4233659190 | 0.83 |
| Incidence | United States of America | 1992 | 16.35 | 23.99 | 10.56 | 0.77 | 169626400 | 3376967464 | 3186826882 | 84813200 | 3102013679 | 0.92 |
| Incidence | Chile | 2021 | 12.89 | 18.20 | 8.76 | 0.77 | 12819950 | 5105255047 | 4261415966 | 6409975 | 4255005991 | 0.83 |
| Incidence | Northern Mariana Islands | 2021 | 3.74 | 5.57 | 2.41 | 0.77 | 33644 | 5105255047 | 4261449610 | 16822 | 4261432788 | 0.83 |

| | | | | | | | | | | | | |
|---|---|---|---|---|---|---|---|---|---|---|---|---|
| Incidence | Iceland | 1992 | 19.59 | 26.96 | 13.65 | 0.77 | 167194.9 | 3376967464 | 3186994077 | 83597.46 | 3186910480 | 0.94 |
| Incidence | Oman | 2021 | 4.28 | 6.15 | 2.84 | 0.77 | 3365550 | 5105255047 | 4264815160 | 1682775 | 4263132385 | 0.84 |
| Incidence | Cook Islands | 2021 | 2.51 | 3.86 | 1.56 | 0.78 | 11633.24 | 5105255047 | 4264826793 | 5816.619 | 4264820977 | 0.84 |
| Incidence | Belarus | 2021 | 14.79 | 21.34 | 9.70 | 0.78 | 6254502 | 5105255047 | 4271081295 | 3127251 | 4267954044 | 0.84 |
| Incidence | Canada | 1992 | 10.46 | 14.39 | 7.19 | 0.79 | 18901940 | 3376967464 | 3205896013 | 9450968 | 3196445045 | 0.95 |
| Incidence | Luxembourg | 1992 | 20.61 | 27.82 | 14.38 | 0.79 | 268190.4 | 3376967464 | 3206164203 | 134095.2 | 3206030108 | 0.95 |
| Incidence | Hungary | 2021 | 36.25 | 48.50 | 25.82 | 0.79 | 6199148 | 5105255047 | 4277280443 | 3099574 | 4274180869 | 0.84 |
| Incidence | Greece | 2021 | 14.04 | 18.95 | 9.98 | 0.79 | 6469340 | 5105255047 | 4283749783 | 3234670 | 4280515113 | 0.84 |
| Incidence | Serbia | 2021 | 21.80 | 30.06 | 15.13 | 0.79 | 5934126 | 5105255047 | 4289683909 | 2967063 | 4286716846 | 0.84 |
| Incidence | Sweden | 1992 | 16.87 | 24.60 | 11.05 | 0.80 | 5553552 | 3376967464 | 3211717756 | 2776776 | 3208940980 | 0.95 |
| Incidence | Montenegro | 2021 | 17.74 | 25.21 | 11.73 | 0.80 | 412912.2 | 5105255047 | 4290096822 | 206456.1 | 4289890365 | 0.84 |
| Incidence | Croatia | 2021 | 36.06 | 48.51 | 25.22 | 0.80 | 2715472 | 5105255047 | 4292812293 | 1357736 | 4291454558 | 0.84 |
| Incidence | Japan | 1992 | 13.60 | 20.00 | 8.78 | 0.80 | 88370150 | 3376967464 | 3300087906 | 44185070 | 3255902831 | 0.96 |
| Incidence | Malta | 2021 | 11.59 | 16.18 | 7.81 | 0.80 | 278137.3 | 5105255047 | 4293090431 | 139068.6 | 4292951362 | 0.84 |
| Incidence | Netherlands | 1992 | 6.46 | 8.76 | 4.55 | 0.80 | 10396300 | 3376967464 | 3310484202 | 5198148 | 3305286054 | 0.98 |
| Incidence | Guam | 2021 | 3.07 | 4.62 | 1.94 | 0.80 | 103477.4 | 5105255047 | 4293193908 | 51738.72 | 4293142170 | 0.84 |
| Incidence | Bahamas | 2021 | 8.24 | 11.38 | 5.59 | 0.81 | 274938.4 | 5105255047 | 4293468847 | 137469.2 | 4293331377 | 0.84 |
| Incidence | Norway | 1992 | 13.29 | 19.11 | 8.84 | 0.81 | 2768970 | 3376967464 | 3313253172 | 1384485 | 3311868687 | 0.98 |
| Incidence | Italy | 2021 | 20.13 | 26.48 | 14.70 | 0.81 | 38186670 | 5105255047 | 4331655515 | 19093330 | 4312562181 | 0.84 |
| Incidence | Denmark | 1992 | 13.74 | 18.98 | 9.46 | 0.81 | 3484450 | 3376967464 | 3316737622 | 1742225 | 3314995397 | 0.98 |
| Incidence | Russian Federation | 2021 | 18.63 | 26.69 | 12.43 | 0.81 | 96028800 | 5105255047 | 4427684315 | 48014400 | 4379669915 | 0.86 |
| Incidence | Israel | 2021 | 8.15 | 11.53 | 5.50 | 0.81 | 5769267 | 5105255047 | 4433453582 | 2884633 | 4430568949 | 0.87 |
| Incidence | Brunei Darussalam | 2021 | 12.85 | 18.21 | 8.64 | 0.81 | 332019.1 | 5105255047 | 4433785601 | 166009.6 | 4433619592 | 0.87 |
| Incidence | Slovakia | 2021 | 16.09 | 22.39 | 11.37 | 0.81 | 3635982 | 5105255047 | 4437421583 | 1817991 | 4435603592 | 0.87 |

| | | | | | | | | | | | | |
|---|---|---|---|---|---|---|---|---|---|---|---|---|
| Incidence | Poland | 2021 | 7.24 | 10.00 | 4.94 | 0.81 | 25179790 | 5105255047 | 4462601375 | 12589900 | 4450011479 | 0.87 |
| Incidence | Saudi Arabia | 2021 | 4.24 | 6.29 | 2.67 | 0.82 | 29117100 | 5105255047 | 4491718473 | 14558550 | 4477159924 | 0.88 |
| Incidence | Bermuda | 2021 | 24.08 | 34.11 | 16.45 | 0.82 | 41969.77 | 5105255047 | 4491760443 | 20984.88 | 4491739458 | 0.88 |
| Incidence | United States Virgin Islands | 2021 | 13.96 | 19.41 | 9.36 | 0.82 | 53866.94 | 5105255047 | 4491814310 | 26933.47 | 4491787376 | 0.88 |
| Incidence | San Marino | 1992 | 11.69 | 17.19 | 7.52 | 0.82 | 16789.73 | 3376967464 | 3316754411 | 8394.865 | 3316746017 | 0.98 |
| Incidence | Puerto Rico | 2021 | 12.10 | 17.07 | 8.23 | 0.83 | 2124836 | 5105255047 | 4493939146 | 1062418 | 4492876728 | 0.88 |
| Incidence | Greenland | 2021 | 14.23 | 20.20 | 9.50 | 0.83 | 39064.81 | 5105255047 | 4493978210 | 19532.41 | 4493958678 | 0.88 |
| Incidence | Germany | 1992 | 18.28 | 24.54 | 13.05 | 0.83 | 55437780 | 3376967464 | 3372192189 | 27718890 | 3344473300 | 0.99 |
| Incidence | Czechia | 2021 | 35.09 | 48.62 | 24.04 | 0.83 | 6708178 | 5105255047 | 4500686388 | 3354089 | 4497332299 | 0.88 |
| Incidence | Latvia | 2021 | 27.01 | 37.68 | 17.99 | 0.83 | 1180840 | 5105255047 | 4501867228 | 590420 | 4501276808 | 0.88 |
| Incidence | Cyprus | 2021 | 14.24 | 20.20 | 9.67 | 0.84 | 940775.7 | 5105255047 | 4502808004 | 470387.8 | 4502337616 | 0.88 |
| Incidence | France | 2021 | 15.73 | 22.33 | 10.27 | 0.84 | 41021790 | 5105255047 | 4543829797 | 20510900 | 4523318900 | 0.89 |
| Incidence | Slovenia | 2021 | 46.79 | 64.12 | 34.16 | 0.84 | 1320405 | 5105255047 | 4545150201 | 660202.5 | 4544489999 | 0.89 |
| Incidence | Australia | 2021 | 12.12 | 17.46 | 8.06 | 0.84 | 16656050 | 5105255047 | 4561806249 | 8328024 | 4553478225 | 0.89 |
| Incidence | Estonia | 2021 | 36.28 | 51.73 | 25.47 | 0.84 | 825205.3 | 5105255047 | 4562631454 | 412602.6 | 4562218851 | 0.89 |
| Incidence | Kuwait | 2021 | 5.22 | 7.72 | 3.25 | 0.85 | 3633714 | 5105255047 | 4566265168 | 1816857 | 4564448311 | 0.89 |
| Incidence | Qatar | 2021 | 5.75 | 8.59 | 3.66 | 0.85 | 2446107 | 5105255047 | 4568711276 | 1223054 | 4567488222 | 0.89 |
| Incidence | United Arab Emirates | 2021 | 5.72 | 8.59 | 3.63 | 0.85 | 8129433 | 5105255047 | 4576840709 | 4064717 | 4572775992 | 0.90 |
| Incidence | New Zealand | 2021 | 14.11 | 19.74 | 9.57 | 0.85 | 3378026 | 5105255047 | 4580218735 | 1689013 | 4578529722 | 0.90 |
| Incidence | Monaco | 1992 | 10.96 | 16.13 | 7.16 | 0.85 | 19942.64 | 3376967464 | 3372212132 | 9971.318 | 3372202160 | 1.00 |
| Incidence | Belgium | 2021 | 18.05 | 25.69 | 12.18 | 0.85 | 7314275 | 5105255047 | 4587533010 | 3657137 | 4583875872 | 0.90 |
| Incidence | Austria | 2021 | 39.05 | 56.55 | 25.65 | 0.85 | 5970418 | 5105255047 | 4593503427 | 2985209 | 4590518219 | 0.90 |
| Incidence | Singapore | 2021 | 10.74 | 15.60 | 7.17 | 0.86 | 4146887 | 5105255047 | 4597650315 | 2073444 | 4595576871 | 0.90 |
| Incidence | Lithuania | 2021 | 20.18 | 27.94 | 14.15 | 0.86 | 1763517 | 5105255047 | 4599413832 | 881758.6 | 4598532074 | 0.90 |

| | | | | | | | | | | | | |
|---|---|---|---|---|---|---|---|---|---|---|---|---|
| Incidence | United Kingdom | 2021 | 14.88 | 20.52 | 10.24 | 0.86 | 43558640 | 5105255047 | 4642972474 | 21779320 | 4621193153 | 0.91 |
| Incidence | Finland | 2021 | 26.47 | 37.47 | 18.14 | 0.86 | 3397926 | 5105255047 | 4646370400 | 1698963 | 4644671437 | 0.91 |
| Incidence | United States of America | 2021 | 17.38 | 23.89 | 11.97 | 0.86 | 216097500 | 5105255047 | 4862467891 | 108048700 | 4754419146 | 0.93 |
| Incidence | Switzerland | 1992 | 20.89 | 28.02 | 14.80 | 0.87 | 4755332 | 3376967464 | 3376967464 | 2377666 | 3374589798 | 1.00 |
| Incidence | Andorra | 2021 | 14.17 | 20.37 | 9.32 | 0.87 | 61742.59 | 5105255047 | 4862529634 | 30871.3 | 4862498763 | 0.95 |
| Incidence | Japan | 2021 | 12.67 | 17.94 | 8.33 | 0.87 | 75404150 | 5105255047 | 4937933788 | 37702080 | 4900231711 | 0.96 |
| Incidence | Canada | 2021 | 14.29 | 20.29 | 9.68 | 0.87 | 24262340 | 5105255047 | 4962196124 | 12131170 | 4950064956 | 0.97 |
| Incidence | Ireland | 2021 | 16.44 | 23.26 | 11.10 | 0.87 | 3192765 | 5105255047 | 4965388890 | 1596383 | 4963792507 | 0.97 |
| Incidence | Taiwan (Province of China) | 2021 | 2.78 | 4.27 | 1.72 | 0.87 | 16681750 | 5105255047 | 4982070642 | 8340876 | 4973729766 | 0.97 |
| Incidence | Iceland | 2021 | 26.10 | 37.07 | 17.71 | 0.88 | 227618.6 | 5105255047 | 4982298260 | 113809.3 | 4982184451 | 0.98 |
| Incidence | Luxembourg | 2021 | 24.43 | 34.27 | 16.19 | 0.88 | 447039.5 | 5105255047 | 4982745300 | 223519.8 | 4982521780 | 0.98 |
| Incidence | Republic of Korea | 2021 | 11.01 | 15.83 | 7.20 | 0.89 | 36999740 | 5105255047 | 5019745039 | 18499870 | 5001245169 | 0.98 |
| Incidence | Sweden | 2021 | 22.29 | 31.91 | 14.35 | 0.89 | 6417083 | 5105255047 | 5026162122 | 3208541 | 5022953580 | 0.98 |
| Incidence | San Marino | 2021 | 13.66 | 19.65 | 9.02 | 0.89 | 21290.2 | 5105255047 | 5026183412 | 10645.1 | 5026172767 | 0.98 |
| Incidence | Netherlands | 2021 | 6.94 | 9.89 | 4.61 | 0.89 | 11069920 | 5105255047 | 5037253336 | 5534962 | 5031718374 | 0.99 |
| Incidence | Denmark | 2021 | 20.96 | 28.96 | 14.32 | 0.90 | 3719018 | 5105255047 | 5040972354 | 1859509 | 5039112845 | 0.99 |
| Incidence | Germany | 2021 | 24.44 | 33.62 | 16.82 | 0.90 | 54851780 | 5105255047 | 5095824131 | 27425890 | 5068398243 | 0.99 |
| Incidence | Monaco | 2021 | 13.33 | 19.54 | 9.00 | 0.91 | 23218.3 | 5105255047 | 5095847350 | 11609.15 | 5095835740 | 1.00 |
| Incidence | Norway | 2021 | 16.25 | 23.49 | 10.50 | 0.92 | 3522494 | 5105255047 | 5099369844 | 1761247 | 5097608597 | 1.00 |
| Incidence | Switzerland | 2021 | 21.37 | 30.54 | 14.10 | 0.93 | 5885204 | 5105255047 | 5105255047 | 2942602 | 5102312446 | 1.00 |
| Incidence | Somalia | 1992 | 0.65 | 0.99 | 0.40 | 0.05 | 3816581 | 3376967464 | 3816581 | 1908290 | 1908290.3 | 0.00 |
| Incidence | Somalia | 2021 | 0.50 | 0.76 | 0.30 | 0.08 | 10890630 | 5105255047 | 10890631 | 5445315 | 5445315.5 | 0.00 |
| Incidence | Niger | 1992 | 0.63 | 0.92 | 0.40 | 0.08 | 4059013 | 3376967464 | 7875594 | 2029507 | 5846087.2 | 0.00 |
| Incidence | Chad | 1992 | 0.63 | 0.91 | 0.40 | 0.12 | 3037794 | 3376967464 | 10913388 | 1518897 | 9394490.9 | 0.00 |

| | | | | | | | | | | | | |
|---|---|---|---|---|---|---|---|---|---|---|---|---|
| Incidence | Mali | 1992 | 0.63 | 0.95 | 0.38 | 0.13 | 4416030 | 3376967464 | 15329418 | 2208015 | 13121403 | 0.00 |
| Incidence | Burkina Faso | 1992 | 0.68 | 0.99 | 0.44 | 0.14 | 4697926 | 3376967464 | 20027344 | 2348963 | 17678381.3 | 0.01 |
| Incidence | Ethiopia | 1992 | 0.61 | 0.93 | 0.36 | 0.15 | 27028040 | 3376967464 | 47055389 | 13514020 | 33541366.7 | 0.01 |
| Incidence | Niger | 2021 | 0.58 | 0.85 | 0.37 | 0.17 | 11701660 | 5105255047 | 22592286 | 5850828 | 16741458.7 | 0.00 |
| Incidence | Mozambique | 1992 | 0.71 | 1.08 | 0.43 | 0.18 | 6841201 | 3376967464 | 53896590 | 3420600 | 50475989.3 | 0.01 |
| Incidence | Afghanistan | 1992 | 1.77 | 2.57 | 1.14 | 0.18 | 5317936 | 3376967464 | 59214526 | 2658968 | 56555557.9 | 0.02 |
| Incidence | Guinea | 1992 | 0.77 | 1.12 | 0.50 | 0.18 | 3194335 | 3376967464 | 62408861 | 1597167 | 60811693.6 | 0.02 |
| Incidence | Uganda | 1992 | 0.61 | 0.94 | 0.37 | 0.19 | 8871780 | 3376967464 | 71280641 | 4435890 | 66844751.1 | 0.02 |
| Incidence | Malawi | 1992 | 0.62 | 0.95 | 0.38 | 0.21 | 5373904 | 3376967464 | 76654545 | 2686952 | 73967593.3 | 0.02 |
| Incidence | Burundi | 1992 | 0.68 | 1.02 | 0.42 | 0.21 | 2868701 | 3376967464 | 79523246 | 1434350 | 78088895.8 | 0.02 |
| Incidence | South Sudan | 1992 | 0.68 | 1.03 | 0.41 | 0.21 | 3170246 | 3376967464 | 82693492 | 1585123 | 81108369 | 0.02 |
| Incidence | Nepal | 1992 | 0.92 | 1.42 | 0.54 | 0.21 | 10843820 | 3376967464 | 93537312 | 5421910 | 88115402.1 | 0.03 |
| Incidence | Sierra Leone | 1992 | 0.67 | 0.98 | 0.43 | 0.21 | 2160724 | 3376967464 | 95698036 | 1080362 | 94617674.1 | 0.03 |
| Incidence | Guinea-Bissau | 1992 | 0.67 | 0.96 | 0.44 | 0.22 | 518730.4 | 3376967464 | 96216766 | 259365.2 | 95957401.1 | 0.03 |
| Incidence | Central African Republic | 1992 | 0.70 | 1.05 | 0.44 | 0.22 | 1511635 | 3376967464 | 97728401 | 755817.3 | 96972583.7 | 0.03 |
| Incidence | Benin | 1992 | 0.67 | 0.98 | 0.43 | 0.23 | 2437709 | 3376967464 | 100166110 | 1218855 | 98947255.7 | 0.03 |
| Incidence | Bhutan | 1992 | 0.87 | 1.34 | 0.51 | 0.23 | 362111 | 3376967464 | 100528221 | 181055.5 | 100347165.9 | 0.03 |
| Incidence | Eritrea | 1992 | 0.63 | 0.94 | 0.39 | 0.23 | 1636011 | 3376967464 | 102164232 | 818005.3 | 101346226.7 | 0.03 |
| Incidence | Yemen | 1992 | 1.39 | 2.04 | 0.89 | 0.23 | 6649314 | 3376967464 | 108813546 | 3324657 | 105488888.8 | 0.03 |
| Incidence | Liberia | 1992 | 0.84 | 1.21 | 0.54 | 0.24 | 970267.7 | 3376967464 | 109783813 | 485133.8 | 109298679.5 | 0.03 |
| Incidence | Chad | 2021 | 0.65 | 0.94 | 0.43 | 0.24 | 8325851 | 5105255047 | 30918137 | 4162925 | 26755211.9 | 0.01 |
| Incidence | Bangladesh | 1992 | 0.82 | 1.27 | 0.49 | 0.25 | 59911290 | 3376967464 | 169695106 | 29955650 | 139739459.7 | 0.04 |
| Incidence | Senegal | 1992 | 0.64 | 0.94 | 0.40 | 0.25 | 3982520 | 3376967464 | 173677626 | 1991260 | 171686366 | 0.05 |
| Incidence | Gambia | 1992 | 0.65 | 0.95 | 0.41 | 0.25 | 540094.8 | 3376967464 | 174217721 | 270047.4 | 173947673.4 | 0.05 |

| | | | | | | | | | | | | |
|---|---|---|---|---|---|---|---|---|---|---|---|---|
| Incidence | United Republic of Tanzania | 1992 | 0.78 | 1.17 | 0.47 | 0.26 | 13815680 | 3376967464 | 188033398 | 6907839 | 181125559.4 | 0.05 |
| Incidence | Mali | 2021 | 0.61 | 0.89 | 0.37 | 0.27 | 11895330 | 5105255047 | 42813466 | 5947664 | 36865801.5 | 0.01 |
| Incidence | Lao People's Democratic Republic | 1992 | 0.54 | 0.84 | 0.31 | 0.27 | 2290031 | 3376967464 | 190323429 | 1145016 | 189178413.7 | 0.06 |
| Incidence | Rwanda | 1992 | 0.74 | 1.09 | 0.47 | 0.28 | 3779329 | 3376967464 | 194102758 | 1889664 | 192213093.7 | 0.06 |
| Incidence | South Sudan | 2021 | 0.73 | 1.12 | 0.44 | 0.28 | 5135653 | 5105255047 | 47949119 | 2567827 | 45381292.2 | 0.01 |
| Incidence | Togo | 1992 | 0.64 | 0.93 | 0.41 | 0.28 | 1939975 | 3376967464 | 196042733 | 969987.5 | 195072745.7 | 0.06 |
| Incidence | Angola | 1992 | 0.69 | 1.04 | 0.42 | 0.28 | 5702682 | 3376967464 | 201745415 | 2851341 | 198894074.2 | 0.06 |
| Incidence | Timor-Leste | 1992 | 0.48 | 0.76 | 0.27 | 0.28 | 447494.9 | 3376967464 | 202192910 | 223747.4 | 201969162.6 | 0.06 |
| Incidence | Madagascar | 1992 | 0.82 | 1.19 | 0.52 | 0.28 | 6432226 | 3376967464 | 208625136 | 3216113 | 205409023.2 | 0.06 |
| Incidence | Equatorial Guinea | 1992 | 0.78 | 1.16 | 0.48 | 0.28 | 229117.5 | 3376967464 | 208854254 | 114558.8 | 208739695 | 0.06 |
| Incidence | Burkina Faso | 2021 | 0.76 | 1.08 | 0.49 | 0.29 | 11698660 | 5105255047 | 59647781 | 5849331 | 53798449.8 | 0.01 |
| Incidence | Cabo Verde | 1992 | 0.74 | 1.08 | 0.46 | 0.29 | 183931.3 | 3376967464 | 209038185 | 91965.67 | 208946219.4 | 0.06 |
| Incidence | Comoros | 1992 | 0.76 | 1.17 | 0.46 | 0.29 | 249116.1 | 3376967464 | 209287301 | 124558 | 209162743.1 | 0.06 |
| Incidence | Burundi | 2021 | 0.61 | 0.92 | 0.36 | 0.29 | 7040681 | 5105255047 | 66688462 | 3520341 | 63168121.4 | 0.01 |
| Incidence | Democratic Republic of the Congo | 1992 | 0.76 | 1.14 | 0.47 | 0.29 | 20457730 | 3376967464 | 229745032 | 10228870 | 219516166.6 | 0.07 |
| Incidence | Coted'Ivoire | 1992 | 0.73 | 1.05 | 0.47 | 0.29 | 6800901 | 3376967464 | 236545933 | 3400450 | 233145482.4 | 0.07 |
| Incidence | Cambodia | 1992 | 0.49 | 0.78 | 0.28 | 0.30 | 5566488 | 3376967464 | 242112421 | 2783244 | 239329176.7 | 0.07 |
| Incidence | Sudan | 1992 | 1.46 | 2.17 | 0.92 | 0.30 | 11202790 | 3376967464 | 253315211 | 5601395 | 247713815.6 | 0.07 |
| Incidence | Zambia | 1992 | 0.64 | 0.97 | 0.38 | 0.31 | 4197849 | 3376967464 | 257513060 | 2098925 | 255414135.1 | 0.08 |
| Incidence | Central African Republic | 2021 | 0.70 | 1.02 | 0.44 | 0.31 | 3075108 | 5105255047 | 69763570 | 1537554 | 68226015.8 | 0.01 |
| Incidence | Nigeria | 1992 | 0.76 | 1.13 | 0.48 | 0.31 | 50478270 | 3376967464 | 307991334 | 25239140 | 282752197 | 0.08 |
| Incidence | Solomon Islands | 1992 | 0.83 | 1.26 | 0.51 | 0.31 | 187733.3 | 3376967464 | 308179068 | 93866.66 | 308085200.9 | 0.09 |

| | | | | | | | | | | | |
|---|---|---|---|---|---|---|---|---|---|---|---|
| Incidence | Sao Tome and Principe | 1992 | 0.69 | 1.04 | 0.42 | 0.31 | 61668.72 | 3376967464 | 308240736 | 30834.36 | 308209902 | 0.09 |
| Incidence | Cameroon | 1992 | 0.83 | 1.20 | 0.54 | 0.32 | 5631620 | 3376967464 | 313872356 | 2815810 | 311056546.2 | 0.09 |
| Incidence | Guatemala | 1992 | 2.91 | 4.26 | 1.86 | 0.32 | 4330417 | 3376967464 | 318202773 | 2165209 | 316037564.7 | 0.09 |
| Incidence | Papua New Guinea | 1992 | 0.71 | 1.10 | 0.42 | 0.32 | 2419850 | 3376967464 | 320622623 | 1209925 | 319412698.1 | 0.09 |
| Incidence | Haiti | 1992 | 2.92 | 4.22 | 1.97 | 0.32 | 3615033 | 3376967464 | 324237656 | 1807516 | 322430139.3 | 0.10 |
| Incidence | Pakistan | 1992 | 1.04 | 1.60 | 0.62 | 0.32 | 60357000 | 3376967464 | 384594651 | 30178500 | 354416153.3 | 0.10 |
| Incidence | Myanmar | 1992 | 0.59 | 0.92 | 0.35 | 0.33 | 24926270 | 3376967464 | 409520920 | 12463130 | 397057785.4 | 0.12 |
| Incidence | Mozambique | 2021 | 0.78 | 1.15 | 0.50 | 0.33 | 16039550 | 5105255047 | 85803118 | 8019774 | 77783343.8 | 0.02 |
| Incidence | Guinea | 2021 | 0.77 | 1.08 | 0.50 | 0.34 | 6958615 | 5105255047 | 92761733 | 3479307 | 89282425.3 | 0.02 |
| Incidence | Afghanistan | 2021 | 1.26 | 1.86 | 0.81 | 0.34 | 16398400 | 5105255047 | 109160135 | 8199201 | 100960934 | 0.02 |
| Incidence | Djibouti | 1992 | 0.64 | 0.97 | 0.39 | 0.34 | 281064.1 | 3376967464 | 409801984 | 140532.1 | 409661452 | 0.12 |
| Incidence | Honduras | 1992 | 3.03 | 4.40 | 1.98 | 0.34 | 2511178 | 3376967464 | 412313162 | 1255589 | 411057573.1 | 0.12 |
| Incidence | India | 1992 | 1.00 | 1.55 | 0.59 | 0.34 | 515839400 | 3376967464 | 928152513 | 257919700 | 670232837.4 | 0.20 |
| Incidence | Kenya | 1992 | 0.63 | 0.96 | 0.37 | 0.35 | 12235470 | 3376967464 | 940387986 | 6117737 | 934270249.4 | 0.28 |
| Incidence | Mauritania | 1992 | 0.80 | 1.16 | 0.51 | 0.35 | 1105278 | 3376967464 | 941493264 | 552639.1 | 940940625.2 | 0.28 |
| Incidence | Lesotho | 1992 | 0.84 | 1.28 | 0.50 | 0.35 | 821010.8 | 3376967464 | 942314275 | 410505.4 | 941903769.7 | 0.28 |
| Incidence | Liberia | 2021 | 0.85 | 1.19 | 0.56 | 0.35 | 3135229 | 5105255047 | 112295364 | 1567614 | 110727749.6 | 0.02 |
| Incidence | Guinea-Bissau | 2021 | 0.73 | 1.03 | 0.49 | 0.35 | 1119372 | 5105255047 | 113414736 | 559686.2 | 112855050.1 | 0.02 |
| Incidence | Nicaragua | 1992 | 3.26 | 4.69 | 2.16 | 0.36 | 2103741 | 3376967464 | 944418017 | 1051871 | 943366145.8 | 0.28 |
| Incidence | Maldives | 1992 | 0.89 | 1.39 | 0.54 | 0.36 | 118284.3 | 3376967464 | 944536301 | 59142.14 | 944477158.7 | 0.28 |
| Incidence | Sierra Leone | 2021 | 0.71 | 1.02 | 0.46 | 0.36 | 5014942 | 5105255047 | 118429679 | 2507471 | 115922207.5 | 0.02 |
| Incidence | Ethiopia | 2021 | 0.55 | 0.84 | 0.32 | 0.36 | 61363610 | 5105255047 | 179793292 | 30681810 | 149111485.3 | 0.03 |
| Incidence | Vanuatu | 1992 | 0.83 | 1.25 | 0.50 | 0.36 | 84103.98 | 3376967464 | 944620405 | 42051.99 | 944578352.8 | 0.28 |
| Incidence | Morocco | 1992 | 1.88 | 2.75 | 1.20 | 0.37 | 15160090 | 3376967464 | 959780492 | 7580044 | 952200448.5 | 0.28 |

| | | | | | | | | | | | | |
|---|---|---|---|---|---|---|---|---|---|---|---|---|
| Incidence | Benin | 2021 | 0.76 | 1.07 | 0.49 | 0.37 | 7048559 | 5105255047 | 186841851 | 3524280 | 183317571.5 | 0.04 |
| Incidence | El Salvador | 1992 | 3.51 | 5.13 | 2.30 | 0.38 | 3000660 | 3376967464 | 962781152 | 1500330 | 961280822.3 | 0.28 |
| Incidence | Democratic Republic of the Congo | 2021 | 0.82 | 1.20 | 0.52 | 0.38 | 49675100 | 5105255047 | 236516952 | 24837550 | 211679401.7 | 0.04 |
| Incidence | Malawi | 2021 | 0.69 | 1.06 | 0.43 | 0.38 | 10785330 | 5105255047 | 247302286 | 5392667 | 241909619.1 | 0.05 |
| Incidence | Ghana | 1992 | 0.74 | 1.09 | 0.47 | 0.38 | 8290370 | 3376967464 | 971071522 | 4145185 | 966926337.2 | 0.29 |
| Incidence | Madagascar | 2021 | 0.87 | 1.26 | 0.55 | 0.40 | 16136660 | 5105255047 | 263438948 | 8068331 | 255370616.9 | 0.05 |
| Incidence | Eritrea | 2021 | 0.66 | 0.99 | 0.41 | 0.40 | 3903951 | 5105255047 | 267342899 | 1951975 | 265390923.2 | 0.05 |
| Incidence | Senegal | 2021 | 0.75 | 1.09 | 0.47 | 0.41 | 8916317 | 5105255047 | 276259216 | 4458159 | 271801057.2 | 0.05 |
| Incidence | Togo | 2021 | 0.89 | 1.25 | 0.59 | 0.41 | 4808399 | 5105255047 | 281067615 | 2404199 | 278663415.3 | 0.05 |
| Incidence | Gambia | 2021 | 0.79 | 1.14 | 0.51 | 0.41 | 1328594 | 5105255047 | 282396209 | 664296.9 | 281731911.6 | 0.06 |
| Incidence | Palestine | 1992 | 1.54 | 2.34 | 0.93 | 0.41 | 1092733 | 3376967464 | 972164255 | 546366.4 | 971617888.3 | 0.29 |
| Incidence | Zimbabwe | 1992 | 0.84 | 1.28 | 0.51 | 0.41 | 5556213 | 3376967464 | 977720467 | 2778106 | 974942361.1 | 0.29 |
| Incidence | Kiribati | 1992 | 0.94 | 1.44 | 0.58 | 0.42 | 43348.47 | 3376967464 | 977763816 | 21674.24 | 977742141.7 | 0.29 |
| Incidence | Eswatini | 1992 | 0.87 | 1.32 | 0.53 | 0.42 | 429553.2 | 3376967464 | 978193369 | 214776.6 | 977978592.6 | 0.29 |
| Incidence | Papua New Guinea | 2021 | 0.91 | 1.38 | 0.54 | 0.42 | 6230283 | 5105255047 | 288626492 | 3115142 | 285511350.2 | 0.06 |
| Incidence | Viet Nam | 1992 | 0.66 | 1.04 | 0.39 | 0.42 | 40268370 | 3376967464 | 1018461738 | 20134180 | 998327553.6 | 0.30 |
| Incidence | Iraq | 1992 | 1.66 | 2.52 | 1.02 | 0.42 | 10043140 | 3376967464 | 1028504876 | 5021569 | 1023483307 | 0.30 |
| Incidence | Tuvalu | 1992 | 1.22 | 1.85 | 0.75 | 0.42 | 5474.875 | 3376967464 | 1028510350 | 2737.437 | 1028507613 | 0.30 |
| Incidence | Uganda | 2021 | 0.69 | 1.03 | 0.42 | 0.42 | 22465620 | 5105255047 | 311092115 | 11232810 | 299859303.5 | 0.06 |
| Incidence | Coted'Ivoire | 2021 | 0.92 | 1.29 | 0.61 | 0.43 | 15562160 | 5105255047 | 326654276 | 7781080 | 318873195.4 | 0.06 |
| Incidence | Solomon Islands | 2021 | 1.17 | 1.80 | 0.72 | 0.43 | 400945.3 | 5105255047 | 327055221 | 200472.7 | 326854748.6 | 0.06 |
| Incidence | Congo | 1992 | 0.83 | 1.26 | 0.52 | 0.43 | 1365818 | 3376967464 | 1029876169 | 682909 | 1029193260 | 0.30 |
| Incidence | Nepal | 2021 | 1.51 | 2.26 | 0.95 | 0.43 | 19987970 | 5105255047 | 347043194 | 9993986 | 337049207.6 | 0.07 |

| | | | | | | | | | | | | |
|---|---|---|---|---|---|---|---|---|---|---|---|---|
| Incidence | Bolivia (Plurinational State of) | 1992 | 3.32 | 4.82 | 2.16 | 0.43 | 3645533 | 3376967464 | 1033521701 | 1822766 | 1031698935 | 0.31 |
| Incidence | Rwanda | 2021 | 0.80 | 1.21 | 0.49 | 0.44 | 7848418 | 5105255047 | 354891612 | 3924209 | 350967402.9 | 0.07 |
| Incidence | Egypt | 1992 | 2.24 | 3.29 | 1.41 | 0.44 | 33112790 | 3376967464 | 1066634488 | 16556390 | 1050078095 | 0.31 |
| Incidence | Botswana | 1992 | 0.83 | 1.25 | 0.51 | 0.44 | 747651.5 | 3376967464 | 1067382140 | 373825.7 | 1067008314 | 0.32 |
| Incidence | Marshall Islands | 1992 | 0.77 | 1.17 | 0.46 | 0.44 | 23628.24 | 3376967464 | 1067405768 | 11814.12 | 1067393954 | 0.32 |
| Incidence | Belize | 1992 | 3.33 | 4.83 | 2.17 | 0.44 | 103326.3 | 3376967464 | 1067509094 | 51663.13 | 1067457431 | 0.32 |
| Incidence | Timor-Leste | 2021 | 0.71 | 1.08 | 0.42 | 0.44 | 802737.4 | 5105255047 | 355694349 | 401368.7 | 355292980.6 | 0.07 |
| Incidence | Syrian Arab Republic | 1992 | 3.10 | 4.50 | 2.04 | 0.45 | 6931728 | 3376967464 | 1074440822 | 3465864 | 1070974958 | 0.32 |
| Incidence | United Republic of Tanzania | 2021 | 0.88 | 1.32 | 0.55 | 0.45 | 32205890 | 5105255047 | 387900240 | 16102950 | 371797294.8 | 0.07 |
| Incidence | Haiti | 2021 | 3.40 | 4.83 | 2.25 | 0.45 | 8005224 | 5105255047 | 395905464 | 4002612 | 391902852.4 | 0.08 |
| Incidence | Dominican Republic | 1992 | 3.08 | 4.50 | 1.98 | 0.45 | 4367623 | 3376967464 | 1078808445 | 2183812 | 1076624634 | 0.32 |
| Incidence | Yemen | 2021 | 1.54 | 2.22 | 1.00 | 0.45 | 18839690 | 5105255047 | 414745156 | 9419846 | 405325310.2 | 0.08 |
| Incidence | Angola | 2021 | 0.84 | 1.24 | 0.52 | 0.45 | 16719890 | 5105255047 | 431465050 | 8359947 | 423105102.9 | 0.08 |
| Incidence | Grenada | 1992 | 4.61 | 6.51 | 3.13 | 0.46 | 48427.92 | 3376967464 | 1078856873 | 24213.96 | 1078832659 | 0.32 |
| Incidence | Oman | 1992 | 1.58 | 2.36 | 0.97 | 0.46 | 1153630 | 3376967464 | 1080010503 | 576815.1 | 1079433688 | 0.32 |
| Incidence | Namibia | 1992 | 0.84 | 1.27 | 0.51 | 0.46 | 804903.9 | 3376967464 | 1080815407 | 402451.9 | 1080412955 | 0.32 |
| Incidence | Guyana | 1992 | 4.98 | 6.80 | 3.47 | 0.47 | 459165.3 | 3376967464 | 1081274573 | 229582.7 | 1081044990 | 0.32 |
| Incidence | Gabon | 1992 | 1.06 | 1.58 | 0.67 | 0.47 | 557425.5 | 3376967464 | 1081831998 | 278712.8 | 1081553285 | 0.32 |
| Incidence | Bhutan | 2021 | 1.65 | 2.50 | 1.01 | 0.47 | 519639.6 | 5105255047 | 431984689 | 259819.8 | 431724869.5 | 0.08 |
| Incidence | Vanuatu | 2021 | 1.30 | 1.95 | 0.80 | 0.47 | 184213.1 | 5105255047 | 432168902 | 92106.55 | 432076795.9 | 0.08 |
| Incidence | Cambodia | 2021 | 0.94 | 1.43 | 0.55 | 0.47 | 10996940 | 5105255047 | 443165844 | 5498471 | 437667373 | 0.09 |
| Incidence | Micronesia (Federated States of) | 1992 | 0.99 | 1.51 | 0.61 | 0.47 | 55679.86 | 3376967464 | 1081887678 | 27839.93 | 1081859838 | 0.32 |
| Incidence | Zimbabwe | 2021 | 0.97 | 1.44 | 0.62 | 0.47 | 8807361 | 5105255047 | 451973205 | 4403681 | 447569524.4 | 0.09 |
| Incidence | Tajikistan | 1992 | 2.99 | 4.45 | 1.91 | 0.48 | 2943035 | 3376967464 | 1084830713 | 1471517 | 1083359195 | 0.32 |

| | | | | | | | | | | | | | |
|---|---|---|---|---|---|---|---|---|---|---|---|---|---|
| Incidence | Indonesia | 1992 | 0.62 | 0.97 | 0.36 | 0.48 | 115716600 | 3376967464 | 1200547263 | 57858280 | 1142688988 | 0.34 |
| Incidence | China | 1992 | 0.94 | 1.46 | 0.55 | 0.48 | 815239200 | 3376967464 | 2015786424 | 407619600 | 1608166844 | 0.48 |
| Incidence | Algeria | 1992 | 2.04 | 3.01 | 1.32 | 0.48 | 14528470 | 3376967464 | 2030314898 | 7264237 | 2023050661 | 0.60 |
| Incidence | Comoros | 2021 | 0.91 | 1.39 | 0.54 | 0.48 | 467233.1 | 5105255047 | 452440438 | 233616.6 | 452206821.7 | 0.09 |
| Incidence | Turkey | 1992 | 2.63 | 3.91 | 1.66 | 0.48 | 36334360 | 3376967464 | 2066649254 | 18167180 | 2048482076 | 0.61 |
| Incidence | Mongolia | 1992 | 3.16 | 4.68 | 2.02 | 0.48 | 1235917 | 3376967464 | 2067885171 | 617958.5 | 2067267212 | 0.61 |
| Incidence | Paraguay | 1992 | 5.03 | 7.03 | 3.37 | 0.48 | 2324234 | 3376967464 | 2070209405 | 1162117 | 2069047288 | 0.61 |
| Incidence | Cameroon | 2021 | 0.98 | 1.38 | 0.66 | 0.48 | 17450670 | 5105255047 | 469891104 | 8725333 | 461165771 | 0.09 |
| Incidence | Iran (Islamic Republic of) | 1992 | 2.10 | 3.11 | 1.31 | 0.48 | 32234900 | 3376967464 | 2102444303 | 16117450 | 2086326854 | 0.62 |
| Incidence | Colombia | 1992 | 5.90 | 8.17 | 4.06 | 0.48 | 20427380 | 3376967464 | 2122871685 | 10213690 | 2112657994 | 0.63 |
| Incidence | Saint Vincent and the Grenadines | 1992 | 3.99 | 5.70 | 2.62 | 0.49 | 63082.62 | 3376967464 | 2122934768 | 31541.31 | 2122903226 | 0.63 |
| Incidence | Djibouti | 2021 | 0.93 | 1.40 | 0.57 | 0.49 | 805751.6 | 5105255047 | 470696855 | 402875.8 | 470293979.5 | 0.09 |
| Incidence | Tunisia | 1992 | 2.42 | 3.58 | 1.54 | 0.49 | 5133459 | 3376967464 | 2128068226 | 2566729 | 2125501497 | 0.63 |
| Incidence | Lao People's Democratic Republic | 2021 | 0.85 | 1.33 | 0.50 | 0.49 | 4754006 | 5105255047 | 475450861 | 2377003 | 473073858.3 | 0.09 |
| Incidence | Bangladesh | 2021 | 1.50 | 2.31 | 0.93 | 0.49 | 107272700 | 5105255047 | 582723583 | 53636360 | 529087222.1 | 0.10 |
| Incidence | Samoa | 1992 | 1.26 | 1.94 | 0.77 | 0.49 | 92361.26 | 3376967464 | 2128160588 | 46180.63 | 2128114407 | 0.63 |
| Incidence | Mauritania | 2021 | 0.91 | 1.30 | 0.57 | 0.50 | 2374531 | 5105255047 | 585098113 | 1187265 | 583910848.2 | 0.11 |
| Incidence | Democratic People's Republic of Korea | 1992 | 1.10 | 1.67 | 0.65 | 0.50 | 13875620 | 3376967464 | 2142036206 | 6937809 | 2135098397 | 0.63 |
| Incidence | Uzbekistan | 1992 | 3.75 | 5.61 | 2.38 | 0.50 | 12096580 | 3376967464 | 2154132781 | 6048288 | 2148084494 | 0.64 |
| Incidence | Nigeria | 2021 | 0.73 | 1.07 | 0.45 | 0.50 | 123377300 | 5105255047 | 708475425 | 61688660 | 646786769.3 | 0.13 |
| Incidence | Tonga | 1992 | 1.39 | 2.10 | 0.85 | 0.50 | 53374.01 | 3376967464 | 2154186155 | 26687 | 2154159468 | 0.64 |
| Incidence | Pakistan | 2021 | 1.30 | 1.97 | 0.80 | 0.50 | 141565600 | 5105255047 | 850041000 | 70782790 | 779258212.8 | 0.15 |

| | | | | | | | | | | | | |
|---|---|---|---|---|---|---|---|---|---|---|---|---|
| Incidence | Sao Tome and Principe | 2021 | 1.00 | 1.44 | 0.65 | 0.51 | 131024.1 | 5105255047 | 850172025 | 65512.04 | 850106512.5 | 0.17 |
| Incidence | Zambia | 2021 | 0.82 | 1.21 | 0.50 | 0.51 | 10789070 | 5105255047 | 860961092 | 5394534 | 855566558.4 | 0.17 |
| Incidence | Brazil | 1992 | 5.82 | 8.39 | 3.80 | 0.51 | 93825900 | 3376967464 | 2248012056 | 46912950 | 2201099106 | 0.65 |
| Incidence | Lesotho | 2021 | 0.98 | 1.45 | 0.62 | 0.51 | 1159988 | 5105255047 | 862121080 | 579994.1 | 861541086.2 | 0.17 |
| Incidence | Suriname | 1992 | 4.20 | 6.07 | 2.80 | 0.51 | 240092.4 | 3376967464 | 2248252148 | 120046.2 | 2248132102 | 0.67 |
| Incidence | Honduras | 2021 | 5.10 | 7.15 | 3.45 | 0.51 | 6325127 | 5105255047 | 868446208 | 3162564 | 865283644 | 0.17 |
| Incidence | Peru | 1992 | 3.39 | 4.90 | 2.22 | 0.51 | 13017280 | 3376967464 | 2261269426 | 6508639 | 2254760787 | 0.67 |
| Incidence | Mexico | 1992 | 4.52 | 6.65 | 2.91 | 0.51 | 51527500 | 3376967464 | 2312796928 | 25763750 | 2287033177 | 0.68 |
| Incidence | Saint Lucia | 1992 | 4.31 | 6.06 | 2.92 | 0.52 | 80360.32 | 3376967464 | 2312877288 | 40180.16 | 2312837108 | 0.68 |
| Incidence | Philippines | 1992 | 0.53 | 0.85 | 0.29 | 0.52 | 37716100 | 3376967464 | 2350593387 | 18858050 | 2331735338 | 0.69 |
| Incidence | Ecuador | 1992 | 4.40 | 6.07 | 3.03 | 0.52 | 6008273 | 3376967464 | 2356601661 | 3004137 | 2353597524 | 0.70 |
| Incidence | Kenya | 2021 | 0.81 | 1.24 | 0.49 | 0.52 | 29742370 | 5105255047 | 898188583 | 14871190 | 883317395.1 | 0.17 |
| Incidence | Nicaragua | 2021 | 6.06 | 8.65 | 3.93 | 0.52 | 4297839 | 5105255047 | 902486421 | 2148919 | 900337502 | 0.18 |
| Incidence | Thailand | 1992 | 0.93 | 1.43 | 0.55 | 0.53 | 39057400 | 3376967464 | 2395659056 | 19528700 | 2376130359 | 0.70 |
| Incidence | Kiribati | 2021 | 1.50 | 2.22 | 0.96 | 0.53 | 74513.75 | 5105255047 | 902560935 | 37256.87 | 902523678.2 | 0.18 |
| Incidence | Kyrgyzstan | 1992 | 4.27 | 6.26 | 2.73 | 0.53 | 2635720 | 3376967464 | 2398294776 | 1317860 | 2396976916 | 0.71 |
| Incidence | Venezuela (Bolivarian Republic of) | 1992 | 5.14 | 7.12 | 3.55 | 0.53 | 11607490 | 3376967464 | 2409902266 | 5803745 | 2404098521 | 0.71 |
| Incidence | Tokelau | 1992 | 1.39 | 2.10 | 0.86 | 0.53 | 834.3087 | 3376967464 | 2409903100 | 417.1543 | 2409902683 | 0.71 |
| Incidence | Sri Lanka | 1992 | 1.46 | 2.14 | 0.91 | 0.53 | 11121210 | 3376967464 | 2421024309 | 5560605 | 2415463705 | 0.72 |
| Incidence | Cabo Verde | 2021 | 1.36 | 1.93 | 0.89 | 0.53 | 382357.4 | 5105255047 | 902943292 | 191178.7 | 902752113.8 | 0.18 |
| Incidence | Myanmar | 2021 | 1.15 | 1.76 | 0.69 | 0.53 | 36996570 | 5105255047 | 939939864 | 18498290 | 921441578.5 | 0.18 |
| Incidence | Nauru | 1992 | 1.16 | 1.78 | 0.70 | 0.54 | 5770.117 | 3376967464 | 2421030079 | 2885.058 | 2421027194 | 0.72 |
| Incidence | Bosnia and Herzegovina | 1992 | 12.19 | 16.97 | 8.04 | 0.54 | 3112775 | 3376967464 | 2424142855 | 1556388 | 2422586467 | 0.72 |

| | | | | | | | | | | | | |
|---|---|---|---|---|---|---|---|---|---|---|---|---|
| Incidence | Guatemala | 2021 | 4.54 | 6.53 | 3.03 | 0.54 | 9913414 | 5105255047 | 949853279 | 4956707 | 944896571.5 | 0.19 |
| Incidence | Tajikistan | 2021 | 4.56 | 6.71 | 2.93 | 0.54 | 6207893 | 5105255047 | 956061172 | 3103947 | 952957225.2 | 0.19 |
| Incidence | Lebanon | 1992 | 3.16 | 4.62 | 2.03 | 0.54 | 1853583 | 3376967464 | 2425996438 | 926791.5 | 2425069646 | 0.72 |
| Incidence | Sudan | 2021 | 1.93 | 2.83 | 1.24 | 0.54 | 25440510 | 5105255047 | 981501682 | 12720260 | 968781427 | 0.19 |
| Incidence | Fiji | 1992 | 0.93 | 1.43 | 0.55 | 0.54 | 465040.9 | 3376967464 | 2426461479 | 232520.5 | 2426228958 | 0.72 |
| Incidence | Costa Rica | 1992 | 6.55 | 9.15 | 4.55 | 0.54 | 1893843 | 3376967464 | 2428355322 | 946921.6 | 2427408400 | 0.72 |
| Incidence | Jamaica | 1992 | 3.62 | 5.30 | 2.31 | 0.55 | 1395283 | 3376967464 | 2429750605 | 697641.5 | 2429052964 | 0.72 |
| Incidence | Jordan | 1992 | 1.92 | 2.90 | 1.20 | 0.55 | 2205439 | 3376967464 | 2431956044 | 1102720 | 2430853325 | 0.72 |
| Incidence | Armenia | 1992 | 5.77 | 8.55 | 3.73 | 0.55 | 2180135 | 3376967464 | 2434136179 | 1090067 | 2433046112 | 0.72 |
| Incidence | South Africa | 1992 | 1.23 | 1.83 | 0.76 | 0.55 | 22998760 | 3376967464 | 2457134939 | 11499380 | 2445635559 | 0.72 |
| Incidence | Albania | 1992 | 7.37 | 10.55 | 4.84 | 0.55 | 2048832 | 3376967464 | 2459183771 | 1024416 | 2458159355 | 0.73 |
| Incidence | Mauritius | 1992 | 1.23 | 1.89 | 0.75 | 0.55 | 729511 | 3376967464 | 2459913282 | 364755.5 | 2459548527 | 0.73 |
| Incidence | Libya | 1992 | 2.11 | 3.18 | 1.33 | 0.55 | 2388892 | 3376967464 | 2462302174 | 1194446 | 2461107728 | 0.73 |
| Incidence | Panama | 1992 | 5.16 | 7.26 | 3.45 | 0.55 | 1507409 | 3376967464 | 2463809583 | 753704.7 | 2463055878 | 0.73 |
| Incidence | Malaysia | 1992 | 0.91 | 1.41 | 0.54 | 0.56 | 11151630 | 3376967464 | 2474961212 | 5575814 | 2469385397 | 0.73 |
| Incidence | Saudi Arabia | 1992 | 1.69 | 2.58 | 1.04 | 0.56 | 9752944 | 3376967464 | 2484714156 | 4876472 | 2479837684 | 0.73 |
| Incidence | Morocco | 2021 | 3.47 | 5.09 | 2.27 | 0.56 | 24647580 | 5105255047 | 1006149263 | 12323790 | 993825472.4 | 0.19 |
| Incidence | El Salvador | 2021 | 6.63 | 9.38 | 4.38 | 0.56 | 4074428 | 5105255047 | 1010223691 | 2037214 | 1008186477 | 0.20 |
| Incidence | Ghana | 2021 | 0.96 | 1.36 | 0.63 | 0.56 | 20161140 | 5105255047 | 1030384835 | 10080570 | 1020304263 | 0.20 |
| Incidence | Turkmenistan | 1992 | 3.63 | 5.40 | 2.31 | 0.57 | 2146841 | 3376967464 | 2486860997 | 1073420 | 2485787576 | 0.74 |
| Incidence | Cuba | 1992 | 6.20 | 8.70 | 4.26 | 0.57 | 7554381 | 3376967464 | 2494415377 | 3777190 | 2490638187 | 0.74 |
| Incidence | Dominica | 1992 | 4.65 | 6.60 | 3.15 | 0.57 | 41551.19 | 3376967464 | 2494456928 | 20775.6 | 2494436153 | 0.74 |
| Incidence | Democratic People's Republic of Korea | 2021 | 1.70 | 2.62 | 1.04 | 0.57 | 18946650 | 5105255047 | 1049331482 | 9473323 | 1039858159 | 0.20 |

| | | | | | | | | | | | | |
|---|---|---|---|---|---|---|---|---|---|---|---|---|
| Incidence | Marshall Islands | 2021 | 1.56 | 2.34 | 0.99 | 0.57 | 36532.79 | 5105255047 | 1049368015 | 18266.39 | 1049349748 | 0.21 |
| Incidence | India | 2021 | 1.46 | 2.23 | 0.89 | 0.58 | 950604900 | 5105255047 | 1999972931 | 475302500 | 1524670473 | 0.30 |
| Incidence | Tuvalu | 2021 | 1.87 | 2.76 | 1.19 | 0.58 | 7770.842 | 5105255047 | 1999980701 | 3885.421 | 1999976816 | 0.39 |
| Incidence | Cook Islands | 1992 | 1.53 | 2.33 | 0.94 | 0.58 | 11553.59 | 3376967464 | 2494468482 | 5776.796 | 2494462705 | 0.74 |
| Incidence | Congo | 2021 | 1.08 | 1.62 | 0.66 | 0.58 | 3289800 | 5105255047 | 2003270502 | 1644900 | 2001625602 | 0.39 |
| Incidence | Eswatini | 2021 | 1.13 | 1.68 | 0.70 | 0.59 | 702572.3 | 5105255047 | 2003973074 | 351286.1 | 2003621788 | 0.39 |
| Incidence | Micronesia (Federated States of) | 2021 | 2.15 | 3.18 | 1.36 | 0.59 | 67172.81 | 5105255047 | 2004040247 | 33586.4 | 2004006661 | 0.39 |
| Incidence | Uruguay | 1992 | 11.20 | 15.12 | 8.02 | 0.59 | 1978724 | 3376967464 | 2496447206 | 989362.1 | 2495457844 | 0.74 |
| Incidence | Seychelles | 1992 | 1.34 | 2.02 | 0.85 | 0.59 | 45508.48 | 3376967464 | 2496492715 | 22754.24 | 2496469961 | 0.74 |
| Incidence | Samoa | 2021 | 1.98 | 3.01 | 1.25 | 0.59 | 122652 | 5105255047 | 2004162899 | 61325.99 | 2004101573 | 0.39 |
| Incidence | Azerbaijan | 1992 | 4.76 | 7.08 | 3.05 | 0.59 | 4664389 | 3376967464 | 2501157104 | 2332195 | 2498824909 | 0.74 |
| Incidence | Bahrain | 1992 | 2.22 | 3.30 | 1.39 | 0.60 | 353670.8 | 3376967464 | 2501510775 | 176835.4 | 2501333939 | 0.74 |
| Incidence | Kazakhstan | 1992 | 5.39 | 7.91 | 3.45 | 0.60 | 10145610 | 3376967464 | 2511656388 | 5072806 | 2506583581 | 0.74 |
| Incidence | Venezuela (Bolivarian Republic of) | 2021 | 12.22 | 16.83 | 8.66 | 0.60 | 17426450 | 5105255047 | 2021589354 | 8713227 | 2012876126 | 0.39 |
| Incidence | Argentina | 1992 | 9.76 | 13.03 | 7.03 | 0.60 | 20617890 | 3376967464 | 2532274273 | 10308940 | 2521965330 | 0.75 |
| Incidence | Bolivia (Plurinational State of) | 2021 | 7.04 | 10.01 | 4.64 | 0.60 | 7560756 | 5105255047 | 2029150109 | 3780378 | 2025369731 | 0.40 |
| Incidence | Chile | 1992 | 7.17 | 10.02 | 5.03 | 0.60 | 8721318 | 3376967464 | 2540995591 | 4360659 | 2536634932 | 0.75 |
| Incidence | Saint Kitts and Nevis | 1992 | 4.37 | 6.25 | 2.95 | 0.60 | 23875.01 | 3376967464 | 2541019466 | 11937.5 | 2541007529 | 0.75 |
| Incidence | Niue | 1992 | 1.73 | 2.62 | 1.06 | 0.60 | 1295.984 | 3376967464 | 2541020762 | 647.9919 | 2541020114 | 0.75 |
| Incidence | Kyrgyzstan | 2021 | 6.74 | 9.76 | 4.31 | 0.60 | 4248862 | 5105255047 | 2033398972 | 2124431 | 2031274540 | 0.40 |
| Incidence | Egypt | 2021 | 3.60 | 5.27 | 2.31 | 0.61 | 64397140 | 5105255047 | 2097796116 | 32198570 | 2065597544 | 0.40 |
| Incidence | Republic of Moldova | 1992 | 9.63 | 13.89 | 6.41 | 0.61 | 2854706 | 3376967464 | 2543875468 | 1427353 | 2542448115 | 0.75 |
| Incidence | Belize | 2021 | 5.83 | 8.54 | 3.88 | 0.61 | 283479.5 | 5105255047 | 2098079595 | 141739.7 | 2097937856 | 0.41 |

| | | | | | | | | | | | | |
|---|---|---|---|---|---|---|---|---|---|---|---|---|
| Incidence | North Macedonia | 1992 | 10.13 | 14.30 | 6.71 | 0.61 | 1323446 | 3376967464 | 2545198915 | 661723.2 | 2544537192 | 0.75 |
| Incidence | Portugal | 1992 | 5.81 | 8.13 | 4.13 | 0.61 | 6780922 | 3376967464 | 2551979837 | 3390461 | 2548589376 | 0.75 |
| Incidence | Namibia | 2021 | 1.12 | 1.70 | 0.67 | 0.62 | 1504481 | 5105255047 | 2099584076 | 752240.5 | 2098831836 | 0.41 |
| Incidence | Mongolia | 2021 | 7.44 | 10.79 | 4.74 | 0.62 | 2105152 | 5105255047 | 2101689228 | 1052576 | 2100636652 | 0.41 |
| Incidence | American Samoa | 1992 | 1.64 | 2.53 | 1.00 | 0.62 | 29749.7 | 3376967464 | 2552009587 | 14874.85 | 2551994712 | 0.76 |
| Incidence | Dominican Republic | 2021 | 6.42 | 9.07 | 4.38 | 0.62 | 7231298 | 5105255047 | 2108920526 | 3615649 | 2105304877 | 0.41 |
| Incidence | Syrian Arab Republic | 2021 | 5.44 | 7.87 | 3.56 | 0.62 | 9352995 | 5105255047 | 2118273521 | 4676498 | 2113597023 | 0.41 |
| Incidence | Antigua and Barbuda | 1992 | 4.48 | 6.47 | 3.02 | 0.62 | 38493.21 | 3376967464 | 2552048080 | 19246.61 | 2552028833 | 0.76 |
| Incidence | Nauru | 2021 | 1.54 | 2.32 | 0.98 | 0.63 | 6647.105 | 5105255047 | 2118280168 | 3323.553 | 2118276845 | 0.41 |
| Incidence | Tonga | 2021 | 2.12 | 3.25 | 1.32 | 0.63 | 60598.31 | 5105255047 | 2118340767 | 30299.16 | 2118310467 | 0.41 |
| Incidence | Viet Nam | 2021 | 1.57 | 2.40 | 0.95 | 0.63 | 67836850 | 5105255047 | 2186177612 | 33918420 | 2152259189 | 0.42 |
| Incidence | Romania | 1992 | 31.64 | 44.12 | 21.00 | 0.63 | 15484770 | 3376967464 | 2567532852 | 7742386 | 2559790466 | 0.76 |
| Incidence | Palestine | 2021 | 1.90 | 2.82 | 1.20 | 0.63 | 3092703 | 5105255047 | 2189270315 | 1546351 | 2187723963 | 0.43 |
| Incidence | Belarus | 1992 | 12.18 | 17.44 | 8.24 | 0.63 | 6938289 | 3376967464 | 2574471141 | 3469144 | 2571001997 | 0.76 |
| Incidence | Suriname | 2021 | 8.04 | 11.29 | 5.45 | 0.63 | 384154.9 | 5105255047 | 2189654470 | 192077.4 | 2189462392 | 0.43 |
| Incidence | Trinidad and Tobago | 1992 | 4.33 | 6.38 | 2.82 | 0.63 | 745716.6 | 3376967464 | 2575216858 | 372858.3 | 2574843999 | 0.76 |
| Incidence | Gabon | 2021 | 1.43 | 2.10 | 0.91 | 0.63 | 1102224 | 5105255047 | 2190756693 | 551111.8 | 2190205581 | 0.43 |
| Incidence | Paraguay | 2021 | 9.93 | 14.12 | 6.74 | 0.64 | 4680073 | 5105255047 | 2195436766 | 2340037 | 2193096730 | 0.43 |
| Incidence | Saint Vincent and the Grenadines | 2021 | 10.02 | 14.04 | 6.91 | 0.64 | 76552.59 | 5105255047 | 2195513319 | 38276.3 | 2195475043 | 0.43 |
| Incidence | Poland | 1992 | 5.40 | 8.10 | 3.39 | 0.64 | 24967360 | 3376967464 | 2600184220 | 12483680 | 2587700539 | 0.77 |
| Incidence | Serbia | 1992 | 13.62 | 18.71 | 9.17 | 0.64 | 6574516 | 3376967464 | 2606758736 | 3287258 | 2603471478 | 0.77 |
| Incidence | Botswana | 2021 | 1.30 | 1.94 | 0.80 | 0.64 | 1590402 | 5105255047 | 2197103721 | 795200.9 | 2196308520 | 0.43 |
| Incidence | Guyana | 2021 | 9.42 | 12.37 | 6.77 | 0.65 | 501391.8 | 5105255047 | 2197605113 | 250695.9 | 2197354417 | 0.43 |
| Incidence | Maldives | 2021 | 1.73 | 2.65 | 1.05 | 0.65 | 394937.9 | 5105255047 | 2198000050 | 197468.9 | 2197802582 | 0.43 |

| | | | | | | | | | | | | | | |
|---|---|---|---|---|---|---|---|---|---|---|---|---|---|---|
| Incidence | Bulgaria | 1992 | 16.16 | 23.55 | 10.60 | 0.65 | 5699135 | 3376967464 | 2612457871 | 2849568 | 2609608303 | 0.77 |
| Incidence | Philippines | 2021 | 0.97 | 1.50 | 0.57 | 0.65 | 73079830 | 5105255047 | 2271079884 | 36539920 | 2234539967 | 0.44 |
| Incidence | Spain | 1992 | 17.23 | 23.04 | 12.20 | 0.65 | 26113210 | 3376967464 | 2638571077 | 13056600 | 2625514474 | 0.78 |
| Incidence | Brazil | 2021 | 9.79 | 14.16 | 6.43 | 0.65 | 150405800 | 5105255047 | 2421485716 | 75202920 | 2346282800 | 0.46 |
| Incidence | Colombia | 2021 | 13.96 | 18.60 | 9.82 | 0.66 | 33609590 | 5105255047 | 2455095302 | 16804790 | 2438290509 | 0.48 |
| Incidence | Indonesia | 2021 | 1.31 | 2.02 | 0.79 | 0.66 | 194150400 | 5105255047 | 2649245676 | 97075190 | 2552170489 | 0.50 |
| Incidence | Ukraine | 1992 | 11.93 | 17.41 | 7.68 | 0.66 | 34804310 | 3376967464 | 2673375387 | 17402160 | 2655973232 | 0.79 |
| Incidence | Equatorial Guinea | 2021 | 0.94 | 1.39 | 0.58 | 0.66 | 893663 | 5105255047 | 2650139339 | 446831.5 | 2649692507 | 0.52 |
| Incidence | Algeria | 2021 | 3.66 | 5.34 | 2.34 | 0.66 | 28054400 | 5105255047 | 2678193741 | 14027200 | 2664166540 | 0.52 |
| Incidence | Hungary | 1992 | 25.32 | 34.40 | 17.83 | 0.66 | 6927328 | 3376967464 | 2680302715 | 3463664 | 2676839051 | 0.79 |
| Incidence | Qatar | 1992 | 3.09 | 4.68 | 1.94 | 0.66 | 335643.3 | 3376967464 | 2680638358 | 167821.6 | 2680470537 | 0.79 |
| Incidence | Ecuador | 2021 | 9.64 | 12.08 | 7.39 | 0.66 | 11574520 | 5105255047 | 2689768262 | 5787261 | 2683981001 | 0.53 |
| Incidence | Peru | 2021 | 7.53 | 10.60 | 5.00 | 0.66 | 23885430 | 5105255047 | 2713653696 | 11942720 | 2701710979 | 0.53 |
| Incidence | Barbados | 1992 | 6.52 | 9.35 | 4.47 | 0.66 | 162757.9 | 3376967464 | 2680801116 | 81378.93 | 2680719737 | 0.79 |
| Incidence | Uzbekistan | 2021 | 9.36 | 13.62 | 6.15 | 0.66 | 22329360 | 5105255047 | 2735983057 | 11164680 | 2724818377 | 0.53 |
| Incidence | Iraq | 2021 | 2.22 | 3.30 | 1.39 | 0.66 | 26081420 | 5105255047 | 2762064476 | 13040710 | 2749023766 | 0.54 |
| Incidence | Mexico | 2021 | 10.31 | 14.86 | 6.79 | 0.66 | 86646750 | 5105255047 | 2848711229 | 43323380 | 2805387853 | 0.55 |
| Incidence | Georgia | 1992 | 7.74 | 11.46 | 4.92 | 0.67 | 3570243 | 3376967464 | 2684371360 | 1785122 | 2682586238 | 0.79 |
| Incidence | Slovakia | 1992 | 7.67 | 10.66 | 5.33 | 0.67 | 3469779 | 3376967464 | 2687841139 | 1734890 | 2686106249 | 0.80 |
| Incidence | Cyprus | 1992 | 12.32 | 17.88 | 8.34 | 0.67 | 522214.5 | 3376967464 | 2688363353 | 261107.2 | 2688102246 | 0.80 |
| Incidence | Malta | 1992 | 8.79 | 12.25 | 6.05 | 0.67 | 249227.5 | 3376967464 | 2688612581 | 124613.8 | 2688487967 | 0.80 |
| Incidence | Guam | 1992 | 1.71 | 2.65 | 1.04 | 0.67 | 93009.67 | 3376967464 | 2688705591 | 46504.84 | 2688659086 | 0.80 |
| Incidence | Puerto Rico | 1992 | 8.49 | 12.18 | 5.70 | 0.67 | 2339319 | 3376967464 | 2691044910 | 1169660 | 2689875250 | 0.80 |
| Incidence | Cuba | 2021 | 15.87 | 22.22 | 11.03 | 0.67 | 7721598 | 5105255047 | 2856432827 | 3860799 | 2852572028 | 0.56 |

| | | | | | | | | | | | | | |
|---|---|---|---|---|---|---|---|---|---|---|---|---|---|
| Incidence | Grenada | 2021 | 11.07 | 16.07 | 7.53 | 0.67 | 71465.1 | 5105255047 | 2856504292 | 35732.55 | 2856468560 | 0.56 |
| Incidence | Kuwait | 1992 | 2.63 | 3.95 | 1.63 | 0.67 | 1142046 | 3376967464 | 2692186956 | 571023.1 | 2691615933 | 0.80 |
| Incidence | United States Virgin Islands | 1992 | 9.16 | 12.85 | 5.96 | 0.67 | 68428.68 | 3376967464 | 2692255385 | 34214.34 | 2692221171 | 0.80 |
| Incidence | Saint Lucia | 2021 | 10.97 | 15.18 | 7.49 | 0.67 | 127254.6 | 5105255047 | 2856631547 | 63627.3 | 2856567920 | 0.56 |
| Incidence | Montenegro | 1992 | 13.21 | 18.66 | 8.77 | 0.67 | 417608.2 | 3376967464 | 2692672993 | 208804.1 | 2692464189 | 0.80 |
| Incidence | Palau | 1992 | 1.39 | 2.13 | 0.85 | 0.67 | 10631.73 | 3376967464 | 2692683625 | 5315.866 | 2692678309 | 0.80 |
| Incidence | Fiji | 2021 | 1.83 | 2.78 | 1.12 | 0.68 | 595530.4 | 5105255047 | 2857227077 | 297765.2 | 2856929312 | 0.56 |
| Incidence | United Arab Emirates | 1992 | 2.57 | 3.75 | 1.64 | 0.68 | 1447269 | 3376967464 | 2694130893 | 723634.3 | 2693407259 | 0.80 |
| Incidence | Brunei Darussalam | 1992 | 5.09 | 7.36 | 3.37 | 0.68 | 172733 | 3376967464 | 2694303626 | 86366.52 | 2694217260 | 0.80 |
| Incidence | Croatia | 1992 | 16.74 | 22.64 | 11.81 | 0.68 | 3329713 | 3376967464 | 2697633339 | 1664857 | 2695968483 | 0.80 |
| Incidence | South Africa | 2021 | 1.70 | 2.55 | 1.04 | 0.68 | 37983600 | 5105255047 | 2895210682 | 18991800 | 2876218879 | 0.56 |
| Incidence | Lithuania | 1992 | 11.74 | 15.89 | 8.33 | 0.68 | 2447071 | 3376967464 | 2700080410 | 1223536 | 2698856875 | 0.80 |
| Incidence | Turkmenistan | 2021 | 8.38 | 12.20 | 5.37 | 0.68 | 3350351 | 5105255047 | 2898561033 | 1675176 | 2896885857 | 0.57 |
| Incidence | Tunisia | 2021 | 4.45 | 6.45 | 2.87 | 0.68 | 7946659 | 5105255047 | 2906507692 | 3973329 | 2902534362 | 0.57 |
| Incidence | Thailand | 2021 | 2.71 | 4.20 | 1.66 | 0.68 | 47274310 | 5105255047 | 2953782004 | 23637160 | 2930144848 | 0.57 |
| Incidence | Jamaica | 2021 | 6.34 | 9.26 | 4.11 | 0.68 | 1946551 | 5105255047 | 2955728554 | 973275.3 | 2954755279 | 0.58 |
| Incidence | Taiwan (Province of China) | 1992 | 1.51 | 2.33 | 0.92 | 0.68 | 14026120 | 3376967464 | 2714106526 | 7013058 | 2707093468 | 0.80 |
| Incidence | Tokelau | 2021 | 2.24 | 3.41 | 1.40 | 0.69 | 843.5528 | 5105255047 | 2955729398 | 421.7764 | 2955728976 | 0.58 |
| Incidence | Greece | 1992 | 13.21 | 18.23 | 9.26 | 0.69 | 7122789 | 3376967464 | 2721229315 | 3561394 | 2717667921 | 0.80 |
| Incidence | Bahamas | 1992 | 4.32 | 6.18 | 2.89 | 0.69 | 171499.1 | 3376967464 | 2721400814 | 85749.56 | 2721315065 | 0.81 |
| Incidence | Russian Federation | 1992 | 11.43 | 16.68 | 7.42 | 0.69 | 101681600 | 3376967464 | 2823082430 | 50840810 | 2772241622 | 0.82 |
| Incidence | Azerbaijan | 2021 | 10.57 | 15.42 | 6.79 | 0.69 | 7440503 | 5105255047 | 2963169901 | 3720252 | 2959449649 | 0.58 |
| Incidence | Estonia | 1992 | 16.48 | 23.19 | 11.26 | 0.70 | 1018565 | 3376967464 | 2824100995 | 509282.4 | 2823591712 | 0.84 |
| Incidence | Latvia | 1992 | 18.88 | 26.28 | 12.71 | 0.70 | 1746906 | 3376967464 | 2825847901 | 873453 | 2824974448 | 0.84 |

| | | | | | | | | | | | | |
|---|---|---|---|---|---|---|---|---|---|---|---|---|
| Incidence | Iran (Islamic Republic of) | 2021 | 3.68 | 5.41 | 2.34 | 0.70 | 59167520 | 5105255047 | 3022337423 | 29583760 | 2992753662 | 0.59 |
| Incidence | Czechia | 1992 | 17.54 | 25.63 | 11.40 | 0.70 | 6884471 | 3376967464 | 2832732372 | 3442236 | 2829290136 | 0.84 |
| Incidence | Costa Rica | 2021 | 17.20 | 22.86 | 12.19 | 0.70 | 3249833 | 5105255047 | 3025587256 | 1624917 | 3023962339 | 0.59 |
| Incidence | Sri Lanka | 2021 | 3.51 | 4.96 | 2.34 | 0.70 | 14714740 | 5105255047 | 3040301997 | 7357370 | 3032944626 | 0.59 |
| Incidence | Armenia | 2021 | 13.96 | 19.91 | 9.28 | 0.70 | 2004496 | 5105255047 | 3042306493 | 1002248 | 3041304245 | 0.60 |
| Incidence | Singapore | 1992 | 7.60 | 10.99 | 5.03 | 0.70 | 2369939 | 3376967464 | 2835102310 | 1184969 | 2833917341 | 0.84 |
| Incidence | Bermuda | 1992 | 16.35 | 23.45 | 10.74 | 0.70 | 42685.4 | 3376967464 | 2835144996 | 21342.7 | 2835123653 | 0.84 |
| Incidence | Albania | 2021 | 20.95 | 29.81 | 14.15 | 0.71 | 1808384 | 5105255047 | 3044114877 | 904192 | 3043210685 | 0.60 |
| Incidence | Panama | 2021 | 8.99 | 12.65 | 6.06 | 0.71 | 2749507 | 5105255047 | 3046864384 | 1374753 | 3045489630 | 0.60 |
| Incidence | Republic of Korea | 1992 | 6.76 | 9.87 | 4.32 | 0.71 | 31435440 | 3376967464 | 2866580440 | 15717720 | 2850862718 | 0.84 |
| Incidence | Turkey | 2021 | 4.47 | 6.57 | 2.83 | 0.71 | 56921960 | 5105255047 | 3103786340 | 28460980 | 3075325362 | 0.60 |
| Incidence | Northern Mariana Islands | 1992 | 1.90 | 2.83 | 1.22 | 0.72 | 33889.48 | 3376967464 | 2866614329 | 16944.74 | 2866597384 | 0.85 |
| Incidence | Italy | 1992 | 18.61 | 27.40 | 12.10 | 0.72 | 38983200 | 3376967464 | 2905597533 | 19491600 | 2886105931 | 0.85 |
| Incidence | Israel | 1992 | 6.16 | 8.68 | 4.23 | 0.72 | 3155772 | 3376967464 | 2908753305 | 1577886 | 2907175419 | 0.86 |
| Incidence | Mauritius | 2021 | 2.84 | 4.27 | 1.74 | 0.72 | 900058.5 | 5105255047 | 3104686398 | 450029.2 | 3104236369 | 0.61 |
| Incidence | Uruguay | 2021 | 18.86 | 25.74 | 12.96 | 0.72 | 2214747 | 5105255047 | 3106901145 | 1107373 | 3105793772 | 0.61 |
| Incidence | China | 2021 | 2.76 | 4.21 | 1.68 | 0.72 | 967106700 | 5105255047 | 4074007884 | 483553400 | 3590454514 | 0.70 |
| Incidence | Bosnia and Herzegovina | 2021 | 30.08 | 41.96 | 20.75 | 0.72 | 2206873 | 5105255047 | 4076214757 | 1103437 | 4075111320 | 0.80 |
| Incidence | Argentina | 2021 | 13.59 | 18.49 | 9.30 | 0.72 | 30052210 | 5105255047 | 4106266964 | 15026100 | 4091240861 | 0.80 |
| Incidence | American Samoa | 2021 | 3.38 | 5.02 | 2.17 | 0.72 | 31908.17 | 5105255047 | 4106298872 | 15954.09 | 4106282918 | 0.80 |
| Incidence | Kazakhstan | 2021 | 10.74 | 15.72 | 6.94 | 0.73 | 12126520 | 5105255047 | 4118425393 | 6063260 | 4112362132 | 0.81 |
| Incidence | Jordan | 2021 | 3.07 | 4.60 | 1.91 | 0.73 | 8180007 | 5105255047 | 4126605399 | 4090003 | 4122515396 | 0.81 |
| Incidence | Libya | 2021 | 3.42 | 5.00 | 2.23 | 0.73 | 5029432 | 5105255047 | 4131634831 | 2514716 | 4129120115 | 0.81 |
| Incidence | Niue | 2021 | 3.31 | 5.00 | 2.09 | 0.73 | 1099.18 | 5105255047 | 4131635930 | 549.5901 | 4131635381 | 0.81 |

| | | | | | | | | | | | | |
|---|---|---|---|---|---|---|---|---|---|---|---|---|
| Incidence | Greenland | 1992 | 8.39 | 12.00 | 5.64 | 0.73 | 38395.59 | 3376967464 | 2908791700 | 19197.79 | 2908772503 | 0.86 |
| Incidence | Seychelles | 2021 | 3.07 | 4.50 | 1.96 | 0.73 | 72957.22 | 5105255047 | 4131708887 | 36478.61 | 4131672409 | 0.81 |
| Incidence | Ireland | 1992 | 13.63 | 18.95 | 9.66 | 0.73 | 2251651 | 3376967464 | 2911043351 | 1125825 | 2909917526 | 0.86 |
| Incidence | Republic of Moldova | 2021 | 19.12 | 27.26 | 12.67 | 0.73 | 2516382 | 5105255047 | 4134225269 | 1258191 | 4132967078 | 0.81 |
| Incidence | Georgia | 2021 | 19.69 | 27.36 | 13.61 | 0.73 | 2299321 | 5105255047 | 4136524590 | 1149660 | 4135374929 | 0.81 |
| Incidence | Australia | 1992 | 9.54 | 13.49 | 6.51 | 0.73 | 11484530 | 3376967464 | 2922527885 | 5742267 | 2916785618 | 0.86 |
| Incidence | Slovenia | 1992 | 28.05 | 38.85 | 20.01 | 0.74 | 1357830 | 3376967464 | 2923885715 | 678914.9 | 2923206800 | 0.87 |
| Incidence | Malaysia | 2021 | 1.89 | 2.86 | 1.17 | 0.74 | 21856660 | 5105255047 | 4158381246 | 10928330 | 4147452918 | 0.81 |
| Incidence | France | 1992 | 16.76 | 22.51 | 12.08 | 0.74 | 38242170 | 3376967464 | 2962127885 | 19121090 | 2943006800 | 0.87 |
| Incidence | Portugal | 2021 | 12.84 | 17.85 | 9.13 | 0.74 | 6825510 | 5105255047 | 4165206755 | 3412755 | 4161794000 | 0.82 |
| Incidence | Lebanon | 2021 | 4.28 | 6.25 | 2.85 | 0.74 | 3716347 | 5105255047 | 4168923102 | 1858174 | 4167064929 | 0.82 |
| Incidence | Barbados | 2021 | 14.13 | 19.91 | 9.56 | 0.75 | 202783.5 | 5105255047 | 4169125886 | 101391.7 | 4169024494 | 0.82 |
| Incidence | Dominica | 2021 | 11.15 | 15.90 | 7.69 | 0.75 | 46130.66 | 5105255047 | 4169172016 | 23065.33 | 4169148951 | 0.82 |
| Incidence | Belgium | 1992 | 18.27 | 25.42 | 12.78 | 0.75 | 6685776 | 3376967464 | 2968813661 | 3342888 | 2965470773 | 0.88 |
| Incidence | Antigua and Barbuda | 2021 | 9.58 | 13.57 | 6.47 | 0.75 | 63578.22 | 5105255047 | 4169235595 | 31789.11 | 4169203806 | 0.82 |
| Incidence | North Macedonia | 2021 | 21.79 | 30.38 | 14.80 | 0.75 | 1541196 | 5105255047 | 4170776791 | 770598.2 | 4170006193 | 0.82 |
| Incidence | Bahrain | 2021 | 4.03 | 6.05 | 2.51 | 0.75 | 1178377 | 5105255047 | 4171955168 | 589188.4 | 4171365980 | 0.82 |
| Incidence | Palau | 2021 | 3.77 | 5.65 | 2.36 | 0.75 | 13185.18 | 5105255047 | 4171968353 | 6592.589 | 4171961761 | 0.82 |
| Incidence | Austria | 1992 | 32.05 | 39.67 | 25.54 | 0.75 | 5270737 | 3376967464 | 2974084397 | 2635368 | 2971449029 | 0.88 |
| Incidence | United Kingdom | 1992 | 15.90 | 22.33 | 10.97 | 0.75 | 37397090 | 3376967464 | 3011481485 | 18698540 | 2992782941 | 0.89 |
| Incidence | Saint Kitts and Nevis | 2021 | 11.00 | 15.54 | 7.53 | 0.75 | 43420.68 | 5105255047 | 4172011774 | 21710.34 | 4171990064 | 0.82 |
| Incidence | New Zealand | 1992 | 11.52 | 16.63 | 7.48 | 0.76 | 2285123 | 3376967464 | 3013766608 | 1142561 | 3012624047 | 0.89 |
| Incidence | Ukraine | 2021 | 18.47 | 26.85 | 11.88 | 0.76 | 29287980 | 5105255047 | 4201299752 | 14643990 | 4186655763 | 0.82 |
| Incidence | Finland | 1992 | 28.93 | 43.11 | 20.09 | 0.76 | 3388185 | 3376967464 | 3017154793 | 1694093 | 3015460701 | 0.89 |

| | | | | | | | | | | | | |
|---|---|---|---|---|---|---|---|---|---|---|---|---|
| Incidence | Andorra | 1992 | 10.35 | 14.81 | 6.77 | 0.77 | 45683.08 | 3376967464 | 3017200476 | 22841.54 | 3017177635 | 0.89 |
| Incidence | Bulgaria | 2021 | 21.00 | 28.75 | 14.02 | 0.77 | 4337506 | 5105255047 | 4205637258 | 2168753 | 4203468505 | 0.82 |
| Incidence | Romania | 2021 | 68.66 | 93.87 | 45.36 | 0.77 | 12142540 | 5105255047 | 4217779801 | 6071271 | 4211708529 | 0.82 |
| Incidence | Trinidad and Tobago | 2021 | 9.52 | 13.93 | 6.17 | 0.77 | 942563.1 | 5105255047 | 4218722364 | 471281.5 | 4218251082 | 0.83 |
| Incidence | Spain | 2021 | 31.11 | 42.14 | 21.42 | 0.77 | 29873650 | 5105255047 | 4248596015 | 14936830 | 4233659190 | 0.83 |
| Incidence | United States of America | 1992 | 16.24 | 23.78 | 10.54 | 0.77 | 169626400 | 3376967464 | 3186826882 | 84813200 | 3102013679 | 0.92 |
| Incidence | Chile | 2021 | 15.60 | 21.96 | 10.64 | 0.77 | 12819950 | 5105255047 | 4261415966 | 6409975 | 4255005991 | 0.83 |
| Incidence | Northern Mariana Islands | 2021 | 4.80 | 7.22 | 3.06 | 0.77 | 33644 | 5105255047 | 4261449610 | 16822 | 4261432788 | 0.83 |
| Incidence | Iceland | 1992 | 19.00 | 26.01 | 13.35 | 0.77 | 167194.9 | 3376967464 | 3186994077 | 83597.46 | 3186910480 | 0.94 |
| Incidence | Oman | 2021 | 3.00 | 4.38 | 1.95 | 0.77 | 3365550 | 5105255047 | 4264815160 | 1682775 | 4263132385 | 0.84 |
| Incidence | Cook Islands | 2021 | 3.53 | 5.39 | 2.24 | 0.78 | 11633.24 | 5105255047 | 4264826793 | 5816.619 | 4264820977 | 0.84 |
| Incidence | Belarus | 2021 | 22.42 | 32.11 | 14.90 | 0.78 | 6254502 | 5105255047 | 4271081295 | 3127251 | 4267954044 | 0.84 |
| Incidence | Canada | 1992 | 10.63 | 14.61 | 7.34 | 0.79 | 18901940 | 3376967464 | 3205896013 | 9450968 | 3196445045 | 0.95 |
| Incidence | Luxembourg | 1992 | 25.18 | 33.71 | 17.76 | 0.79 | 268190.4 | 3376967464 | 3206164203 | 134095.2 | 3206030108 | 0.95 |
| Incidence | Hungary | 2021 | 51.98 | 69.16 | 37.32 | 0.79 | 6199148 | 5105255047 | 4277280443 | 3099574 | 4274180869 | 0.84 |
| Incidence | Greece | 2021 | 21.57 | 28.93 | 15.44 | 0.79 | 6469340 | 5105255047 | 4283749783 | 3234670 | 4280515113 | 0.84 |
| Incidence | Serbia | 2021 | 30.46 | 41.60 | 21.44 | 0.79 | 5934126 | 5105255047 | 4289683909 | 2967063 | 4286716846 | 0.84 |
| Incidence | Sweden | 1992 | 20.02 | 29.11 | 13.14 | 0.80 | 5553552 | 3376967464 | 3211717756 | 2776776 | 3208940980 | 0.95 |
| Incidence | Montenegro | 2021 | 24.06 | 34.06 | 16.04 | 0.80 | 412912.2 | 5105255047 | 4290096822 | 206456.1 | 4289890365 | 0.84 |
| Incidence | Croatia | 2021 | 56.46 | 75.48 | 39.91 | 0.80 | 2715472 | 5105255047 | 4292812293 | 1357736 | 4291454558 | 0.84 |
| Incidence | Japan | 1992 | 17.02 | 25.01 | 10.99 | 0.80 | 88370150 | 3376967464 | 3300087906 | 44185070 | 3255902831 | 0.96 |
| Incidence | Malta | 2021 | 17.01 | 23.62 | 11.61 | 0.80 | 278137.3 | 5105255047 | 4293090431 | 139068.6 | 4292951362 | 0.84 |
| Incidence | Netherlands | 1992 | 6.85 | 9.29 | 4.85 | 0.80 | 10396300 | 3376967464 | 3310484202 | 5198148 | 3305286054 | 0.98 |
| Incidence | Guam | 2021 | 3.84 | 5.79 | 2.44 | 0.80 | 103477.4 | 5105255047 | 4293193908 | 51738.72 | 4293142170 | 0.84 |

| | | | | | | | | | | | | | |
|---|---|---|---|---|---|---|---|---|---|---|---|---|---|
| Incidence | Bahamas | 2021 | 9.17 | 12.66 | 6.22 | 0.81 | 274938.4 | 5105255047 | 4293468847 | 137469.2 | 4293331377 | 0.84 |
| Incidence | Norway | 1992 | 14.16 | 20.34 | 9.46 | 0.81 | 2768970 | 3376967464 | 3313253172 | 1384485 | 3311868687 | 0.98 |
| Incidence | Italy | 2021 | 32.93 | 43.10 | 24.16 | 0.81 | 38186670 | 5105255047 | 4331655515 | 19093330 | 4312562181 | 0.84 |
| Incidence | Denmark | 1992 | 15.43 | 21.30 | 10.64 | 0.81 | 3484450 | 3376967464 | 3316737622 | 1742225 | 3314995397 | 0.98 |
| Incidence | Russian Federation | 2021 | 26.88 | 38.38 | 18.07 | 0.81 | 96028800 | 5105255047 | 4427684315 | 48014400 | 4379669915 | 0.86 |
| Incidence | Israel | 2021 | 8.79 | 12.40 | 5.94 | 0.81 | 5769267 | 5105255047 | 4433453582 | 2884633 | 4430568949 | 0.87 |
| Incidence | Brunei Darussalam | 2021 | 11.55 | 16.45 | 7.71 | 0.81 | 332019.1 | 5105255047 | 4433785601 | 166009.6 | 4433619592 | 0.87 |
| Incidence | Slovakia | 2021 | 22.21 | 30.80 | 15.75 | 0.81 | 3635982 | 5105255047 | 4437421583 | 1817991 | 4435603592 | 0.87 |
| Incidence | Poland | 2021 | 10.17 | 13.94 | 7.03 | 0.81 | 25179790 | 5105255047 | 4462601375 | 12589900 | 4450011479 | 0.87 |
| Incidence | Saudi Arabia | 2021 | 3.36 | 5.02 | 2.09 | 0.82 | 29117100 | 5105255047 | 4491718473 | 14558550 | 4477159924 | 0.88 |
| Incidence | Bermuda | 2021 | 39.97 | 56.43 | 27.49 | 0.82 | 41969.77 | 5105255047 | 4491760443 | 20984.88 | 4491739458 | 0.88 |
| Incidence | United States Virgin Islands | 2021 | 23.53 | 32.48 | 15.88 | 0.82 | 53866.94 | 5105255047 | 4491814310 | 26933.47 | 4491787376 | 0.88 |
| Incidence | San Marino | 1992 | 13.16 | 19.29 | 8.50 | 0.82 | 16789.73 | 3376967464 | 3316754411 | 8394.865 | 3316746017 | 0.98 |
| Incidence | Puerto Rico | 2021 | 17.42 | 24.37 | 12.00 | 0.83 | 2124836 | 5105255047 | 4493939146 | 1062418 | 4492876728 | 0.88 |
| Incidence | Greenland | 2021 | 20.29 | 28.64 | 13.58 | 0.83 | 39064.81 | 5105255047 | 4493978210 | 19532.41 | 4493958678 | 0.88 |
| Incidence | Germany | 1992 | 23.34 | 31.19 | 16.75 | 0.83 | 55437780 | 3376967464 | 3372192189 | 27718890 | 3344473300 | 0.99 |
| Incidence | Czechia | 2021 | 49.97 | 69.16 | 34.23 | 0.83 | 6708178 | 5105255047 | 4500686388 | 3354089 | 4497332299 | 0.88 |
| Incidence | Latvia | 2021 | 43.15 | 59.70 | 29.02 | 0.83 | 1180840 | 5105255047 | 4501867228 | 590420 | 4501276808 | 0.88 |
| Incidence | Cyprus | 2021 | 17.56 | 24.86 | 11.99 | 0.84 | 940775.7 | 5105255047 | 4502808004 | 470387.8 | 4502337616 | 0.88 |
| Incidence | France | 2021 | 23.13 | 32.58 | 15.20 | 0.84 | 41021790 | 5105255047 | 4543829797 | 20510900 | 4523318900 | 0.89 |
| Incidence | Slovenia | 2021 | 77.16 | 104.52 | 57.34 | 0.84 | 1320405 | 5105255047 | 4545150201 | 660202.5 | 4544489999 | 0.89 |
| Incidence | Australia | 2021 | 16.12 | 23.09 | 10.82 | 0.84 | 16656050 | 5105255047 | 4561806249 | 8328024 | 4553478225 | 0.89 |
| Incidence | Estonia | 2021 | 54.44 | 76.96 | 38.76 | 0.84 | 825205.3 | 5105255047 | 4562631454 | 412602.6 | 4562218851 | 0.89 |
| Incidence | Kuwait | 2021 | 4.68 | 6.98 | 2.89 | 0.85 | 3633714 | 5105255047 | 4566265168 | 1816857 | 4564448311 | 0.89 |

| | | | | | | | | | | | | |
|---|---|---|---|---|---|---|---|---|---|---|---|---|
| Incidence | Qatar | 2021 | 3.92 | 5.90 | 2.44 | 0.85 | 2446107 | 5105255047 | 4568711276 | 1223054 | 4567488222 | 0.89 |
| Incidence | United Arab Emirates | 2021 | 5.65 | 8.64 | 3.59 | 0.85 | 8129433 | 5105255047 | 4576840709 | 4064717 | 4572775992 | 0.90 |
| Incidence | New Zealand | 2021 | 18.86 | 26.33 | 12.85 | 0.85 | 3378026 | 5105255047 | 4580218735 | 1689013 | 4578529722 | 0.90 |
| Incidence | Monaco | 1992 | 15.89 | 23.20 | 10.47 | 0.85 | 19942.64 | 3376967464 | 3372212132 | 9971.318 | 3372202160 | 1.00 |
| Incidence | Belgium | 2021 | 27.06 | 38.25 | 18.44 | 0.85 | 7314275 | 5105255047 | 4587533010 | 3657137 | 4583875872 | 0.90 |
| Incidence | Austria | 2021 | 58.65 | 84.97 | 38.64 | 0.85 | 5970418 | 5105255047 | 4593503427 | 2985209 | 4590518219 | 0.90 |
| Incidence | Singapore | 2021 | 14.10 | 20.42 | 9.44 | 0.86 | 4146887 | 5105255047 | 4597650315 | 2073444 | 4595576871 | 0.90 |
| Incidence | Lithuania | 2021 | 32.75 | 44.84 | 23.30 | 0.86 | 1763517 | 5105255047 | 4599413832 | 881758.6 | 4598532074 | 0.90 |
| Incidence | United Kingdom | 2021 | 20.97 | 28.82 | 14.49 | 0.86 | 43558640 | 5105255047 | 4642972474 | 21779320 | 4621193153 | 0.91 |
| Incidence | Finland | 2021 | 39.84 | 55.91 | 27.62 | 0.86 | 3397926 | 5105255047 | 4646370400 | 1698963 | 4644671437 | 0.91 |
| Incidence | United States of America | 2021 | 24.51 | 33.51 | 16.98 | 0.86 | 216097500 | 5105255047 | 4862467891 | 108048700 | 4754419146 | 0.93 |
| Incidence | Switzerland | 1992 | 23.88 | 31.95 | 16.95 | 0.87 | 4755332 | 3376967464 | 3376967464 | 2377666 | 3374589798 | 1.00 |
| Incidence | Andorra | 2021 | 21.17 | 30.47 | 13.89 | 0.87 | 61742.59 | 5105255047 | 4862529634 | 30871.3 | 4862498763 | 0.95 |
| Incidence | Japan | 2021 | 18.78 | 26.51 | 12.36 | 0.87 | 75404150 | 5105255047 | 4937933788 | 37702080 | 4900231711 | 0.96 |
| Incidence | Canada | 2021 | 20.90 | 29.44 | 14.33 | 0.87 | 24262340 | 5105255047 | 4962196124 | 12131170 | 4950064956 | 0.97 |
| Incidence | Ireland | 2021 | 21.52 | 30.38 | 14.56 | 0.87 | 3192765 | 5105255047 | 4965388890 | 1596383 | 4963792507 | 0.97 |
| Incidence | Taiwan (Province of China) | 2021 | 4.24 | 6.43 | 2.67 | 0.87 | 16681750 | 5105255047 | 4982070642 | 8340876 | 4973729766 | 0.97 |
| Incidence | Iceland | 2021 | 35.10 | 49.49 | 24.06 | 0.88 | 227618.6 | 5105255047 | 4982298260 | 113809.3 | 4982184451 | 0.98 |
| Incidence | Luxembourg | 2021 | 32.98 | 46.22 | 21.86 | 0.88 | 447039.5 | 5105255047 | 4982745300 | 223519.8 | 4982521780 | 0.98 |
| Incidence | Republic of Korea | 2021 | 16.88 | 24.09 | 11.16 | 0.89 | 36999740 | 5105255047 | 5019745039 | 18499870 | 5001245169 | 0.98 |
| Incidence | Sweden | 2021 | 30.85 | 44.12 | 19.90 | 0.89 | 6417083 | 5105255047 | 5026162122 | 3208541 | 5022953580 | 0.98 |
| Incidence | San Marino | 2021 | 21.40 | 30.77 | 14.14 | 0.89 | 21290.2 | 5105255047 | 5026183412 | 10645.1 | 5026172767 | 0.98 |
| Incidence | Netherlands | 2021 | 10.41 | 14.81 | 6.94 | 0.89 | 11069920 | 5105255047 | 5037253336 | 5534962 | 5031718374 | 0.99 |
| Incidence | Denmark | 2021 | 30.28 | 41.64 | 20.82 | 0.90 | 3719018 | 5105255047 | 5040972354 | 1859509 | 5039112845 | 0.99 |

| | | | | | | | | | | | | | |
|---|---|---|---|---|---|---|---|---|---|---|---|---|---|
| Incidence | Germany | 2021 | 40.22 | 55.03 | 27.92 | 0.90 | 54851780 | 5105255047 | 5095824131 | 27425890 | 5068398243 | 0.99 |
| Incidence | Monaco | 2021 | 23.07 | 33.68 | 15.76 | 0.91 | 23218.3 | 5105255047 | 5095847350 | 11609.15 | 5095835740 | 1.00 |
| Incidence | Norway | 2021 | 22.25 | 32.11 | 14.42 | 0.92 | 3522494 | 5105255047 | 5099369844 | 1761247 | 5097608597 | 1.00 |
| Incidence | Switzerland | 2021 | 31.57 | 44.97 | 20.88 | 0.93 | 5885204 | 5105255047 | 5105255047 | 2942602 | 5102312446 | 1.00 |
| Prevalence | Somalia | 1992 | 6.43 | 9.65 | 4.08 | 0.05 | 3816581 | 3376967464 | 3816581 | 1908290 | 1908290.3 | 0.00 |
| Prevalence | Somalia | 2021 | 5.74 | 8.53 | 3.69 | 0.08 | 10890630 | 5105255047 | 10890631 | 5445315 | 5445315.5 | 0.00 |
| Prevalence | Niger | 1992 | 7.08 | 9.98 | 4.77 | 0.08 | 4059013 | 3376967464 | 7875594 | 2029507 | 5846087.2 | 0.00 |
| Prevalence | Chad | 1992 | 6.62 | 9.31 | 4.51 | 0.12 | 3037794 | 3376967464 | 10913388 | 1518897 | 9394490.9 | 0.00 |
| Prevalence | Mali | 1992 | 6.50 | 9.33 | 4.25 | 0.13 | 4416030 | 3376967464 | 15329418 | 2208015 | 13121403 | 0.00 |
| Prevalence | Burkina Faso | 1992 | 7.11 | 9.95 | 4.84 | 0.14 | 4697926 | 3376967464 | 20027344 | 2348963 | 17678381.3 | 0.01 |
| Prevalence | Ethiopia | 1992 | 6.32 | 9.37 | 4.07 | 0.15 | 27028040 | 3376967464 | 47055389 | 13514020 | 33541366.7 | 0.01 |
| Prevalence | Niger | 2021 | 7.17 | 10.05 | 4.96 | 0.17 | 11701660 | 5105255047 | 22592286 | 5850828 | 16741458.7 | 0.00 |
| Prevalence | Mozambique | 1992 | 6.84 | 10.17 | 4.38 | 0.18 | 6841201 | 3376967464 | 53896590 | 3420600 | 50475989.3 | 0.01 |
| Prevalence | Afghanistan | 1992 | 15.19 | 21.39 | 10.57 | 0.18 | 5317936 | 3376967464 | 59214526 | 2658968 | 56555557.9 | 0.02 |
| Prevalence | Guinea | 1992 | 7.66 | 10.62 | 5.31 | 0.18 | 3194335 | 3376967464 | 62408861 | 1597167 | 60811693.6 | 0.02 |
| Prevalence | Uganda | 1992 | 7.25 | 10.79 | 4.69 | 0.19 | 8871780 | 3376967464 | 71280641 | 4435890 | 66844751.1 | 0.02 |
| Prevalence | Malawi | 1992 | 7.10 | 10.51 | 4.50 | 0.21 | 5373904 | 3376967464 | 76654545 | 2686952 | 73967593.3 | 0.02 |
| Prevalence | Burundi | 1992 | 7.10 | 10.45 | 4.62 | 0.21 | 2868701 | 3376967464 | 79523246 | 1434350 | 78088895.8 | 0.02 |
| Prevalence | South Sudan | 1992 | 8.00 | 11.84 | 5.17 | 0.21 | 3170246 | 3376967464 | 82693492 | 1585123 | 81108369 | 0.02 |
| Prevalence | Nepal | 1992 | 9.66 | 14.05 | 6.19 | 0.21 | 10843820 | 3376967464 | 93537312 | 5421910 | 88115402.1 | 0.03 |
| Prevalence | Sierra Leone | 1992 | 7.29 | 10.18 | 4.94 | 0.21 | 2160724 | 3376967464 | 95698036 | 1080362 | 94617674.1 | 0.03 |
| Prevalence | Guinea-Bissau | 1992 | 6.99 | 9.76 | 4.79 | 0.22 | 518730.4 | 3376967464 | 96216766 | 259365.2 | 95957401.1 | 0.03 |
| Prevalence | Central African Republic | 1992 | 6.35 | 9.44 | 4.07 | 0.22 | 1511635 | 3376967464 | 97728401 | 755817.3 | 96972583.7 | 0.03 |
| Prevalence | Benin | 1992 | 7.90 | 10.90 | 5.49 | 0.23 | 2437709 | 3376967464 | 100166110 | 1218855 | 98947255.7 | 0.03 |

| | | | | | | | | | | | | |
|---|---|---|---|---|---|---|---|---|---|---|---|---|
| Prevalence | Bhutan | 1992 | 11.05 | 16.29 | 7.15 | 0.23 | 362111 | 3376967464 | 100528221 | 181055.5 | 100347165.9 | 0.03 |
| Prevalence | Eritrea | 1992 | 6.40 | 9.38 | 4.13 | 0.23 | 1636011 | 3376967464 | 102164232 | 818005.3 | 101346226.7 | 0.03 |
| Prevalence | Yemen | 1992 | 17.43 | 24.37 | 12.02 | 0.23 | 6649314 | 3376967464 | 108813546 | 3324657 | 105488888.8 | 0.03 |
| Prevalence | Liberia | 1992 | 8.31 | 11.54 | 5.70 | 0.24 | 970267.7 | 3376967464 | 109783813 | 485133.8 | 109298679.5 | 0.03 |
| Prevalence | Chad | 2021 | 7.76 | 10.82 | 5.46 | 0.24 | 8325851 | 5105255047 | 30918137 | 4162925 | 26755211.9 | 0.01 |
| Prevalence | Bangladesh | 1992 | 10.17 | 14.89 | 6.54 | 0.25 | 59911290 | 3376967464 | 169695106 | 29955650 | 139739459.7 | 0.04 |
| Prevalence | Senegal | 1992 | 7.54 | 10.58 | 5.09 | 0.25 | 3982520 | 3376967464 | 173677626 | 1991260 | 171686366 | 0.05 |
| Prevalence | Gambia | 1992 | 8.20 | 11.40 | 5.58 | 0.25 | 540094.8 | 3376967464 | 174217721 | 270047.4 | 173947673.4 | 0.05 |
| Prevalence | United Republic of Tanzania | 1992 | 8.71 | 12.65 | 5.80 | 0.26 | 13815680 | 3376967464 | 188033398 | 6907839 | 181125559.4 | 0.05 |
| Prevalence | Mali | 2021 | 7.41 | 10.38 | 4.93 | 0.27 | 11895330 | 5105255047 | 42813466 | 5947664 | 36865801.5 | 0.01 |
| Prevalence | Lao People's Democratic Republic | 1992 | 5.23 | 7.95 | 3.24 | 0.27 | 2290031 | 3376967464 | 190323429 | 1145016 | 189178413.7 | 0.06 |
| Prevalence | Rwanda | 1992 | 7.88 | 11.46 | 5.23 | 0.28 | 3779329 | 3376967464 | 194102758 | 1889664 | 192213093.7 | 0.06 |
| Prevalence | South Sudan | 2021 | 7.72 | 11.40 | 4.98 | 0.28 | 5135653 | 5105255047 | 47949119 | 2567827 | 45381292.2 | 0.01 |
| Prevalence | Togo | 1992 | 7.64 | 10.68 | 5.29 | 0.28 | 1939975 | 3376967464 | 196042733 | 969987.5 | 195072745.7 | 0.06 |
| Prevalence | Angola | 1992 | 7.14 | 10.44 | 4.58 | 0.28 | 5702682 | 3376967464 | 201745415 | 2851341 | 198894074.2 | 0.06 |
| Prevalence | Timor-Leste | 1992 | 5.63 | 8.56 | 3.53 | 0.28 | 447494.9 | 3376967464 | 202192910 | 223747.4 | 201969162.6 | 0.06 |
| Prevalence | Madagascar | 1992 | 8.82 | 12.62 | 5.95 | 0.28 | 6432226 | 3376967464 | 208625136 | 3216113 | 205409023.2 | 0.06 |
| Prevalence | Equatorial Guinea | 1992 | 7.42 | 10.79 | 4.87 | 0.28 | 229117.5 | 3376967464 | 208854254 | 114558.8 | 208739695 | 0.06 |
| Prevalence | Burkina Faso | 2021 | 8.61 | 11.93 | 6.00 | 0.29 | 11698660 | 5105255047 | 59647781 | 5849331 | 53798449.8 | 0.01 |
| Prevalence | Cabo Verde | 1992 | 9.11 | 12.60 | 6.35 | 0.29 | 183931.3 | 3376967464 | 209038185 | 91965.67 | 208946219.4 | 0.06 |
| Prevalence | Comoros | 1992 | 8.30 | 12.11 | 5.43 | 0.29 | 249116.1 | 3376967464 | 209287301 | 124558 | 209162743.1 | 0.06 |
| Prevalence | Burundi | 2021 | 6.69 | 9.80 | 4.31 | 0.29 | 7040681 | 5105255047 | 66688462 | 3520341 | 63168121.4 | 0.01 |
| Prevalence | Democratic Republic of the | 1992 | 7.64 | 11.10 | 5.00 | 0.29 | 20457730 | 3376967464 | 229745032 | 10228870 | 219516166.6 | 0.07 |

| | | | | | | | | | | | | |
|---|---|---|---|---|---|---|---|---|---|---|---|---|
| | Congo | | | | | | | | | | | |
| Prevalence | Coted'Ivoire | 1992 | 8.70 | 11.89 | 6.00 | 0.29 | 6800901 | 3376967464 | 236545933 | 3400450 | 233145482.4 | 0.07 |
| Prevalence | Cambodia | 1992 | 5.19 | 7.97 | 3.17 | 0.30 | 5566488 | 3376967464 | 242112421 | 2783244 | 239329176.7 | 0.07 |
| Prevalence | Sudan | 1992 | 18.62 | 26.15 | 12.90 | 0.30 | 11202790 | 3376967464 | 253315211 | 5601395 | 247713815.6 | 0.07 |
| Prevalence | Zambia | 1992 | 7.36 | 10.91 | 4.74 | 0.31 | 4197849 | 3376967464 | 257513060 | 2098925 | 255414135.1 | 0.08 |
| Prevalence | Central African Republic | 2021 | 6.34 | 9.23 | 4.11 | 0.31 | 3075108 | 5105255047 | 69763570 | 1537554 | 68226015.8 | 0.01 |
| Prevalence | Nigeria | 1992 | 8.44 | 11.79 | 5.73 | 0.31 | 50478270 | 3376967464 | 307991334 | 25239140 | 282752197 | 0.08 |
| Prevalence | Solomon Islands | 1992 | 8.47 | 12.40 | 5.45 | 0.31 | 187733.3 | 3376967464 | 308179068 | 93866.66 | 308085200.9 | 0.09 |
| Prevalence | Sao Tome and Principe | 1992 | 8.46 | 11.87 | 5.84 | 0.31 | 61668.72 | 3376967464 | 308240736 | 30834.36 | 308209902 | 0.09 |
| Prevalence | Cameroon | 1992 | 9.23 | 12.61 | 6.44 | 0.32 | 5631620 | 3376967464 | 313872356 | 2815810 | 311056546.2 | 0.09 |
| Prevalence | Guatemala | 1992 | 33.67 | 46.70 | 23.30 | 0.32 | 4330417 | 3376967464 | 318202773 | 2165209 | 316037564.7 | 0.09 |
| Prevalence | Papua New Guinea | 1992 | 7.75 | 11.53 | 4.84 | 0.32 | 2419850 | 3376967464 | 320622623 | 1209925 | 319412698.1 | 0.09 |
| Prevalence | Haiti | 1992 | 30.66 | 41.88 | 21.70 | 0.32 | 3615033 | 3376967464 | 324237656 | 1807516 | 322430139.3 | 0.10 |
| Prevalence | Pakistan | 1992 | 11.07 | 15.99 | 7.26 | 0.32 | 60357000 | 3376967464 | 384594651 | 30178500 | 354416153.3 | 0.10 |
| Prevalence | Myanmar | 1992 | 5.61 | 8.42 | 3.48 | 0.33 | 24926270 | 3376967464 | 409520920 | 12463130 | 397057785.4 | 0.12 |
| Prevalence | Mozambique | 2021 | 8.77 | 12.77 | 5.85 | 0.33 | 16039550 | 5105255047 | 85803118 | 8019774 | 77783343.8 | 0.02 |
| Prevalence | Guinea | 2021 | 8.85 | 12.18 | 6.28 | 0.34 | 6958615 | 5105255047 | 92761733 | 3479307 | 89282425.3 | 0.02 |
| Prevalence | Afghanistan | 2021 | 16.48 | 22.79 | 11.60 | 0.34 | 16398400 | 5105255047 | 109160135 | 8199201 | 100960934 | 0.02 |
| Prevalence | Djibouti | 1992 | 8.17 | 11.89 | 5.36 | 0.34 | 281064.1 | 3376967464 | 409801984 | 140532.1 | 409661452 | 0.12 |
| Prevalence | Honduras | 1992 | 36.74 | 50.52 | 25.90 | 0.34 | 2511178 | 3376967464 | 412313162 | 1255589 | 411057573.1 | 0.12 |
| Prevalence | India | 1992 | 9.82 | 14.26 | 6.42 | 0.34 | 515839400 | 3376967464 | 928152513 | 257919700 | 670232837.4 | 0.20 |
| Prevalence | Kenya | 1992 | 7.88 | 11.58 | 5.15 | 0.35 | 12235470 | 3376967464 | 940387986 | 6117737 | 934270249.4 | 0.28 |
| Prevalence | Mauritania | 1992 | 9.72 | 13.29 | 6.82 | 0.35 | 1105278 | 3376967464 | 941493264 | 552639.1 | 940940625.2 | 0.28 |
| Prevalence | Lesotho | 1992 | 8.56 | 12.55 | 5.52 | 0.35 | 821010.8 | 3376967464 | 942314275 | 410505.4 | 941903769.7 | 0.28 |

| | | | | | | | | | | | | |
|---|---|---|---|---|---|---|---|---|---|---|---|---|
| Prevalence | Liberia | 2021 | 9.85 | 13.42 | 6.92 | 0.35 | 3135229 | 5105255047 | 112295364 | 1567614 | 110727749.6 | 0.02 |
| Prevalence | Guinea-Bissau | 2021 | 7.97 | 11.08 | 5.57 | 0.35 | 1119372 | 5105255047 | 113414736 | 559686.2 | 112855050.1 | 0.02 |
| Prevalence | Nicaragua | 1992 | 44.04 | 59.51 | 31.90 | 0.36 | 2103741 | 3376967464 | 944418017 | 1051871 | 943366145.8 | 0.28 |
| Prevalence | Maldives | 1992 | 9.50 | 14.27 | 6.11 | 0.36 | 118284.3 | 3376967464 | 944536301 | 59142.14 | 944477158.7 | 0.28 |
| Prevalence | Sierra Leone | 2021 | 8.33 | 11.52 | 5.79 | 0.36 | 5014942 | 5105255047 | 118429679 | 2507471 | 115922207.5 | 0.02 |
| Prevalence | Ethiopia | 2021 | 6.58 | 9.74 | 4.20 | 0.36 | 61363610 | 5105255047 | 179793292 | 30681810 | 149111485.3 | 0.03 |
| Prevalence | Vanuatu | 1992 | 9.00 | 13.08 | 5.67 | 0.36 | 84103.98 | 3376967464 | 944620405 | 42051.99 | 944578352.8 | 0.28 |
| Prevalence | Morocco | 1992 | 22.53 | 31.02 | 15.82 | 0.37 | 15160090 | 3376967464 | 959780492 | 7580044 | 952200448.5 | 0.28 |
| Prevalence | Benin | 2021 | 9.15 | 12.50 | 6.44 | 0.37 | 7048559 | 5105255047 | 186841851 | 3524280 | 183317571.5 | 0.04 |
| Prevalence | El Salvador | 1992 | 39.93 | 54.47 | 28.21 | 0.38 | 3000660 | 3376967464 | 962781152 | 1500330 | 961280822.3 | 0.28 |
| Prevalence | Democratic Republic of the Congo | 2021 | 8.55 | 12.38 | 5.69 | 0.38 | 49675100 | 5105255047 | 236516952 | 24837550 | 211679401.7 | 0.04 |
| Prevalence | Malawi | 2021 | 8.41 | 12.42 | 5.52 | 0.38 | 10785330 | 5105255047 | 247302286 | 5392667 | 241909619.1 | 0.05 |
| Prevalence | Ghana | 1992 | 8.21 | 11.52 | 5.66 | 0.38 | 8290370 | 3376967464 | 971071522 | 4145185 | 966926337.2 | 0.29 |
| Prevalence | Madagascar | 2021 | 9.29 | 13.09 | 6.27 | 0.40 | 16136660 | 5105255047 | 263438948 | 8068331 | 255370616.9 | 0.05 |
| Prevalence | Eritrea | 2021 | 6.54 | 9.54 | 4.27 | 0.40 | 3903951 | 5105255047 | 267342899 | 1951975 | 265390923.2 | 0.05 |
| Prevalence | Senegal | 2021 | 8.62 | 11.97 | 6.02 | 0.41 | 8916317 | 5105255047 | 276259216 | 4458159 | 271801057.2 | 0.05 |
| Prevalence | Togo | 2021 | 9.13 | 12.37 | 6.45 | 0.41 | 4808399 | 5105255047 | 281067615 | 2404199 | 278663415.3 | 0.05 |
| Prevalence | Gambia | 2021 | 9.78 | 13.37 | 6.86 | 0.41 | 1328594 | 5105255047 | 282396209 | 664296.9 | 281731911.6 | 0.06 |
| Prevalence | Palestine | 1992 | 22.81 | 32.09 | 15.48 | 0.41 | 1092733 | 3376967464 | 972164255 | 546366.4 | 971617888.3 | 0.29 |
| Prevalence | Zimbabwe | 1992 | 11.32 | 16.13 | 7.59 | 0.41 | 5556213 | 3376967464 | 977720467 | 2778106 | 974942361.1 | 0.29 |
| Prevalence | Kiribati | 1992 | 9.21 | 13.48 | 5.96 | 0.42 | 43348.47 | 3376967464 | 977763816 | 21674.24 | 977742141.7 | 0.29 |
| Prevalence | Eswatini | 1992 | 11.46 | 16.37 | 7.41 | 0.42 | 429553.2 | 3376967464 | 978193369 | 214776.6 | 977978592.6 | 0.29 |
| Prevalence | Papua New Guinea | 2021 | 9.09 | 13.11 | 5.83 | 0.42 | 6230283 | 5105255047 | 288626492 | 3115142 | 285511350.2 | 0.06 |

| | | | | | | | | | | | | |
|---|---|---|---|---|---|---|---|---|---|---|---|---|
| Prevalence | Viet Nam | 1992 | 7.03 | 10.47 | 4.38 | 0.42 | 40268370 | 3376967464 | 1018461738 | 20134180 | 998327553.6 | 0.30 |
| Prevalence | Iraq | 1992 | 22.88 | 32.34 | 15.74 | 0.42 | 10043140 | 3376967464 | 1028504876 | 5021569 | 1023483307 | 0.30 |
| Prevalence | Tuvalu | 1992 | 9.41 | 13.69 | 6.13 | 0.42 | 5474.875 | 3376967464 | 1028510350 | 2737.437 | 1028507613 | 0.30 |
| Prevalence | Uganda | 2021 | 8.52 | 12.31 | 5.66 | 0.42 | 22465620 | 5105255047 | 311092115 | 11232810 | 299859303.5 | 0.06 |
| Prevalence | Coted'Ivoire | 2021 | 10.24 | 13.87 | 7.26 | 0.43 | 15562160 | 5105255047 | 326654276 | 7781080 | 318873195.4 | 0.06 |
| Prevalence | Solomon Islands | 2021 | 11.20 | 16.69 | 7.31 | 0.43 | 400945.3 | 5105255047 | 327055221 | 200472.7 | 326854748.6 | 0.06 |
| Prevalence | Congo | 1992 | 8.52 | 12.62 | 5.52 | 0.43 | 1365818 | 3376967464 | 1029876169 | 682909 | 1029193260 | 0.30 |
| Prevalence | Nepal | 2021 | 13.82 | 19.79 | 9.27 | 0.43 | 19987970 | 5105255047 | 347043194 | 9993986 | 337049207.6 | 0.07 |
| Prevalence | Bolivia (Plurinational State of) | 1992 | 37.35 | 51.57 | 26.40 | 0.43 | 3645533 | 3376967464 | 1033521701 | 1822766 | 1031698935 | 0.31 |
| Prevalence | Rwanda | 2021 | 8.23 | 12.21 | 5.34 | 0.44 | 7848418 | 5105255047 | 354891612 | 3924209 | 350967402.9 | 0.07 |
| Prevalence | Egypt | 1992 | 25.76 | 35.67 | 17.94 | 0.44 | 33112790 | 3376967464 | 1066634488 | 16556390 | 1050078095 | 0.31 |
| Prevalence | Botswana | 1992 | 10.38 | 14.74 | 6.83 | 0.44 | 747651.5 | 3376967464 | 1067382140 | 373825.7 | 1067008314 | 0.32 |
| Prevalence | Marshall Islands | 1992 | 9.67 | 14.12 | 6.18 | 0.44 | 23628.24 | 3376967464 | 1067405768 | 11814.12 | 1067393954 | 0.32 |
| Prevalence | Belize | 1992 | 43.90 | 59.32 | 31.08 | 0.44 | 103326.3 | 3376967464 | 1067509094 | 51663.13 | 1067457431 | 0.32 |
| Prevalence | Timor-Leste | 2021 | 7.38 | 10.96 | 4.74 | 0.44 | 802737.4 | 5105255047 | 355694349 | 401368.7 | 355292980.6 | 0.07 |
| Prevalence | Syrian Arab Republic | 1992 | 42.42 | 57.23 | 31.10 | 0.45 | 6931728 | 3376967464 | 1074440822 | 3465864 | 1070974958 | 0.32 |
| Prevalence | United Republic of Tanzania | 2021 | 9.83 | 14.04 | 6.55 | 0.45 | 32205890 | 5105255047 | 387900240 | 16102950 | 371797294.8 | 0.07 |
| Prevalence | Haiti | 2021 | 35.76 | 48.21 | 25.52 | 0.45 | 8005224 | 5105255047 | 395905464 | 4002612 | 391902852.4 | 0.08 |
| Prevalence | Dominican Republic | 1992 | 38.05 | 52.13 | 26.92 | 0.45 | 4367623 | 3376967464 | 1078808445 | 2183812 | 1076624634 | 0.32 |
| Prevalence | Yemen | 2021 | 19.32 | 26.21 | 13.77 | 0.45 | 18839690 | 5105255047 | 414745156 | 9419846 | 405325310.2 | 0.08 |
| Prevalence | Angola | 2021 | 8.81 | 12.71 | 5.81 | 0.45 | 16719890 | 5105255047 | 431465050 | 8359947 | 423105102.9 | 0.08 |
| Prevalence | Grenada | 1992 | 46.23 | 61.95 | 33.30 | 0.46 | 48427.92 | 3376967464 | 1078856873 | 24213.96 | 1078832659 | 0.32 |
| Prevalence | Oman | 1992 | 22.02 | 30.78 | 14.97 | 0.46 | 1153630 | 3376967464 | 1080010503 | 576815.1 | 1079433688 | 0.32 |
| Prevalence | Namibia | 1992 | 10.50 | 14.97 | 6.99 | 0.46 | 804903.9 | 3376967464 | 1080815407 | 402451.9 | 1080412955 | 0.32 |

| | | | | | | | | | | | | |
|---|---|---|---|---|---|---|---|---|---|---|---|
| Prevalence | Guyana | 1992 | 60.27 | 79.04 | 44.71 | 0.47 | 459165.3 | 3376967464 | 1081274573 | 229582.7 | 1081044990 | 0.32 |
| Prevalence | Gabon | 1992 | 9.90 | 14.28 | 6.59 | 0.47 | 557425.5 | 3376967464 | 1081831998 | 278712.8 | 1081553285 | 0.32 |
| Prevalence | Bhutan | 2021 | 15.86 | 22.79 | 10.55 | 0.47 | 519639.6 | 5105255047 | 431984689 | 259819.8 | 431724869.5 | 0.08 |
| Prevalence | Vanuatu | 2021 | 11.98 | 17.44 | 7.92 | 0.47 | 184213.1 | 5105255047 | 432168902 | 92106.55 | 432076795.9 | 0.08 |
| Prevalence | Cambodia | 2021 | 7.68 | 11.27 | 4.80 | 0.47 | 10996940 | 5105255047 | 443165844 | 5498471 | 437667373 | 0.09 |
| Prevalence | Micronesia (Federated States of) | 1992 | 10.68 | 15.89 | 6.93 | 0.47 | 55679.86 | 3376967464 | 1081887678 | 27839.93 | 1081859838 | 0.32 |
| Prevalence | Zimbabwe | 2021 | 11.36 | 16.01 | 7.79 | 0.47 | 8807361 | 5105255047 | 451973205 | 4403681 | 447569524.4 | 0.09 |
| Prevalence | Tajikistan | 1992 | 38.15 | 52.68 | 27.04 | 0.48 | 2943035 | 3376967464 | 1084830713 | 1471517 | 1083359195 | 0.32 |
| Prevalence | Indonesia | 1992 | 6.29 | 9.41 | 3.94 | 0.48 | 115716600 | 3376967464 | 1200547263 | 57858280 | 1142688988 | 0.34 |
| Prevalence | China | 1992 | 9.13 | 13.43 | 5.86 | 0.48 | 815239200 | 3376967464 | 2015786424 | 407619600 | 1608166844 | 0.48 |
| Prevalence | Algeria | 1992 | 26.59 | 36.79 | 18.89 | 0.48 | 14528470 | 3376967464 | 2030314898 | 7264237 | 2023050661 | 0.60 |
| Prevalence | Comoros | 2021 | 8.42 | 12.44 | 5.42 | 0.48 | 467233.1 | 5105255047 | 452440438 | 233616.6 | 452206821.7 | 0.09 |
| Prevalence | Turkey | 1992 | 30.06 | 41.44 | 20.92 | 0.48 | 36334360 | 3376967464 | 2066649254 | 18167180 | 2048482076 | 0.61 |
| Prevalence | Mongolia | 1992 | 41.94 | 58.12 | 29.55 | 0.48 | 1235917 | 3376967464 | 2067885171 | 617958.5 | 2067267212 | 0.61 |
| Prevalence | Paraguay | 1992 | 56.77 | 76.41 | 40.89 | 0.48 | 2324234 | 3376967464 | 2070209405 | 1162117 | 2069047288 | 0.61 |
| Prevalence | Cameroon | 2021 | 11.44 | 15.82 | 8.10 | 0.48 | 17450670 | 5105255047 | 469891104 | 8725333 | 461165771 | 0.09 |
| Prevalence | Iran (Islamic Republic of) | 1992 | 27.47 | 37.65 | 19.61 | 0.48 | 32234900 | 3376967464 | 2102444303 | 16117450 | 2086326854 | 0.62 |
| Prevalence | Colombia | 1992 | 66.14 | 86.57 | 49.03 | 0.48 | 20427380 | 3376967464 | 2122871685 | 10213690 | 2112657994 | 0.63 |
| Prevalence | Saint Vincent and the Grenadines | 1992 | 45.92 | 62.14 | 33.09 | 0.49 | 63082.62 | 3376967464 | 2122934768 | 31541.31 | 2122903226 | 0.63 |
| Prevalence | Djibouti | 2021 | 9.16 | 13.20 | 6.07 | 0.49 | 805751.6 | 5105255047 | 470696855 | 402875.8 | 470293979.5 | 0.09 |
| Prevalence | Tunisia | 1992 | 29.19 | 40.27 | 20.54 | 0.49 | 5133459 | 3376967464 | 2128068226 | 2566729 | 2125501497 | 0.63 |
| Prevalence | Lao People's Democratic Republic | 2021 | 7.68 | 11.66 | 4.88 | 0.49 | 4754006 | 5105255047 | 475450861 | 2377003 | 473073858.3 | 0.09 |
| Prevalence | Bangladesh | 2021 | 13.40 | 19.53 | 8.95 | 0.49 | 107272700 | 5105255047 | 582723583 | 53636360 | 529087222.1 | 0.10 |

| | | | | | | | | | | | | | |
|---|---|---|---|---|---|---|---|---|---|---|---|---|---|
| Prevalence | Samoa | 1992 | 12.86 | 18.88 | 8.45 | 0.49 | 92361.26 | 3376967464 | 2128160588 | 46180.63 | 2128114407 | 0.63 |
| Prevalence | Mauritania | 2021 | 11.21 | 15.33 | 7.80 | 0.50 | 2374531 | 5105255047 | 585098113 | 1187265 | 583910848.2 | 0.11 |
| Prevalence | Democratic People's Republic of Korea | 1992 | 8.10 | 11.90 | 5.14 | 0.50 | 13875620 | 3376967464 | 2142036206 | 6937809 | 2135098397 | 0.63 |
| Prevalence | Uzbekistan | 1992 | 45.65 | 63.00 | 32.13 | 0.50 | 12096580 | 3376967464 | 2154132781 | 6048288 | 2148084494 | 0.64 |
| Prevalence | Nigeria | 2021 | 8.41 | 11.75 | 5.78 | 0.50 | 123377300 | 5105255047 | 708475425 | 61688660 | 646786769.3 | 0.13 |
| Prevalence | Tonga | 1992 | 12.80 | 18.57 | 8.42 | 0.50 | 53374.01 | 3376967464 | 2154186155 | 26687 | 2154159468 | 0.64 |
| Prevalence | Pakistan | 2021 | 13.55 | 19.38 | 9.16 | 0.50 | 141565600 | 5105255047 | 850041000 | 70782790 | 779258212.8 | 0.15 |
| Prevalence | Sao Tome and Principe | 2021 | 11.53 | 15.96 | 8.20 | 0.51 | 131024.1 | 5105255047 | 850172025 | 65512.04 | 850106512.5 | 0.17 |
| Prevalence | Zambia | 2021 | 9.61 | 13.75 | 6.37 | 0.51 | 10789070 | 5105255047 | 860961092 | 5394534 | 855566558.4 | 0.17 |
| Prevalence | Brazil | 1992 | 62.96 | 84.49 | 45.42 | 0.51 | 93825900 | 3376967464 | 2248012056 | 46912950 | 2201099106 | 0.65 |
| Prevalence | Lesotho | 2021 | 10.80 | 15.21 | 7.30 | 0.51 | 1159988 | 5105255047 | 862121080 | 579994.1 | 861541086.2 | 0.17 |
| Prevalence | Suriname | 1992 | 41.91 | 56.98 | 29.74 | 0.51 | 240092.4 | 3376967464 | 2248252148 | 120046.2 | 2248132102 | 0.67 |
| Prevalence | Honduras | 2021 | 53.97 | 72.21 | 38.96 | 0.51 | 6325127 | 5105255047 | 868446208 | 3162564 | 865283644 | 0.17 |
| Prevalence | Peru | 1992 | 39.16 | 53.36 | 27.71 | 0.51 | 13017280 | 3376967464 | 2261269426 | 6508639 | 2254760787 | 0.67 |
| Prevalence | Mexico | 1992 | 54.80 | 74.94 | 38.55 | 0.51 | 51527500 | 3376967464 | 2312796928 | 25763750 | 2287033177 | 0.68 |
| Prevalence | Saint Lucia | 1992 | 49.69 | 66.68 | 35.80 | 0.52 | 80360.32 | 3376967464 | 2312877288 | 40180.16 | 2312837108 | 0.68 |
| Prevalence | Philippines | 1992 | 6.02 | 9.18 | 3.71 | 0.52 | 37716100 | 3376967464 | 2350593387 | 18858050 | 2331735338 | 0.69 |
| Prevalence | Ecuador | 1992 | 51.25 | 67.45 | 37.93 | 0.52 | 6008273 | 3376967464 | 2356601661 | 3004137 | 2353597524 | 0.70 |
| Prevalence | Kenya | 2021 | 9.12 | 13.32 | 6.04 | 0.52 | 29742370 | 5105255047 | 898188583 | 14871190 | 883317395.1 | 0.17 |
| Prevalence | Nicaragua | 2021 | 60.60 | 81.93 | 43.44 | 0.52 | 4297839 | 5105255047 | 902486421 | 2148919 | 900337502 | 0.18 |
| Prevalence | Thailand | 1992 | 8.81 | 13.09 | 5.68 | 0.53 | 39057400 | 3376967464 | 2395659056 | 19528700 | 2376130359 | 0.70 |
| Prevalence | Kiribati | 2021 | 12.35 | 17.92 | 8.20 | 0.53 | 74513.75 | 5105255047 | 902560935 | 37256.87 | 902523678.2 | 0.18 |
| Prevalence | Kyrgyzstan | 1992 | 46.39 | 63.99 | 32.56 | 0.53 | 2635720 | 3376967464 | 2398294776 | 1317860 | 2396976916 | 0.71 |

| | | | | | | | | | | | | |
|---|---|---|---|---|---|---|---|---|---|---|---|---|
| Prevalence | Venezuela (Bolivarian Republic of) | 1992 | 59.65 | 79.52 | 43.78 | 0.53 | 11607490 | 3376967464 | 2409902266 | 5803745 | 2404098521 | 0.71 |
| Prevalence | Tokelau | 1992 | 10.22 | 15.01 | 6.69 | 0.53 | 834.3087 | 3376967464 | 2409903100 | 417.1543 | 2409902683 | 0.71 |
| Prevalence | Sri Lanka | 1992 | 13.67 | 19.21 | 9.14 | 0.53 | 11121210 | 3376967464 | 2421024309 | 5560605 | 2415463705 | 0.72 |
| Prevalence | Cabo Verde | 2021 | 13.65 | 18.65 | 9.77 | 0.53 | 382357.4 | 5105255047 | 902943292 | 191178.7 | 902752113.8 | 0.18 |
| Prevalence | Myanmar | 2021 | 8.24 | 12.20 | 5.21 | 0.53 | 36996570 | 5105255047 | 939939864 | 18498290 | 921441578.5 | 0.18 |
| Prevalence | Nauru | 1992 | 11.97 | 17.69 | 7.70 | 0.54 | 5770.117 | 3376967464 | 2421030079 | 2885.058 | 2421027194 | 0.72 |
| Prevalence | Bosnia and Herzegovina | 1992 | 91.84 | 122.42 | 65.02 | 0.54 | 3112775 | 3376967464 | 2424142855 | 1556388 | 2422586467 | 0.72 |
| Prevalence | Guatemala | 2021 | 47.36 | 65.24 | 33.62 | 0.54 | 9913414 | 5105255047 | 949853279 | 4956707 | 944896571.5 | 0.19 |
| Prevalence | Tajikistan | 2021 | 49.28 | 66.91 | 35.23 | 0.54 | 6207893 | 5105255047 | 956061172 | 3103947 | 952957225.2 | 0.19 |
| Prevalence | Lebanon | 1992 | 32.67 | 44.69 | 23.41 | 0.54 | 1853583 | 3376967464 | 2425996438 | 926791.5 | 2425069646 | 0.72 |
| Prevalence | Sudan | 2021 | 24.58 | 33.83 | 17.28 | 0.54 | 25440510 | 5105255047 | 981501682 | 12720260 | 968781427 | 0.19 |
| Prevalence | Fiji | 1992 | 9.71 | 14.25 | 6.18 | 0.54 | 465040.9 | 3376967464 | 2426461479 | 232520.5 | 2426228958 | 0.72 |
| Prevalence | Costa Rica | 1992 | 73.75 | 98.24 | 55.09 | 0.54 | 1893843 | 3376967464 | 2428355322 | 946921.6 | 2427408400 | 0.72 |
| Prevalence | Jamaica | 1992 | 43.74 | 59.65 | 31.06 | 0.55 | 1395283 | 3376967464 | 2429750605 | 697641.5 | 2429052964 | 0.72 |
| Prevalence | Jordan | 1992 | 28.93 | 40.26 | 20.34 | 0.55 | 2205439 | 3376967464 | 2431956044 | 1102720 | 2430853325 | 0.72 |
| Prevalence | Armenia | 1992 | 52.01 | 72.04 | 36.45 | 0.55 | 2180135 | 3376967464 | 2434136179 | 1090067 | 2433046112 | 0.72 |
| Prevalence | South Africa | 1992 | 14.22 | 19.95 | 9.86 | 0.55 | 22998760 | 3376967464 | 2457134939 | 11499380 | 2445635559 | 0.72 |
| Prevalence | Albania | 1992 | 80.62 | 107.47 | 58.11 | 0.55 | 2048832 | 3376967464 | 2459183771 | 1024416 | 2458159355 | 0.73 |
| Prevalence | Mauritius | 1992 | 12.43 | 18.07 | 8.15 | 0.55 | 729511 | 3376967464 | 2459913282 | 364755.5 | 2459548527 | 0.73 |
| Prevalence | Libya | 1992 | 29.01 | 40.54 | 20.17 | 0.55 | 2388892 | 3376967464 | 2462302174 | 1194446 | 2461107728 | 0.73 |
| Prevalence | Panama | 1992 | 56.96 | 76.30 | 41.33 | 0.55 | 1507409 | 3376967464 | 2463809583 | 753704.7 | 2463055878 | 0.73 |
| Prevalence | Malaysia | 1992 | 9.62 | 14.30 | 6.19 | 0.56 | 11151630 | 3376967464 | 2474961212 | 5575814 | 2469385397 | 0.73 |
| Prevalence | Saudi Arabia | 1992 | 24.04 | 33.61 | 16.32 | 0.56 | 9752944 | 3376967464 | 2484714156 | 4876472 | 2479837684 | 0.73 |

| | | | | | | | | | | | | |
|---|---|---|---|---|---|---|---|---|---|---|---|---|
| Prevalence | Morocco | 2021 | 30.14 | 41.59 | 21.36 | 0.56 | 24647580 | 5105255047 | 1006149263 | 12323790 | 993825472.4 | 0.19 |
| Prevalence | El Salvador | 2021 | 59.67 | 80.90 | 42.64 | 0.56 | 4074428 | 5105255047 | 1010223691 | 2037214 | 1008186477 | 0.20 |
| Prevalence | Ghana | 2021 | 10.41 | 14.28 | 7.26 | 0.56 | 20161140 | 5105255047 | 1030384835 | 10080570 | 1020304263 | 0.20 |
| Prevalence | Turkmenistan | 1992 | 44.98 | 61.79 | 31.58 | 0.57 | 2146841 | 3376967464 | 2486860997 | 1073420 | 2485787576 | 0.74 |
| Prevalence | Cuba | 1992 | 60.27 | 80.27 | 44.76 | 0.57 | 7554381 | 3376967464 | 2494415377 | 3777190 | 2490638187 | 0.74 |
| Prevalence | Dominica | 1992 | 46.40 | 62.64 | 33.34 | 0.57 | 41551.19 | 3376967464 | 2494456928 | 20775.6 | 2494436153 | 0.74 |
| Prevalence | Democratic People's Republic of Korea | 2021 | 10.75 | 15.81 | 6.94 | 0.57 | 18946650 | 5105255047 | 1049331482 | 9473323 | 1039858159 | 0.20 |
| Prevalence | Marshall Islands | 2021 | 13.25 | 19.20 | 8.90 | 0.57 | 36532.79 | 5105255047 | 1049368015 | 18266.39 | 1049349748 | 0.21 |
| Prevalence | India | 2021 | 12.87 | 18.56 | 8.70 | 0.58 | 950604900 | 5105255047 | 1999972931 | 475302500 | 1524670473 | 0.30 |
| Prevalence | Tuvalu | 2021 | 14.14 | 20.06 | 9.43 | 0.58 | 7770.842 | 5105255047 | 1999980701 | 3885.421 | 1999976816 | 0.39 |
| Prevalence | Cook Islands | 1992 | 13.62 | 19.98 | 8.95 | 0.58 | 11553.59 | 3376967464 | 2494468482 | 5776.796 | 2494462705 | 0.74 |
| Prevalence | Congo | 2021 | 10.14 | 14.82 | 6.69 | 0.58 | 3289800 | 5105255047 | 2003270502 | 1644900 | 2001625602 | 0.39 |
| Prevalence | Eswatini | 2021 | 13.33 | 18.64 | 8.86 | 0.59 | 702572.3 | 5105255047 | 2003973074 | 351286.1 | 2003621788 | 0.39 |
| Prevalence | Micronesia (Federated States of) | 2021 | 15.58 | 22.23 | 10.56 | 0.59 | 67172.81 | 5105255047 | 2004040247 | 33586.4 | 2004006661 | 0.39 |
| Prevalence | Uruguay | 1992 | 73.92 | 95.96 | 55.28 | 0.59 | 1978724 | 3376967464 | 2496447206 | 989362.1 | 2495457844 | 0.74 |
| Prevalence | Seychelles | 1992 | 12.02 | 17.62 | 7.87 | 0.59 | 45508.48 | 3376967464 | 2496492715 | 22754.24 | 2496469961 | 0.74 |
| Prevalence | Samoa | 2021 | 16.62 | 23.90 | 11.10 | 0.59 | 122652 | 5105255047 | 2004162899 | 61325.99 | 2004101573 | 0.39 |
| Prevalence | Azerbaijan | 1992 | 48.74 | 67.36 | 34.61 | 0.59 | 4664389 | 3376967464 | 2501157104 | 2332195 | 2498824909 | 0.74 |
| Prevalence | Bahrain | 1992 | 32.21 | 44.76 | 22.40 | 0.60 | 353670.8 | 3376967464 | 2501510775 | 176835.4 | 2501333939 | 0.74 |
| Prevalence | Kazakhstan | 1992 | 51.04 | 70.32 | 35.56 | 0.60 | 10145610 | 3376967464 | 2511656388 | 5072806 | 2506583581 | 0.74 |
| Prevalence | Venezuela (Bolivarian Republic of) | 2021 | 80.01 | 106.76 | 59.17 | 0.60 | 17426450 | 5105255047 | 2021589354 | 8713227 | 2012876126 | 0.39 |
| Prevalence | Argentina | 1992 | 72.57 | 94.68 | 54.11 | 0.60 | 20617890 | 3376967464 | 2532274273 | 10308940 | 2521965330 | 0.75 |

| | | | | | | | | | | | | |
|---|---|---|---|---|---|---|---|---|---|---|---|---|
| Prevalence | Bolivia (Plurinational State of) | 2021 | 64.24 | 88.33 | 45.77 | 0.60 | 7560756 | 5105255047 | 2029150109 | 3780378 | 2025369731 | 0.40 |
| Prevalence | Chile | 1992 | 70.96 | 93.71 | 53.11 | 0.60 | 8721318 | 3376967464 | 2540995591 | 4360659 | 2536634932 | 0.75 |
| Prevalence | Saint Kitts and Nevis | 1992 | 47.11 | 64.21 | 33.99 | 0.60 | 23875.01 | 3376967464 | 2541019466 | 11937.5 | 2541007529 | 0.75 |
| Prevalence | Niue | 1992 | 12.53 | 18.29 | 8.21 | 0.60 | 1295.984 | 3376967464 | 2541020762 | 647.9919 | 2541020114 | 0.75 |
| Prevalence | Kyrgyzstan | 2021 | 65.48 | 89.06 | 46.57 | 0.60 | 4248862 | 5105255047 | 2033398972 | 2124431 | 2031274540 | 0.40 |
| Prevalence | Egypt | 2021 | 36.45 | 50.82 | 25.89 | 0.61 | 64397140 | 5105255047 | 2097796116 | 32198570 | 2065597544 | 0.40 |
| Prevalence | Republic of Moldova | 1992 | 71.72 | 98.25 | 51.28 | 0.61 | 2854706 | 3376967464 | 2543875468 | 1427353 | 2542448115 | 0.75 |
| Prevalence | Belize | 2021 | 59.93 | 81.43 | 43.38 | 0.61 | 283479.5 | 5105255047 | 2098079595 | 141739.7 | 2097937856 | 0.41 |
| Prevalence | North Macedonia | 1992 | 86.58 | 115.74 | 62.31 | 0.61 | 1323446 | 3376967464 | 2545198915 | 661723.2 | 2544537192 | 0.75 |
| Prevalence | Portugal | 1992 | 40.07 | 53.15 | 29.59 | 0.61 | 6780922 | 3376967464 | 2551979837 | 3390461 | 2548589376 | 0.75 |
| Prevalence | Namibia | 2021 | 12.51 | 17.83 | 8.35 | 0.62 | 1504481 | 5105255047 | 2099584076 | 752240.5 | 2098831836 | 0.41 |
| Prevalence | Mongolia | 2021 | 65.62 | 88.31 | 46.23 | 0.62 | 2105152 | 5105255047 | 2101689228 | 1052576 | 2100636652 | 0.41 |
| Prevalence | American Samoa | 1992 | 17.40 | 25.53 | 11.52 | 0.62 | 29749.7 | 3376967464 | 2552009587 | 14874.85 | 2551994712 | 0.76 |
| Prevalence | Dominican Republic | 2021 | 57.69 | 77.17 | 42.12 | 0.62 | 7231298 | 5105255047 | 2108920526 | 3615649 | 2105304877 | 0.41 |
| Prevalence | Syrian Arab Republic | 2021 | 45.66 | 60.97 | 33.29 | 0.62 | 9352995 | 5105255047 | 2118273521 | 4676498 | 2113597023 | 0.41 |
| Prevalence | Antigua and Barbuda | 1992 | 48.92 | 66.59 | 35.59 | 0.62 | 38493.21 | 3376967464 | 2552048080 | 19246.61 | 2552028833 | 0.76 |
| Prevalence | Nauru | 2021 | 15.39 | 22.16 | 10.37 | 0.63 | 6647.105 | 5105255047 | 2118280168 | 3323.553 | 2118276845 | 0.41 |
| Prevalence | Tonga | 2021 | 17.42 | 25.46 | 11.58 | 0.63 | 60598.31 | 5105255047 | 2118340767 | 30299.16 | 2118310467 | 0.41 |
| Prevalence | Viet Nam | 2021 | 10.77 | 15.83 | 6.92 | 0.63 | 67836850 | 5105255047 | 2186177612 | 33918420 | 2152259189 | 0.42 |
| Prevalence | Romania | 1992 | 213.76 | 285.31 | 153.07 | 0.63 | 15484770 | 3376967464 | 2567532852 | 7742386 | 2559790466 | 0.76 |
| Prevalence | Palestine | 2021 | 24.78 | 33.97 | 17.26 | 0.63 | 3092703 | 5105255047 | 2189270315 | 1546351 | 2187723963 | 0.43 |
| Prevalence | Belarus | 1992 | 77.52 | 105.85 | 55.53 | 0.63 | 6938289 | 3376967464 | 2574471141 | 3469144 | 2571001997 | 0.76 |
| Prevalence | Suriname | 2021 | 57.18 | 77.44 | 41.91 | 0.63 | 384154.9 | 5105255047 | 2189654470 | 192077.4 | 2189462392 | 0.43 |
| Prevalence | Trinidad and Tobago | 1992 | 46.70 | 64.35 | 33.25 | 0.63 | 745716.6 | 3376967464 | 2575216858 | 372858.3 | 2574843999 | 0.76 |

| | | | | | | | | | | | | |
|---|---|---|---|---|---|---|---|---|---|---|---|---|
| Prevalence | Gabon | 2021 | 13.27 | 18.93 | 8.86 | 0.63 | 1102224 | 5105255047 | 2190756693 | 551111.8 | 2190205581 | 0.43 |
| Prevalence | Paraguay | 2021 | 89.56 | 121.26 | 65.96 | 0.64 | 4680073 | 5105255047 | 2195436766 | 2340037 | 2193096730 | 0.43 |
| Prevalence | Saint Vincent and the Grenadines | 2021 | 66.52 | 89.56 | 48.22 | 0.64 | 76552.59 | 5105255047 | 2195513319 | 38276.3 | 2195475043 | 0.43 |
| Prevalence | Poland | 1992 | 46.36 | 63.73 | 32.66 | 0.64 | 24967360 | 3376967464 | 2600184220 | 12483680 | 2587700539 | 0.77 |
| Prevalence | Serbia | 1992 | 88.69 | 118.66 | 63.49 | 0.64 | 6574516 | 3376967464 | 2606758736 | 3287258 | 2603471478 | 0.77 |
| Prevalence | Botswana | 2021 | 13.63 | 19.33 | 9.07 | 0.64 | 1590402 | 5105255047 | 2197103721 | 795200.9 | 2196308520 | 0.43 |
| Prevalence | Guyana | 2021 | 76.55 | 98.62 | 57.24 | 0.65 | 501391.8 | 5105255047 | 2197605113 | 250695.9 | 2197354417 | 0.43 |
| Prevalence | Maldives | 2021 | 16.64 | 24.27 | 11.13 | 0.65 | 394937.9 | 5105255047 | 2198000050 | 197468.9 | 2197802582 | 0.43 |
| Prevalence | Bulgaria | 1992 | 104.49 | 142.38 | 74.52 | 0.65 | 5699135 | 3376967464 | 2612457871 | 2849568 | 2609608303 | 0.77 |
| Prevalence | Philippines | 2021 | 8.44 | 12.42 | 5.42 | 0.65 | 73079830 | 5105255047 | 2271079884 | 36539920 | 2234539967 | 0.44 |
| Prevalence | Spain | 1992 | 111.31 | 145.54 | 82.49 | 0.65 | 26113210 | 3376967464 | 2638571077 | 13056600 | 2625514474 | 0.78 |
| Prevalence | Brazil | 2021 | 71.08 | 95.81 | 50.62 | 0.65 | 150405800 | 5105255047 | 2421485716 | 75202920 | 2346282800 | 0.46 |
| Prevalence | Colombia | 2021 | 100.44 | 129.57 | 73.97 | 0.66 | 33609590 | 5105255047 | 2455095302 | 16804790 | 2438290509 | 0.48 |
| Prevalence | Indonesia | 2021 | 9.47 | 13.80 | 6.11 | 0.66 | 194150400 | 5105255047 | 2649245676 | 97075190 | 2552170489 | 0.50 |
| Prevalence | Ukraine | 1992 | 75.93 | 104.56 | 53.69 | 0.66 | 34804310 | 3376967464 | 2673375387 | 17402160 | 2655973232 | 0.79 |
| Prevalence | Equatorial Guinea | 2021 | 12.47 | 17.67 | 8.27 | 0.66 | 893663 | 5105255047 | 2650139339 | 446831.5 | 2649692507 | 0.52 |
| Prevalence | Algeria | 2021 | 34.22 | 46.73 | 24.35 | 0.66 | 28054400 | 5105255047 | 2678193741 | 14027200 | 2664166540 | 0.52 |
| Prevalence | Hungary | 1992 | 159.60 | 210.29 | 117.74 | 0.66 | 6927328 | 3376967464 | 2680302715 | 3463664 | 2676839051 | 0.79 |
| Prevalence | Qatar | 1992 | 45.47 | 63.11 | 31.87 | 0.66 | 335643.3 | 3376967464 | 2680638358 | 167821.6 | 2680470537 | 0.79 |
| Prevalence | Ecuador | 2021 | 80.16 | 97.35 | 65.05 | 0.66 | 11574520 | 5105255047 | 2689768262 | 5787261 | 2683981001 | 0.53 |
| Prevalence | Peru | 2021 | 67.09 | 89.48 | 48.02 | 0.66 | 23885430 | 5105255047 | 2713653696 | 11942720 | 2701710979 | 0.53 |
| Prevalence | Barbados | 1992 | 62.17 | 84.50 | 45.89 | 0.66 | 162757.9 | 3376967464 | 2680801116 | 81378.93 | 2680719737 | 0.79 |
| Prevalence | Uzbekistan | 2021 | 79.79 | 108.46 | 56.62 | 0.66 | 22329360 | 5105255047 | 2735983057 | 11164680 | 2724818377 | 0.53 |
| Prevalence | Iraq | 2021 | 25.19 | 35.11 | 17.23 | 0.66 | 26081420 | 5105255047 | 2762064476 | 13040710 | 2749023766 | 0.54 |

| | | | | | | | | | | | | |
|---|---|---|---|---|---|---|---|---|---|---|---|---|
| Prevalence | Mexico | 2021 | 79.70 | 108.83 | 56.86 | 0.66 | 86646750 | 5105255047 | 2848711229 | 43323380 | 2805387853 | 0.55 |
| Prevalence | Georgia | 1992 | 58.27 | 80.85 | 40.72 | 0.67 | 3570243 | 3376967464 | 2684371360 | 1785122 | 2682586238 | 0.79 |
| Prevalence | Slovakia | 1992 | 64.71 | 84.59 | 48.66 | 0.67 | 3469779 | 3376967464 | 2687841139 | 1734890 | 2686106249 | 0.80 |
| Prevalence | Cyprus | 1992 | 79.40 | 107.03 | 57.83 | 0.67 | 522214.5 | 3376967464 | 2688363353 | 261107.2 | 2688102246 | 0.80 |
| Prevalence | Malta | 1992 | 65.09 | 86.55 | 47.17 | 0.67 | 249227.5 | 3376967464 | 2688612581 | 124613.8 | 2688487967 | 0.80 |
| Prevalence | Guam | 1992 | 16.72 | 24.23 | 11.14 | 0.67 | 93009.67 | 3376967464 | 2688705591 | 46504.84 | 2688659086 | 0.80 |
| Prevalence | Puerto Rico | 1992 | 70.35 | 97.41 | 50.39 | 0.67 | 2339319 | 3376967464 | 2691044910 | 1169660 | 2689875250 | 0.80 |
| Prevalence | Cuba | 2021 | 87.48 | 116.71 | 64.69 | 0.67 | 7721598 | 5105255047 | 2856432827 | 3860799 | 2852572028 | 0.56 |
| Prevalence | Grenada | 2021 | 76.56 | 108.11 | 56.34 | 0.67 | 71465.1 | 5105255047 | 2856504292 | 35732.55 | 2856468560 | 0.56 |
| Prevalence | Kuwait | 1992 | 38.87 | 53.36 | 27.08 | 0.67 | 1142046 | 3376967464 | 2692186956 | 571023.1 | 2691615933 | 0.80 |
| Prevalence | United States Virgin Islands | 1992 | 72.02 | 95.79 | 50.88 | 0.67 | 68428.68 | 3376967464 | 2692255385 | 34214.34 | 2692221171 | 0.80 |
| Prevalence | Saint Lucia | 2021 | 71.30 | 95.66 | 52.39 | 0.67 | 127254.6 | 5105255047 | 2856631547 | 63627.3 | 2856567920 | 0.56 |
| Prevalence | Montenegro | 1992 | 107.17 | 143.92 | 77.32 | 0.67 | 417608.2 | 3376967464 | 2692672993 | 208804.1 | 2692464189 | 0.80 |
| Prevalence | Palau | 1992 | 13.92 | 20.25 | 9.17 | 0.67 | 10631.73 | 3376967464 | 2692683625 | 5315.866 | 2692678309 | 0.80 |
| Prevalence | Fiji | 2021 | 12.83 | 18.81 | 8.32 | 0.68 | 595530.4 | 5105255047 | 2857227077 | 297765.2 | 2856929312 | 0.56 |
| Prevalence | United Arab Emirates | 1992 | 39.31 | 52.96 | 27.80 | 0.68 | 1447269 | 3376967464 | 2694130893 | 723634.3 | 2693407259 | 0.80 |
| Prevalence | Brunei Darussalam | 1992 | 73.15 | 98.70 | 52.55 | 0.68 | 172733 | 3376967464 | 2694303626 | 86366.52 | 2694217260 | 0.80 |
| Prevalence | Croatia | 1992 | 110.24 | 143.88 | 82.93 | 0.68 | 3329713 | 3376967464 | 2697633339 | 1664857 | 2695968483 | 0.80 |
| Prevalence | South Africa | 2021 | 15.99 | 22.36 | 10.94 | 0.68 | 37983600 | 5105255047 | 2895210682 | 18991800 | 2876218879 | 0.56 |
| Prevalence | Lithuania | 1992 | 75.37 | 98.28 | 56.12 | 0.68 | 2447071 | 3376967464 | 2700080410 | 1223536 | 2698856875 | 0.80 |
| Prevalence | Turkmenistan | 2021 | 76.08 | 103.19 | 53.63 | 0.68 | 3350351 | 5105255047 | 2898561033 | 1675176 | 2896885857 | 0.57 |
| Prevalence | Tunisia | 2021 | 36.98 | 50.23 | 26.63 | 0.68 | 7946659 | 5105255047 | 2906507692 | 3973329 | 2902534362 | 0.57 |
| Prevalence | Thailand | 2021 | 13.13 | 19.77 | 8.46 | 0.68 | 47274310 | 5105255047 | 2953782004 | 23637160 | 2930144848 | 0.57 |
| Prevalence | Jamaica | 2021 | 56.66 | 77.00 | 40.84 | 0.68 | 1946551 | 5105255047 | 2955728554 | 973275.3 | 2954755279 | 0.58 |

| | | | | | | | | | | | | |
|---|---|---|---|---|---|---|---|---|---|---|---|---|
| Prevalence | Taiwan (Province of China) | 1992 | 12.35 | 18.40 | 7.91 | 0.68 | 14026120 | 3376967464 | 2714106526 | 7013058 | 2707093468 | 0.80 |
| Prevalence | Tokelau | 2021 | 15.32 | 22.55 | 10.09 | 0.69 | 843.5528 | 5105255047 | 2955729398 | 421.7764 | 2955728976 | 0.58 |
| Prevalence | Greece | 1992 | 77.83 | 103.72 | 56.57 | 0.69 | 7122789 | 3376967464 | 2721229315 | 3561394 | 2717667921 | 0.80 |
| Prevalence | Bahamas | 1992 | 50.35 | 68.00 | 36.53 | 0.69 | 171499.1 | 3376967464 | 2721400814 | 85749.56 | 2721315065 | 0.81 |
| Prevalence | Russian Federation | 1992 | 75.44 | 103.12 | 52.99 | 0.69 | 101681600 | 3376967464 | 2823082430 | 50840810 | 2772241622 | 0.82 |
| Prevalence | Azerbaijan | 2021 | 80.86 | 109.82 | 57.51 | 0.69 | 7440503 | 5105255047 | 2963169901 | 3720252 | 2959449649 | 0.58 |
| Prevalence | Estonia | 1992 | 94.52 | 127.74 | 67.80 | 0.70 | 1018565 | 3376967464 | 2824100995 | 509282.4 | 2823591712 | 0.84 |
| Prevalence | Latvia | 1992 | 109.27 | 147.30 | 78.08 | 0.70 | 1746906 | 3376967464 | 2825847901 | 873453 | 2824974448 | 0.84 |
| Prevalence | Iran (Islamic Republic of) | 2021 | 34.28 | 46.27 | 24.62 | 0.70 | 59167520 | 5105255047 | 3022337423 | 29583760 | 2992753662 | 0.59 |
| Prevalence | Czechia | 1992 | 130.60 | 176.17 | 93.40 | 0.70 | 6884471 | 3376967464 | 2832732372 | 3442236 | 2829290136 | 0.84 |
| Prevalence | Costa Rica | 2021 | 117.56 | 152.26 | 86.25 | 0.70 | 3249833 | 5105255047 | 3025587256 | 1624917 | 3023962339 | 0.59 |
| Prevalence | Sri Lanka | 2021 | 21.05 | 28.91 | 14.77 | 0.70 | 14714740 | 5105255047 | 3040301997 | 7357370 | 3032944626 | 0.59 |
| Prevalence | Armenia | 2021 | 91.43 | 123.06 | 66.22 | 0.70 | 2004496 | 5105255047 | 3042306493 | 1002248 | 3041304245 | 0.60 |
| Prevalence | Singapore | 1992 | 86.26 | 115.28 | 63.88 | 0.70 | 2369939 | 3376967464 | 2835102310 | 1184969 | 2833917341 | 0.84 |
| Prevalence | Bermuda | 1992 | 129.87 | 174.19 | 95.01 | 0.70 | 42685.4 | 3376967464 | 2835144996 | 21342.7 | 2835123653 | 0.84 |
| Prevalence | Albania | 2021 | 131.64 | 177.35 | 95.90 | 0.71 | 1808384 | 5105255047 | 3044114877 | 904192 | 3043210685 | 0.60 |
| Prevalence | Panama | 2021 | 73.81 | 98.89 | 53.82 | 0.71 | 2749507 | 5105255047 | 3046864384 | 1374753 | 3045489630 | 0.60 |
| Prevalence | Republic of Korea | 1992 | 72.94 | 99.46 | 51.67 | 0.71 | 31435440 | 3376967464 | 2866580440 | 15717720 | 2850862718 | 0.84 |
| Prevalence | Turkey | 2021 | 37.46 | 51.38 | 26.43 | 0.71 | 56921960 | 5105255047 | 3103786340 | 28460980 | 3075325362 | 0.60 |
| Prevalence | Northern Mariana Islands | 1992 | 24.46 | 34.70 | 17.16 | 0.72 | 33889.48 | 3376967464 | 2866614329 | 16944.74 | 2866597384 | 0.85 |
| Prevalence | Italy | 1992 | 119.50 | 162.41 | 85.54 | 0.72 | 38983200 | 3376967464 | 2905597533 | 19491600 | 2886105931 | 0.85 |
| Prevalence | Israel | 1992 | 50.49 | 68.01 | 36.82 | 0.72 | 3155772 | 3376967464 | 2908753305 | 1577886 | 2907175419 | 0.86 |
| Prevalence | Mauritius | 2021 | 17.79 | 25.49 | 11.78 | 0.72 | 900058.5 | 5105255047 | 3104686398 | 450029.2 | 3104236369 | 0.61 |
| Prevalence | Uruguay | 2021 | 109.32 | 144.93 | 81.40 | 0.72 | 2214747 | 5105255047 | 3106901145 | 1107373 | 3105793772 | 0.61 |

| | | | | | | | | | | | | |
|---|---|---|---|---|---|---|---|---|---|---|---|---|
| Prevalence | China | 2021 | 15.95 | 22.84 | 10.73 | 0.72 | 967106700 | 5105255047 | 4074007884 | 483553400 | 3590454514 | 0.70 |
| Prevalence | Bosnia and Herzegovina | 2021 | 158.89 | 213.29 | 115.17 | 0.72 | 2206873 | 5105255047 | 4076214757 | 1103437 | 4075111320 | 0.80 |
| Prevalence | Argentina | 2021 | 94.97 | 124.21 | 69.27 | 0.72 | 30052210 | 5105255047 | 4106266964 | 15026100 | 4091240861 | 0.80 |
| Prevalence | American Samoa | 2021 | 20.92 | 29.95 | 14.25 | 0.72 | 31908.17 | 5105255047 | 4106298872 | 15954.09 | 4106282918 | 0.80 |
| Prevalence | Kazakhstan | 2021 | 84.92 | 115.92 | 59.82 | 0.73 | 12126520 | 5105255047 | 4118425393 | 6063260 | 4112362132 | 0.81 |
| Prevalence | Jordan | 2021 | 34.91 | 48.42 | 24.43 | 0.73 | 8180007 | 5105255047 | 4126605399 | 4090003 | 4122515396 | 0.81 |
| Prevalence | Libya | 2021 | 34.17 | 46.35 | 25.04 | 0.73 | 5029432 | 5105255047 | 4131634831 | 2514716 | 4129120115 | 0.81 |
| Prevalence | Niue | 2021 | 18.12 | 26.42 | 12.02 | 0.73 | 1099.18 | 5105255047 | 4131635930 | 549.5901 | 4131635381 | 0.81 |
| Prevalence | Greenland | 1992 | 77.11 | 103.43 | 55.70 | 0.73 | 38395.59 | 3376967464 | 2908791700 | 19197.79 | 2908772503 | 0.86 |
| Prevalence | Seychelles | 2021 | 17.93 | 25.65 | 12.06 | 0.73 | 72957.22 | 5105255047 | 4131708887 | 36478.61 | 4131672409 | 0.81 |
| Prevalence | Ireland | 1992 | 101.30 | 136.33 | 74.16 | 0.73 | 2251651 | 3376967464 | 2911043351 | 1125825 | 2909917526 | 0.86 |
| Prevalence | Republic of Moldova | 2021 | 111.33 | 151.95 | 79.65 | 0.73 | 2516382 | 5105255047 | 4134225269 | 1258191 | 4132967078 | 0.81 |
| Prevalence | Georgia | 2021 | 106.92 | 144.22 | 77.69 | 0.73 | 2299321 | 5105255047 | 4136524590 | 1149660 | 4135374929 | 0.81 |
| Prevalence | Australia | 1992 | 67.61 | 91.16 | 48.65 | 0.73 | 11484530 | 3376967464 | 2922527885 | 5742267 | 2916785618 | 0.86 |
| Prevalence | Slovenia | 1992 | 167.77 | 228.40 | 122.26 | 0.74 | 1357830 | 3376967464 | 2923885715 | 678914.9 | 2923206800 | 0.87 |
| Prevalence | Malaysia | 2021 | 14.71 | 21.62 | 9.55 | 0.74 | 21856660 | 5105255047 | 4158381246 | 10928330 | 4147452918 | 0.81 |
| Prevalence | France | 1992 | 98.25 | 128.30 | 72.99 | 0.74 | 38242170 | 3376967464 | 2962127885 | 19121090 | 2943006800 | 0.87 |
| Prevalence | Portugal | 2021 | 62.49 | 82.67 | 45.61 | 0.74 | 6825510 | 5105255047 | 4165206755 | 3412755 | 4161794000 | 0.82 |
| Prevalence | Lebanon | 2021 | 42.80 | 57.58 | 31.72 | 0.74 | 3716347 | 5105255047 | 4168923102 | 1858174 | 4167064929 | 0.82 |
| Prevalence | Barbados | 2021 | 80.44 | 110.73 | 57.83 | 0.75 | 202783.5 | 5105255047 | 4169125886 | 101391.7 | 4169024494 | 0.82 |
| Prevalence | Dominica | 2021 | 72.85 | 99.08 | 52.92 | 0.75 | 46130.66 | 5105255047 | 4169172016 | 23065.33 | 4169148951 | 0.82 |
| Prevalence | Belgium | 1992 | 101.69 | 136.10 | 74.04 | 0.75 | 6685776 | 3376967464 | 2968813661 | 3342888 | 2965470773 | 0.88 |
| Prevalence | Antigua and Barbuda | 2021 | 66.20 | 90.05 | 47.66 | 0.75 | 63578.22 | 5105255047 | 4169235595 | 31789.11 | 4169203806 | 0.82 |
| Prevalence | North Macedonia | 2021 | 143.15 | 190.39 | 104.84 | 0.75 | 1541196 | 5105255047 | 4170776791 | 770598.2 | 4170006193 | 0.82 |

| | | | | | | | | | | | | |
|---|---|---|---|---|---|---|---|---|---|---|---|---|
| Prevalence | Bahrain | 2021 | 40.22 | 55.46 | 28.38 | 0.75 | 1178377 | 5105255047 | 4171955168 | 589188.4 | 4171365980 | 0.82 |
| Prevalence | Palau | 2021 | 19.39 | 28.24 | 13.04 | 0.75 | 13185.18 | 5105255047 | 4171968353 | 6592.589 | 4171961761 | 0.82 |
| Prevalence | Austria | 1992 | 207.46 | 250.91 | 170.71 | 0.75 | 5270737 | 3376967464 | 2974084397 | 2635368 | 2971449029 | 0.88 |
| Prevalence | United Kingdom | 1992 | 102.11 | 134.76 | 75.68 | 0.75 | 37397090 | 3376967464 | 3011481485 | 18698540 | 2992782941 | 0.89 |
| Prevalence | Saint Kitts and Nevis | 2021 | 68.46 | 91.43 | 49.63 | 0.75 | 43420.68 | 5105255047 | 4172011774 | 21710.34 | 4171990064 | 0.82 |
| Prevalence | New Zealand | 1992 | 79.58 | 107.00 | 57.93 | 0.76 | 2285123 | 3376967464 | 3013766608 | 1142561 | 3012624047 | 0.89 |
| Prevalence | Ukraine | 2021 | 103.54 | 141.67 | 72.38 | 0.76 | 29287980 | 5105255047 | 4201299752 | 14643990 | 4186655763 | 0.82 |
| Prevalence | Finland | 1992 | 157.81 | 210.24 | 114.62 | 0.76 | 3388185 | 3376967464 | 3017154793 | 1694093 | 3015460701 | 0.89 |
| Prevalence | Andorra | 1992 | 88.69 | 119.64 | 63.93 | 0.77 | 45683.08 | 3376967464 | 3017200476 | 22841.54 | 3017177635 | 0.89 |
| Prevalence | Bulgaria | 2021 | 128.66 | 168.57 | 94.07 | 0.77 | 4337506 | 5105255047 | 4205637258 | 2168753 | 4203468505 | 0.82 |
| Prevalence | Romania | 2021 | 378.61 | 505.17 | 265.40 | 0.77 | 12142540 | 5105255047 | 4217779801 | 6071271 | 4211708529 | 0.82 |
| Prevalence | Trinidad and Tobago | 2021 | 65.29 | 89.64 | 46.44 | 0.77 | 942563.1 | 5105255047 | 4218722364 | 471281.5 | 4218251082 | 0.83 |
| Prevalence | Spain | 2021 | 146.29 | 192.93 | 106.21 | 0.77 | 29873650 | 5105255047 | 4248596015 | 14936830 | 4233659190 | 0.83 |
| Prevalence | United States of America | 1992 | 130.73 | 176.39 | 95.00 | 0.77 | 169626400 | 3376967464 | 3186826882 | 84813200 | 3102013679 | 0.92 |
| Prevalence | Chile | 2021 | 104.35 | 138.88 | 75.46 | 0.77 | 12819950 | 5105255047 | 4261415966 | 6409975 | 4255005991 | 0.83 |
| Prevalence | Northern Mariana Islands | 2021 | 29.06 | 40.26 | 20.57 | 0.77 | 33644 | 5105255047 | 4261449610 | 16822 | 4261432788 | 0.83 |
| Prevalence | Iceland | 1992 | 139.01 | 185.68 | 101.79 | 0.77 | 167194.9 | 3376967464 | 3186994077 | 83597.46 | 3186910480 | 0.94 |
| Prevalence | Oman | 2021 | 36.96 | 49.86 | 26.48 | 0.77 | 3365550 | 5105255047 | 4264815160 | 1682775 | 4263132385 | 0.84 |
| Prevalence | Cook Islands | 2021 | 18.78 | 27.41 | 12.49 | 0.78 | 11633.24 | 5105255047 | 4264826793 | 5816.619 | 4264820977 | 0.84 |
| Prevalence | Belarus | 2021 | 118.10 | 160.40 | 83.71 | 0.78 | 6254502 | 5105255047 | 4271081295 | 3127251 | 4267954044 | 0.84 |
| Prevalence | Canada | 1992 | 85.45 | 112.35 | 62.29 | 0.79 | 18901940 | 3376967464 | 3205896013 | 9450968 | 3196445045 | 0.95 |
| Prevalence | Luxembourg | 1992 | 140.05 | 183.23 | 103.10 | 0.79 | 268190.4 | 3376967464 | 3206164203 | 134095.2 | 3206030108 | 0.95 |
| Prevalence | Hungary | 2021 | 267.13 | 348.20 | 196.41 | 0.79 | 6199148 | 5105255047 | 4277280443 | 3099574 | 4274180869 | 0.84 |
| Prevalence | Greece | 2021 | 104.22 | 134.40 | 79.72 | 0.79 | 6469340 | 5105255047 | 4283749783 | 3234670 | 4280515113 | 0.84 |

| | | | | | | | | | | | | |
|---|---|---|---|---|---|---|---|---|---|---|---|---|
| Prevalence | Serbia | 2021 | 162.77 | 217.15 | 118.32 | 0.79 | 5934126 | 5105255047 | 4289683909 | 2967063 | 4286716846 | 0.84 |
| Prevalence | Sweden | 1992 | 121.69 | 166.11 | 87.86 | 0.80 | 5553552 | 3376967464 | 3211717756 | 2776776 | 3208940980 | 0.95 |
| Prevalence | Montenegro | 2021 | 155.10 | 206.64 | 112.03 | 0.80 | 412912.2 | 5105255047 | 4290096822 | 206456.1 | 4289890365 | 0.84 |
| Prevalence | Croatia | 2021 | 259.46 | 336.61 | 190.41 | 0.80 | 2715472 | 5105255047 | 4292812293 | 1357736 | 4291454558 | 0.84 |
| Prevalence | Japan | 1992 | 115.73 | 155.68 | 83.63 | 0.80 | 88370150 | 3376967464 | 3300087906 | 44185070 | 3255902831 | 0.96 |
| Prevalence | Malta | 2021 | 92.14 | 121.08 | 67.81 | 0.80 | 278137.3 | 5105255047 | 4293090431 | 139068.6 | 4292951362 | 0.84 |
| Prevalence | Netherlands | 1992 | 45.92 | 60.53 | 33.71 | 0.80 | 10396300 | 3376967464 | 3310484202 | 5198148 | 3305286054 | 0.98 |
| Prevalence | Guam | 2021 | 24.45 | 34.44 | 17.12 | 0.80 | 103477.4 | 5105255047 | 4293193908 | 51738.72 | 4293142170 | 0.84 |
| Prevalence | Bahamas | 2021 | 68.35 | 90.61 | 49.95 | 0.81 | 274938.4 | 5105255047 | 4293468847 | 137469.2 | 4293331377 | 0.84 |
| Prevalence | Norway | 1992 | 92.72 | 125.35 | 67.17 | 0.81 | 2768970 | 3376967464 | 3313253172 | 1384485 | 3311868687 | 0.98 |
| Prevalence | Italy | 2021 | 135.72 | 173.15 | 104.63 | 0.81 | 38186670 | 5105255047 | 4331655515 | 19093330 | 4312562181 | 0.84 |
| Prevalence | Denmark | 1992 | 99.30 | 132.10 | 72.00 | 0.81 | 3484450 | 3376967464 | 3316737622 | 1742225 | 3314995397 | 0.98 |
| Prevalence | Russian Federation | 2021 | 143.05 | 192.59 | 102.10 | 0.81 | 96028800 | 5105255047 | 4427684315 | 48014400 | 4379669915 | 0.86 |
| Prevalence | Israel | 2021 | 63.32 | 84.55 | 46.35 | 0.81 | 5769267 | 5105255047 | 4433453582 | 2884633 | 4430568949 | 0.87 |
| Prevalence | Brunei Darussalam | 2021 | 100.12 | 136.99 | 72.41 | 0.81 | 332019.1 | 5105255047 | 4433785601 | 166009.6 | 4433619592 | 0.87 |
| Prevalence | Slovakia | 2021 | 135.00 | 180.46 | 101.94 | 0.81 | 3635982 | 5105255047 | 4437421583 | 1817991 | 4435603592 | 0.87 |
| Prevalence | Poland | 2021 | 57.55 | 75.04 | 42.50 | 0.81 | 25179790 | 5105255047 | 4462601375 | 12589900 | 4450011479 | 0.87 |
| Prevalence | Saudi Arabia | 2021 | 37.84 | 52.42 | 26.38 | 0.82 | 29117100 | 5105255047 | 4491718473 | 14558550 | 4477159924 | 0.88 |
| Prevalence | Bermuda | 2021 | 184.99 | 251.30 | 133.69 | 0.82 | 41969.77 | 5105255047 | 4491760443 | 20984.88 | 4491739458 | 0.88 |
| Prevalence | United States Virgin Islands | 2021 | 108.45 | 143.67 | 78.06 | 0.82 | 53866.94 | 5105255047 | 4491814310 | 26933.47 | 4491787376 | 0.88 |
| Prevalence | San Marino | 1992 | 91.83 | 126.18 | 65.70 | 0.82 | 16789.73 | 3376967464 | 3316754411 | 8394.865 | 3316746017 | 0.98 |
| Prevalence | Puerto Rico | 2021 | 96.55 | 132.74 | 68.95 | 0.83 | 2124836 | 5105255047 | 4493939146 | 1062418 | 4492876728 | 0.88 |
| Prevalence | Greenland | 2021 | 110.09 | 146.01 | 80.38 | 0.83 | 39064.81 | 5105255047 | 4493978210 | 19532.41 | 4493958678 | 0.88 |
| Prevalence | Germany | 1992 | 129.46 | 170.28 | 96.49 | 0.83 | 55437780 | 3376967464 | 3372192189 | 27718890 | 3344473300 | 0.99 |

| | | | | | | | | | | | | |
|---|---|---|---|---|---|---|---|---|---|---|---|---|
| Prevalence | Czechia | 2021 | 263.97 | 357.05 | 190.76 | 0.83 | 6708178 | 5105255047 | 4500686388 | 3354089 | 4497332299 | 0.88 |
| Prevalence | Latvia | 2021 | 191.48 | 260.29 | 136.23 | 0.83 | 1180840 | 5105255047 | 4501867228 | 590420 | 4501276808 | 0.88 |
| Prevalence | Cyprus | 2021 | 94.69 | 126.83 | 69.81 | 0.84 | 940775.7 | 5105255047 | 4502808004 | 470387.8 | 4502337616 | 0.88 |
| Prevalence | France | 2021 | 109.96 | 152.06 | 77.68 | 0.84 | 41021790 | 5105255047 | 4543829797 | 20510900 | 4523318900 | 0.89 |
| Prevalence | Slovenia | 2021 | 287.20 | 396.55 | 205.67 | 0.84 | 1320405 | 5105255047 | 4545150201 | 660202.5 | 4544489999 | 0.89 |
| Prevalence | Australia | 2021 | 88.80 | 120.42 | 63.83 | 0.84 | 16656050 | 5105255047 | 4561806249 | 8328024 | 4553478225 | 0.89 |
| Prevalence | Estonia | 2021 | 235.25 | 327.27 | 168.40 | 0.84 | 825205.3 | 5105255047 | 4562631454 | 412602.6 | 4562218851 | 0.89 |
| Prevalence | Kuwait | 2021 | 48.86 | 67.14 | 34.42 | 0.85 | 3633714 | 5105255047 | 4566265168 | 1816857 | 4564448311 | 0.89 |
| Prevalence | Qatar | 2021 | 50.33 | 69.86 | 35.38 | 0.85 | 2446107 | 5105255047 | 4568711276 | 1223054 | 4567488222 | 0.89 |
| Prevalence | United Arab Emirates | 2021 | 52.72 | 73.17 | 37.62 | 0.85 | 8129433 | 5105255047 | 4576840709 | 4064717 | 4572775992 | 0.90 |
| Prevalence | New Zealand | 2021 | 93.86 | 123.51 | 69.80 | 0.85 | 3378026 | 5105255047 | 4580218735 | 1689013 | 4578529722 | 0.90 |
| Prevalence | Monaco | 1992 | 86.87 | 118.87 | 62.31 | 0.85 | 19942.64 | 3376967464 | 3372212132 | 9971.318 | 3372202160 | 1.00 |
| Prevalence | Belgium | 2021 | 127.40 | 173.85 | 92.20 | 0.85 | 7314275 | 5105255047 | 4587533010 | 3657137 | 4583875872 | 0.90 |
| Prevalence | Austria | 2021 | 256.68 | 354.05 | 182.47 | 0.85 | 5970418 | 5105255047 | 4593503427 | 2985209 | 4590518219 | 0.90 |
| Prevalence | Singapore | 2021 | 102.03 | 138.46 | 74.55 | 0.86 | 4146887 | 5105255047 | 4597650315 | 2073444 | 4595576871 | 0.90 |
| Prevalence | Lithuania | 2021 | 147.58 | 200.80 | 106.55 | 0.86 | 1763517 | 5105255047 | 4599413832 | 881758.6 | 4598532074 | 0.90 |
| Prevalence | United Kingdom | 2021 | 103.85 | 137.05 | 76.67 | 0.86 | 43558640 | 5105255047 | 4642972474 | 21779320 | 4621193153 | 0.91 |
| Prevalence | Finland | 2021 | 186.51 | 253.89 | 134.75 | 0.86 | 3397926 | 5105255047 | 4646370400 | 1698963 | 4644671437 | 0.91 |
| Prevalence | United States of America | 2021 | 133.86 | 170.66 | 102.05 | 0.86 | 216097500 | 5105255047 | 4862467891 | 108048700 | 4754419146 | 0.93 |
| Prevalence | Switzerland | 1992 | 146.94 | 192.52 | 110.03 | 0.87 | 4755332 | 3376967464 | 3376967464 | 2377666 | 3374589798 | 1.00 |
| Prevalence | Andorra | 2021 | 111.34 | 152.64 | 80.46 | 0.87 | 61742.59 | 5105255047 | 4862529634 | 30871.3 | 4862498763 | 0.95 |
| Prevalence | Japan | 2021 | 104.43 | 138.67 | 77.21 | 0.87 | 75404150 | 5105255047 | 4937933788 | 37702080 | 4900231711 | 0.96 |
| Prevalence | Canada | 2021 | 121.38 | 161.99 | 87.75 | 0.87 | 24262340 | 5105255047 | 4962196124 | 12131170 | 4950064956 | 0.97 |
| Prevalence | Ireland | 2021 | 123.56 | 165.99 | 88.81 | 0.87 | 3192765 | 5105255047 | 4965388890 | 1596383 | 4963792507 | 0.97 |

| | | | | | | | | | | | | |
|---|---|---|---|---|---|---|---|---|---|---|---|---|
| Prevalence | Taiwan (Province of China) | 2021 | 18.91 | 28.28 | 12.49 | 0.87 | 16681750 | 5105255047 | 4982070642 | 8340876 | 4973729766 | 0.97 |
| Prevalence | Iceland | 2021 | 186.79 | 253.76 | 134.03 | 0.88 | 227618.6 | 5105255047 | 4982298260 | 113809.3 | 4982184451 | 0.98 |
| Prevalence | Luxembourg | 2021 | 170.90 | 230.35 | 121.84 | 0.88 | 447039.5 | 5105255047 | 4982745300 | 223519.8 | 4982521780 | 0.98 |
| Prevalence | Republic of Korea | 2021 | 96.79 | 132.55 | 69.00 | 0.89 | 36999740 | 5105255047 | 5019745039 | 18499870 | 5001245169 | 0.98 |
| Prevalence | Sweden | 2021 | 159.41 | 213.06 | 113.98 | 0.89 | 6417083 | 5105255047 | 5026162122 | 3208541 | 5022953580 | 0.98 |
| Prevalence | San Marino | 2021 | 107.63 | 146.12 | 77.76 | 0.89 | 21290.2 | 5105255047 | 5026183412 | 10645.1 | 5026172767 | 0.98 |
| Prevalence | Netherlands | 2021 | 51.42 | 68.85 | 36.82 | 0.89 | 11069920 | 5105255047 | 5037253336 | 5534962 | 5031718374 | 0.99 |
| Prevalence | Denmark | 2021 | 146.33 | 193.77 | 106.86 | 0.90 | 3719018 | 5105255047 | 5040972354 | 1859509 | 5039112845 | 0.99 |
| Prevalence | Germany | 2021 | 165.25 | 219.61 | 120.02 | 0.90 | 54851780 | 5105255047 | 5095824131 | 27425890 | 5068398243 | 0.99 |
| Prevalence | Monaco | 2021 | 108.31 | 149.04 | 78.65 | 0.91 | 23218.3 | 5105255047 | 5095847350 | 11609.15 | 5095835740 | 1.00 |
| Prevalence | Norway | 2021 | 112.50 | 151.74 | 80.80 | 0.92 | 3522494 | 5105255047 | 5099369844 | 1761247 | 5097608597 | 1.00 |
| Prevalence | Switzerland | 2021 | 155.87 | 212.33 | 111.58 | 0.93 | 5885204 | 5105255047 | 5105255047 | 2942602 | 5102312446 | 1.00 |
| Prevalence | Somalia | 1992 | 4.12 | 6.21 | 2.60 | 0.05 | 3816581 | 3376967464 | 3816581 | 1908290 | 1908290.3 | 0.00 |
| Prevalence | Somalia | 2021 | 3.08 | 4.62 | 1.96 | 0.08 | 10890630 | 5105255047 | 10890631 | 5445315 | 5445315.5 | 0.00 |
| Prevalence | Niger | 1992 | 4.52 | 6.44 | 3.02 | 0.08 | 4059013 | 3376967464 | 7875594 | 2029507 | 5846087.2 | 0.00 |
| Prevalence | Chad | 1992 | 4.47 | 6.32 | 3.03 | 0.12 | 3037794 | 3376967464 | 10913388 | 1518897 | 9394490.9 | 0.00 |
| Prevalence | Mali | 1992 | 4.86 | 7.00 | 3.17 | 0.13 | 4416030 | 3376967464 | 15329418 | 2208015 | 13121403 | 0.00 |
| Prevalence | Burkina Faso | 1992 | 5.09 | 7.16 | 3.45 | 0.14 | 4697926 | 3376967464 | 20027344 | 2348963 | 17678381.3 | 0.01 |
| Prevalence | Ethiopia | 1992 | 4.10 | 6.10 | 2.62 | 0.15 | 27028040 | 3376967464 | 47055389 | 13514020 | 33541366.7 | 0.01 |
| Prevalence | Niger | 2021 | 4.39 | 6.19 | 3.00 | 0.17 | 11701660 | 5105255047 | 22592286 | 5850828 | 16741458.7 | 0.00 |
| Prevalence | Mozambique | 1992 | 4.88 | 7.29 | 3.11 | 0.18 | 6841201 | 3376967464 | 53896590 | 3420600 | 50475989.3 | 0.01 |
| Prevalence | Afghanistan | 1992 | 14.27 | 20.02 | 9.97 | 0.18 | 5317936 | 3376967464 | 59214526 | 2658968 | 56555557.9 | 0.02 |
| Prevalence | Guinea | 1992 | 5.80 | 8.08 | 3.99 | 0.18 | 3194335 | 3376967464 | 62408861 | 1597167 | 60811693.6 | 0.02 |
| Prevalence | Uganda | 1992 | 4.32 | 6.47 | 2.77 | 0.19 | 8871780 | 3376967464 | 71280641 | 4435890 | 66844751.1 | 0.02 |

| | | | | | | | | | | | | | | |
|---|---|---|---|---|---|---|---|---|---|---|---|---|---|---|
| Prevalence | Malawi | 1992 | 4.21 | 6.28 | 2.65 | 0.21 | 5373904 | 3376967464 | 76654545 | 2686952 | 73967593.3 | 0.02 | | |
| Prevalence | Burundi | 1992 | 4.47 | 6.63 | 2.89 | 0.21 | 2868701 | 3376967464 | 79523246 | 1434350 | 78088895.8 | 0.02 | | |
| Prevalence | South Sudan | 1992 | 4.66 | 6.93 | 3.00 | 0.21 | 3170246 | 3376967464 | 82693492 | 1585123 | 81108369 | 0.02 | | |
| Prevalence | Nepal | 1992 | 6.92 | 10.14 | 4.41 | 0.21 | 10843820 | 3376967464 | 93537312 | 5421910 | 88115402.1 | 0.03 | | |
| Prevalence | Sierra Leone | 1992 | 4.95 | 6.97 | 3.33 | 0.21 | 2160724 | 3376967464 | 95698036 | 1080362 | 94617674.1 | 0.03 | | |
| Prevalence | Guinea-Bissau | 1992 | 4.41 | 6.21 | 3.00 | 0.22 | 518730.4 | 3376967464 | 96216766 | 259365.2 | 95957401.1 | 0.03 | | |
| Prevalence | Central African Republic | 1992 | 4.22 | 6.30 | 2.69 | 0.22 | 1511635 | 3376967464 | 97728401 | 755817.3 | 96972583.7 | 0.03 | | |
| Prevalence | Benin | 1992 | 4.97 | 6.93 | 3.42 | 0.23 | 2437709 | 3376967464 | 100166110 | 1218855 | 98947255.7 | 0.03 | | |
| Prevalence | Bhutan | 1992 | 6.60 | 9.81 | 4.22 | 0.23 | 362111 | 3376967464 | 100528221 | 181055.5 | 100347165.9 | 0.03 | | |
| Prevalence | Eritrea | 1992 | 4.08 | 6.02 | 2.62 | 0.23 | 1636011 | 3376967464 | 102164232 | 818005.3 | 101346226.7 | 0.03 | | |
| Prevalence | Yemen | 1992 | 11.19 | 15.77 | 7.64 | 0.23 | 6649314 | 3376967464 | 108813546 | 3324657 | 105488888.8 | 0.03 | | |
| Prevalence | Liberia | 1992 | 6.61 | 9.25 | 4.51 | 0.24 | 970267.7 | 3376967464 | 109783813 | 485133.8 | 109298679.5 | 0.03 | | |
| Prevalence | Chad | 2021 | 4.69 | 6.58 | 3.27 | 0.24 | 8325851 | 5105255047 | 30918137 | 4162925 | 26755211.9 | 0.01 | | |
| Prevalence | Bangladesh | 1992 | 6.30 | 9.32 | 4.01 | 0.25 | 59911290 | 3376967464 | 169695106 | 29955650 | 139739459.7 | 0.04 | | |
| Prevalence | Senegal | 1992 | 4.94 | 6.99 | 3.30 | 0.25 | 3982520 | 3376967464 | 173677626 | 1991260 | 171686366 | 0.05 | | |
| Prevalence | Gambia | 1992 | 4.81 | 6.75 | 3.23 | 0.25 | 540094.8 | 3376967464 | 174217721 | 270047.4 | 173947673.4 | 0.05 | | |
| Prevalence | United Republic of Tanzania | 1992 | 5.58 | 8.14 | 3.69 | 0.26 | 13815680 | 3376967464 | 188033398 | 6907839 | 181125559.4 | 0.05 | | |
| Prevalence | Mali | 2021 | 4.66 | 6.60 | 3.08 | 0.27 | 11895330 | 5105255047 | 42813466 | 5947664 | 36865801.5 | 0.01 | | |
| Prevalence | Lao People's Democratic Republic | 1992 | 3.60 | 5.52 | 2.22 | 0.27 | 2290031 | 3376967464 | 190323429 | 1145016 | 189178413.7 | 0.06 | | |
| Prevalence | Rwanda | 1992 | 4.85 | 7.10 | 3.19 | 0.28 | 3779329 | 3376967464 | 194102758 | 1889664 | 192213093.7 | 0.06 | | |
| Prevalence | South Sudan | 2021 | 5.28 | 7.84 | 3.40 | 0.28 | 5135653 | 5105255047 | 47949119 | 2567827 | 45381292.2 | 0.01 | | |
| Prevalence | Togo | 1992 | 4.53 | 6.40 | 3.09 | 0.28 | 1939975 | 3376967464 | 196042733 | 969987.5 | 195072745.7 | 0.06 | | |
| Prevalence | Angola | 1992 | 4.56 | 6.73 | 2.90 | 0.28 | 5702682 | 3376967464 | 201745415 | 2851341 | 198894074.2 | 0.06 | | |

| | | | | | | | | | | | | |
|---|---|---|---|---|---|---|---|---|---|---|---|---|
| Prevalence | Timor-Leste | 1992 | 3.35 | 5.17 | 2.07 | 0.28 | 447494.9 | 3376967464 | 202192910 | 223747.4 | 201969162.6 | 0.06 |
| Prevalence | Madagascar | 1992 | 5.73 | 8.22 | 3.85 | 0.28 | 6432226 | 3376967464 | 208625136 | 3216113 | 205409023.2 | 0.06 |
| Prevalence | Equatorial Guinea | 1992 | 5.27 | 7.70 | 3.44 | 0.28 | 229117.5 | 3376967464 | 208854254 | 114558.8 | 208739695 | 0.06 |
| Prevalence | Burkina Faso | 2021 | 5.53 | 7.74 | 3.82 | 0.29 | 11698660 | 5105255047 | 59647781 | 5849331 | 53798449.8 | 0.01 |
| Prevalence | Cabo Verde | 1992 | 6.67 | 9.28 | 4.62 | 0.29 | 183931.3 | 3376967464 | 209038185 | 91965.67 | 208946219.4 | 0.06 |
| Prevalence | Comoros | 1992 | 5.54 | 8.11 | 3.61 | 0.29 | 249116.1 | 3376967464 | 209287301 | 124558 | 209162743.1 | 0.06 |
| Prevalence | Burundi | 2021 | 4.18 | 6.15 | 2.67 | 0.29 | 7040681 | 5105255047 | 66688462 | 3520341 | 63168121.4 | 0.01 |
| Prevalence | Democratic Republic of the Congo | 1992 | 5.09 | 7.44 | 3.32 | 0.29 | 20457730 | 3376967464 | 229745032 | 10228870 | 219516166.6 | 0.07 |
| Prevalence | Coted'Ivoire | 1992 | 5.26 | 7.29 | 3.58 | 0.29 | 6800901 | 3376967464 | 236545933 | 3400450 | 233145482.4 | 0.07 |
| Prevalence | Cambodia | 1992 | 3.34 | 5.18 | 2.02 | 0.30 | 5566488 | 3376967464 | 242112421 | 2783244 | 239329176.7 | 0.07 |
| Prevalence | Sudan | 1992 | 12.01 | 16.98 | 8.23 | 0.30 | 11202790 | 3376967464 | 253315211 | 5601395 | 247713815.6 | 0.07 |
| Prevalence | Zambia | 1992 | 4.31 | 6.43 | 2.76 | 0.31 | 4197849 | 3376967464 | 257513060 | 2098925 | 255414135.1 | 0.08 |
| Prevalence | Central African Republic | 2021 | 4.30 | 6.29 | 2.78 | 0.31 | 3075108 | 5105255047 | 69763570 | 1537554 | 68226015.8 | 0.01 |
| Prevalence | Nigeria | 1992 | 5.94 | 8.34 | 4.01 | 0.31 | 50478270 | 3376967464 | 307991334 | 25239140 | 282752197 | 0.08 |
| Prevalence | Solomon Islands | 1992 | 5.41 | 7.96 | 3.45 | 0.31 | 187733.3 | 3376967464 | 308179068 | 93866.66 | 308085200.9 | 0.09 |
| Prevalence | Sao Tome and Principe | 1992 | 6.09 | 8.60 | 4.19 | 0.31 | 61668.72 | 3376967464 | 308240736 | 30834.36 | 308209902 | 0.09 |
| Prevalence | Cameroon | 1992 | 6.30 | 8.66 | 4.37 | 0.32 | 5631620 | 3376967464 | 313872356 | 2815810 | 311056546.2 | 0.09 |
| Prevalence | Guatemala | 1992 | 21.96 | 30.64 | 15.09 | 0.32 | 4330417 | 3376967464 | 318202773 | 2165209 | 316037564.7 | 0.09 |
| Prevalence | Papua New Guinea | 1992 | 4.92 | 7.39 | 3.04 | 0.32 | 2419850 | 3376967464 | 320622623 | 1209925 | 319412698.1 | 0.09 |
| Prevalence | Haiti | 1992 | 21.99 | 30.19 | 15.50 | 0.32 | 3615033 | 3376967464 | 324237656 | 1807516 | 322430139.3 | 0.10 |
| Prevalence | Pakistan | 1992 | 7.54 | 10.98 | 4.90 | 0.32 | 60357000 | 3376967464 | 384594651 | 30178500 | 354416153.3 | 0.10 |
| Prevalence | Myanmar | 1992 | 4.03 | 6.07 | 2.49 | 0.33 | 24926270 | 3376967464 | 409520920 | 12463130 | 397057785.4 | 0.12 |
| Prevalence | Mozambique | 2021 | 5.23 | 7.65 | 3.46 | 0.33 | 16039550 | 5105255047 | 85803118 | 8019774 | 77783343.8 | 0.02 |

| | | | | | | | | | | | | |
|---|---|---|---|---|---|---|---|---|---|---|---|---|
| Prevalence | Guinea | 2021 | 5.72 | 7.93 | 4.02 | 0.34 | 6958615 | 5105255047 | 92761733 | 3479307 | 89282425.3 | 0.02 |
| Prevalence | Afghanistan | 2021 | 8.99 | 12.70 | 6.25 | 0.34 | 16398400 | 5105255047 | 109160135 | 8199201 | 100960934 | 0.02 |
| Prevalence | Djibouti | 1992 | 4.48 | 6.57 | 2.91 | 0.34 | 281064.1 | 3376967464 | 409801984 | 140532.1 | 409661452 | 0.12 |
| Prevalence | Honduras | 1992 | 23.04 | 31.92 | 16.09 | 0.34 | 2511178 | 3376967464 | 412313162 | 1255589 | 411057573.1 | 0.12 |
| Prevalence | India | 1992 | 7.53 | 10.99 | 4.90 | 0.34 | 515839400 | 3376967464 | 928152513 | 257919700 | 670232837.4 | 0.20 |
| Prevalence | Kenya | 1992 | 4.56 | 6.73 | 2.95 | 0.35 | 12235470 | 3376967464 | 940387986 | 6117737 | 934270249.4 | 0.28 |
| Prevalence | Mauritania | 1992 | 6.57 | 9.05 | 4.57 | 0.35 | 1105278 | 3376967464 | 941493264 | 552639.1 | 940940625.2 | 0.28 |
| Prevalence | Lesotho | 1992 | 6.55 | 9.62 | 4.20 | 0.35 | 821010.8 | 3376967464 | 942314275 | 410505.4 | 941903769.7 | 0.28 |
| Prevalence | Liberia | 2021 | 6.35 | 8.71 | 4.43 | 0.35 | 3135229 | 5105255047 | 112295364 | 1567614 | 110727749.6 | 0.02 |
| Prevalence | Guinea-Bissau | 2021 | 4.83 | 6.78 | 3.34 | 0.35 | 1119372 | 5105255047 | 113414736 | 559686.2 | 112855050.1 | 0.02 |
| Prevalence | Nicaragua | 1992 | 25.51 | 34.80 | 18.26 | 0.36 | 2103741 | 3376967464 | 944418017 | 1051871 | 943366145.8 | 0.28 |
| Prevalence | Maldives | 1992 | 6.53 | 9.85 | 4.17 | 0.36 | 118284.3 | 3376967464 | 944536301 | 59142.14 | 944477158.7 | 0.28 |
| Prevalence | Sierra Leone | 2021 | 5.29 | 7.37 | 3.64 | 0.36 | 5014942 | 5105255047 | 118429679 | 2507471 | 115922207.5 | 0.02 |
| Prevalence | Ethiopia | 2021 | 3.81 | 5.67 | 2.41 | 0.36 | 61363610 | 5105255047 | 179793292 | 30681810 | 149111485.3 | 0.03 |
| Prevalence | Vanuatu | 1992 | 5.56 | 8.15 | 3.46 | 0.36 | 84103.98 | 3376967464 | 944620405 | 42051.99 | 944578352.8 | 0.28 |
| Prevalence | Morocco | 1992 | 16.22 | 22.43 | 11.30 | 0.37 | 15160090 | 3376967464 | 959780492 | 7580044 | 952200448.5 | 0.28 |
| Prevalence | Benin | 2021 | 5.74 | 7.90 | 4.00 | 0.37 | 7048559 | 5105255047 | 186841851 | 3524280 | 183317571.5 | 0.04 |
| Prevalence | El Salvador | 1992 | 27.63 | 37.89 | 19.41 | 0.38 | 3000660 | 3376967464 | 962781152 | 1500330 | 961280822.3 | 0.28 |
| Prevalence | Democratic Republic of the Congo | 2021 | 5.65 | 8.23 | 3.73 | 0.38 | 49675100 | 5105255047 | 236516952 | 24837550 | 211679401.7 | 0.04 |
| Prevalence | Malawi | 2021 | 4.78 | 7.08 | 3.12 | 0.38 | 10785330 | 5105255047 | 247302286 | 5392667 | 241909619.1 | 0.05 |
| Prevalence | Ghana | 1992 | 5.57 | 7.89 | 3.81 | 0.38 | 8290370 | 3376967464 | 971071522 | 4145185 | 966926337.2 | 0.29 |
| Prevalence | Madagascar | 2021 | 6.20 | 8.77 | 4.17 | 0.40 | 16136660 | 5105255047 | 263438948 | 8068331 | 255370616.9 | 0.05 |
| Prevalence | Eritrea | 2021 | 4.36 | 6.42 | 2.83 | 0.40 | 3903951 | 5105255047 | 267342899 | 1951975 | 265390923.2 | 0.05 |

| | | | | | | | | | | | | |
|---|---|---|---|---|---|---|---|---|---|---|---|---|
| Prevalence | Senegal | 2021 | 6.04 | 8.44 | 4.20 | 0.41 | 8916317 | 5105255047 | 276259216 | 4458159 | 271801057.2 | 0.05 |
| Prevalence | Togo | 2021 | 6.64 | 9.08 | 4.68 | 0.41 | 4808399 | 5105255047 | 281067615 | 2404199 | 278663415.3 | 0.05 |
| Prevalence | Gambia | 2021 | 5.93 | 8.20 | 4.10 | 0.41 | 1328594 | 5105255047 | 282396209 | 664296.9 | 281731911.6 | 0.06 |
| Prevalence | Palestine | 1992 | 14.48 | 20.51 | 9.76 | 0.41 | 1092733 | 3376967464 | 972164255 | 546366.4 | 971617888.3 | 0.29 |
| Prevalence | Zimbabwe | 1992 | 6.83 | 9.77 | 4.54 | 0.41 | 5556213 | 3376967464 | 977720467 | 2778106 | 974942361.1 | 0.29 |
| Prevalence | Kiribati | 1992 | 6.10 | 9.00 | 3.92 | 0.42 | 43348.47 | 3376967464 | 977763816 | 21674.24 | 977742141.7 | 0.29 |
| Prevalence | Eswatini | 1992 | 6.71 | 9.65 | 4.30 | 0.42 | 429553.2 | 3376967464 | 978193369 | 214776.6 | 977978592.6 | 0.29 |
| Prevalence | Papua New Guinea | 2021 | 6.41 | 9.33 | 4.06 | 0.42 | 6230283 | 5105255047 | 288626492 | 3115142 | 285511350.2 | 0.06 |
| Prevalence | Viet Nam | 1992 | 5.01 | 7.48 | 3.11 | 0.42 | 40268370 | 3376967464 | 1018461738 | 20134180 | 998327553.6 | 0.30 |
| Prevalence | Iraq | 1992 | 14.23 | 20.33 | 9.65 | 0.42 | 10043140 | 3376967464 | 1028504876 | 5021569 | 1023483307 | 0.30 |
| Prevalence | Tuvalu | 1992 | 8.39 | 12.21 | 5.45 | 0.42 | 5474.875 | 3376967464 | 1028510350 | 2737.437 | 1028507613 | 0.30 |
| Prevalence | Uganda | 2021 | 4.86 | 7.08 | 3.19 | 0.42 | 22465620 | 5105255047 | 311092115 | 11232810 | 299859303.5 | 0.06 |
| Prevalence | Coted'Ivoire | 2021 | 6.89 | 9.39 | 4.85 | 0.43 | 15562160 | 5105255047 | 326654276 | 7781080 | 318873195.4 | 0.06 |
| Prevalence | Solomon Islands | 2021 | 7.88 | 11.79 | 5.10 | 0.43 | 400945.3 | 5105255047 | 327055221 | 200472.7 | 326854748.6 | 0.06 |
| Prevalence | Congo | 1992 | 5.48 | 8.14 | 3.54 | 0.43 | 1365818 | 3376967464 | 1029876169 | 682909 | 1029193260 | 0.30 |
| Prevalence | Nepal | 2021 | 11.35 | 16.31 | 7.59 | 0.43 | 19987970 | 5105255047 | 347043194 | 9993986 | 337049207.6 | 0.07 |
| Prevalence | Bolivia (Plurinational State of) | 1992 | 25.33 | 35.14 | 17.78 | 0.43 | 3645533 | 3376967464 | 1033521701 | 1822766 | 1031698935 | 0.31 |
| Prevalence | Rwanda | 2021 | 5.59 | 8.33 | 3.60 | 0.44 | 7848418 | 5105255047 | 354891612 | 3924209 | 350967402.9 | 0.07 |
| Prevalence | Egypt | 1992 | 19.09 | 26.59 | 13.21 | 0.44 | 33112790 | 3376967464 | 1066634488 | 16556390 | 1050078095 | 0.31 |
| Prevalence | Botswana | 1992 | 6.60 | 9.42 | 4.32 | 0.44 | 747651.5 | 3376967464 | 1067382140 | 373825.7 | 1067008314 | 0.32 |
| Prevalence | Marshall Islands | 1992 | 5.00 | 7.38 | 3.14 | 0.44 | 23628.24 | 3376967464 | 1067405768 | 11814.12 | 1067393954 | 0.32 |
| Prevalence | Belize | 1992 | 27.09 | 36.89 | 19.04 | 0.44 | 103326.3 | 3376967464 | 1067509094 | 51663.13 | 1067457431 | 0.32 |
| Prevalence | Timor-Leste | 2021 | 5.19 | 7.74 | 3.32 | 0.44 | 802737.4 | 5105255047 | 355694349 | 401368.7 | 355292980.6 | 0.07 |
| Prevalence | Syrian Arab Republic | 1992 | 27.62 | 37.39 | 20.19 | 0.45 | 6931728 | 3376967464 | 1074440822 | 3465864 | 1070974958 | 0.32 |

| | | | | | | | | | | | | |
|---|---|---|---|---|---|---|---|---|---|---|---|---|
| Prevalence | United Republic of Tanzania | 2021 | 6.42 | 9.26 | 4.24 | 0.45 | 32205890 | 5105255047 | 387900240 | 16102950 | 371797294.8 | 0.07 |
| Prevalence | Haiti | 2021 | 26.12 | 35.54 | 18.52 | 0.45 | 8005224 | 5105255047 | 395905464 | 4002612 | 391902852.4 | 0.08 |
| Prevalence | Dominican Republic | 1992 | 25.06 | 34.48 | 17.62 | 0.45 | 4367623 | 3376967464 | 1078808445 | 2183812 | 1076624634 | 0.32 |
| Prevalence | Yemen | 2021 | 12.37 | 16.99 | 8.73 | 0.45 | 18839690 | 5105255047 | 414745156 | 9419846 | 405325310.2 | 0.08 |
| Prevalence | Angola | 2021 | 5.62 | 8.17 | 3.68 | 0.45 | 16719890 | 5105255047 | 431465050 | 8359947 | 423105102.9 | 0.08 |
| Prevalence | Grenada | 1992 | 36.81 | 49.43 | 26.48 | 0.46 | 48427.92 | 3376967464 | 1078856873 | 24213.96 | 1078832659 | 0.32 |
| Prevalence | Oman | 1992 | 13.55 | 19.19 | 9.00 | 0.46 | 1153630 | 3376967464 | 1080010503 | 576815.1 | 1079433688 | 0.32 |
| Prevalence | Namibia | 1992 | 6.83 | 9.80 | 4.52 | 0.46 | 804903.9 | 3376967464 | 1080815407 | 402451.9 | 1080412955 | 0.32 |
| Prevalence | Guyana | 1992 | 36.30 | 48.02 | 26.80 | 0.47 | 459165.3 | 3376967464 | 1081274573 | 229582.7 | 1081044990 | 0.32 |
| Prevalence | Gabon | 1992 | 7.37 | 10.66 | 4.90 | 0.47 | 557425.5 | 3376967464 | 1081831998 | 278712.8 | 1081553285 | 0.32 |
| Prevalence | Bhutan | 2021 | 12.68 | 18.34 | 8.37 | 0.47 | 519639.6 | 5105255047 | 431984689 | 259819.8 | 431724869.5 | 0.08 |
| Prevalence | Vanuatu | 2021 | 9.07 | 13.24 | 5.95 | 0.47 | 184213.1 | 5105255047 | 432168902 | 92106.55 | 432076795.9 | 0.08 |
| Prevalence | Cambodia | 2021 | 6.69 | 9.83 | 4.17 | 0.47 | 10996940 | 5105255047 | 443165844 | 5498471 | 437667373 | 0.09 |
| Prevalence | Micronesia (Federated States of) | 1992 | 6.50 | 9.69 | 4.18 | 0.47 | 55679.86 | 3376967464 | 1081887678 | 27839.93 | 1081859838 | 0.32 |
| Prevalence | Zimbabwe | 2021 | 7.62 | 10.79 | 5.20 | 0.47 | 8807361 | 5105255047 | 451973205 | 4403681 | 447569524.4 | 0.09 |
| Prevalence | Tajikistan | 1992 | 26.99 | 37.45 | 19.09 | 0.48 | 2943035 | 3376967464 | 1084830713 | 1471517 | 1083359195 | 0.32 |
| Prevalence | Indonesia | 1992 | 4.49 | 6.75 | 2.79 | 0.48 | 115716600 | 3376967464 | 1200547263 | 57858280 | 1142688988 | 0.34 |
| Prevalence | China | 1992 | 7.29 | 10.75 | 4.66 | 0.48 | 815239200 | 3376967464 | 2015786424 | 407619600 | 1608166844 | 0.48 |
| Prevalence | Algeria | 1992 | 17.90 | 24.92 | 12.65 | 0.48 | 14528470 | 3376967464 | 2030314898 | 7264237 | 2023050661 | 0.60 |
| Prevalence | Comoros | 2021 | 6.85 | 10.17 | 4.40 | 0.48 | 467233.1 | 5105255047 | 452440438 | 233616.6 | 452206821.7 | 0.09 |
| Prevalence | Turkey | 1992 | 24.05 | 33.24 | 16.69 | 0.48 | 36334360 | 3376967464 | 2066649254 | 18167180 | 2048482076 | 0.61 |
| Prevalence | Mongolia | 1992 | 25.71 | 35.88 | 17.93 | 0.48 | 1235917 | 3376967464 | 2067885171 | 617958.5 | 2067267212 | 0.61 |
| Prevalence | Paraguay | 1992 | 39.92 | 54.12 | 28.53 | 0.48 | 2324234 | 3376967464 | 2070209405 | 1162117 | 2069047288 | 0.61 |
| Prevalence | Cameroon | 2021 | 7.30 | 10.17 | 5.13 | 0.48 | 17450670 | 5105255047 | 469891104 | 8725333 | 461165771 | 0.09 |

| | | | | | | | | | | | | |
|---|---|---|---|---|---|---|---|---|---|---|---|---|
| Prevalence | Iran (Islamic Republic of) | 1992 | 19.28 | 26.53 | 13.69 | 0.48 | 32234900 | 3376967464 | 2102444303 | 16117450 | 2086326854 | 0.62 |
| Prevalence | Colombia | 1992 | 44.41 | 58.58 | 32.66 | 0.48 | 20427380 | 3376967464 | 2122871685 | 10213690 | 2112657994 | 0.63 |
| Prevalence | Saint Vincent and the Grenadines | 1992 | 31.58 | 42.89 | 22.64 | 0.49 | 63082.62 | 3376967464 | 2122934768 | 31541.31 | 2122903226 | 0.63 |
| Prevalence | Djibouti | 2021 | 6.95 | 10.08 | 4.56 | 0.49 | 805751.6 | 5105255047 | 470696855 | 402875.8 | 470293979.5 | 0.09 |
| Prevalence | Tunisia | 1992 | 22.42 | 31.05 | 15.72 | 0.49 | 5133459 | 3376967464 | 2128068226 | 2566729 | 2125501497 | 0.63 |
| Prevalence | Lao People's Democratic Republic | 2021 | 5.90 | 8.99 | 3.72 | 0.49 | 4754006 | 5105255047 | 475450861 | 2377003 | 473073858.3 | 0.09 |
| Prevalence | Bangladesh | 2021 | 11.88 | 17.35 | 7.90 | 0.49 | 107272700 | 5105255047 | 582723583 | 53636360 | 529087222.1 | 0.10 |
| Prevalence | Samoa | 1992 | 8.80 | 12.96 | 5.76 | 0.49 | 92361.26 | 3376967464 | 2128160588 | 46180.63 | 2128114407 | 0.63 |
| Prevalence | Mauritania | 2021 | 7.92 | 10.90 | 5.48 | 0.50 | 2374531 | 5105255047 | 585098113 | 1187265 | 583910848.2 | 0.11 |
| Prevalence | Democratic People's Republic of Korea | 1992 | 7.94 | 11.69 | 5.03 | 0.50 | 13875620 | 3376967464 | 2142036206 | 6937809 | 2135098397 | 0.63 |
| Prevalence | Uzbekistan | 1992 | 32.99 | 45.69 | 23.17 | 0.50 | 12096580 | 3376967464 | 2154132781 | 6048288 | 2148084494 | 0.64 |
| Prevalence | Nigeria | 2021 | 5.68 | 7.99 | 3.88 | 0.50 | 123377300 | 5105255047 | 708475425 | 61688660 | 646786769.3 | 0.13 |
| Prevalence | Tonga | 1992 | 9.94 | 14.43 | 6.52 | 0.50 | 53374.01 | 3376967464 | 2154186155 | 26687 | 2154159468 | 0.64 |
| Prevalence | Pakistan | 2021 | 9.49 | 13.67 | 6.36 | 0.50 | 141565600 | 5105255047 | 850041000 | 70782790 | 779258212.8 | 0.15 |
| Prevalence | Sao Tome and Principe | 2021 | 8.65 | 12.02 | 6.10 | 0.51 | 131024.1 | 5105255047 | 850172025 | 65512.04 | 850106512.5 | 0.17 |
| Prevalence | Zambia | 2021 | 5.50 | 7.94 | 3.59 | 0.51 | 10789070 | 5105255047 | 860961092 | 5394534 | 855566558.4 | 0.17 |
| Prevalence | Brazil | 1992 | 46.76 | 62.98 | 33.64 | 0.51 | 93825900 | 3376967464 | 2248012056 | 46912950 | 2201099106 | 0.65 |
| Prevalence | Lesotho | 2021 | 7.59 | 10.73 | 5.10 | 0.51 | 1159988 | 5105255047 | 862121080 | 579994.1 | 861541086.2 | 0.17 |
| Prevalence | Suriname | 1992 | 33.97 | 46.30 | 24.06 | 0.51 | 240092.4 | 3376967464 | 2248252148 | 120046.2 | 2248132102 | 0.67 |
| Prevalence | Honduras | 2021 | 38.48 | 51.79 | 27.58 | 0.51 | 6325127 | 5105255047 | 868446208 | 3162564 | 865283644 | 0.17 |
| Prevalence | Peru | 1992 | 27.26 | 37.35 | 19.17 | 0.51 | 13017280 | 3376967464 | 2261269426 | 6508639 | 2254760787 | 0.67 |
| Prevalence | Mexico | 1992 | 34.87 | 47.94 | 24.34 | 0.51 | 51527500 | 3376967464 | 2312796928 | 25763750 | 2287033177 | 0.68 |

| | | | | | | | | | | | | |
|---|---|---|---|---|---|---|---|---|---|---|---|---|
| Prevalence | Saint Lucia | 1992 | 34.11 | 45.92 | 24.47 | 0.52 | 80360.32 | 3376967464 | 2312877288 | 40180.16 | 2312837108 | 0.68 |
| Prevalence | Philippines | 1992 | 3.83 | 5.90 | 2.33 | 0.52 | 37716100 | 3376967464 | 2350593387 | 18858050 | 2331735338 | 0.69 |
| Prevalence | Ecuador | 1992 | 33.86 | 44.90 | 24.84 | 0.52 | 6008273 | 3376967464 | 2356601661 | 3004137 | 2353597524 | 0.70 |
| Prevalence | Kenya | 2021 | 5.97 | 8.76 | 3.92 | 0.52 | 29742370 | 5105255047 | 898188583 | 14871190 | 883317395.1 | 0.17 |
| Prevalence | Nicaragua | 2021 | 49.01 | 66.62 | 34.95 | 0.52 | 4297839 | 5105255047 | 902486421 | 2148919 | 900337502 | 0.18 |
| Prevalence | Thailand | 1992 | 6.72 | 10.03 | 4.30 | 0.53 | 39057400 | 3376967464 | 2395659056 | 19528700 | 2376130359 | 0.70 |
| Prevalence | Kiribati | 2021 | 10.29 | 14.94 | 6.82 | 0.53 | 74513.75 | 5105255047 | 902560935 | 37256.87 | 902523678.2 | 0.18 |
| Prevalence | Kyrgyzstan | 1992 | 38.30 | 52.98 | 26.90 | 0.53 | 2635720 | 3376967464 | 2398294776 | 1317860 | 2396976916 | 0.71 |
| Prevalence | Venezuela (Bolivarian Republic of) | 1992 | 37.82 | 50.81 | 27.50 | 0.53 | 11607490 | 3376967464 | 2409902266 | 5803745 | 2404098521 | 0.71 |
| Prevalence | Tokelau | 1992 | 10.00 | 14.58 | 6.60 | 0.53 | 834.3087 | 3376967464 | 2409903100 | 417.1543 | 2409902683 | 0.71 |
| Prevalence | Sri Lanka | 1992 | 10.17 | 14.40 | 6.75 | 0.53 | 11121210 | 3376967464 | 2421024309 | 5560605 | 2415463705 | 0.72 |
| Prevalence | Cabo Verde | 2021 | 12.18 | 16.74 | 8.66 | 0.53 | 382357.4 | 5105255047 | 902943292 | 191178.7 | 902752113.8 | 0.18 |
| Prevalence | Myanmar | 2021 | 8.07 | 11.96 | 5.09 | 0.53 | 36996570 | 5105255047 | 939939864 | 18498290 | 921441578.5 | 0.18 |
| Prevalence | Nauru | 1992 | 7.75 | 11.52 | 4.93 | 0.54 | 5770.117 | 3376967464 | 2421030079 | 2885.058 | 2421027194 | 0.72 |
| Prevalence | Bosnia and Herzegovina | 1992 | 100.56 | 133.90 | 71.27 | 0.54 | 3112775 | 3376967464 | 2424142855 | 1556388 | 2422586467 | 0.72 |
| Prevalence | Guatemala | 2021 | 35.47 | 49.03 | 25.05 | 0.54 | 9913414 | 5105255047 | 949853279 | 4956707 | 944896571.5 | 0.19 |
| Prevalence | Tajikistan | 2021 | 41.73 | 56.81 | 29.73 | 0.54 | 6207893 | 5105255047 | 956061172 | 3103947 | 952957225.2 | 0.19 |
| Prevalence | Lebanon | 1992 | 30.27 | 41.39 | 21.70 | 0.54 | 1853583 | 3376967464 | 2425996438 | 926791.5 | 2425069646 | 0.72 |
| Prevalence | Sudan | 2021 | 15.93 | 22.09 | 11.12 | 0.54 | 25440510 | 5105255047 | 981501682 | 12720260 | 968781427 | 0.19 |
| Prevalence | Fiji | 1992 | 6.51 | 9.65 | 4.08 | 0.54 | 465040.9 | 3376967464 | 2426461479 | 232520.5 | 2426228958 | 0.72 |
| Prevalence | Costa Rica | 1992 | 49.25 | 65.98 | 36.48 | 0.54 | 1893843 | 3376967464 | 2428355322 | 946921.6 | 2427408400 | 0.72 |
| Prevalence | Jamaica | 1992 | 32.20 | 44.06 | 22.76 | 0.55 | 1395283 | 3376967464 | 2429750605 | 697641.5 | 2429052964 | 0.72 |
| Prevalence | Jordan | 1992 | 17.37 | 24.45 | 12.06 | 0.55 | 2205439 | 3376967464 | 2431956044 | 1102720 | 2430853325 | 0.72 |

| | | | | | | | | | | | | |
|---|---|---|---|---|---|---|---|---|---|---|---|---|
| Prevalence | Armenia | 1992 | 53.44 | 73.95 | 37.61 | 0.55 | 2180135 | 3376967464 | 2434136179 | 1090067 | 2433046112 | 0.72 |
| Prevalence | South Africa | 1992 | 10.10 | 14.25 | 6.97 | 0.55 | 22998760 | 3376967464 | 2457134939 | 11499380 | 2445635559 | 0.72 |
| Prevalence | Albania | 1992 | 64.42 | 86.19 | 46.26 | 0.55 | 2048832 | 3376967464 | 2459183771 | 1024416 | 2458159355 | 0.73 |
| Prevalence | Mauritius | 1992 | 9.44 | 13.78 | 6.14 | 0.55 | 729511 | 3376967464 | 2459913282 | 364755.5 | 2459548527 | 0.73 |
| Prevalence | Libya | 1992 | 19.00 | 26.69 | 13.15 | 0.55 | 2388892 | 3376967464 | 2462302174 | 1194446 | 2461107728 | 0.73 |
| Prevalence | Panama | 1992 | 40.32 | 54.34 | 29.08 | 0.55 | 1507409 | 3376967464 | 2463809583 | 753704.7 | 2463055878 | 0.73 |
| Prevalence | Malaysia | 1992 | 6.36 | 9.56 | 4.03 | 0.56 | 11151630 | 3376967464 | 2474961212 | 5575814 | 2469385397 | 0.73 |
| Prevalence | Saudi Arabia | 1992 | 14.21 | 20.17 | 9.47 | 0.56 | 9752944 | 3376967464 | 2484714156 | 4876472 | 2479837684 | 0.73 |
| Prevalence | Morocco | 2021 | 30.84 | 42.54 | 21.86 | 0.56 | 24647580 | 5105255047 | 1006149263 | 12323790 | 993825472.4 | 0.19 |
| Prevalence | El Salvador | 2021 | 53.94 | 73.23 | 38.48 | 0.56 | 4074428 | 5105255047 | 1010223691 | 2037214 | 1008186477 | 0.20 |
| Prevalence | Ghana | 2021 | 7.37 | 10.18 | 5.11 | 0.56 | 20161140 | 5105255047 | 1030384835 | 10080570 | 1020304263 | 0.20 |
| Prevalence | Turkmenistan | 1992 | 31.64 | 43.63 | 22.14 | 0.57 | 2146841 | 3376967464 | 2486860997 | 1073420 | 2485787576 | 0.74 |
| Prevalence | Cuba | 1992 | 51.88 | 69.32 | 38.38 | 0.57 | 7554381 | 3376967464 | 2494415377 | 3777190 | 2490638187 | 0.74 |
| Prevalence | Dominica | 1992 | 36.91 | 49.93 | 26.47 | 0.57 | 41551.19 | 3376967464 | 2494456928 | 20775.6 | 2494436153 | 0.74 |
| Prevalence | Democratic People's Republic of Korea | 2021 | 13.09 | 19.27 | 8.46 | 0.57 | 18946650 | 5105255047 | 1049331482 | 9473323 | 1039858159 | 0.20 |
| Prevalence | Marshall Islands | 2021 | 10.91 | 15.85 | 7.28 | 0.57 | 36532.79 | 5105255047 | 1049368015 | 18266.39 | 1049349748 | 0.21 |
| Prevalence | India | 2021 | 11.40 | 16.49 | 7.68 | 0.58 | 950604900 | 5105255047 | 1999972931 | 475302500 | 1524670473 | 0.30 |
| Prevalence | Tuvalu | 2021 | 13.39 | 18.99 | 8.94 | 0.58 | 7770.842 | 5105255047 | 1999980701 | 3885.421 | 1999976816 | 0.39 |
| Prevalence | Cook Islands | 1992 | 11.36 | 16.73 | 7.43 | 0.58 | 11553.59 | 3376967464 | 2494468482 | 5776.796 | 2494462705 | 0.74 |
| Prevalence | Congo | 2021 | 7.39 | 10.91 | 4.83 | 0.58 | 3289800 | 5105255047 | 2003270502 | 1644900 | 2001625602 | 0.39 |
| Prevalence | Eswatini | 2021 | 8.77 | 12.36 | 5.77 | 0.59 | 702572.3 | 5105255047 | 2003973074 | 351286.1 | 2003621788 | 0.39 |
| Prevalence | Micronesia (Federated States of) | 2021 | 14.97 | 21.34 | 10.16 | 0.59 | 67172.81 | 5105255047 | 2004040247 | 33586.4 | 2004006661 | 0.39 |
| Prevalence | Uruguay | 1992 | 84.16 | 108.76 | 63.19 | 0.59 | 1978724 | 3376967464 | 2496447206 | 989362.1 | 2495457844 | 0.74 |

| | | | | | | | | | | | | |
|---|---|---|---|---|---|---|---|---|---|---|---|---|
| Prevalence | Seychelles | 1992 | 9.28 | 13.60 | 6.07 | 0.59 | 45508.48 | 3376967464 | 2496492715 | 22754.24 | 2496469961 | 0.74 |
| Prevalence | Samoa | 2021 | 14.51 | 20.88 | 9.67 | 0.59 | 122652 | 5105255047 | 2004162899 | 61325.99 | 2004101573 | 0.39 |
| Prevalence | Azerbaijan | 1992 | 42.81 | 59.25 | 30.44 | 0.59 | 4664389 | 3376967464 | 2501157104 | 2332195 | 2498824909 | 0.74 |
| Prevalence | Bahrain | 1992 | 19.11 | 27.00 | 12.92 | 0.60 | 353670.8 | 3376967464 | 2501510775 | 176835.4 | 2501333939 | 0.74 |
| Prevalence | Kazakhstan | 1992 | 47.09 | 65.11 | 32.78 | 0.60 | 10145610 | 3376967464 | 2511656388 | 5072806 | 2506583581 | 0.74 |
| Prevalence | Venezuela (Bolivarian Republic of) | 2021 | 93.99 | 125.27 | 69.63 | 0.60 | 17426450 | 5105255047 | 2021589354 | 8713227 | 2012876126 | 0.39 |
| Prevalence | Argentina | 1992 | 72.90 | 94.99 | 54.41 | 0.60 | 20617890 | 3376967464 | 2532274273 | 10308940 | 2521965330 | 0.75 |
| Prevalence | Bolivia (Plurinational State of) | 2021 | 54.60 | 75.26 | 38.81 | 0.60 | 7560756 | 5105255047 | 2029150109 | 3780378 | 2025369731 | 0.40 |
| Prevalence | Chile | 1992 | 58.58 | 77.62 | 43.69 | 0.60 | 8721318 | 3376967464 | 2540995591 | 4360659 | 2536634932 | 0.75 |
| Prevalence | Saint Kitts and Nevis | 1992 | 34.86 | 47.58 | 25.17 | 0.60 | 23875.01 | 3376967464 | 2541019466 | 11937.5 | 2541007529 | 0.75 |
| Prevalence | Niue | 1992 | 12.42 | 18.14 | 8.14 | 0.60 | 1295.984 | 3376967464 | 2541020762 | 647.9919 | 2541020114 | 0.75 |
| Prevalence | Kyrgyzstan | 2021 | 60.80 | 82.82 | 43.20 | 0.60 | 4248862 | 5105255047 | 2033398972 | 2124431 | 2031274540 | 0.40 |
| Prevalence | Egypt | 2021 | 31.04 | 43.43 | 21.96 | 0.61 | 64397140 | 5105255047 | 2097796116 | 32198570 | 2065597544 | 0.40 |
| Prevalence | Republic of Moldova | 1992 | 77.98 | 106.75 | 55.92 | 0.61 | 2854706 | 3376967464 | 2543875468 | 1427353 | 2542448115 | 0.75 |
| Prevalence | Belize | 2021 | 47.96 | 65.48 | 34.55 | 0.61 | 283479.5 | 5105255047 | 2098079595 | 141739.7 | 2097937856 | 0.41 |
| Prevalence | North Macedonia | 1992 | 90.89 | 121.33 | 65.53 | 0.61 | 1323446 | 3376967464 | 2545198915 | 661723.2 | 2544537192 | 0.75 |
| Prevalence | Portugal | 1992 | 47.08 | 62.24 | 35.02 | 0.61 | 6780922 | 3376967464 | 2551979837 | 3390461 | 2548589376 | 0.75 |
| Prevalence | Namibia | 2021 | 9.30 | 13.33 | 6.17 | 0.62 | 1504481 | 5105255047 | 2099584076 | 752240.5 | 2098831836 | 0.41 |
| Prevalence | Mongolia | 2021 | 63.04 | 85.16 | 44.23 | 0.62 | 2105152 | 5105255047 | 2101689228 | 1052576 | 2100636652 | 0.41 |
| Prevalence | American Samoa | 1992 | 11.15 | 16.47 | 7.29 | 0.62 | 29749.7 | 3376967464 | 2552009587 | 14874.85 | 2551994712 | 0.76 |
| Prevalence | Dominican Republic | 2021 | 52.87 | 70.86 | 38.49 | 0.62 | 7231298 | 5105255047 | 2108920526 | 3615649 | 2105304877 | 0.41 |
| Prevalence | Syrian Arab Republic | 2021 | 50.41 | 67.17 | 36.80 | 0.62 | 9352995 | 5105255047 | 2118273521 | 4676498 | 2113597023 | 0.41 |
| Prevalence | Antigua and Barbuda | 1992 | 36.72 | 50.22 | 26.55 | 0.62 | 38493.21 | 3376967464 | 2552048080 | 19246.61 | 2552028833 | 0.76 |

| | | | | | | | | | | | | |
|---|---|---|---|---|---|---|---|---|---|---|---|---|
| Prevalence | Nauru | 2021 | 10.82 | 15.66 | 7.23 | 0.63 | 6647.105 | 5105255047 | 2118280168 | 3323.553 | 2118276845 | 0.41 |
| Prevalence | Tonga | 2021 | 15.45 | 22.64 | 10.24 | 0.63 | 60598.31 | 5105255047 | 2118340767 | 30299.16 | 2118310467 | 0.41 |
| Prevalence | Viet Nam | 2021 | 11.98 | 17.60 | 7.70 | 0.63 | 67836850 | 5105255047 | 2186177612 | 33918420 | 2152259189 | 0.42 |
| Prevalence | Romania | 1992 | 262.12 | 348.45 | 188.36 | 0.63 | 15484770 | 3376967464 | 2567532852 | 7742386 | 2559790466 | 0.76 |
| Prevalence | Palestine | 2021 | 18.22 | 25.19 | 12.60 | 0.63 | 3092703 | 5105255047 | 2189270315 | 1546351 | 2187723963 | 0.43 |
| Prevalence | Belarus | 1992 | 98.85 | 134.42 | 71.37 | 0.63 | 6938289 | 3376967464 | 2574471141 | 3469144 | 2571001997 | 0.76 |
| Prevalence | Suriname | 2021 | 66.69 | 90.19 | 48.99 | 0.63 | 384154.9 | 5105255047 | 2189654470 | 192077.4 | 2189462392 | 0.43 |
| Prevalence | Trinidad and Tobago | 1992 | 35.33 | 48.95 | 24.99 | 0.63 | 745716.6 | 3376967464 | 2575216858 | 372858.3 | 2574843999 | 0.76 |
| Prevalence | Gabon | 2021 | 10.35 | 14.82 | 6.88 | 0.63 | 1102224 | 5105255047 | 2190756693 | 551111.8 | 2190205581 | 0.43 |
| Prevalence | Paraguay | 2021 | 77.42 | 104.94 | 56.92 | 0.64 | 4680073 | 5105255047 | 2195436766 | 2340037 | 2193096730 | 0.43 |
| Prevalence | Saint Vincent and the Grenadines | 2021 | 82.42 | 110.60 | 60.00 | 0.64 | 76552.59 | 5105255047 | 2195513319 | 38276.3 | 2195475043 | 0.43 |
| Prevalence | Poland | 1992 | 51.48 | 70.67 | 36.44 | 0.64 | 24967360 | 3376967464 | 2600184220 | 12483680 | 2587700539 | 0.77 |
| Prevalence | Serbia | 1992 | 112.03 | 149.25 | 80.39 | 0.64 | 6574516 | 3376967464 | 2606758736 | 3287258 | 2603471478 | 0.77 |
| Prevalence | Botswana | 2021 | 10.65 | 15.14 | 7.04 | 0.64 | 1590402 | 5105255047 | 2197103721 | 795200.9 | 2196308520 | 0.43 |
| Prevalence | Guyana | 2021 | 74.04 | 95.39 | 55.37 | 0.65 | 501391.8 | 5105255047 | 2197605113 | 250695.9 | 2197354417 | 0.43 |
| Prevalence | Maldives | 2021 | 12.23 | 18.02 | 8.01 | 0.65 | 394937.9 | 5105255047 | 2198000050 | 197468.9 | 2197802582 | 0.43 |
| Prevalence | Bulgaria | 1992 | 139.35 | 188.93 | 100.00 | 0.65 | 5699135 | 3376967464 | 2612457871 | 2849568 | 2609608303 | 0.77 |
| Prevalence | Philippines | 2021 | 7.27 | 10.73 | 4.64 | 0.65 | 73079830 | 5105255047 | 2271079884 | 36539920 | 2234539967 | 0.44 |
| Prevalence | Spain | 1992 | 131.99 | 171.67 | 98.14 | 0.65 | 26113210 | 3376967464 | 2638571077 | 13056600 | 2625514474 | 0.78 |
| Prevalence | Brazil | 2021 | 78.13 | 105.23 | 55.68 | 0.65 | 150405800 | 5105255047 | 2421485716 | 75202920 | 2346282800 | 0.46 |
| Prevalence | Colombia | 2021 | 106.95 | 137.70 | 78.87 | 0.66 | 33609590 | 5105255047 | 2455095302 | 16804790 | 2438290509 | 0.48 |
| Prevalence | Indonesia | 2021 | 9.47 | 13.84 | 6.10 | 0.66 | 194150400 | 5105255047 | 2649245676 | 97075190 | 2552170489 | 0.50 |
| Prevalence | Ukraine | 1992 | 100.54 | 138.14 | 71.57 | 0.66 | 34804310 | 3376967464 | 2673375387 | 17402160 | 2655973232 | 0.79 |
| Prevalence | Equatorial Guinea | 2021 | 6.27 | 9.00 | 4.11 | 0.66 | 893663 | 5105255047 | 2650139339 | 446831.5 | 2649692507 | 0.52 |

| | | | | | | | | | | | | |
|---|---|---|---|---|---|---|---|---|---|---|---|---|
| Prevalence | Algeria | 2021 | 32.74 | 44.80 | 23.22 | 0.66 | 28054400 | 5105255047 | 2678193741 | 14027200 | 2664166540 | 0.52 |
| Prevalence | Hungary | 1992 | 199.27 | 261.42 | 147.34 | 0.66 | 6927328 | 3376967464 | 2680302715 | 3463664 | 2676839051 | 0.79 |
| Prevalence | Qatar | 1992 | 24.73 | 35.25 | 16.91 | 0.66 | 335643.3 | 3376967464 | 2680638358 | 167821.6 | 2680470537 | 0.79 |
| Prevalence | Ecuador | 2021 | 73.32 | 89.20 | 59.42 | 0.66 | 11574520 | 5105255047 | 2689768262 | 5787261 | 2683981001 | 0.53 |
| Prevalence | Peru | 2021 | 61.92 | 82.79 | 44.19 | 0.66 | 23885430 | 5105255047 | 2713653696 | 11942720 | 2701710979 | 0.53 |
| Prevalence | Barbados | 1992 | 53.39 | 72.72 | 39.32 | 0.66 | 162757.9 | 3376967464 | 2680801116 | 81378.93 | 2680719737 | 0.79 |
| Prevalence | Uzbekistan | 2021 | 78.18 | 106.36 | 55.42 | 0.66 | 22329360 | 5105255047 | 2735983057 | 11164680 | 2724818377 | 0.53 |
| Prevalence | Iraq | 2021 | 19.60 | 27.51 | 13.31 | 0.66 | 26081420 | 5105255047 | 2762064476 | 13040710 | 2749023766 | 0.54 |
| Prevalence | Mexico | 2021 | 79.80 | 109.06 | 56.88 | 0.66 | 86646750 | 5105255047 | 2848711229 | 43323380 | 2805387853 | 0.55 |
| Prevalence | Georgia | 1992 | 70.59 | 97.72 | 49.72 | 0.67 | 3570243 | 3376967464 | 2684371360 | 1785122 | 2682586238 | 0.79 |
| Prevalence | Slovakia | 1992 | 68.01 | 88.83 | 51.24 | 0.67 | 3469779 | 3376967464 | 2687841139 | 1734890 | 2686106249 | 0.80 |
| Prevalence | Cyprus | 1992 | 77.77 | 104.88 | 56.59 | 0.67 | 522214.5 | 3376967464 | 2688363353 | 261107.2 | 2688102246 | 0.80 |
| Prevalence | Malta | 1992 | 69.98 | 92.93 | 50.78 | 0.67 | 249227.5 | 3376967464 | 2688612581 | 124613.8 | 2688487967 | 0.80 |
| Prevalence | Guam | 1992 | 12.31 | 17.96 | 8.14 | 0.67 | 93009.67 | 3376967464 | 2688705591 | 46504.84 | 2688659086 | 0.80 |
| Prevalence | Puerto Rico | 1992 | 67.40 | 93.32 | 48.24 | 0.67 | 2339319 | 3376967464 | 2691044910 | 1169660 | 2689875250 | 0.80 |
| Prevalence | Cuba | 2021 | 131.49 | 175.09 | 97.65 | 0.67 | 7721598 | 5105255047 | 2856432827 | 3860799 | 2852572028 | 0.56 |
| Prevalence | Grenada | 2021 | 87.38 | 123.53 | 64.36 | 0.67 | 71465.1 | 5105255047 | 2856504292 | 35732.55 | 2856468560 | 0.56 |
| Prevalence | Kuwait | 1992 | 24.26 | 33.97 | 16.59 | 0.67 | 1142046 | 3376967464 | 2692186956 | 571023.1 | 2691615933 | 0.80 |
| Prevalence | United States Virgin Islands | 1992 | 70.10 | 93.63 | 49.42 | 0.67 | 68428.68 | 3376967464 | 2692255385 | 34214.34 | 2692221171 | 0.80 |
| Prevalence | Saint Lucia | 2021 | 90.15 | 120.69 | 66.44 | 0.67 | 127254.6 | 5105255047 | 2856631547 | 63627.3 | 2856567920 | 0.56 |
| Prevalence | Montenegro | 1992 | 116.96 | 156.80 | 84.55 | 0.67 | 417608.2 | 3376967464 | 2692672993 | 208804.1 | 2692464189 | 0.80 |
| Prevalence | Palau | 1992 | 9.65 | 14.09 | 6.30 | 0.67 | 10631.73 | 3376967464 | 2692683625 | 5315.866 | 2692678309 | 0.80 |
| Prevalence | Fiji | 2021 | 13.16 | 19.28 | 8.54 | 0.68 | 595530.4 | 5105255047 | 2857227077 | 297765.2 | 2856929312 | 0.56 |
| Prevalence | United Arab Emirates | 1992 | 20.55 | 28.52 | 14.07 | 0.68 | 1447269 | 3376967464 | 2694130893 | 723634.3 | 2693407259 | 0.80 |

| | | | | | | | | | | | |
|---|---|---|---|---|---|---|---|---|---|---|---|
| Prevalence | Brunei Darussalam | 1992 | 41.47 | 56.90 | 29.43 | 0.68 | 172733 | 3376967464 | 2694303626 | 86366.52 | 2694217260 | 0.80 |
| Prevalence | Croatia | 1992 | 143.07 | 185.54 | 108.37 | 0.68 | 3329713 | 3376967464 | 2697633339 | 1664857 | 2695968483 | 0.80 |
| Prevalence | South Africa | 2021 | 14.44 | 20.25 | 9.86 | 0.68 | 37983600 | 5105255047 | 2895210682 | 18991800 | 2876218879 | 0.56 |
| Prevalence | Lithuania | 1992 | 90.93 | 118.16 | 68.04 | 0.68 | 2447071 | 3376967464 | 2700080410 | 1223536 | 2698856875 | 0.80 |
| Prevalence | Turkmenistan | 2021 | 73.96 | 100.35 | 52.13 | 0.68 | 3350351 | 5105255047 | 2898561033 | 1675176 | 2896885857 | 0.57 |
| Prevalence | Tunisia | 2021 | 42.38 | 57.44 | 30.57 | 0.68 | 7946659 | 5105255047 | 2906507692 | 3973329 | 2902534362 | 0.57 |
| Prevalence | Thailand | 2021 | 20.08 | 30.06 | 13.06 | 0.68 | 47274310 | 5105255047 | 2953782004 | 23637160 | 2930144848 | 0.57 |
| Prevalence | Jamaica | 2021 | 57.81 | 78.55 | 41.71 | 0.68 | 1946551 | 5105255047 | 2955728554 | 973275.3 | 2954755279 | 0.58 |
| Prevalence | Taiwan (Province of China) | 1992 | 10.54 | 15.72 | 6.73 | 0.68 | 14026120 | 3376967464 | 2714106526 | 7013058 | 2707093468 | 0.80 |
| Prevalence | Tokelau | 2021 | 16.27 | 23.93 | 10.75 | 0.69 | 843.5528 | 5105255047 | 2955729398 | 421.7764 | 2955728976 | 0.58 |
| Prevalence | Greece | 1992 | 99.94 | 132.41 | 73.09 | 0.69 | 7122789 | 3376967464 | 2721229315 | 3561394 | 2717667921 | 0.80 |
| Prevalence | Bahamas | 1992 | 34.09 | 46.45 | 24.46 | 0.69 | 171499.1 | 3376967464 | 2721400814 | 85749.56 | 2721315065 | 0.81 |
| Prevalence | Russian Federation | 1992 | 92.97 | 126.97 | 65.63 | 0.69 | 101681600 | 3376967464 | 2823082430 | 50840810 | 2772241622 | 0.82 |
| Prevalence | Azerbaijan | 2021 | 95.80 | 129.71 | 68.42 | 0.69 | 7440503 | 5105255047 | 2963169901 | 3720252 | 2959449649 | 0.58 |
| Prevalence | Estonia | 1992 | 122.23 | 164.82 | 88.08 | 0.70 | 1018565 | 3376967464 | 2824100995 | 509282.4 | 2823591712 | 0.84 |
| Prevalence | Latvia | 1992 | 143.70 | 192.89 | 103.14 | 0.70 | 1746906 | 3376967464 | 2825847901 | 873453 | 2824974448 | 0.84 |
| Prevalence | Iran (Islamic Republic of) | 2021 | 34.45 | 46.64 | 24.67 | 0.70 | 59167520 | 5105255047 | 3022337423 | 29583760 | 2992753662 | 0.59 |
| Prevalence | Czechia | 1992 | 150.41 | 202.67 | 107.85 | 0.70 | 6884471 | 3376967464 | 2832732372 | 3442236 | 2829290136 | 0.84 |
| Prevalence | Costa Rica | 2021 | 130.17 | 168.18 | 95.68 | 0.70 | 3249833 | 5105255047 | 3025587256 | 1624917 | 3023962339 | 0.59 |
| Prevalence | Sri Lanka | 2021 | 25.45 | 34.82 | 17.95 | 0.70 | 14714740 | 5105255047 | 3040301997 | 7357370 | 3032944626 | 0.59 |
| Prevalence | Armenia | 2021 | 124.12 | 166.18 | 90.87 | 0.70 | 2004496 | 5105255047 | 3042306493 | 1002248 | 3041304245 | 0.60 |
| Prevalence | Singapore | 1992 | 69.20 | 92.88 | 50.90 | 0.70 | 2369939 | 3376967464 | 2835102310 | 1184969 | 2833917341 | 0.84 |
| Prevalence | Bermuda | 1992 | 128.92 | 173.07 | 94.22 | 0.70 | 42685.4 | 3376967464 | 2835144996 | 21342.7 | 2835123653 | 0.84 |
| Prevalence | Albania | 2021 | 184.66 | 247.65 | 135.53 | 0.71 | 1808384 | 5105255047 | 3044114877 | 904192 | 3043210685 | 0.60 |

| | | | | | | | | | | | | |
|---|---|---|---|---|---|---|---|---|---|---|---|---|
| Prevalence | Panama | 2021 | 74.40 | 99.73 | 54.23 | 0.71 | 2749507 | 5105255047 | 3046864384 | 1374753 | 3045489630 | 0.60 |
| Prevalence | Republic of Korea | 1992 | 59.85 | 81.90 | 42.15 | 0.71 | 31435440 | 3376967464 | 2866580440 | 15717720 | 2850862718 | 0.84 |
| Prevalence | Turkey | 2021 | 41.16 | 56.35 | 29.09 | 0.71 | 56921960 | 5105255047 | 3103786340 | 28460980 | 3075325362 | 0.60 |
| Prevalence | Northern Mariana Islands | 1992 | 14.24 | 20.38 | 9.85 | 0.72 | 33889.48 | 3376967464 | 2866614329 | 16944.74 | 2866597384 | 0.85 |
| Prevalence | Italy | 1992 | 149.46 | 202.70 | 107.30 | 0.72 | 38983200 | 3376967464 | 2905597533 | 19491600 | 2886105931 | 0.85 |
| Prevalence | Israel | 1992 | 45.73 | 61.71 | 33.34 | 0.72 | 3155772 | 3376967464 | 2908753305 | 1577886 | 2907175419 | 0.86 |
| Prevalence | Mauritius | 2021 | 23.78 | 33.88 | 15.80 | 0.72 | 900058.5 | 5105255047 | 3104686398 | 450029.2 | 3104236369 | 0.61 |
| Prevalence | Uruguay | 2021 | 136.03 | 179.73 | 101.58 | 0.72 | 2214747 | 5105255047 | 3106901145 | 1107373 | 3105793772 | 0.61 |
| Prevalence | China | 2021 | 22.23 | 31.77 | 14.99 | 0.72 | 967106700 | 5105255047 | 4074007884 | 483553400 | 3590454514 | 0.70 |
| Prevalence | Bosnia and Herzegovina | 2021 | 245.09 | 327.82 | 178.45 | 0.72 | 2206873 | 5105255047 | 4076214757 | 1103437 | 4075111320 | 0.80 |
| Prevalence | Argentina | 2021 | 100.31 | 131.10 | 73.20 | 0.72 | 30052210 | 5105255047 | 4106266964 | 15026100 | 4091240861 | 0.80 |
| Prevalence | American Samoa | 2021 | 23.78 | 34.05 | 16.21 | 0.72 | 31908.17 | 5105255047 | 4106298872 | 15954.09 | 4106282918 | 0.80 |
| Prevalence | Kazakhstan | 2021 | 93.96 | 128.09 | 66.29 | 0.73 | 12126520 | 5105255047 | 4118425393 | 6063260 | 4112362132 | 0.81 |
| Prevalence | Jordan | 2021 | 28.42 | 39.69 | 19.74 | 0.73 | 8180007 | 5105255047 | 4126605399 | 4090003 | 4122515396 | 0.81 |
| Prevalence | Libya | 2021 | 31.21 | 42.60 | 22.70 | 0.73 | 5029432 | 5105255047 | 4131634831 | 2514716 | 4129120115 | 0.81 |
| Prevalence | Niue | 2021 | 24.29 | 35.23 | 16.25 | 0.73 | 1099.18 | 5105255047 | 4131635930 | 549.5901 | 4131635381 | 0.81 |
| Prevalence | Greenland | 1992 | 62.59 | 84.25 | 45.03 | 0.73 | 38395.59 | 3376967464 | 2908791700 | 19197.79 | 2908772503 | 0.86 |
| Prevalence | Seychelles | 2021 | 21.85 | 31.22 | 14.74 | 0.73 | 72957.22 | 5105255047 | 4131708887 | 36478.61 | 4131672409 | 0.81 |
| Prevalence | Ireland | 1992 | 99.19 | 133.50 | 72.64 | 0.73 | 2251651 | 3376967464 | 2911043351 | 1125825 | 2909917526 | 0.86 |
| Prevalence | Republic of Moldova | 2021 | 159.08 | 216.26 | 114.59 | 0.73 | 2516382 | 5105255047 | 4134225269 | 1258191 | 4132967078 | 0.81 |
| Prevalence | Georgia | 2021 | 159.03 | 212.68 | 116.51 | 0.73 | 2299321 | 5105255047 | 4136524590 | 1149660 | 4135374929 | 0.81 |
| Prevalence | Australia | 1992 | 67.67 | 91.26 | 48.61 | 0.73 | 11484530 | 3376967464 | 2922527885 | 5742267 | 2916785618 | 0.86 |
| Prevalence | Slovenia | 1992 | 200.50 | 272.58 | 146.63 | 0.74 | 1357830 | 3376967464 | 2923885715 | 678914.9 | 2923206800 | 0.87 |
| Prevalence | Malaysia | 2021 | 13.53 | 19.90 | 8.77 | 0.74 | 21856660 | 5105255047 | 4158381246 | 10928330 | 4147452918 | 0.81 |

| | | | | | | | | | | | | |
|---|---|---|---|---|---|---|---|---|---|---|---|---|
| Prevalence | France | 1992 | 113.68 | 147.99 | 84.68 | 0.74 | 38242170 | 3376967464 | 2962127885 | 19121090 | 2943006800 | 0.87 |
| Prevalence | Portugal | 2021 | 95.12 | 125.45 | 69.89 | 0.74 | 6825510 | 5105255047 | 4165206755 | 3412755 | 4161794000 | 0.82 |
| Prevalence | Lebanon | 2021 | 40.72 | 54.84 | 30.11 | 0.74 | 3716347 | 5105255047 | 4168923102 | 1858174 | 4167064929 | 0.82 |
| Prevalence | Barbados | 2021 | 118.39 | 162.36 | 85.61 | 0.75 | 202783.5 | 5105255047 | 4169125886 | 101391.7 | 4169024494 | 0.82 |
| Prevalence | Dominica | 2021 | 89.97 | 122.00 | 65.67 | 0.75 | 46130.66 | 5105255047 | 4169172016 | 23065.33 | 4169148951 | 0.82 |
| Prevalence | Belgium | 1992 | 126.30 | 168.72 | 92.28 | 0.75 | 6685776 | 3376967464 | 2968813661 | 3342888 | 2965470773 | 0.88 |
| Prevalence | Antigua and Barbuda | 2021 | 78.57 | 106.76 | 56.70 | 0.75 | 63578.22 | 5105255047 | 4169235595 | 31789.11 | 4169203806 | 0.82 |
| Prevalence | North Macedonia | 2021 | 191.98 | 254.40 | 141.08 | 0.75 | 1541196 | 5105255047 | 4170776791 | 770598.2 | 4170006193 | 0.82 |
| Prevalence | Bahrain | 2021 | 35.88 | 49.84 | 25.06 | 0.75 | 1178377 | 5105255047 | 4171955168 | 589188.4 | 4171365980 | 0.82 |
| Prevalence | Palau | 2021 | 27.45 | 39.93 | 18.52 | 0.75 | 13185.18 | 5105255047 | 4171968353 | 6592.589 | 4171961761 | 0.82 |
| Prevalence | Austria | 1992 | 238.18 | 287.87 | 196.15 | 0.75 | 5270737 | 3376967464 | 2974084397 | 2635368 | 2971449029 | 0.88 |
| Prevalence | United Kingdom | 1992 | 119.32 | 157.18 | 88.65 | 0.75 | 37397090 | 3376967464 | 3011481485 | 18698540 | 2992782941 | 0.89 |
| Prevalence | Saint Kitts and Nevis | 2021 | 88.32 | 117.41 | 64.35 | 0.75 | 43420.68 | 5105255047 | 4172011774 | 21710.34 | 4171990064 | 0.82 |
| Prevalence | New Zealand | 1992 | 78.69 | 105.91 | 57.30 | 0.76 | 2285123 | 3376967464 | 3013766608 | 1142561 | 3012624047 | 0.89 |
| Prevalence | Ukraine | 2021 | 155.88 | 212.44 | 109.53 | 0.76 | 29287980 | 5105255047 | 4201299752 | 14643990 | 4186655763 | 0.82 |
| Prevalence | Finland | 1992 | 185.96 | 247.71 | 135.11 | 0.76 | 3388185 | 3376967464 | 3017154793 | 1694093 | 3015460701 | 0.89 |
| Prevalence | Andorra | 1992 | 80.54 | 108.90 | 57.95 | 0.77 | 45683.08 | 3376967464 | 3017200476 | 22841.54 | 3017177635 | 0.89 |
| Prevalence | Bulgaria | 2021 | 193.27 | 251.61 | 141.87 | 0.77 | 4337506 | 5105255047 | 4205637258 | 2168753 | 4203468505 | 0.82 |
| Prevalence | Romania | 2021 | 552.30 | 737.42 | 386.48 | 0.77 | 12142540 | 5105255047 | 4217779801 | 6071271 | 4211708529 | 0.82 |
| Prevalence | Trinidad and Tobago | 2021 | 84.23 | 115.04 | 60.21 | 0.77 | 942563.1 | 5105255047 | 4218722364 | 471281.5 | 4218251082 | 0.83 |
| Prevalence | Spain | 2021 | 224.73 | 295.52 | 163.42 | 0.77 | 29873650 | 5105255047 | 4248596015 | 14936830 | 4233659190 | 0.83 |
| Prevalence | United States of America | 1992 | 130.40 | 176.17 | 94.78 | 0.77 | 169626400 | 3376967464 | 3186826882 | 84813200 | 3102013679 | 0.92 |
| Prevalence | Chile | 2021 | 127.25 | 168.87 | 92.15 | 0.77 | 12819950 | 5105255047 | 4261415966 | 6409975 | 4255005991 | 0.83 |
| Prevalence | Northern Mariana Islands | 2021 | 37.80 | 52.40 | 26.76 | 0.77 | 33644 | 5105255047 | 4261449610 | 16822 | 4261432788 | 0.83 |

| | | | | | | | | | | | | |
|---|---|---|---|---|---|---|---|---|---|---|---|---|
| Prevalence | Iceland | 1992 | 135.09 | 180.49 | 99.01 | 0.77 | 167194.9 | 3376967464 | 3186994077 | 83597.46 | 3186910480 | 0.94 |
| Prevalence | Oman | 2021 | 24.46 | 33.91 | 17.01 | 0.77 | 3365550 | 5105255047 | 4264815160 | 1682775 | 4263132385 | 0.84 |
| Prevalence | Cook Islands | 2021 | 26.94 | 39.01 | 18.14 | 0.78 | 11633.24 | 5105255047 | 4264826793 | 5816.619 | 4264820977 | 0.84 |
| Prevalence | Belarus | 2021 | 184.49 | 248.94 | 131.77 | 0.78 | 6254502 | 5105255047 | 4271081295 | 3127251 | 4267954044 | 0.84 |
| Prevalence | Canada | 1992 | 87.52 | 115.20 | 63.77 | 0.79 | 18901940 | 3376967464 | 3205896013 | 9450968 | 3196445045 | 0.95 |
| Prevalence | Luxembourg | 1992 | 170.43 | 222.32 | 125.77 | 0.79 | 268190.4 | 3376967464 | 3206164203 | 134095.2 | 3206030108 | 0.95 |
| Prevalence | Hungary | 2021 | 388.48 | 504.92 | 285.90 | 0.79 | 6199148 | 5105255047 | 4277280443 | 3099574 | 4274180869 | 0.84 |
| Prevalence | Greece | 2021 | 158.87 | 203.82 | 121.97 | 0.79 | 6469340 | 5105255047 | 4283749783 | 3234670 | 4280515113 | 0.84 |
| Prevalence | Serbia | 2021 | 229.24 | 304.48 | 167.24 | 0.79 | 5934126 | 5105255047 | 4289683909 | 2967063 | 4286716846 | 0.84 |
| Prevalence | Sweden | 1992 | 143.96 | 196.17 | 104.13 | 0.80 | 5553552 | 3376967464 | 3211717756 | 2776776 | 3208940980 | 0.95 |
| Prevalence | Montenegro | 2021 | 214.03 | 283.70 | 155.57 | 0.80 | 412912.2 | 5105255047 | 4290096822 | 206456.1 | 4289890365 | 0.84 |
| Prevalence | Croatia | 2021 | 409.84 | 531.73 | 300.68 | 0.80 | 2715472 | 5105255047 | 4292812293 | 1357736 | 4291454558 | 0.84 |
| Prevalence | Japan | 1992 | 145.39 | 195.08 | 105.30 | 0.80 | 88370150 | 3376967464 | 3300087906 | 44185070 | 3255902831 | 0.96 |
| Prevalence | Malta | 2021 | 135.31 | 177.19 | 100.20 | 0.80 | 278137.3 | 5105255047 | 4293090431 | 139068.6 | 4292951362 | 0.84 |
| Prevalence | Netherlands | 1992 | 48.83 | 64.35 | 35.88 | 0.80 | 10396300 | 3376967464 | 3310484202 | 5198148 | 3305286054 | 0.98 |
| Prevalence | Guam | 2021 | 30.76 | 43.20 | 21.61 | 0.80 | 103477.4 | 5105255047 | 4293193908 | 51738.72 | 4293142170 | 0.84 |
| Prevalence | Bahamas | 2021 | 76.05 | 100.75 | 55.65 | 0.81 | 274938.4 | 5105255047 | 4293468847 | 137469.2 | 4293331377 | 0.84 |
| Prevalence | Norway | 1992 | 98.70 | 133.47 | 71.60 | 0.81 | 2768970 | 3376967464 | 3313253172 | 1384485 | 3311868687 | 0.98 |
| Prevalence | Italy | 2021 | 219.53 | 278.73 | 170.04 | 0.81 | 38186670 | 5105255047 | 4331655515 | 19093330 | 4312562181 | 0.84 |
| Prevalence | Denmark | 1992 | 111.67 | 148.29 | 81.08 | 0.81 | 3484450 | 3376967464 | 3316737622 | 1742225 | 3314995397 | 0.98 |
| Prevalence | Russian Federation | 2021 | 213.65 | 286.50 | 153.12 | 0.81 | 96028800 | 5105255047 | 4427684315 | 48014400 | 4379669915 | 0.86 |
| Prevalence | Israel | 2021 | 68.15 | 90.86 | 49.98 | 0.81 | 5769267 | 5105255047 | 4433453582 | 2884633 | 4430568949 | 0.87 |
| Prevalence | Brunei Darussalam | 2021 | 90.73 | 124.21 | 65.45 | 0.81 | 332019.1 | 5105255047 | 4433785601 | 166009.6 | 4433619592 | 0.87 |
| Prevalence | Slovakia | 2021 | 189.35 | 252.65 | 143.62 | 0.81 | 3635982 | 5105255047 | 4437421583 | 1817991 | 4435603592 | 0.87 |

| | | | | | | | | | | | | |
|---|---|---|---|---|---|---|---|---|---|---|---|---|
| Prevalence | Poland | 2021 | 81.37 | 105.57 | 60.35 | 0.81 | 25179790 | 5105255047 | 4462601375 | 12589900 | 4450011479 | 0.87 |
| Prevalence | Saudi Arabia | 2021 | 28.10 | 39.42 | 19.21 | 0.82 | 29117100 | 5105255047 | 4491718473 | 14558550 | 4477159924 | 0.88 |
| Prevalence | Bermuda | 2021 | 315.30 | 429.75 | 227.95 | 0.82 | 41969.77 | 5105255047 | 4491760443 | 20984.88 | 4491739458 | 0.88 |
| Prevalence | United States Virgin Islands | 2021 | 190.88 | 250.58 | 138.24 | 0.82 | 53866.94 | 5105255047 | 4491814310 | 26933.47 | 4491787376 | 0.88 |
| Prevalence | San Marino | 1992 | 103.29 | 141.73 | 74.13 | 0.82 | 16789.73 | 3376967464 | 3316754411 | 8394.865 | 3316746017 | 0.98 |
| Prevalence | Puerto Rico | 2021 | 142.75 | 195.56 | 102.51 | 0.83 | 2124836 | 5105255047 | 4493939146 | 1062418 | 4492876728 | 0.88 |
| Prevalence | Greenland | 2021 | 156.19 | 206.25 | 114.59 | 0.83 | 39064.81 | 5105255047 | 4493978210 | 19532.41 | 4493958678 | 0.88 |
| Prevalence | Germany | 1992 | 164.66 | 216.28 | 122.99 | 0.83 | 55437780 | 3376967464 | 3372192189 | 27718890 | 3344473300 | 0.99 |
| Prevalence | Czechia | 2021 | 378.17 | 510.76 | 273.45 | 0.83 | 6708178 | 5105255047 | 4500686388 | 3354089 | 4497332299 | 0.88 |
| Prevalence | Latvia | 2021 | 312.99 | 424.35 | 223.48 | 0.83 | 1180840 | 5105255047 | 4501867228 | 590420 | 4501276808 | 0.88 |
| Prevalence | Cyprus | 2021 | 116.62 | 156.04 | 86.13 | 0.84 | 940775.7 | 5105255047 | 4502808004 | 470387.8 | 4502337616 | 0.88 |
| Prevalence | France | 2021 | 159.88 | 220.85 | 113.16 | 0.84 | 41021790 | 5105255047 | 4543829797 | 20510900 | 4523318900 | 0.89 |
| Prevalence | Slovenia | 2021 | 464.81 | 641.61 | 333.69 | 0.84 | 1320405 | 5105255047 | 4545150201 | 660202.5 | 4544489999 | 0.89 |
| Prevalence | Australia | 2021 | 117.83 | 159.28 | 85.07 | 0.84 | 16656050 | 5105255047 | 4561806249 | 8328024 | 4553478225 | 0.89 |
| Prevalence | Estonia | 2021 | 356.97 | 497.01 | 255.84 | 0.84 | 825205.3 | 5105255047 | 4562631454 | 412602.6 | 4562218851 | 0.89 |
| Prevalence | Kuwait | 2021 | 42.66 | 59.18 | 29.57 | 0.85 | 3633714 | 5105255047 | 4566265168 | 1816857 | 4564448311 | 0.89 |
| Prevalence | Qatar | 2021 | 32.42 | 46.37 | 21.98 | 0.85 | 2446107 | 5105255047 | 4568711276 | 1223054 | 4567488222 | 0.89 |
| Prevalence | United Arab Emirates | 2021 | 48.84 | 68.54 | 34.62 | 0.85 | 8129433 | 5105255047 | 4576840709 | 4064717 | 4572775992 | 0.90 |
| Prevalence | New Zealand | 2021 | 124.61 | 163.56 | 93.02 | 0.85 | 3378026 | 5105255047 | 4580218735 | 1689013 | 4578529722 | 0.90 |
| Prevalence | Monaco | 1992 | 126.72 | 172.92 | 91.39 | 0.85 | 19942.64 | 3376967464 | 3372212132 | 9971.318 | 3372202160 | 1.00 |
| Prevalence | Belgium | 2021 | 188.73 | 257.04 | 137.12 | 0.85 | 7314275 | 5105255047 | 4587533010 | 3657137 | 4583875872 | 0.90 |
| Prevalence | Austria | 2021 | 382.51 | 528.16 | 272.35 | 0.85 | 5970418 | 5105255047 | 4593503427 | 2985209 | 4590518219 | 0.90 |
| Prevalence | Singapore | 2021 | 135.97 | 184.38 | 99.61 | 0.86 | 4146887 | 5105255047 | 4597650315 | 2073444 | 4595576871 | 0.90 |
| Prevalence | Lithuania | 2021 | 244.28 | 331.10 | 177.24 | 0.86 | 1763517 | 5105255047 | 4599413832 | 881758.6 | 4598532074 | 0.90 |

| | | | | | | | | | | | |
|---|---|---|---|---|---|---|---|---|---|---|---|
| Prevalence | United Kingdom | 2021 | 145.28 | 191.05 | 107.59 | 0.86 | 43558640 | 5105255047 | 4642972474 | 21779320 | 4621193153 | 0.91 |
| Prevalence | Finland | 2021 | 280.27 | 381.27 | 202.91 | 0.86 | 3397926 | 5105255047 | 4646370400 | 1698963 | 4644671437 | 0.91 |
| Prevalence | United States of America | 2021 | 185.87 | 235.69 | 142.13 | 0.86 | 216097500 | 5105255047 | 4862467891 | 108048700 | 4754419146 | 0.93 |
| Prevalence | Switzerland | 1992 | 167.98 | 219.86 | 125.95 | 0.87 | 4755332 | 3376967464 | 3376967464 | 2377666 | 3374589798 | 1.00 |
| Prevalence | Andorra | 2021 | 167.00 | 228.30 | 121.04 | 0.87 | 61742.59 | 5105255047 | 4862529634 | 30871.3 | 4862498763 | 0.95 |
| Prevalence | Japan | 2021 | 154.25 | 203.95 | 114.54 | 0.87 | 75404150 | 5105255047 | 4937933788 | 37702080 | 4900231711 | 0.96 |
| Prevalence | Canada | 2021 | 175.86 | 233.63 | 127.66 | 0.87 | 24262340 | 5105255047 | 4962196124 | 12131170 | 4950064956 | 0.97 |
| Prevalence | Ireland | 2021 | 161.95 | 217.18 | 116.55 | 0.87 | 3192765 | 5105255047 | 4965388890 | 1596383 | 4963792507 | 0.97 |
| Prevalence | Taiwan (Province of China) | 2021 | 29.01 | 43.11 | 19.38 | 0.87 | 16681750 | 5105255047 | 4982070642 | 8340876 | 4973729766 | 0.97 |
| Prevalence | Iceland | 2021 | 249.89 | 339.29 | 179.57 | 0.88 | 227618.6 | 5105255047 | 4982298260 | 113809.3 | 4982184451 | 0.98 |
| Prevalence | Luxembourg | 2021 | 230.29 | 310.11 | 164.23 | 0.88 | 447039.5 | 5105255047 | 4982745300 | 223519.8 | 4982521780 | 0.98 |
| Prevalence | Republic of Korea | 2021 | 148.78 | 203.41 | 106.77 | 0.89 | 36999740 | 5105255047 | 5019745039 | 18499870 | 5001245169 | 0.98 |
| Prevalence | Sweden | 2021 | 220.39 | 293.67 | 158.00 | 0.89 | 6417083 | 5105255047 | 5026162122 | 3208541 | 5022953580 | 0.98 |
| Prevalence | San Marino | 2021 | 168.56 | 227.77 | 122.34 | 0.89 | 21290.2 | 5105255047 | 5026183412 | 10645.1 | 5026172767 | 0.98 |
| Prevalence | Netherlands | 2021 | 76.15 | 101.52 | 54.79 | 0.89 | 11069920 | 5105255047 | 5037253336 | 5534962 | 5031718374 | 0.99 |
| Prevalence | Denmark | 2021 | 209.52 | 276.54 | 153.58 | 0.90 | 3719018 | 5105255047 | 5040972354 | 1859509 | 5039112845 | 0.99 |
| Prevalence | Germany | 2021 | 268.54 | 356.26 | 195.98 | 0.90 | 54851780 | 5105255047 | 5095824131 | 27425890 | 5068398243 | 0.99 |
| Prevalence | Monaco | 2021 | 188.75 | 258.94 | 138.25 | 0.91 | 23218.3 | 5105255047 | 5095847350 | 11609.15 | 5095835740 | 1.00 |
| Prevalence | Norway | 2021 | 153.07 | 205.96 | 110.22 | 0.92 | 3522494 | 5105255047 | 5099369844 | 1761247 | 5097608597 | 1.00 |
| Prevalence | Switzerland | 2021 | 229.21 | 311.73 | 164.43 | 0.93 | 5885204 | 5105255047 | 5105255047 | 2942602 | 5102312446 | 1.00 |

**Abbreviation:** SDI: Sociodemographic index; DALYs: Disability-Adjusted Life Years.
