# Supplementary material for: Trends in the global burden of aortic valve calcification disease in the working-age population from 1992 to 2021
Source: Front Cardiovasc Med. 2025 Aug 12;12:1544273. doi: 10.3389/fcvm.2025.1544273 (PMC12379075; doi:10.3389/fcvm.2025.1544273)
Supplement: Supplementary file 3 [file Datasheet3.zip › Supplementary Table 1.PDF]

Supplementary

Table S1. The disease burden of ASPR, ASIR, ASDALYs, and ASDR in the working-age range of 204 countries and regions worldwide from 1992 to 2021

Measure	Location	Value	Upper	Lower
Deaths	Chile	0.478508524	0.584326082	0.387858914
Deaths	Chile	0.118068609	0.146447859	0.093264499
Deaths	Chile	0.291262782	0.346610776	0.243754853
Deaths	Nepal	0.295684703	0.518910999	0.149502795
Deaths	Nepal	0.217904386	0.425657575	0.090521823
Deaths	Nepal	0.253870047	0.413272807	0.141457412
Deaths	Armenia	0.127498876	0.149652871	0.104807826
Deaths	Armenia	0.058553103	0.069760971	0.047114451
Deaths	Armenia	0.090753327	0.106756107	0.074486966
Deaths	Thailand	0.217329802	0.384097777	0.113034893
Deaths	Thailand	0.11750109	0.211158023	0.051285274
Deaths	Thailand	0.165583929	0.266954661	0.097472517
Deaths	Zimbabwe	0.539445699	1.01381033	0.249765849
Deaths	Zimbabwe	0.263054809	0.583808928	0.098990799
Deaths	Zimbabwe	0.388975752	0.682949337	0.20219457
Deaths	Bermuda	2.434972911	3.145794103	1.853364843
Deaths	Bermuda	0.706815701	0.945975995	0.52539258
Deaths	Bermuda	1.558105074	1.96816001	1.217968086
Deaths	Poland	1.126738661	1.262147045	0.996159661
Deaths	Poland	0.362677884	0.411531582	0.31266294

Deaths	Poland	0.737167654	0.815302011	0.6661023
Deaths	Morocco	0.440557229	0.874047344	0.132526427
Deaths	Morocco	0.233053505	0.630698287	0.040621722
Deaths	Morocco	0.336800228	0.640885416	0.106847374
Deaths	Greece	0.646583866	0.765949913	0.538184006
Deaths	Greece	0.304376885	0.372052693	0.244512028
Deaths	Greece	0.470748488	0.53942722	0.404672435
Deaths	Montenegro	0.234667693	0.363604017	0.143462159
Deaths	Montenegro	0.096386203	0.186163991	0.049515193
Deaths	Montenegro	0.164319591	0.245434603	0.108387581
Deaths	Kenya	0.31805968	0.439849309	0.217442463
Deaths	Kenya	0.192337407	0.338003376	0.105413316
Deaths	Kenya	0.254540423	0.350605248	0.182563306
Deaths	Nigeria	0.298809629	0.628714289	0.145211871
Deaths	Nigeria	0.177211117	0.311682116	0.072391737
Deaths	Nigeria	0.232477661	0.428680956	0.122882594
Deaths	Mauritius	0.740933578	0.90393689	0.594710758
Deaths	Mauritius	0.388547644	0.480535668	0.306560881
Deaths	Mauritius	0.56307039	0.659984222	0.469931405
Deaths	Timor-Leste	0.033526544	0.071378754	0.008315287
Deaths	Timor-Leste	0.029545249	0.128069565	0.004885058
Deaths	Timor-Leste	0.031574755	0.078859859	0.009599456
Deaths	Jamaica	0.128620408	0.186047632	0.083714569
Deaths	Jamaica	0.076818816	0.110115529	0.051983939

Deaths	Jamaica	0.102614536	0.14155745	0.071600114
Deaths	Ireland	0.479701611	0.591311346	0.384181895
Deaths	Ireland	0.207576785	0.26283323	0.159099217
Deaths	Ireland	0.341779411	0.407281998	0.284213121
Deaths	Cook Islands	0.086318918	0.143367044	0.048292981
Deaths	Cook Islands	0.310995425	0.687501769	0.138826676
Deaths	Cook Islands	0.206429133	0.413968743	0.104951051
Deaths	Nicaragua	0.208449234	0.326901826	0.12510795
Deaths	Nicaragua	0.120305201	0.196698272	0.064566172
Deaths	Nicaragua	0.161265553	0.23023145	0.108639299
Deaths	Eswatini	0.56385206	1.077203483	0.23897504
Deaths	Eswatini	0.208702384	0.46730235	0.06744414
Deaths	Eswatini	0.373821542	0.67044455	0.1755658
Deaths	South Sudan	0.381818785	0.771177555	0.137733464
Deaths	South Sudan	0.202524186	0.442686391	0.0717094
Deaths	South Sudan	0.293051771	0.53050682	0.133179188
Deaths	Madagascar	0.759103842	1.36372147	0.343696161
Deaths	Madagascar	0.442918105	0.924451085	0.169015234
Deaths	Madagascar	0.59720277	0.986104308	0.316191502
Deaths	Sri Lanka	0.185913219	0.324565983	0.093039063
Deaths	Sri Lanka	0.080495142	0.17485994	0.037368235
Deaths	Sri Lanka	0.13062134	0.215766473	0.071518205
Deaths	Iceland	0.740806458	0.911213812	0.589140582
Deaths	Iceland	0.415622054	0.515663982	0.32922059

Deaths	Iceland	0.580774199	0.691902651	0.477842191
Deaths	Benin	0.290426979	0.566882753	0.118461083
Deaths	Benin	0.14875329	0.32178599	0.052780622
Deaths	Benin	0.217464039	0.384420331	0.103129519
Deaths	Venezuela (Bolivarian Republic of)	0.704313492	1.013181218	0.477326974
Deaths	Venezuela (Bolivarian Republic of)	0.353321866	0.508487264	0.237194835
Deaths	Venezuela (Bolivarian Republic of)	0.522065815	0.715584423	0.370178099
Deaths	Saint Lucia	0.524542038	0.689494987	0.3881649
Deaths	Saint Lucia	0.200401695	0.267814522	0.144543614
Deaths	Saint Lucia	0.362518095	0.463889156	0.275813738
Deaths	Saint Vincent and the Grenadines	0.702913935	0.893778454	0.536484514
Deaths	Saint Vincent and the Grenadines	0.285204393	0.368773136	0.215746993
Deaths	Saint Vincent and the Grenadines	0.501743464	0.616390997	0.40136407
Deaths	Palestine	0.227659697	0.366172575	0.133637835
Deaths	Palestine	0.081159645	0.160200238	0.035941334
Deaths	Palestine	0.156046925	0.236692154	0.099493163
Deaths	Sao Tome and Principe	0.33700577	0.816593906	0.139885486
Deaths	Sao Tome and Principe	0.277195146	0.582337844	0.107126821
Deaths	Sao Tome and Principe	0.307441182	0.609174652	0.152407852
Deaths	Georgia	0.43862194	0.561964345	0.332141421
Deaths	Georgia	0.296871166	0.362159738	0.238756077
Deaths	Georgia	0.36513404	0.451622439	0.28897628
Deaths	Solomon Islands	0.327724387	0.599210715	0.161341603
Deaths	Solomon Islands	0.307423575	0.647987386	0.130237093

Deaths	Solomon Islands	0.319099847	0.572381138	0.163353391
Deaths	Republic of Korea	0.120775065	0.180199688	0.076392863
Deaths	Republic of Korea	0.074482946	0.127356451	0.039434192
Deaths	Republic of Korea	0.097978357	0.137425844	0.06785872
Deaths	Oman	0.166292666	0.279601883	0.087614416
Deaths	Oman	0.013353538	0.033106353	0.004319331
Deaths	Oman	0.11086095	0.184043792	0.060057912
Deaths	Canada	0.4356807	0.529455079	0.353267616
Deaths	Canada	0.201986304	0.248244379	0.160464837
Deaths	Canada	0.31701383	0.37044069	0.269719382
Deaths	Malawi	0.589196443	1.099345559	0.281114516
Deaths	Malawi	0.214406439	0.451759039	0.077051742
Deaths	Malawi	0.394250504	0.662515432	0.2086888
Deaths	Uruguay	1.491286019	1.797313896	1.221323973
Deaths	Uruguay	0.603719178	0.743369353	0.479344863
Deaths	Uruguay	1.026215274	1.188530215	0.879425525
Deaths	Angola	0.586133255	1.067825711	0.280716263
Deaths	Angola	0.323928846	0.664143177	0.12209415
Deaths	Angola	0.446149479	0.750799331	0.241046061
Deaths	Senegal	0.339131788	0.666521701	0.137396208
Deaths	Senegal	0.241917613	0.505213441	0.0906974
Deaths	Senegal	0.28858092	0.515893954	0.138545383
Deaths	Guam	0.909165525	1.3468511	0.59824066
Deaths	Guam	0.693045214	1.04294295	0.433521568

Deaths	Guam	0.803366137	1.114110532	0.568061345
Deaths	Azerbaijan	0.01372088	0.024945986	0.007253939
Deaths	Azerbaijan	0.029713418	0.047994962	0.016607557
Deaths	Azerbaijan	0.022066919	0.034509427	0.013797654
Deaths	United States Virgin Islands	0.757355602	1.410879561	0.356490431
Deaths	United States Virgin Islands	0.32619646	0.710730865	0.124024247
Deaths	United States Virgin Islands	0.536993264	0.933026166	0.288286655
Deaths	Saudi Arabia	0.286658994	0.52932408	0.137613405
Deaths	Saudi Arabia	0.317014736	0.660674436	0.126823027
Deaths	Saudi Arabia	0.298717766	0.496409097	0.166243484
Deaths	Brunei Darussalam	0.460656158	0.721748618	0.276834633
Deaths	Brunei Darussalam	0.315801137	0.583366766	0.14952898
Deaths	Brunei Darussalam	0.39163637	0.577780515	0.254695049
Deaths	Pakistan	0.373185854	0.604187729	0.21314983
Deaths	Pakistan	0.237714436	0.464575182	0.102770584
Deaths	Pakistan	0.307456975	0.471815687	0.193033736
Deaths	Panama	0.514393638	0.690725023	0.365137392
Deaths	Panama	0.268626085	0.356100502	0.197279663
Deaths	Panama	0.390951792	0.501453151	0.290672273
Deaths	Cambodia	0.031491577	0.060581206	0.010696698
Deaths	Cambodia	0.024347844	0.102103175	0.004048456
Deaths	Cambodia	0.02753135	0.07079102	0.00979966
Deaths	Italy	0.528951291	0.572939414	0.487418201
Deaths	Italy	0.229144208	0.249051877	0.209260308

Deaths	Italy	0.376892779	0.404519308	0.350609392
Deaths	Kazakhstan	0.103219858	0.134376044	0.079039619
Deaths	Kazakhstan	0.066470772	0.082175702	0.051606228
Deaths	Kazakhstan	0.083888682	0.105718454	0.066058287
Deaths	Singapore	0.097703706	0.117967182	0.079952159
Deaths	Singapore	0.05255635	0.063824263	0.042504767
Deaths	Singapore	0.075859806	0.087894227	0.06507987
Deaths	Central African Republic	0.674172438	1.388765817	0.270531658
Deaths	Central African Republic	0.281846988	0.617139277	0.098878821
Deaths	Central African Republic	0.473067742	0.870911131	0.228103106
Deaths	Cameroon	0.412209727	0.772084839	0.175930455
Deaths	Cameroon	0.277853211	0.56584557	0.110431366
Deaths	Cameroon	0.344536047	0.587610834	0.17357532
Deaths	Burkina Faso	0.344895686	0.718598333	0.129687025
Deaths	Burkina Faso	0.215175386	0.463749635	0.072246857
Deaths	Burkina Faso	0.277728412	0.499351905	0.128590446
Deaths	Sierra Leone	0.344758678	0.816148395	0.124503816
Deaths	Sierra Leone	0.23650796	0.479110871	0.083037831
Deaths	Sierra Leone	0.292230197	0.577841368	0.133131934
Deaths	United States of America	0.656897216	0.698332257	0.616121727
Deaths	United States of America	0.288683146	0.305006215	0.272243831
Deaths	United States of America	0.469028349	0.491352205	0.44671674
Deaths	Albania	0.255779736	0.394764106	0.150102153
Deaths	Albania	0.098329939	0.202423722	0.041222011

Deaths	Albania	0.175214573	0.266425185	0.10856353
Deaths	Rwanda	0.39468967	0.761626896	0.174262531
Deaths	Rwanda	0.238400432	0.517962619	0.087704255
Deaths	Rwanda	0.30964207	0.538950663	0.155880241
Deaths	Slovakia	0.544359238	0.829153319	0.33359257
Deaths	Slovakia	0.193942056	0.320472928	0.097485681
Deaths	Slovakia	0.366222497	0.512704604	0.249097955
Deaths	Japan	0.274192257	0.288188721	0.260521575
Deaths	Japan	0.128181451	0.135826366	0.119786459
Deaths	Japan	0.201303012	0.209909841	0.19252115
Deaths	Brazil	0.721436582	0.776104566	0.668592473
Deaths	Brazil	0.377941651	0.412723004	0.343882776
Deaths	Brazil	0.541339335	0.574676272	0.509735769
Deaths	Kyrgyzstan	0.066636511	0.088935229	0.049239281
Deaths	Kyrgyzstan	0.036223902	0.046519546	0.027135074
Deaths	Kyrgyzstan	0.050758931	0.066111236	0.038104515
Deaths	Trinidad and Tobago	0.377188049	0.539925375	0.255458317
Deaths	Trinidad and Tobago	0.226264967	0.319614415	0.154164138
Deaths	Trinidad and Tobago	0.302215943	0.409963854	0.215143366
Deaths	Israel	0.534855093	0.65399381	0.426701663
Deaths	Israel	0.277815975	0.345485495	0.219873252
Deaths	Israel	0.403573769	0.472926646	0.33842919
Deaths	Suriname	0.464326691	0.836307438	0.231164442
Deaths	Suriname	0.267897856	0.520081177	0.110796932

Deaths	Suriname	0.364063424	0.597297879	0.204954712
Deaths	Greenland	1.093970374	1.783469596	0.580884276
Deaths	Greenland	0.499187069	0.943146077	0.229922077
Deaths	Greenland	0.820264962	1.246390974	0.481450908
Deaths	Slovenia	1.617110274	1.975951257	1.283038628
Deaths	Slovenia	0.817189786	1.032909949	0.616576086
Deaths	Slovenia	1.223455112	1.47033244	0.984069178
Deaths	Fiji	0.258415223	0.426937867	0.143240678
Deaths	Fiji	0.051226573	0.101682612	0.021979599
Deaths	Fiji	0.155757965	0.252010967	0.090402299
Deaths	Luxembourg	0.694076005	0.846261382	0.55625765
Deaths	Luxembourg	0.461419693	0.566347024	0.372047498
Deaths	Luxembourg	0.580758416	0.680405838	0.491438401
Deaths	Nauru	0.809328086	1.441770014	0.405751894
Deaths	Nauru	0.92573071	1.817592566	0.407586241
Deaths	Nauru	0.860190665	1.515256836	0.441158482
Deaths	Romania	0.418000043	0.510179946	0.335168606
Deaths	Romania	0.169468157	0.213717575	0.131036678
Deaths	Romania	0.293143714	0.352226262	0.238981394
Deaths	Malta	0.478309899	0.584707627	0.38787568
Deaths	Malta	0.195786537	0.253089658	0.148770192
Deaths	Malta	0.338595777	0.400111714	0.282315974
Deaths	Syrian Arab Republic	0.930381778	1.581560263	0.488698884
Deaths	Syrian Arab Republic	0.750519805	1.513972177	0.251532932

Deaths	Syrian Arab Republic	0.840016625	1.376975384	0.448264925
Deaths	Antigua and Barbuda	0.275115505	0.35528259	0.205703576
Deaths	Antigua and Barbuda	0.203293901	0.256455633	0.159201283
Deaths	Antigua and Barbuda	0.237855503	0.287600164	0.195288558
Deaths	Viet Nam	0.042385021	0.084856465	0.015197043
Deaths	Viet Nam	0.020459947	0.089154275	0.002742688
Deaths	Viet Nam	0.030962575	0.06662435	0.012837066
Deaths	Seychelles	0.172789098	0.292283786	0.065131215
Deaths	Seychelles	0.121124035	0.224988825	0.03166185
Deaths	Seychelles	0.147773958	0.231569739	0.060172631
Deaths	Bolivia (Plurinational State of)	0.535219311	0.9876303	0.264379086
Deaths	Bolivia (Plurinational State of)	0.327294868	0.618212231	0.141100537
Deaths	Bolivia (Plurinational State of)	0.429203283	0.706896669	0.247739033
Deaths	Uzbekistan	0.125979921	0.159265782	0.097147352
Deaths	Uzbekistan	0.098552381	0.122828754	0.077008464
Deaths	Uzbekistan	0.112003184	0.138783465	0.088202071
Deaths	New Zealand	0.749278064	0.868778657	0.643925951
Deaths	New Zealand	0.273783326	0.324933468	0.22900025
Deaths	New Zealand	0.506743546	0.571538841	0.450131807
Deaths	Democratic Republic of the Congo	0.499638645	0.912326749	0.238610418
Deaths	Democratic Republic of the Congo	0.304125564	0.660126366	0.109406984
Deaths	Democratic Republic of the Congo	0.401272854	0.679245297	0.210834915
Deaths	Democratic People's Republic of Korea	0.059098215	0.111577536	0.02103164
Deaths	Democratic People's Republic of Korea	0.038723249	0.13346093	0.008271398

Deaths	Democratic People's Republic of Korea	0.048847661	0.097181367	0.022424483
Deaths	Kiribati	0.757937554	1.350520456	0.390795598
Deaths	Kiribati	1.896337592	3.412565953	0.90771711
Deaths	Kiribati	1.349028644	2.2733397	0.735443084
Deaths	Taiwan (Province of China)	0.240464863	0.297309515	0.190522907
Deaths	Taiwan (Province of China)	0.165913033	0.205806335	0.131292802
Deaths	Taiwan (Province of China)	0.202658171	0.236368175	0.171641102
Deaths	Equatorial Guinea	0.542549347	1.093450844	0.240680699
Deaths	Equatorial Guinea	0.283526759	0.622075947	0.094729232
Deaths	Equatorial Guinea	0.402149172	0.747312302	0.19024258
Deaths	Croatia	0.929362097	1.12629044	0.757157624
Deaths	Croatia	0.376748259	0.47192893	0.292736551
Deaths	Croatia	0.648381534	0.783855047	0.530033662
Deaths	Qatar	0.221230092	0.403179187	0.105121355
Deaths	Qatar	0.275532728	0.566988022	0.118019685
Deaths	Qatar	0.236358272	0.396676474	0.129060601
Deaths	Algeria	0.357094835	0.727870746	0.090316576
Deaths	Algeria	0.195939654	0.497547904	0.027300826
Deaths	Algeria	0.27716049	0.518536728	0.077926041
Deaths	Bahamas	0.461060028	0.625588001	0.336220645
Deaths	Bahamas	0.334073925	0.456037112	0.24009671
Deaths	Bahamas	0.394856842	0.51645745	0.298706331
Deaths	Tajikistan	0.004914529	0.009048269	0.002332023
Deaths	Tajikistan	0.001301036	0.002478127	0.00053306

Deaths	Tajikistan	0.003086018	0.005265725	0.001657735
Deaths	Niue	0.474170364	0.800781243	0.261914942
Deaths	Niue	0.656838023	1.318425193	0.318949751
Deaths	Niue	0.569257846	0.994385103	0.326661891
Deaths	Somalia	0.313972647	0.725184363	0.079209434
Deaths	Somalia	0.189904242	0.444297595	0.059923512
Deaths	Somalia	0.247585937	0.494157705	0.091949289
Deaths	China	0.037990475	0.052696414	0.026374957
Deaths	China	0.017479085	0.029112915	0.010636824
Deaths	China	0.027917005	0.038422166	0.020583199
Deaths	Serbia	0.726626232	1.136100588	0.437385764
Deaths	Serbia	0.340481849	0.62575732	0.167490266
Deaths	Serbia	0.530014671	0.771736148	0.344308066
Deaths	Mozambique	0.833583319	1.566928632	0.381079136
Deaths	Mozambique	0.281523489	0.610783544	0.093535821
Deaths	Mozambique	0.540098681	0.938861683	0.267018653
Deaths	Tonga	0.333538725	0.592679896	0.172631865
Deaths	Tonga	0.276399755	0.574196667	0.127451316
Deaths	Tonga	0.307883755	0.546741789	0.169055702
Deaths	Gambia	0.426724186	0.839208856	0.180505888
Deaths	Gambia	0.269798798	0.561249425	0.107420814
Deaths	Gambia	0.348527468	0.613101996	0.17286253
Deaths	Tunisia	0.399257312	0.819259631	0.117988761
Deaths	Tunisia	0.169648921	0.443857441	0.022756859

Deaths	Tunisia	0.282759964	0.55826166	0.081755014
Deaths	Coted'Ivoire	0.400051578	0.800893342	0.16194718
Deaths	Coted'Ivoire	0.224891981	0.466919687	0.077846878
Deaths	Coted'Ivoire	0.318845791	0.585744506	0.153863687
Deaths	Togo	0.421665831	0.805429068	0.186617701
Deaths	Togo	0.255122497	0.532887883	0.096130272
Deaths	Togo	0.333275314	0.578558977	0.170039668
Deaths	Barbados	0.65140087	0.907027695	0.456454055
Deaths	Barbados	0.334337838	0.46508246	0.235884543
Deaths	Barbados	0.484758394	0.64717217	0.355448958
Deaths	Ecuador	0.509498428	0.700253359	0.357917803
Deaths	Ecuador	0.271568443	0.376924527	0.188337394
Deaths	Ecuador	0.387846437	0.511615968	0.283521505
Deaths	Estonia	1.664379914	2.066421163	1.338968739
Deaths	Estonia	0.492665836	0.61987746	0.388901797
Deaths	Estonia	1.051439047	1.262818089	0.87090204
Deaths	Turkmenistan	0.077783083	0.108412836	0.053583352
Deaths	Turkmenistan	0.054281078	0.07561009	0.036893462
Deaths	Turkmenistan	0.06616688	0.089382379	0.046984117
Deaths	Andorra	0.224143977	0.408346934	0.108469829
Deaths	Andorra	0.263907548	0.499961961	0.123839588
Deaths	Andorra	0.242963858	0.4070008	0.133645358
Deaths	Spain	0.648096528	0.778550319	0.542219072
Deaths	Spain	0.250938651	0.306554937	0.201626343

Deaths	Spain	0.446889672	0.514743398	0.387831313
Deaths	Australia	0.470269648	0.564884152	0.38799132
Deaths	Australia	0.161826815	0.19747498	0.129580238
Deaths	Australia	0.312851592	0.364040252	0.267677286
Deaths	Paraguay	1.108445322	1.755309038	0.661816224
Deaths	Paraguay	0.617112495	1.072993705	0.331227805
Deaths	Paraguay	0.862046017	1.268405654	0.563542029
Deaths	Russian Federation	0.644721615	0.719523331	0.568044425
Deaths	Russian Federation	0.184712093	0.208748961	0.158379367
Deaths	Russian Federation	0.395955014	0.436547481	0.35767505
Deaths	Northern Mariana Islands	0.865989871	1.362700522	0.515705677
Deaths	Northern Mariana Islands	0.901210451	1.521264973	0.506833545
Deaths	Northern Mariana Islands	0.909258574	1.363361613	0.579355524
Deaths	Latvia	1.095407538	1.353348654	0.866053077
Deaths	Latvia	0.429849624	0.540888292	0.333927531
Deaths	Latvia	0.74261998	0.885836113	0.613485833
Deaths	Zambia	0.546002297	0.9554621	0.270567438
Deaths	Zambia	0.297594264	0.646030818	0.11818367
Deaths	Zambia	0.423270643	0.716457155	0.232631417
Deaths	Congo	0.615196268	1.116419766	0.295179596
Deaths	Congo	0.392798809	0.798250649	0.157024319
Deaths	Congo	0.505785777	0.844638385	0.277453046
Deaths	Cabo Verde	0.366467969	0.740809482	0.168379159
Deaths	Cabo Verde	0.184278729	0.461895715	0.0567749

Deaths	Cabo Verde	0.27395044	0.523761516	0.138968261
Deaths	Samoa	0.39645881	0.668730637	0.212638036
Deaths	Samoa	0.542822778	1.035490883	0.253042644
Deaths	Samoa	0.467037672	0.775718839	0.26182036
Deaths	Micronesia (Federated States of)	0.560933176	0.956475151	0.302287494
Deaths	Micronesia (Federated States of)	0.667861867	1.287833269	0.302465671
Deaths	Micronesia (Federated States of)	0.610508761	1.032312869	0.33343858
Deaths	Czechia	0.840020502	1.01828321	0.681360324
Deaths	Czechia	0.329185298	0.400788691	0.269264931
Deaths	Czechia	0.586162096	0.696786955	0.485039787
Deaths	Netherlands	0.538196311	0.658563588	0.439062668
Deaths	Netherlands	0.28832294	0.358013205	0.227066954
Deaths	Netherlands	0.413294785	0.48152284	0.353329628
Deaths	Colombia	0.814639255	1.059308106	0.60880027
Deaths	Colombia	0.385241018	0.49643325	0.285312745
Deaths	Colombia	0.587234024	0.737301239	0.459099528
Deaths	Mongolia	0.178181831	0.306187729	0.090565048
Deaths	Mongolia	0.073428424	0.137569187	0.033709785
Deaths	Mongolia	0.122326249	0.18924542	0.074182032
Deaths	Burundi	0.377168858	0.725116274	0.169620703
Deaths	Burundi	0.200901291	0.450756618	0.070340895
Deaths	Burundi	0.294257599	0.513696875	0.148874145
Deaths	Yemen	0.515427937	1.072019409	0.12849187
Deaths	Yemen	0.192233573	0.528558016	0.026975117

Deaths	Yemen	0.35195673	0.701502239	0.095376387
Deaths	Lithuania	0.936708293	1.148973163	0.752229459
Deaths	Lithuania	0.223922288	0.284506429	0.172607951
Deaths	Lithuania	0.556110331	0.662982382	0.458281796
Deaths	Belize	0.184948671	0.248588109	0.135736312
Deaths	Belize	0.095944484	0.127381209	0.069384185
Deaths	Belize	0.139844552	0.176061813	0.10865015
Deaths	Monaco	0.283678497	0.488628728	0.147193128
Deaths	Monaco	0.094588663	0.193803866	0.039690537
Deaths	Monaco	0.187905767	0.309708679	0.105328655
Deaths	Norway	0.599276736	0.655235827	0.547017187
Deaths	Norway	0.188021718	0.207949228	0.168434
Deaths	Norway	0.397770826	0.430360301	0.365872938
Deaths	Myanmar	0.051276001	0.097292373	0.016702443
Deaths	Myanmar	0.030372443	0.13387104	0.005084462
Deaths	Myanmar	0.040010898	0.097065537	0.014400192
Deaths	Lao People's Democratic Republic	0.039111373	0.073720793	0.012606419
Deaths	Lao People's Democratic Republic	0.032091	0.145655483	0.004987405
Deaths	Lao People's Democratic Republic	0.035555804	0.090754665	0.011943617
Deaths	Jordan	0.222406226	0.352538778	0.133597962
Deaths	Jordan	0.162610922	0.292560664	0.086777672
Deaths	Jordan	0.195492775	0.293123189	0.127495498
Deaths	Turkey	0.482758908	0.799487709	0.25879241
Deaths	Turkey	0.303373867	0.573501432	0.128506763

Deaths	Turkey	0.393453741	0.6053047	0.237443617
Deaths	Sudan	0.510717918	1.010868219	0.144512903
Deaths	Sudan	0.204136601	0.551450242	0.031676218
Deaths	Sudan	0.361327203	0.698057667	0.107979705
Deaths	Indonesia	0.038899524	0.07405224	0.011818541
Deaths	Indonesia	0.033513969	0.142106618	0.00675018
Deaths	Indonesia	0.036210791	0.084028596	0.012029199
Deaths	Maldives	0.12972771	0.221162047	0.070944593
Deaths	Maldives	0.100931613	0.196861465	0.043759816
Deaths	Maldives	0.118910006	0.187893118	0.071734611
Deaths	Bulgaria	0.437946886	0.538571513	0.350815763
Deaths	Bulgaria	0.150818657	0.18992477	0.119470693
Deaths	Bulgaria	0.292954306	0.360530778	0.236037313
Deaths	Papua New Guinea	0.278128217	0.495640435	0.140494572
Deaths	Papua New Guinea	0.508653868	1.104010936	0.191057924
Deaths	Papua New Guinea	0.393978895	0.739639202	0.191365214
Deaths	Gabon	0.629941626	1.137846591	0.309764168
Deaths	Gabon	0.300495865	0.604354234	0.11886874
Deaths	Gabon	0.463451394	0.780054247	0.257941447
Deaths	Portugal	0.693231185	0.815524333	0.580045794
Deaths	Portugal	0.252685891	0.313847294	0.201953136
Deaths	Portugal	0.461285803	0.529396935	0.398925448
Deaths	Saint Kitts and Nevis	0.43189935	0.606223804	0.297891767
Deaths	Saint Kitts and Nevis	0.211068778	0.283578978	0.15141701

Deaths	Saint Kitts and Nevis	0.323050834	0.428246717	0.239478649
Deaths	Dominica	0.683016314	1.153906174	0.365744904
Deaths	Dominica	0.134493482	0.230658363	0.070297194
Deaths	Dominica	0.422550275	0.676846179	0.246496604
Deaths	Iraq	0.537868789	0.950093051	0.252271772
Deaths	Iraq	0.356656467	0.707630866	0.102414133
Deaths	Iraq	0.450752194	0.769729493	0.212473736
Deaths	Dominican Republic	0.522873801	0.886347495	0.281506157
Deaths	Dominican Republic	0.225066165	0.409286547	0.107893158
Deaths	Dominican Republic	0.374105705	0.576653027	0.230144901
Deaths	Comoros	0.388902005	0.7771106	0.166072713
Deaths	Comoros	0.243667318	0.501456312	0.092187366
Deaths	Comoros	0.314503392	0.5552582	0.161416764
Deaths	Peru	0.334303916	0.562765176	0.179821771
Deaths	Peru	0.180151785	0.327073187	0.087439952
Deaths	Peru	0.255710243	0.404378444	0.156082493
Deaths	Chad	0.278945343	0.581984419	0.103879442
Deaths	Chad	0.229583356	0.510525672	0.078345741
Deaths	Chad	0.253870217	0.46741384	0.117986144
Deaths	Djibouti	0.409397741	0.796837813	0.179717197
Deaths	Djibouti	0.220005076	0.479695271	0.076982939
Deaths	Djibouti	0.323926487	0.582135605	0.161921017
Deaths	Marshall Islands	0.558287556	0.960745322	0.281453011
Deaths	Marshall Islands	0.742572048	1.580617497	0.286125685

Deaths	Marshall Islands	0.644618809	1.179330641	0.311031217
Deaths	Guinea-Bissau	0.530204173	1.030923674	0.235677949
Deaths	Guinea-Bissau	0.317316006	0.687353379	0.120887961
Deaths	Guinea-Bissau	0.416670616	0.734007019	0.208209542
Deaths	San Marino	0.114351797	0.212175165	0.047830026
Deaths	San Marino	0.150975042	0.279371866	0.066563855
Deaths	San Marino	0.133355489	0.224899433	0.066804781
Deaths	Belarus	0.145854641	0.192858968	0.112118135
Deaths	Belarus	0.024471118	0.032899144	0.017911739
Deaths	Belarus	0.080170452	0.104227143	0.062390881
Deaths	Bangladesh	0.291843848	0.552166287	0.131615467
Deaths	Bangladesh	0.203508968	0.396103633	0.084035674
Deaths	Bangladesh	0.247095287	0.415878219	0.13462958
Deaths	Austria	0.563942166	0.681135152	0.459725149
Deaths	Austria	0.204953061	0.251480704	0.164307812
Deaths	Austria	0.383343933	0.447598519	0.325964952
Deaths	Malaysia	0.199378619	0.322242555	0.113015182
Deaths	Malaysia	0.114864893	0.203313588	0.060972655
Deaths	Malaysia	0.157922615	0.232499674	0.102179273
Deaths	Denmark	0.63911173	0.778821065	0.51659042
Deaths	Denmark	0.325714864	0.405012132	0.262237571
Deaths	Denmark	0.482873483	0.562734409	0.409492292
Deaths	United Arab Emirates	0.546878884	0.886671713	0.301321261
Deaths	United Arab Emirates	0.812747771	1.381969363	0.461329035

Deaths	United Arab Emirates	0.601623527	0.90890236	0.381162143
Deaths	Cyprus	0.89884955	1.420649193	0.536275307
Deaths	Cyprus	0.444980308	0.774594332	0.239777129
Deaths	Cyprus	0.664432715	0.968595957	0.449005961
Deaths	Namibia	0.417834751	0.784579794	0.183433801
Deaths	Namibia	0.178336188	0.398899	0.060107123
Deaths	Namibia	0.289265456	0.501946488	0.150327541
Deaths	Bosnia and Herzegovina	0.386783856	0.694961661	0.18161865
Deaths	Bosnia and Herzegovina	0.178681365	0.354269231	0.073006262
Deaths	Bosnia and Herzegovina	0.280080879	0.454200055	0.151763035
Deaths	Ukraine	0.29925933	0.442304632	0.18664473
Deaths	Ukraine	0.067937656	0.106289172	0.039567916
Deaths	Ukraine	0.174897803	0.24558064	0.117541977
Deaths	Ghana	0.465440433	0.878378369	0.218461179
Deaths	Ghana	0.255816352	0.555598304	0.103836504
Deaths	Ghana	0.351950816	0.607376999	0.18944594
Deaths	Hungary	1.901158103	2.218015837	1.612096715
Deaths	Hungary	0.707828852	0.822423178	0.599995323
Deaths	Hungary	1.282071986	1.473953032	1.105921056
Deaths	Bahrain	0.218255438	0.346663617	0.125810446
Deaths	Bahrain	0.496045821	0.850828904	0.276838391
Deaths	Bahrain	0.317172631	0.470218111	0.210559902
Deaths	United Republic of Tanzania	0.467445857	0.969370785	0.20735706
Deaths	United Republic of Tanzania	0.236999028	0.507139653	0.089999394

Deaths	United Republic of Tanzania	0.348240067	0.642730172	0.180528102
Deaths	Cuba	0.72458693	0.92491408	0.559473145
Deaths	Cuba	0.285580571	0.363736093	0.217369041
Deaths	Cuba	0.50167531	0.612773504	0.40235411
Deaths	Mali	0.167619176	0.348031888	0.055927598
Deaths	Mali	0.238829379	0.521226231	0.085031242
Deaths	Mali	0.202807345	0.379443772	0.088760031
Deaths	Liberia	0.326011074	0.698220688	0.118252266
Deaths	Liberia	0.273815543	0.570458198	0.104475226
Deaths	Liberia	0.301399002	0.555323768	0.140946363
Deaths	Bhutan	0.274602142	0.488279805	0.135927
Deaths	Bhutan	0.205003295	0.408135781	0.083510753
Deaths	Bhutan	0.241412877	0.401829809	0.132650963
Deaths	Botswana	0.281640952	0.546603732	0.128762602
Deaths	Botswana	0.140610772	0.296779191	0.052606818
Deaths	Botswana	0.208132981	0.359840096	0.107698659
Deaths	American Samoa	0.786042215	1.303520313	0.433915087
Deaths	American Samoa	0.453554007	0.769482679	0.232262545
Deaths	American Samoa	0.624684609	0.971237666	0.367932524
Deaths	Tokelau	0.412510233	0.725193614	0.214612978
Deaths	Tokelau	0.790941386	1.50449357	0.387429692
Deaths	Tokelau	0.598983986	1.036261357	0.341309008
Deaths	Honduras	0.578155716	0.977107132	0.314570817
Deaths	Honduras	0.396579687	0.745228617	0.162901947

Deaths	Honduras	0.481855665	0.736394247	0.28773524
Deaths	Belgium	0.646358063	0.785943032	0.523809885
Deaths	Belgium	0.417095247	0.515326505	0.334916388
Deaths	Belgium	0.531778049	0.615518203	0.458012326
Deaths	United Kingdom	0.709697787	0.74098877	0.679698054
Deaths	United Kingdom	0.335473995	0.351129069	0.319795245
Deaths	United Kingdom	0.51977873	0.538244314	0.500886518
Deaths	South Africa	0.436314951	0.58088856	0.321662033
Deaths	South Africa	0.241634681	0.403187615	0.135020037
Deaths	South Africa	0.334456645	0.449615417	0.252570519
Deaths	Sweden	0.406362677	0.506089393	0.323204821
Deaths	Sweden	0.155237357	0.191714558	0.12299917
Deaths	Sweden	0.282365829	0.337508245	0.233176187
Deaths	Uganda	0.407992521	0.752869981	0.189172223
Deaths	Uganda	0.166095009	0.36938177	0.05587024
Deaths	Uganda	0.279101993	0.480405361	0.139665404
Deaths	Egypt	0.591538924	0.949091367	0.33631969
Deaths	Egypt	0.266687252	0.501242564	0.110988283
Deaths	Egypt	0.434506205	0.661918378	0.266385563
Deaths	Mexico	0.523838905	0.623770561	0.42939939
Deaths	Mexico	0.290829849	0.34591366	0.239838577
Deaths	Mexico	0.401375867	0.457530495	0.348748445
Deaths	Vanuatu	0.485521881	0.824896537	0.258397905
Deaths	Vanuatu	0.467534937	0.958174384	0.188199705

Deaths	Vanuatu	0.477056081	0.817370161	0.252932441
Deaths	Argentina	0.960179335	1.167838967	0.789746282
Deaths	Argentina	0.386002763	0.47047181	0.315379683
Deaths	Argentina	0.661802877	0.767283227	0.56736677
Deaths	Palau	0.416029997	0.692773886	0.229275942
Deaths	Palau	0.613923844	1.205417027	0.286071257
Deaths	Palau	0.487811477	0.827776397	0.274099449
Deaths	Costa Rica	0.950453376	1.188419794	0.739385455
Deaths	Costa Rica	0.45430411	0.570307881	0.353865474
Deaths	Costa Rica	0.687227273	0.827085348	0.56151023
Deaths	El Salvador	0.242135197	0.380058486	0.146564925
Deaths	El Salvador	0.093475792	0.155013425	0.049888965
Deaths	El Salvador	0.156597414	0.227468202	0.102772291
Deaths	Republic of Moldova	0.170205939	0.216803821	0.130403086
Deaths	Republic of Moldova	0.021711286	0.028197278	0.016560764
Deaths	Republic of Moldova	0.090199068	0.113360435	0.070668211
Deaths	Guinea	0.321342756	0.660719622	0.118426932
Deaths	Guinea	0.244910384	0.517938237	0.092130377
Deaths	Guinea	0.280951097	0.521581296	0.132349104
Deaths	Grenada	1.220561302	1.576141423	0.931912905
Deaths	Grenada	0.102163342	0.134425105	0.076062183
Deaths	Grenada	0.681836797	0.869992162	0.526537537
Deaths	Libya	0.572848719	1.403910216	0.125150881
Deaths	Libya	0.352171762	0.904119665	0.035012906

Deaths	Libya	0.465730854	1.029481715	0.106871764
Deaths	Philippines	0.0709945	0.104115867	0.04957305
Deaths	Philippines	0.040595571	0.063757172	0.020551223
Deaths	Philippines	0.055680322	0.076762351	0.042787264
Deaths	Iran (Islamic Republic of)	0.398076129	0.540422905	0.305233732
Deaths	Iran (Islamic Republic of)	0.163158965	0.338927321	0.102087826
Deaths	Iran (Islamic Republic of)	0.281846668	0.41377877	0.217794862
Deaths	Puerto Rico	1.143509548	1.474233358	0.861289035
Deaths	Puerto Rico	0.367223213	0.482894914	0.273331506
Deaths	Puerto Rico	0.735810643	0.927903444	0.568705888
Deaths	Afghanistan	0.57909971	1.322216714	0.14579306
Deaths	Afghanistan	0.160561318	0.560633956	0.020639612
Deaths	Afghanistan	0.354963694	0.800270762	0.098604029
Deaths	India	0.329557936	0.450583371	0.203856237
Deaths	India	0.216342759	0.320615916	0.12508455
Deaths	India	0.273318677	0.350907783	0.202283748
Deaths	Switzerland	0.40297284	0.492919972	0.327124693
Deaths	Switzerland	0.168044204	0.210714041	0.130438411
Deaths	Switzerland	0.286740067	0.334940933	0.241326222
Deaths	Eritrea	0.485180936	0.922582529	0.217064375
Deaths	Eritrea	0.250547561	0.52716066	0.092865974
Deaths	Eritrea	0.362084011	0.62673358	0.187073781
Deaths	Finland	0.819366565	0.992723862	0.667561111
Deaths	Finland	0.327141963	0.406814094	0.25385276

Deaths	Finland	0.574054054	0.673606767	0.483522788
Deaths	Guatemala	0.275788976	0.362665936	0.205076802
Deaths	Guatemala	0.160509019	0.208300069	0.120223759
Deaths	Guatemala	0.214111264	0.266640307	0.169423849
Deaths	North Macedonia	0.225340759	0.360363251	0.134364012
Deaths	North Macedonia	0.136185602	0.245720566	0.065155512
Deaths	North Macedonia	0.181569607	0.266284317	0.120543726
Deaths	Guyana	1.548809555	2.193022093	1.046956558
Deaths	Guyana	0.598158162	0.857059086	0.403399589
Deaths	Guyana	1.059565209	1.444552086	0.752050039
Deaths	Mauritania	0.257719035	0.519848835	0.107771409
Deaths	Mauritania	0.289146401	0.599139521	0.12248909
Deaths	Mauritania	0.273311565	0.479254617	0.143702076
Deaths	Lesotho	0.508035459	0.97625605	0.226834567
Deaths	Lesotho	0.236524406	0.526231606	0.079311968
Deaths	Lesotho	0.359294819	0.630825661	0.178905286
Deaths	Ethiopia	0.233814673	0.36975903	0.127854722
Deaths	Ethiopia	0.168190082	0.306611157	0.072504738
Deaths	Ethiopia	0.201099974	0.296674122	0.114917777
Deaths	France	0.551801677	0.681732896	0.438717412
Deaths	France	0.248892306	0.30774402	0.197239835
Deaths	France	0.396985486	0.466145197	0.336210467
Deaths	Kuwait	0.28580152	0.379456843	0.214701053
Deaths	Kuwait	0.196481871	0.251676776	0.150222579

Deaths	Kuwait	0.24493633	0.306554984	0.193274583
Deaths	Haiti	0.786874524	1.490619326	0.324746236
Deaths	Haiti	0.436169426	0.970953016	0.142572679
Deaths	Haiti	0.602601496	1.058992196	0.288740287
Deaths	Lebanon	0.827230405	1.313516754	0.488062808
Deaths	Lebanon	0.496367922	0.865965649	0.258328963
Deaths	Lebanon	0.653890153	0.968637955	0.423588086
Deaths	Germany	0.885532999	1.069603733	0.719931166
Deaths	Germany	0.319800903	0.393224365	0.256288842
Deaths	Germany	0.603960197	0.706049459	0.510774202
Deaths	Niger	0.162748552	0.376998145	0.041801276
Deaths	Niger	0.213974571	0.470406923	0.069917795
Deaths	Niger	0.188379137	0.35434263	0.069639604
Deaths	Tuvalu	0.508687262	0.885317065	0.270869579
Deaths	Tuvalu	0.627663931	1.236025332	0.294178505
Deaths	Tuvalu	0.557300346	0.939049028	0.322100402
DALYs (Disability-Adjusted Life Years)	Chile	18.43756821	22.55967928	14.92560834
DALYs (Disability-Adjusted Life Years)	Chile	4.538941313	5.609487684	3.618308318
DALYs (Disability-Adjusted Life Years)	Chile	11.25502504	13.33142647	9.431159662
DALYs (Disability-Adjusted Life Years)	Nepal	11.61668629	20.44775038	5.857130077
DALYs (Disability-Adjusted Life Years)	Nepal	8.364586211	16.60436932	3.37594477
DALYs (Disability-Adjusted Life Years)	Nepal	9.858959393	16.17336793	5.454678319
DALYs (Disability-Adjusted Life Years)	Armenia	5.6272114	6.850393052	4.537807102
DALYs (Disability-Adjusted Life Years)	Armenia	2.469108538	3.03422597	1.965971353

DALYs (Disability-Adjusted Life Years)	Armenia	3.954461822	4.752596319	3.204781829
DALYs (Disability-Adjusted Life Years)	Thailand	9.394256925	16.57645717	4.854684556
DALYs (Disability-Adjusted Life Years)	Thailand	4.826752755	8.765770727	2.102488516
DALYs (Disability-Adjusted Life Years)	Thailand	7.036451736	11.31040511	4.121220409
DALYs (Disability-Adjusted Life Years)	Zimbabwe	23.64037749	45.65714937	10.60163989
DALYs (Disability-Adjusted Life Years)	Zimbabwe	11.81546513	27.13564126	4.211001658
DALYs (Disability-Adjusted Life Years)	Zimbabwe	17.25972568	31.13077682	8.698729484
DALYs (Disability-Adjusted Life Years)	Bermuda	97.27160441	125.5996943	74.06793862
DALYs (Disability-Adjusted Life Years)	Bermuda	28.13643738	37.66348004	20.97732188
DALYs (Disability-Adjusted Life Years)	Bermuda	62.29247616	78.72565095	48.71108633
DALYs (Disability-Adjusted Life Years)	Poland	42.38699529	47.38118892	37.54585414
DALYs (Disability-Adjusted Life Years)	Poland	13.09115652	14.86464016	11.3163102
DALYs (Disability-Adjusted Life Years)	Poland	27.53866661	30.4036945	24.89724997
DALYs (Disability-Adjusted Life Years)	Morocco	18.16716606	36.93517297	5.367098149
DALYs (Disability-Adjusted Life Years)	Morocco	10.0321068	27.00511083	1.770335893
DALYs (Disability-Adjusted Life Years)	Morocco	14.09991382	27.26552992	4.373712262
DALYs (Disability-Adjusted Life Years)	Greece	25.36637461	30.08355817	21.11040261
DALYs (Disability-Adjusted Life Years)	Greece	11.65879609	14.23753958	9.398074963
DALYs (Disability-Adjusted Life Years)	Greece	18.35562236	21.09256327	15.86510024
DALYs (Disability-Adjusted Life Years)	Montenegro	9.611531384	14.78799196	5.969216359
DALYs (Disability-Adjusted Life Years)	Montenegro	3.975242086	7.253286123	2.198770452
DALYs (Disability-Adjusted Life Years)	Montenegro	6.754780706	9.898226045	4.521474102
DALYs (Disability-Adjusted Life Years)	Kenya	12.8089729	17.87309563	8.681819871
DALYs (Disability-Adjusted Life Years)	Kenya	7.690232574	13.6163427	4.170292833

DALYs (Disability-Adjusted Life Years)	Kenya	10.22682801	14.19265679	7.302134855
DALYs (Disability-Adjusted Life Years)	Nigeria	11.91419019	25.10850482	5.772452331
DALYs (Disability-Adjusted Life Years)	Nigeria	6.787857063	11.89745553	2.754227038
DALYs (Disability-Adjusted Life Years)	Nigeria	9.133112971	16.90976282	4.812916984
DALYs (Disability-Adjusted Life Years)	Mauritius	30.34108278	37.12893511	24.27287946
DALYs (Disability-Adjusted Life Years)	Mauritius	16.10615748	19.9062618	12.72823305
DALYs (Disability-Adjusted Life Years)	Mauritius	23.19118928	27.18384867	19.37694684
DALYs (Disability-Adjusted Life Years)	Timor-Leste	1.404275463	2.942726282	0.3886242
DALYs (Disability-Adjusted Life Years)	Timor-Leste	1.215018507	5.128066846	0.230116141
DALYs (Disability-Adjusted Life Years)	Timor-Leste	1.31107559	3.214070702	0.427802464
DALYs (Disability-Adjusted Life Years)	Jamaica	5.504199283	7.96549175	3.617563317
DALYs (Disability-Adjusted Life Years)	Jamaica	3.150399038	4.478845062	2.156560611
DALYs (Disability-Adjusted Life Years)	Jamaica	4.320343441	5.964955851	3.050415174
DALYs (Disability-Adjusted Life Years)	Ireland	18.7720903	23.3189842	14.89858663
DALYs (Disability-Adjusted Life Years)	Ireland	7.913550642	9.99785713	6.135256849
DALYs (Disability-Adjusted Life Years)	Ireland	13.268708	15.91423075	10.98771253
DALYs (Disability-Adjusted Life Years)	Cook Islands	4.032508304	6.686483599	2.238185893
DALYs (Disability-Adjusted Life Years)	Cook Islands	16.23793669	35.85535736	7.15711895
DALYs (Disability-Adjusted Life Years)	Cook Islands	10.57558008	21.38179544	5.299549549
DALYs (Disability-Adjusted Life Years)	Nicaragua	8.423416347	12.91873639	5.182556588
DALYs (Disability-Adjusted Life Years)	Nicaragua	4.891720594	7.96842477	2.688490265
DALYs (Disability-Adjusted Life Years)	Nicaragua	6.541213434	9.216161416	4.471623109
DALYs (Disability-Adjusted Life Years)	Eswatini	24.14069333	46.60751651	10.07405684
DALYs (Disability-Adjusted Life Years)	Eswatini	9.035496256	20.66869098	2.862171834

DALYs (Disability-Adjusted Life Years)	Eswatini	16.15221557	29.23446719	7.516282395
DALYs (Disability-Adjusted Life Years)	South Sudan	15.20660739	31.02437669	5.444181121
DALYs (Disability-Adjusted Life Years)	South Sudan	8.102174057	18.12749742	2.786103108
DALYs (Disability-Adjusted Life Years)	South Sudan	11.63830504	21.26042924	5.254065381
DALYs (Disability-Adjusted Life Years)	Madagascar	32.41320841	58.55983268	14.58783764
DALYs (Disability-Adjusted Life Years)	Madagascar	18.57416143	39.60185599	6.92032682
DALYs (Disability-Adjusted Life Years)	Madagascar	25.3169235	42.10613945	13.34253696
DALYs (Disability-Adjusted Life Years)	Sri Lanka	7.744708261	13.39481704	3.968168764
DALYs (Disability-Adjusted Life Years)	Sri Lanka	3.374550985	7.146352756	1.616436961
DALYs (Disability-Adjusted Life Years)	Sri Lanka	5.462591185	8.910590186	3.045371031
DALYs (Disability-Adjusted Life Years)	Iceland	30.87553849	37.88542564	24.5846826
DALYs (Disability-Adjusted Life Years)	Iceland	15.60807308	19.38160291	12.34335384
DALYs (Disability-Adjusted Life Years)	Iceland	23.3898137	27.8739613	19.1983556
DALYs (Disability-Adjusted Life Years)	Benin	11.32897744	22.23059605	4.673570466
DALYs (Disability-Adjusted Life Years)	Benin	5.769847587	12.57059129	2.03207255
DALYs (Disability-Adjusted Life Years)	Benin	8.461928657	15.01350182	4.045775802
DALYs (Disability-Adjusted Life Years)	Venezuela (Bolivarian Republic of)	28.86406434	41.1449079	19.69831993
DALYs (Disability-Adjusted Life Years)	Venezuela (Bolivarian Republic of)	14.52896476	20.85416615	9.817203309
DALYs (Disability-Adjusted Life Years)	Venezuela (Bolivarian Republic of)	21.41273834	29.1713469	15.30111962
DALYs (Disability-Adjusted Life Years)	Saint Lucia	22.24902334	29.19320069	16.59344919
DALYs (Disability-Adjusted Life Years)	Saint Lucia	8.013430178	10.67346528	5.826290677
DALYs (Disability-Adjusted Life Years)	Saint Lucia	15.15155962	19.37629721	11.5819224
DALYs (Disability-Adjusted Life Years)	Saint Vincent and the Grenadines	29.21680628	37.20213367	22.38207109
DALYs (Disability-Adjusted Life Years)	Saint Vincent and the Grenadines	11.79215976	15.33228568	8.831762585

DALYs (Disability-Adjusted Life Years)	Saint Vincent and the Grenadines	20.79168453	25.55049685	16.62999314
DALYs (Disability-Adjusted Life Years)	Palestine	9.661355519	15.63155069	5.655327837
DALYs (Disability-Adjusted Life Years)	Palestine	3.404008209	6.671708276	1.566291219
DALYs (Disability-Adjusted Life Years)	Palestine	6.601245525	10.04804598	4.198916258
DALYs (Disability-Adjusted Life Years)	Sao Tome and Principe	13.37025454	32.7017816	5.434088537
DALYs (Disability-Adjusted Life Years)	Sao Tome and Principe	10.73462354	22.93005378	4.074638983
DALYs (Disability-Adjusted Life Years)	Sao Tome and Principe	12.06700778	24.21183331	5.861569005
DALYs (Disability-Adjusted Life Years)	Georgia	17.68643479	22.44962794	13.55163897
DALYs (Disability-Adjusted Life Years)	Georgia	11.36890774	13.9087605	9.121882694
DALYs (Disability-Adjusted Life Years)	Georgia	14.42016962	17.83268984	11.47433883
DALYs (Disability-Adjusted Life Years)	Solomon Islands	14.1575899	26.06794692	6.833745326
DALYs (Disability-Adjusted Life Years)	Solomon Islands	14.99224093	31.72117215	6.253778407
DALYs (Disability-Adjusted Life Years)	Solomon Islands	14.64096397	26.56551455	7.383583337
DALYs (Disability-Adjusted Life Years)	Republic of Korea	5.190784371	7.618365928	3.375039497
DALYs (Disability-Adjusted Life Years)	Republic of Korea	3.17498776	5.180457208	1.763765441
DALYs (Disability-Adjusted Life Years)	Republic of Korea	4.203183125	5.817216037	2.984147201
DALYs (Disability-Adjusted Life Years)	Oman	7.023842387	11.77599364	3.824127484
DALYs (Disability-Adjusted Life Years)	Oman	0.678963011	1.48820258	0.282202298
DALYs (Disability-Adjusted Life Years)	Oman	4.734002524	7.783484606	2.657587146
DALYs (Disability-Adjusted Life Years)	Canada	17.54613991	21.33527397	14.15983186
DALYs (Disability-Adjusted Life Years)	Canada	7.849670079	9.626459495	6.273608445
DALYs (Disability-Adjusted Life Years)	Canada	12.63335489	14.81627916	10.73562441
DALYs (Disability-Adjusted Life Years)	Malawi	23.98496442	45.11278604	11.35444567
DALYs (Disability-Adjusted Life Years)	Malawi	8.599094595	18.42424694	3.036794297

DALYs (Disability-Adjusted Life Years)	Malawi	16.00353337	27.10487693	8.425810523
DALYs (Disability-Adjusted Life Years)	Uruguay	57.18548986	69.26615269	46.65936328
DALYs (Disability-Adjusted Life Years)	Uruguay	22.56004517	27.73254617	17.93109809
DALYs (Disability-Adjusted Life Years)	Uruguay	39.12215418	45.54534773	33.38038926
DALYs (Disability-Adjusted Life Years)	Angola	22.48630533	41.36108095	10.68648662
DALYs (Disability-Adjusted Life Years)	Angola	12.11452736	25.35547538	4.522037702
DALYs (Disability-Adjusted Life Years)	Angola	16.96431985	28.72656981	9.110997721
DALYs (Disability-Adjusted Life Years)	Senegal	13.25826698	26.13292543	5.428564819
DALYs (Disability-Adjusted Life Years)	Senegal	9.321109669	19.77047312	3.431309315
DALYs (Disability-Adjusted Life Years)	Senegal	11.20808531	20.11010457	5.397392548
DALYs (Disability-Adjusted Life Years)	Guam	43.52919733	64.23530863	28.65245221
DALYs (Disability-Adjusted Life Years)	Guam	34.52132821	51.84285453	21.60810121
DALYs (Disability-Adjusted Life Years)	Guam	39.15713131	54.26633326	27.71307018
DALYs (Disability-Adjusted Life Years)	Azerbaijan	1.200392738	2.208935232	0.629074342
DALYs (Disability-Adjusted Life Years)	Azerbaijan	1.509618795	2.356489914	0.929931563
DALYs (Disability-Adjusted Life Years)	Azerbaijan	1.354283441	2.120582637	0.861917829
DALYs (Disability-Adjusted Life Years)	United States Virgin Islands	36.34961898	69.16665199	17.11710755
DALYs (Disability-Adjusted Life Years)	United States Virgin Islands	13.35154765	29.22203535	5.093011228
DALYs (Disability-Adjusted Life Years)	United States Virgin Islands	24.60136998	43.62624597	13.06728686
DALYs (Disability-Adjusted Life Years)	Saudi Arabia	12.62766787	23.35119801	6.064569081
DALYs (Disability-Adjusted Life Years)	Saudi Arabia	13.50313756	27.95934305	5.491071902
DALYs (Disability-Adjusted Life Years)	Saudi Arabia	12.96747522	21.74828808	7.218468767
DALYs (Disability-Adjusted Life Years)	Brunei Darussalam	18.98036844	29.58489142	11.53797336
DALYs (Disability-Adjusted Life Years)	Brunei Darussalam	12.1785766	22.34222385	5.874558186

DALYs (Disability-Adjusted Life Years)	Brunei Darussalam	15.76327551	23.0831947	10.40775938
DALYs (Disability-Adjusted Life Years)	Pakistan	14.93396688	24.2002783	8.574798836
DALYs (Disability-Adjusted Life Years)	Pakistan	9.554934174	19.05677406	3.995100929
DALYs (Disability-Adjusted Life Years)	Pakistan	12.30562011	19.05167816	7.6420738
DALYs (Disability-Adjusted Life Years)	Panama	20.23887924	26.89492311	14.51470671
DALYs (Disability-Adjusted Life Years)	Panama	10.80806313	14.24360554	7.942092932
DALYs (Disability-Adjusted Life Years)	Panama	15.50911499	19.82533446	11.5664384
DALYs (Disability-Adjusted Life Years)	Cambodia	1.263508457	2.405262996	0.467726412
DALYs (Disability-Adjusted Life Years)	Cambodia	1.008158635	4.051355955	0.199163974
DALYs (Disability-Adjusted Life Years)	Cambodia	1.12244952	2.786007259	0.428027773
DALYs (Disability-Adjusted Life Years)	Italy	21.84265845	23.80047364	20.02990719
DALYs (Disability-Adjusted Life Years)	Italy	9.338640773	10.17222633	8.517207623
DALYs (Disability-Adjusted Life Years)	Italy	15.53439367	16.75611694	14.38707378
DALYs (Disability-Adjusted Life Years)	Kazakhstan	4.621056037	6.14511849	3.41944264
DALYs (Disability-Adjusted Life Years)	Kazakhstan	2.778016221	3.483506997	2.130432351
DALYs (Disability-Adjusted Life Years)	Kazakhstan	3.653368743	4.68303201	2.815784778
DALYs (Disability-Adjusted Life Years)	Singapore	4.323118165	5.385663286	3.448667483
DALYs (Disability-Adjusted Life Years)	Singapore	2.238808616	2.795925112	1.763044105
DALYs (Disability-Adjusted Life Years)	Singapore	3.308168726	3.954243012	2.777654982
DALYs (Disability-Adjusted Life Years)	Central African Republic	25.9317719	54.1213255	10.26509033
DALYs (Disability-Adjusted Life Years)	Central African Republic	10.447933	23.35424058	3.609583025
DALYs (Disability-Adjusted Life Years)	Central African Republic	18.00674895	33.50909382	8.543402323
DALYs (Disability-Adjusted Life Years)	Cameroon	16.22896358	30.58938026	6.908182416
DALYs (Disability-Adjusted Life Years)	Cameroon	10.81989095	22.31860888	4.222569067

DALYs (Disability-Adjusted Life Years)	Cameroon	13.50360603	23.18275929	6.785988518
DALYs (Disability-Adjusted Life Years)	Burkina Faso	13.00238431	27.14076278	4.897457493
DALYs (Disability-Adjusted Life Years)	Burkina Faso	8.116635955	17.71023868	2.706120079
DALYs (Disability-Adjusted Life Years)	Burkina Faso	10.47357828	18.95260583	4.846610211
DALYs (Disability-Adjusted Life Years)	Sierra Leone	13.62492041	32.22810407	4.902011383
DALYs (Disability-Adjusted Life Years)	Sierra Leone	9.210236661	18.99022678	3.181381515
DALYs (Disability-Adjusted Life Years)	Sierra Leone	11.47211809	22.73241193	5.248092926
DALYs (Disability-Adjusted Life Years)	United States of America	25.75093118	27.53576228	23.98839785
DALYs (Disability-Adjusted Life Years)	United States of America	11.21990858	12.01789745	10.56937274
DALYs (Disability-Adjusted Life Years)	United States of America	18.36000045	19.42147759	17.3411519
DALYs (Disability-Adjusted Life Years)	Albania	10.61461855	16.2833235	6.342178341
DALYs (Disability-Adjusted Life Years)	Albania	4.113787533	7.973716117	1.890903121
DALYs (Disability-Adjusted Life Years)	Albania	7.301716171	11.03891961	4.673799315
DALYs (Disability-Adjusted Life Years)	Rwanda	15.76056697	30.51528283	6.948895931
DALYs (Disability-Adjusted Life Years)	Rwanda	9.561662734	20.98973579	3.437953729
DALYs (Disability-Adjusted Life Years)	Rwanda	12.41130695	21.69628079	6.271513803
DALYs (Disability-Adjusted Life Years)	Slovakia	20.92767978	31.71795758	13.07661135
DALYs (Disability-Adjusted Life Years)	Slovakia	7.52830836	12.2501321	3.871148473
DALYs (Disability-Adjusted Life Years)	Slovakia	14.15719962	19.84242332	9.730966439
DALYs (Disability-Adjusted Life Years)	Japan	11.2534129	11.98066301	10.59895527
DALYs (Disability-Adjusted Life Years)	Japan	5.478522381	5.908348843	5.101539008
DALYs (Disability-Adjusted Life Years)	Japan	8.379074837	8.875921875	7.935145817
DALYs (Disability-Adjusted Life Years)	Brazil	28.41169823	30.66183164	26.29922967
DALYs (Disability-Adjusted Life Years)	Brazil	14.90920396	16.30923894	13.58736667

DALYs (Disability-Adjusted Life Years)	Brazil	21.37140502	22.76025576	20.1166934
DALYs (Disability-Adjusted Life Years)	Kyrgyzstan	3.172291226	4.309900913	2.334494389
DALYs (Disability-Adjusted Life Years)	Kyrgyzstan	1.674727911	2.167418676	1.262160005
DALYs (Disability-Adjusted Life Years)	Kyrgyzstan	2.390526363	3.148527268	1.798500694
DALYs (Disability-Adjusted Life Years)	Trinidad and Tobago	16.26288511	22.94772593	11.22207927
DALYs (Disability-Adjusted Life Years)	Trinidad and Tobago	9.399462543	13.2948	6.481641626
DALYs (Disability-Adjusted Life Years)	Trinidad and Tobago	12.85930465	17.42448199	9.286309397
DALYs (Disability-Adjusted Life Years)	Israel	20.15797119	24.61595448	16.13378912
DALYs (Disability-Adjusted Life Years)	Israel	10.27810729	12.75535563	8.155265524
DALYs (Disability-Adjusted Life Years)	Israel	15.13388829	17.69183236	12.73237304
DALYs (Disability-Adjusted Life Years)	Suriname	19.59560126	35.05213975	9.805454997
DALYs (Disability-Adjusted Life Years)	Suriname	11.13364117	21.72191229	4.634005618
DALYs (Disability-Adjusted Life Years)	Suriname	15.27916966	24.94040178	8.628740232
DALYs (Disability-Adjusted Life Years)	Greenland	42.04614886	68.90917466	22.42386395
DALYs (Disability-Adjusted Life Years)	Greenland	19.24271493	36.4875818	8.662321343
DALYs (Disability-Adjusted Life Years)	Greenland	31.50491707	47.97780248	18.48403541
DALYs (Disability-Adjusted Life Years)	Slovenia	60.81392481	74.63817124	48.20193271
DALYs (Disability-Adjusted Life Years)	Slovenia	30.00936264	38.06790797	22.72182259
DALYs (Disability-Adjusted Life Years)	Slovenia	45.71463053	55.08849525	36.81743349
DALYs (Disability-Adjusted Life Years)	Fiji	11.65417225	19.40392059	6.42879682
DALYs (Disability-Adjusted Life Years)	Fiji	2.368078556	4.829024386	1.001037105
DALYs (Disability-Adjusted Life Years)	Fiji	7.070433462	11.51245776	4.060498697
DALYs (Disability-Adjusted Life Years)	Luxembourg	25.97285726	31.82278204	20.87718139
DALYs (Disability-Adjusted Life Years)	Luxembourg	16.86389135	20.70338506	13.48527819

DALYs (Disability-Adjusted Life Years)	Luxembourg	21.53779099	25.35030342	18.17598793
DALYs (Disability-Adjusted Life Years)	Nauru	37.94256674	68.37953567	19.0330738
DALYs (Disability-Adjusted Life Years)	Nauru	48.6237501	95.25913387	21.53838705
DALYs (Disability-Adjusted Life Years)	Nauru	43.08028504	76.1842204	22.18332112
DALYs (Disability-Adjusted Life Years)	Romania	18.27589372	22.67694408	14.47076004
DALYs (Disability-Adjusted Life Years)	Romania	7.558855971	10.10269963	5.627411367
DALYs (Disability-Adjusted Life Years)	Romania	12.91434345	16.07075867	10.27937927
DALYs (Disability-Adjusted Life Years)	Malta	18.68418616	22.82413958	15.14743525
DALYs (Disability-Adjusted Life Years)	Malta	7.303994296	9.355550267	5.571731897
DALYs (Disability-Adjusted Life Years)	Malta	13.0747811	15.51413998	10.93278508
DALYs (Disability-Adjusted Life Years)	Syrian Arab Republic	39.62262522	67.43018179	20.81901701
DALYs (Disability-Adjusted Life Years)	Syrian Arab Republic	31.77086304	64.09236864	10.79326297
DALYs (Disability-Adjusted Life Years)	Syrian Arab Republic	35.62783963	58.48292113	19.06273173
DALYs (Disability-Adjusted Life Years)	Antigua and Barbuda	10.82687115	14.10593826	8.080943692
DALYs (Disability-Adjusted Life Years)	Antigua and Barbuda	7.873651483	9.921150593	6.184799267
DALYs (Disability-Adjusted Life Years)	Antigua and Barbuda	9.29848718	11.2642991	7.607952973
DALYs (Disability-Adjusted Life Years)	Viet Nam	1.712925359	3.404033227	0.651026599
DALYs (Disability-Adjusted Life Years)	Viet Nam	0.857175782	3.494061319	0.150728833
DALYs (Disability-Adjusted Life Years)	Viet Nam	1.271086405	2.671534633	0.54473799
DALYs (Disability-Adjusted Life Years)	Seychelles	6.841521829	11.60072757	2.652581595
DALYs (Disability-Adjusted Life Years)	Seychelles	5.061258023	9.488910524	1.341378205
DALYs (Disability-Adjusted Life Years)	Seychelles	5.98079441	9.44108241	2.450593126
DALYs (Disability-Adjusted Life Years)	Bolivia (Plurinational State of)	21.23899916	39.56628129	10.68370038
DALYs (Disability-Adjusted Life Years)	Bolivia (Plurinational State of)	13.24145401	25.23991683	5.760219371

DALYs (Disability-Adjusted Life Years)	Bolivia (Plurinational State of)	17.17237891	28.51010687	9.937312376
DALYs (Disability-Adjusted Life Years)	Uzbekistan	5.694685512	7.304571716	4.365390806
DALYs (Disability-Adjusted Life Years)	Uzbekistan	4.089440707	5.21361918	3.15138655
DALYs (Disability-Adjusted Life Years)	Uzbekistan	4.873367074	6.099358012	3.823964987
DALYs (Disability-Adjusted Life Years)	New Zealand	28.36863338	33.01420565	24.30890123
DALYs (Disability-Adjusted Life Years)	New Zealand	10.00284792	11.86700968	8.387095926
DALYs (Disability-Adjusted Life Years)	New Zealand	19.03023096	21.55129662	16.7875501
DALYs (Disability-Adjusted Life Years)	Democratic Republic of the Congo	18.99626917	34.98178072	8.962748512
DALYs (Disability-Adjusted Life Years)	Democratic Republic of the Congo	11.13679822	24.36218906	3.964168229
DALYs (Disability-Adjusted Life Years)	Democratic Republic of the Congo	15.06316016	25.66499571	7.861835007
DALYs (Disability-Adjusted Life Years)	Democratic People's Republic of Korea	2.563585166	4.835714656	0.950347471
DALYs (Disability-Adjusted Life Years)	Democratic People's Republic of Korea	1.68831026	5.604726534	0.412139638
DALYs (Disability-Adjusted Life Years)	Democratic People's Republic of Korea	2.127781983	4.163657616	1.009818887
DALYs (Disability-Adjusted Life Years)	Kiribati	33.70855769	60.33563412	17.0222498
DALYs (Disability-Adjusted Life Years)	Kiribati	97.00693587	174.7165422	45.92223029
DALYs (Disability-Adjusted Life Years)	Kiribati	66.52964521	112.4665675	35.78567492
DALYs (Disability-Adjusted Life Years)	Taiwan (Province of China)	9.903074113	12.22618072	7.830375063
DALYs (Disability-Adjusted Life Years)	Taiwan (Province of China)	6.721446831	8.346808475	5.330545358
DALYs (Disability-Adjusted Life Years)	Taiwan (Province of China)	8.296050217	9.678882686	7.000780566
DALYs (Disability-Adjusted Life Years)	Equatorial Guinea	21.00947029	42.56146219	9.197119178
DALYs (Disability-Adjusted Life Years)	Equatorial Guinea	10.68094943	23.92911959	3.540514172
DALYs (Disability-Adjusted Life Years)	Equatorial Guinea	15.52779243	29.11243558	7.287002013
DALYs (Disability-Adjusted Life Years)	Croatia	35.78039955	43.46333037	29.06816219
DALYs (Disability-Adjusted Life Years)	Croatia	14.26679033	17.87682344	11.11215923

DALYs (Disability-Adjusted Life Years)	Croatia	24.89623549	30.06322859	20.42845168
DALYs (Disability-Adjusted Life Years)	Qatar	9.84757635	17.64578827	4.841759708
DALYs (Disability-Adjusted Life Years)	Qatar	10.87495337	22.17931071	4.783850486
DALYs (Disability-Adjusted Life Years)	Qatar	10.07522862	16.6784778	5.603542475
DALYs (Disability-Adjusted Life Years)	Algeria	15.40960545	31.46296228	3.942453472
DALYs (Disability-Adjusted Life Years)	Algeria	8.578190252	21.69443081	1.322340794
DALYs (Disability-Adjusted Life Years)	Algeria	12.02189248	22.64240907	3.413101729
DALYs (Disability-Adjusted Life Years)	Bahamas	19.74830355	26.90293766	14.33594175
DALYs (Disability-Adjusted Life Years)	Bahamas	13.67607377	18.68343178	9.761913106
DALYs (Disability-Adjusted Life Years)	Bahamas	16.58262668	21.6533311	12.52119854
DALYs (Disability-Adjusted Life Years)	Tajikistan	0.576608388	1.103877396	0.251542701
DALYs (Disability-Adjusted Life Years)	Tajikistan	0.254616938	0.497995043	0.117390171
DALYs (Disability-Adjusted Life Years)	Tajikistan	0.413152382	0.778206771	0.197400266
DALYs (Disability-Adjusted Life Years)	Niue	22.31746326	37.9487395	12.21475404
DALYs (Disability-Adjusted Life Years)	Niue	37.01120203	73.84579308	18.01260443
DALYs (Disability-Adjusted Life Years)	Niue	29.81401074	52.2763542	17.10249457
DALYs (Disability-Adjusted Life Years)	Somalia	12.49550668	29.36247139	3.157562827
DALYs (Disability-Adjusted Life Years)	Somalia	7.46071991	17.63030392	2.306107837
DALYs (Disability-Adjusted Life Years)	Somalia	9.839895425	19.87868597	3.650224472
DALYs (Disability-Adjusted Life Years)	China	1.687214517	2.343219311	1.182021181
DALYs (Disability-Adjusted Life Years)	China	0.778393542	1.258595245	0.497716796
DALYs (Disability-Adjusted Life Years)	China	1.242741859	1.677941845	0.92635588
DALYs (Disability-Adjusted Life Years)	Serbia	27.08279798	41.86818806	16.48860749
DALYs (Disability-Adjusted Life Years)	Serbia	12.59340179	22.85472788	6.418139043

DALYs (Disability-Adjusted Life Years)	Serbia	19.75268858	28.63793126	13.10590881
DALYs (Disability-Adjusted Life Years)	Mozambique	33.72717378	64.08348251	15.21943379
DALYs (Disability-Adjusted Life Years)	Mozambique	11.0586595	24.23212461	3.644067479
DALYs (Disability-Adjusted Life Years)	Mozambique	21.67001159	38.10251975	10.65576544
DALYs (Disability-Adjusted Life Years)	Tonga	15.22695669	27.43855036	7.796091005
DALYs (Disability-Adjusted Life Years)	Tonga	13.92677085	28.88368714	6.374321947
DALYs (Disability-Adjusted Life Years)	Tonga	14.74771498	26.37383526	8.046367167
DALYs (Disability-Adjusted Life Years)	Gambia	17.03125812	33.62196523	7.152729553
DALYs (Disability-Adjusted Life Years)	Gambia	10.40273642	22.01557715	4.042808767
DALYs (Disability-Adjusted Life Years)	Gambia	13.70442299	24.14129579	6.793788266
DALYs (Disability-Adjusted Life Years)	Tunisia	16.97146209	35.1153493	5.012661047
DALYs (Disability-Adjusted Life Years)	Tunisia	7.304762849	19.16947032	1.094303787
DALYs (Disability-Adjusted Life Years)	Tunisia	12.06657718	23.85552648	3.549957877
DALYs (Disability-Adjusted Life Years)	Coted'Ivoire	15.79475512	31.77622388	6.41204321
DALYs (Disability-Adjusted Life Years)	Coted'Ivoire	8.803030899	18.40096222	3.065484266
DALYs (Disability-Adjusted Life Years)	Coted'Ivoire	12.54291011	23.13899591	6.03213222
DALYs (Disability-Adjusted Life Years)	Togo	16.40597016	31.46241022	7.164246252
DALYs (Disability-Adjusted Life Years)	Togo	9.881449152	20.82414362	3.662176659
DALYs (Disability-Adjusted Life Years)	Togo	12.93799235	22.50454796	6.575532634
DALYs (Disability-Adjusted Life Years)	Barbados	26.66020765	37.14758742	18.7984536
DALYs (Disability-Adjusted Life Years)	Barbados	13.40230108	18.61626895	9.488857886
DALYs (Disability-Adjusted Life Years)	Barbados	19.72214102	26.48124967	14.55524555
DALYs (Disability-Adjusted Life Years)	Ecuador	21.10344682	28.87120705	14.89465518
DALYs (Disability-Adjusted Life Years)	Ecuador	10.92629104	15.02227789	7.642644566

DALYs (Disability-Adjusted Life Years)	Ecuador	15.91858414	21.00263127	11.72308954
DALYs (Disability-Adjusted Life Years)	Estonia	62.94155589	78.48379988	50.56872419
DALYs (Disability-Adjusted Life Years)	Estonia	19.35402037	24.35508376	15.18082476
DALYs (Disability-Adjusted Life Years)	Estonia	40.32640447	48.59563347	33.41814481
DALYs (Disability-Adjusted Life Years)	Turkmenistan	3.848827912	5.336242789	2.665605319
DALYs (Disability-Adjusted Life Years)	Turkmenistan	2.535151163	3.468731627	1.718846367
DALYs (Disability-Adjusted Life Years)	Turkmenistan	3.194796244	4.284103489	2.306341648
DALYs (Disability-Adjusted Life Years)	Andorra	9.080438554	16.17640315	4.558065501
DALYs (Disability-Adjusted Life Years)	Andorra	10.00879558	18.83875221	4.738750363
DALYs (Disability-Adjusted Life Years)	Andorra	9.519535461	15.82312212	5.444795898
DALYs (Disability-Adjusted Life Years)	Spain	25.15360338	30.33246627	20.81084387
DALYs (Disability-Adjusted Life Years)	Spain	9.735829042	11.89448137	7.835649639
DALYs (Disability-Adjusted Life Years)	Spain	17.36861733	20.06822578	14.9328846
DALYs (Disability-Adjusted Life Years)	Australia	18.34063871	22.08504586	15.04601319
DALYs (Disability-Adjusted Life Years)	Australia	6.090123151	7.466801147	4.877336571
DALYs (Disability-Adjusted Life Years)	Australia	12.0997598	14.1310772	10.28132237
DALYs (Disability-Adjusted Life Years)	Paraguay	43.16259205	68.37511227	25.64670615
DALYs (Disability-Adjusted Life Years)	Paraguay	24.50067466	42.77266655	13.21974746
DALYs (Disability-Adjusted Life Years)	Paraguay	33.82839196	49.68444684	22.03496976
DALYs (Disability-Adjusted Life Years)	Russian Federation	26.01135581	29.11426593	22.93945751
DALYs (Disability-Adjusted Life Years)	Russian Federation	7.503425912	8.539641132	6.449870036
DALYs (Disability-Adjusted Life Years)	Russian Federation	16.1041847	17.78307279	14.53841314
DALYs (Disability-Adjusted Life Years)	Northern Mariana Islands	38.34916473	61.00072944	22.65313282
DALYs (Disability-Adjusted Life Years)	Northern Mariana Islands	45.33582607	76.4663504	25.38940252

DALYs (Disability-Adjusted Life Years)	Northern Mariana Islands	42.63162738	64.11154164	27.0868075
DALYs (Disability-Adjusted Life Years)	Latvia	43.48302962	53.72635541	34.53131112
DALYs (Disability-Adjusted Life Years)	Latvia	16.70651376	20.92505825	13.01679787
DALYs (Disability-Adjusted Life Years)	Latvia	29.43080083	35.29857389	24.26474288
DALYs (Disability-Adjusted Life Years)	Zambia	21.69647807	38.49077823	10.54124757
DALYs (Disability-Adjusted Life Years)	Zambia	12.00367357	26.52393959	4.596392192
DALYs (Disability-Adjusted Life Years)	Zambia	16.91583288	29.01219717	9.166511223
DALYs (Disability-Adjusted Life Years)	Congo	23.91031329	44.00567702	11.35305761
DALYs (Disability-Adjusted Life Years)	Congo	14.75811285	30.53281313	5.76832468
DALYs (Disability-Adjusted Life Years)	Congo	19.39149014	32.69156487	10.5545461
DALYs (Disability-Adjusted Life Years)	Cabo Verde	14.29906744	29.32446982	6.523024686
DALYs (Disability-Adjusted Life Years)	Cabo Verde	7.337133348	18.48606086	2.23079233
DALYs (Disability-Adjusted Life Years)	Cabo Verde	10.80113064	20.73595619	5.45947511
DALYs (Disability-Adjusted Life Years)	Samoa	18.13369762	31.00332765	9.625143985
DALYs (Disability-Adjusted Life Years)	Samoa	27.59360393	52.93419199	12.76824219
DALYs (Disability-Adjusted Life Years)	Samoa	22.71589797	38.243111	12.60447965
DALYs (Disability-Adjusted Life Years)	Micronesia (Federated States of)	25.65935673	44.01667704	13.655503
DALYs (Disability-Adjusted Life Years)	Micronesia (Federated States of)	34.35986683	66.18139148	15.30383593
DALYs (Disability-Adjusted Life Years)	Micronesia (Federated States of)	29.80103789	50.58022523	16.11995389
DALYs (Disability-Adjusted Life Years)	Czechia	33.02772218	39.83626126	26.85626202
DALYs (Disability-Adjusted Life Years)	Czechia	12.63520944	15.26728423	10.24214189
DALYs (Disability-Adjusted Life Years)	Czechia	22.93222909	27.24753412	18.9375297
DALYs (Disability-Adjusted Life Years)	Netherlands	20.85578468	25.5821047	16.93689335
DALYs (Disability-Adjusted Life Years)	Netherlands	10.92451534	13.5790117	8.636784122

DALYs (Disability-Adjusted Life Years)	Netherlands	15.89688378	18.54419357	13.53311273
DALYs (Disability-Adjusted Life Years)	Colombia	32.36017418	42.18108911	24.35114335
DALYs (Disability-Adjusted Life Years)	Colombia	14.98570354	19.31612919	11.19279861
DALYs (Disability-Adjusted Life Years)	Colombia	23.22441449	29.12635709	18.19626361
DALYs (Disability-Adjusted Life Years)	Mongolia	7.514581613	12.58429422	4.057729966
DALYs (Disability-Adjusted Life Years)	Mongolia	3.119885769	5.665390399	1.515925226
DALYs (Disability-Adjusted Life Years)	Mongolia	5.188458691	7.808632076	3.253024218
DALYs (Disability-Adjusted Life Years)	Burundi	15.20450456	29.38715742	6.771529726
DALYs (Disability-Adjusted Life Years)	Burundi	8.230065257	18.50629433	2.827689432
DALYs (Disability-Adjusted Life Years)	Burundi	11.90823789	20.921484	5.987358911
DALYs (Disability-Adjusted Life Years)	Yemen	21.01196774	44.16444864	5.12269527
DALYs (Disability-Adjusted Life Years)	Yemen	7.958358662	21.70985414	1.152065291
DALYs (Disability-Adjusted Life Years)	Yemen	14.42539249	29.07006309	3.802861665
DALYs (Disability-Adjusted Life Years)	Lithuania	36.73665898	44.72666773	29.59976461
DALYs (Disability-Adjusted Life Years)	Lithuania	8.754444138	11.07340248	6.768215093
DALYs (Disability-Adjusted Life Years)	Lithuania	21.93970601	26.03126752	18.1295677
DALYs (Disability-Adjusted Life Years)	Belize	8.110707157	10.84034242	5.92593158
DALYs (Disability-Adjusted Life Years)	Belize	3.69882376	4.864245849	2.69571989
DALYs (Disability-Adjusted Life Years)	Belize	5.873475632	7.43222081	4.552627933
DALYs (Disability-Adjusted Life Years)	Monaco	11.8592182	20.23351737	6.298636811
DALYs (Disability-Adjusted Life Years)	Monaco	3.859027021	7.768527264	1.69181219
DALYs (Disability-Adjusted Life Years)	Monaco	7.800887427	12.70327012	4.464120483
DALYs (Disability-Adjusted Life Years)	Norway	21.65901392	23.73869001	19.725392
DALYs (Disability-Adjusted Life Years)	Norway	6.894235589	7.628172589	6.219347918

DALYs (Disability-Adjusted Life Years)	Norway	14.43369165	15.7101372	13.24063707
DALYs (Disability-Adjusted Life Years)	Myanmar	2.157909194	4.069330105	0.762824265
DALYs (Disability-Adjusted Life Years)	Myanmar	1.280850564	5.490299298	0.250653868
DALYs (Disability-Adjusted Life Years)	Myanmar	1.689597336	3.985609693	0.654355462
DALYs (Disability-Adjusted Life Years)	Lao People's Democratic Republic	1.632381188	3.06446318	0.572739837
DALYs (Disability-Adjusted Life Years)	Lao People's Democratic Republic	1.372111028	6.003903087	0.252095426
DALYs (Disability-Adjusted Life Years)	Lao People's Democratic Republic	1.500548481	3.769098447	0.538610381
DALYs (Disability-Adjusted Life Years)	Jordan	9.803798726	15.50420006	5.918486648
DALYs (Disability-Adjusted Life Years)	Jordan	6.752858809	11.94130083	3.651856504
DALYs (Disability-Adjusted Life Years)	Jordan	8.428568542	12.55645885	5.524832029
DALYs (Disability-Adjusted Life Years)	Turkey	20.25334742	33.64797713	10.8941552
DALYs (Disability-Adjusted Life Years)	Turkey	12.44076291	23.53475962	5.396663478
DALYs (Disability-Adjusted Life Years)	Turkey	16.37530464	25.28377532	9.888583099
DALYs (Disability-Adjusted Life Years)	Sudan	22.06725256	44.09508428	6.108150478
DALYs (Disability-Adjusted Life Years)	Sudan	9.001980991	24.30987615	1.439052416
DALYs (Disability-Adjusted Life Years)	Sudan	15.63219849	30.50772592	4.59618111
DALYs (Disability-Adjusted Life Years)	Indonesia	1.604765147	3.023466255	0.545578019
DALYs (Disability-Adjusted Life Years)	Indonesia	1.368922747	5.586745727	0.312507384
DALYs (Disability-Adjusted Life Years)	Indonesia	1.48736678	3.327498373	0.539518319
DALYs (Disability-Adjusted Life Years)	Maldives	5.470094523	9.290054503	3.009641973
DALYs (Disability-Adjusted Life Years)	Maldives	3.977919422	7.751851033	1.720305605
DALYs (Disability-Adjusted Life Years)	Maldives	4.915202148	7.775764231	2.975575101
DALYs (Disability-Adjusted Life Years)	Bulgaria	17.47556766	21.40220178	14.029033
DALYs (Disability-Adjusted Life Years)	Bulgaria	6.058926203	7.609195296	4.76168244

DALYs (Disability-Adjusted Life Years)	Bulgaria	11.75250525	14.35088004	9.540957135
DALYs (Disability-Adjusted Life Years)	Papua New Guinea	12.53698202	22.56142171	6.228639484
DALYs (Disability-Adjusted Life Years)	Papua New Guinea	27.20424614	58.78965543	9.832935176
DALYs (Disability-Adjusted Life Years)	Papua New Guinea	19.83100227	37.65833446	9.323068157
DALYs (Disability-Adjusted Life Years)	Gabon	24.29714083	44.15978089	11.81107339
DALYs (Disability-Adjusted Life Years)	Gabon	11.22544935	22.93341743	4.353620543
DALYs (Disability-Adjusted Life Years)	Gabon	17.64702088	29.93960651	9.684132409
DALYs (Disability-Adjusted Life Years)	Portugal	25.61485924	30.20757731	21.30286934
DALYs (Disability-Adjusted Life Years)	Portugal	9.480394812	11.76985797	7.57964965
DALYs (Disability-Adjusted Life Years)	Portugal	17.15361863	19.73644863	14.80517813
DALYs (Disability-Adjusted Life Years)	Saint Kitts and Nevis	16.60993836	23.43510153	11.43990874
DALYs (Disability-Adjusted Life Years)	Saint Kitts and Nevis	8.108034274	10.89635002	5.807225371
DALYs (Disability-Adjusted Life Years)	Saint Kitts and Nevis	12.4282827	16.57495641	9.195420366
DALYs (Disability-Adjusted Life Years)	Dominica	27.42565243	46.76181242	14.59708551
DALYs (Disability-Adjusted Life Years)	Dominica	5.626159719	9.648421138	2.985800559
DALYs (Disability-Adjusted Life Years)	Dominica	17.02386833	27.47948325	10.0023714
DALYs (Disability-Adjusted Life Years)	Iraq	22.30306257	39.81777575	10.46723406
DALYs (Disability-Adjusted Life Years)	Iraq	15.04389679	29.87549949	4.455924497
DALYs (Disability-Adjusted Life Years)	Iraq	18.82640336	32.38287638	8.927539531
DALYs (Disability-Adjusted Life Years)	Dominican Republic	22.92845309	38.92543456	12.30941718
DALYs (Disability-Adjusted Life Years)	Dominican Republic	9.548745233	17.40606695	4.559282898
DALYs (Disability-Adjusted Life Years)	Dominican Republic	16.26083881	25.13213837	10.00145684
DALYs (Disability-Adjusted Life Years)	Comoros	15.7098344	31.50773097	6.647432834
DALYs (Disability-Adjusted Life Years)	Comoros	10.04944486	21.08544855	3.688616777

DALYs (Disability-Adjusted Life Years)	Comoros	12.82714398	22.72566846	6.505238074
DALYs (Disability-Adjusted Life Years)	Peru	13.9164152	23.42665979	7.589895226
DALYs (Disability-Adjusted Life Years)	Peru	7.497783905	13.63589851	3.697881693
DALYs (Disability-Adjusted Life Years)	Peru	10.65456556	16.71985148	6.530670248
DALYs (Disability-Adjusted Life Years)	Chad	10.89104452	22.69021468	4.073846814
DALYs (Disability-Adjusted Life Years)	Chad	8.642777948	19.44771303	2.915254335
DALYs (Disability-Adjusted Life Years)	Chad	9.745369895	18.07970997	4.532808563
DALYs (Disability-Adjusted Life Years)	Djibouti	16.49401982	32.25935441	7.166903843
DALYs (Disability-Adjusted Life Years)	Djibouti	8.574470679	18.95843826	2.9259259
DALYs (Disability-Adjusted Life Years)	Djibouti	12.8882197	23.38517437	6.339584081
DALYs (Disability-Adjusted Life Years)	Marshall Islands	25.50794067	44.66745137	12.71588722
DALYs (Disability-Adjusted Life Years)	Marshall Islands	38.38848889	81.59984587	14.65653363
DALYs (Disability-Adjusted Life Years)	Marshall Islands	31.63759524	58.45305584	15.11940034
DALYs (Disability-Adjusted Life Years)	Guinea-Bissau	21.06984321	41.50791122	9.221934311
DALYs (Disability-Adjusted Life Years)	Guinea-Bissau	12.43094552	27.64219956	4.553733977
DALYs (Disability-Adjusted Life Years)	Guinea-Bissau	16.45504294	29.41358096	8.188474796
DALYs (Disability-Adjusted Life Years)	San Marino	4.793881282	8.503461764	2.313558255
DALYs (Disability-Adjusted Life Years)	San Marino	5.793028288	10.56528105	2.68241924
DALYs (Disability-Adjusted Life Years)	San Marino	5.309808294	8.706131525	2.860145683
DALYs (Disability-Adjusted Life Years)	Belarus	6.31274184	8.299591979	4.780088391
DALYs (Disability-Adjusted Life Years)	Belarus	1.262824631	1.803360367	0.892414947
DALYs (Disability-Adjusted Life Years)	Belarus	3.600663115	4.73960381	2.756131167
DALYs (Disability-Adjusted Life Years)	Bangladesh	11.24807619	21.41435182	5.059550961
DALYs (Disability-Adjusted Life Years)	Bangladesh	8.003558208	15.82969979	3.231467835

DALYs (Disability-Adjusted Life Years)	Bangladesh	9.597129261	16.28615035	5.216470444
DALYs (Disability-Adjusted Life Years)	Austria	22.67204668	27.60440825	18.38497013
DALYs (Disability-Adjusted Life Years)	Austria	8.185919982	10.13967726	6.548990335
DALYs (Disability-Adjusted Life Years)	Austria	15.41339888	18.12917873	13.03231648
DALYs (Disability-Adjusted Life Years)	Malaysia	7.832454664	12.68734341	4.451551146
DALYs (Disability-Adjusted Life Years)	Malaysia	4.464893333	7.939976381	2.353075438
DALYs (Disability-Adjusted Life Years)	Malaysia	6.189735955	9.148174543	4.007150799
DALYs (Disability-Adjusted Life Years)	Denmark	24.74798785	30.21478382	19.99224268
DALYs (Disability-Adjusted Life Years)	Denmark	12.24739333	15.11880959	9.887621534
DALYs (Disability-Adjusted Life Years)	Denmark	18.52921	21.67282199	15.62219003
DALYs (Disability-Adjusted Life Years)	United Arab Emirates	24.95525055	40.75553711	13.62826273
DALYs (Disability-Adjusted Life Years)	United Arab Emirates	33.25751278	57.2771011	18.74166338
DALYs (Disability-Adjusted Life Years)	United Arab Emirates	26.26537183	39.89934787	16.57198344
DALYs (Disability-Adjusted Life Years)	Cyprus	33.91966091	53.82506466	20.37995911
DALYs (Disability-Adjusted Life Years)	Cyprus	15.88997469	27.68588566	8.590726833
DALYs (Disability-Adjusted Life Years)	Cyprus	24.62412904	35.91995823	16.61796468
DALYs (Disability-Adjusted Life Years)	Namibia	17.31517526	33.07392652	7.488580482
DALYs (Disability-Adjusted Life Years)	Namibia	7.483037783	17.25018276	2.408146326
DALYs (Disability-Adjusted Life Years)	Namibia	12.08261625	21.4160071	6.141852438
DALYs (Disability-Adjusted Life Years)	Bosnia and Herzegovina	15.06667734	26.53868176	7.410100816
DALYs (Disability-Adjusted Life Years)	Bosnia and Herzegovina	6.977822821	13.59965562	3.130266764
DALYs (Disability-Adjusted Life Years)	Bosnia and Herzegovina	10.94097874	17.63314544	6.13310791
DALYs (Disability-Adjusted Life Years)	Ukraine	12.97513984	18.79057997	8.211985416
DALYs (Disability-Adjusted Life Years)	Ukraine	3.070669546	4.681245202	1.82344751

DALYs (Disability-Adjusted Life Years)	Ukraine	7.707662098	10.6417768	5.301896537
DALYs (Disability-Adjusted Life Years)	Ghana	18.22769744	34.64089415	8.44035776
DALYs (Disability-Adjusted Life Years)	Ghana	9.837422774	21.38244985	3.943284858
DALYs (Disability-Adjusted Life Years)	Ghana	13.70603029	23.58871552	7.352544925
DALYs (Disability-Adjusted Life Years)	Hungary	72.23458445	84.82673203	61.18717435
DALYs (Disability-Adjusted Life Years)	Hungary	27.00472442	31.65572833	22.86650827
DALYs (Disability-Adjusted Life Years)	Hungary	48.96155854	56.69604249	42.08936561
DALYs (Disability-Adjusted Life Years)	Bahrain	9.738358074	15.3783702	5.755419719
DALYs (Disability-Adjusted Life Years)	Bahrain	20.21053532	34.52306252	11.44346241
DALYs (Disability-Adjusted Life Years)	Bahrain	13.41362591	19.75083607	9.006408467
DALYs (Disability-Adjusted Life Years)	United Republic of Tanzania	19.03746004	39.45295193	8.35176426
DALYs (Disability-Adjusted Life Years)	United Republic of Tanzania	9.347330802	20.1561251	3.477263053
DALYs (Disability-Adjusted Life Years)	United Republic of Tanzania	14.00138978	25.84558033	7.207573879
DALYs (Disability-Adjusted Life Years)	Cuba	28.70337229	36.53293489	22.15659866
DALYs (Disability-Adjusted Life Years)	Cuba	10.74869387	13.68298506	8.177305573
DALYs (Disability-Adjusted Life Years)	Cuba	19.63891059	23.96039528	15.77315199
DALYs (Disability-Adjusted Life Years)	Mali	6.709976242	14.02367968	2.265233763
DALYs (Disability-Adjusted Life Years)	Mali	9.619786214	21.40384054	3.312030814
DALYs (Disability-Adjusted Life Years)	Mali	8.165848477	15.47650165	3.568640076
DALYs (Disability-Adjusted Life Years)	Liberia	12.92832782	27.73073873	4.761402203
DALYs (Disability-Adjusted Life Years)	Liberia	10.72416274	22.5545398	3.984937665
DALYs (Disability-Adjusted Life Years)	Liberia	11.88975172	21.9166704	5.585879815
DALYs (Disability-Adjusted Life Years)	Bhutan	10.74869659	19.31098176	5.270738898
DALYs (Disability-Adjusted Life Years)	Bhutan	7.89871198	15.94411969	3.10971416

DALYs (Disability-Adjusted Life Years)	Bhutan	9.393203714	15.77478395	5.132356914
DALYs (Disability-Adjusted Life Years)	Botswana	11.88379805	23.34475431	5.320937936
DALYs (Disability-Adjusted Life Years)	Botswana	6.097112019	13.14103696	2.186955129
DALYs (Disability-Adjusted Life Years)	Botswana	8.896599021	15.59734184	4.533871197
DALYs (Disability-Adjusted Life Years)	American Samoa	34.79362012	58.51670161	18.62158077
DALYs (Disability-Adjusted Life Years)	American Samoa	22.32621846	37.88218442	11.35616265
DALYs (Disability-Adjusted Life Years)	American Samoa	28.75307516	45.16684764	16.59313085
DALYs (Disability-Adjusted Life Years)	Tokelau	20.30268407	35.8898624	10.53303178
DALYs (Disability-Adjusted Life Years)	Tokelau	44.34298047	84.31149774	21.85157218
DALYs (Disability-Adjusted Life Years)	Tokelau	32.13298336	55.63570133	18.40696884
DALYs (Disability-Adjusted Life Years)	Honduras	20.99477453	35.78344399	11.33889779
DALYs (Disability-Adjusted Life Years)	Honduras	14.62007442	27.73501366	5.933753584
DALYs (Disability-Adjusted Life Years)	Honduras	17.6123246	27.14272975	10.45202046
DALYs (Disability-Adjusted Life Years)	Belgium	25.840006	31.5275335	20.88912355
DALYs (Disability-Adjusted Life Years)	Belgium	16.10794556	19.86969794	12.89996049
DALYs (Disability-Adjusted Life Years)	Belgium	20.98290918	24.34804745	18.02446246
DALYs (Disability-Adjusted Life Years)	United Kingdom	27.42194679	28.82946071	26.14445458
DALYs (Disability-Adjusted Life Years)	United Kingdom	12.61229947	13.263109	11.97864467
DALYs (Disability-Adjusted Life Years)	United Kingdom	19.90593852	20.77169379	19.088826
DALYs (Disability-Adjusted Life Years)	South Africa	18.74793624	25.07217083	13.76683473
DALYs (Disability-Adjusted Life Years)	South Africa	9.938116161	17.03374025	5.476725142
DALYs (Disability-Adjusted Life Years)	South Africa	14.20001789	19.27265135	10.68815608
DALYs (Disability-Adjusted Life Years)	Sweden	15.79004475	19.68959381	12.55453666
DALYs (Disability-Adjusted Life Years)	Sweden	5.880353748	7.243708757	4.658127133

DALYs (Disability-Adjusted Life Years)	Sweden	10.90681914	13.11858617	8.991316568
DALYs (Disability-Adjusted Life Years)	Uganda	16.50761766	30.5787407	7.583401663
DALYs (Disability-Adjusted Life Years)	Uganda	6.793968173	15.24838279	2.25896845
DALYs (Disability-Adjusted Life Years)	Uganda	11.34780674	19.63414128	5.684322174
DALYs (Disability-Adjusted Life Years)	Egypt	29.16556896	46.96810207	16.54719241
DALYs (Disability-Adjusted Life Years)	Egypt	11.9794747	22.37091016	5.016208675
DALYs (Disability-Adjusted Life Years)	Egypt	20.83563693	31.78553649	12.84633471
DALYs (Disability-Adjusted Life Years)	Mexico	20.94009393	24.79224489	17.29252509
DALYs (Disability-Adjusted Life Years)	Mexico	11.57008641	13.75597232	9.545995795
DALYs (Disability-Adjusted Life Years)	Mexico	16.03040616	18.2557433	14.01025335
DALYs (Disability-Adjusted Life Years)	Vanuatu	22.39050808	38.34186122	11.66449346
DALYs (Disability-Adjusted Life Years)	Vanuatu	23.98873445	49.30415076	9.467449036
DALYs (Disability-Adjusted Life Years)	Vanuatu	23.2536975	40.16009168	12.09849358
DALYs (Disability-Adjusted Life Years)	Argentina	35.89705366	43.48255828	29.43022058
DALYs (Disability-Adjusted Life Years)	Argentina	14.56920582	17.75443136	11.92496218
DALYs (Disability-Adjusted Life Years)	Argentina	24.8527512	28.8023367	21.30053391
DALYs (Disability-Adjusted Life Years)	Palau	18.8516242	31.45569573	10.34936622
DALYs (Disability-Adjusted Life Years)	Palau	35.95896976	70.12996032	16.74883027
DALYs (Disability-Adjusted Life Years)	Palau	25.64360695	43.95191032	14.25451467
DALYs (Disability-Adjusted Life Years)	Costa Rica	37.49414178	46.88373672	29.15053987
DALYs (Disability-Adjusted Life Years)	Costa Rica	18.06328801	22.64007448	14.04220552
DALYs (Disability-Adjusted Life Years)	Costa Rica	27.2098781	32.74920486	22.18188596
DALYs (Disability-Adjusted Life Years)	El Salvador	10.15147701	15.89609769	6.217430952
DALYs (Disability-Adjusted Life Years)	El Salvador	3.916324234	6.334901435	2.196978523

DALYs (Disability-Adjusted Life Years)	El Salvador	6.587290602	9.497618436	4.378059082
DALYs (Disability-Adjusted Life Years)	Republic of Moldova	7.276697656	9.356714021	5.603309998
DALYs (Disability-Adjusted Life Years)	Republic of Moldova	1.161098195	1.679481576	0.808640249
DALYs (Disability-Adjusted Life Years)	Republic of Moldova	4.01548184	5.16683523	3.141309388
DALYs (Disability-Adjusted Life Years)	Guinea	12.68434202	26.18667642	4.67895932
DALYs (Disability-Adjusted Life Years)	Guinea	9.438184356	20.20585247	3.497992353
DALYs (Disability-Adjusted Life Years)	Guinea	10.95604957	20.42084384	5.170877503
DALYs (Disability-Adjusted Life Years)	Grenada	48.86898151	63.2461938	37.14604744
DALYs (Disability-Adjusted Life Years)	Grenada	4.006746458	5.268818381	2.972535834
DALYs (Disability-Adjusted Life Years)	Grenada	27.25318102	34.90376365	20.95366445
DALYs (Disability-Adjusted Life Years)	Libya	26.14923211	63.88601509	5.839138824
DALYs (Disability-Adjusted Life Years)	Libya	15.71669108	40.03732075	1.70436281
DALYs (Disability-Adjusted Life Years)	Libya	21.081824	46.355242	4.909063214
DALYs (Disability-Adjusted Life Years)	Philippines	2.960623818	4.351326888	2.08357004
DALYs (Disability-Adjusted Life Years)	Philippines	1.76673275	2.840901936	0.903035353
DALYs (Disability-Adjusted Life Years)	Philippines	2.362704951	3.278304281	1.81724612
DALYs (Disability-Adjusted Life Years)	Iran (Islamic Republic of)	17.06674956	22.80830416	13.14669133
DALYs (Disability-Adjusted Life Years)	Iran (Islamic Republic of)	6.936655143	13.68327469	4.220618017
DALYs (Disability-Adjusted Life Years)	Iran (Islamic Republic of)	12.06773729	17.13099272	9.33455229
DALYs (Disability-Adjusted Life Years)	Puerto Rico	49.77763309	63.83677979	37.71767118
DALYs (Disability-Adjusted Life Years)	Puerto Rico	14.8169646	19.42452437	11.04073525
DALYs (Disability-Adjusted Life Years)	Puerto Rico	31.5185882	39.5601677	24.52084093
DALYs (Disability-Adjusted Life Years)	Afghanistan	24.68445606	56.38162759	6.162253672
DALYs (Disability-Adjusted Life Years)	Afghanistan	7.237124409	24.54873986	0.946969401

DALYs (Disability-Adjusted Life Years)	Afghanistan	15.52225345	34.92906362	4.254937287
DALYs (Disability-Adjusted Life Years)	India	12.76076355	17.47568177	7.894590796
DALYs (Disability-Adjusted Life Years)	India	8.28736371	12.46073776	4.746649271
DALYs (Disability-Adjusted Life Years)	India	10.54566137	13.55742782	7.783197024
DALYs (Disability-Adjusted Life Years)	Switzerland	16.07005322	19.67714317	12.96213745
DALYs (Disability-Adjusted Life Years)	Switzerland	6.532841114	8.141855443	5.117901861
DALYs (Disability-Adjusted Life Years)	Switzerland	11.36016348	13.37832511	9.554005233
DALYs (Disability-Adjusted Life Years)	Eritrea	19.84807609	38.21215751	8.783197778
DALYs (Disability-Adjusted Life Years)	Eritrea	9.984640693	21.4651698	3.606298854
DALYs (Disability-Adjusted Life Years)	Eritrea	14.76217247	25.8456195	7.564675706
DALYs (Disability-Adjusted Life Years)	Finland	32.26911357	39.16164083	26.20360793
DALYs (Disability-Adjusted Life Years)	Finland	12.66725068	15.7007423	9.869290838
DALYs (Disability-Adjusted Life Years)	Finland	22.53996445	26.49779653	18.99696051
DALYs (Disability-Adjusted Life Years)	Guatemala	11.35513232	14.87518575	8.515975053
DALYs (Disability-Adjusted Life Years)	Guatemala	6.538817579	8.476407474	4.924061487
DALYs (Disability-Adjusted Life Years)	Guatemala	8.781814086	10.91774455	6.974792341
DALYs (Disability-Adjusted Life Years)	North Macedonia	9.123543324	14.16121954	5.62975168
DALYs (Disability-Adjusted Life Years)	North Macedonia	5.446811283	9.44434626	2.767525131
DALYs (Disability-Adjusted Life Years)	North Macedonia	7.322427896	10.6028828	4.917840112
DALYs (Disability-Adjusted Life Years)	Guyana	63.17725984	89.07012422	43.07900756
DALYs (Disability-Adjusted Life Years)	Guyana	23.68494223	34.08962655	15.94824696
DALYs (Disability-Adjusted Life Years)	Guyana	42.87559371	58.4084602	30.4814355
DALYs (Disability-Adjusted Life Years)	Mauritania	10.03173631	20.18529202	4.20082619
DALYs (Disability-Adjusted Life Years)	Mauritania	10.87672909	22.6939079	4.52828752

DALYs (Disability-Adjusted Life Years)	Mauritania	10.44776751	18.3903649	5.507699433
DALYs (Disability-Adjusted Life Years)	Lesotho	21.25002302	41.22378053	9.370477796
DALYs (Disability-Adjusted Life Years)	Lesotho	10.13920953	22.79684863	3.329758639
DALYs (Disability-Adjusted Life Years)	Lesotho	15.25884482	27.00032291	7.540594618
DALYs (Disability-Adjusted Life Years)	Ethiopia	9.410197066	14.85267473	5.140767428
DALYs (Disability-Adjusted Life Years)	Ethiopia	6.864900452	12.7303652	2.901498212
DALYs (Disability-Adjusted Life Years)	Ethiopia	8.136252875	12.1267556	4.657655751
DALYs (Disability-Adjusted Life Years)	France	22.48662616	27.71022523	17.99005218
DALYs (Disability-Adjusted Life Years)	France	9.856676972	12.10120043	7.852239281
DALYs (Disability-Adjusted Life Years)	France	16.05422778	18.91413649	13.53635797
DALYs (Disability-Adjusted Life Years)	Kuwait	12.75331834	16.8729222	9.632193786
DALYs (Disability-Adjusted Life Years)	Kuwait	8.379783221	10.69229039	6.46115346
DALYs (Disability-Adjusted Life Years)	Kuwait	10.72102286	13.37680435	8.531003658
DALYs (Disability-Adjusted Life Years)	Haiti	31.88283754	60.48318896	13.01380711
DALYs (Disability-Adjusted Life Years)	Haiti	17.4649784	39.43540032	5.543166849
DALYs (Disability-Adjusted Life Years)	Haiti	24.3063065	42.99699857	11.44035531
DALYs (Disability-Adjusted Life Years)	Lebanon	35.79500905	56.99815421	21.05425743
DALYs (Disability-Adjusted Life Years)	Lebanon	20.96976465	36.58437426	10.9482325
DALYs (Disability-Adjusted Life Years)	Lebanon	28.16460194	41.72195046	18.22805452
DALYs (Disability-Adjusted Life Years)	Germany	33.53053939	40.46367518	27.29961042
DALYs (Disability-Adjusted Life Years)	Germany	12.08451586	14.82891149	9.648598046
DALYs (Disability-Adjusted Life Years)	Germany	22.89957518	26.77360287	19.37175226
DALYs (Disability-Adjusted Life Years)	Niger	6.25076922	14.47570972	1.629427294
DALYs (Disability-Adjusted Life Years)	Niger	8.077131933	17.96262801	2.616616832

DALYs (Disability-Adjusted Life Years)	Niger	7.16803562	13.58995404	2.67627245
DALYs (Disability-Adjusted Life Years)	Tuvalu	23.41223136	40.91519114	12.3238418
DALYs (Disability-Adjusted Life Years)	Tuvalu	32.07196936	63.06993889	14.9270452
DALYs (Disability-Adjusted Life Years)	Tuvalu	27.21004939	46.01321138	15.54120925
Prevalence	Chile	141.5420204	191.0004365	102.4522253
Prevalence	Chile	70.17625996	96.28168613	49.05021143
Prevalence	Chile	104.3480506	138.8838571	75.46068334
Prevalence	Nepal	17.54852389	25.03530129	11.83739567
Prevalence	Nepal	10.53476788	15.76354887	6.621569565
Prevalence	Nepal	13.81880712	19.79358942	9.273937948
Prevalence	Armenia	122.4905893	166.7122565	87.95565234
Prevalence	Armenia	65.12448191	91.06586298	45.56327355
Prevalence	Armenia	91.43029931	123.0604951	66.22442502
Prevalence	Thailand	16.07469564	24.27686246	10.40845676
Prevalence	Thailand	10.46940399	16.11955158	6.548282513
Prevalence	Thailand	13.1313075	19.77417327	8.458439314
Prevalence	Zimbabwe	17.03149878	23.73577742	11.76593767
Prevalence	Zimbabwe	6.756763422	10.2463817	4.126525135
Prevalence	Zimbabwe	11.36156134	16.01316736	7.790430417
Prevalence	Bermuda	272.2211642	383.0142066	189.6017649
Prevalence	Bermuda	101.8652819	139.2685552	71.91569009
Prevalence	Bermuda	184.9946891	251.3046074	133.690365
Prevalence	Poland	79.12778939	102.8203367	58.87188518
Prevalence	Poland	36.87208913	49.5233415	26.400425

Prevalence	Poland	57.54585694	75.0367136	42.50169715
Prevalence	Morocco	35.50269364	49.59495068	24.94691289
Prevalence	Morocco	24.77467411	34.75758194	16.9349047
Prevalence	Morocco	30.14421658	41.59311192	21.35560067
Prevalence	Greece	149.1649703	191.2931432	113.80457
Prevalence	Greece	61.95750579	82.79668206	45.03204286
Prevalence	Greece	104.2218441	134.3961495	79.71889997
Prevalence	Montenegro	206.068701	280.3261936	146.8583599
Prevalence	Montenegro	105.9346566	145.8911328	74.37156506
Prevalence	Montenegro	155.0966421	206.643203	112.0266672
Prevalence	Kenya	11.53940684	16.79762787	7.75514315
Prevalence	Kenya	6.73671939	10.08066822	4.238933322
Prevalence	Kenya	9.123282563	13.32036049	6.043100705
Prevalence	Nigeria	11.93943353	16.54989942	8.348434463
Prevalence	Nigeria	5.468097187	8.189202025	3.371809414
Prevalence	Nigeria	8.413842897	11.75080033	5.775345539
Prevalence	Mauritius	19.28985634	28.56010978	12.67654173
Prevalence	Mauritius	16.35761611	23.79350391	10.7188963
Prevalence	Mauritius	17.78616762	25.49463614	11.77808665
Prevalence	Timor-Leste	9.175616832	13.5785871	5.84575081
Prevalence	Timor-Leste	5.458485972	8.354069106	3.243127015
Prevalence	Timor-Leste	7.380807216	10.96160277	4.743321518
Prevalence	Jamaica	77.89342448	106.517831	55.88241221
Prevalence	Jamaica	35.6821967	49.57033415	24.60335834

Prevalence	Jamaica	56.65741985	77.00302112	40.84046708
Prevalence	Ireland	178.9222606	241.5484924	127.1870575
Prevalence	Ireland	69.7066281	96.94964461	48.70138045
Prevalence	Ireland	123.5633399	165.9918356	88.80612494
Prevalence	Cook Islands	24.49696448	36.04158716	16.20141287
Prevalence	Cook Islands	13.02419218	19.60631163	8.197689189
Prevalence	Cook Islands	18.78230533	27.4077528	12.49328662
Prevalence	Nicaragua	81.32282388	110.2677243	57.38348945
Prevalence	Nicaragua	42.93590174	59.12206306	30.08205335
Prevalence	Nicaragua	60.59807167	81.92818174	43.44156853
Prevalence	Eswatini	19.75246011	27.36571372	13.22047334
Prevalence	Eswatini	8.030943548	12.30633603	4.885033695
Prevalence	Eswatini	13.32684302	18.64043649	8.857083139
Prevalence	South Sudan	10.13439083	14.80222025	6.547856697
Prevalence	South Sudan	5.257976795	8.1986821	3.164864563
Prevalence	South Sudan	7.720091202	11.39680209	4.983705844
Prevalence	Madagascar	12.42645179	17.4505601	8.446039053
Prevalence	Madagascar	6.301690999	9.366196958	4.032493844
Prevalence	Madagascar	9.29030425	13.08518983	6.267600012
Prevalence	Sri Lanka	23.74839231	33.2080053	16.03980284
Prevalence	Sri Lanka	18.64864049	26.82875041	12.49611354
Prevalence	Sri Lanka	21.05035097	28.91388938	14.76931841
Prevalence	Iceland	279.5803653	381.5641585	197.2387383
Prevalence	Iceland	92.66490165	126.5678982	65.47284456

Prevalence	Iceland	186.7927618	253.760963	134.0290275
Prevalence	Benin	13.54178645	18.35945237	9.398808556
Prevalence	Benin	4.996240771	7.614119071	3.004066044
Prevalence	Benin	9.145883857	12.49964145	6.440318535
Prevalence	Venezuela (Bolivarian Republic of)	108.0466996	146.04273	78.80419586
Prevalence	Venezuela (Bolivarian Republic of)	53.97863742	75.30800024	37.46683755
Prevalence	Venezuela (Bolivarian Republic of)	80.01191136	106.7614415	59.17376173
Prevalence	Saint Lucia	101.9404859	138.6988763	73.43821026
Prevalence	Saint Lucia	41.08208557	57.35761889	28.61877397
Prevalence	Saint Lucia	71.29977531	95.65559188	52.39437822
Prevalence	Saint Vincent and the Grenadines	91.82885951	123.973352	65.7404553
Prevalence	Saint Vincent and the Grenadines	38.71795746	54.41212136	26.84378829
Prevalence	Saint Vincent and the Grenadines	66.52314661	89.56084761	48.2204342
Prevalence	Palestine	28.66757276	39.28632132	19.75897487
Prevalence	Palestine	20.71964293	29.27669029	14.14870269
Prevalence	Palestine	24.7790691	33.96976698	17.25701481
Prevalence	Sao Tome and Principe	16.22576993	22.20298716	11.56995318
Prevalence	Sao Tome and Principe	6.723311546	10.12958421	4.123705172
Prevalence	Sao Tome and Principe	11.53029115	15.96437211	8.195060052
Prevalence	Georgia	141.6455575	192.3002402	102.452407
Prevalence	Georgia	76.85281613	105.2749999	53.68375826
Prevalence	Georgia	106.9169874	144.2163541	77.68783475
Prevalence	Solomon Islands	14.67192037	21.61028671	9.538040355
Prevalence	Solomon Islands	7.593988673	11.7497008	4.582669073

Prevalence	Solomon Islands	11.20136201	16.69295774	7.309123894
Prevalence	Republic of Korea	126.0528133	171.9733978	90.45521373
Prevalence	Republic of Korea	66.50814146	94.73265384	45.88849424
Prevalence	Republic of Korea	96.79308232	132.5545832	68.99809429
Prevalence	Oman	42.70118557	57.39400439	30.50440379
Prevalence	Oman	27.536634	38.92227015	18.74944628
Prevalence	Oman	36.96384778	49.86266786	26.47652742
Prevalence	Canada	161.1772198	215.5814383	115.667522
Prevalence	Canada	83.01599628	115.6591836	58.79247592
Prevalence	Canada	121.3768936	161.991212	87.75103567
Prevalence	Malawi	11.29550665	16.67508377	7.330080841
Prevalence	Malawi	5.733022547	8.825349305	3.49887672
Prevalence	Malawi	8.40910453	12.41576331	5.52408033
Prevalence	Uruguay	150.7126247	198.6348271	109.9150865
Prevalence	Uruguay	72.12292086	100.6270629	50.68902183
Prevalence	Uruguay	109.3218379	144.9342859	81.39578546
Prevalence	Angola	11.79128148	16.97375827	7.781453283
Prevalence	Angola	6.205710795	9.322982276	3.831761348
Prevalence	Angola	8.808376512	12.70742344	5.809195059
Prevalence	Senegal	12.75686868	17.57977666	8.966069259
Prevalence	Senegal	4.784395561	7.268996942	2.822527321
Prevalence	Senegal	8.618374778	11.96873928	6.023728102
Prevalence	Guam	28.10790634	39.68045836	19.1394851
Prevalence	Guam	20.7626541	29.47088259	14.2166945

Prevalence	Guam	24.44745447	34.44100244	17.11747851
Prevalence	Azerbaijan	110.1063916	150.1754877	77.8475081
Prevalence	Azerbaijan	54.42166956	75.50394439	37.00755285
Prevalence	Azerbaijan	80.85872076	109.8175862	57.5094347
Prevalence	United States Virgin Islands	149.4134818	204.5207394	104.1720142
Prevalence	United States Virgin Islands	69.51558179	93.24301131	48.9943285
Prevalence	United States Virgin Islands	108.4535241	143.6734554	78.06005503
Prevalence	Saudi Arabia	41.58704977	57.82002815	28.58667746
Prevalence	Saudi Arabia	32.28281173	45.12355503	22.23711968
Prevalence	Saudi Arabia	37.83897181	52.42396736	26.38008775
Prevalence	Brunei Darussalam	128.5832134	174.7463331	93.10942812
Prevalence	Brunei Darussalam	69.88939492	99.47035433	48.48454558
Prevalence	Brunei Darussalam	100.1185614	136.9871595	72.40974587
Prevalence	Pakistan	17.42343382	24.73963752	11.82625015
Prevalence	Pakistan	9.325480127	13.78050991	5.964639125
Prevalence	Pakistan	13.54626488	19.38223129	9.162219778
Prevalence	Panama	98.16418627	132.7993098	70.69985898
Prevalence	Panama	49.80756079	69.30885903	34.96575388
Prevalence	Panama	73.81320762	98.89469643	53.81727701
Prevalence	Cambodia	9.819720992	14.48117296	6.079829739
Prevalence	Cambodia	5.912098196	8.917393162	3.545639171
Prevalence	Cambodia	7.679298913	11.27366245	4.798171053
Prevalence	Italy	195.2168296	247.9932727	150.7786666
Prevalence	Italy	78.76371845	101.6448426	59.45885041

Prevalence	Italy	135.7202279	173.1489597	104.6334547
Prevalence	Kazakhstan	115.8045337	158.468529	81.41753953
Prevalence	Kazakhstan	58.28881365	81.33010631	39.72745315
Prevalence	Kazakhstan	84.91749323	115.9212032	59.8178824
Prevalence	Singapore	130.1556372	175.0999386	95.48376447
Prevalence	Singapore	71.10601809	101.3248767	48.79306017
Prevalence	Singapore	102.0344084	138.4625257	74.54632713
Prevalence	Central African Republic	8.566304812	12.40356383	5.574557041
Prevalence	Central African Republic	4.216554361	6.492390244	2.540445549
Prevalence	Central African Republic	6.342219737	9.229210261	4.106855295
Prevalence	Cameroon	17.09093461	23.30870219	12.13438251
Prevalence	Cameroon	5.862985789	8.907445032	3.547707217
Prevalence	Cameroon	11.43728581	15.81651463	8.102853865
Prevalence	Burkina Faso	12.9678769	17.5285698	9.151118307
Prevalence	Burkina Faso	4.562485794	6.972707047	2.682701769
Prevalence	Burkina Faso	8.606847477	11.92711963	6.000112307
Prevalence	Sierra Leone	11.93838156	16.25209769	8.410824965
Prevalence	Sierra Leone	4.494861231	6.854342256	2.708098319
Prevalence	Sierra Leone	8.332469641	11.5247868	5.788459258
Prevalence	United States of America	174.7736652	222.1131533	133.5264282
Prevalence	United States of America	94.87062566	122.1232847	71.52057369
Prevalence	United States of America	133.857918	170.6589992	102.054489
Prevalence	Albania	175.1210638	235.6715784	127.1772668
Prevalence	Albania	90.53260993	126.775537	62.99371384

Prevalence	Albania	131.6393297	177.3459386	95.90132316
Prevalence	Rwanda	10.37871766	15.29375558	6.688348962
Prevalence	Rwanda	6.414468788	9.847852697	4.008735012
Prevalence	Rwanda	8.22739096	12.21423393	5.342771286
Prevalence	Slovakia	187.8190072	247.6016736	140.0696288
Prevalence	Slovakia	83.8887241	120.1845699	59.56041566
Prevalence	Slovakia	134.9965173	180.4617401	101.9405514
Prevalence	Japan	141.0218531	185.3470335	104.9753758
Prevalence	Japan	67.51103329	92.89751852	48.32398748
Prevalence	Japan	104.4312461	138.674153	77.211089
Prevalence	Brazil	89.5634281	120.7174587	63.73625274
Prevalence	Brazil	54.64534287	74.67778106	38.75180684
Prevalence	Brazil	71.082791	95.81030889	50.61537018
Prevalence	Kyrgyzstan	88.31753739	120.8551177	62.37265289
Prevalence	Kyrgyzstan	45.38335308	62.80924962	31.18858994
Prevalence	Kyrgyzstan	65.47600272	89.06100478	46.57246835
Prevalence	Trinidad and Tobago	88.90210195	123.0933171	61.95033629
Prevalence	Trinidad and Tobago	41.64956921	58.01221818	29.41889959
Prevalence	Trinidad and Tobago	65.29231344	89.64491527	46.44236432
Prevalence	Israel	94.65494315	127.2311743	68.5848894
Prevalence	Israel	33.40202185	47.16741869	23.246111
Prevalence	Israel	63.31891012	84.55283873	46.35259084
Prevalence	Suriname	77.48036505	105.5711012	56.47853418
Prevalence	Suriname	37.83468474	52.87778179	26.27829214

Prevalence	Suriname	57.18461208	77.43712981	41.91330731
Prevalence	Greenland	144.9304261	195.2348816	103.0772204
Prevalence	Greenland	68.63431696	94.47812433	47.86659285
Prevalence	Greenland	110.089638	146.0139028	80.38496954
Prevalence	Slovenia	389.283658	554.7724842	272.0839694
Prevalence	Slovenia	182.6925516	258.2804711	131.813157
Prevalence	Slovenia	287.1959714	396.5534438	205.6698574
Prevalence	Fiji	17.52195524	25.33751024	11.47514612
Prevalence	Fiji	8.118651353	12.4153668	4.85697069
Prevalence	Fiji	12.83060849	18.81263367	8.318843581
Prevalence	Luxembourg	244.4356492	337.1282173	167.9661345
Prevalence	Luxembourg	93.62060263	125.9111989	65.99946667
Prevalence	Luxembourg	170.9016254	230.3493257	121.8374152
Prevalence	Nauru	20.65495349	29.55457015	13.91098792
Prevalence	Nauru	10.78157499	16.08262453	6.915209972
Prevalence	Nauru	15.38762496	22.15643273	10.37080412
Prevalence	Romania	471.7838878	632.082598	332.0930863
Prevalence	Romania	288.107685	435.0542033	195.4859299
Prevalence	Romania	378.6115585	505.168533	265.395777
Prevalence	Malta	130.0417539	171.4803932	94.64571756
Prevalence	Malta	53.68504501	75.58626891	36.96953594
Prevalence	Malta	92.13717081	121.0848033	67.80590747
Prevalence	Syrian Arab Republic	44.8309583	61.3481583	31.72249427
Prevalence	Syrian Arab Republic	46.37274814	62.3959895	33.83013167

Prevalence	Syrian Arab Republic	45.65776493	60.96570376	33.28538352
Prevalence	Antigua and Barbuda	92.3098745	127.0565178	65.16240638
Prevalence	Antigua and Barbuda	42.06890599	57.28911306	29.06946492
Prevalence	Antigua and Barbuda	66.19766742	90.05303875	47.66187527
Prevalence	Viet Nam	13.64844719	20.08952821	8.693060475
Prevalence	Viet Nam	8.15842781	12.34520161	4.917921524
Prevalence	Viet Nam	10.76783344	15.82799306	6.916319248
Prevalence	Seychelles	21.89698147	31.03351922	14.52340426
Prevalence	Seychelles	13.69447075	20.6944085	8.849190647
Prevalence	Seychelles	17.92539088	25.6544719	12.06212545
Prevalence	Bolivia (Plurinational State of)	86.7392003	119.1485095	60.53331547
Prevalence	Bolivia (Plurinational State of)	42.75704898	60.23140881	29.6040188
Prevalence	Bolivia (Plurinational State of)	64.2358466	88.33322867	45.77146044
Prevalence	Uzbekistan	108.8807555	148.514012	76.99186688
Prevalence	Uzbekistan	53.23799474	75.16534857	36.60093356
Prevalence	Uzbekistan	79.79242909	108.4610543	56.61586475
Prevalence	New Zealand	133.7709789	176.3898532	100.1920163
Prevalence	New Zealand	55.7996675	74.18126055	40.74633178
Prevalence	New Zealand	93.85512971	123.5074155	69.80430909
Prevalence	Democratic Republic of the Congo	11.31487062	16.31931197	7.570510562
Prevalence	Democratic Republic of the Congo	5.814757423	8.695435435	3.605174965
Prevalence	Democratic Republic of the Congo	8.54936361	12.38467152	5.686140224
Prevalence	Democratic People's Republic of Korea	13.4961335	20.1742772	8.831342865
Prevalence	Democratic People's Republic of Korea	8.15021317	12.29362316	4.973882397

Prevalence	Democratic People's Republic of Korea	10.74968205	15.80738233	6.943840489
Prevalence	Kiribati	17.6396484	25.54263174	11.74312875
Prevalence	Kiribati	7.630896144	11.41640031	4.678385874
Prevalence	Kiribati	12.35007951	17.91960952	8.198041308
Prevalence	Taiwan (Province of China)	21.82583024	32.16893957	14.38652758
Prevalence	Taiwan (Province of China)	16.13131582	25.07428468	10.13608318
Prevalence	Taiwan (Province of China)	18.91062161	28.28405879	12.48938538
Prevalence	Equatorial Guinea	17.42358351	24.51531016	11.50275145
Prevalence	Equatorial Guinea	8.406863565	12.60192004	5.172451261
Prevalence	Equatorial Guinea	12.469111	17.66789109	8.27069894
Prevalence	Croatia	368.2457933	489.6598861	260.1759322
Prevalence	Croatia	155.7095588	207.0827621	109.9236992
Prevalence	Croatia	259.4649312	336.6083323	190.408061
Prevalence	Qatar	53.59708613	74.35628019	37.30900839
Prevalence	Qatar	41.48812694	59.22830367	28.33127816
Prevalence	Qatar	50.33010903	69.8566571	35.37817115
Prevalence	Algeria	39.34520062	53.70406056	28.162713
Prevalence	Algeria	29.01662375	41.32229676	20.14135484
Prevalence	Algeria	34.22420414	46.73474197	24.35301201
Prevalence	Bahamas	92.57126262	123.4304721	65.91581012
Prevalence	Bahamas	46.80099947	65.24522007	33.69933638
Prevalence	Bahamas	68.350593	90.61180909	49.94589951
Prevalence	Tajikistan	66.06892738	90.18348862	46.68714884
Prevalence	Tajikistan	33.0783991	46.19459614	22.85622851

Prevalence	Tajikistan	49.27577856	66.91264831	35.22626464
Prevalence	Niue	23.48334153	34.45901147	15.4062402
Prevalence	Niue	12.3452492	18.56727681	7.810247123
Prevalence	Niue	18.11539951	26.42237798	12.02058237
Prevalence	Somalia	7.94197891	11.91670854	5.156600145
Prevalence	Somalia	3.857795493	5.978378056	2.223567039
Prevalence	Somalia	5.741586593	8.533495475	3.692625944
Prevalence	China	19.76851416	28.19873735	13.36045524
Prevalence	China	12.06702679	17.57738084	7.836487689
Prevalence	China	15.94887143	22.83517532	10.72594628
Prevalence	Serbia	210.3944906	290.6797715	147.8252518
Prevalence	Serbia	116.7695143	163.0465294	82.97998242
Prevalence	Serbia	162.7713696	217.1450108	118.3182656
Prevalence	Mozambique	12.21098187	17.69411228	8.213024984
Prevalence	Mozambique	5.73831981	8.702320754	3.5144558
Prevalence	Mozambique	8.767147113	12.77253117	5.846184756
Prevalence	Tonga	23.48315137	34.47906172	15.66257554
Prevalence	Tonga	11.476841	17.25332118	7.239480146
Prevalence	Tonga	17.41667797	25.46054322	11.58455162
Prevalence	Gambia	14.3372476	19.51565985	10.0882418
Prevalence	Gambia	5.116581169	7.878348084	3.089260171
Prevalence	Gambia	9.776914458	13.36774117	6.85552961
Prevalence	Tunisia	42.04136641	57.56280508	29.90396711
Prevalence	Tunisia	32.03749386	44.43872647	22.45625658

Prevalence	Tunisia	36.97818016	50.22792416	26.6277709
Prevalence	Coted'Ivoire	14.38618479	19.36008877	10.19135106
Prevalence	Coted'Ivoire	5.401447213	8.292419508	3.244996071
Prevalence	Coted'Ivoire	10.24463484	13.86994505	7.262778287
Prevalence	Togo	13.89120633	18.75321527	9.849374901
Prevalence	Togo	4.857533988	7.328279039	2.905503697
Prevalence	Togo	9.127513721	12.37316445	6.450197836
Prevalence	Barbados	113.3695482	159.1194042	80.21900541
Prevalence	Barbados	51.32076173	70.58954989	36.11939648
Prevalence	Barbados	80.44338713	110.7328389	57.82858859
Prevalence	Ecuador	101.9272305	124.2254321	83.51765258
Prevalence	Ecuador	59.63301003	74.81763144	46.92861396
Prevalence	Ecuador	80.16412778	97.35249244	65.04506419
Prevalence	Estonia	375.1074662	544.1409951	262.5018119
Prevalence	Estonia	111.4836456	158.841588	75.2749747
Prevalence	Estonia	235.2472573	327.266417	168.4004709
Prevalence	Turkmenistan	103.1621199	140.6227706	72.03626518
Prevalence	Turkmenistan	51.1402792	71.67467912	34.5578506
Prevalence	Turkmenistan	76.07951593	103.1873172	53.6287705
Prevalence	Andorra	151.7270246	206.6279762	109.2618786
Prevalence	Andorra	67.10683473	93.73631448	47.05647551
Prevalence	Andorra	111.3436375	152.6360539	80.45767891
Prevalence	Spain	217.1417014	290.0011025	156.2051194
Prevalence	Spain	77.51851771	105.8129369	55.59446355

Prevalence	Spain	146.2914939	192.9275462	106.2139387
Prevalence	Australia	123.5428834	167.6804514	87.75952304
Prevalence	Australia	55.62384272	80.40701243	38.41829747
Prevalence	Australia	88.79845577	120.4189804	63.83178181
Prevalence	Paraguay	113.437502	154.2947584	82.22519889
Prevalence	Paraguay	65.91628981	90.61666695	46.59747855
Prevalence	Paraguay	89.55958682	121.2573807	65.96080459
Prevalence	Russian Federation	226.0461866	303.1211049	162.3893884
Prevalence	Russian Federation	75.506901	103.4768075	52.94510439
Prevalence	Russian Federation	143.0504895	192.5904401	102.096128
Prevalence	Northern Mariana Islands	32.80606755	46.52922075	22.40740082
Prevalence	Northern Mariana Islands	24.67685085	33.88393352	17.59288114
Prevalence	Northern Mariana Islands	29.05778457	40.26013176	20.56658008
Prevalence	Latvia	288.9769211	407.4170689	200.6745451
Prevalence	Latvia	108.9985077	155.4475015	73.26901676
Prevalence	Latvia	191.4844177	260.2944738	136.230562
Prevalence	Zambia	12.52479349	17.70619259	8.285001105
Prevalence	Zambia	6.608749144	10.08677229	4.134636055
Prevalence	Zambia	9.61078496	13.74829256	6.369513888
Prevalence	Congo	13.25948703	19.26125697	8.749060461
Prevalence	Congo	6.897218789	10.6598303	4.203850098
Prevalence	Congo	10.1443803	14.82398851	6.687599326
Prevalence	Cabo Verde	19.13189914	25.74165707	13.68241572
Prevalence	Cabo Verde	8.168049562	12.21807487	5.16276903

Prevalence	Cabo Verde	13.64941903	18.65433669	9.766324707
Prevalence	Samoa	21.06423983	30.42648766	14.02369393
Prevalence	Samoa	11.83957695	17.8384166	7.474300636
Prevalence	Samoa	16.62365597	23.89653855	11.10129276
Prevalence	Micronesia (Federated States of)	20.86817756	29.95220656	14.05557011
Prevalence	Micronesia (Federated States of)	10.26241543	15.1473975	6.423789756
Prevalence	Micronesia (Federated States of)	15.58272393	22.22770435	10.56163272
Prevalence	Czechia	373.6392586	515.8918233	259.3864028
Prevalence	Czechia	153.7584052	209.2016254	108.201444
Prevalence	Czechia	263.9709835	357.0499628	190.7605149
Prevalence	Netherlands	75.35736184	102.331064	54.06904596
Prevalence	Netherlands	27.52017537	38.25955909	18.95211528
Prevalence	Netherlands	51.42344531	68.85245469	36.8234045
Prevalence	Colombia	139.0353175	183.4102901	102.2436396
Prevalence	Colombia	67.27227926	91.58177545	47.31016629
Prevalence	Colombia	100.4396972	129.5724338	73.97151166
Prevalence	Mongolia	92.75661588	126.0836942	65.02912502
Prevalence	Mongolia	42.71089747	59.37316859	29.24024886
Prevalence	Mongolia	65.62441744	88.30651893	46.23381582
Prevalence	Burundi	8.51198567	12.48063108	5.482697868
Prevalence	Burundi	4.653263324	7.1715164	2.731867371
Prevalence	Burundi	6.692771447	9.80374528	4.311847208
Prevalence	Yemen	22.85283286	31.24175826	16.10089186
Prevalence	Yemen	15.89220418	22.49357187	10.79686599

Prevalence	Yemen	19.31728308	26.20796912	13.76932954
Prevalence	Lithuania	224.506678	305.1666593	158.4011664
Prevalence	Lithuania	82.85062398	120.6884032	57.58621525
Prevalence	Lithuania	147.5838449	200.7972976	106.5512893
Prevalence	Belize	84.52347057	115.6693274	60.59605281
Prevalence	Belize	35.22389321	49.71828293	24.06276751
Prevalence	Belize	59.92803918	81.43233478	43.38426153
Prevalence	Monaco	157.015447	215.3212973	114.0835098
Prevalence	Monaco	60.42854947	86.79169219	41.54729527
Prevalence	Monaco	108.3143672	149.0382935	78.64542289
Prevalence	Norway	163.6739345	219.6280383	117.8659988
Prevalence	Norway	59.26326635	81.84329514	41.54974499
Prevalence	Norway	112.5019334	151.7387967	80.8030169
Prevalence	Myanmar	10.80521845	16.00398894	6.879279359
Prevalence	Myanmar	6.115340914	9.344402897	3.639789541
Prevalence	Myanmar	8.244037749	12.20275505	5.205798197
Prevalence	Lao People's Democratic Republic	9.740100088	14.75998099	6.284275444
Prevalence	Lao People's Democratic Republic	5.652898767	8.749837797	3.313110833
Prevalence	Lao People's Democratic Republic	7.675242129	11.65934288	4.878777883
Prevalence	Jordan	40.25878739	55.67983085	27.84711096
Prevalence	Jordan	28.52707137	40.99669112	19.20952142
Prevalence	Jordan	34.91050912	48.4198966	24.43141199
Prevalence	Turkey	42.69219606	58.54646048	29.78765498
Prevalence	Turkey	32.27819961	45.34193855	22.24030865

Prevalence	Turkey	37.46474018	51.38442268	26.43215623
Prevalence	Sudan	29.56037215	41.56716954	20.6368195
Prevalence	Sudan	19.07821657	26.60288167	13.01396239
Prevalence	Sudan	24.57832714	33.83371493	17.27848399
Prevalence	Indonesia	12.07291905	17.49551539	7.948303425
Prevalence	Indonesia	6.866496732	10.27315279	4.232503378
Prevalence	Indonesia	9.465992492	13.79854151	6.110391357
Prevalence	Maldives	19.37635051	27.7941511	12.86003845
Prevalence	Maldives	12.7378387	19.36776621	8.237781889
Prevalence	Maldives	16.63903758	24.27436623	11.12869756
Prevalence	Bulgaria	170.972036	226.3699488	124.5702662
Prevalence	Bulgaria	87.06418772	118.2010595	61.18580442
Prevalence	Bulgaria	128.6635097	168.5655178	94.06756739
Prevalence	Papua New Guinea	11.61684989	16.70832884	7.533697052
Prevalence	Papua New Guinea	6.204636212	9.547128662	3.731996443
Prevalence	Papua New Guinea	9.086118339	13.11278914	5.82587728
Prevalence	Gabon	17.77500291	25.79004868	11.86475726
Prevalence	Gabon	8.840104529	13.13699851	5.560968573
Prevalence	Gabon	13.27413565	18.92643416	8.864326813
Prevalence	Portugal	91.76866919	122.6146265	66.8947228
Prevalence	Portugal	36.13483102	50.61458975	24.7584585
Prevalence	Portugal	62.49015981	82.6686482	45.6072179
Prevalence	Saint Kitts and Nevis	91.1554559	125.2742629	65.12342721
Prevalence	Saint Kitts and Nevis	45.53817108	62.03187848	31.69320055

Prevalence	Saint Kitts and Nevis	68.45802679	91.43158246	49.6253264
Prevalence	Dominica	106.8100366	146.0650411	76.68713824
Prevalence	Dominica	34.5752413	48.39991622	23.56420925
Prevalence	Dominica	72.84780868	99.08094203	52.9167211
Prevalence	Iraq	30.46810684	42.24602613	20.87374484
Prevalence	Iraq	19.63677363	28.22155162	12.96342526
Prevalence	Iraq	25.1913951	35.10645419	17.23229063
Prevalence	Dominican Republic	81.24004817	109.08516	58.73341461
Prevalence	Dominican Republic	34.534523	48.40831519	23.99382731
Prevalence	Dominican Republic	57.6909246	77.169951	42.11933166
Prevalence	Comoros	10.99310146	16.22125486	7.146813241
Prevalence	Comoros	5.956166556	9.063653473	3.635821978
Prevalence	Comoros	8.421546517	12.43875661	5.424447289
Prevalence	Peru	89.90522378	119.6005121	64.96405065
Prevalence	Peru	45.36954638	62.79059249	31.9341862
Prevalence	Peru	67.09006182	89.48342488	48.01631541
Prevalence	Chad	11.3999973	15.46154546	8.058653407
Prevalence	Chad	3.908251915	6.098364821	2.307995436
Prevalence	Chad	7.763580642	10.82287759	5.462307684
Prevalence	Djibouti	11.74154015	16.90058254	7.747838613
Prevalence	Djibouti	6.00800918	9.107398067	3.640561226
Prevalence	Djibouti	9.164852605	13.20447559	6.066597477
Prevalence	Marshall Islands	17.84372271	25.71409635	12.03625776
Prevalence	Marshall Islands	8.697853628	13.24002158	5.376639253

Prevalence	Marshall Islands	13.24661989	19.20275465	8.895551947
Prevalence	Guinea-Bissau	12.37205189	16.9900943	8.519281049
Prevalence	Guinea-Bissau	4.116085756	6.294809562	2.427177737
Prevalence	Guinea-Bissau	7.972285471	11.07791632	5.57222914
Prevalence	San Marino	150.7972998	204.294567	108.9255736
Prevalence	San Marino	68.82395767	95.37843982	47.76117217
Prevalence	San Marino	107.6256814	146.1212018	77.76142258
Prevalence	Belarus	176.7241885	240.5832259	125.1851509
Prevalence	Belarus	69.59463327	99.89562428	48.02806603
Prevalence	Belarus	118.1045691	160.4016032	83.70860232
Prevalence	Bangladesh	17.18816726	24.92878721	11.51045332
Prevalence	Bangladesh	9.717359205	14.7569591	6.093795669
Prevalence	Bangladesh	13.40392568	19.5288178	8.951038413
Prevalence	Austria	373.0485335	519.5185709	266.0442593
Prevalence	Austria	142.6345553	201.3114946	97.01735501
Prevalence	Austria	256.683205	354.0539777	182.4691956
Prevalence	Malaysia	17.99249744	26.56032453	11.53424664
Prevalence	Malaysia	11.36819945	17.10809298	7.157014634
Prevalence	Malaysia	14.71311113	21.62348255	9.55408334
Prevalence	Denmark	213.5469079	289.4800162	152.9969365
Prevalence	Denmark	79.02741064	109.6530523	55.46931956
Prevalence	Denmark	146.3261176	193.766253	106.862947
Prevalence	United Arab Emirates	56.4259213	78.29974136	39.8224177
Prevalence	United Arab Emirates	40.49638755	57.59365551	28.3833584

Prevalence	United Arab Emirates	52.7155951	73.16845432	37.62474317
Prevalence	Cyprus	134.5670036	181.54607	96.99012832
Prevalence	Cyprus	57.37724078	80.30882841	39.48691061
Prevalence	Cyprus	94.68551314	126.8343698	69.80742759
Prevalence	Namibia	17.51490617	24.9270891	11.54617883
Prevalence	Namibia	8.267918957	12.39492555	5.121837133
Prevalence	Namibia	12.50966701	17.82986274	8.354793888
Prevalence	Bosnia and Herzegovina	214.4318301	290.0752745	154.6640044
Prevalence	Bosnia and Herzegovina	105.9929565	146.8030551	74.14930667
Prevalence	Bosnia and Herzegovina	158.8885129	213.2898855	115.166433
Prevalence	Ukraine	161.6471597	221.1646679	112.3302237
Prevalence	Ukraine	56.61434864	80.904821	38.49553499
Prevalence	Ukraine	103.5358317	141.6745914	72.38311457
Prevalence	Ghana	15.79710224	21.29571529	11.06836116
Prevalence	Ghana	5.839960618	8.756343543	3.522701577
Prevalence	Ghana	10.41061912	14.27921759	7.256445591
Prevalence	Hungary	373.4005897	510.2074219	256.3816059
Prevalence	Hungary	170.0346075	231.019926	116.8724784
Prevalence	Hungary	267.1308181	348.1970763	196.4114465
Prevalence	Bahrain	43.81277579	59.8533557	30.86901477
Prevalence	Bahrain	33.64585809	48.51902829	22.79336926
Prevalence	Bahrain	40.22100985	55.46404398	28.38221347
Prevalence	United Republic of Tanzania	12.95365149	18.66024866	8.547961472
Prevalence	United Republic of Tanzania	6.904687897	10.25453395	4.256632995

Prevalence	United Republic of Tanzania	9.829835049	14.04420416	6.553466895
Prevalence	Cuba	122.8094048	170.4029414	86.89790465
Prevalence	Cuba	54.05091388	72.86405503	39.30325839
Prevalence	Cuba	87.47928802	116.7146776	64.69417624
Prevalence	Mali	10.84921168	15.06861401	7.232855174
Prevalence	Mali	3.887967626	6.009298071	2.250113489
Prevalence	Mali	7.407532771	10.38364905	4.932458832
Prevalence	Liberia	14.0019566	18.78997925	9.988744797
Prevalence	Liberia	5.232466978	7.940181541	3.071817935
Prevalence	Liberia	9.850833075	13.42348575	6.916259782
Prevalence	Bhutan	19.91096871	28.41164265	13.26369084
Prevalence	Bhutan	11.4617429	17.29748854	7.154614768
Prevalence	Bhutan	15.86092339	22.78828127	10.55327891
Prevalence	Botswana	18.97127943	27.20639412	12.57252732
Prevalence	Botswana	8.875510261	13.22811007	5.539310144
Prevalence	Botswana	13.63144485	19.33088523	9.065893209
Prevalence	American Samoa	28.78645029	40.89522215	19.58999129
Prevalence	American Samoa	12.64175603	19.09328659	7.814664658
Prevalence	American Samoa	20.91876158	29.95480638	14.24515805
Prevalence	Tokelau	19.33725496	28.07300918	12.72782066
Prevalence	Tokelau	11.15852947	16.48750672	6.996297223
Prevalence	Tokelau	15.31859004	22.55345287	10.09179898
Prevalence	Honduras	73.8009407	99.39412985	52.37136191
Prevalence	Honduras	36.40714467	51.70142541	25.06314275

Prevalence	Honduras	53.97046199	72.2064802	38.95993622
Prevalence	Belgium	211.1153237	291.6235145	150.9431183
Prevalence	Belgium	43.92707237	62.76884969	30.64696036
Prevalence	Belgium	127.3950579	173.8503818	92.19927037
Prevalence	United Kingdom	141.1516143	184.4287473	105.3823614
Prevalence	United Kingdom	67.66251495	90.98500697	48.98890306
Prevalence	United Kingdom	103.8490335	137.0536158	76.6733051
Prevalence	South Africa	23.45478664	32.52430961	16.09093694
Prevalence	South Africa	9.660797001	13.97203418	6.297474099
Prevalence	South Africa	15.99196568	22.36247259	10.93577722
Prevalence	Sweden	217.5467224	292.9032485	156.5122558
Prevalence	Sweden	100.0619414	138.566743	69.92761752
Prevalence	Sweden	159.4133544	213.0611312	113.9763994
Prevalence	Uganda	11.17586959	16.05479498	7.330477878
Prevalence	Uganda	6.185487546	9.380924393	3.86454046
Prevalence	Uganda	8.521334015	12.31341714	5.662578744
Prevalence	Egypt	42.54777095	59.09037333	30.4679059
Prevalence	Egypt	29.71110116	42.93511366	20.05898947
Prevalence	Egypt	36.44701932	50.82413765	25.88870885
Prevalence	Mexico	106.9524681	145.2370779	76.28080715
Prevalence	Mexico	55.42111842	76.47273683	39.02799612
Prevalence	Mexico	79.69835576	108.8348613	56.85617238
Prevalence	Vanuatu	16.16503697	23.18910624	10.79046289
Prevalence	Vanuatu	7.845045068	11.97464494	4.629782587

Prevalence	Vanuatu	11.98041008	17.44009933	7.920036146
Prevalence	Argentina	130.2592525	177.2984239	91.05375845
Prevalence	Argentina	62.38709043	86.31554016	44.12909704
Prevalence	Argentina	94.96500636	124.2071596	69.27278485
Prevalence	Palau	25.52176877	36.76464606	17.00947982
Prevalence	Palau	12.78695941	19.15321164	8.203440387
Prevalence	Palau	19.39066484	28.24279358	13.04210522
Prevalence	Costa Rica	169.9861911	225.9209821	120.7631807
Prevalence	Costa Rica	71.64688821	96.32248016	50.339612
Prevalence	Costa Rica	117.5624374	152.25532	86.25072555
Prevalence	El Salvador	82.09893481	110.6875052	57.4906702
Prevalence	El Salvador	43.6622234	60.63391569	30.33215758
Prevalence	El Salvador	59.67081514	80.89711122	42.63549223
Prevalence	Republic of Moldova	166.6658352	226.2901761	118.2935662
Prevalence	Republic of Moldova	65.01209747	91.34794808	44.90302958
Prevalence	Republic of Moldova	111.3330916	151.9470526	79.65192905
Prevalence	Guinea	13.59785688	18.36155685	9.677438882
Prevalence	Guinea	4.426166937	6.692980339	2.584935845
Prevalence	Guinea	8.854636658	12.17968217	6.281042364
Prevalence	Grenada	118.1036776	168.6269946	85.13964977
Prevalence	Grenada	31.7107687	45.73467094	21.63759702
Prevalence	Grenada	76.55523325	108.1133079	56.33761886
Prevalence	Libya	38.77003285	52.91380603	28.31451391
Prevalence	Libya	29.31590343	40.7050825	20.46455555

Prevalence	Libya	34.16877347	46.3472076	25.04260108
Prevalence	Philippines	10.20373742	14.88197379	6.598047154
Prevalence	Philippines	6.716521216	9.975041538	4.172909259
Prevalence	Philippines	8.444580215	12.41629109	5.418792594
Prevalence	Iran (Islamic Republic of)	39.65863417	53.50288636	28.47243739
Prevalence	Iran (Islamic Republic of)	28.84128253	39.23585467	20.40669137
Prevalence	Iran (Islamic Republic of)	34.27668877	46.26591395	24.62391194
Prevalence	Puerto Rico	136.3462376	190.5452176	94.48457667
Prevalence	Puerto Rico	61.91399382	89.40266216	42.65435221
Prevalence	Puerto Rico	96.54612412	132.7425683	68.94961553
Prevalence	Afghanistan	21.69958177	29.82086459	15.20714466
Prevalence	Afghanistan	12.23171888	17.74135246	7.998594504
Prevalence	Afghanistan	16.47873903	22.78850672	11.60172314
Prevalence	India	16.03175647	22.88499121	10.7828116
Prevalence	India	9.693908586	14.24302092	6.351963344
Prevalence	India	12.86689873	18.56359425	8.697449751
Prevalence	Switzerland	225.8916778	313.6442826	159.6369424
Prevalence	Switzerland	84.64923413	118.3619312	58.58140363
Prevalence	Switzerland	155.8671549	212.333759	111.5808004
Prevalence	Eritrea	8.401848072	12.26442328	5.463885043
Prevalence	Eritrea	4.863494255	7.496554395	2.934981607
Prevalence	Eritrea	6.536351397	9.538839275	4.271350199
Prevalence	Finland	273.989093	387.5668444	192.2387659
Prevalence	Finland	99.37881839	138.2797216	69.24987832

Prevalence	Finland	186.5082089	253.885175	134.7463392
Prevalence	Guatemala	64.21450544	88.12107182	45.14271425
Prevalence	Guatemala	32.76864101	46.62934495	22.36031807
Prevalence	Guatemala	47.3567065	65.23589869	33.61599172
Prevalence	North Macedonia	186.7659067	251.3428227	136.683134
Prevalence	North Macedonia	97.72121715	131.8624055	68.20926401
Prevalence	North Macedonia	143.1536636	190.3913851	104.8388675
Prevalence	Guyana	114.7022782	149.0244292	84.95405311
Prevalence	Guyana	40.77258485	55.25390777	28.05879438
Prevalence	Guyana	76.54612465	98.62372561	57.23949622
Prevalence	Mauritania	16.55514083	22.12598238	11.58925978
Prevalence	Mauritania	6.220312164	9.53598049	3.749123287
Prevalence	Mauritania	11.21270034	15.33483585	7.803271398
Prevalence	Lesotho	15.73762697	21.89959377	10.74935913
Prevalence	Lesotho	6.9751643	10.52208536	4.235384363
Prevalence	Lesotho	10.79637592	15.21428259	7.296683968
Prevalence	Ethiopia	8.305592377	12.26458331	5.412143728
Prevalence	Ethiopia	4.837380042	7.385906554	2.887618862
Prevalence	Ethiopia	6.580768819	9.741029779	4.2042358
Prevalence	France	164.3939448	227.6359773	115.156742
Prevalence	France	58.50521522	83.94540185	40.57344782
Prevalence	France	109.9621869	152.0606817	77.68494791
Prevalence	Kuwait	54.17901294	75.4951209	37.4559903
Prevalence	Kuwait	41.84224125	58.40082494	29.49176606

Prevalence	Kuwait	48.86378804	67.14099647	34.42211099
Prevalence	Haiti	52.16404115	70.69058935	37.02482413
Prevalence	Haiti	20.94038331	28.94387724	14.28536683
Prevalence	Haiti	35.75553065	48.20912493	25.5197752
Prevalence	Lebanon	52.52729607	71.00682819	38.50298813
Prevalence	Lebanon	34.46917755	47.85597874	24.2369647
Prevalence	Lebanon	42.79652676	57.57542441	31.71745435
Prevalence	Germany	251.5402484	341.32721	179.529728
Prevalence	Germany	78.7085492	110.1782039	55.14794868
Prevalence	Germany	165.2523283	219.613222	120.0179024
Prevalence	Niger	10.71942937	14.82550817	7.39418834
Prevalence	Niger	3.937422267	6.057115449	2.322248043
Prevalence	Niger	7.173824021	10.05214978	4.955482045
Prevalence	Tuvalu	18.74216357	26.46456459	12.54119116
Prevalence	Tuvalu	9.617240659	14.35546438	5.970572355
Prevalence	Tuvalu	14.13826315	20.06108741	9.43358316
Incidence	Chile	16.65928996	23.80446117	11.10947644
Incidence	Chile	9.380801918	13.43828418	6.085067689
Incidence	Chile	12.8891458	18.19846758	8.761778274
Incidence	Nepal	2.243583431	3.333658992	1.397528963
Incidence	Nepal	1.445867812	2.229412464	0.848176637
Incidence	Nepal	1.816508665	2.71168724	1.149674849
Incidence	Armenia	13.7233917	20.0741224	8.892588634
Incidence	Armenia	7.9574072	11.82055959	4.965593363

Incidence	Armenia	10.62054701	15.26249116	6.977831297
Incidence	Thailand	2.178101423	3.439470996	1.309123086
Incidence	Thailand	1.46636921	2.331932694	0.846709505
Incidence	Thailand	1.805897433	2.822341143	1.092010837
Incidence	Zimbabwe	1.864673398	2.777059984	1.171362039
Incidence	Zimbabwe	0.841339585	1.339268621	0.464405908
Incidence	Zimbabwe	1.32076003	1.961552228	0.83116836
Incidence	Bermuda	36.01795033	53.04682071	23.40384116
Incidence	Bermuda	12.57947861	18.23103191	8.174569367
Incidence	Bermuda	24.07621764	34.11236917	16.45460045
Incidence	Poland	9.677791685	13.19136748	6.714536901
Incidence	Poland	4.899308244	7.019546758	3.230690226
Incidence	Poland	7.242993663	9.995254849	4.942069942
Incidence	Morocco	4.068091279	6.028748039	2.622856581
Incidence	Morocco	2.728068882	4.095963207	1.678912241
Incidence	Morocco	3.397484474	4.984119111	2.220864058
Incidence	Greece	19.07945175	25.75900338	13.37216017
Incidence	Greece	9.293587038	13.15889064	6.19387127
Incidence	Greece	14.04094488	18.94920423	9.980621969
Incidence	Montenegro	22.49078959	32.91019732	14.5551118
Incidence	Montenegro	13.118661	19.28402136	8.418081176
Incidence	Montenegro	17.73515917	25.20694264	11.73240084
Incidence	Kenya	1.435051393	2.190170624	0.877060737
Incidence	Kenya	0.907381653	1.409406553	0.525227107

Incidence	Kenya	1.170179838	1.79225198	0.708659965
Incidence	Nigeria	1.354257541	1.939601594	0.869148889
Incidence	Nigeria	0.668490711	1.058920943	0.367966569
Incidence	Nigeria	0.989572072	1.461774468	0.614004993
Incidence	Mauritius	2.431267794	3.748216968	1.463807181
Incidence	Mauritius	1.891204586	2.959449466	1.112917623
Incidence	Mauritius	2.156330937	3.25887207	1.31088425
Incidence	Timor-Leste	1.206643427	1.844668563	0.727308551
Incidence	Timor-Leste	0.758594748	1.197157822	0.417600451
Incidence	Timor-Leste	0.990418519	1.503871451	0.595607971
Incidence	Jamaica	8.641233169	12.61254065	5.517073924
Incidence	Jamaica	3.880577916	5.916042169	2.402107602
Incidence	Jamaica	6.24720322	9.130450151	4.045672002
Incidence	Ireland	23.1232499	33.63788068	15.15467943
Incidence	Ireland	9.92966494	14.44093477	6.358544139
Incidence	Ireland	16.44066134	23.26085417	11.09614441
Incidence	Cook Islands	3.251349554	5.092199383	2.000699899
Incidence	Cook Islands	1.776254549	2.782290136	1.04236014
Incidence	Cook Islands	2.508373736	3.855045523	1.564379019
Incidence	Nicaragua	10.10283489	14.55526433	6.486443514
Incidence	Nicaragua	4.9883143	7.360233807	3.12575822
Incidence	Nicaragua	7.350293741	10.44569009	4.785735092
Incidence	Eswatini	2.191842249	3.233091477	1.360761327
Incidence	Eswatini	1.034434278	1.664795514	0.588837074

Incidence	Eswatini	1.586939041	2.356874074	0.984822204
Incidence	South Sudan	1.299090187	1.950665918	0.764302133
Incidence	South Sudan	0.750134695	1.20988223	0.414374573
Incidence	South Sudan	1.019473062	1.540431089	0.611737528
Incidence	Madagascar	1.55224766	2.268537112	0.970203634
Incidence	Madagascar	0.911856528	1.387554074	0.541334768
Incidence	Madagascar	1.223153021	1.779034758	0.768974553
Incidence	Sri Lanka	3.287410561	4.790518996	2.112140295
Incidence	Sri Lanka	2.619721076	3.860622759	1.697277273
Incidence	Sri Lanka	2.93619387	4.174614854	1.946159007
Incidence	Iceland	38.10031922	54.63968472	24.92599359
Incidence	Iceland	13.93547381	20.35336892	9.0073073
Incidence	Iceland	26.09983996	37.07475109	17.70771735
Incidence	Benin	1.579239002	2.215756132	1.034365924
Incidence	Benin	0.62679725	1.010179591	0.333172417
Incidence	Benin	1.08666958	1.535344633	0.70374953
Incidence	Venezuela (Bolivarian Republic of)	14.48361058	20.45181517	10.11432316
Incidence	Venezuela (Bolivarian Republic of)	6.680637659	9.670036727	4.348878537
Incidence	Venezuela (Bolivarian Republic of)	10.45528292	14.42035875	7.388263982
Incidence	Saint Lucia	12.70914117	18.08138934	8.320958585
Incidence	Saint Lucia	4.825041802	7.130651458	3.08659244
Incidence	Saint Lucia	8.746728037	12.13474083	5.959916537
Incidence	Saint Vincent and the Grenadines	11.50341977	16.41946119	7.686729734
Incidence	Saint Vincent and the Grenadines	4.621266841	6.867970205	2.934421244

Incidence	Saint Vincent and the Grenadines	8.217416155	11.5400945	5.638386215
Incidence	Palestine	2.95382365	4.350128223	1.842404143
Incidence	Palestine	1.936290317	2.928600582	1.151284912
Incidence	Palestine	2.456497993	3.637572124	1.554344879
Incidence	Sao Tome and Principe	1.653786566	2.360087256	1.072417174
Incidence	Sao Tome and Principe	0.793477597	1.266116484	0.423709169
Incidence	Sao Tome and Principe	1.233513285	1.782977769	0.789516027
Incidence	Georgia	17.01620961	24.18061963	11.05300431
Incidence	Georgia	10.6316593	15.06367018	7.016869492
Incidence	Georgia	13.64988442	19.17648166	9.323754949
Incidence	Solomon Islands	2.079970863	3.189032025	1.289100278
Incidence	Solomon Islands	1.140568754	1.788496055	0.664934597
Incidence	Solomon Islands	1.618123648	2.459063057	1.015083844
Incidence	Republic of Korea	12.86416643	18.98230742	8.237561417
Incidence	Republic of Korea	9.033593232	13.20202811	5.802994329
Incidence	Republic of Korea	11.00880502	15.82940768	7.200739373
Incidence	Oman	5.074220665	7.21166681	3.35296085
Incidence	Oman	2.910557037	4.442665412	1.762289435
Incidence	Oman	4.281255574	6.149393418	2.844507954
Incidence	Canada	18.92197136	27.22859262	12.43320716
Incidence	Canada	9.816600298	14.56982527	6.341328364
Incidence	Canada	14.28976654	20.28564657	9.681636787
Incidence	Malawi	1.470096121	2.215561957	0.888764742
Incidence	Malawi	0.815501736	1.292032357	0.456517847

Incidence	Malawi	1.132919643	1.722992689	0.694519099
Incidence	Uruguay	20.31777484	28.03792842	13.13932898
Incidence	Uruguay	10.54632495	15.54910575	6.789138705
Incidence	Uruguay	15.18164511	20.81597428	10.41110799
Incidence	Angola	1.599829582	2.367606017	0.98503753
Incidence	Angola	0.931503274	1.444779446	0.53881465
Incidence	Angola	1.244771133	1.826681697	0.781697785
Incidence	Senegal	1.442109817	2.041978806	0.940290183
Incidence	Senegal	0.59286477	0.960916146	0.29156059
Incidence	Senegal	0.999991474	1.448826574	0.623065947
Incidence	Guam	3.704975635	5.575265182	2.297960394
Incidence	Guam	2.423806898	3.71178202	1.47978428
Incidence	Guam	3.066578807	4.621310325	1.942501299
Incidence	Azerbaijan	12.1536311	17.88839453	7.709667675
Incidence	Azerbaijan	6.279628244	9.385651879	3.882632359
Incidence	Azerbaijan	9.085174254	13.29793631	5.809422997
Incidence	United States Virgin Islands	19.22871193	27.7331534	12.08001353
Incidence	United States Virgin Islands	8.932627585	12.59203446	5.893389265
Incidence	United States Virgin Islands	13.96080894	19.40719841	9.360107216
Incidence	Saudi Arabia	4.710283292	6.98574228	2.932395532
Incidence	Saudi Arabia	3.547101124	5.378225608	2.167493391
Incidence	Saudi Arabia	4.244553428	6.288825422	2.6725689
Incidence	Brunei Darussalam	15.11858336	21.996778	9.94869231
Incidence	Brunei Darussalam	10.4030075	15.18567039	6.739550042

Incidence	Brunei Darussalam	12.85374925	18.20683049	8.638844953
Incidence	Pakistan	2.229833309	3.364903241	1.381268491
Incidence	Pakistan	1.318295037	2.048596503	0.798384835
Incidence	Pakistan	1.789356373	2.714495956	1.114807757
Incidence	Panama	12.08341026	16.95402157	7.947536727
Incidence	Panama	5.706152848	8.403753981	3.644191822
Incidence	Panama	8.876961191	12.49690196	5.984147771
Incidence	Cambodia	1.357301525	2.076915625	0.798815583
Incidence	Cambodia	0.830717274	1.305139435	0.459605332
Incidence	Cambodia	1.071422784	1.637377853	0.630245321
Incidence	Italy	26.93006201	35.59710899	19.49349304
Incidence	Italy	13.59619615	17.84321146	9.960821814
Incidence	Italy	20.12849952	26.48463476	14.6976793
Incidence	Kazakhstan	12.98968671	19.44012298	8.314711227
Incidence	Kazakhstan	6.987348501	10.35203749	4.354188955
Incidence	Kazakhstan	9.796294392	14.33834958	6.321789888
Incidence	Singapore	12.38570009	18.21591665	8.114163253
Incidence	Singapore	8.976319266	13.49795198	5.718207606
Incidence	Singapore	10.74049329	15.5962838	7.166584077
Incidence	Central African Republic	1.31013477	1.875019425	0.834784801
Incidence	Central African Republic	0.673060189	1.049077949	0.385489441
Incidence	Central African Republic	0.985131981	1.424902776	0.621371551
Incidence	Cameroon	2.009629681	2.806701372	1.377179977
Incidence	Cameroon	0.75644147	1.215515885	0.405062977

Incidence	Cameroon	1.380597849	1.948916383	0.923717745
Incidence	Burkina Faso	1.603083113	2.215666734	1.071396046
Incidence	Burkina Faso	0.590474134	0.961750739	0.306418529
Incidence	Burkina Faso	1.07785824	1.540844945	0.699851404
Incidence	Sierra Leone	1.432791834	2.011827455	0.946885581
Incidence	Sierra Leone	0.574888257	0.928279219	0.294323078
Incidence	Sierra Leone	1.011540769	1.457145655	0.643340288
Incidence	United States of America	22.56632355	30.89006333	15.54417829
Incidence	United States of America	12.44166318	17.20290775	8.5163309
Incidence	United States of America	17.38384614	23.89074404	11.97366082
Incidence	Albania	19.20376006	27.74384833	12.54138054
Incidence	Albania	11.35869265	16.78140846	7.327447809
Incidence	Albania	15.17936397	21.73383176	10.12903119
Incidence	Rwanda	1.345815606	2.027075104	0.796287165
Incidence	Rwanda	0.948787424	1.468911009	0.554437411
Incidence	Rwanda	1.13518764	1.70819483	0.691518286
Incidence	Slovakia	21.61488353	30.07283766	14.82106532
Incidence	Slovakia	10.68206387	15.82537134	7.056221992
Incidence	Slovakia	16.08577164	22.39280303	11.37159122
Incidence	Japan	15.32934766	21.91845704	10.09446919
Incidence	Japan	9.960851655	14.28241286	6.507407139
Incidence	Japan	12.66832663	17.93626553	8.333971183
Incidence	Brazil	11.56064757	16.6494197	7.676541005
Incidence	Brazil	6.579746967	9.677025316	4.236121887

Incidence	Brazil	8.935365354	12.93338459	5.866350029
Incidence	Kyrgyzstan	9.501398268	13.99405862	6.048379531
Incidence	Kyrgyzstan	5.210693291	7.634961701	3.272420122
Incidence	Kyrgyzstan	7.237043189	10.46413235	4.628261044
Incidence	Trinidad and Tobago	10.42041283	15.230453	6.53303173
Incidence	Trinidad and Tobago	4.762820007	7.110488	3.037706545
Incidence	Trinidad and Tobago	7.601485492	11.16235673	4.908466271
Incidence	Israel	11.91732711	16.87727882	7.854669388
Incidence	Israel	4.55911046	6.845134913	2.853072955
Incidence	Israel	8.153587817	11.53247024	5.500801919
Incidence	Suriname	9.471495548	13.54497594	6.401479519
Incidence	Suriname	4.539107132	6.761825017	2.895441001
Incidence	Suriname	6.949622176	9.775811912	4.692448679
Incidence	Greenland	18.55264444	26.8416822	12.030401
Incidence	Greenland	9.119261966	13.26398605	5.854601994
Incidence	Greenland	14.23443907	20.20308401	9.498354778
Incidence	Slovenia	58.58556346	83.28211572	40.77839825
Incidence	Slovenia	34.64843509	47.0842969	25.76224058
Incidence	Slovenia	46.7862496	64.12120581	34.16062093
Incidence	Fiji	2.420593176	3.658657483	1.496242959
Incidence	Fiji	1.142161812	1.80700331	0.646400783
Incidence	Fiji	1.785217505	2.713976932	1.092300538
Incidence	Luxembourg	33.50554384	47.85204446	21.55532101
Incidence	Luxembourg	14.86542678	21.3433563	9.659078154

Incidence	Luxembourg	24.42864096	34.27009727	16.18851685
Incidence	Nauru	2.875222878	4.333050117	1.817208746
Incidence	Nauru	1.481637652	2.315890611	0.886730695
Incidence	Nauru	2.143094999	3.222819442	1.369689125
Incidence	Romania	56.10921875	78.58460173	35.58326403
Incidence	Romania	39.56280079	62.23629467	25.28317921
Incidence	Romania	47.82118847	66.01124617	31.22711015
Incidence	Malta	15.96596269	22.71939486	10.5461149
Incidence	Malta	7.140470138	10.63513253	4.583898547
Incidence	Malta	11.58804306	16.1807117	7.806312061
Incidence	Syrian Arab Republic	4.966577855	7.272461069	3.10343462
Incidence	Syrian Arab Republic	4.955780864	7.265598354	3.230863706
Incidence	Syrian Arab Republic	4.968705745	7.1722396	3.264626139
Incidence	Antigua and Barbuda	11.50552909	16.57456286	7.407602879
Incidence	Antigua and Barbuda	4.953929104	7.040069687	3.188493859
Incidence	Antigua and Barbuda	8.099732899	11.47850707	5.461990358
Incidence	Viet Nam	1.809095961	2.811379004	1.10041161
Incidence	Viet Nam	1.050849644	1.663360081	0.581412248
Incidence	Viet Nam	1.414554489	2.172648867	0.855623109
Incidence	Seychelles	3.054113179	4.485308815	1.899152096
Incidence	Seychelles	1.950961397	3.033721974	1.194790267
Incidence	Seychelles	2.524879423	3.709543479	1.60606132
Incidence	Bolivia (Plurinational State of)	11.11803654	15.9014869	7.17078025
Incidence	Bolivia (Plurinational State of)	5.399617363	7.977525122	3.401731619

Incidence	Bolivia (Plurinational State of)	8.198275351	11.62244242	5.416789911
Incidence	Uzbekistan	12.63936968	18.34508223	8.276661447
Incidence	Uzbekistan	6.680827164	10.01556207	4.157150202
Incidence	Uzbekistan	9.537489022	13.85903507	6.28354154
Incidence	New Zealand	19.87338662	27.82280332	13.38473465
Incidence	New Zealand	8.603737956	12.26051466	5.674203802
Incidence	New Zealand	14.10539796	19.74453218	9.574833864
Incidence	Democratic Republic of the Congo	1.507467708	2.227615318	0.93243706
Incidence	Democratic Republic of the Congo	0.865034973	1.31941393	0.513723715
Incidence	Democratic Republic of the Congo	1.187836042	1.723159911	0.749074739
Incidence	Democratic People's Republic of Korea	1.728545043	2.689680352	1.057844503
Incidence	Democratic People's Republic of Korea	1.080826808	1.675267241	0.607779112
Incidence	Democratic People's Republic of Korea	1.399658322	2.159860078	0.851034631
Incidence	Kiribati	2.508855715	3.737480313	1.583752453
Incidence	Kiribati	1.150764408	1.750439181	0.68446898
Incidence	Kiribati	1.792165681	2.643582881	1.141585606
Incidence	Taiwan (Province of China)	3.150966821	4.801353861	1.941426455
Incidence	Taiwan (Province of China)	2.418777348	3.836030637	1.413004106
Incidence	Taiwan (Province of China)	2.776721896	4.266243001	1.719696506
Incidence	Equatorial Guinea	2.258241429	3.305628753	1.401570588
Incidence	Equatorial Guinea	1.255714428	1.947197462	0.733786052
Incidence	Equatorial Guinea	1.722788031	2.519897674	1.0781865
Incidence	Croatia	49.84503518	70.134453	32.80314776
Incidence	Croatia	22.81775742	31.12572192	15.20959582

Incidence	Croatia	36.05690232	48.51055158	25.21845809
Incidence	Qatar	6.05918156	9.053924722	3.826763129
Incidence	Qatar	4.847111824	7.52925227	2.895797817
Incidence	Qatar	5.746632767	8.589336461	3.657381489
Incidence	Algeria	4.381821189	6.442992887	2.80320147
Incidence	Algeria	3.158550086	4.782909613	1.93357183
Incidence	Algeria	3.774806243	5.506722941	2.417169518
Incidence	Bahamas	11.24452834	15.74943559	7.305581634
Incidence	Bahamas	5.547109281	8.121459925	3.664865951
Incidence	Bahamas	8.24009825	11.37907624	5.587799808
Incidence	Tajikistan	6.948959367	10.24882773	4.413562567
Incidence	Tajikistan	3.747397022	5.586175935	2.351244252
Incidence	Tajikistan	5.323943131	7.824707481	3.420391432
Incidence	Niue	3.224164735	4.945427463	1.968582104
Incidence	Niue	1.713612671	2.674219649	1.020424989
Incidence	Niue	2.491748041	3.787466487	1.553714795
Incidence	Somalia	1.125875554	1.714396144	0.688450043
Incidence	Somalia	0.583115223	0.930010509	0.314423543
Incidence	Somalia	0.840902096	1.266681598	0.51133846
Incidence	China	2.427370378	3.708085337	1.488759528
Incidence	China	1.54188395	2.374449294	0.916590355
Incidence	China	1.989281076	3.04451822	1.205270988
Incidence	Serbia	26.6150723	38.49159856	17.42336195
Incidence	Serbia	17.10668393	23.7795549	11.61604402

Incidence	Serbia	21.80422506	30.05722249	15.12505531
Incidence	Mozambique	1.665118658	2.441831707	1.058001722
Incidence	Mozambique	0.834147081	1.310903186	0.476502807
Incidence	Mozambique	1.222422322	1.795943974	0.787085688
Incidence	Tonga	3.178032761	4.847862638	1.969697007
Incidence	Tonga	1.594048568	2.499545456	0.938731368
Incidence	Tonga	2.375858093	3.636054898	1.495558607
Incidence	Gambia	1.67503192	2.367475191	1.103803007
Incidence	Gambia	0.643157273	1.055097833	0.329216067
Incidence	Gambia	1.15615542	1.676330872	0.737267568
Incidence	Tunisia	4.585963041	6.686896926	2.936519339
Incidence	Tunisia	3.295779948	4.929438564	2.007718497
Incidence	Tunisia	3.931338134	5.713859195	2.531916074
Incidence	Coted'Ivoire	1.712130421	2.348505702	1.153501075
Incidence	Coted'Ivoire	0.672543045	1.086897703	0.343471989
Incidence	Coted'Ivoire	1.234499587	1.729786531	0.806171136
Incidence	Togo	1.677397695	2.29936444	1.146108813
Incidence	Togo	0.609415556	0.977884131	0.313226382
Incidence	Togo	1.117754757	1.574936704	0.735320312
Incidence	Barbados	14.09642262	20.97837594	9.100369969
Incidence	Barbados	6.159934486	8.76306227	4.09699294
Incidence	Barbados	9.895305822	14.04416059	6.640553795
Incidence	Ecuador	13.5344903	17.09341668	10.29413859
Incidence	Ecuador	7.59028219	10.02089993	5.627923888

Incidence	Ecuador	10.47756582	13.10149128	8.051567836
Incidence	Estonia	54.58546718	81.46964465	36.103022
Incidence	Estonia	19.71205933	27.83356713	13.09991372
Incidence	Estonia	36.27688342	51.72695402	25.46521356
Incidence	Turkmenistan	11.28533772	16.44504819	7.141804993
Incidence	Turkmenistan	6.067355757	9.024569052	3.757868462
Incidence	Turkmenistan	8.608126942	12.52359967	5.518819206
Incidence	Andorra	18.40886685	26.71049294	11.80493662
Incidence	Andorra	9.525871267	13.85403712	6.277150575
Incidence	Andorra	14.16703009	20.37123581	9.31806464
Incidence	Spain	28.54164707	39.82541518	19.20639126
Incidence	Spain	12.10345765	17.24519803	8.084479016
Incidence	Spain	20.2202585	27.49574263	13.88702846
Incidence	Australia	16.60447579	23.88590026	11.01933253
Incidence	Australia	7.839107684	11.87169441	4.981218158
Incidence	Australia	12.12203863	17.45938023	8.059443921
Incidence	Paraguay	14.51903765	20.93650001	9.794071192
Incidence	Paraguay	8.355855188	11.80695883	5.435311058
Incidence	Paraguay	11.42677619	16.24316615	7.764437538
Incidence	Russian Federation	28.44275478	40.66185932	18.98991816
Incidence	Russian Federation	10.44170941	15.22018102	6.87050613
Incidence	Russian Federation	18.62613342	26.69090873	12.4309396
Incidence	Northern Mariana Islands	4.454959059	6.715472942	2.771205016
Incidence	Northern Mariana Islands	2.968687076	4.478082971	1.876965849

Incidence	Northern Mariana Islands	3.741834986	5.57468223	2.414408444
Incidence	Latvia	38.74127535	56.74512575	24.21652838
Incidence	Latvia	16.85537354	24.43833703	11.06134355
Incidence	Latvia	27.00588373	37.6786182	17.98550512
Incidence	Zambia	1.662141421	2.431012166	1.030117981
Incidence	Zambia	0.990896734	1.536259221	0.581312019
Incidence	Zambia	1.333758988	1.959443327	0.832253631
Incidence	Congo	1.777905173	2.652505312	1.089889162
Incidence	Congo	1.05185377	1.623077117	0.617082867
Incidence	Congo	1.42146519	2.092112901	0.878904274
Incidence	Cabo Verde	2.053698888	2.857996885	1.375012608
Incidence	Cabo Verde	0.90265875	1.453564484	0.486614902
Incidence	Cabo Verde	1.503952058	2.133715237	0.981443432
Incidence	Samoa	2.838776509	4.276418866	1.768026255
Incidence	Samoa	1.626105713	2.502649798	0.982197802
Incidence	Samoa	2.259342824	3.418814996	1.427238495
Incidence	Micronesia (Federated States of)	2.935038764	4.437803966	1.850180722
Incidence	Micronesia (Federated States of)	1.521048956	2.303141135	0.908230496
Incidence	Micronesia (Federated States of)	2.231453006	3.310643824	1.409137438
Incidence	Czechia	48.00091101	67.68828017	30.48959095
Incidence	Czechia	22.01673431	30.71037382	14.97100403
Incidence	Czechia	35.08790739	48.62201048	24.04236734
Incidence	Netherlands	10.04623281	14.76782156	6.470537283
Incidence	Netherlands	3.829288011	5.48978521	2.47167566

Incidence	Netherlands	6.936109367	9.888162406	4.605346514
Incidence	Colombia	18.53885988	25.3822869	13.0578427
Incidence	Colombia	8.560354139	12.32081261	5.577516585
Incidence	Colombia	13.19307569	17.628996	9.260658519
Incidence	Mongolia	10.58339088	15.33014933	6.62139184
Incidence	Mongolia	5.044814031	7.522810873	3.130256367
Incidence	Mongolia	7.611317451	10.99092391	4.878876761
Incidence	Burundi	1.122888772	1.694839511	0.669129937
Incidence	Burundi	0.677435729	1.079285865	0.373975797
Incidence	Burundi	0.910091581	1.386577929	0.539110428
Incidence	Yemen	2.724066245	3.894112674	1.793846945
Incidence	Yemen	1.809934838	2.688869224	1.117214809
Incidence	Yemen	2.260503742	3.243746448	1.478216568
Incidence	Lithuania	29.48818492	41.61611909	19.86223417
Incidence	Lithuania	12.19276967	18.20387202	8.118700802
Incidence	Lithuania	20.17615374	27.93801865	14.15252665
Incidence	Belize	10.27443691	14.93170295	6.820697786
Incidence	Belize	3.97109286	5.970391274	2.475164083
Incidence	Belize	7.119877228	10.3773231	4.768918312
Incidence	Monaco	18.7323495	27.32440348	12.30199265
Incidence	Monaco	8.014557744	12.23112038	5.055237354
Incidence	Monaco	13.33188582	19.54379707	8.999749389
Incidence	Norway	23.19252293	33.24196808	15.14854232
Incidence	Norway	9.026622852	13.41485715	5.540491525

Incidence	Norway	16.24976399	23.48933929	10.50356412
Incidence	Myanmar	1.521935945	2.318622541	0.909802699
Incidence	Myanmar	0.874274195	1.372167486	0.49014509
Incidence	Myanmar	1.170198331	1.78913026	0.704969151
Incidence	Lao People's Democratic Republic	1.358379895	2.125887448	0.806288944
Incidence	Lao People's Democratic Republic	0.814293826	1.299065014	0.450335328
Incidence	Lao People's Democratic Republic	1.083796488	1.680710671	0.643470598
Incidence	Jordan	4.241146255	6.359070615	2.630630598
Incidence	Jordan	2.91735011	4.488457489	1.741773056
Incidence	Jordan	3.640193689	5.427095877	2.271935884
Incidence	Turkey	4.755690819	6.9739371	2.991020119
Incidence	Turkey	3.432838052	5.136513898	2.091260749
Incidence	Turkey	4.094115167	6.008731665	2.598536813
Incidence	Sudan	3.403359353	5.004953456	2.197205589
Incidence	Sudan	2.138803774	3.168694688	1.310368694
Incidence	Sudan	2.795336036	4.094284851	1.811590814
Incidence	Indonesia	1.635588169	2.500309127	0.998727091
Incidence	Indonesia	0.955471385	1.479398856	0.554249107
Incidence	Indonesia	1.295429552	1.989552658	0.785403407
Incidence	Maldives	2.601076405	3.994667159	1.567366886
Incidence	Maldives	1.92150603	2.997997548	1.163014412
Incidence	Maldives	2.328678349	3.51610007	1.448951713
Incidence	Bulgaria	18.1553939	25.84735736	11.68764166
Incidence	Bulgaria	10.46669267	15.02210915	6.554752554

Incidence	Bulgaria	14.30651279	19.71606977	9.495019764
Incidence	Papua New Guinea	1.585127862	2.361759893	0.964499294
Incidence	Papua New Guinea	0.888785929	1.398170552	0.499669254
Incidence	Papua New Guinea	1.256335074	1.891516166	0.765631108
Incidence	Gabon	2.31562179	3.449141891	1.4480826
Incidence	Gabon	1.292076293	1.9807976	0.768459963
Incidence	Gabon	1.794454581	2.622482054	1.144411635
Incidence	Portugal	11.97899932	16.77264524	8.292058892
Incidence	Portugal	5.168960245	7.592079306	3.308473467
Incidence	Portugal	8.398344229	11.71860734	5.915855328
Incidence	Saint Kitts and Nevis	11.54332274	16.86679608	7.498891
Incidence	Saint Kitts and Nevis	5.731739097	8.084619214	3.800892047
Incidence	Saint Kitts and Nevis	8.670049293	12.30614828	5.898234859
Incidence	Dominica	13.85778525	19.8771821	9.423199531
Incidence	Dominica	3.945327794	5.846121541	2.413268054
Incidence	Dominica	9.177426533	13.11976416	6.292298208
Incidence	Iraq	3.381516042	5.010097037	2.105756512
Incidence	Iraq	2.017219161	3.115339568	1.200479761
Incidence	Iraq	2.722982873	4.023249501	1.716910161
Incidence	Dominican Republic	9.974414678	14.07367558	6.723847898
Incidence	Dominican Republic	3.941379964	5.932887013	2.454018077
Incidence	Dominican Republic	6.938957824	9.776189986	4.750163938
Incidence	Comoros	1.33908107	2.045646198	0.785403002
Incidence	Comoros	0.835948567	1.298694207	0.476072351

Incidence	Comoros	1.085999544	1.652942141	0.651446193
Incidence	Peru	10.95359333	15.39909924	7.288758057
Incidence	Peru	5.321462079	7.817852536	3.337073895
Incidence	Peru	8.071165403	11.35040537	5.382225715
Incidence	Chad	1.433794061	1.995926462	0.951391559
Incidence	Chad	0.510352908	0.847716971	0.258610593
Incidence	Chad	0.968554463	1.388586239	0.625345069
Incidence	Djibouti	1.451325753	2.188971409	0.884534778
Incidence	Djibouti	0.832139339	1.299901117	0.469354731
Incidence	Djibouti	1.168354314	1.747539241	0.718648937
Incidence	Marshall Islands	2.490405994	3.698007163	1.574752674
Incidence	Marshall Islands	1.265644668	1.956722859	0.746904584
Incidence	Marshall Islands	1.877669639	2.807354973	1.205480289
Incidence	Guinea-Bissau	1.6790529	2.315683458	1.154163823
Incidence	Guinea-Bissau	0.555627717	0.906060552	0.286996992
Incidence	Guinea-Bissau	1.077250167	1.523472751	0.718696843
Incidence	San Marino	17.60142901	25.65674515	11.40228645
Incidence	San Marino	10.13396096	14.6812264	6.433062116
Incidence	San Marino	13.65755909	19.65077326	9.020712552
Incidence	Belarus	21.29070656	31.11945379	13.87033312
Incidence	Belarus	9.282737441	13.93888013	6.025906236
Incidence	Belarus	14.78542994	21.34350323	9.699487916
Incidence	Bangladesh	2.093918692	3.17723618	1.289052146
Incidence	Bangladesh	1.28407988	2.019495166	0.752492008

Incidence	Bangladesh	1.68040581	2.571816868	1.04690465
Incidence	Austria	53.85368859	78.77428119	34.80422456
Incidence	Austria	24.49206032	36.7768813	14.71570347
Incidence	Austria	39.04889356	56.54828671	25.64980656
Incidence	Malaysia	2.452732477	3.755752566	1.498347698
Incidence	Malaysia	1.636258244	2.49656523	0.983637514
Incidence	Malaysia	2.051382982	3.111431068	1.270638749
Incidence	Denmark	29.42529913	42.08611918	19.31483353
Incidence	Denmark	12.46764961	18.42661721	7.659132132
Incidence	Denmark	20.95777833	28.96098717	14.32354571
Incidence	United Arab Emirates	6.148616305	9.25711829	3.828266095
Incidence	United Arab Emirates	4.280105849	6.60217446	2.610865989
Incidence	United Arab Emirates	5.718105458	8.593167305	3.630320033
Incidence	Cyprus	19.67478513	28.2473295	13.02186041
Incidence	Cyprus	9.148731506	13.50930305	5.78768815
Incidence	Cyprus	14.23794093	20.20141839	9.671405552
Incidence	Namibia	1.867120524	2.836942922	1.130363782
Incidence	Namibia	1.001502902	1.576311691	0.55496154
Incidence	Namibia	1.412990031	2.150584347	0.847564979
Incidence	Bosnia and Herzegovina	25.64749729	36.63899963	17.20549493
Incidence	Bosnia and Herzegovina	13.9904806	20.25464943	9.017383039
Incidence	Bosnia and Herzegovina	19.69800306	27.67299055	13.43628085
Incidence	Ukraine	19.18193594	28.46728225	12.02836875
Incidence	Ukraine	7.264504222	10.90825787	4.594120524

Incidence	Ukraine	12.65822601	18.48100721	8.078879165
Incidence	Ghana	1.81965945	2.500207473	1.213464938
Incidence	Ghana	0.739076948	1.1810381	0.394125394
Incidence	Ghana	1.245289638	1.764242773	0.8038686
Incidence	Hungary	48.87304684	70.2648454	31.9062755
Incidence	Hungary	24.48127746	34.11840543	16.20117693
Incidence	Hungary	36.25055283	48.50029456	25.8169896
Incidence	Bahrain	4.696475998	7.018610403	2.923078147
Incidence	Bahrain	3.86828001	6.077164656	2.345225875
Incidence	Bahrain	4.418503057	6.582803625	2.792466875
Incidence	United Republic of Tanzania	1.610733961	2.400854473	0.995115122
Incidence	United Republic of Tanzania	0.990650987	1.510195211	0.574693384
Incidence	United Republic of Tanzania	1.287032882	1.903557117	0.801255743
Incidence	Cuba	15.15564843	22.24315791	9.922447097
Incidence	Cuba	6.306742277	8.876818043	4.19916998
Incidence	Cuba	10.62201901	14.89643588	7.354474176
Incidence	Mali	1.26813497	1.81149205	0.782732745
Incidence	Mali	0.502203313	0.836582357	0.238831393
Incidence	Mali	0.88295076	1.299919329	0.530503995
Incidence	Liberia	1.57268892	2.193519488	1.035715412
Incidence	Liberia	0.672954692	1.087456542	0.352634415
Incidence	Liberia	1.145495557	1.618590239	0.736932403
Incidence	Bhutan	2.47346208	3.680610202	1.505255643
Incidence	Bhutan	1.554769142	2.453412819	0.918723713

Incidence	Bhutan	2.035073662	3.063756701	1.251105486
Incidence	Botswana	2.054813875	3.093548974	1.242706732
Incidence	Botswana	1.092227095	1.699461334	0.623521407
Incidence	Botswana	1.565108707	2.333253891	0.957456647
Incidence	American Samoa	4.037260672	5.989098136	2.585061823
Incidence	American Samoa	1.864593844	2.885037333	1.110495417
Incidence	American Samoa	2.980322542	4.417312361	1.92591351
Incidence	Tokelau	2.628255105	4.016838229	1.639435356
Incidence	Tokelau	1.56421411	2.402545953	0.915253678
Incidence	Tokelau	2.107758213	3.221743407	1.313180668
Incidence	Honduras	9.686154945	13.51762887	6.447527525
Incidence	Honduras	4.5782605	6.80065602	2.903212774
Incidence	Honduras	6.978171031	9.72826238	4.751305481
Incidence	Belgium	29.55280687	42.32943296	19.66635696
Incidence	Belgium	6.579653526	10.3299432	4.096276666
Incidence	Belgium	18.04695255	25.68655429	12.18358898
Incidence	United Kingdom	19.7792074	26.9968516	13.66500498
Incidence	United Kingdom	10.11950157	14.29333547	6.798918759
Incidence	United Kingdom	14.87728877	20.52024288	10.24349431
Incidence	South Africa	2.56918416	3.837106893	1.578565963
Incidence	South Africa	1.181622035	1.82476179	0.695291484
Incidence	South Africa	1.838822184	2.768000172	1.122828947
Incidence	Sweden	29.64442112	42.33439132	19.47591312
Incidence	Sweden	14.76591266	21.88178564	9.161713421

Incidence	Sweden	22.28789299	31.91237092	14.34814723
Incidence	Uganda	1.407793969	2.108220206	0.854442996
Incidence	Uganda	0.858772698	1.338927117	0.495814744
Incidence	Uganda	1.117709357	1.673049417	0.68814657
Incidence	Egypt	4.914280881	7.125972549	3.184363259
Incidence	Egypt	3.336693811	5.14118334	1.993391646
Incidence	Egypt	4.161876235	6.074955063	2.670638285
Incidence	Mexico	14.02062021	20.01554122	9.317726961
Incidence	Mexico	6.812912671	10.01250446	4.391930683
Incidence	Mexico	10.21667864	14.72663036	6.733247181
Incidence	Vanuatu	2.243809058	3.349391788	1.435831951
Incidence	Vanuatu	1.125244558	1.751574252	0.645292076
Incidence	Vanuatu	1.682726132	2.518107259	1.060133204
Incidence	Argentina	16.89503099	24.18953302	10.87186318
Incidence	Argentina	9.139974808	13.55848272	6.014841573
Incidence	Argentina	12.8763484	17.54651754	8.796767786
Incidence	Palau	3.491407778	5.227067452	2.210932346
Incidence	Palau	1.789402877	2.760182018	1.074901302
Incidence	Palau	2.690743807	4.032712806	1.685783838
Incidence	Costa Rica	22.98528884	31.89767243	15.60155537
Incidence	Costa Rica	9.257015667	13.06462033	6.110979205
Incidence	Costa Rica	15.68179852	20.90888599	11.08278152
Incidence	El Salvador	10.38162699	14.74530791	6.753208627
Incidence	El Salvador	5.031755975	7.571121858	3.178457041

Incidence	El Salvador	7.264752145	10.25691685	4.80146941
Incidence	Republic of Moldova	19.78358013	28.50110407	12.82257034
Incidence	Republic of Moldova	8.563526996	12.74579939	5.52374689
Incidence	Republic of Moldova	13.75093528	19.70582237	9.025888217
Incidence	Guinea	1.661724218	2.288905267	1.114236515
Incidence	Guinea	0.584573507	0.929722683	0.305488216
Incidence	Guinea	1.086411472	1.52959008	0.70995721
Incidence	Grenada	15.29331926	22.25217704	10.2677358
Incidence	Grenada	3.584142222	5.565634945	2.217697068
Incidence	Grenada	9.658983448	14.01445115	6.566938376
Incidence	Libya	4.192592818	6.17505845	2.740607493
Incidence	Libya	3.043392463	4.561827144	1.89224745
Incidence	Libya	3.63511087	5.312378685	2.38850732
Incidence	Philippines	1.336041058	2.054784798	0.796824724
Incidence	Philippines	0.888952602	1.383682671	0.500719755
Incidence	Philippines	1.11209936	1.718114838	0.651183844
Incidence	Iran (Islamic Republic of)	4.313897083	6.30965046	2.750277656
Incidence	Iran (Islamic Republic of)	2.943984313	4.391709751	1.831848204
Incidence	Iran (Islamic Republic of)	3.634769216	5.351542962	2.302662213
Incidence	Puerto Rico	17.34363094	25.05224098	11.23082963
Incidence	Puerto Rico	7.505967097	11.28207006	4.760717099
Incidence	Puerto Rico	12.09925913	17.0748513	8.226652535
Incidence	Afghanistan	2.655681973	3.753084746	1.724087874
Incidence	Afghanistan	1.517487876	2.273064788	0.918966755

Incidence	Afghanistan	2.040716546	2.926007923	1.335053044
Incidence	India	1.987075295	3.006423277	1.230129264
Incidence	India	1.262195004	1.973717141	0.759975174
Incidence	India	1.626976014	2.483287559	0.996156509
Incidence	Switzerland	30.18447402	43.97034091	19.28957122
Incidence	Switzerland	12.40025633	18.55102978	7.962336716
Incidence	Switzerland	21.37072222	30.53580643	14.09575292
Incidence	Eritrea	1.15450697	1.718023867	0.703837388
Incidence	Eritrea	0.71293483	1.121584932	0.403427484
Incidence	Eritrea	0.929858241	1.390572914	0.576100448
Incidence	Finland	37.29449636	54.14997209	24.69772604
Incidence	Finland	15.63381507	23.23762575	9.556859478
Incidence	Finland	26.46973472	37.4671046	18.1444056
Incidence	Guatemala	8.24581495	11.65086939	5.456312441
Incidence	Guatemala	3.942409703	5.956759891	2.474700166
Incidence	Guatemala	5.939396755	8.497316567	3.99264524
Incidence	North Macedonia	20.25229451	29.27124209	13.48150069
Incidence	North Macedonia	12.43034564	17.539846	8.054916172
Incidence	North Macedonia	16.43229594	23.00180102	11.10104542
Incidence	Guyana	14.64093856	19.44102189	10.46565302
Incidence	Guyana	5.04440669	7.035562623	3.203108551
Incidence	Guyana	9.700367393	12.73725021	6.978739126
Incidence	Mauritania	1.676444158	2.354780183	1.067504977
Incidence	Mauritania	0.772829993	1.239981518	0.401833118

Incidence	Mauritania	1.202194804	1.714925019	0.751569492
Incidence	Lesotho	1.79926118	2.64020465	1.138446558
Incidence	Lesotho	0.907781717	1.417658837	0.506321194
Incidence	Lesotho	1.323867631	1.951835266	0.833434895
Incidence	Ethiopia	1.076865973	1.641143089	0.642233033
Incidence	Ethiopia	0.686054391	1.089999676	0.378040924
Incidence	Ethiopia	0.881498237	1.343989344	0.518303191
Incidence	France	23.28542268	33.45436086	14.42221467
Incidence	France	8.585080144	12.89035385	5.489722517
Incidence	France	15.72891152	22.32500008	10.26677496
Incidence	Kuwait	5.898961772	8.876730118	3.624715718
Incidence	Kuwait	4.329375823	6.582737915	2.621569921
Incidence	Kuwait	5.217468917	7.723728855	3.24702398
Incidence	Haiti	6.580713724	9.269220984	4.33646232
Incidence	Haiti	2.633115961	3.806310919	1.659205377
Incidence	Haiti	4.508211675	6.370956764	3.004996282
Incidence	Lebanon	5.499521111	8.183146911	3.604732368
Incidence	Lebanon	3.540236156	5.26863368	2.196515363
Incidence	Lebanon	4.453870788	6.509404923	2.954279549
Incidence	Germany	36.13640675	51.26352866	24.6084642
Incidence	Germany	12.69479381	18.42689793	7.976034024
Incidence	Germany	24.44154618	33.62435345	16.81816301
Incidence	Niger	1.285401759	1.820989558	0.831854885
Incidence	Niger	0.508533823	0.839589362	0.251973824

Incidence	Niger	0.873343253	1.273950254	0.549850467
Incidence	Tuvalu	2.561581437	3.807189697	1.626825996
Incidence	Tuvalu	1.369339005	2.119846279	0.817206087
Incidence	Tuvalu	1.970405943	2.913487055	1.243850589
